# Unusual conservation among genes encoding small secreted salivary gland proteins from a gall midge

**DOI:** 10.1186/1471-2148-10-296

**Published:** 2010-09-28

**Authors:** Ming-Shun Chen, Xuming Liu, Ziheng Yang, Huixian Zhao, Richard H Shukle, Jeffrey J Stuart, Scot Hulbert

**Affiliations:** 1 USDA-ARS, Hard Winter Wheat Genetics Research Unit, 4008 Throckmorton Hall, Kansas State University, Manhattan, KS 66506, USA; 2 Department of Entomology, Kansas State University, Manhattan, KS 66506, USA; 3 Department of Biology, University College London, London NW12HE, UK; 4 College of Life Sciences, Northwest A&F University, Yangling, Shaanxi, China; 5 USDA-ARS and Department of Entomology, Purdue University, West Lafayette, IN 47907, USA; 6 Department of Plant Pathology, Washington State University, Pullman, WA 99164, USA

## Abstract

**Background:**

In most protein-coding genes, greater sequence variation is observed in noncoding regions (introns and untranslated regions) than in coding regions due to selective constraints. During characterization of genes and transcripts encoding small secreted salivary gland proteins (SSSGPs) from the Hessian fly, we found exactly the opposite pattern of conservation in several families of genes: the non-coding regions were highly conserved, but the coding regions were highly variable.

**Results:**

Seven genes from the *SSSGP-1 *family are clustered as one inverted and six tandem repeats within a 15 kb region of the genome. Except for *SSSGP-1A2*, a gene that encodes a protein identical to that encoded by *SSSGP-1A1*, the other six genes consist of a highly diversified, mature protein-coding region as well as highly conserved regions including the promoter, 5'- and 3'-UTRs, a signal peptide coding region, and an intron. This unusual pattern of highly diversified coding regions coupled with highly conserved regions in the rest of the gene was also observed in several other groups of SSSGP-encoding genes or cDNAs. The unusual conservation pattern was also found in some of the SSSGP cDNAs from the Asian rice gall midge, but not from the orange wheat blossom midge. Strong positive selection was one of the forces driving for diversification whereas concerted homogenization was likely a mechanism for sequence conservation.

**Conclusion:**

Rapid diversification in mature SSSGPs suggests that the genes are under selection pressure for functional adaptation. The conservation in the noncoding regions of these genes including introns also suggested potential mechanisms for sequence homogenization that are not yet fully understood. This report should be useful for future studies on genetic mechanisms involved in evolution and functional adaptation of parasite genes.

## Background

Insect salivary glands are the main organs for producing proteins that are injected into hosts [1]. Plant-feeding insects, especially those with sucking mouthparts, inject proteins and other substances into host plants to facilitate mouthpart penetration, partially digest food before ingestion, and suppress plant defense [2-4]. Substances, including proteins with regulatory roles that can alter host physiology, are referred to as effectors [5]. Pathogens, including bacteria, fungi, oomycetes, and nematodes, deliver various effector proteins into host tissues [5-8]. Substantial evidence suggests that some of the salivary proteins injected into host plants by insects also act as effectors to suppress defense and/or reprogram physiological pathways of host plants [3,5,9-12]. Gall midges (Cecidomyiidae), a large family of plant-feeding insects, apparently secrete effectors into host tissues, inducing various forms of plant outgrowth (galls) and altering other aspects of host physiology [13,14]. Plant galls contain a zone of "metabolic habitat modification" in which the parasite experiences a selective advantage because of enhanced nutrition and reduced plant defense [15]. Several organic compounds and enzymes injected into host plants by galling insects have been identified, including amino acids, auxin, proteases, oxidases, and pectinases [13], but the general composition of the proteins delivered into host plants by gall midges has not yet been fully characterized.

The Hessian fly, *Mayetiola destructor*, is the most destructive insect pest of wheat worldwide [16]. Because of its importance in agriculture, intriguing behavior, ease of maintenance in culture, and relatively well-characterized genetics, Hessian fly is becoming a model species for studying insect-plant interactions [17,18]. Hessian fly does not induce the formation of an outgrowth gall, but nutritive cells with similarity to those inside macroscopic galls are formed at the larval feeding site [19]. Larvae do not cause extensive tissue damage to host plants, with their specialized mandibles making only a pair of small holes [19,20]. Nevertheless, wheat plants become permanently and irreversibly stunted after 4-5 days of feeding by a single larva [9]. Even if larvae are removed, growth of wheat seedlings cannot be restored [9,20], suggesting that larvae inject substances into host plants that dramatically alter biochemical and physiological pathways of the attacked plant [21,22].

As the first step to identify some of those proteins that are injected into host plants, we have previously generated numerous ESTs from cDNAs derived from dissected salivary glands of Hessian fly first instar larvae [23,24]. The majority of the salivary gland transcripts encode small proteins (50 to 200 amino acids) with typical secretion signal peptides at the N-termini. We refer to these proteins as "small secreted salivary gland proteins" (SSSGPs). Here we report unusual conservation patterns of SSSGP-encoding genes and we discuss potential mechanisms for gene evolution and functional adaptation.

## Results and Discussion

### Unconventional conservation of SSSGP-encoding genes

**Figure 1:**
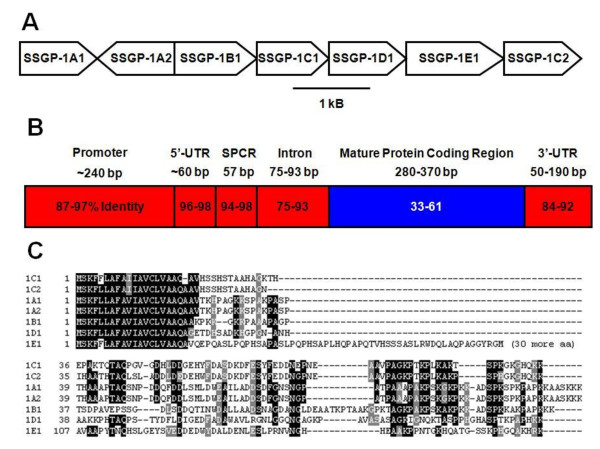
**Genomic organization and structural comparison of the Hessian fly *SSSGP-1 *family members**. **A: ***SSSGP-1 *family members derived by sequencing a BAC clone made from biotype GP. **B: **Nucleotide sequence comparison of the seven *SSSGP-1 *genes. Comparisons were divided into the promoter region (Promoter), 5'-untranslated region (5'-UTR), signal peptide coding region (SPCR), an intron, mature protein coding region (MPCR), and 3'-untranslated region (3'-UTR). The numbers in boxes are average scores and score range (in parentheses) derived from pair-wise comparisons of all possible combinations of the genes (see Materials and Methods). Red color indicates conserved regions. Blue color indicates diversified regions. The lowest score for any pairwise comparison in the MPCR was 13. Unrelated random sequences can produce scores as high as 15. The actual alignments of these genes are shown in Additional file 1, Figure S1A. **C: **Sequence alignments of putative proteins. Dashes represent gaps in the sequence alignments. The first 18 amino acids constitute a putative signal peptide.

The *SSSGP-1 *gene family includes seven members and is clustered as one inverted and six tandemly repeated genes within a 15 kb region of the genome (Figure 1A). The predicted structures of the genes were verified by comparing the genomic sequences with cDNA clones corresponding to genes *SSSGP-1A*, *SSSGP-1B1*, *SSSGP-1C1 *and *SSSGP-1D1 *(a cDNA for *SSSGP-1E1 *has yet to be identified). All seven genes have a common structure, including a conserved putative promoter region, a 5'-untranslated region (5'-UTR), a signal peptide-coding region (SPCR), an intron, a mature protein-coding region (MPCR), and a 3'-untranslated region (3'-UTR; Figure 1B). Intergenic regions are small, ranging from 0.2 to 1 kB (Genbank accession: GU196316). Among the seven genes, *SSSGP-1A2*, present in the inverted repeat, was apparently recently duplicated and encodes an identical protein with *SSSGP-1A1*. The other six genes consist of highly diversified MPCRs as well as highly conserved regions, including the promoter region, 5'- and 3'-UTRs, SPCR, and the intron (Figure 1B, Additional file 1, Figure S1A). The predicted proteins are almost identical in their putative signal peptides, but share little similarity among the mature proteins (Figure 1C). This extreme pattern of diversification in MPCR, which we refer to here as super-diversification, coupled with strong conservation in other regions was also observed in several other groups of SSSGP-encoding genes (Additional file 1, Figure S1) or cDNAs from Hessian fly (Table 1, Additional file 2, Figure S2).

**Table 1:** Similarity of different regions among cDNAs from different gene groups

Gene group	No. of Seq.	5'-Untranslated region (5'-UTR)			Signal peptide coding region (SPCR)			Mature protein coding region (MPCR)			3'-untranslated region (3'-UTR)		
		**Length**	**Score AVE**	**Score** **range**	**Length**	**Score AVE**	**Score** **range**	**Length**	**Score AVE**	**Score** **range**	**Length**	**Score AVE**	**Score** **range**

*SSSGP-1*	6	58 - 59	92	87 - 98	57	94	92 - 98	252 - 484	49	13 - 100	186 - 258	88	79 - 94

*SSSGP-2*	15	6 - 198	84	52 - 100	60	87	78 - 95	258 - 462	10	3 - 57	77 - 269	81	57 - 92

*SSSGP-3*	4	31 - 36	87	80 - 100	54	80	70 - 96	189 - 312	22	6 - 71	64 - 210	73	49 - 98

*SSSGP-4*	7	37 - 47	94	83 - 100	75	80	58 - 93	360 - 636	10	4 - 27	121 - 222	52	14 - 82

*SSSGP-5*	3	81 - 87	94	93 - 95	69	96	95 - 97	4 - 279	15	11 - 19	71 - 124	65	48 - 85

*SSSGP-6*	2	24 - 25	87	87	63	95	95	249 - 255	79	79	57 - 60	91	91

*SSSGP-31*	4	52 - 65	93	88 - 97	39	97	94 - 100	213 - 330	16	8 - 28	90 - 122	92	85 - 96

Score average (AVE) and score range were derived by pair-wise comparison (Materials and Methods). Except for the two members from group *SSSGP-6*, the score average for MPCR is at least 40% less than those for other regions. The two members from group *SSSGP-6 *are likely relatively recent duplicates since they share an overall 83% sequence identity. The score average for these two members is 13% less than those for other regions.

Except for the common features of diversification/conservation, there are no noticeable sequence or structural similarities between the different groups of *SSSGP *genes, and no apparent sequence similarities could be detected among different groups with currently available alignment methods such as BLAST. Most groups of *SSSGP *genes contain one intron (Additional file 1, Figures S1A, S1C, S1D). However, one group lacks introns (Additional file 1, Figure S1B) and several other groups contain multiple introns (Additional file 1, Figure S1E). For those genes containing introns, the first (or the sole) intron is located either at the boundary between the SPCR and MPCR, or within the SPCR (Additional file 1, Figure S1). The positions of intron/exon boundaries are generally conserved among members within a group. However, deletions or shifts in intron/exon boundaries occur in gene groups with multiple introns (Additional file 1, Figure S1E). For all gene groups, multiple members in each group are clustered within short chromosome regions in the Hessian fly genome (Additional file 3, Figure S3).

To determine if such a genetic phenomenon exists in other gall midges, a similar analysis of salivary gland cDNAs was conducted on two other related insects, the orange wheat blossom midge (*Sitodiplosis mosellana*) and the Asian rice gall midge (*Orseolia oryzae*). Approximately 8,500 cDNAs from the wheat blossom midge and 3,500 from the Asian rice gall midge were sequenced. In each case, a similar proportion (45-50%) of cDNA clones was found encoding different SSSGPs. Forty-eight different groups of putative SSSGPs were identified from the wheat blossom midge while 25 different groups of putative SSSGPs were identified from the Asian rice gall midge. Comparative analysis revealed that cDNAs and their encoded proteins from the Asian rice midge, wheat blossom midge, and Hessian fly were typically found to be species-specific; cDNAs from one species shared no detectable sequence similarity with those from the other two species, consistent with the rapidly evolving nature of SSSGP-encoding genes. The species-specific nature of SSSGP-encoding genes was further confirmed by PCR and by Southern blot analysis. No PCR amplification could be achieved using primer pairs designed according to cDNAs from another species. Similarly, no cross hybridization could be observed on Southern blots using cDNA probes from a different species (data not shown). The typical unconventional conservation pattern of SSSGP-encoding genes observed in Hessian fly was also found in some of the SSSGP-encoding transcripts of the Asian rice midge (Additional file 2, Figure S2G), but not in any transcripts of the wheat blossom midge. This observation indicates that the unconventional conservation of SSSGP-encoding genes might be linked to adaption to environmental changes such as a change in host plants. Even though they live on different plant species, the Asian rice midge and Hessian fly larvae share a similar feeding mechanism. Larvae of both species feed on the meristem of a leaf-sheath within a plant, and their survival strictly depends on their ability to induce the formation of nutritive cells of plant tissue at the feeding site, to inhibit plant growth, and to suppress host defense [17,19,25]. Wheat blossom midges, on the other hand, feed on developing wheat seeds and either do not require extensive manipulation of host plants such as growth inhibition [19], or manipulate host plants in different ways.

Several genes from different mosquito species have been found encoding diverse secreted salivary proteins and some of these genes are also organized as tandem repeats [26]. Diverse toxic small peptides have been found in the venoms of predatory cone snails [27]. However, the extreme cases described here with a very short (100 to 500 bp), highly diversified segment followed by a very short (~500 bp), highly conserved segment arranged as multiple tandem repeats has not been found in any other organisms.

### Strong positive selection on *SSSGP *loci and alleles

Strong positive (diversifying) selection appears to be one of the forces driving diversifications in MPCRs. Highly diversified members with less than 80% sequence identity within MPCRs did not produce meaningful alignments for analyzing nonsynonymous to synonymous substitution ratio (*dN/dS*), but the fact that the coding regions are hard to align is itself evidence for fast evolution by positive selection or other mechanisms such as Y-family polymerases [28]. Analysis of moderately diversified group members with 80 to 95% sequence identity in their MPCRs all yielded *dN/dS *above one (Table 2, Additional file 4, Figure S4). One pair of group members produced a *dN/dS *ratio above 18, indicating very strong positive selection. Due to the small size, similar sequences with greater than 95% sequence identity within MPCRs did not possess sufficient nucleotide substitutions to confidently discern evolutionary patterns through analyzing *dN/dS*. However, a different analysis of similar sequences derived from different alleles also produced strong evidence for positive selection (below).

**Table 2:** Evidence for positive selection on *SSSGP *group members

Group	cDNA pair	Codon	dN/dS	dN ± SE	dS ± SE
*SSSGP-1*	1C1/1C2	85	1.32	0.11 ± 0.03	0.08 ± 0.04

*SSSGP-2*	G19B4/S21C6	122	9.43	0.12 ± 0.02	0.01 ± 0.01

*SSSGP-2*	G10H7/G14E6	129	3.59	0.08 ± 0.02	0.02 ± 0.01

*SSSGP-2*	L4C4/G28G12	78	1.12	0.22 ± 0.04	0.20 ± 0.07

*SSSGP-4*	G13E6/G22C4	127	1.21	0.10 ± 0.02	0.08 ± 0.04

*SSSGP-4*	G2C8/L4H12	173	1.46	0.14 ± 0.02	0.10 ± 0.03

*SSSGP-4*	G16C10/G29D6	156	2.49	0.26 ± 0.03	0.11 ± 0.03

*SSSGP-4*	G9B3/L1C12	179	1.52	0.13 ± 0.02	0.09 ± 0.03

*SSSGP-6*	G7H5/G8C4	81	2.92	0.31 ± 0.05	0.11 ± 0.05

*SSSGP-7*	G10C11/G15G6	119	1.48	0.11 ± 0.02	0.07 ± 0.03

*SSSGP-10*	G8C1/G14G1	75	2.51	0.18 ± 0.04	0.07 ± 0.03

*SSSGP-26*	G4C3/S22E4	162	5.67	0.04 ± 0.01	0.00 ± 0.00

*SSSGP-31*	G7D10/S4A9	100	1.82	0.06 ± 0.02	0.03 ± 0.03

*SSSGP-37*	S5E9/G9G9	41	2.51	0.11 ± 0.04	0.04 ± 0.04

*SSSGP-79*	S14G9/S22D12	63	18.8	0.19 ± 0.04	0.01 ± 0.01

*SSSGP-80*	L7H8/S8C9	123	1.84	0.04 ± 0.01	0.03 ± 0.02

dN/dS ratio were obtained by comparing MPCRs using PAML42 [53].

Multiple transcripts corresponding to genes *SSSGP-1A1, SSSGP-1B1*, and *SSSGP-1C1 *were isolated from three different Hessian fly populations. These different transcripts were likely derived from different alleles since evidence from *in situ *hybridization, Southern blots with genomic and BAC DNA samples, and primer specific PCR suggests a single locus for this gene family (Additional file 5, Figure S5). The ratio between nonsynonymous and synonymous substitutions was 1.5 or more within the MPCR, but less than 0.9 in the SPCR (Table 3, Additional files 6 and 7), again indicating positive selection in MPCRs for different alleles.

**Table 3:** Evidence for positive selection on different alleles (Additional file 6, Figure S6)

Gene	Total cDNAs sequenced				Unique	Unique	Nonsyn/Syn	
	**GP**	**L**	**S**	**Total**	**cDNAs**	**proteins**	**SPCR**	**MPCR**

***SSSGP-1A1***	60	43	45	148	71	27	0.83 (5/6)	1.56 (28/18)

***SSSGP-1B1***	106	24	36	166	66	35	0.80 (4/5)	1.50 (21/14)

***SSSGP-1C1***	27	5	12	44	20	14	0.60 (3/5)	2.33 (21/9)

**RPs**	157	34	23	214	96	47	N/A	0.26 (50/195)

cDNAs were derived from three Hessian fly populations: biotype GP, biotype L, and a Syrian population (S). As a control, cDNAs coding for 26 different ribosomal proteins (RPs) that were isolated along with *SSSGP *cDNAs were included in this analysis. Sequence alignments for different *SSSGP *cDNAs are shown in Additional file 6, Figure S6 whereas alignments for RP cDNAs are shown in Additional file 7, Figure S7. "Nonsyn/Syn" represents the ratios of non-synonymous against synonymous substitutions in SPCR and MPCR, respectively.

Evidence for positive selection is not common but has been demonstrated at several different types of genes controlling interactions between organisms that are mediated by molecular recognition. Typical examples are defense-related genes including the major histocompatibility complex [29], immunoglobulins [30], defensins [31], plant resistance genes [32], plant chitinase genes [33], and pathogen effector genes [34]. The strong positive selection observed in SSSGP-encoding genes indicated that SSSGPs are also likely involved in interactions between Hessian fly and other organisms. Considering that Hessian fly larvae live within host plants, some of these SSSGPs may be secreted into host plants as effector proteins with a role in the insect's virulence. In plant-herbivore interactions, successful pathogens and parasitic arthropods not only require a large number of genes coding for effector proteins to suppress innate defense of host plants [35], but also require the ability to change this arsenal in response to shifts in the host population [36]. Evolution of plant populations in parasite recognition and surveillance systems thus provides strong selection for counter changes in effector proteins from parasites [36,37]. The Hessian fly has been very successful in adaptation to changes in host plant populations [16,17]. The super-diversification in SSSGP genes may have provided the genetic basis for the development of counter-resistance in Hessian fly in response to changes in host plants.

### Concerted homogenization of noncoding regions

**Figure 2:**
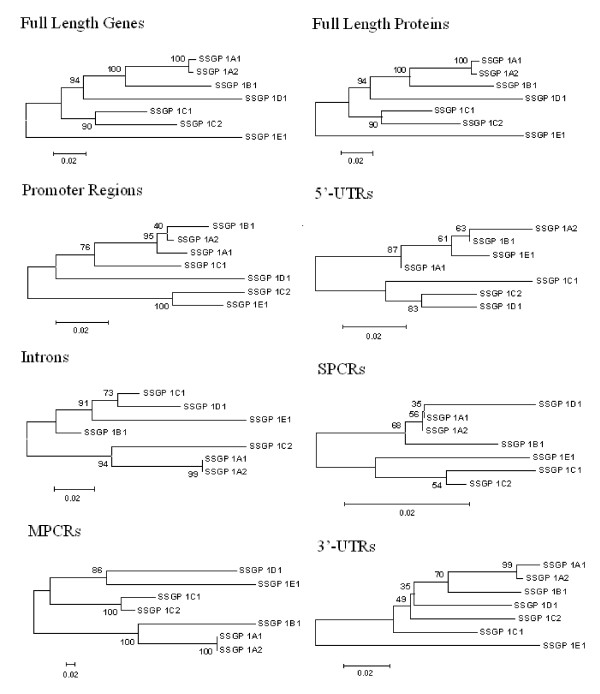
**Phylogenetic tree for different regions of *SSSGP-1 *family members inferred using the Neighbor-joining method implemented in MEGA**.

Very strong selection for divergence could account for rapid divergence of MPCR but the high homology of the other regions of the genes is difficult to explain. Recombination between gene-family members, particularly those arranged in tandem arrays, acts to homogenize their sequences so they evolve in a concerted fashion [38,39]. Typically, however, this homogenization occurs throughout the whole gene and even the intergenic regions, not just specific domains in the genes. While crossover events would tend to homogenize the whole array, smaller gene conversion events might homogenize smaller regions. Little is known about recombination in gall midges, but conversion tracts at the *Rosey *locus of *Drosophila *have been found to be in the order of a few hundred base pairs [40]. Differences in sequence affinity among the various sub-regions of the *SSSGP-1 *family members corroborate frequent recombination in short DNA regions during Hessian fly evolution (Figure 2). The homogenization could be confined to termini of the genes if the conversion events were initiated near the ends of the genes or in intergenic regions. The nature of recombination hotspots varies between species [41], but they are commonly initiated intergenically [42], possibly at specific sequence motifs [43] or regulatory regions. Sequence heterogeneity in the MPCR due to strong positive selection could, in turn, affect the length of conversion tracks or how the recombination intermediates are resolved; conversion or crossover events [44]. If the sequence homogeneity of the SSSGP-encoding families was caused by concerted evolution from short conversion tracks initiated in the flanking regions, one would expect introns in the middle of the larger genes to be less homogenized. This is in fact what was observed in the *SSSGP-2 *family; noticeably, several introns (introns 22, 23, 26, 27, 35, 36, 37) were rearranged or deleted (Additional file 1, Figure S1E). The coding regions of the two *SSSGP-2 *family members correspond to approximately 950 nucleotides with 35 introns.

**Figure 3:**
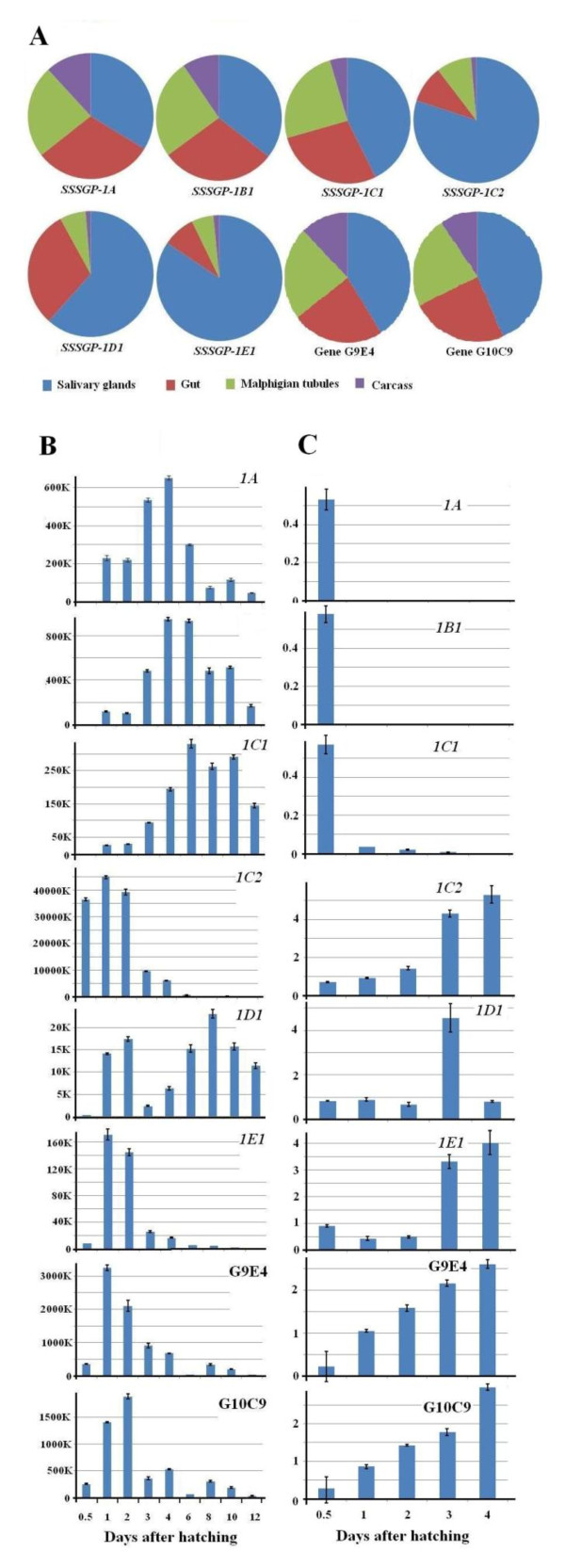
**Distribution and abundance of transcripts corresponding to specific *SSSGP-1 *family members**. **A**: Transcript distribution among tissues was determined using 3-day old biotype GP larvae. The remains after removing salivary glands, gut, and Malphigian tubules were designated as carcass. **B**: Transcript abundance in 0.5 to 12-day old larvae on susceptible wheat plants (cultivar 'Newton'). **C**: Transcript abundance in 0.5 to 4-day old (dying) larvae on resistant wheat (cultivar 'Molly' containing *H13 *R-gene). Primer pairs and methods are shown in Additional file 8, Table S1.

To explore whether functional adaptation might explain conservation of certain regions of gene families [45], we analyzed the patterns of transcript levels corresponding to specific genes under different conditions (Figure 3). In general, SSSGPs with higher sequence similarity in the promoter regions had more similar patterns of gene expression (Figures 2, 3). *SSSGP-1A1*, *SSSGP-1A2*, *SSSGP-1B1*, and *SSSGP-1C1*, whose promoters were very similar (Figure. 2C), also exhibited similar expression patterns among tissues (Figure 3A) and developmental stages (Figure 3B), and among insects interacting with different plant genotypes (Figure 3C). The promoters of *SSSGP-1C2*, and *SSSGP-1E1 *were also similar to each other (Figure 2C), and these two genes also exhibited similar transcription patterns. However, the genes in the first group (*SSSGP-1A1*, *SSSGP-1A2*, *SSSGP-1B1*, and *SSSGP-1C1*) and the second group (*SSSGP-1C2 *and *SSSGP-1E1*) had strikingly different expression patterns (Figure 3). Small differences in the transcription patterns among members in the same promoter group were also observed. For example, *SSSGP-1C2 *was expressed abundantly in 0.5-day old larvae (Figure 3B, *1C2*), whereas little *SSSGP-1E1 *expression was observed in the same larvae (Figure 3B, *1E1*). These differences could indicate that small differences in the promoter (or other regulatory elements in other regions) of the genes can fine-tune the level of transcripts to satisfy specific requirements. These observations suggest that the conservation/diversification of the promoter regions has been strictly driven by functional adaptation.

The homogenization of 5'- and possibly even 3'-UTRs may also have a functional basis. Because UTRs play critical roles in post-transcriptional regulation of gene expression [46,47], we speculate that the SSSGP UTRs are critical for proper post-transcriptional regulation. For example, part of the conserved UTRs could serve as elements for binding with regulatory proteins or as pairing sites for interacting with micro-RNAs that may affect RNA stability or translation efficiency [48]. Multiple layers of gene regulation may be needed to ensure spatial and tissue-specific expression and prompt response of SSSGP-encoding genes to changes of host and other environmental conditions.

### Functional division of SSSGPs: initiators and maintainers

SSSGPs appear to have a division of labor, with "initiators" expressed only immediately after the start of feeding and "maintainers" expressed at later stages in the time course of feeding and plant response. Initiators, such as *SSSGP-1C2 *and *SSSGP-1E *in the *SSSGP-1 *family, were predominantly expressed in salivary glands (Figure 3A) at early stage of larval development (Figure 3B), and their expression was elevated at later time points in larvae feeding on resistant plants (Figure 3C). These observations are consistent with the postulation that initiators are secreted into plant tissue as effectors to manipulate plant cells. Hessian fly suppresses plant defense and induces the formation of nutritive cells within the first couple of days [9,19]. Once the insect has successfully manipulated host plants, one would expect that the expression of initiators is no longer needed. Indeed the manipulation of wheat seedlings is irreversibly achieved within the first few days following the Hessian fly initial attack [9]. The elevated expression of initiators in larvae feeding on resistant plants at later stages may reflect the fact that Hessian fly larvae continue to secret effectors to counter increased plant defense in these plants [21,22].

Maintainers, such as *SSSGP-1A*, *SSSGP-1C1*, and *SSGP-1C1*, were also expressed in other tissues besides the salivary glands (Figure 3A). The proteins produced in Malphigian tubules and carcass are unlikely to play a role in interaction with host plants, but could play a role in regulating Hessian fly symbiotic or associated microbes in insect tissues [49]. In addition, some SSSGPs could also play a role in regulating secondary microbial infection of the host tissues damaged at the feeding site [50]. The maintainers may possess antimicrobial activity, and are under selection pressure from changes in microbial populations. Further research on the network of these initiators and maintainers encoded by rapidly evolving genes will shed light on the biology and feeding behavior of gall midges.

## Conclusion

In this study, we observed an unconventional conservation pattern in genes encoding SSSGPs in the Hessian fly. In the SSSGP-encoding genes, noncoding regions are highly conserved whereas regions coding for mature proteins are highly diversified. Rapid diversification in mature SSSGPs suggests that the genes are under selection pressure for functional adaptation. Considering the fact that most SSSGP-encoding genes are exclusively expressed in salivary glands, it is likely that rapid diversification in SSSGP-encoding genes is for the insect to counter changes in host plants for virulence. The conservation in the noncoding regions of these genes including introns also suggested potential mechanisms for sequence homogenization that are not yet fully understood. This report should be useful for future studies on genetic mechanisms involved in evolution and functional adaptation of parasite genes.

## Methods

### DNA libraries and sequencing

cDNA libraries and sequencing were as described previously [23,24]. A BAC library with 5× coverage was made from biotype GP Hessian fly larvae through a commercial contract with Amplicon Express (Pullman, WA). The BAC library contains inserts with average size of ~150 kB ligated into *Hind *III of pECBA1. A positive BAC clone, 10A23, was identified by screening the BAC library with a cDNA probe corresponding to the *SSSGP-1C1 *gene. A shotgun library with average sizes of 1.5 kB was made with 10 times coverage of the BAC clone 10A23, again through a commercial contract with Amplicon Express. The shotgun library was sequenced using ABI 3730 DNA analyzer at Kansas State University DNA sequencing facility. The shotgun sequences were assembled using Cap3 [51] and confirmed by PCR amplification and resequencing. The sequence of the whole BAC clone is 130 kB and was deposited into Genbank with accession number GU196316. The 15 kB cluster was located in the middle region toward 5'-end of the BAC.

### Quantitative real-time polymerase chain reaction (qRT-PCR) analysis

RNA extraction, reverse-transcription and real-time PCR were carried out as described previously [24]. Two hundred larvae or tissues from 200 larvae were collected and pooled for RNA isolation for each replicate. Three biological replicates were included for each analysis. The ratio between abundances on resistant plants and the corresponding ones on susceptible plants were calculated. Primers used for PCR reactions are listed in Additional file 8, Table S1.

### Sequence analysis and comparison

Sequence alignments and comparison were conducted using ClustalW [52]. For pairwise comparison, each sequence was compared with every other sequence. Scores for individual alignments are calculated based on the method of Wilbur and Lipman [53]. The higher the score is for a pairwise alignment, the higher the degree of conservation is between the two aligned sequences. Average scores were derived by dividing the sum of all pair-wise scores with the number of alignments. Score range was the lowest score to the highest score among all pair-wise alignments. For analysis of nucleotide substitutions, pair-wise alignments were obtained using ClusterW. Nonsynomonous (*dN*) and synomonous (*dS*) substitution ratios (*dN/dS*) were obtained using PAML42 [54].

Phylogenetic trees were produced based on neighbor joining and maximum likelihood using MEGA4 [55].

### Southern blot analysis

Hessian fly genomic DNA was isolated following a salting out protocol [56]. For Southern blot, 10 μg of purified genomic DNA was digested with individual restriction enzymes. The digested DNA fragments were separated on a 0.8% agarose gel and blotted onto GeneScreen membrane (Perkin Elmer, Beltsville, MD). The membranes were then hybridized separately to individual probes of cDNAs from either the Hessian fly, or Asian rice midge, or wheat blossom midge. cDNA probes were produced with ^32^P dCTP using a random labeling kit from Stratagene (La Jolla, CA). Hybridization was carried out overnight at 42°C in a plastic bag containing a 15-mL hybridization solution, which consisted of 10% dextran sulfate, 1% SDS, 1 M NaCl, pH 8.0. After hybridization, the membranes were washed twice with 2 × SSC at room temperature for 30 min, twice with 2 × SSC (0.3 M sodium chloride and 30 mM tri-Sodium citrate dihydrate, pH 7.0) plus 1% SDS at 65°C for 30 min, and twice with 0.1 × SSC plus 1% SDS at room temperature for 30 min. Images were visualized by exposing the membranes to Kodak SR-5 X-ray film overnight.

## Authors' contributions

MSC participated in sequence analysis and manuscript preparation. XL participated in library construction and sequence analysis. ZY involved in phylogenetic analysis and bioinformatics. HZ did real-time PCR analysis. RHS characterized cDNAs from rice midge and wheat midge. JJS and SH participated in data analysis and manuscript preparation. All authors have read and approved the final manuscript.

## Supplementary Material

Additional file 1 Figure S1: Sequence alignments of different groups of SSSGP-encoding genes. Click here for file

Additional file 2 Figure S2: Sequence alignments of different groups of SSSGP-encoding cDNAs. Click here for file

Additional file 3 Figure S3: Evidence for clustered organization of SSSGP-encoding genes. Click here for file

Additional file 4 Figure S4: Alignments of moderately diversified *SSSGP *group members (cDNAs). Click here for file

Additional file 5 Figure S5: Evidence for single location of genes in the *SSSGP-1 *family. Click here for file

Additional file 6 Figure S6: Sequence alignment of similar SSSGP-encoding cDNAs (presumably derived from different alleles). Click here for file

Additional file 7 Figure S7: Sequence alignment of cDNAs encoding ribosomal proteins. Click here for file

Additional file 8 Table S1: Primers used for PCR reactions. Click here for file
