# Supplementary material for: Unusual conservation among genes encoding small secreted salivary gland proteins from a gall midge
Source: BMC Evol Biol. 2010 Sep 28;10:296. doi: 10.1186/1471-2148-10-296 (PMC2955719; doi:10.1186/1471-2148-10-296)
Supplement: Additional file 1 — Figure S1: Sequence alignments of different groups of SSSGP-encoding genes. [file 1471-2148-10-296-S1.DOC]

**A**

| SSSGP_1C1 | - | - | - | - | - | - | - | - | G | T | G | T | A | T | G | T | G | A | G | T | T | T | G | T | G | T | A | A | A | C | G | T | T | C | C | G | T | A | T | T | T | A | A | A | T | A | - | A | T | A | T | A | T | T | A | T | A | C | A | T |
| --- | --- | --- | --- | --- | --- | --- | --- | --- | --- | --- | --- | --- | --- | --- | --- | --- | --- | --- | --- | --- | --- | --- | --- | --- | --- | --- | --- | --- | --- | --- | --- | --- | --- | --- | --- | --- | --- | --- | --- | --- | --- | --- | --- | --- | --- | --- | --- | --- | --- | --- | --- | --- | --- | --- | --- | --- | --- | --- | --- | --- |
| SSSGP_1C2 | - | - | - | - | - | - | - | - | - | - | - | - | - | - | - | - | - | - | - | - | - | - | - | - | - | - | - | - | - | - | - | - | - | - | - | - | - | - | - | - | - | - | - | - | - | - | - | - | - | - | - | - | - | - | - | - | - | - | - | - |
| SSSGP-1A1 | - | - | - | - | - | - | - | - | - | - | - | - | A | A | T | G | A | A | A | A | T | G | C | T | A | T | A | A | A | T | G | A | G | A | T | G | C | A | A | A | T | A | A | T | T | A | T | G | T | G | G | A | A | A | G | C | A | C | T | G |
| SSSGP_1A2 | - | - | - | - | - | - | - | - | - | - | - | - | - | - | - | - | - | - | A | A | T | G | C | T | A | T | A | A | A | T | G | A | G | A | T | G | C | A | A | A | T | A | A | T | T | A | T | G | T | G | G | A | A | A | G | C | A | C | T | G |
| SSSGP_1D1 | T | A | T | A | A | T | T | T | T | T | G | C | A | A | G | A | A | A | A | A | T | G | C | T | A | T | A | A | A | - | - | - | - | - | T | G | T | A | A | A | T | A | A | T | T | A | G | G | A | C | G | A | T | A | G | C | A | C | T | G |
| SSSGP_1B1 | - | - | - | - | - | - | - | T | T | G | G | C | A | A | A | C | A | A | A | A | T | G | C | T | A | T | A | A | A | T | G | A | G | T | T | G | C | A | A | A | T | A | T | T | T | A | T | G | T | G | G | A | A | A | G | C | A | C | T | G |
| SSSGP-1E1 | - | - | - | - | - | - | - | - | - | - | - | - | T | A | A | T | G | C | G | A | T | T | C | T | G | A | A | A | A | T | - | T | C | A | T | A | G | A | T | A | A | A | A | G | T | G | A | A | T | G | G | C | A | A | A | A | T | T | C | A |
|  |  |  |  |  |  |  |  |  |  |  |  |  |  |  |  |  |  |  |  |  |  |  |  |  |  |  |  |  |  |  |  |  |  |  |  |  |  |  |  |  |  |  |  |  |  |  |  |  |  |  |  |  |  |  |  |  |  |  |  |  |
| SSSGP_1C1 | C | T | A | T | C | A | A | T | G | T | T | T | T | C | A | C | C | T | G | A | T | C | C | C | A | A | G | G | A | G | A | A | T | T | C | C | C | C | A | T | T | T | A | A | T | A | A | A | A | T | A | - | - | - | A | A | A | A | A | A |
| SSSGP_1C2 | - | - | - | - | - | - | - | - | - | - | - | - | - | - | - | - | - | - | - | - | - | - | - | - | - | - | - | - | - | - | T | T | A | T | T | T | C | T | A | T | T | T | T | G | T | T | A | A | T | T | T | - | - | - | A | A | A | A | A | A |
| SSSGP-1A1 | A | G | C | C | C | T | T | T | C | T | T | T | T | A | A | T | C | G | A | A | A | A | A | C | A | A | A | A | A | A | A | A | A | A | A | A | T | A | A | A | A | A | G | A | G | T | A | T | T | T | T | T | A | A | A | A | A | A | A | A |
| SSSGP_1A2 | A | G | C | C | C | T | T | T | C | T | T | T | T | A | A | T | C | G | A | A | A | A | A | C | A | A | A | A | A | A | A | - | - | - | A | A | T | A | A | A | A | A | G | A | A | T | A | T | T | T | T | - | - | A | A | A | A | A | A | A |
| SSSGP_1D1 | A | G | C | C | C | T | T | T | A | T | G | T | - | A | A | T | C | - | - | - | A | A | A | C | A | A | A | G | A | A | A | A | A | T | A | A | A | A | A | A | A | T | A | A | A | C | A | A | T | T | T | T | - | - | A | A | A | A | A | G |
| SSSGP_1B1 | A | G | C | C | C | T | T | T | C | T | T | T | T | C | A | T | C | - | - | - | A | A | A | C | A | A | T | A | A | A | A | A | A | A | A | A | T | A | G | A | A | G | C | A | G | C | A | T | T | C | T | T | - | - | A | A | A | A | A | A |
| SSSGP-1E1 | T | T | T | T | C | A | T | T | G | C | G | T | T | C | A | A | A | T | A | A | T | G | A | T | T | A | T | T | A | T | T | A | T | C | G | A | T | A | G | A | A | T | G | C | G | A | C | C | A | C | T | G | A | G | A | T | T | A | A | A |
|  |  |  |  |  |  |  |  |  |  |  |  |  |  |  |  |  |  |  |  |  |  |  |  |  |  |  |  |  |  |  |  |  |  |  |  |  |  |  |  |  |  |  |  |  |  |  |  |  |  |  |  |  |  |  |  |  |  |  |  |  |
| SSSGP_1C1 | G | T | G | A | T | C | A | T | T | C | T | T | G | T | A | A | T | A | C | G | G | A | T | T | A | T | C | G | A | T | C | T | C | A | A | A | A | T | T | G | C | G | A | T | T | A | C | A | T | G | A | A | C | G | T | G | C | T | G | G |
| SSSGP_1C2 | G | G | G | C | T | C | A | G | T | - | - | - | - | - | - | - | - | - | - | - | - | G | C | T | A | T | C | G | A | T | A | T | - | - | - | A | A | T | T | A | T | T | T | G | T | A | T | A | T | - | - | - | C | A | T | C | T | T | T | A |
| SSSGP-1A1 | G | T | A | A | T | C | A | T | T | C | T | T | G | T | A | A | T | A | C | G | G | A | T | T | A | A | C | G | A | T | C | C | C | A | A | A | A | T | T | G | C | G | A | T | A | A | C | A | T | G | A | A | C | A | T | G | T | T | A | C |
| SSSGP_1A2 | G | T | A | A | T | C | A | T | T | C | T | T | G | T | T | A | T | A | T | G | G | A | T | T | A | T | C | G | A | T | A | T | C | A | A | A | A | T | T | G | C | G | A | T | A | A | C | A | T | G | A | A | C | A | T | G | T | T | A | C |
| SSSGP_1D1 | G | T | T | A | T | C | A | T | T | C | T | T | G | T | A | A | T | A | C | G | G | A | T | A | A | C | C | G | A | T | C | T | C | A | A | A | - | T | T | A | C | G | A | T | C | A | C | A | T | G | A | A | C | G | T | G | T | T | G | C |
| SSSGP_1B1 | G | T | A | A | T | C | A | T | T | C | T | T | G | T | A | A | T | A | C | G | G | A | T | T | A | A | C | G | A | T | C | T | C | A | A | A | A | T | T | G | C | G | A | T | T | A | C | A | T | G | A | A | C | G | T | G | T | T | A | C |
| SSSGP-1E1 | T | C | A | A | T | G | A | A | T | C | T | T | T | G | A | A | T | A | C | G | G | A | T | T | A | C | C | G | A | T | C | T | C | A | A | A | A | T | T | A | C | G | A | T | T | A | C | A | T | G | A | A | A | G | T | A | T | T | G | C |
|  |  |  |  |  |  |  |  |  |  |  |  |  |  |  |  |  |  |  |  |  |  |  |  |  |  |  |  |  |  |  |  |  |  |  |  |  |  |  |  |  |  |  |  |  |  |  |  |  |  |  |  |  |  |  |  |  |  |  |  |  |
| SSSGP_1C1 | A | A | A | T | T | T | A | C | A | T | G | T | T | T | T | G | T | A | A | T | C | G | G | C | A | A | - | - | A | A | A | G | A | C | A | A | T | T | G | C | C | G | G | A | C | T | T | T | G | A | T | G | A | G | C | A | T | A | A | A |
| SSSGP_1C2 | G | C | A | T | T | T | T | T | A | T | C | G | C | A | A | A | A | A | A | A | A | T | A | C | A | A | T | G | A | A | A | G | A | T | T | A | C | T | G | C | C | G | A | A | C | T | C | T | G | A | T | G | A | C | C | A | T | A | A | A |
| SSSGP-1A1 | A | A | G | T | T | T | A | C | A | A | G | C | A | T | T | G | T | A | A | T | C | A | G | C | A | A | - | - | A | A | A | G | A | T | A | A | T | T | G | C | C | G | G | A | C | T | C | T | G | T | T | G | A | G | C | A | T | A | A | A |
| SSSGP_1A2 | A | A | G | T | T | T | A | C | A | A | G | C | A | T | T | G | T | A | A | T | C | G | G | C | A | A | - | - | A | A | A | G | A | C | A | A | T | T | G | C | C | G | G | A | C | T | C | T | G | T | T | G | A | G | C | A | T | A | A | A |
| SSSGP_1D1 | A | A | A | T | T | T | A | C | A | A | G | T | T | T | A | A | T | A | T | T | T | G | G | C | A | A | - | - | A | A | G | G | A | A | T | A | C | T | G | C | C | G | G | A | C | T | C | T | G | A | T | G | A | T | C | A | T | A | A | A |
| SSSGP_1B1 | A | A | A | T | T | T | A | C | A | A | G | C | A | T | T | T | T | A | A | T | C | G | G | C | A | A | - | - | A | A | A | G | A | C | A | A | T | T | G | C | C | G | G | A | C | T | C | T | G | A | T | G | A | G | C | A | T | A | A | A |
| SSSGP-1E1 | A | A | A | A | T | T | A | C | A | A | G | T | T | T | T | A | T | A | A | C | C | G | G | C | A | A | - | A | A | A | A | A | A | C | T | A | C | T | G | C | C | A | G | A | C | T | C | T | G | T | T | G | A | T | C | A | T | A | A | A |
|  |  |  |  |  |  |  |  |  |  |  |  |  |  |  |  |  |  |  |  |  |  |  |  |  |  |  |  |  |  |  |  |  |  |  |  |  |  |  |  |  |  |  |  |  |  |  |  |  |  |  |  |  |  |  |  |  |  |  |  |  |
| SSSGP_1C1 | A | T | C | A | T | T | T | C | C | G | T | - | - | - | T | A | A | T | C | A | C | G | - | A | A | T | C | A | T | C | G | A | T | T | C | A | G | A | T | G | A | A | A | T | T | G | A | A | T | T | T | C | A | A | G | T | T | T | T | C |
| SSSGP_1C2 | A | T | C | A | T | A | T | T | C | G | T | - | - | - | T | A | A | T | C | A | C | A | T | A | A | T | C | A | T | C | G | A | T | T | T | A | G | A | T | G | A | A | A | T | T | G | A | A | T | T | T | C | A | A | G | T | T | T | T | T |
| SSSGP-1A1 | A | T | C | A | T | T | C | T | C | G | T | A | T | C | T | G | A | T | C | A | C | G | - | A | A | T | C | A | T | C | G | A | T | T | C | A | G | A | T | G | A | A | A | T | T | G | A | A | T | T | G | C | A | A | G | T | T | T | T | T |
| SSSGP_1A2 | A | T | C | A | T | T | C | T | C | G | T | A | T | C | T | G | A | T | C | A | C | G | - | A | A | T | C | A | T | C | G | A | T | T | C | A | G | A | T | G | A | A | A | T | T | G | A | A | T | T | G | C | A | A | G | T | T | T | T | T |
| SSSGP_1D1 | A | T | C | A | T | T | T | T | C | G | T | - | - | - | T | A | T | T | C | A | C | G | - | A | A | T | C | A | T | C | G | A | T | T | T | A | G | A | T | G | A | A | A | T | T | G | A | A | T | T | T | C | A | T | G | T | T | T | T | T |
| SSSGP_1B1 | A | T | C | A | T | T | C | T | C | G | T | A | T | C | T | G | A | T | C | A | C | G | - | A | A | T | C | A | T | C | G | A | T | T | C | A | G | A | T | G | A | A | A | T | T | G | A | A | T | T | G | C | A | A | G | T | T | T | T | T |
| SSSGP-1E1 | A | T | C | A | T | T | T | T | C | G | T | - | - | - | T | A | A | T | C | A | C | A | T | A | A | T | C | A | T | C | G | A | T | T | T | A | G | A | T | G | A | A | A | T | T | G | A | A | T | T | T | C | A | A | G | T | T | T | T | T |
|  |  |  |  |  |  |  |  |  |  |  |  |  |  |  |  |  |  |  |  |  |  |  |  |  |  |  |  |  |  |  |  |  |  |  |  |  |  |  |  |  |  |  |  |  |  |  |  |  |  |  |  |  |  |  |  |  |  |  |  |  |
| SSSGP_1C1 | G | T | C | A | C | A | A | G | A | C | A | T | T | T | T | T | C | C | A | A | A | T | A | C | A | G | A | T | G | T | T | G | T | A | C | G | T | G | T | T | C | G | T | C | A | A | A | T | G | A | C | A | A | T | A | C | G | T | T | G |
| SSSGP_1C2 | G | T | C | A | C | A | A | A | A | C | A | T | T | C | G | T | C | C | A | G | G | T | A | C | A | G | A | T | G | T | T | G | T | A | C | G | T | A | T | T | C | G | T | C | A | A | A | T | G | A | C | A | A | T | A | T | G | T | G | G |
| SSSGP-1A1 | G | T | C | G | C | A | A | A | A | C | A | T | T | T | T | T | C | A | A | A | A | T | A | C | A | G | A | T | G | T | T | G | T | A | C | G | T | A | T | T | T | G | T | C | A | A | A | T | G | A | C | A | A | T | A | C | G | T | G | G |
| SSSGP_1A2 | G | T | C | G | C | A | A | A | A | C | A | T | T | T | T | T | C | A | A | A | A | T | A | C | A | G | A | T | G | T | T | G | T | A | C | G | T | A | T | T | C | G | T | C | A | A | A | T | G | A | C | A | A | T | A | C | G | T | G | G |
| SSSGP_1D1 | G | T | C | A | C | A | A | G | G | C | A | T | T | T | T | T | C | C | A | G | A | T | A | C | G | G | A | T | G | T | T | G | T | A | C | G | T | G | T | T | C | G | T | C | A | A | A | T | G | G | C | A | A | T | A | C | T | T | G | A |
| SSSGP_1B1 | G | T | C | G | C | A | A | A | A | A | T | T | T | T | T | T | C | A | A | A | A | T | A | C | A | G | A | T | G | T | T | G | T | A | C | G | T | A | T | T | T | G | T | C | A | A | A | T | G | A | C | A | A | T | A | C | G | T | G | G |
| SSSGP-1E1 | G | T | C | A | C | A | A | A | A | C | A | T | T | C | G | T | C | C | A | G | G | T | A | C | A | G | A | T | G | T | T | G | T | A | C | G | T | A | T | T | T | G | T | C | A | A | A | T | G | A | C | A | A | T | A | T | G | T | G | G |
|  |  |  |  |  |  |  |  |  |  |  |  |  |  |  |  |  |  |  |  |  |  |  |  |  |  |  |  |  |  |  |  |  |  |  |  |  |  |  |  |  |  |  |  |  |  |  |  |  |  |  |  |  |  |  |  |  |  |  |  |  |
| SSSGP_1C1 | A | T | A | G | A | A | T | T | G | T | T | C | C | A | C | G | T | T | G | G | C | T | C | A | A | A | A | C | A | A | A | C | A | T | C | A | T | T | A | A | A | A | T | T | A | A | A | C | G | A | T | T | C | A | A | T | G | G | A | A |
| SSSGP_1C2 | A | T | A | G | A | A | T | T | G | A | T | C | C | A | C | G | T | T | G | G | C | T | C | A | A | A | A | C | A | A | A | C | A | T | T | A | T | - | - | A | C | A | T | A | G | A | C | A | T | A | T | T | C | A | T | T | G | G | A | A |
| SSSGP-1A1 | A | T | A | G | A | A | T | T | G | T | T | C | C | A | C | G | T | T | G | G | C | T | C | A | A | A | A | C | A | A | A | C | A | T | C | A | T | T | A | A | A | A | T | G | A | A | A | C | G | A | T | T | C | A | A | C | G | G | A | A |
| SSSGP_1A2 | A | T | A | G | A | A | T | T | G | T | T | C | C | A | C | G | T | T | G | G | C | T | C | A | A | A | A | C | A | A | A | C | A | T | C | A | T | T | A | A | A | A | T | G | A | A | A | C | G | A | T | T | C | A | A | C | G | G | A | A |
| SSSGP_1D1 | A | T | G | G | A | A | T | G | G | A | T | C | C | A | T | G | T | T | G | G | C | - | C | A | A | A | A | C | A | A | A | C | A | T | C | A | T | T | A | A | A | A | T | G | A | A | A | C | G | A | T | T | C | A | A | C | G | G | A | A |
| SSSGP_1B1 | A | T | A | G | A | A | T | T | G | T | T | C | C | A | C | G | T | T | G | G | C | T | C | A | A | A | A | C | A | A | A | C | A | T | C | A | T | T | A | A | A | A | T | G | A | A | A | C | G | A | T | T | C | A | A | C | G | G | A | A |
| SSSGP-1E1 | A | T | A | G | A | A | T | T | G | A | T | C | C | A | C | G | T | T | G | G | C | - | T | A | A | A | A | C | A | A | A | C | A | T | T | A | T | - | - | A | C | A | T | A | G | A | C | A | T | A | T | T | C | A | T | T | G | G | A | A |

**Putative TATA box ├**5’-UTR

| SSSGP_1C1 | T | G | C | G | A | T | G | G | A | T | G | G | A | A | A | A | A | T | T | T | T | G | C | **T** | **A** | **T** | **A** | **A** | A | A | A | T | G | G | A | T | G | G | A | T | C | G | G | C | A | C | C | T | A | C | A | A | T | T | C | A | **C** | A | G | T |
| --- | --- | --- | --- | --- | --- | --- | --- | --- | --- | --- | --- | --- | --- | --- | --- | --- | --- | --- | --- | --- | --- | --- | --- | --- | --- | --- | --- | --- | --- | --- | --- | --- | --- | --- | --- | --- | --- | --- | --- | --- | --- | --- | --- | --- | --- | --- | --- | --- | --- | --- | --- | --- | --- | --- | --- | --- | --- | --- | --- | --- |
| SSSGP_1C2 | T | G | C | G | A | T | G | G | A | T | G | G | G | A | A | A | A | T | T | T | T | G | A | **T** | **A** | **T** | **A** | **A** | G | A | A | T | G | G | A | T | T | G | A | T | C | G | G | C | A | T | C | T | A | T | A | A | T | T | C | A | **C** | A | G | T |
| SSSGP-1A1 | T | G | C | G | A | T | G | G | A | T | G | G | G | A | A | A | A | T | T | T | T | G | C | **T** | **A** | **T** | **A** | **A** | A | A | A | T | G | G | A | T | T | G | A | T | C | G | G | C | A | T | C | T | A | T | A | A | T | T | C | A | **C** | A | G | T |
| SSSGP_1A2 | T | G | C | G | A | T | G | G | A | T | G | G | G | A | A | A | A | T | T | T | T | G | C | **T** | **A** | **T** | **A** | **A** | A | A | A | T | G | G | A | T | G | G | A | T | C | G | G | C | A | C | C | T | A | C | A | A | T | T | C | A | **C** | A | G | T |
| SSSGP_1D1 | T | G | C | G | A | T | G | G | A | T | G | G | G | A | A | T | A | T | T | T | T | G | C | **T** | **A** | **T** | **A** | **A** | A | A | A | T | G | G | A | T | G | A | A | T | C | G | G | C | A | C | C | T | A | C | A | A | T | T | C | A | **C** | A | G | T |
| SSSGP_1B1 | T | G | C | G | A | T | G | G | A | T | G | G | G | A | A | A | A | T | T | T | T | G | C | **T** | **A** | **T** | **A** | **A** | A | A | A | T | G | G | A | T | G | G | A | T | C | A | G | C | A | C | C | T | A | C | A | A | T | T | C | A | **C** | A | G | T |
| SSSGP-1E1 | T | G | C | G | A | T | G | G | A | T | G | G | G | A | A | A | A | T | T | T | G | G | C | **T** | **A** | **T** | **A** | **A** | A | A | A | T | G | G | A | T | G | G | A | T | C | G | G | C | A | T | C | T | A | T | A | A | T | T | C | A | **C** | A | G | T |

**├** SPCR

| SSSGP_1C1 | T | C | C | A | A | T | T | G | A | A | C | C | G | T | T | T | A | T | T | C | T | T | T | C | G | A | T | A | T | C | C | A | C | T | G | G | A | A | C | A | T | C | C | A | A | A | A | A | - | C | G | A | A | A | **A** | **T** | **G** | T | C | A |
| --- | --- | --- | --- | --- | --- | --- | --- | --- | --- | --- | --- | --- | --- | --- | --- | --- | --- | --- | --- | --- | --- | --- | --- | --- | --- | --- | --- | --- | --- | --- | --- | --- | --- | --- | --- | --- | --- | --- | --- | --- | --- | --- | --- | --- | --- | --- | --- | --- | --- | --- | --- | --- | --- | --- | --- | --- | --- | --- | --- | --- |
| SSSGP_1C2 | T | C | G | A | A | T | T | A | A | A | C | C | G | T | T | A | A | T | T | C | T | T | T | C | A | A | T | A | T | C | C | A | C | T | G | G | A | A | C | A | T | C | C | A | A | A | A | A | A | C | G | A | A | A | **A** | **T** | **G** | T | C | A |
| SSSGP-1A1 | T | C | A | A | T | T | C | G | A | A | C | C | G | T | T | A | A | T | T | C | T | T | T | C | A | A | T | A | T | C | C | A | C | T | G | G | A | A | C | A | T | C | C | A | A | A | A | A | - | C | G | A | A | A | **A** | **T** | **G** | T | C | A |
| SSSGP_1A2 | T | C | A | A | T | T | C | G | A | A | C | C | A | T | T | A | A | T | A | C | T | T | T | C | A | A | T | A | T | C | C | A | C | T | G | G | A | A | C | A | T | C | C | A | A | A | A | A | - | C | G | A | A | A | **A** | **T** | **G** | T | C | A |
| SSSGP_1D1 | T | C | G | A | A | T | T | G | A | G | C | C | G | T | T | A | A | T | T | C | T | T | T | C | A | A | T | A | T | C | C | A | C | T | G | G | A | A | C | A | T | C | C | A | A | A | A | A | - | C | G | A | A | A | **A** | **T** | **G** | T | C | A |
| SSSGP_1B1 | T | C | A | A | T | T | C | G | A | A | C | C | A | T | T | A | A | T | T | C | T | T | T | C | A | A | T | A | T | C | C | A | C | T | G | G | A | A | C | A | T | C | C | A | A | A | A | A | - | C | G | A | A | A | **A** | **T** | **G** | T | C | A |
| SSSGP-1E1 | T | C | A | A | T | T | C | G | A | A | C | C | A | T | T | A | A | T | T | C | T | T | T | C | G | A | T | A | T | C | C | A | C | T | G | G | A | A | C | A | T | C | C | A | A | A | A | A | - | C | G | A | A | A | **A** | **T** | **G** | T | C | A |

**├ MPCR**

**├ Intron 1**

| SSSGP_1C1 | A | A | A | T | T | T | T | T | C | T | T | A | G | C | T | T | T | C | G | C | C | A | T | C | A | T | C | G | C | C | G | T | C | T | G | C | C | T | T | G | T | T | G | C | A | G | C | T | C | A | G | **G** | **T** | A | A | A | T | A | A | T |
| --- | --- | --- | --- | --- | --- | --- | --- | --- | --- | --- | --- | --- | --- | --- | --- | --- | --- | --- | --- | --- | --- | --- | --- | --- | --- | --- | --- | --- | --- | --- | --- | --- | --- | --- | --- | --- | --- | --- | --- | --- | --- | --- | --- | --- | --- | --- | --- | --- | --- | --- | --- | --- | --- | --- | --- | --- | --- | --- | --- | --- |
| SSSGP_1C2 | A | A | A | T | T | T | T | T | A | T | T | A | G | C | T | T | T | C | G | C | C | A | T | C | A | T | C | G | C | C | G | T | C | T | G | C | C | T | T | G | T | T | G | C | A | G | C | T | C | A | G | **G** | **T** | A | A | A | T | A | A | T |
| SSSGP-1A1 | A | A | A | T | T | T | T | T | A | C | T | A | G | C | T | T | T | C | G | C | C | G | T | C | A | T | C | G | C | C | G | T | C | T | G | C | C | T | T | G | T | T | G | C | A | G | C | T | C | A | G | **G** | **T** | A | A | A | T | A | A | C |
| SSSGP_1A2 | A | A | A | T | T | T | T | T | A | C | T | A | G | C | T | T | T | C | G | C | C | G | T | C | A | T | C | G | C | C | G | T | C | T | G | C | C | T | T | G | T | T | G | C | A | G | C | T | C | A | G | **G** | **T** | A | A | A | T | A | A | C |
| SSSGP_1D1 | A | A | A | T | T | T | T | T | A | C | T | A | G | C | T | T | T | C | G | C | C | G | T | C | A | T | C | G | C | T | G | T | C | T | G | C | C | T | T | G | T | T | G | C | A | G | C | T | C | A | G | **G** | **T** | A | A | A | T | A | A | T |
| SSSGP_1B1 | A | A | A | T | T | T | T | T | A | C | T | A | G | C | T | T | T | C | G | C | C | G | T | C | A | T | C | G | C | C | G | T | C | T | G | C | C | T | T | G | T | C | G | C | A | G | C | T | C | A | G | **G** | **T** | A | A | A | T | A | A | T |
| SSSGP-1E1 | A | A | A | T | T | T | T | T | C | T | T | A | G | C | T | T | T | C | G | C | C | G | T | C | A | T | C | G | C | C | G | T | C | T | G | C | C | T | T | G | T | A | G | C | A | G | C | T | C | A | G | **G** | **T** | A | A | A | T | A | A | T |

| SSSGP_1C1 | A | A | T | A | A | A | C | A | A | A | A | T | T | C | T | C | A | C | A | T | T | C | A | T | T | T | - | A | T | T | G | A | G | T | G | G | C | T | G | G | A | T | T | C | C | A | C | T | T | T | G | G | T | T | T | G | T | T | C | C |
| --- | --- | --- | --- | --- | --- | --- | --- | --- | --- | --- | --- | --- | --- | --- | --- | --- | --- | --- | --- | --- | --- | --- | --- | --- | --- | --- | --- | --- | --- | --- | --- | --- | --- | --- | --- | --- | --- | --- | --- | --- | --- | --- | --- | --- | --- | --- | --- | --- | --- | --- | --- | --- | --- | --- | --- | --- | --- | --- | --- | --- |
| SSSGP_1C2 | A | A | C | A | T | A | - | A | A | A | T | T | T | T | C | A | A | C | A | C | T | C | A | T | T | T | T | A | T | T | G | T | G | T | G | G | C | T | G | G | A | T | T | T | C | A | T | T | T | T | G | G | T | T | A | C | A | T | T | C |
| SSSGP-1A1 | A | T | - | - | - | - | A | A | A | A | T | T | T | T | C | A | A | C | A | C | T | C | A | T | T | T | T | A | T | T | G | T | G | T | G | G | C | T | G | G | A | T | T | C | C | A | C | T | T | T | G | G | T | T | T | C | T | T | C | C |
| SSSGP_1A2 | A | T | - | - | - | - | A | A | A | A | T | T | T | T | C | A | A | C | A | C | T | C | A | T | T | T | T | A | T | T | G | T | G | T | G | G | C | T | G | G | A | T | T | C | C | A | C | T | T | T | G | G | T | T | T | C | T | T | C | C |
| SSSGP_1D1 | A | A | T | A | A | A | A | A | A | A | A | T | T | C | T | C | A | C | A | T | T | C | A | T | T | T | - | A | T | T | G | T | G | T | G | G | C | T | G | G | A | T | T | C | C | G | C | T | T | T | A | G | T | T | T | G | T | T | C | C |
| SSSGP_1B1 | A | A | T | A | A | A | C | A | A | A | A | T | T | C | T | C | A | C | A | T | T | C | A | T | T | T | - | A | T | T | G | T | G | T | G | G | C | T | G | G | A | T | T | C | C | A | C | T | T | T | G | A | T | T | T | C | T | T | C | C |
| SSSGP-1E1 | A | A | T | A | A | A | T | A | A | A | A | A | T | C | T | C | A | C | C | T | T | C | A | T | T | C | - | A | T | T | G | T | G | T | G | G | C | T | G | G | A | T | T | C | G | T | A | T | T | T | G | G | T | T | T | C | C | T | C | C |

┤

| SSSGP_1C1 | T | T | G | G | C | A | T | T | T | C | T | C | C | A | A | T | T | T | A | A | T | T | A | A | A | A | T | C | T | A | T | C | A | A | T | T | T | A | A | T | T | A | T | T | T | C | A | T | A | **A** | **G** | G | C | T | G | - | - | - | - | - |
| --- | --- | --- | --- | --- | --- | --- | --- | --- | --- | --- | --- | --- | --- | --- | --- | --- | --- | --- | --- | --- | --- | --- | --- | --- | --- | --- | --- | --- | --- | --- | --- | --- | --- | --- | --- | --- | --- | --- | --- | --- | --- | --- | --- | --- | --- | --- | --- | --- | --- | --- | --- | --- | --- | --- | --- | --- | --- | --- | --- | --- |
| SSSGP_1C2 | C | T | G | G | C | A | T | T | T | C | T | C | C | A | A | T | T | T | A | A | T | A | A | A | A | A | T | C | T | A | T | T | A | A | T | T | T | A | A | T | T | A | T | T | T | C | A | T | A | **A** | **G** | G | C | T | G | C | T | - | - | - |
| SSSGP-1A1 | C | T | G | T | C | A | T | T | T | C | T | C | C | A | A | T | T | T | A | A | T | T | A | A | A | A | T | C | T | A | T | C | A | A | T | T | T | A | T | T | T | A | T | T | T | C | A | T | A | **A** | **G** | G | C | T | G | C | T | - | - | - |
| SSSGP_1A2 | C | T | G | T | C | A | T | T | T | C | T | C | C | A | A | T | T | T | A | A | T | T | A | A | A | A | T | C | T | A | T | C | A | A | T | T | T | A | T | T | T | A | T | T | T | C | A | T | A | **A** | **G** | G | C | T | G | C | T | - | - | - |
| SSSGP_1D1 | T | T | G | G | C | A | T | T | T | T | T | C | C | A | A | T | T | T | A | A | T | T | A | A | A | A | T | C | T | A | T | C | A | A | T | T | T | A | A | T | T | A | T | T | T | C | A | T | A | **A** | **G** | G | C | T | G | G | A | - | - | - |
| SSSGP_1B1 | C | T | G | T | C | A | T | T | T | C | T | C | C | A | A | T | T | T | A | A | T | T | A | A | A | A | T | C | T | A | T | C | A | A | T | T | T | A | T | T | T | A | T | T | T | C | A | T | A | **A** | **G** | G | C | T | G | C | T | - | - | - |
| SSSGP-1E1 | - | - | - | - | - | A | T | T | T | C | T | C | - | A | A | T | T | T | A | A | T | T | A | A | A | A | T | A | T | A | T | C | A | A | T | T | T | A | A | T | T | A | T | T | T | C | A | T | A | **A** | **G** | G | C | T | G | T | A | C | A | G |

| SSSGP_1C1 | - | - | - | - | - | - | T | A | C | A | C | A | - | - | - | - | - | - | - | - | G | C | A | G | - | - | C | C | A | T | T | C | C | A | C | A | G | C | T | G | C | A | C | A | - | - | - | - | - | C | G | C | T | G | G | T | A | A | A | A |
| --- | --- | --- | --- | --- | --- | --- | --- | --- | --- | --- | --- | --- | --- | --- | --- | --- | --- | --- | --- | --- | --- | --- | --- | --- | --- | --- | --- | --- | --- | --- | --- | --- | --- | --- | --- | --- | --- | --- | --- | --- | --- | --- | --- | --- | --- | --- | --- | --- | --- | --- | --- | --- | --- | --- | --- | --- | --- | --- | --- | --- |
| SSSGP_1C2 | - | G | - | - | - | - | T | A | C | A | C | A | - | - | - | - | - | - | - | - | G | C | A | G | - | - | C | C | A | T | T | C | C | A | C | A | G | C | T | G | C | A | C | A | - | - | - | - | - | C | G | C | A | G | G | A | A | A | T | A |
| SSSGP-1A1 | - | G | T | A | A | C | T | A | A | A | C | A | T | C | C | A | G | C | A | G | G | A | A | A | - | - | A | A | A | G | T | C | C | C | C | A | G | C | T | A | A | A | C | C | - | - | - | - | - | A | G | C | A | A | G | T | C | C | A | A |
| SSSGP_1A2 | - | G | T | A | A | C | T | A | A | A | C | A | T | C | C | A | G | C | A | G | G | A | A | A | - | - | A | A | A | G | T | C | C | C | C | A | G | C | T | A | A | A | C | C | - | - | - | - | - | A | G | C | A | A | G | T | C | C | A | A |
| SSSGP_1D1 | - | G | A | A | A | C | A | G | A | T | C | A | T | T | C | A | G | C | A | G | A | C | A | A | - | - | A | C | A | T | G | G | T | C | C | G | G | G | A | A | A | T | - | - | - | - | - | - | - | - | G | C | A | A | A | C | C | A | T | G |
| SSSGP_1B1 | - | A | A | A | C | C | T | A | A | A | A | A | - | - | - | - | - | - | A | G | G | C | A | A | - | - | A | A | A | G | C | C | C | G | C | A | G | C | T | G | C | A | C | C | - | - | - | - | - | G | G | C | A | G | G | T | C | C | A | A |
| SSSGP-1E1 | G | A | A | C | C | A | C | A | A | G | C | A | T | C | G | C | T | T | C | C | C | C | A | A | C | C | A | C | A | T | T | C | T | G | C | A | C | C | A | G | C | A | T | C | G | C | T | T | C | C | C | C | A | A | C | C | A | C | A | T |
|  |  |  |  |  |  |  |  |  |  |  |  |  |  |  |  |  |  |  |  |  |  |  |  |  |  |  |  |  |  |  |  |  |  |  |  |  |  |  |  |  |  |  |  |  |  |  |  |  |  |  |  |  |  |  |  |  |  |  |  |  |
| SSSGP_1C1 | C | C | C | A | T | G | A | G | C | C | - | - | - | - | - | - | - | A | G | C | A | A | A | A | A | C | G | C | A | A | A | C | A | G | C | C | C | A | A | C | C | A | G | G | C | G | T | A | G | G | T | G | A | T | C | A | T | C | T | T |
| SSSGP_1C2 | T | C | C | A | T | G | - | - | - | - | - | - | - | - | - | - | - | - | - | C | A | G | C | A | A | C | G | C | A | T | A | C | A | G | C | C | C | A | A | C | T | A | A | G | C | C | T | A | G | C | T | G | A | C | G | A | T | C | T | T |
| SSSGP-1A1 | C | C | C | A | T | G | - | - | - | - | - | - | - | - | - | - | - | - | - | C | A | G | C | A | G | C | G | C | C | A | A | C | A | G | C | C | C | A | A | T | C | A | A | A | C | C | C | A | G | A | T | G | A | C | C | A | A | T | T | T |
| SSSGP_1A2 | C | C | C | A | T | G | - | - | - | - | - | - | - | - | - | - | - | - | - | C | A | G | C | A | G | C | G | C | C | A | A | C | A | G | C | C | C | A | A | T | C | A | A | A | C | C | C | A | G | A | T | G | A | C | C | A | A | T | T | T |
| SSSGP_1D1 | C | T | G | C | A | A | - | - | - | - | - | - | - | - | - | - | - | - | - | A | G | A | A | A | C | C | G | C | A | C | A | C | A | G | C | C | C | A | A | C | C | A | A | G | C | A | C | A | T | A | T | G | A | C | T | T | T | C | T | T |
| SSSGP_1B1 | C | C | T | C | T | G | - | - | - | - | - | - | - | - | - | - | - | - | - | A | T | - | - | - | - | - | - | C | C | A | G | C | A | G | T | C | G | A | G | C | C | A | A | G | C | T | C | A | G | G | T | G | A | C | C | T | T | T | C | T |
| SSSGP-1E1 | T | C | T | G | C | A | C | C | A | C | T | T | C | A | C | C | A | A | C | C | A | G | C | A | C | C | G | C | A | A | A | C | A | G | T | C | C | A | T | T | C | T | A | G | C | T | C | C | G | C | - | - | A | T | C | G | T | T | G | A |
|  |  |  |  |  |  |  |  |  |  |  |  |  |  |  |  |  |  |  |  |  |  |  |  |  |  |  |  |  |  |  |  |  |  |  |  |  |  |  |  |  |  |  |  |  |  |  |  |  |  |  |  |  |  |  |  |  |  |  |  |  |
| SSSGP_1C1 | G | A | C | G | - | - | - | A | T | G | G | T | G | A | G | C | A | C | T | A | T | T | T | C | G | A | C | G | C | A | T | C | G | G | A | C | A | A | A | G | A | T | T | T | C | - | - | - | - | - | - | - | G | A | A | T | C | A | T | A |
| SSSGP_1C2 | G | A | C | G | - | - | - | A | A | G | A | T | G | A | G | C | A | C | T | G | G | T | T | C | G | A | C | G | C | A | T | C | G | G | A | C | A | A | A | G | A | T | T | T | C | - | - | - | - | - | - | - | G | A | A | T | C | A | T | A |
| SSSGP-1A1 | G | A | C | G | A | T | C | T | T | T | C | C | A | T | G | C | T | A | G | A | T | T | G | G | G | A | G | G | C | T | A | T | T | C | T | - | - | - | A | G | C | A | G | A | C | - | - | - | - | - | - | - | G | A | T | T | C | A | G | A |
| SSSGP_1A2 | G | A | C | G | A | T | C | T | T | T | C | C | A | T | G | C | T | A | G | A | T | T | G | G | G | A | G | G | C | T | A | T | T | C | T | - | - | - | A | G | C | A | G | A | C | - | - | - | - | - | - | - | G | A | T | T | C | A | G | A |
| SSSGP_1D1 | G | A | C | A | - | - | - | T | A | G | G | C | G | A | G | G | A | C | T | T | T | G | C | A | G | A | C | G | C | A | T | G | G | G | C | - | - | - | A | G | T | A | C | T | T | - | - | - | - | - | - | - | C | G | C | G | G | A | A | A |
| SSSGP_1B1 | G | A | T | G | A | C | C | A | A | A | C | C | A | T | A | A | A | - | - | - | T | T | G | G | G | A | T | G | C | A | C | T | T | T | T | - | - | - | A | G | C | T | G | C | C | - | - | - | - | - | - | - | G | A | C | T | C | A | A | A |
| SSSGP-1E1 | G | G | T | G | G | G | A | T | C | A | G | C | T | T | G | C | G | C | A | A | C | C | G | G | C | A | G | G | T | G | G | C | T | A | T | A | G | A | G | G | A | A | T | G | C | C | A | A | C | G | A | G | G | A | C | T | G | A | A | C |
|  |  |  |  |  |  |  |  |  |  |  |  |  |  |  |  |  |  |  |  |  |  |  |  |  |  |  |  |  |  |  |  |  |  |  |  |  |  |  |  |  |  |  |  |  |  |  |  |  |  |  |  |  |  |  |  |  |  |  |  |  |
| SSSGP_1C1 | T | T | T | C | G | A | A | G | A | T | G | A | T | A | - | - | - | - | A | T | G | A | G | C | C | T | A | A | T | G | A | A | - | - | - | G | C | A | G | C | C | G | T | A | - | - | - | - | - | - | - | - | C | C | G | G | C | - | - | - |
| SSSGP_1C2 | T | T | T | C | G | A | A | G | A | T | G | A | T | A | - | - | - | - | A | T | G | G | C | C | C | T | A | A | T | G | A | A | - | - | - | G | C | A | G | C | C | G | T | A | - | - | - | - | - | - | - | - | C | C | G | G | C | - | - | - |
| SSSGP-1A1 | T | T | T | C | G | G | A | A | A | T | T | C | T | A | - | - | - | - | A | T | G | G | A | C | C | T | G | C | C | A | C | A | C | C | A | G | C | A | G | C | T | G | C | A | - | - | - | - | - | - | - | - | C | C | A | G | C | - | - | - |
| SSSGP_1A2 | T | T | T | C | G | G | A | A | A | T | T | C | T | A | - | - | - | - | A | T | G | G | A | C | C | T | G | C | C | A | C | A | C | C | A | G | C | A | G | C | T | G | C | A | - | - | - | - | - | - | - | - | C | C | A | G | C | - | - | - |
| SSSGP_1D1 | T | T | T | A | G | G | A | G | G | G | C | A | G | A | - | - | - | - | A | T | G | G | A | G | C | A | G | G | C | A | A | A | C | C | G | G | C | A | G | T | T | G | C | A | - | - | - | - | - | - | - | - | T | C | A | G | C | - | - | - |
| SSSGP_1B1 | T | G | C | G | G | G | A | G | A | T | G | C | T | A | - | - | - | - | A | T | G | G | G | C | T | T | G | A | T | G | A | A | G | C | G | G | C | A | A | C | G | A | A | A | - | - | - | - | - | - | - | - | C | C | G | A | C | A | G | C |
| SSSGP-1E1 | C | A | C | C | G | A | A | A | A | C | A | A | G | A | G | G | G | G | A | C | G | G | A | T | C | A | T | C | A | A | C | A | A | C | A | A | C | A | A | T | C | G | C | A | A | A | A | A | A | A | T | G | T | C | G | T | C | A | C | G |
|  |  |  |  |  |  |  |  |  |  |  |  |  |  |  |  |  |  |  |  |  |  |  |  |  |  |  |  |  |  |  |  |  |  |  |  |  |  |  |  |  |  |  |  |  |  |  |  |  |  |  |  |  |  |  |  |  |  |  |  |  |
| SSSGP_1C1 | - | - | - | - | - | - | - | - | - | - | - | - | - | - | - | - | - | - | - | - | - | - | A | G | - | - | - | - | - | - | - | - | G | A | A | A | A | C | C | A | A | C | A | A | A | A | C | C | - | - | - | - | - | - | - | - | - | - | - | - |
| SSSGP_1C2 | - | - | - | - | - | - | - | - | - | - | - | - | - | - | - | - | - | - | - | - | - | - | A | G | - | - | - | - | - | - | - | - | G | A | A | A | A | C | C | A | A | C | A | A | A | A | C | C | - | - | - | - | - | - | - | - | - | - | - | - |
| SSSGP-1A1 | - | - | - | - | - | - | - | - | - | - | T | A | - | A | A | C | C | A | T | C | A | A | A | A | G | - | - | - | - | - | - | - | G | A | A | A | A | C | C | A | A | A | G | A | A | G | G | C | - | - | - | - | - | - | - | - | - | - | - | - |
| SSSGP_1A2 | - | - | - | - | - | - | - | - | - | - | T | A | - | A | A | C | C | A | T | C | A | A | A | A | G | - | - | - | - | - | - | - | G | A | A | A | A | C | C | A | A | A | G | A | A | G | G | C | - | - | - | - | - | - | - | - | - | - | - | - |
| SSSGP_1D1 | - | - | - | - | - | - | - | - | - | - | T | T | C | A | G | C | T | G | G | C | A | A | A | A | T | T | - | - | - | - | - | - | G | G | A | A | A | C | C | A | A | A | - | - | A | G | A | C | - | - | - | - | - | - | - | - | - | - | - | - |
| SSSGP_1B1 | - | - | - | - | - | - | - | - | C | G | C | A | A | A | A | G | G | A | C | C | A | A | A | G | A | C | G | - | G | C | C | G | G | A | A | A | A | C | C | A | G | C | T | A | A | A | C | C | - | - | - | - | - | - | - | - | - | - | - | - |
| SSSGP-1E1 | G | A | A | A | T | G | C | A | C | C | C | A | G | T | G | C | T | G | T | A | G | C | A | G | C | A | C | C | G | T | A | C | A | C | A | A | A | C | C | A | A | C | A | T | A | G | C | C | T | A | G | G | G | G | A | A | T | A | T | T |
|  |  |  |  |  |  |  |  |  |  |  |  |  |  |  |  |  |  |  |  |  |  |  |  |  |  |  |  |  |  |  |  |  |  |  |  |  |  |  |  |  |  |  |  |  |  |  |  |  |  |  |  |  |  |  |  |  |  |  |  |  |
| SSSGP_1C1 | - | - | A | T | T | G | A | A | G | G | C | C | A | - | - | - | - | A | A | A | C | A | T | C | G | C | C | A | A | A | A | G | - | - | - | - | - | - | - | G | A | A | A | A | G | G | C | C | A | - | - | - | - | - | - | - | - | - | C | C |
| SSSGP_1C2 | - | - | A | T | T | G | A | A | G | G | C | C | A | - | - | - | - | A | A | C | C | A | T | C | G | C | C | A | C | A | T | G | - | - | - | - | - | - | - | G | A | A | A | A | G | G | C | C | A | - | - | - | - | - | - | - | - | - | C | C |
| SSSGP-1A1 | - | - | C | G | A | T | T | C | A | C | C | A | A | - | - | - | - | A | A | T | C | A | C | C | A | A | A | A | C | C | A | G | - | - | - | C | C | C | C | A | A | A | A | A | A | A | G | C | A | - | - | - | - | - | - | G | C | C | T | C |
| SSSGP_1A2 | - | - | C | G | A | T | T | C | A | C | C | A | A | - | - | - | - | A | A | T | C | A | C | C | A | A | A | A | C | C | A | G | - | - | - | C | C | C | C | A | A | A | A | A | A | A | G | C | A | - | - | - | - | - | - | G | C | C | T | C |
| SSSGP_1D1 | - | - | G | G | C | C | T | C | A | C | C | A | C | - | - | - | - | C | A | C | A | T | G | G | G | C | A | C | G | C | A | T | - | - | - | C | A | C | C | A | A | C | A | A | A | G | C | C | A | - | - | - | - | - | - | G | C | C | C | C |
| SSSGP_1B1 | - | - | A | T | C | A | A | A | G | G | C | A | A | - | - | - | - | A | A | C | C | A | A | A | G | A | A | G | G | C | C | G | A | T | T | C | A | C | C | A | A | A | A | A | A | A | G | C | A | A | A | A | C | C | A | G | C | C | C | C |
| SSSGP-1E1 | C | T | G | T | C | G | A | A | G | A | T | G | A | T | G | A | G | G | A | C | T | G | G | T | A | C | G | A | C | G | C | A | T | T | G | G | A | C | G | A | A | A | A | T | T | T | G | G | A | A | T | C | A | C | T | T | C | C | C | C |

**├** 3’-UTR

| SSSGP_1C1 | A | A | A | - | - | - | - | - | - | - | - | - | - | - | - | - | - | - | - | A | A | A | A | A | **T** | **G** | **A** | T | C | A | T | T | T | C | A | T | T | C | A | A | T | - | T | G | A | A | A | G | A | A | C | A | T | T | T | G | G | - | - | - |
| --- | --- | --- | --- | --- | --- | --- | --- | --- | --- | --- | --- | --- | --- | --- | --- | --- | --- | --- | --- | --- | --- | --- | --- | --- | --- | --- | --- | --- | --- | --- | --- | --- | --- | --- | --- | --- | --- | --- | --- | --- | --- | --- | --- | --- | --- | --- | --- | --- | --- | --- | --- | --- | --- | --- | --- | --- | --- | --- | --- | --- |
| SSSGP_1C2 | A | A | A | - | - | - | - | - | - | - | - | - | - | - | - | - | - | - | - | A | A | A | A | G | **T** | **A** | **A** | T | C | A | T | T | T | C | A | T | T | C | A | A | T | - | T | G | A | A | A | G | A | A | C | A | T | T | T | G | G | - | - | - |
| SSSGP-1A1 | A | A | A | G | - | - | - | - | - | - | - | - | - | - | - | - | - | - | A | A | G | A | A | G | **T** | **G** | **A** | T | C | A | T | T | T | C | A | T | T | C | A | A | T | - | T | G | A | A | A | G | A | A | C | A | T | T | C | G | G | - | - | - |
| SSSGP_1A2 | A | A | A | G | - | - | - | - | - | - | - | - | - | - | - | - | - | - | A | A | G | A | A | G | **T** | **G** | **A** | T | C | A | T | T | T | C | A | T | T | C | A | A | T | - | T | G | A | A | A | G | A | A | C | A | T | T | C | G | G | - | - | - |
| SSSGP_1D1 | A | C | A | T | - | - | - | - | - | - | - | - | - | - | - | - | - | - | A | A | A | A | A | G | **T** | **G** | **A** | T | C | A | T | T | T | C | A | T | T | C | A | A | T | - | T | G | A | A | A | G | A | A | C | A | T | T | T | G | G | - | - | - |
| SSSGP_1B1 | A | A | A | G | - | - | - | - | - | - | - | - | - | - | - | - | - | - | A | A | G | A | A | G | **T** | **G** | **A** | T | C | A | T | T | T | C | A | T | T | C | A | A | T | - | T | G | A | A | A | G | A | A | C | A | T | T | C | G | G | - | - | - |
| SSSGP-1E1 | G | A | A | A | T | G | T | T | A | A | T | G | G | G | C | A | T | C | A | T | G | A | A | G | C | A | G | C | C | A | A | A | C | C | A | C | C | C | A | A | T | A | C | T | G | G | A | A | A | A | C | A | C | C | A | G | G | C | G | A |

|  |  |  |  |  |  |  |  |  |  |  |  |  |  |  |  |  |  |  |  |  |  |  |  |  |  |  |  |  |  |  |  |  |  |  |  |  |  |  |  |  |  |  |  |  |  |  |  |  |  |  |  |  |  |  |  |  |  |  |  |  |
| --- | --- | --- | --- | --- | --- | --- | --- | --- | --- | --- | --- | --- | --- | --- | --- | --- | --- | --- | --- | --- | --- | --- | --- | --- | --- | --- | --- | --- | --- | --- | --- | --- | --- | --- | --- | --- | --- | --- | --- | --- | --- | --- | --- | --- | --- | --- | --- | --- | --- | --- | --- | --- | --- | --- | --- | --- | --- | --- | --- | --- |
|  |  |  |  |  |  |  |  |  |  |  |  |  |  |  |  |  |  |  |  |  |  |  |  |  |  |  |  |  |  |  |  |  |  |  |  |  |  |  |  |  |  |  |  |  |  |  |  |  |  |  |  |  |  |  |  |  |  |  |  |  |
|  |  |  |  |  |  |  |  |  |  |  |  |  |  |  |  |  |  |  |  |  |  |  |  |  |  |  |  |  |  |  |  |  |  |  |  |  |  |  |  |  |  |  |  |  |  |  |  |  |  |  |  |  |  |  |  |  |  |  |  |  |
|  |  |  |  |  |  |  |  |  |  |  |  |  |  |  |  |  |  |  |  |  |  |  |  |  |  |  |  |  |  |  |  |  |  |  |  |  |  |  |  |  |  |  |  |  |  |  |  |  |  |  |  |  |  |  |  |  |  |  |  |  |
|  |  |  |  |  |  |  |  |  |  |  |  |  |  |  |  |  |  |  |  |  |  |  |  |  |  |  |  |  |  |  |  |  |  |  |  |  |  |  |  |  |  |  |  |  |  |  |  |  |  |  |  |  |  |  |  |  |  |  |  |  |
|  |  |  |  |  |  |  |  |  |  |  |  |  |  |  |  |  |  |  |  |  |  |  |  |  |  |  |  |  |  |  |  |  |  |  |  |  |  |  |  |  |  |  |  |  |  |  |  |  |  |  |  |  |  |  |  |  |  |  |  |  |
|  |  |  |  |  |  |  |  |  |  |  |  |  |  |  |  |  |  |  |  |  |  |  |  |  |  |  |  |  |  |  |  |  |  |  |  |  |  |  |  |  |  |  |  |  |  |  |  |  |  |  |  |  |  |  |  |  |  |  |  |  |
| SSSGP_1C1 | - | - | - | - | - | - | - | - | - | - | - | A | G | G | C | G | T | C | G | C | G | T | G | T | A | A | C | C | A | A | A | A | - | - | - | - | - | - | - | - | - | - | - | - | - | - | - | - | - | T | T | A | T | A | T | A | G | T | T | A |
| SSSGP_1C2 | - | - | - | - | - | - | - | - | - | - | - | A | G | G | C | G | T | C | G | C | G | T | G | T | A | A | C | C | A | A | A | A | - | - | - | - | - | - | - | - | - | - | - | - | - | - | - | - | - | T | T | A | A | A | T | A | G | T | T | A |
| SSSGP-1A1 | - | - | - | - | - | - | - | - | - | - | - | A | G | A | C | G | A | C | G | - | - | T | G | T | A | A | T | C | A | A | A | A | - | - | - | - | - | - | - | - | - | - | - | - | - | - | - | - | - | T | T | G | A | A | T | A | G | T | T | A |
| SSSGP_1A2 | - | - | - | - | - | - | - | - | - | - | - | A | G | A | C | G | T | C | G | - | - | T | G | T | A | A | T | C | A | A | A | A | - | - | - | - | - | - | - | - | - | - | - | - | - | - | - | - | - | T | T | A | A | A | T | A | G | T | T | A |
| SSSGP_1D1 | - | - | - | - | - | - | - | - | - | - | - | A | G | A | C | G | T | C | G | - | - | T | G | T | A | A | C | C | A | A | A | A | - | - | - | - | - | - | - | - | - | - | - | - | - | - | - | - | - | T | T | A | A | A | C | A | T | T | T | A |
| SSSGP_1B1 | - | - | - | - | - | - | - | - | - | - | - | A | G | A | C | G | T | C | G | - | - | T | G | T | A | A | T | C | A | A | A | A | - | - | - | - | - | - | - | - | - | - | - | - | - | - | - | - | - | T | T | A | A | A | T | A | G | T | T | A |
| SSSGP-1E1 | C | C | G | G | A | T | C | A | T | C | A | A | A | A | C | C | A | C | A | T | G | G | G | C | A | A | G | C | A | A | A | A | C | A | T | A | G | A | A | A | G | T | G | A | T | C | A | T | T | T | C | A | T | T | **T** | **A** | **A** | T | T | G |
|  |  |  |  |  |  |  |  |  |  |  |  |  |  |  |  |  |  |  |  |  |  |  |  |  |  |  |  |  |  |  |  |  |  |  |  |  |  |  |  |  |  |  |  |  |  |  |  |  |  |  |  |  |  |  |  |  |  |  |  |  |
| SSSGP_1C1 | T | A | C | A | C | - | - | - | T | C | T | C | A | T | A | T | T | T | T | C | A | A | T | T | T | G | T | C | T | A | T | T | G | A | A | A | T | C | A | A | T | T | C | T | T | G | A | A | A | T | T | A | A | A | T | T | C | T | T | G |
| SSSGP_1C2 | T | A | T | G | C | - | - | - | T | C | T | C | A | T | A | A | A | T | T | C | A | A | T | T | T | G | T | C | A | A | T | T | G | A | A | A | T | C | A | A | T | T | C | T | T | G | A | A | A | T | T | A | A | A | T | T | C | T | T | G |
| SSSGP-1A1 | T | A | C | G | C | - | - | - | T | C | T | C | A | T | A | T | T | T | T | C | A | A | T | T | T | G | T | C | C | A | T | T | G | A | A | A | T | C | A | A | T | T | C | T | T | G | A | G | A | T | T | A | A | A | T | T | C | T | T | G |
| SSSGP_1A2 | T | A | C | G | C | - | - | - | T | C | T | C | A | T | A | T | T | T | T | C | A | A | T | T | T | G | T | C | T | A | T | T | G | A | A | A | T | C | A | A | T | T | C | T | T | G | A | G | A | T | T | A | A | A | T | T | C | T | T | G |
| SSSGP_1D1 | T | A | T | G | C | - | - | - | T | C | T | C | A | T | A | T | T | T | T | C | A | A | T | T | T | G | T | C | A | A | T | T | T | A | A | A | T | C | A | A | T | T | C | T | T | G | C | A | A | T | T | A | A | A | T | T | T | T | T | G |
| SSSGP_1B1 | T | A | C | G | C | - | - | - | T | C | T | C | A | T | A | T | T | T | T | C | A | A | T | T | T | G | T | C | A | A | T | T | G | A | A | A | T | C | A | A | T | T | C | T | T | G | A | G | A | T | A | A | A | A | T | T | C | T | T | G |
| SSSGP-1E1 | A | A | A | G | T | A | C | A | T | C | T | C | A | T | A | T | T | T | T | C | A | A | A | T | T | G | T | C | C | A | T | T | G | A | A | A | T | C | G | A | T | T | C | T | T | G | C | A | A | T | T | A | A | A | T | T | C | T | T | G |
|  |  |  |  |  |  |  |  |  |  |  |  |  |  |  |  |  |  |  |  |  |  |  |  |  |  |  |  |  |  |  |  |  |  |  |  |  |  |  |  |  |  |  |  |  |  |  |  |  |  |  |  |  |  |  |  |  |  |  |  |  |
| SSSGP_1C1 | A | A | T | G | **T** | A | A | A | A | A | A | A | A | A | A | A | - | - | - | - | - | - | - | - | - | - | - | - | A | T | A | A | T | T | G | A | G | T | A | T | T | T | T | A | T | T | C | A | G | A | A | C | A | A | T | C | T | C | C | A |
| SSSGP_1C2 | A | A | T | G | T | A | A | A | A | A | A | A | A | A | A | A | T | A | A | A | A | T | A | A | A | A | T | A | A | T | A | A | T | T | G | A | G | T | A | T | T | T | **T** | A | T | T | C | A | G | A | A | C | G | A | T | C | T | C | C | A |
| SSSGP-1A1 | A | A | T | G | T | A | A | A | A | A | A | A | T | A | A | A | T | A | - | - | - | - | - | - | - | - | - | - | A | - | - | - | T | T | G | A | G | T | A | T | T | T | T | A | T | **T** | C | A | G | A | A | A | A | A | T | C | T | C | C | C |
| SSSGP_1A2 | A | A | T | G | T | A | A | A | A | A | A | A | T | A | A | A | T | A | - | - | - | - | - | - | - | - | - | - | A | - | - | - | T | T | G | A | G | T | A | T | T | T | T | A | T | **T** | C | A | G | A | A | A | A | A | T | C | T | C | C | C |
| SSSGP_1D1 | A | A | T | G | **T** | A | A | A | A | A | A | A | A | A | A | A | - | - | - | - | - | - | - | - | - | - | - | - | - | - | - | - | T | T | G | A | G | T | A | T | T | T | T | A | T | T | C | A | G | A | A | C | A | A | T | C | T | C | C | C |
| SSSGP_1B1 | A | A | T | G | **T** | A | A | A | A | A | A | A | A | A | A | A | T | A | - | - | - | - | - | - | - | - | - | - | A | T | A | A | T | T | G | A | G | T | A | T | T | T | T | A | T | T | C | A | G | A | A | C | A | A | T | C | T | C | C | C |
| SSSGP-1E1 | A | A | T | G | G | A | A | A | A | A | A | A | A | A | T | A | - | - | - | - | - | - | - | - | - | - | - | - | - | - | - | A | T | T | G | A | G | T | A | T | T | T | T | A | T | T | C | A | G | A | A | C | G | A | T | C | T | C | C | A |
|  |  |  |  |  |  |  |  |  |  |  |  |  |  |  |  |  |  |  |  |  |  |  |  |  |  |  |  |  |  |  |  |  |  |  |  |  |  |  |  |  |  |  |  |  |  |  |  |  |  |  |  |  |  |  |  |  |  |  |  |  |
| SSSGP_1C1 | C | - | C | A | T | T | C | T | T | A | T | T | C | A | T | C | C | A | T | A | G | G | - | T | A | A | - | - | - | - | - | T | C | T | C | T | T | T | A | G | - | - | T | T | A | T | A | A | T | T | T | T | - | - | - | - | G | T | A | C |
| SSSGP_1C2 | C | - | C | A | T | T | C | T | T | A | T | T | C | T | T | C | C | A | T | A | G | G | A | T | A | A | G | G | T | C | T | T | T | C | C | T | T | T | A | G | - | - | T | T | G | T | T | A | T | T | T | T | - | - | - | - | G | T | T | C |
| SSSGP-1A1 | C | - | C | A | T | T | T | T | C | A | T | T | C | T | T | C | C | A | T | A | G | G | - | T | A | A | - | - | - | - | - | T | C | T | C | T | T | T | A | G | - | - | T | T | A | T | T | A | T | T | T | T | - | - | - | - | G | T | T | C |
| SSSGP_1A2 | C | - | C | A | T | T | T | T | C | A | T | T | C | T | T | C | C | A | T | A | G | G | - | T | A | A | - | - | - | - | - | T | C | T | C | T | T | T | A | G | - | - | T | T | A | T | T | A | T | T | T | T | - | - | - | - | G | T | T | C |
| SSSGP_1D1 | C | T | C | A | T | T | C | T | T | A | T | C | C | A | T | C | C | A | T | A | G | G | - | T | A | A | - | - | - | - | - | T | C | T | A | T | T | T | A | G | - | - | T | T | A | T | T | A | T | T | T | T | - | - | - | - | G | T | T | C |
| SSSGP_1B1 | C | - | C | G | T | T | C | T | T | A | T | C | C | A | T | C | C | A | T | A | G | G | - | T | A | A | - | - | - | - | - | T | C | T | T | T | T | T | A | G | - | - | T | T | A | T | C | A | T | T | T | T | - | - | - | - | G | T | T | A |
| SSSGP-1E1 | C | - | C | A | T | T | G | T | T | A | T | T | C | A | T | C | A | A | T | A | G | G | - | T | A | A | - | - | - | - | - | T | C | T | C | T | T | T | A | G | G | G | T | A | A | T | C | T | T | T | T | T | A | G | G | T | G | T | T | T |
|  |  |  |  |  |  |  |  |  |  |  |  |  |  |  |  |  |  |  |  |  |  |  |  |  |  |  |  |  |  |  |  |  |  |  |  |  |  |  |  |  |  |  |  |  |  |  |  |  |  |  |  |  |  |  |  |  |  |  |  |  |
| SSSGP_1C1 | A | A | A | T | C | A | T | G | A | A | C | A | A | G | T | T | C | G | A | T | G | A | T | T | A | T | T | G | G | C | A | T | C | T | C | T | A | T | T | T | T | T | A | C | T | A | C | T | G | T | T | G | T | G | G | C | C | A | C | A |
| SSSGP_1C2 | A | A | A | T | C | A | T | A | A | A | C | A | A | G | T | T | C | G | A | A | A | A | T | T | - | T | G | A | T | A | A | T | T | T | C | T | A | T | T | C | A | A | T | G | T | A | C | G | A | G | T | G | - | A | A | C | G | T | C | A |
| SSSGP-1A1 | A | A | A | T | C | A | T | G | A | A | C | A | A | G | T | T | C | A | A | G | A | A | T | T | - | T | C | A | T | C | A | C | A | A | A | C | G | A | T | T | C | A | T | A | T | - | C | G | A | A | T | A | C | A | G | A | G | A | T | A |
| SSSGP_1A2 | A | A | A | T | C | A | T | G | A | A | C | A | A | G | T | T | C | A | A | G | A | A | T | T | - | T | C | A | T | C | A | C | A | A | A | C | G | A | T | T | C | A | T | A | T | - | C | G | A | A | T | A | C | A | G | A | G | A | T | A |
| SSSGP_1D1 | A | A | A | T | C | A | T | G | A | A | C | A | A | G | T | T | C | G | A | T | G | A | T | T | - | T | C | A | T | G | A | C | A | A | A | T | G | A | C | T | C | A | T | A | T | - | C | G | A | A | T | A | A | A | A | A | G | A | T | A |
| SSSGP_1B1 | A | A | A | T | C | A | T | G | A | A | C | A | A | G | T | T | C | G | A | G | A | A | T | C | - | T | C | A | T | C | A | C | A | C | A | C | G | A | T | T | G | A | C | C | A | - | C | G | A | A | T | C | A | A | A | T | G | A | - | - |
| SSSGP-1E1 | A | A | A | T | C | A | T | G | T | A | C | A | A | G | T | T | C | C | A | A | T | A | T | T | - | T | C | A | T | C | A | C | A | A | A | C | A | A | T | T | C | A | T | A | T | - | C | G | A | A | T | A | A | A | A | A | G | A | T | A |
|  |  |  |  |  |  |  |  |  |  |  |  |  |  |  |  |  |  |  |  |  |  |  |  |  |  |  |  |  |  |  |  |  |  |  |  |  |  |  |  |  |  |  |  |  |  |  |  |  |  |  |  |  |  |  |  |  |  |  |  |  |
| SSSGP_1C1 | C | A | T | A | C | A | A | T | G | A | T | - | - | - | - | - | - | - | - | - | - | A | C | A | A | C | G | A | T | A | A | A | C | G | A | T | T | G | T | T | G | T | A | T | C | A | C | A | T | G | T | G | T | A | A | A | C | T | A | A |
| SSSGP_1C2 | C | A | T | T | C | G | T | C | T | A | C | - | - | - | - | - | - | - | - | - | - | G | T | G | A | A | A | C | T | T | C | A | - | A | T | T | C | A | T | A | A | C | A | G | A | A | A | T | T | A | T | C | T | G | G | A | A | T | G | A |
| SSSGP-1A1 | C | A | C | T | T | C | T | G | T | T | C | - | - | - | - | - | - | - | - | A | A | T | G | T | T | C | G | A | T | T | T | A | G | G | T | T | C | A | C | A | T | T | C | G | T | C | T | A | T | G | T | T | T | G | G | C | T | T | C | A |
| SSSGP_1A2 | C | A | C | T | T | C | T | G | T | T | C | T | G | A | A | T | T | G | A | A | A | T | G | T | T | C | G | A | T | T | T | A | G | G | T | T | C | A | C | A | T | T | C | G | T | C | T | A | T | G | T | T | T | G | G | C | T | T | C | A |
| SSSGP_1D1 | C | A | C | T | T | C | T | G | T | A | T | - | - | - | - | - | - | - | G | A | A | T | T | T | A | C | G | T | T | T | A | A | C | G | T | T | C | A | C | A | T | T | C | G | T | C | T | A | C | G | T | T | T | G | G | C | T | T | C | A |
| SSSGP_1B1 | - | A | T | G | A | T | G | G | A | A | T | - | - | - | - | - | - | - | - | - | - | C | G | T | T | C | A | A | A | T | C | A | T | A | T | T | T | C | C | A | A | C | G | G | A | A | T | A | T | T | C | A | A | T | G | C | T | T | T | G |
| SSSGP-1E1 | C | A | C | T | T | C | T | A | A | T | G | A | A | T | T | T | C | T | C | T | A | C | G | T | T | T | G | G | C | T | T | - | C | A | A | T | A | A | T | A | A | C | T | A | A | C | T | T | A | G | T | C | T | A | A | C | T | T | A | A |
|  |  |  |  |  |  |  |  |  |  |  |  |  |  |  |  |  |  |  |  |  |  |  |  |  |  |  |  |  |  |  |  |  |  |  |  |  |  |  |  |  |  |  |  |  |  |  |  |  |  |  |  |  |  |  |  |  |  |  |  |  |
| SSSGP_1C1 | A | A | A | A | T | - | - | - | A | T | T | G | T | A | C | A | C | T | T | C | G | G | T | A | A | A | G | T | T | T | A | T | G | A | T | A | T | G | G | T | A | - | - | - | - | - | - | - | - | C | G | A | A | T | T | G | G | T | A | A |
| SSSGP_1C2 | A | C | - | - | T | - | - | - | G | T | T | G | A | A | A | C | G | T | T | C | A | T | T | G | T | T | A | A | A | C | G | T | A | T | C | A | C | C | G | A | A | - | - | - | - | - | - | - | - | G | A | A | T | T | T | T | G | T | T | T |
| SSSGP-1A1 | A | T | A | A | A | - | - | A | A | C | A | G | A | A | T | T | T | G | T | C | T | G | G | A | A | T | G | A | A | T | T | G | A | T | A | A | C | A | T | G | T | T | C | A | C | T | G | T | T | G | T | G | A | A | T | C | A | T | C | G |
| SSSGP_1A2 | A | T | A | A | A | - | - | A | A | C | A | G | A | A | T | T | T | G | T | C | T | G | G | G | A | T | G | A | A | T | T | G | A | T | G | A | - | - | - | - | - | - | - | - | - | - | - | - | - | G | C | G | A | A | T | C | A | T | C | G |
| SSSGP_1D1 | A | T | T | C | A | T | - | A | A | C | A | G | A | A | T | T | T | G | T | C | T | G | G | A | A | T | G | A | A | C | T | G | A | T | G | A | C | A | T | - | - | - | - | - | - | - | - | - | T | T | T | C | A | A | T | T | - | T | G | G |
| SSSGP_1B1 | A | T | T | G | A | T | C | G | A | T | A | A | T | C | T | T | T | T | T | C | A | T | T | G | G | T | G | G | A | C | G | A | A | A | A | A | T | G | T | A | A | - | - | - | - | - | - | - | C | A | A | G | A | A | C | T | - | T | G | A |
| SSSGP-1E1 | C | C | A | A | T | G | T | T | A | A | A | C | G | A | C | A | T | T | A | G | G | G | G | A | A | A | A | C | A | C | G | A | A | T | G | A | T | G | A | A | - | - | - | - | - | - | - | - | - | G | T | G | T | G | C | C | A | T | A | T |
|  |  |  |  |  |  |  |  |  |  |  |  |  |  |  |  |  |  |  |  |  |  |  |  |  |  |  |  |  |  |  |  |  |  |  |  |  |  |  |  |  |  |  |  |  |  |  |  |  |  |  |  |  |  |  |  |  |  |  |  |  |
| SSSGP_1C1 | A | C | C | C | T | G | T | A | A | A | A | T | A | A | T | T | T | A | A | A | T | A | A | T | T | T | T | G | G | G | C | T | A | A | T | A | T | T | T | G | G | T | A | C | A | A | C | A | T | A | T | T | C | T | C | T | T | A | T | A |
| SSSGP_1C2 | T | C | A | A | C | A | T | T | C | A | A | T | T | - | C | T | T | A | A | A | A | A | T | T | C | A | A | A | A | A | T | A | G | A | T | G | T | G | A | A | T | A | G | C | A | A | C | A | T | T | A | T | T | G | C | G | T | T | T | A |
| SSSGP-1A1 | A | C | A | A | A | A | T | G | T | G | T | T | - | - | - | C | T | C | A | A | C | A | T | T | C | A | A | A | T | T | T | C | A | A | T | T | T | G | G | T | C | G | A | - | - | A | - | A | T | - | - | G | C | G | A | T | T | T | C | - |
| SSSGP_1A2 | A | C | A | A | A | A | T | G | T | T | T | T | T | T | T | C | T | T | T | C | T | T | T | T | T | T | G | C | C | T | C | C | A | A | A | T | C | G | A | T | A | G | G | - | - | A | G | A | T | - | - | G | T | A | T | T | T | T | T | T |
| SSSGP_1D1 | T | C | G | T | A | A | T | G | C | G | A | T | T | - | - | C | T | G | A | A | A | A | T | T | C | A | T | A | G | A | T | A | A | A | A | G | T | G | A | A | T | G | G | C | A | A | A | A | T | - | - | T | C | A | T | T | T | T | C | A |
| SSSGP_1B1 | T | C | A | A | A | T | T | A | G | T | G | C | T | - | - | A | T | T | G | A | C | A | C | G | A | A | A | C | G | T | T | G | G | A | A | T | C | T | T | C | A | G | G | A | A | A | G | T | T | A | - | T | C | G | C | T | T | T | T | G |
| SSSGP-1E1 | A | T | T | T | C | C | T | A | T | G | A | T | - | - | - | - | T | C | A | A | A | A | T | - | - | - | - | A | A | G | T | T | A | G | T | T | T | T | T | T | T | C | G | C | A | A | T | G | T | T | T | T | G | C | C | C | C | T | T | - |
|  |  |  |  |  |  |  |  |  |  |  |  |  |  |  |  |  |  |  |  |  |  |  |  |  |  |  |  |  |  |  |  |  |  |  |  |  |  |  |  |  |  |  |  |  |  |  |  |  |  |  |  |  |  |  |  |  |  |  |  |  |
| SSSGP_1C1 | - | - | - | - | - | - |  |  |  |  |  |  |  |  |  |  |  |  |  |  |  |  |  |  |  |  |  |  |  |  |  |  |  |  |  |  |  |  |  |  |  |  |  |  |  |  |  |  |  |  |  |  |  |  |  |  |  |  |  |  |
| SSSGP_1C2 | A | T | T | A | A | T |  |  |  |  |  |  |  |  |  |  |  |  |  |  |  |  |  |  |  |  |  |  |  |  |  |  |  |  |  |  |  |  |  |  |  |  |  |  |  |  |  |  |  |  |  |  |  |  |  |  |  |  |  |  |
| SSSGP-1A1 | - | - | - | - | - | - |  |  |  |  |  |  |  |  |  |  |  |  |  |  |  |  |  |  |  |  |  |  |  |  |  |  |  |  |  |  |  |  |  |  |  |  |  |  |  |  |  |  |  |  |  |  |  |  |  |  |  |  |  |  |
| SSSGP_1A2 | - | - | - | - | - | - |  |  |  |  |  |  |  |  |  |  |  |  |  |  |  |  |  |  |  |  |  |  |  |  |  |  |  |  |  |  |  |  |  |  |  |  |  |  |  |  |  |  |  |  |  |  |  |  |  |  |  |  |  |  |
| SSSGP_1D1 | T | T | G | - | - | - |  |  |  |  |  |  |  |  |  |  |  |  |  |  |  |  |  |  |  |  |  |  |  |  |  |  |  |  |  |  |  |  |  |  |  |  |  |  |  |  |  |  |  |  |  |  |  |  |  |  |  |  |  |  |
| SSSGP_1B1 | A | T | C | C | G | - |  |  |  |  |  |  |  |  |  |  |  |  |  |  |  |  |  |  |  |  |  |  |  |  |  |  |  |  |  |  |  |  |  |  |  |  |  |  |  |  |  |  |  |  |  |  |  |  |  |  |  |  |  |  |
| SSSGP-1E1 | - | - | - | - | - | - |  |  |  |  |  |  |  |  |  |  |  |  |  |  |  |  |  |  |  |  |  |  |  |  |  |  |  |  |  |  |  |  |  |  |  |  |  |  |  |  |  |  |  |  |  |  |  |  |  |  |  |  |  |  |
|  |  |  |  |  |  |  |  |  |  |  |  |  |  |  |  |  |  |  |  |  |  |  |  |  |  |  |  |  |  |  |  |  |  |  |  |  |  |  |  |  |  |  |  |  |  |  |  |  |  |  |  |  |  |  |  |  |  |  |  |  |

**Protein alignment**

Intron/exon boundary

▼

| SSGP_1C1 | ***M*** | ***S*** | ***K*** | ***F*** | ***F*** | ***L*** | ***A*** | ***F*** | ***A*** | ***I*** | ***I*** | ***A*** | ***V*** | ***C*** | ***L*** | ***V*** | ***A*** | ***A*** | Q | - | - | - | - | - | - | - | - | - | - | - | - | - | - | - | - | - | - | - | - | - | - | - | - | - | - | - | - | - | - | - | - | - | A | V | H | S | S | - | - | - |
| --- | --- | --- | --- | --- | --- | --- | --- | --- | --- | --- | --- | --- | --- | --- | --- | --- | --- | --- | --- | --- | --- | --- | --- | --- | --- | --- | --- | --- | --- | --- | --- | --- | --- | --- | --- | --- | --- | --- | --- | --- | --- | --- | --- | --- | --- | --- | --- | --- | --- | --- | --- | --- | --- | --- | --- | --- | --- | --- | --- | --- |
| SSGP_1C2 | ***M*** | ***S*** | ***K*** | ***F*** | ***L*** | ***L*** | ***A*** | ***F*** | ***A*** | ***I*** | ***I*** | ***A*** | ***V*** | ***C*** | ***L*** | ***V*** | ***A*** | ***A*** | Q | A | - | - | - | - | - | - | - | - | - | - | - | - | - | - | - | - | - | - | - | - | - | - | - | - | - | - | - | - | - | - | - | - | A | V | H | S | S | - | - | - |
| SSGP_1E1 | ***M*** | ***S*** | ***K*** | ***F*** | ***F*** | ***L*** | ***A*** | ***F*** | ***A*** | ***V*** | ***I*** | ***A*** | ***V*** | ***C*** | ***L*** | ***V*** | ***A*** | ***A*** | Q | A | V | Q | E | P | Q | A | S | L | P | Q | P | H | S | A | P | A | S | L | P | Q | P | H | S | A | P | L | H | Q | P | A | P | Q | T | V | H | S | S | S | A | S |
| SSGP_1A1 | ***M*** | ***S*** | ***K*** | ***F*** | ***L*** | ***L*** | ***A*** | ***F*** | ***A*** | ***V*** | ***I*** | ***A*** | ***V*** | ***C*** | ***L*** | ***V*** | ***A*** | ***A*** | Q | A | A | V | - | - | - | - | - | - | - | - | - | - | - | - | - | - | - | - | - | - | - | - | - | - | - | - | - | - | - | - | - | - | T | K | H | P | A | - | - | - |
| SSGP_1A2 | ***M*** | ***S*** | ***K*** | ***F*** | ***L*** | ***L*** | ***A*** | ***F*** | ***A*** | ***V*** | ***I*** | ***A*** | ***V*** | ***C*** | ***L*** | ***V*** | ***A*** | ***A*** | Q | A | A | V | - | - | - | - | - | - | - | - | - | - | - | - | - | - | - | - | - | - | - | - | - | - | - | - | - | - | - | - | - | - | T | K | H | P | A | - | - | - |
| SSGP_1B1 | ***M*** | ***S*** | ***K*** | ***F*** | ***L*** | ***L*** | ***A*** | ***F*** | ***A*** | ***V*** | ***I*** | ***A*** | ***V*** | ***C*** | ***L*** | ***V*** | ***A*** | ***A*** | Q | A | A | K | - | - | - | - | - | - | - | - | - | - | - | - | - | - | - | - | - | - | - | - | - | - | - | - | - | - | - | - | - | - | P | K | K | - | - | - | - | - |
| SSGP_1D1 | ***M*** | ***S*** | ***K*** | ***F*** | ***L*** | ***L*** | ***A*** | ***F*** | ***A*** | ***V*** | ***I*** | ***A*** | ***V*** | ***C*** | ***L*** | ***V*** | ***A*** | ***A*** | Q | A | G | E | - | - | - | - | - | - | - | - | - | - | - | - | - | - | - | - | - | - | - | - | - | - | - | - | - | - | - | - | - | - | T | D | H | S | A | - | - | - |
|  |  |  |  |  |  |  |  |  |  |  |  |  |  |  |  |  |  |  |  |  |  |  |  |  |  |  |  |  |  |  |  |  |  |  |  |  |  |  |  |  |  |  |  |  |  |  |  |  |  |  |  |  |  |  |  |  |  |  |  |  |
| SSGP_1C1 | - | - | - | - | - | - | - | - | - | - | - | - | - | - | - | - | - | - | - | - | - | - | - | - | - | - | - | - | - | H | S | T | A | A | H | A | G | K | T | H | E | - | - | - | - | - | - | P | A | K | T | Q | T | A | Q | P | G | V | G | D |
| SSGP_1C2 | - | - | - | - | - | - | - | - | - | - | - | - | - | - | - | - | - | - | - | - | - | - | - | - | - | - | - | - | - | H | S | T | A | A | H | A | G | N | I | H | - | - | - | - | - | - | - | - | A | A | T | H | T | A | Q | L | S | L | A | D |
| SSGP_1E1 | L | R | W | D | Q | L | A | Q | P | A | G | G | Y | R | G | M | P | T | R | T | E | P | P | K | T | R | G | D | G | S | S | T | T | T | I | A | K | K | C | R | H | G | N | A | P | S | A | V | A | A | P | Y | T | N | Q | H | S | L | G | E |
| SSGP_1A1 | - | - | - | - | - | - | - | - | - | - | - | - | - | - | - | - | - | - | - | - | - | - | - | - | - | - | - | G | K | K | S | P | A | K | P | A | S | P | T | H | - | - | - | - | - | - | - | - | A | A | A | P | T | A | Q | S | N | P | D | D |
| SSGP_1A2 | - | - | - | - | - | - | - | - | - | - | - | - | - | - | - | - | - | - | - | - | - | - | - | - | - | - | - | G | K | K | S | P | A | K | P | A | S | P | T | H | - | - | - | - | - | - | - | - | A | A | A | P | T | A | Q | S | N | P | D | D |
| SSGP_1B1 | - | - | - | - | - | - | - | - | - | - | - | - | - | - | - | - | - | - | - | - | - | - | - | - | - | - | - | G | K | K | P | A | A | A | P | A | G | P | T | - | - | - | - | - | - | - | - | - | - | S | D | P | A | V | E | P | S | S | G | D |
| SSGP_1D1 | - | - | - | - | - | - | - | - | - | - | - | - | - | - | - | - | - | - | - | - | - | - | - | - | - | - | - | D | K | H | G | P | G | N | - | A | N | H | A | A | - | - | - | - | - | - | - | - | K | K | P | H | T | A | Q | P | S | T | Y | D |
|  |  |  |  |  |  |  |  |  |  |  |  |  |  |  |  |  |  |  |  |  |  |  |  |  |  |  |  |  |  |  |  |  |  |  |  |  |  |  |  |  |  |  |  |  |  |  |  |  |  |  |  |  |  |  |  |  |  |  |  |  |
| SSGP_1C1 | H | - | L | D | D | G | E | H | Y | F | D | A | S | D | K | D | F | E | S | Y | F | E | D | D | N | E | - | - | - | - | - | P | N | E | A | A | V | - | - | - | - | - | - | - | - | P | A | G | K | P | T | - | K | P | L | K | A | K | T | S |
| SSGP_1C2 | D | - | L | D | E | D | E | H | W | F | D | A | S | D | K | D | F | E | S | Y | F | E | D | D | N | G | - | - | - | - | - | P | N | E | A | A | V | - | - | - | - | - | - | - | - | P | A | G | K | P | T | - | K | P | L | K | A | K | P | S |
| SSGP_1E1 | Y | S | V | E | D | D | E | D | W | Y | D | A | L | D | E | N | L | E | S | L | P | R | N | V | N | G | - | - | - | - | - | H | H | E | A | A | K | - | - | - | - | - | - | - | - | P | P | N | T | G | K | - | H | Q | A | T | G | S | S | K |
| SSGP_1A1 | Q | F | D | D | L | S | M | L | D | W | E | A | I | L | A | D | - | D | S | D | F | G | N | S | N | G | - | - | - | - | P | A | T | P | A | A | A | - | - | - | - | - | - | - | - | P | A | K | P | S | K | G | K | P | K | K | A | D | S | P |
| SSGP_1A2 | Q | F | D | D | L | S | M | L | D | W | E | A | I | L | A | D | - | D | S | D | F | G | N | S | N | G | - | - | - | - | P | A | T | P | A | A | A | - | - | - | - | - | - | - | - | P | A | K | P | S | K | G | K | P | K | K | A | D | S | P |
| SSGP_1B1 | L | S | D | D | Q | - | T | I | N | W | D | A | L | L | A | A | - | D | S | N | A | G | D | A | N | G | L | D | E | A | A | T | K | P | T | A | A | K | G | P | K | T | A | G | K | P | A | K | P | S | K | A | K | P | K | K | A | D | S | P |
| SSGP_1D1 | F | L | D | I | G | - | E | D | F | A | D | A | W | A | V | L | - | R | G | N | L | G | G | Q | N | G | - | - | - | - | A | G | K | P | A | V | A | - | - | - | - | - | - | - | - | S | A | S | A | G | K | I | G | N | Q | K | T | A | S | P |
|  |  |  |  |  |  |  |  |  |  |  |  |  |  |  |  |  |  |  |  |  |  |  |  |  |  |  |  |  |  |  |  |  |  |  |  |  |  |  |  |  |  |  |  |  |  |  |  |  |  |  |  |  |  |  |  |  |  |  |  |  |
| SSGP_1C1 | P | K | G | K | G | H | Q | K | K | - | - | - | - | - | - |  |  |  |  |  |  |  |  |  |  |  |  |  |  |  |  |  |  |  |  |  |  |  |  |  |  |  |  |  |  |  |  |  |  |  |  |  |  |  |  |  |  |  |  |  |
| SSGP_1C2 | P | H | G | K | G | H | Q | K | K | - | - | - | - | - | - |  |  |  |  |  |  |  |  |  |  |  |  |  |  |  |  |  |  |  |  |  |  |  |  |  |  |  |  |  |  |  |  |  |  |  |  |  |  |  |  |  |  |  |  |  |
| SSGP_1E1 | P | H | G | Q | A | K | H | R | K | - | - | - | - | - | - |  |  |  |  |  |  |  |  |  |  |  |  |  |  |  |  |  |  |  |  |  |  |  |  |  |  |  |  |  |  |  |  |  |  |  |  |  |  |  |  |  |  |  |  |  |
| SSGP_1A1 | K | S | P | K | P | A | P | K | K | A | A | S | K | K | K |  |  |  |  |  |  |  |  |  |  |  |  |  |  |  |  |  |  |  |  |  |  |  |  |  |  |  |  |  |  |  |  |  |  |  |  |  |  |  |  |  |  |  |  |  |
| SSGP_1A2 | K | S | P | K | P | A | P | K | K | A | A | S | K | K | K |  |  |  |  |  |  |  |  |  |  |  |  |  |  |  |  |  |  |  |  |  |  |  |  |  |  |  |  |  |  |  |  |  |  |  |  |  |  |  |  |  |  |  |  |  |
| SSGP_1B1 | K | K | A | K | P | A | P | K | K | K | - | - | - | - | - |  |  |  |  |  |  |  |  |  |  |  |  |  |  |  |  |  |  |  |  |  |  |  |  |  |  |  |  |  |  |  |  |  |  |  |  |  |  |  |  |  |  |  |  |  |
| SSGP_1D1 | P | H | G | H | A | S | P | T | K | P | A | P | H | K | K |  |  |  |  |  |  |  |  |  |  |  |  |  |  |  |  |  |  |  |  |  |  |  |  |  |  |  |  |  |  |  |  |  |  |  |  |  |  |  |  |  |  |  |  |  |
|  |  |  |  |  |  |  |  |  |  |  |  |  |  |  |  |  |  |  |  |  |  |  |  |  |  |  |  |  |  |  |  |  |  |  |  |  |  |  |  |  |  |  |  |  |  |  |  |  |  |  |  |  |  |  |  |  |  |  |  |  |

**B**

| Gene_G9E4 | - | - | - | - | - | - | - | - | - | - | - | - | - | C | G | A | T | T | T | T | G | A | T | T | G | T | T | T | C | A | A | T | G | T | T | C | T | C | A | A | T | T | G | A | C | G | C | A | A | A | T | A | T | T | G | A | T | A | T | T |
| --- | --- | --- | --- | --- | --- | --- | --- | --- | --- | --- | --- | --- | --- | --- | --- | --- | --- | --- | --- | --- | --- | --- | --- | --- | --- | --- | --- | --- | --- | --- | --- | --- | --- | --- | --- | --- | --- | --- | --- | --- | --- | --- | --- | --- | --- | --- | --- | --- | --- | --- | --- | --- | --- | --- | --- | --- | --- | --- | --- | --- |
| Gene_G10C9 | G | C | G | C | A | G | A | T | G | A | T | A | C | C | G | A | T | T | T | T | G | A | T | T | G | T | T | T | C | A | A | T | G | T | T | C | T | C | A | A | C | T | G | A | C | G | C | A | A | A | T | A | T | T | G | A | T | A | T | T |
|  |  |  |  |  |  |  |  |  |  |  |  |  |  |  |  |  |  |  |  |  |  |  |  |  |  |  |  |  |  |  |  |  |  |  |  |  |  |  |  |  |  |  |  |  |  |  |  |  |  |  |  |  |  |  |  |  |  |  |  |  |
| Gene_G9E4 | C | T | C | C | G | A | T | G | T | A | A | A | T | G | T | T | T | G | T | T | A | T | G | C | A | T | A | A | A | T | C | G | T | A | C | G | A | T | A | G | T | T | T | T | A | G | G | A | A | T | A | T | T | G | T | C | A | A | T | A |
| Gene_G10C9 | T | T | C | C | G | A | T | A | T | A | A | A | T | G | T | T | T | G | T | T | A | T | G | C | A | T | A | A | A | T | C | G | T | A | C | G | A | T | C | G | T | T | T | T | A | G | G | A | A | T | A | T | T | G | T | C | A | A | T | A |
|  |  |  |  |  |  |  |  |  |  |  |  |  |  |  |  |  |  |  |  |  |  |  |  |  |  |  |  |  |  |  |  |  |  |  |  |  |  |  |  |  |  |  |  |  |  |  |  |  |  |  |  |  |  |  |  |  |  |  |  |  |
| Gene_G9E4 | T | C | A | T | T | T | G | T | C | T | T | T | T | A | T | T | T | G | T | T | T | T | A | A | A | A | T | A | G | C | T | G | T | T | T | - | T | T | C | C | C | G | T | T | T | C | G | A | A | T | G | C | T | T | C | A | T | G | C | G |
| Gene_G10C9 | T | T | A | A | T | T | A | A | C | T | T | T | T | A | T | T | T | G | T | T | T | T | A | G | A | A | T | G | G | A | T | T | T | T | T | G | T | T | T | C | C | A | T | T | T | C | G | A | A | T | G | C | T | T | T | A | T | G | C | G |

**Putative TATA box ├5’-UTR**

| Gene_G9E4 | T | A | G | C | A | A | A | A | T | C | A | G | - | - | - | A | G | **T** | **A** | **T** | **A** | **A** | A | A | G | T | G | G | A | T | C | G | A | G | T | T | T | T | G | G | T | T | A | A | A | A | G | T | **A** | T | C | A | G | T | T | A | T | T | C | A |
| --- | --- | --- | --- | --- | --- | --- | --- | --- | --- | --- | --- | --- | --- | --- | --- | --- | --- | --- | --- | --- | --- | --- | --- | --- | --- | --- | --- | --- | --- | --- | --- | --- | --- | --- | --- | --- | --- | --- | --- | --- | --- | --- | --- | --- | --- | --- | --- | --- | --- | --- | --- | --- | --- | --- | --- | --- | --- | --- | --- | --- |
| Gene_G10C9 | T | A | G | C | A | A | A | A | T | C | A | G | C | A | G | A | G | **T** | **A** | **T** | **A** | **A** | A | A | G | T | G | G | A | C | C | G | A | G | T | T | T | T | G | G | T | T | A | A | A | G | G | T | **A** | T | C | A | G | T | T | A | T | T | C | A |

**├ SPCR**

| Gene_G9E4 | A | T | T | C | A | T | T | C | G | A | A | A | C | G | A | G | T | A | A | C | A | G | T | T | T | A | A | T | A | T | T | T | G | A | A | A | A | A | A | A | T | A | A | A | A | **A** | **T** | **G** | A | A | A | G | T | C | A | T | C | A | T | T |
| --- | --- | --- | --- | --- | --- | --- | --- | --- | --- | --- | --- | --- | --- | --- | --- | --- | --- | --- | --- | --- | --- | --- | --- | --- | --- | --- | --- | --- | --- | --- | --- | --- | --- | --- | --- | --- | --- | --- | --- | --- | --- | --- | --- | --- | --- | --- | --- | --- | --- | --- | --- | --- | --- | --- | --- | --- | --- | --- | --- | --- |
| Gene_G10C9 | A | T | T | C | A | T | T | C | G | A | A | A | C | G | A | G | T | A | A | C | A | G | T | T | T | A | A | T | A | T | T | T | G | A | A | A | A | A | A | A | T | A | A | A | A | **A** | **T** | **G** | A | A | A | G | T | C | A | T | C | A | T | T |

**├ MPCR**

| Gene_G9E4 | T | T | A | G | C | T | T | T | G | T | T | C | G | C | A | A | T | C | A | T | T | - | - | - | - | - | - | G | C | C | T | G | T | G | T | A | T | C | A | G | G | T | C | A | A | G | G | A | C | C | A | C | C | T | G | G | T | C | C | T |
| --- | --- | --- | --- | --- | --- | --- | --- | --- | --- | --- | --- | --- | --- | --- | --- | --- | --- | --- | --- | --- | --- | --- | --- | --- | --- | --- | --- | --- | --- | --- | --- | --- | --- | --- | --- | --- | --- | --- | --- | --- | --- | --- | --- | --- | --- | --- | --- | --- | --- | --- | --- | --- | --- | --- | --- | --- | --- | --- | --- | --- |
| Gene_G10C9 | T | T | A | G | C | T | T | T | G | T | T | C | G | C | A | A | T | C | A | T | T | G | C | T | G | T | G | G | C | C | T | G | T | G | T | G | T | C | A | G | G | T | C | - | - | - | - | - | - | - | - | - | - | - | - | - | - | - | - | - |
|  |  |  |  |  |  |  |  |  |  |  |  |  |  |  |  |  |  |  |  |  |  |  |  |  |  |  |  |  |  |  |  |  |  |  |  |  |  |  |  |  |  |  |  |  |  |  |  |  |  |  |  |  |  |  |  |  |  |  |  |  |
| Gene_G9E4 | G | T | T | C | C | A | C | A | A | C | C | T | A | G | T | T | T | G | G | T | T | T | T | G | C | A | A | C | C | A | C | C | A | C | C | A | C | A | A | A | C | C | G | G | T | C | C | T | G | G | C | C | C | T | G | T | T | G | C | C |
| Gene_G10C9 | - | - | - | - | - | - | - | - | - | - | - | - | - | - | - | - | - | - | - | - | - | T | A | C | C | A | A | C | T | T | T | C | A | C | T | C | T | - | - | - | - | - | - | - | T | C | C | T | G | T | T | C | C | A | G | G | T | G | T | - |
|  |  |  |  |  |  |  |  |  |  |  |  |  |  |  |  |  |  |  |  |  |  |  |  |  |  |  |  |  |  |  |  |  |  |  |  |  |  |  |  |  |  |  |  |  |  |  |  |  |  |  |  |  |  |  |  |  |  |  |  |  |
| Gene_G9E4 | C | C | C | C | C | A | C | C | A | C | C | A | C | C | A | C | C | A | T | T | T | G | G | C | C | C | T | G | T | T | C | C | A | G | C | A | A | C | A | T | G | T | G | T | T | C | C | T | G | C | T | C | C | G | T | T | A | C | C | A |
| Gene_G10C9 | - | - | - | - | - | A | G | G | A | G | C | A | T | C | C | C | T | T | A | C | T | G | G | T | C | C | A | A | G | T | A | T | A | G | A | A | G | C | A | T | C | T | C | T | T | C | C | T | T | T | T | G | C | A | - | - | - | - | - | - |
|  |  |  |  |  |  |  |  |  |  |  |  |  |  |  |  |  |  |  |  |  |  |  |  |  |  |  |  |  |  |  |  |  |  |  |  |  |  |  |  |  |  |  |  |  |  |  |  |  |  |  |  |  |  |  |  |  |  |  |  |  |
| Gene_G9E4 | C | C | A | T | G | T | A | A | T | C | T | T | G | A | A | T | C | A | T | C | T | G | G | C | T | C | T | G | G | T | C | A | A | C | C | A | G | C | A | T | C | T | A | A | T | T | T | A | G | T | T | C | C | A | T | C | A | T | C | G |
| Gene_G10C9 | - | - | - | - | - | - | A | G | T | C | T | A | G | G | A | G | C | A | T | C | T | C | T | T | C | C | A | A | G | T | T | T | A | G | G | A | G | T | A | - | - | - | A | A | C | C | T | T | G | C | T | - | - | - | - | - | - | - | - | - |
|  |  |  |  |  |  |  |  |  |  |  |  |  |  |  |  |  |  |  |  |  |  |  |  |  |  |  |  |  |  |  |  |  |  |  |  |  |  |  |  |  |  |  |  |  |  |  |  |  |  |  |  |  |  |  |  |  |  |  |  |  |
| Gene_G9E4 | G | T | T | C | C | A | T | G | C | C | T | A | C | A | A | A | C | A | C | C | A | A | T | T | G | A | T | G | C | A | A | G | T | C | A | A | G | T | A | C | C | A | G | C | T | G | T | T | C | A | A | C | A | A | C | C | A | C | C | T |
| Gene_G10C9 | - | - | - | - | - | - | - | - | - | - | - | - | - | - | - | - | - | - | - | - | - | - | - | - | G | G | T | C | C | A | A | G | T | C | A | A | G | C | A | G | C | A | A | C | T | C | T | T | C | - | - | - | - | - | - | - | - | - | C | T |
|  |  |  |  |  |  |  |  |  |  |  |  |  |  |  |  |  |  |  |  |  |  |  |  |  |  |  |  |  |  |  |  |  |  |  |  |  |  |  |  |  |  |  |  |  |  |  |  |  |  |  |  |  |  |  |  |  |  |  |  |  |
| Gene_G9E4 | G | T | T | C | C | A | G | G | T | C | A | A | G | G | A | C | C | A | T | C | T | C | T | C | A | C | C | G | G | T | C | C | G | G | C | A | C | C | A | C | C | A | A | C | A | A | C | T | C | C | A | A | C | C | A | C | T | G | C | A |
| Gene_G10C9 | T | C | C | T | C | A | A | G | T | G | C | A | G | A | T | - | - | - | - | - | - | - | - | - | - | - | - | - | - | - | - | - | - | G | C | A | T | C | A | T | C | A | A | C | T | G | C | T | A | C | C | G | C | T | A | T | T | T | C | A |

**├ 3’-UTR**

| Gene_G9E4 | **T** | **A** | **A** | A | A | A | C | G | A | T | T | C | G | A | T | T | C | C | A | A | T | T | C | A | A | A | G | A | T | A | A | A | A | A | T | T | T | C | A | T | G | T | A | A | T | T | C | A | T | T | G | C | T | T | A | T | A | C | A | A |
| --- | --- | --- | --- | --- | --- | --- | --- | --- | --- | --- | --- | --- | --- | --- | --- | --- | --- | --- | --- | --- | --- | --- | --- | --- | --- | --- | --- | --- | --- | --- | --- | --- | --- | --- | --- | --- | --- | --- | --- | --- | --- | --- | --- | --- | --- | --- | --- | --- | --- | --- | --- | --- | --- | --- | --- | --- | --- | --- | --- | --- |
| Gene_G10C9 | **T** | **A** | **A** | A | A | A | C | G | A | T | T | C | G | A | T | T | T | C | A | A | T | T | C | A | A | A | G | A | T | A | A | A | A | A | T | T | T | C | A | T | G | T | A | A | T | T | C | A | T | T | G | - | - | - | - | - | - | - | A | A |
|  |  |  |  |  |  |  |  |  |  |  |  |  |  |  |  |  |  |  |  |  |  |  |  |  |  |  |  |  |  |  |  |  |  |  |  |  |  |  |  |  |  |  |  |  |  |  |  |  |  |  |  |  |  |  |  |  |  |  |  |  |
| Gene_G9E4 | C | T | A | A | A | C | T | T | T | C | A | A | T | A | A | A | A | T | A | A | A | T | T | T | G | A | A | A | G | A | C | A | A | A | A | A | T | G | T | C | T | - | - | G | C | A | T | G | A | A | A | A | A | A | T | A | T | T | G | A |
| Gene_G10C9 | C | T | A | A | A | C | T | T | T | C | A | A | T | A | A | A | A | T | A | A | A | T | T | T | A | A | A | A | G | A | C | G | A | A | A | A | T | G | T | C | T | T | T | G | C | A | T | G | A | A | A | A | A | A | T | G | T | T | G | A |
|  |  |  |  |  |  |  |  |  |  |  |  |  |  |  |  |  |  |  |  |  |  |  |  |  |  |  |  |  |  |  |  |  |  |  |  |  |  |  |  |  |  |  |  |  |  |  |  |  |  |  |  |  |  |  |  |  |  |  |  |  |
| Gene_G9E4 | G | T | T | T | T | T | G | G | T | T | T | T | T | G | T | G | T | C | A | T | G | T | T | A | A | C | A | G | A | A | T | A | G | A | A | T | C | C | T | A | C | C | T | T | T | T | G | A | C | A | T | A | A | G | A | T | A | - | - | T |
| Gene_G10C9 | G | T | T | T | T | C | G | A | C | T | T | T | T | G | T | G | T | T | A | T | T | T | G | A | A | G | A | G | A | A | A | A | G | A | A | T | C | C | T | A | T | T | T | T | T | T | G | G | C | A | T | A | A | A | A | A | A | A | A | T |
|  |  |  |  |  |  |  |  |  |  |  |  |  |  |  |  |  |  |  |  |  |  |  |  |  |  |  |  |  |  |  |  |  |  |  |  |  |  |  |  |  |  |  |  |  |  |  |  |  |  |  |  |  |  |  |  |  |  |  |  |  |
| Gene_G9E4 | A | A | T | A | A | T | T | C | A | G | C | C | T | A | T | T | T | A | C | T | G | G | T | A | C | T | T | C | G | A | A | T | T | G | C | A | T | A | C | C | T | T | T | A | T | G | G | G | C | A | T | A | T | T | G | - | - | - | - | - |
| Gene_G10C9 | G | A | T | A | A | T | T | C | A | G | C | C | T | A | T | T | T | A | C | T | G | G | T | G | C | T | T | T | G | A | A | T | C | G | C | A | T | A | T | T | T | T | T | A | T | G | G | G | C | A | T | T | T | T | T | C | C | T | A | A |
|  |  |  |  |  |  |  |  |  |  |  |  |  |  |  |  |  |  |  |  |  |  |  |  |  |  |  |  |  |  |  |  |  |  |  |  |  |  |  |  |  |  |  |  |  |  |  |  |  |  |  |  |  |  |  |  |  |  |  |  |  |
| Gene_G9E4 | - | - | - | - | - | - |  |  |  |  |  |  |  |  |  |  |  |  |  |  |  |  |  |  |  |  |  |  |  |  |  |  |  |  |  |  |  |  |  |  |  |  |  |  |  |  |  |  |  |  |  |  |  |  |  |  |  |  |  |  |
| Gene_G10C9 | T | C | G | G | T | C |  |  |  |  |  |  |  |  |  |  |  |  |  |  |  |  |  |  |  |  |  |  |  |  |  |  |  |  |  |  |  |  |  |  |  |  |  |  |  |  |  |  |  |  |  |  |  |  |  |  |  |  |  |  |
|  |  |  |  |  |  |  |  |  |  |  |  |  |  |  |  |  |  |  |  |  |  |  |  |  |  |  |  |  |  |  |  |  |  |  |  |  |  |  |  |  |  |  |  |  |  |  |  |  |  |  |  |  |  |  |  |  |  |  |  |  |

**Protein alignment:**

| G9E4 | ***M*** | ***K*** | ***V*** | ***I*** | ***I*** | ***L*** | ***A*** | ***L*** | ***F*** | ***A*** | ***I*** | ***I*** | ***A*** | ***C*** | ***V*** | ***S*** | ***G*** | Q | G | P | P | G | P | V | P | Q | P | S | L | V | L | Q | P | P | P | Q | T | G | P | G | P | V | A | P | P | P | P | P | P | F | G | P | V | P | A | T | C | V | P | A |
| --- | --- | --- | --- | --- | --- | --- | --- | --- | --- | --- | --- | --- | --- | --- | --- | --- | --- | --- | --- | --- | --- | --- | --- | --- | --- | --- | --- | --- | --- | --- | --- | --- | --- | --- | --- | --- | --- | --- | --- | --- | --- | --- | --- | --- | --- | --- | --- | --- | --- | --- | --- | --- | --- | --- | --- | --- | --- | --- | --- | --- |
| G10C9 | ***M*** | ***K*** | ***V*** | ***I*** | ***I*** | ***L*** | ***A*** | ***L*** | ***F*** | ***A*** | ***I*** | ***I*** | ***A*** | ***V*** | ***A*** | ***C*** | ***V*** | ***S*** | ***G*** | - | - | - | - | - | - | L | P | T | F | T | L | - | P | V | P | G | V | G | A | S | L | T | G | P | S | I | E | A | S | L | - | - | - | P | F | A | S | L | G | A |
|  |  |  |  |  |  |  |  |  |  |  |  |  |  |  |  |  |  |  |  |  |  |  |  |  |  |  |  |  |  |  |  |  |  |  |  |  |  |  |  |  |  |  |  |  |  |  |  |  |  |  |  |  |  |  |  |  |  |  |  |  |
| G9E4 | P | L | P | P | C | N | L | E | S | S | G | S | G | Q | P | A | S | N | L | V | P | S | S | V | P | C | L | Q | T | P | I | D | A | S | Q | V | P | A | V | Q | Q | P | P | V | P | G | Q | G | P | S | L | T | G | P | A | P | P | T | T | P |
| G10C9 | S | L | P | S | L | G | V | N | L | A | G | P | S | Q | A | A | T | - | - | L | P | S | - | - | - | - | - | - | S | S | A | D | A | S | S | T | A | T | A | I | S | - | - | - | - | - | - | - | - | - | - | - | - | - | - | - | - | - | - | - |
|  |  |  |  |  |  |  |  |  |  |  |  |  |  |  |  |  |  |  |  |  |  |  |  |  |  |  |  |  |  |  |  |  |  |  |  |  |  |  |  |  |  |  |  |  |  |  |  |  |  |  |  |  |  |  |  |  |  |  |  |  |
| G9E4 | T | T | A |  |  |  |  |  |  |  |  |  |  |  |  |  |  |  |  |  |  |  |  |  |  |  |  |  |  |  |  |  |  |  |  |  |  |  |  |  |  |  |  |  |  |  |  |  |  |  |  |  |  |  |  |  |  |  |  |  |
| G10C9 | - | - | - |  |  |  |  |  |  |  |  |  |  |  |  |  |  |  |  |  |  |  |  |  |  |  |  |  |  |  |  |  |  |  |  |  |  |  |  |  |  |  |  |  |  |  |  |  |  |  |  |  |  |  |  |  |  |  |  |  |
|  |  |  |  |  |  |  |  |  |  |  |  |  |  |  |  |  |  |  |  |  |  |  |  |  |  |  |  |  |  |  |  |  |  |  |  |  |  |  |  |  |  |  |  |  |  |  |  |  |  |  |  |  |  |  |  |  |  |  |  |  |

**C**

| Gene_L6C11 | C | C | G | T | T | T | T | G | A | T | T | T | C | G | T | T | T | T | G | A | T | C | T | G | A | A | T | C | A | T | C | G | A | A | T | G | A | T | A | T | C | A | T | T | T | C | G | A | A | T | A | A | A | C | T | G | A | T | C | G |
| --- | --- | --- | --- | --- | --- | --- | --- | --- | --- | --- | --- | --- | --- | --- | --- | --- | --- | --- | --- | --- | --- | --- | --- | --- | --- | --- | --- | --- | --- | --- | --- | --- | --- | --- | --- | --- | --- | --- | --- | --- | --- | --- | --- | --- | --- | --- | --- | --- | --- | --- | --- | --- | --- | --- | --- | --- | --- | --- | --- | --- |
| Gene_G8B10 | C | G | A | A | A | T | T | A | A | T | T | G | A | G | A | A | A | C | A | G | C | C | A | C | G | A | A | G | T | T | C | T | G | T | T | T | G | A | A | T | C | A | G | A | T | T | C | C | A | T | A | T | - | T | T | C | A | T | T | A |
|  |  |  |  |  |  |  |  |  |  |  |  |  |  |  |  |  |  |  |  |  |  |  |  |  |  |  |  |  |  |  |  |  |  |  |  |  |  |  |  |  |  |  |  |  |  |  |  |  |  |  |  |  |  |  |  |  |  |  |  |  |
| Gene_L6C11 | A | C | G | A | T | C | T | A | T | A | G | A | A | C | G | A | A | A | T | T | A | T | T | T | G | A | G | A | A | A | T | G | G | C | C | A | C | G | A | A | A | T | A | A | T | G | A | T | T | G | A | A | A | A | A | C | G | A | T | T |
| Gene_G8B10 | G | C | A | T | T | T | G | A | T | T | C | A | A | C | - | A | A | T | T | T | T | T | T | T | G | A | T | A | A | A | T | A | A | - | - | - | - | G | A | A | A | T | A | A | T | G | A | T | T | G | A | A | A | A | A | C | G | A | T | T |
|  |  |  |  |  |  |  |  |  |  |  |  |  |  |  |  |  |  |  |  |  |  |  |  |  |  |  |  |  |  |  |  |  |  |  |  |  |  |  |  |  |  |  |  |  |  |  |  |  |  |  |  |  |  |  |  |  |  |  |  |  |
| Gene_L6C11 | T | T | T | - | T | A | T | T | T | T | A | T | T | C | C | A | A | A | A | T | G | A | A | A | A | T | A | A | A | T | A | A | A | T | G | G | A | A | T | C | C | A | A | A | A | T | G | C | G | A | A | A | T | A | A | T | T | G | T | A |
| Gene_G8B10 | T | T | T | A | T | A | T | T | T | T | A | T | T | C | C | A | A | A | A | T | G | A | A | A | A | T | A | A | A | T | A | A | A | C | G | A | A | A | T | C | C | G | A | A | A | T | G | C | G | A | A | A | T | A | A | T | T | G | T | G |
|  |  |  |  |  |  |  |  |  |  |  |  |  |  |  |  |  |  |  |  |  |  |  |  |  |  |  |  |  |  |  |  |  |  |  |  |  |  |  |  |  |  |  |  |  |  |  |  |  |  |  |  |  |  |  |  |  |  |  |  |  |
| Gene_L6C11 | T | T | C | T | C | A | T | A | A | T | A | A | G | A | T | A | C | A | T | A | T | G | T | A | T | A | T | G | A | T | T | G | A | G | T | A | G | C | G | A | A | T | G | T | G | T | G | T | G | G | A | A | T | A | T | A | C | T | T | A |
| Gene_G8B10 | A | T | T | T | C | A | T | A | A | T | A | A | G | A | T | A | C | A | T | A | T | G | T | A | T | A | T | G | A | A | T | G | A | G | T | A | G | C | G | A | A | T | G | T | G | T | G | T | G | G | A | A | T | A | T | A | C | A | T | A |
|  |  |  |  |  |  |  |  |  |  |  |  |  |  |  |  |  |  |  |  |  |  |  |  |  |  |  |  |  |  |  |  |  |  |  |  |  |  |  |  |  |  |  |  |  |  |  |  |  |  |  |  |  |  |  |  |  |  |  |  |  |
| Gene_L6C11 | G | C | T | T | A | C | G | G | A | G | C | C | A | A | A | C | G | C | A | T | G | C | T | T | A | A | C | T | T | A | T | T | T | T | A | T | A | T | T | T | T | A | C | T | G | T | G | A | A | C | T | A | T | A | A | A | G | T | G | T |
| Gene_G8B10 | G | C | T | T | A | C | G | C | A | G | C | C | A | A | A | C | G | C | A | T | A | C | T | T | A | A | C | T | T | A | T | T | T | T | A | T | A | T | A | C | T | A | C | T | T | T | G | A | A | T | T | A | T | A | A | A | G | T | A | T |

**Putative TATA box**

| Gene_L6C11 | - | - | - | - | A | T | G | A | A | T | G | T | A | T | G | T | A | C | G | T | C | T | G | T | T | T | T | C | G | A | T | A | A | A | A | A | A | A | - | - | G | T | T | C | C | G | C | A | C | C | A | **T** | **A** | **T** | **A** | **A** | A | A | G | A |
| --- | --- | --- | --- | --- | --- | --- | --- | --- | --- | --- | --- | --- | --- | --- | --- | --- | --- | --- | --- | --- | --- | --- | --- | --- | --- | --- | --- | --- | --- | --- | --- | --- | --- | --- | --- | --- | --- | --- | --- | --- | --- | --- | --- | --- | --- | --- | --- | --- | --- | --- | --- | --- | --- | --- | --- | --- | --- | --- | --- | --- |
| Gene_G8B10 | T | T | A | T | A | T | G | G | A | T | G | T | A | T | G | G | A | C | G | T | C | T | G | T | T | T | T | T | G | A | T | A | A | A | A | A | A | A | A | A | G | T | T | C | C | G | C | A | C | C | G | **T** | **A** | **T** | **A** | **A** | A | A | G | A |

**├5’-UTR ├ SPCR**

| Gene_L6C11 | A | G | G | C | C | C | G | C | C | A | A | A | C | A | A | G | A | A | T | T | **G** | A | A | T | A | T | T | C | T | C | G | A | C | T | G | A | A | A | A | A | A | A | C | A | C | G | C | T | G | T | A | T | A | A | C | G | **A** | **T** | **G** | G |
| --- | --- | --- | --- | --- | --- | --- | --- | --- | --- | --- | --- | --- | --- | --- | --- | --- | --- | --- | --- | --- | --- | --- | --- | --- | --- | --- | --- | --- | --- | --- | --- | --- | --- | --- | --- | --- | --- | --- | --- | --- | --- | --- | --- | --- | --- | --- | --- | --- | --- | --- | --- | --- | --- | --- | --- | --- | --- | --- | --- | --- |
| Gene_G8B10 | A | G | G | C | C | C | A | C | C | A | A | A | C | A | A | G | A | A | T | T | **G** | A | A | T | A | T | T | C | T | C | G | T | C | T | G | A | A | T | A | A | A | A | C | A | C | G | C | T | T | T | A | T | A | A | C | G | **A** | **T** | **G** | G |

**├ Intron 1**

| Gene_L6C11 | C | A | A | A | T | A | A | A | C | T | T | T | T | T | G | **G** | **T** | G | A | G | G | A | A | C | A | T | T | T | T | T | T | T | A | A | A | T | T | T | T | A | C | T | T | C | A | A | T | A | G | A | A | A | T | C | T | A | A | T | C | A |
| --- | --- | --- | --- | --- | --- | --- | --- | --- | --- | --- | --- | --- | --- | --- | --- | --- | --- | --- | --- | --- | --- | --- | --- | --- | --- | --- | --- | --- | --- | --- | --- | --- | --- | --- | --- | --- | --- | --- | --- | --- | --- | --- | --- | --- | --- | --- | --- | --- | --- | --- | --- | --- | --- | --- | --- | --- | --- | --- | --- | --- |
| Gene_G8B10 | C | A | A | A | T | A | C | G | T | T | T | T | T | T | G | **G** | **T** | G | A | G | G | C | A | G | A | T | T | T | T | T | A | T | A | A | A | T | T | T | T | A | C | T | T | C | A | A | T | A | G | A | A | A | A | C | T | A | A | T | C | A |

**┤ ├ MPCR**

| Gene_L6C11 | A | T | T | T | T | T | G | A | A | T | T | C | **A** | **G** | T | T | T | T | T | C | T | G | T | C | A | T | T | T | G | C | C | G | C | A | T | T | A | G | T | G | G | C | C | A | G | C | G | C | A | T | G | T | G | G | G | G | C | A | C | C |
| --- | --- | --- | --- | --- | --- | --- | --- | --- | --- | --- | --- | --- | --- | --- | --- | --- | --- | --- | --- | --- | --- | --- | --- | --- | --- | --- | --- | --- | --- | --- | --- | --- | --- | --- | --- | --- | --- | --- | --- | --- | --- | --- | --- | --- | --- | --- | --- | --- | --- | --- | --- | --- | --- | --- | --- | --- | --- | --- | --- | --- |
| Gene_G8B10 | A | T | T | T | T | C | G | A | A | T | T | C | **A** | **G** | T | T | T | T | G | T | T | G | G | C | A | T | T | C | G | C | C | G | C | A | T | T | A | G | T | G | G | C | C | T | T | C | G | C | A | T | G | T | G | G | G | G | C | A | C | C |

| Gene_L6C11 | T | A | A | A | - | - | - | - | - | - | - | - | - | - | - | - | A | T | A | C | A | T | C | C | T | T | C | G | G | A | A | A | C | A | A | C | T | C | A | - | - | - | A | A | C | A | G | C | T | C | C | G | C | A | A | C | C | A | G | G |
| --- | --- | --- | --- | --- | --- | --- | --- | --- | --- | --- | --- | --- | --- | --- | --- | --- | --- | --- | --- | --- | --- | --- | --- | --- | --- | --- | --- | --- | --- | --- | --- | --- | --- | --- | --- | --- | --- | --- | --- | --- | --- | --- | --- | --- | --- | --- | --- | --- | --- | --- | --- | --- | --- | --- | --- | --- | --- | --- | --- | --- |
| Gene_G8B10 | T | A | A | A | C | A | A | G | A | A | C | A | G | A | G | A | A | A | A | A | A | T | A | G | A | G | G | G | G | G | A | A | C | T | G | C | T | G | A | T | G | C | A | A | A | A | A | A | C | C | A | A | G | A | G | C | C | A | G | A |

| Gene_L6C11 | - | A | C | A | A | A | T | G | A | C | T | A | C | C | G | A | A | C | A | C | C | A | C | C | A | C | A | A | T | C | C | A | C | - | C | A | A | C | T | G | T | T | G | G | A | G | C | A | G | A | A | A | A | G | T | G | T | T | G | C |
| --- | --- | --- | --- | --- | --- | --- | --- | --- | --- | --- | --- | --- | --- | --- | --- | --- | --- | --- | --- | --- | --- | --- | --- | --- | --- | --- | --- | --- | --- | --- | --- | --- | --- | --- | --- | --- | --- | --- | --- | --- | --- | --- | --- | --- | --- | --- | --- | --- | --- | --- | --- | --- | --- | --- | --- | --- | --- | --- | --- | --- |
| Gene_G8B10 | C | A | C | A | G | A | A | G | A | T | T | G | G | A | G | A | A | C | A | A | T | T | G | A | A | T | T | A | G | C | T | C | C | G | C | A | A | C | C | A | G | A | G | C | A | A | A | T | G | A | C | A | T | T | T | C | C | A | C | C |
|  |  |  |  |  |  |  |  |  |  |  |  |  |  |  |  |  |  |  |  |  |  |  |  |  |  |  |  |  |  |  |  |  |  |  |  |  |  |  |  |  |  |  |  |  |  |  |  |  |  |  |  |  |  |  |  |  |  |  |  |  |
| Gene_L6C11 | T | C | C | T | G | T | T | T | T | T | G | T | A | - | - | - | - | A | A | G | G | T | T | G | T | - | - | - | G | G | A | A | A | A | T | G | T | T | G | C | A | C | C | G | G | T | T | T | A | G | G | A | A | A | A | T | G | T | T | G |
| Gene_G8B10 | A | T | C | T | G | C | A | G | A | T | G | C | T | G | A | G | C | A | A | G | G | C | T | G | T | T | C | A | G | A | A | A | A | T | T | G | T | T | G | C | T | C | C | C | G | T | T | T | A | C | G | A | A | A | A | T | G | T | T | C |
|  |  |  |  |  |  |  |  |  |  |  |  |  |  |  |  |  |  |  |  |  |  |  |  |  |  |  |  |  |  |  |  |  |  |  |  |  |  |  |  |  |  |  |  |  |  |  |  |  |  |  |  |  |  |  |  |  |  |  |  |  |
| Gene_L6C11 | C | G | A | C | A | G | T | T | T | A | G | G | A | A | A | T | T | G | T | T | G | C | G | C | A | A | A | C | T | G | T | T | G | C | C | A | C | A | A | T | T | G | T | T | G | C | T | G | T | T | G | T | G | G | A | G | A | - | - | - |
| Gene_G8B10 | A | G | G | A | A | A | A | A | G | T | T | G | C | T | C | C | T | G | T | T | T | A | G | G | A | A | A | A | T | C | T | G | T | A | A | A | A | A | A | T | T | G | T | T | G | C | T | C | C | T | G | T | G | T | A | G | G | A | A | A |
|  |  |  |  |  |  |  |  |  |  |  |  |  |  |  |  |  |  |  |  |  |  |  |  |  |  |  |  |  |  |  |  |  |  |  |  |  |  |  |  |  |  |  |  |  |  |  |  |  |  |  |  |  |  |  |  |  |  |  |  |  |
| Gene_L6C11 | - | - | - | T | C | C | A | G | A | - | - | - | A | C | G | T | T | G | - | - | - | - | - | - | T | G | T | A | G | C | A | T | G | T | T | G | T | G | T | A | G | C | A | G | G | T | T | G | T | A | T | A | T | G | T | T | T | T | T | G |
| Gene_G8B10 | A | T | C | T | T | T | A | G | A | T | A | A | A | T | G | T | T | G | C | C | T | C | C | G | T | T | T | A | G | G | A | A | A | A | A | A | T | T | T | A | A | A | A | A | A | A | T | G | T | T | G | C | T | G | T | T | T | T | T | G |
|  |  |  |  |  |  |  |  |  |  |  |  |  |  |  |  |  |  |  |  |  |  |  |  |  |  |  |  |  |  |  |  |  |  |  |  |  |  |  |  |  |  |  |  |  |  |  |  |  |  |  |  |  |  |  |  |  |  |  |  |  |
| Gene_L6C11 | G | G | G | A | G | T | A | G | G | T | A | C | C | G | T | T | G | T | T | G | C | T | T | C | A | T | G | C | C | T | T | A | A | T | C | A | A | **T** | **A** | **A** | T | T | T | G | A | A | G | T | G | T | T | C | C | A | G | A | A | T | T | T |
| Gene_G8B10 | T | G | C | A | A | A | A | A | A | T | C | C | A | G | A | - | - | - | - | A | C | T | T | T | G | T | G | A | A | A | T | A | T | G | T | G | T | T | C | T | G | T | T | A | C | T | T | T | G | T | C | T | C | G | A | A | G | A | A | T |

**├ 3’-UTR**

| Gene_L6C11 | A | C | T | G | T | T | A | A | T | A | A | - | - | - | C | A | A | A | G | - | - | - | - | C | C | T | A | A | A | T | G | T | T | T | C | G | C | A | G | A | A | A | T | T | A | T | T | G | A | T | T | G | T | G | A | A | A | A | T | A |
| --- | --- | --- | --- | --- | --- | --- | --- | --- | --- | --- | --- | --- | --- | --- | --- | --- | --- | --- | --- | --- | --- | --- | --- | --- | --- | --- | --- | --- | --- | --- | --- | --- | --- | --- | --- | --- | --- | --- | --- | --- | --- | --- | --- | --- | --- | --- | --- | --- | --- | --- | --- | --- | --- | --- | --- | --- | --- | --- | --- | --- |
| Gene_G8B10 | G | T | G | G | T | **T** | **A** | **A** | G | A | A | A | G | T | T | G | A | A | G | T | G | T | T | C | C | T | A | A | A | T | G | T | T | T | C | G | C | A | G | A | A | A | T | T | A | T | T | G | A | T | T | G | T | G | A | A | A | A | T | A |
|  |  |  |  |  |  |  |  |  |  |  |  |  |  |  |  |  |  |  |  |  |  |  |  |  |  |  |  |  |  |  |  |  |  |  |  |  |  |  |  |  |  |  |  |  |  |  |  |  |  |  |  |  |  |  |  |  |  |  |  |  |
| Gene_L6C11 | T | T | G | T | G | G | A | T | T | T | T | A | T | C | G | A | A | A | T | A | A | A | A | T | T | A | C | T | G | T | A | T | T | C | T | G | - | - | - | - | - | - | - | - | - | - | - | - | - | - | - | - | - | - | - | - | - | - | - | - |
| Gene_G8B10 | T | T | G | T | G | G | A | T | T | T | T | A | T | C | G | A | A | A | T | A | A | A | A | T | T | A | C | T | C | T | A | T | T | C | T | G | A | G | A | A | T | A | T | T | C | G | T | T | T | T | T | C | C | T | A | A | T | G | A | T |
|  |  |  |  |  |  |  |  |  |  |  |  |  |  |  |  |  |  |  |  |  |  |  |  |  |  |  |  |  |  |  |  |  |  |  |  |  |  |  |  |  |  |  |  |  |  |  |  |  |  |  |  |  |  |  |  |  |  |  |  |  |
| Gene_L6C11 | - | - | - | - | - | - | - | - | - | - | - | - | - | - | - | - | - | - | - | - | - | - | - | - | - |  |  |  |  |  |  |  |  |  |  |  |  |  |  |  |  |  |  |  |  |  |  |  |  |  |  |  |  |  |  |  |  |  |  |  |
| Gene_G8B10 | A | A | A | T | G | C | T | C | G | T | T | G | C | T | G | C | A | T | C | A | T | C | A | A | T |  |  |  |  |  |  |  |  |  |  |  |  |  |  |  |  |  |  |  |  |  |  |  |  |  |  |  |  |  |  |  |  |  |  |  |

**Protein alignment:**

Intron/exon boundary

▼

| L6C11 | ***M*** | ***A*** | ***N*** | ***T*** | ***F*** | ***F*** | ***V*** | ***L*** | ***L*** | ***A*** | ***F*** | ***A*** | ***A*** | ***L*** | ***V*** | ***A*** | ***F*** | ***A*** | ***C*** | ***G*** | A | P | K | Q | E | Q | R | K | N | R | G | G | T | A | D | A | K | N | Q | E | P | D | T | E | D | W | R | T | I | E | L | A | P | Q | P | E | Q | M | T | F |
| --- | --- | --- | --- | --- | --- | --- | --- | --- | --- | --- | --- | --- | --- | --- | --- | --- | --- | --- | --- | --- | --- | --- | --- | --- | --- | --- | --- | --- | --- | --- | --- | --- | --- | --- | --- | --- | --- | --- | --- | --- | --- | --- | --- | --- | --- | --- | --- | --- | --- | --- | --- | --- | --- | --- | --- | --- | --- | --- | --- | --- |
| G8B10 | ***M*** | ***A*** | ***N*** | ***K*** | ***L*** | ***F*** | ***V*** | ***F*** | ***L*** | ***S*** | ***F*** | ***A*** | ***A*** | ***L*** | ***V*** | ***A*** | ***S*** | ***A*** | ***C*** | ***G*** | A | P | K | I | H | P | S | - | - | - | - | - | - | - | - | - | - | - | - | - | - | - | - | - | - | - | E | T | T | Q | T | A | P | Q | P | G | Q | M | T | T |
|  |  |  |  |  |  |  |  |  |  |  |  |  |  |  |  |  |  |  |  |  |  |  |  |  |  |  |  |  |  |  |  |  |  |  |  |  |  |  |  |  |  |  |  |  |  |  |  |  |  |  |  |  |  |  |  |  |  |  |  |  |
| L6C11 | P | P | S | A | D | A | E | Q | G | C | S | E | N | C | C | S | R | L | R | K | C | S | G | K | S | C | S | C | L | G | K | S | V | K | N | C | C | S | C | V | G | K | S | L | D | K | C | C | L | R | L | G | K | N | L | K | K | C | C | C |
| G8B10 | E | H | H | H | N | P | P | T | V | G | A | E | K | C | C | S | C | F | C | K | G | C | G | K | C | C | T | G | L | G | K | C | C | D | S | L | G | N | C | C | A | N | C | C | H | N | C | C | C | C | G | D | P | E | R | C | V | A | C | C |
|  |  |  |  |  |  |  |  |  |  |  |  |  |  |  |  |  |  |  |  |  |  |  |  |  |  |  |  |  |  |  |  |  |  |  |  |  |  |  |  |  |  |  |  |  |  |  |  |  |  |  |  |  |  |  |  |  |  |  |  |  |
| L6C11 | F | C | A | K | N | P | E | L | C | E | I | C | V | L | L | L | C | L | E | E | C | G |  |  |  |  |  |  |  |  |  |  |  |  |  |  |  |  |  |  |  |  |  |  |  |  |  |  |  |  |  |  |  |  |  |  |  |  |  |  |
| G8B10 | V | A | G | C | I | C | F | W | G | V | G | T | V | V | A | S | C | L | N | Q | - | - |  |  |  |  |  |  |  |  |  |  |  |  |  |  |  |  |  |  |  |  |  |  |  |  |  |  |  |  |  |  |  |  |  |  |  |  |  |  |
|  |  |  |  |  |  |  |  |  |  |  |  |  |  |  |  |  |  |  |  |  |  |  |  |  |  |  |  |  |  |  |  |  |  |  |  |  |  |  |  |  |  |  |  |  |  |  |  |  |  |  |  |  |  |  |  |  |  |  |  |  |

**D**

| Gene_L2A8L | G | A | A | A | T | G | A | A | T | T | T | G | T | T | T | A | C | G | A | A | C | T | T | C | G | A | G | C | C | T | C | C | C | A | A | A | C | A | T | A | T | G | T | A | T | T | - | G | T | A | T | T | T | G | A | A | T | T | T | G |
| --- | --- | --- | --- | --- | --- | --- | --- | --- | --- | --- | --- | --- | --- | --- | --- | --- | --- | --- | --- | --- | --- | --- | --- | --- | --- | --- | --- | --- | --- | --- | --- | --- | --- | --- | --- | --- | --- | --- | --- | --- | --- | --- | --- | --- | --- | --- | --- | --- | --- | --- | --- | --- | --- | --- | --- | --- | --- | --- | --- | --- |
| Gene_L4E10L | - | - | - | - | - | - | - | - | - | - | - | - | - | - | - | - | - | - | - | - | - | - | - | - | - | - | - | - | - | - | - | - | - | - | - | - | - | - | - | - | C | G | A | A | T | T | A | G | C | G | A | A | A | C | A | A | A | T | T | A |
|  |  |  |  |  |  |  |  |  |  |  |  |  |  |  |  |  |  |  |  |  |  |  |  |  |  |  |  |  |  |  |  |  |  |  |  |  |  |  |  |  |  |  |  |  |  |  |  |  |  |  |  |  |  |  |  |  |  |  |  |  |
| Gene_L2A8L | G | C | T | T | T | G | C | A | A | A | A | T | T | T | T | G | T | T | A | G | C | A | A | A | A | T | T | T | T | T | G | T | G | A | C | A | A | A | G | A | T | C | T | T | C | G | T | A | A | A | T | T | G | T | T | A | T | T | T | G |
| Gene_L4E10L | T | T | A | T | T | A | T | A | C | A | A | T | A | T | T | A | A | A | A | T | T | A | T | T | G | T | A | T | T | A | G | T | G | A | C | A | A | A | C | A | T | C | T | T | C | A | T | A | A | A | T | T | G | T | T | A | T | C | C | G |
|  |  |  |  |  |  |  |  |  |  |  |  |  |  |  |  |  |  |  |  |  |  |  |  |  |  |  |  |  |  |  |  |  |  |  |  |  |  |  |  |  |  |  |  |  |  |  |  |  |  |  |  |  |  |  |  |  |  |  |  |  |
| Gene_L2A8L | A | A | G | G | A | T | A | A | A | A | T | T | A | C | A | A | T | A | A | C | A | A | T | T | G | A | A | T | T | C | A | A | A | A | C | A | G | A | C | A | T | A | T | T | T | C | T | A | A | A | A | A | T | A | T | T | T | C | G | T |
| Gene_L4E10L | A | A | A | A | A | T | A | C | A | A | T | A | A | C | A | A | T | A | A | C | A | A | T | T | G | A | A | T | T | C | A | A | A | A | A | A | G | A | C | A | T | A | T | T | T | C | T | A | A | A | T | A | T | A | T | T | T | C | G | T |

**Putative TATA box ├5’-UTR**

| Gene_L2A8L | A | T | T | C | A | T | T | G | T | A | C | G | A | G | A | A | T | G | G | G | T | A | **T** | **A** | **T** | **A** | **A** | G | G | A | T | G | T | T | C | A | G | T | T | A | T | T | C | C | A | T | A | G | A | A | G | A | C | A | **G** | A | A | - | - | G |
| --- | --- | --- | --- | --- | --- | --- | --- | --- | --- | --- | --- | --- | --- | --- | --- | --- | --- | --- | --- | --- | --- | --- | --- | --- | --- | --- | --- | --- | --- | --- | --- | --- | --- | --- | --- | --- | --- | --- | --- | --- | --- | --- | --- | --- | --- | --- | --- | --- | --- | --- | --- | --- | --- | --- | --- | --- | --- | --- | --- | --- |
| Gene_L4E10L | A | T | T | C | A | T | C | G | T | G | C | G | A | G | A | A | T | G | A | G | T | A | **T** | **A** | **T** | **A** | **A** | G | G | A | C | G | T | T | C | A | G | T | T | A | T | T | C | A | G | C | A | G | A | A | G | A | C | A | **G** | A | A | C | T | A |
|  |  |  |  |  |  |  |  |  |  |  |  |  |  |  |  |  |  |  |  |  |  |  |  |  |  |  |  |  |  |  |  |  |  |  |  |  |  |  |  |  |  |  |  |  |  |  |  |  |  |  |  |  |  |  |  |  |  |  |  |  |
| Gene_L2A8L | T | T | A | G | - | T | T | A | A | C | T | G | T | T | T | A | T | T | C | T | T | T | A | T | T | G | A | A | C | G | G | T | A | T | A | A | A | T | A | C | T | A | A | A | A | A | C | T | T | C | A | G | T | T | A | T | A | C | T | A |
| Gene_L4E10L | T | T | A | A | C | T | T | A | A | T | T | G | T | T | T | A | T | T | C | T | T | T | A | G | T | A | A | A | C | G | A | C | A | T | A | A | A | T | A | C | T | A | A | A | T | A | C | T | T | C | A | G | A | T | A | T | C | T | T | A |

**├ SPCR**

| Gene_L2A8L | A | A | A | C | A | G | A | A | G | C | C | A | A | A | G | C | C | C | G | A | A | G | **A** | **T** | **G** | A | A | A | T | T | A | T | T | T | T | T | C | T | G | G | T | T | G | T | T | T | G | C | A | A | T | T | T | T | G | G | T | T | G | T |
| --- | --- | --- | --- | --- | --- | --- | --- | --- | --- | --- | --- | --- | --- | --- | --- | --- | --- | --- | --- | --- | --- | --- | --- | --- | --- | --- | --- | --- | --- | --- | --- | --- | --- | --- | --- | --- | --- | --- | --- | --- | --- | --- | --- | --- | --- | --- | --- | --- | --- | --- | --- | --- | --- | --- | --- | --- | --- | --- | --- | --- |
| Gene_L4E10L | A | A | A | T | A | G | A | A | G | C | C | A | A | G | G | C | C | T | G | A | A | G | **A** | **T** | **G** | A | A | A | T | T | A | T | T | T | T | T | C | T | G | G | T | T | G | T | T | T | G | C | A | A | T | T | T | T | G | G | T | T | G | T |

**├ Intron 1**

| Gene_L2A8L | T | G | T | A | C | A | G | **G** | **T** | A | G | A | C | A | T | T | T | A | T | C | A | A | A | A | A | G | G | A | A | A | T | T | T | C | T | T | A | A | C | G | T | T | C | A | - | T | T | A | C | G | T | A | A | T | T | T | C | G | A | T |
| --- | --- | --- | --- | --- | --- | --- | --- | --- | --- | --- | --- | --- | --- | --- | --- | --- | --- | --- | --- | --- | --- | --- | --- | --- | --- | --- | --- | --- | --- | --- | --- | --- | --- | --- | --- | --- | --- | --- | --- | --- | --- | --- | --- | --- | --- | --- | --- | --- | --- | --- | --- | --- | --- | --- | --- | --- | --- | --- | --- | --- |
| Gene_L4E10L | T | G | T | G | C | A | G | **G** | **T** | A | A | A | C | A | T | T | T | A | T | C | A | A | A | A | A | G | A | A | A | A | T | T | T | C | C | T | A | G | G | A | T | T | T | A | A | T | T | A | A | T | G | A | A | T | T | T | C | G | A | T |

**┤ ├ MPCR**

| Gene_L2A8L | C | G | A | T | T | T | T | G | T | T | A | **A** | **G** | G | T | A | C | T | C | G | T | T | C | C | A | A | G | A | G | C | A | T | C | G | G | C | T | G | G | T | C | G | C | A | A | A | A | G | - | - | - | A | A | A | A | T | T | A | G | A |
| --- | --- | --- | --- | --- | --- | --- | --- | --- | --- | --- | --- | --- | --- | --- | --- | --- | --- | --- | --- | --- | --- | --- | --- | --- | --- | --- | --- | --- | --- | --- | --- | --- | --- | --- | --- | --- | --- | --- | --- | --- | --- | --- | --- | --- | --- | --- | --- | --- | --- | --- | --- | --- | --- | --- | --- | --- | --- | --- | --- | --- |
| Gene_L4E10L | C | G | A | T | T | T | T | G | T | G | A | **A** | **G** | G | T | A | C | T | C | G | T | T | C | C | A | A | A | A | A | C | C | T | C | G | G | C | T | G | G | T | G | G | T | G | G | T | G | G | C | G | G | A | C | A | A | T | C | A | A | T |

| Gene_L2A8L | - | - | - | - | - | - | A | G | C | T | G | G | T | G | A | T | G | G | T | - | - | G | G | T | G | A | T | G | G | A | C | - | - | - | - | - | - | - | A | A | C | C | A | A | C | A | G | C | G | T | C | G | G | C | T | G | G | G | T | - |
| --- | --- | --- | --- | --- | --- | --- | --- | --- | --- | --- | --- | --- | --- | --- | --- | --- | --- | --- | --- | --- | --- | --- | --- | --- | --- | --- | --- | --- | --- | --- | --- | --- | --- | --- | --- | --- | --- | --- | --- | --- | --- | --- | --- | --- | --- | --- | --- | --- | --- | --- | --- | --- | --- | --- | --- | --- | --- | --- | --- | --- |
| Gene_L4E10L | T | G | C | G | T | C | G | G | C | T | G | G | T | G | A | T | G | A | G | A | A | G | A | A | G | A | A | G | G | A | T | T | C | A | G | T | A | A | A | A | C | C | T | G | C | G | A | C | A | T | T | G | C | T | C | G | A | G | T | T |
|  |  |  |  |  |  |  |  |  |  |  |  |  |  |  |  |  |  |  |  |  |  |  |  |  |  |  |  |  |  |  |  |  |  |  |  |  |  |  |  |  |  |  |  |  |  |  |  |  |  |  |  |  |  |  |  |  |  |  |  |  |
| Gene_L2A8L | - | - | - | - | - | - | - | - | G | T | G | G | T | G | C | T | G | A | T | G | G | A | C | C | A | C | C | A | A | T | - | - | - | - | - | - | - | - | - | A | G | C | G | T | C | G | G | C | T | G | A | T | G | G | T | - | - | - | - | - |
| Gene_L4E10L | A | A | T | T | C | G | T | A | A | C | C | A | T | T | T | T | A | G | A | A | G | T | C | C | C | C | C | A | A | C | C | G | A | G | C | T | T | T | C | A | G | T | A | T | C | G | A | T | C | A | A | A | G | G | C | A | T | A | G | A |
|  |  |  |  |  |  |  |  |  |  |  |  |  |  |  |  |  |  |  |  |  |  |  |  |  |  |  |  |  |  |  |  |  |  |  |  |  |  |  |  |  |  |  |  |  |  |  |  |  |  |  |  |  |  |  |  |  |  |  |  |  |
| Gene_L2A8L | - | - | - | - | - | - | - | - | - | - | - | - | - | T | T | T | T | A | T | G | C | - | - | - | - | - | - | - | - | G | A | G | T | C | C | G | C | C | A | C | G | C | G | A | A | T | - | - | - | - | - | - | - | - | - | - | - | - | - | - |
| Gene_L4E10L | G | A | A | G | G | T | C | C | A | G | A | A | A | T | T | T | A | A | T | G | A | A | G | C | T | C | A | A | A | G | G | A | T | C | T | A | T | G | A | T | C | T | A | A | A | T | A | T | G | G | C | G | A | A | A | G | A | G | G | C |
|  |  |  |  |  |  |  |  |  |  |  |  |  |  |  |  |  |  |  |  |  |  |  |  |  |  |  |  |  |  |  |  |  |  |  |  |  |  |  |  |  |  |  |  |  |  |  |  |  |  |  |  |  |  |  |  |  |  |  |  |  |
| Gene_L2A8L | - | - | C | G | C | A | T | A | T | C | A | A | A | T | C | C | T | T | A | G | C | A | G | A | A | C | - | - | - | - | - | C | C | C | C | A | A | G | T | T | T | A | A | A | A | G | A | A | G | C | A | T | T | - | - | - | - | A | T | T |
| Gene_L4E10L | A | G | C | G | A | G | A | G | G | A | A | A | A | A | T | C | A | T | A | G | C | G | G | A | G | C | A | G | T | A | T | C | T | C | C | A | A | G | A | A | A | C | G | C | A | A | A | A | A | A | A | T | T | T | A | A | G | A | G | A |

| Gene_L2A8L | A | A | A | G | G | C | T | A | A | C | A | G | A | - | - | - | - | - | - | - | G | A | A | C | G | A | A | A | T | - | T | C | A | A | A | - | - | - | - | - | - | A | G | C | T | T | T | T | C | C | G | G | A | A | T | C | T | **T** | **A** | **G** |
| --- | --- | --- | --- | --- | --- | --- | --- | --- | --- | --- | --- | --- | --- | --- | --- | --- | --- | --- | --- | --- | --- | --- | --- | --- | --- | --- | --- | --- | --- | --- | --- | --- | --- | --- | --- | --- | --- | --- | --- | --- | --- | --- | --- | --- | --- | --- | --- | --- | --- | --- | --- | --- | --- | --- | --- | --- | --- | --- | --- | --- |
| Gene_L4E10L | A | A | A | A | G | C | G | A | A | T | G | A | A | T | T | G | T | A | T | G | G | A | A | A | A | A | A | A | T | A | T | C | A | T | A | C | C | G | G | A | T | A | G | G | A | A | T | A | T | T | G | G | A | T | T | C | T | T | T | G |

**├ 3’-UTR**

| Gene_L2A8L | A | A | A | C | - | - | - | G | A | T | A | G | A | T | C | A | T | A | A | A | C | T | G | T | T | G | C | G | A | A | A | T | T | T | A | A | A | T | C | T | G | T | T | T | C | A | T | T | T | G | C | T | A | A | T | A | A | A | T | A |
| --- | --- | --- | --- | --- | --- | --- | --- | --- | --- | --- | --- | --- | --- | --- | --- | --- | --- | --- | --- | --- | --- | --- | --- | --- | --- | --- | --- | --- | --- | --- | --- | --- | --- | --- | --- | --- | --- | --- | --- | --- | --- | --- | --- | --- | --- | --- | --- | --- | --- | --- | --- | --- | --- | --- | --- | --- | --- | --- | --- | --- |
| Gene_L4E10L | A | A | T | C | C | T | C | A | A | **T** | **A** | **A** | A | A | T | A | T | G | A | A | G | T | G | C | T | G | C | G | A | A | A | T | T | C | A | A | A | T | C | T | G | T | T | A | C | A | T | T | T | G | C | T | A | A | T | A | A | A | T | T |
|  |  |  |  |  |  |  |  |  |  |  |  |  |  |  |  |  |  |  |  |  |  |  |  |  |  |  |  |  |  |  |  |  |  |  |  |  |  |  |  |  |  |  |  |  |  |  |  |  |  |  |  |  |  |  |  |  |  |  |  |  |
| Gene_L2A8L | T | T | C | T | T | T | A | T | T | T | C | A | T | C | G | C | A | T | T | G | T | G | C | A | T | G | A | A | A | C | A | A | G | T | G | T | A | T | T | C | T | T | C | G | T | A | T | T | T | T | T | T | T | T | T | T | G | T | C | T |
| Gene_L4E10L | T | C | C | T | T | T | A | T | T | T | C | A | T | C | G | C | A | T | T | G | T | G | C | A | T | G | A | A | A | C | A | A | T | T | G | T | A | T | T | T | T | T | T | G | T | A | T | T | T | T | T | C | T | - | - | T | G | T | C | T |
|  |  |  |  |  |  |  |  |  |  |  |  |  |  |  |  |  |  |  |  |  |  |  |  |  |  |  |  |  |  |  |  |  |  |  |  |  |  |  |  |  |  |  |  |  |  |  |  |  |  |  |  |  |  |  |  |  |  |  |  |  |
| Gene_L2A8L | T | A | T | C | G | T | T | A | A | C | A | G | C | T | T | T | A | A | T | T | A | A | A | T | T | A | T | T | G | G | G | G | C | C | T | T | A | T | T | A | T | T | T | C | A | T | C | G | T | A | A | T | G | G | A | G | A | A | C | T |
| Gene_L4E10L | T | G | C | T | G | T | T | A | A | C | A | G | C | T | T | C | A | A | C | T | G | A | A | T | A | A | T | T | T | G | G | G | C | C | T | T | A | G | T | A | T | T | T | C | A | T | C | A | T | G | T | T | G | A | A | A | A | G | T | T |
|  |  |  |  |  |  |  |  |  |  |  |  |  |  |  |  |  |  |  |  |  |  |  |  |  |  |  |  |  |  |  |  |  |  |  |  |  |  |  |  |  |  |  |  |  |  |  |  |  |  |  |  |  |  |  |  |  |  |  |  |  |
| Gene_L2A8L | T | C | C | C | G | T | A | G | T | C | C | A | C | A | T | C | G | G | A | C | G | G | G | G | T | T | T | T | T | T | T | T | T | A | T | T | A | T | C | T | T | T | A | A | T | C | T | A | A | T | C | G | A | A | T | G | A | C | T | T |
| Gene_L4E10L | G | C | T | T | G | T | A | G | C | T | C | A | C | A | T | C | G | G | A | - | G | A | A | G | T | C | T | T | C | T | T | T | T | A | T | C | A | T | T | C | T | C | C | A | T | C | T | A | A | T | C | G | A | A | T | G | A | C | T | T |
|  |  |  |  |  |  |  |  |  |  |  |  |  |  |  |  |  |  |  |  |  |  |  |  |  |  |  |  |  |  |  |  |  |  |  |  |  |  |  |  |  |  |  |  |  |  |  |  |  |  |  |  |  |  |  |  |  |  |  |  |  |
| Gene_L2A8L | T | T | T | T | T | T | C | A | C | A | C | T | C | A | C | A | C | A | A | A | T | T | G | A | A | A | A | T | G | T | G | G | T | A | - | - | A | A | A | A | T | C | A | C | A | A | G | T | C | A | C | G | G | T | T | A | A | A | T | G |
| Gene_L4E10L | T | T | T | T | C | C | A | T | G | A | A | A | A | A | T | A | A | A | A | A | A | A | A | A | A | C | T | A | G | C | G | A | C | A | G | A | A | A | A | A | T | T | A | T | A | G | G | A | T | A | A | A | - | - | - | A | A | A | T | G |
|  |  |  |  |  |  |  |  |  |  |  |  |  |  |  |  |  |  |  |  |  |  |  |  |  |  |  |  |  |  |  |  |  |  |  |  |  |  |  |  |  |  |  |  |  |  |  |  |  |  |  |  |  |  |  |  |  |  |  |  |  |
| Gene_L2A8L | T | C | A | A | C | A | T | T | C | C | C | C | C | G | A | A | A | A | G | T | T | G | A | T | A | A | T | T | T | A | G | C | A | C | G | A | A | A | C | A | G | C | T | T | T | T | C | C | A | C | C | T | A | T | T | C |  |  |  |  |
| Gene_L4E10L | T | A | T | T | C | T | T | A | G | C | C | T | T | C | G | A | A | T | T | T | C | G | A | A | - | - | - | C | C | A | C | C | G | A | A | A | C | A | C | A | T | G | T | T | T | T | G | T | A | T | T | A | - | - | - | - |  |  |  |  |

**Protein alignment:**

Intron/exon boundary

▼

| L4E10L | ***M*** | ***K*** | ***L*** | ***F*** | ***F*** | ***W*** | ***L*** | ***F*** | ***A*** | ***I*** | ***L*** | ***V*** | ***V*** | ***V*** | ***Q*** | ***V*** | ***L*** | ***V*** | ***P*** | ***K*** | ***T*** | ***S*** | ***A*** | G | G | G | G | G | Q | S | I | A | S | A | G | D | E | K | K | K | D | S | V | - | - | - | K | P | A | T | L | L | E | L | I | R | N | H | F | R |
| --- | --- | --- | --- | --- | --- | --- | --- | --- | --- | --- | --- | --- | --- | --- | --- | --- | --- | --- | --- | --- | --- | --- | --- | --- | --- | --- | --- | --- | --- | --- | --- | --- | --- | --- | --- | --- | --- | --- | --- | --- | --- | --- | --- | --- | --- | --- | --- | --- | --- | --- | --- | --- | --- | --- | --- | --- | --- | --- | --- | --- |
| L2A8L | ***M*** | ***K*** | ***L*** | ***F*** | ***F*** | ***W*** | ***L*** | ***F*** | ***A*** | ***I*** | ***L*** | ***V*** | ***V*** | ***V*** | ***Q*** | ***V*** | ***L*** | ***V*** | ***P*** | ***R*** | ***A*** | ***S*** | ***A*** | G | R | K | R | K | L | E | A | G | D | G | G | D | G | Q | P | T | A | S | A | G | C | G | A | D | G | P | P | I | A | S | A | D | G | F | Y | A |
|  |  |  |  |  |  |  |  |  |  |  |  |  |  |  |  |  |  |  |  |  |  |  |  |  |  |  |  |  |  |  |  |  |  |  |  |  |  |  |  |  |  |  |  |  |  |  |  |  |  |  |  |  |  |  |  |  |  |  |  |  |
| L4E10L | S | P | P | T | E | L | S | V | S | I | K | G | I | E | K | V | Q | K | F | N | E | A | Q | R | I | Y | D | L | N | M | A | K | E | A | A | R | G | K | I | I | A | E | Q | Y | L | Q | E | T | Q | K | N | L | R | E | K | A | N | E | L | Y |
| L2A8L | S | P | P | R | E | S | - | - | H | I | K | S | L | A | E | P | P | S | L | K | E | A | L | - | - | - | - | - | - | - | - | - | - | - | - | - | - | - | - | - | - | - | - | - | - | - | - | - | L | K | A | N | R | E | R | N | S | K | A | F |
|  |  |  |  |  |  |  |  |  |  |  |  |  |  |  |  |  |  |  |  |  |  |  |  |  |  |  |  |  |  |  |  |  |  |  |  |  |  |  |  |  |  |  |  |  |  |  |  |  |  |  |  |  |  |  |  |  |  |  |  |  |
| L4E10L | G | K | K | Y | H | T | G |  |  |  |  |  |  |  |  |  |  |  |  |  |  |  |  |  |  |  |  |  |  |  |  |  |  |  |  |  |  |  |  |  |  |  |  |  |  |  |  |  |  |  |  |  |  |  |  |  |  |  |  |  |
| L2A8L | P | E | S | - | - | - | - |  |  |  |  |  |  |  |  |  |  |  |  |  |  |  |  |  |  |  |  |  |  |  |  |  |  |  |  |  |  |  |  |  |  |  |  |  |  |  |  |  |  |  |  |  |  |  |  |  |  |  |  |  |
|  |  |  |  |  |  |  |  |  |  |  |  |  |  |  |  |  |  |  |  |  |  |  |  |  |  |  |  |  |  |  |  |  |  |  |  |  |  |  |  |  |  |  |  |  |  |  |  |  |  |  |  |  |  |  |  |  |  |  |  |  |

**E**

**--- Intron 17**

| Gene_G2D9 | A | G | A | T | A | A | T | A | A | T | A | T | A | A | A | T | A | A | A | T | A | A | A | A | A | T | A | A | A | T | C | C | A | T | T | T | C | A | G | A | T | A | A | T | T | A | T | A | A | T | G | A | A | A | C | A | A | T | T | A |
| --- | --- | --- | --- | --- | --- | --- | --- | --- | --- | --- | --- | --- | --- | --- | --- | --- | --- | --- | --- | --- | --- | --- | --- | --- | --- | --- | --- | --- | --- | --- | --- | --- | --- | --- | --- | --- | --- | --- | --- | --- | --- | --- | --- | --- | --- | --- | --- | --- | --- | --- | --- | --- | --- | --- | --- | --- | --- | --- | --- | --- |
| Gene_G3H12 | - | - | - | - | - | - | - | - | - | - | - | - | - | - | - | - | - | - | - | - | - | - | - | - | - | - | - | - | - | - | - | - | - | - | - | - | - | - | - | - | - | - | - | - | - | - | - | - | - | - | - | - | - | - | T | A | A | T | T | A |

**┤ ├ Intron 18**

| Gene_G2D9 | T | T | A | T | T | T | T | C | T | C | T | G | T | A | A | C | A | A | A | T | T | C | A | C | **A** | **G** | C | T | C | A | G | C | A | C | A | G | G | G | A | A | C | **G** | **T** | A | T | G | T | C | C | A | T | A | T | C | A | G | A | T | A | A |
| --- | --- | --- | --- | --- | --- | --- | --- | --- | --- | --- | --- | --- | --- | --- | --- | --- | --- | --- | --- | --- | --- | --- | --- | --- | --- | --- | --- | --- | --- | --- | --- | --- | --- | --- | --- | --- | --- | --- | --- | --- | --- | --- | --- | --- | --- | --- | --- | --- | --- | --- | --- | --- | --- | --- | --- | --- | --- | --- | --- | --- |
| Gene_G3H12 | T | T | A | T | T | T | T | T | T | G | T | T | C | A | A | T | A | C | A | A | T | T | A | C | **A** | **G** | C | C | C | T | A | A | C | C | T | G | A | A | T | C | A | **G** | **T** | A | A | G | T | C | C | A | T | T | T | C | A | G | A | T | A | A |

| Gene_G2D9 | T | A | A | T | A | T | T | A | A | C | C | A | A | T | C | A | T | T | - | C | C | A | A | A | A | A | T | A | A | A | T | T | T | A | G | T | A | A | T | T | T | T | A | T | A | - | A | T | A | A | T | G | A | A | A | C | A | A | T | T |
| --- | --- | --- | --- | --- | --- | --- | --- | --- | --- | --- | --- | --- | --- | --- | --- | --- | --- | --- | --- | --- | --- | --- | --- | --- | --- | --- | --- | --- | --- | --- | --- | --- | --- | --- | --- | --- | --- | --- | --- | --- | --- | --- | --- | --- | --- | --- | --- | --- | --- | --- | --- | --- | --- | --- | --- | --- | --- | --- | --- | --- |
| Gene_G3H12 | T | A | A | T | A | T | T | A | A | C | T | A | A | T | C | A | T | T | A | T | C | A | A | A | A | A | C | A | A | A | T | T | T | A | A | T | A | A | T | T | T | T | A | T | C | T | A | A | A | A | T | G | A | A | A | G | A | A | T | C |

**┤ ├ Intron 19**

| Gene_G2D9 | A | T | T | A | T | T | T | T | C | T | C | A | T | C | A | A | T | A | C | A | T | T | T | A | T | **A** | **G** | G | C | C | T | C | A | T | T | T | T | T | A | **G** | **T** | G | A | G | T | A | A | A | T | T | T | - | - | - | A | G | A | T | A | A |
| --- | --- | --- | --- | --- | --- | --- | --- | --- | --- | --- | --- | --- | --- | --- | --- | --- | --- | --- | --- | --- | --- | --- | --- | --- | --- | --- | --- | --- | --- | --- | --- | --- | --- | --- | --- | --- | --- | --- | --- | --- | --- | --- | --- | --- | --- | --- | --- | --- | --- | --- | --- | --- | --- | --- | --- | --- | --- | --- | --- | --- |
| Gene_G3H12 | A | C | T | A | T | T | T | T | C | T | C | T | T | A | A | A | T | A | C | A | T | T | T | A | T | **A** | **G** | C | C | G | T | G | T | T | T | T | T | G | T | **G** | **T** | A | A | G | T | A | A | A | T | T | T | T | A | G | A | A | A | T | A | A |

| Gene_G2D9 | T | A | A | T | A | T | T | A | A | C | C | A | A | T | C | G | T | A | A | C | A | G | A | A | A | C | A | A | A | T | T | T | A | G | T | A | A | T | T | T | T | A | T | C | T | A | T | A | A | T | G | A | A | A | G | A | A | T | T | A |
| --- | --- | --- | --- | --- | --- | --- | --- | --- | --- | --- | --- | --- | --- | --- | --- | --- | --- | --- | --- | --- | --- | --- | --- | --- | --- | --- | --- | --- | --- | --- | --- | --- | --- | --- | --- | --- | --- | --- | --- | --- | --- | --- | --- | --- | --- | --- | --- | --- | --- | --- | --- | --- | --- | --- | --- | --- | --- | --- | --- | --- |
| Gene_G3H12 | C | A | A | T | T | T | T | A | A | C | C | A | A | T | C | G | T | T | T | C | A | A | A | A | G | T | A | A | A | T | A | T | C | G | T | T | A | T | T | T | T | A | T | C | T | A | T | A | A | T | G | A | C | T | G | A | A | T | T | A |

**┤ ├ Intron 20**

| Gene_G2D9 | T | T | T | T | T | T | T | C | T | C | T | C | C | A | A | T | A | C | A | T | T | T | A | T | **A** | **G** | C | A | T | G | C | G | A | C | T | G | G | T | **G** | **T** | A | A | G | T | A | A | A | T | T | T | T | A | G | A | T | A | A | T | A | A |
| --- | --- | --- | --- | --- | --- | --- | --- | --- | --- | --- | --- | --- | --- | --- | --- | --- | --- | --- | --- | --- | --- | --- | --- | --- | --- | --- | --- | --- | --- | --- | --- | --- | --- | --- | --- | --- | --- | --- | --- | --- | --- | --- | --- | --- | --- | --- | --- | --- | --- | --- | --- | --- | --- | --- | --- | --- | --- | --- | --- | --- |
| Gene_G3H12 | T | T | A | T | T | T | T | C | T | C | T | T | T | A | A | T | A | C | A | T | T | T | A | C | **A** | **G** | C | A | A | T | G | A | G | A | T | A | C | T | **G** | **T** | A | A | G | T | A | A | A | T | T | T | T | A | A | A | T | A | A | C | A | A |

| Gene_G2D9 | A | A | T | T | A | A | C | C | A | A | T | C | C | T | T | C | C | A | A | A | A | A | A | T | A | A | T | T | C | A | G | T | A | A | T | T | T | T | A | T | C | T | T | T | A | A | T | G | A | A | A | A | A | A | T | T | A | T | C | A |
| --- | --- | --- | --- | --- | --- | --- | --- | --- | --- | --- | --- | --- | --- | --- | --- | --- | --- | --- | --- | --- | --- | --- | --- | --- | --- | --- | --- | --- | --- | --- | --- | --- | --- | --- | --- | --- | --- | --- | --- | --- | --- | --- | --- | --- | --- | --- | --- | --- | --- | --- | --- | --- | --- | --- | --- | --- | --- | --- | --- | --- |
| Gene_G3H12 | T | T | T | T | A | A | C | C | A | A | T | C | G | C | T | T | C | A | A | A | A | A | C | A | A | A | T | T | T | C | G | T | A | A | T | T | G | T | A | T | G | T | A | T | A | A | T | G | A | C | A | A | A | A | T | G | A | T | T | A |

**┤ ├ Intron 21**

| Gene_G2D9 | T | T | T | T | T | T | C | T | T | T | A | A | T | A | C | A | T | T | T | A | C | **A** | **G** | C | C | C | T | A | A | A | C | T | G | G | T | T | C | G | **G** | **T** | A | A | A | C | C | C | A | T | T | T | C | A | G | A | T | A | A | T | A | A |
| --- | --- | --- | --- | --- | --- | --- | --- | --- | --- | --- | --- | --- | --- | --- | --- | --- | --- | --- | --- | --- | --- | --- | --- | --- | --- | --- | --- | --- | --- | --- | --- | --- | --- | --- | --- | --- | --- | --- | --- | --- | --- | --- | --- | --- | --- | --- | --- | --- | --- | --- | --- | --- | --- | --- | --- | --- | --- | --- | --- | --- |
| Gene_G3H12 | T | T | G | T | C | T | C | T | T | T | T | A | T | A | T | A | T | T | T | A | C | **A** | **G** | C | C | C | T | G | A | A | C | T | G | C | A | A | C | G | **G** | **T** | A | A | G | T | C | C | A | T | T | T | T | A | A | A | T | A | A | T | A | A |

| Gene_G2D9 | T | A | T | T | A | A | C | C | A | A | T | C | A | T | C | C | C | A | A | A | C | A | T | A | A | A | T | T | T | A | G | T | A | A | T | T | T | T | A | T | C | T | A | T | A | A | T | A | A | T | G | A | A | A | G | A | A | T | T | G |
| --- | --- | --- | --- | --- | --- | --- | --- | --- | --- | --- | --- | --- | --- | --- | --- | --- | --- | --- | --- | --- | --- | --- | --- | --- | --- | --- | --- | --- | --- | --- | --- | --- | --- | --- | --- | --- | --- | --- | --- | --- | --- | --- | --- | --- | --- | --- | --- | --- | --- | --- | --- | --- | --- | --- | --- | --- | --- | --- | --- | --- |
| Gene_G3H12 | T | A | T | T | A | A | C | C | A | A | T | C | A | T | T | T | C | A | A | A | A | A | T | A | A | A | T | T | T | A | G | T | A | A | T | T | T | T | A | T | C | T | T | T | A | - | - | - | A | T | G | A | A | A | G | A | A | T | C | A |

**┤ ├ Intron 22**

| Gene_G2D9 | T | T | A | T | T | T | T | C | T | G | T | T | T | G | A | T | A | C | A | T | T | T | A | C | **A** | **G** | C | A | A | T | G | C | A | T | G | T | G | A | T | C | T | **G** | **T** | A | A | G | T | C | C | A | T | T | T | C | A | G | A | T | A | A |
| --- | --- | --- | --- | --- | --- | --- | --- | --- | --- | --- | --- | --- | --- | --- | --- | --- | --- | --- | --- | --- | --- | --- | --- | --- | --- | --- | --- | --- | --- | --- | --- | --- | --- | --- | --- | --- | --- | --- | --- | --- | --- | --- | --- | --- | --- | --- | --- | --- | --- | --- | --- | --- | --- | --- | --- | --- | --- | --- | --- | --- |
| Gene_G3H12 | T | T | A | T | T | T | T | C | T | T | T | T | C | A | A | T | A | C | A | C | T | G | A | C | **A** | **G** | C | C | T | T | T | T | A | C | T | G | G | A | A | C | G | **G** | **T** | G | A | G | T | C | C | A | T | T | T | C | A | A | A | T | A | A |

| Gene_G2D9 | A | A | A | T | T | T | C | A | A | C | C | A | A | T | C | A | T | T | C | C | A | A | A | A | A | C | A | A | T | T | T | T | A | G | T | A | A | T | A | A | T | A | T | C | T | A | A | T | A | T | G | A | A | A | C | A | A | T | T | A |
| --- | --- | --- | --- | --- | --- | --- | --- | --- | --- | --- | --- | --- | --- | --- | --- | --- | --- | --- | --- | --- | --- | --- | --- | --- | --- | --- | --- | --- | --- | --- | --- | --- | --- | --- | --- | --- | --- | --- | --- | --- | --- | --- | --- | --- | --- | --- | --- | --- | --- | --- | --- | --- | --- | --- | --- | --- | --- | --- | --- | --- |
| Gene_G3H12 | T | A | A | T | A | T | T | A | A | C | C | A | A | T | C | A | T | T | T | C | A | A | A | A | A | T | A | A | T | T | T | T | A | G | T | A | A | T | T | T | T | A | T | T | T | T | A | A | A | T | G | A | A | A | G | A | T | T | G | A |

**┤ ├ Intron 23**

| Gene_G2D9 | T | A | A | T | T | T | T | C | T | C | T | T | C | A | A | T | A | C | A | T | T | T | A | C | **A** | **G** | C | T | T | T | C | A | A | C | T | G | A | G | A | G | A | **G** | **T** | G | A | G | T | T | C | A | T | T | T | C | A | G | A | T | A | A |
| --- | --- | --- | --- | --- | --- | --- | --- | --- | --- | --- | --- | --- | --- | --- | --- | --- | --- | --- | --- | --- | --- | --- | --- | --- | --- | --- | --- | --- | --- | --- | --- | --- | --- | --- | --- | --- | --- | --- | --- | --- | --- | --- | --- | --- | --- | --- | --- | --- | --- | --- | --- | --- | --- | --- | --- | --- | --- | --- | --- | --- |
| Gene_G3H12 | T | T | A | T | T | T | T | C | T | T | T | T | A | A | A | T | A | T | A | T | T | T | T | T | G | - | - | - | - | - | - | - | - | - | - | - | - | - | - | - | - | - | - | - | - | - | - | - | - | - | - | - | - | - | - | - | - | - | - | - |

**┤**

| Gene_G2D9 | T | A | A | A | A | T | T | A | A | C | C | A | A | T | C | A | T | T | T | C | A | A | A | A | A | C | A | A | A | T | T | T | A | G | T | A | A | T | T | T | T | A | T | T | T | A | C | A | T | T | T | A | C | **A** | **G** | C | A | T | T | A |
| --- | --- | --- | --- | --- | --- | --- | --- | --- | --- | --- | --- | --- | --- | --- | --- | --- | --- | --- | --- | --- | --- | --- | --- | --- | --- | --- | --- | --- | --- | --- | --- | --- | --- | --- | --- | --- | --- | --- | --- | --- | --- | --- | --- | --- | --- | --- | --- | --- | --- | --- | --- | --- | --- | --- | --- | --- | --- | --- | --- | --- |
| Gene_G3H12 | - | - | - | - | - | - | - | - | - | - | - | - | - | - | - | - | - | - | - | - | - | - | - | - | - | - | - | - | - | - | - | - | - | - | - | - | - | - | - | - | - | - | - | - | T | A | T | A | T | T | T | A | C | **A** | **G** | C | G | C | T | C |

**├ Intron 24**

| Gene_G2D9 | G | A | G | C | G | A | A | A | C | A | **G** | **T** | A | A | G | T | C | C | A | T | T | C | C | A | C | A | T | A | A | A | A | A | T | A | A | T | A | A | C | C | A | A | T | C | A | T | T | T | C | A | A | A | A | A | C | A | A | A | A | T |
| --- | --- | --- | --- | --- | --- | --- | --- | --- | --- | --- | --- | --- | --- | --- | --- | --- | --- | --- | --- | --- | --- | --- | --- | --- | --- | --- | --- | --- | --- | --- | --- | --- | --- | --- | --- | --- | --- | --- | --- | --- | --- | --- | --- | --- | --- | --- | --- | --- | --- | --- | --- | --- | --- | --- | --- | --- | --- | --- | --- | --- |
| Gene_G3H12 | A | A | C | T | G | G | A | A | C | G | **G** | **T** | A | A | T | T | C | C | A | T | T | T | T | A | G | A | T | A | A | T | A | A | T | A | T | T | A | A | C | C | A | A | T | T | T | T | T | C | C | A | A | A | A | A | C | A | A | T | - | T |

**┤**

| Gene_G2D9 | T | C | A | G | T | A | A | T | T | T | T | A | T | C | C | T | T | A | - | T | A | A | A | A | C | A | A | T | T | A | T | A | A | T | T | T | T | C | T | C | T | T | A | A | A | T | A | C | A | T | T | C | A | C | **A** | **G** | C | T | T | T |
| --- | --- | --- | --- | --- | --- | --- | --- | --- | --- | --- | --- | --- | --- | --- | --- | --- | --- | --- | --- | --- | --- | --- | --- | --- | --- | --- | --- | --- | --- | --- | --- | --- | --- | --- | --- | --- | --- | --- | --- | --- | --- | --- | --- | --- | --- | --- | --- | --- | --- | --- | --- | --- | --- | --- | --- | --- | --- | --- | --- | --- |
| Gene_G3H12 | T | T | A | G | T | A | A | T | T | T | T | T | T | C | T | T | T | A | A | T | G | A | C | T | G | A | A | T | T | A | T | T | A | T | T | T | T | C | T | A | T | T | T | G | A | T | A | C | A | T | T | T | A | C | **A** | **G** | C | A | G | T |

**├ Intron 25**

| Gene_G2D9 | G | A | A | A | C | G | C | A | A | A | G | **G** | **T** | A | A | G | T | T | C | A | T | T | T | C | A | G | A | T | A | A | T | A | A | T | T | T | C | A | A | C | C | A | A | T | C | A | T | T | C | C | A | A | A | A | A | C | A | A | A | T |
| --- | --- | --- | --- | --- | --- | --- | --- | --- | --- | --- | --- | --- | --- | --- | --- | --- | --- | --- | --- | --- | --- | --- | --- | --- | --- | --- | --- | --- | --- | --- | --- | --- | --- | --- | --- | --- | --- | --- | --- | --- | --- | --- | --- | --- | --- | --- | --- | --- | --- | --- | --- | --- | --- | --- | --- | --- | --- | --- | --- | --- |
| Gene_G3H12 | G | T | A | G | T | G | T | C | A | A | C | **G** | **T** | A | A | G | T | C | C | A | C | T | A | C | A | A | A | T | A | A | T | T | G | T | A | T | T | A | A | C | C | A | A | T | C | A | T | T | C | C | A | A | A | A | A | T | G | A | A | T |

**┤**

| Gene_G2D9 | T | T | A | G | T | A | A | T | T | T | T | A | T | C | T | T | C | A | A | T | A | A | G | T | G | A | A | A | T | A | T | T | A | A | T | T | T | C | T | C | T | T | A | A | A | T | A | C | A | T | T | T | A | C | **A** | **G** | C | C | C | T |
| --- | --- | --- | --- | --- | --- | --- | --- | --- | --- | --- | --- | --- | --- | --- | --- | --- | --- | --- | --- | --- | --- | --- | --- | --- | --- | --- | --- | --- | --- | --- | --- | --- | --- | --- | --- | --- | --- | --- | --- | --- | --- | --- | --- | --- | --- | --- | --- | --- | --- | --- | --- | --- | --- | --- | --- | --- | --- | --- | --- | --- |
| Gene_G3H12 | T | T | A | A | T | G | A | T | T | T | T | A | T | C | T | T | C | A | A | T | G | A | G | A | G | A | A | T | T | A | T | T | A | A | T | T | T | C | T | T | T | T | C | A | A | T | A | C | A | T | T | T | A | C | **A** | **G** | C | G | C | T |

**├ Intron 26**

| Gene_G2D9 | G | C | A | G | C | G | C | A | A | A | G | **G** | **T** | A | A | G | T | C | A | A | T | T | T | C | A | G | A | T | A | A | T | T | A | T | T | T | T | G | A | C | C | A | A | T | C | A | T | T | T | C | A | A | A | A | A | T | A | A | A | T |
| --- | --- | --- | --- | --- | --- | --- | --- | --- | --- | --- | --- | --- | --- | --- | --- | --- | --- | --- | --- | --- | --- | --- | --- | --- | --- | --- | --- | --- | --- | --- | --- | --- | --- | --- | --- | --- | --- | --- | --- | --- | --- | --- | --- | --- | --- | --- | --- | --- | --- | --- | --- | --- | --- | --- | --- | --- | --- | --- | --- | --- |
| Gene_G3H12 | G | A | A | G | G | T | G | A | T | C | T | **G** | **T** | A | A | G | T | C | C | A | T | T | T | C | A | A | A | T | A | C | A | A | A | T | T | T | A | G | G | C | C | A | A | C | C | A | T | T | C | C | A | A | A | A | G | T | - | - | - | T |

**┤**

| Gene_G2D9 | T | T | A | G | T | A | A | T | T | T | T | A | T | C | T | T | T | A | A | T | G | G | A | A | G | A | A | T | T | A | T | T | T | T | G | T | T | C | T | T | T | T | T | A | A | A | A | C | A | T | T | T | A | C | **A** | **G** | C | G | T | T |
| --- | --- | --- | --- | --- | --- | --- | --- | --- | --- | --- | --- | --- | --- | --- | --- | --- | --- | --- | --- | --- | --- | --- | --- | --- | --- | --- | --- | --- | --- | --- | --- | --- | --- | --- | --- | --- | --- | --- | --- | --- | --- | --- | --- | --- | --- | --- | --- | --- | --- | --- | --- | --- | --- | --- | --- | --- | --- | --- | --- | --- |
| Gene_G3H12 | T | C | A | G | T | T | A | T | T | T | T | A | C | C | T | A | T | A | A | T | G | A | A | A | G | A | A | T | T | A | T | T | A | A | T | T | T | C | T | C | C | T | T | A | A | - | - | - | - | - | - | - | - | - | - | - | - | - | - | - |

**├ Intron 27**

| Gene_G2D9 | G | A | A | T | G | T | G | A | T | C | T | **G** | **T** | A | A | G | T | C | A | A | T | T | T | C | A | G | A | T | A | A | T | A | A | T | T | T | T | A | T | T | T | A | A | T | A | A | T | G | A | G | G | G | A | A | T | T | A | T | A | A |
| --- | --- | --- | --- | --- | --- | --- | --- | --- | --- | --- | --- | --- | --- | --- | --- | --- | --- | --- | --- | --- | --- | --- | --- | --- | --- | --- | --- | --- | --- | --- | --- | --- | --- | --- | --- | --- | --- | --- | --- | --- | --- | --- | --- | --- | --- | --- | --- | --- | --- | --- | --- | --- | --- | --- | --- | --- | --- | --- | --- | --- |
| Gene_G3H12 | - | - | - | - | - | - | - | - | - | - | - | - | - | - | - | - | - | - | - | - | - | - | - | - | - | - | - | - | - | - | - | - | - | - | - | - | - | - | - | - | - | - | - | - | - | - | - | - | - | - | - | - | - | - | - | - | - | - | - | - |

**┤ ├ Intron 28**

| Gene_G2D9 | T | T | T | T | C | T | C | T | T | T | A | A | T | A | C | C | T | T | T | A | C | **A** | **G** | C | T | C | T | G | A | A | T | T | A | T | T | **G** | **T** | A | A | G | T | A | A | A | T | T | T | T | A | G | A | T | A | A | T | A | A | T | A | T |
| --- | --- | --- | --- | --- | --- | --- | --- | --- | --- | --- | --- | --- | --- | --- | --- | --- | --- | --- | --- | --- | --- | --- | --- | --- | --- | --- | --- | --- | --- | --- | --- | --- | --- | --- | --- | --- | --- | --- | --- | --- | --- | --- | --- | --- | --- | --- | --- | --- | --- | --- | --- | --- | --- | --- | --- | --- | --- | --- | --- | --- |
| Gene_G3H12 | - | - | - | - | - | - | - | - | T | T | A | T | T | A | C | A | T | T | T | A | C | **A** | **G** | C | G | C | T | G | A | A | T | T | A | C | T | **G** | **T** | A | A | A | T | A | A | A | T | T | T | T | A | G | A | T | A | A | T | A | A | T | T | T |

| Gene_G2D9 | T | A | A | C | C | A | A | T | C | A | A | T | C | C | A | A | A | A | A | C | G | A | A | A | T | T | A | G | T | A | A | T | T | T | T | A | T | C | T | T | T | A | A | T | G | G | A | A | A | A | A | T | T | A | T | T | A | T | T | T |
| --- | --- | --- | --- | --- | --- | --- | --- | --- | --- | --- | --- | --- | --- | --- | --- | --- | --- | --- | --- | --- | --- | --- | --- | --- | --- | --- | --- | --- | --- | --- | --- | --- | --- | --- | --- | --- | --- | --- | --- | --- | --- | --- | --- | --- | --- | --- | --- | --- | --- | --- | --- | --- | --- | --- | --- | --- | --- | --- | --- | --- |
| Gene_G3H12 | T | A | A | C | C | A | A | T | C | A | T | T | C | C | A | A | G | A | A | T | A | A | A | T | T | T | A | G | T | A | A | T | T | T | T | G | T | - | - | - | - | A | G | T | A | A | A | C | G | A | T | T | T | A | T | T | A | T | T | T |

**┤ ├ Intron 29**

| Gene_G2D9 | T | C | A | T | T | T | T | A | A | T | A | C | A | T | T | T | A | C | **A** | **G** | C | T | T | T | G | T | A | C | T | G | G | C | A | C | G | G | T | T | A | **G** | **T** | C | C | A | T | T | T | C | A | G | A | T | A | A | T | A | A | T | A | A |
| --- | --- | --- | --- | --- | --- | --- | --- | --- | --- | --- | --- | --- | --- | --- | --- | --- | --- | --- | --- | --- | --- | --- | --- | --- | --- | --- | --- | --- | --- | --- | --- | --- | --- | --- | --- | --- | --- | --- | --- | --- | --- | --- | --- | --- | --- | --- | --- | --- | --- | --- | --- | --- | --- | --- | --- | --- | --- | --- | --- | --- |
| Gene_G3H12 | T | C | C | T | T | T | C | A | A | T | A | C | A | T | T | T | A | C | **A** | **G** | C | G | T | T | G | A | A | C | T | G | T | C | A | C | G | G | T | T | A | **G** | **T** | C | T | A | T | T | T | T | A | G | A | T | A | A | T | A | A | T | - | - |

| Gene_G2D9 | C | A | T | T | A | A | G | C | A | A | T | C | A | T | T | C | C | A | A | A | A | A | C | A | A | A | T | T | T | A | G | T | A | A | T | A | T | T | A | T | C | T | A | T | G | T | A | T | A | A | T | G | A | A | A | A | A | A | C | T |
| --- | --- | --- | --- | --- | --- | --- | --- | --- | --- | --- | --- | --- | --- | --- | --- | --- | --- | --- | --- | --- | --- | --- | --- | --- | --- | --- | --- | --- | --- | --- | --- | --- | --- | --- | --- | --- | --- | --- | --- | --- | --- | --- | --- | --- | --- | --- | --- | --- | --- | --- | --- | --- | --- | --- | --- | --- | --- | --- | --- | --- |
| Gene_G3H12 | - | A | T | T | A | A | C | C | A | A | T | C | A | T | T | T | C | A | A | A | A | A | T | A | A | A | T | T | T | A | G | T | A | A | T | T | T | T | A | T | - | - | - | - | - | - | - | - | A | A | T | G | A | C | A | A | A | A | T | G |

**┤ ├ Intron 30**

| Gene_G2D9 | A | T | T | A | T | T | T | T | C | T | C | T | T | T | A | A | T | A | C | A | T | T | T | A | C | **A** | **G** | C | A | A | T | G | C | A | G | C | G | C | A | A | C | G | G | T | A | A | **G** | **T** | G | C | A | T | T | T | C | A | G | A | T | A |
| --- | --- | --- | --- | --- | --- | --- | --- | --- | --- | --- | --- | --- | --- | --- | --- | --- | --- | --- | --- | --- | --- | --- | --- | --- | --- | --- | --- | --- | --- | --- | --- | --- | --- | --- | --- | --- | --- | --- | --- | --- | --- | --- | --- | --- | --- | --- | --- | --- | --- | --- | --- | --- | --- | --- | --- | --- | --- | --- | --- | --- |
| Gene_G3H12 | A | T | T | A | A | T | T | T | C | T | C | C | T | T | T | A | T | A | C | A | T | T | T | A | C | **A** | **G** | T | T | C | T | G | C | A | G | C | G | G | A | A | C | G | G | T | A | A | **G** | **T** | C | C | A | T | T | T | C | A | G | A | T | A |

| Gene_G2D9 | A | T | A | A | C | A | T | T | A | A | A | C | A | A | T | C | A | T | - | - | - | T | C | T | A | A | A | G | A | C | G | A | A | A | T | T | A | G | T | T | A | A | T | T | - | A | T | C | T | A | T | A | A | T | G | A | A | A | G | A |
| --- | --- | --- | --- | --- | --- | --- | --- | --- | --- | --- | --- | --- | --- | --- | --- | --- | --- | --- | --- | --- | --- | --- | --- | --- | --- | --- | --- | --- | --- | --- | --- | --- | --- | --- | --- | --- | --- | --- | --- | --- | --- | --- | --- | --- | --- | --- | --- | --- | --- | --- | --- | --- | --- | --- | --- | --- | --- | --- | --- | --- |
| Gene_G3H12 | A | T | A | A | T | A | T | A | A | A | A | C | A | A | T | A | G | T | A | G | T | T | C | C | A | A | A | A | A | C | A | T | A | T | T | T | A | G | T | A | A | T | T | T | T | A | T | C | C | T | T | A | A | T | G | A | A | A | G | A |

**┤ ├ Intron 31**

| Gene_G2D9 | A | T | T | A | T | T | A | T | T | T | T | C | T | T | T | T | T | A | A | T | A | C | A | T | T | T | A | C | **A** | **G** | C | - | - | - | C | G | T | G | C | G | A | T | A | C | T | **G** | **T** | A | A | G | T | C | G | A | T | T | T | C | A | G |
| --- | --- | --- | --- | --- | --- | --- | --- | --- | --- | --- | --- | --- | --- | --- | --- | --- | --- | --- | --- | --- | --- | --- | --- | --- | --- | --- | --- | --- | --- | --- | --- | --- | --- | --- | --- | --- | --- | --- | --- | --- | --- | --- | --- | --- | --- | --- | --- | --- | --- | --- | --- | --- | --- | --- | --- | --- | --- | --- | --- | --- |
| Gene_G3H12 | A | T | T | A | T | T | A | T | T | T | T | C | T | C | T | T | T | G | A | T | A | C | A | T | T | T | A | T | **A** | **G** | C | G | C | T | C | A | A | C | T | G | A | A | A | A | T | **G** | **T** | A | A | G | T | C | C | A | T | T | T | T | A | G |

| Gene_G2D9 | A | T | A | A | T | A | A | T | A | T | T | A | A | A | T | A | A | T | C | A | T | T | C | A | A | A | A | A | A | C | A | A | A | T | T | T | A | G | C | A | A | T | T | T | T | A | T | C | T | A | T | A | A | T | G | A | - | - | - | A |
| --- | --- | --- | --- | --- | --- | --- | --- | --- | --- | --- | --- | --- | --- | --- | --- | --- | --- | --- | --- | --- | --- | --- | --- | --- | --- | --- | --- | --- | --- | --- | --- | --- | --- | --- | --- | --- | --- | --- | --- | --- | --- | --- | --- | --- | --- | --- | --- | --- | --- | --- | --- | --- | --- | --- | --- | --- | --- | --- | --- | --- |
| Gene_G3H12 | A | T | A | A | T | A | A | T | A | T | T | A | A | T | G | A | A | T | C | A | T | T | C | C | A | A | A | A | C | C | A | A | A | T | T | T | A | G | T | A | G | T | T | T | T | A | T | C | T | A | T | A | A | T | A | A | T | A | A | A |

**┤ ├ Intron 32**

| Gene_G2D9 | A | G | A | A | T | T | T | T | T | T | C | T | T | T | T | G | A | T | A | T | A | C | - | A | T | T | T | A | A | **A** | **G** | C | G | C | T | T | C | A | C | G | G | G | A | A | C | T | **G** | **T** | A | A | G | T | C | C | A | T | T | T | C | A |
| --- | --- | --- | --- | --- | --- | --- | --- | --- | --- | --- | --- | --- | --- | --- | --- | --- | --- | --- | --- | --- | --- | --- | --- | --- | --- | --- | --- | --- | --- | --- | --- | --- | --- | --- | --- | --- | --- | --- | --- | --- | --- | --- | --- | --- | --- | --- | --- | --- | --- | --- | --- | --- | --- | --- | --- | --- | --- | --- | --- | --- |
| Gene_G3H12 | A | G | A | A | T | T | A | T | T | A | T | T | T | T | C | T | C | T | T | T | A | T | T | A | T | T | T | A | T | **A** | **G** | C | T | A | T | T | T | T | C | C | G | G | C | T | C | T | **G** | **T** | A | T | G | T | A | C | A | T | T | T | C | A |

| Gene_G2D9 | G | A | T | A | A | T | A | A | T | A | T | T | - | A | A | C | C | A | A | C | A | A | T | T | C | C | A | A | A | A | A | T | A | A | A | T | T | T | A | G | T | A | A | T | T | T | T | A | T | A | C | T | A | A | A | A | G | A | A | T |
| --- | --- | --- | --- | --- | --- | --- | --- | --- | --- | --- | --- | --- | --- | --- | --- | --- | --- | --- | --- | --- | --- | --- | --- | --- | --- | --- | --- | --- | --- | --- | --- | --- | --- | --- | --- | --- | --- | --- | --- | --- | --- | --- | --- | --- | --- | --- | --- | --- | --- | --- | --- | --- | --- | --- | --- | --- | --- | --- | --- | --- |
| Gene_G3H12 | G | A | T | A | A | A | A | A | T | A | T | T | C | A | A | C | C | A | A | T | C | A | T | T | C | C | A | A | A | A | A | C | A | G | A | T | T | T | A | G | T | A | A | T | T | T | T | A | T | C | T | A | T | A | A | T | G | A | A | **A** |

**┤ ├ Intron 33**

| Gene_G2D9 | T | T | T | T | T | A | T | T | A | T | C | T | A | T | T | T | T | C | T | A | T | T | T | A | T | **A** | **G** | T | - | - | - | - | - | - | - | - | - | A | C | T | C | G | A | C | A | G | C | A | T | A | A | **G** | **T** | G | A | G | T | T | C | A |
| --- | --- | --- | --- | --- | --- | --- | --- | --- | --- | --- | --- | --- | --- | --- | --- | --- | --- | --- | --- | --- | --- | --- | --- | --- | --- | --- | --- | --- | --- | --- | --- | --- | --- | --- | --- | --- | --- | --- | --- | --- | --- | --- | --- | --- | --- | --- | --- | --- | --- | --- | --- | --- | --- | --- | --- | --- | --- | --- | --- | --- |
| Gene_G3H12 | **G** | A | A | T | T | A | T | C | A | T | T | T | T | T | T | T | T | T | T | A | A | A | T | A | C | A | G | T | T | A | C | A | C | T | A | G | G | A | C | T | C | C | A | A | A | G | G | A | T | A | T | **G** | **T** | A | A | A | T | T | C | A |

| Gene_G2D9 | T | T | T | C | A | G | A | T | A | A | T | A | A | T | A | T | T | A | A | C | C | A | A | T | C | A | T | T | C | T | A | A | A | A | A | T | A | A | A | T | T | T | A | G | T | A | A | T | T | T | G | A | T | C | T | A | T | A | A | T |
| --- | --- | --- | --- | --- | --- | --- | --- | --- | --- | --- | --- | --- | --- | --- | --- | --- | --- | --- | --- | --- | --- | --- | --- | --- | --- | --- | --- | --- | --- | --- | --- | --- | --- | --- | --- | --- | --- | --- | --- | --- | --- | --- | --- | --- | --- | --- | --- | --- | --- | --- | --- | --- | --- | --- | --- | --- | --- | --- | --- | --- |
| Gene_G3H12 | T | T | T | C | A | G | A | T | A | A | T | A | A | T | A | T | T | A | A | C | C | A | T | T | C | A | T | T | C | C | A | A | A | A | A | C | A | A | A | T | T | T | A | A | T | A | A | T | T | T | - | - | - | - | T | A | T | A | T | T |

**┤ ├ Intron 34**

| Gene_G2D9 | T | A | A | C | A | A | A | T | T | A | T | T | A | T | T | T | T | C | T | C | T | T | T | A | A | C | A | C | A | T | T | A | A | C | **A** | **G** | G | A | C | T | G | A | C | T | T | G | G | G | T | C | T | **G** | **T** | A | A | G | T | C | T | T |
| --- | --- | --- | --- | --- | --- | --- | --- | --- | --- | --- | --- | --- | --- | --- | --- | --- | --- | --- | --- | --- | --- | --- | --- | --- | --- | --- | --- | --- | --- | --- | --- | --- | --- | --- | --- | --- | --- | --- | --- | --- | --- | --- | --- | --- | --- | --- | --- | --- | --- | --- | --- | --- | --- | --- | --- | --- | --- | --- | --- | --- |
| Gene_G3H12 | G | T | A | T | G | A | A | T | T | A | T | T | A | A | T | T | T | C | T | C | T | T | A | A | A | T | A | C | A | A | T | T | A | C | **A** | **G** | - | - | C | T | T | A | A | T | - | - | A | T | T | C | C | T | A | G | A | **G** | **T** | A | A | G |

| Gene_G2D9 | T | T | T | T | A | T | A | T | A | A | T | A | A | T | A | T | T | A | A | T | C | A | T | A | T | C | A | T | T | C | C | A | A | A | A | A | C | A | A | A | T | T | T | **A** | **G** | T | A | A | A | T | T | T | G | T | A | A | T | G | A | A |
| --- | --- | --- | --- | --- | --- | --- | --- | --- | --- | --- | --- | --- | --- | --- | --- | --- | --- | --- | --- | --- | --- | --- | --- | --- | --- | --- | --- | --- | --- | --- | --- | --- | --- | --- | --- | --- | --- | --- | --- | --- | --- | --- | --- | --- | --- | --- | --- | --- | --- | --- | --- | --- | --- | --- | --- | --- | --- | --- | --- | --- |
| Gene_G3H12 | T | T | T | C | A | G | A | T | A | A | T | A | A | T | A | T | T | A | T | G | C | A | - | A | T | C | A | T | T | C | A | A | A | A | A | A | C | A | A | T | T | T | T | A | G | T | A | A | A | T | T | T | A | T | - | - | C | T | A | T |

**┤ ├ Intron 35**

| Gene_G2D9 | A | C | A | A | T | T | A | T | C | A | T | T | T | T | T | C | T | T | T | C | A | T | A | C | **A** | **G** | T | T | A | C | A | C | C | A | C | T | C | C | A | A | G | G | G | A | T | C | T | **G** | **T** | A | A | G | T | C | C | A | C | T | T | T |
| --- | --- | --- | --- | --- | --- | --- | --- | --- | --- | --- | --- | --- | --- | --- | --- | --- | --- | --- | --- | --- | --- | --- | --- | --- | --- | --- | --- | --- | --- | --- | --- | --- | --- | --- | --- | --- | --- | --- | --- | --- | --- | --- | --- | --- | --- | --- | --- | --- | --- | --- | --- | --- | --- | --- | --- | --- | --- | --- | --- | --- |
| Gene_G3H12 | T | C | A | A | A | T | G | A | T | T | C | **A** | **G** | T | T | C | A | C | G | A | G | T | G | G | A | A | T | T | G | - | A | G | T | G | C | T | T | T | A | A | A | A | G | A | - | - | - | **G** | **T** | A | A | G | T | T | - | - | - | - | - | - |

| Gene_G2D9 | A | G | A | T | A | A | T | G | A | T | A | C | A | A | A | C | G | A | A | T | C | A | T | T | C | C | A | A | A | A | A | C | A | A | T | T | T | T | A | G | T | A | A | C | T | T | T | A | T | T | A | T | G | A | A | A | G | A | A | T |
| --- | --- | --- | --- | --- | --- | --- | --- | --- | --- | --- | --- | --- | --- | --- | --- | --- | --- | --- | --- | --- | --- | --- | --- | --- | --- | --- | --- | --- | --- | --- | --- | --- | --- | --- | --- | --- | --- | --- | --- | --- | --- | --- | --- | --- | --- | --- | --- | --- | --- | --- | --- | --- | --- | --- | --- | --- | --- | --- | --- | --- |
| Gene_G3H12 | - | - | - | T | G | A | T | G | A | T | T | T | T | T | A | T | A | T | A | T | T | T | T | T | C | C | G | A | A | T | - | - | - | T | T | T | A | T | A | G | A | A | A | G | T | C | G | A | T | T | C | T | A | A | A | T | C | A | A | G |

**┤ ├ Intron 36**

| Gene_G2D9 | T | A | T | G | A | T | T | T | T | C | T | C | T | T | T | G | A | T | A | C | A | T | T | T | A | C | **A** | **G** | C | A | C | T | G | T | G | A | T | A | C | C | **G** | **T** | A | A | G | T | C | C | A | T | T | T | T | A | G | A | C | A | A | T |
| --- | --- | --- | --- | --- | --- | --- | --- | --- | --- | --- | --- | --- | --- | --- | --- | --- | --- | --- | --- | --- | --- | --- | --- | --- | --- | --- | --- | --- | --- | --- | --- | --- | --- | --- | --- | --- | --- | --- | --- | --- | --- | --- | --- | --- | --- | --- | --- | --- | --- | --- | --- | --- | --- | --- | --- | --- | --- | --- | --- | --- |
| Gene_G3H12 | C | - | - | - | - | - | - | - | - | - | - | - | - | - | - | G | G | C | G | C | A | - | - | - | - | - | A | G | C | G | A | T | A | C | G | A | A | A | - | - | - | - | - | - | - | - | - | - | - | - | T | C | T | G | T | A | C | G | G | A |

| Gene_G2D9 | A | A | T | A | T | T | A | A | T | T | A | A | T | C | A | T | T | T | C | A | A | A | A | A | C | A | A | T | T | A | A | A | T | A | A | A | A | A | T | A | A | T | T | T | T | A | T | C | T | A | T | T | C | A | A | A | T | G | A | T |
| --- | --- | --- | --- | --- | --- | --- | --- | --- | --- | --- | --- | --- | --- | --- | --- | --- | --- | --- | --- | --- | --- | --- | --- | --- | --- | --- | --- | --- | --- | --- | --- | --- | --- | --- | --- | --- | --- | --- | --- | --- | --- | --- | --- | --- | --- | --- | --- | --- | --- | --- | --- | --- | --- | --- | --- | --- | --- | --- | --- | --- |
| Gene_G3H12 | A | A | A | A | T | C | G | G | A | G | A | A | T | C | G | A | T | A | C | G | - | - | - | - | - | - | - | - | T | A | T | G | T | A | C | G | G | A | G | A | - | - | T | T | T | A | T | G | T | A | T | G | C | C | G | T | A | C | A | T |

| Gene_G2D9 | A | A | T | A | T | T | A | A | T | T | A | A | T | C | A | T | T | T | C | A | A | A | A | A | C | A | A | T | T | A | A | A | T | A | A | A | A | A | T | A | A | T | T | T | T | A | T | C | T | A | T | T | C | A | A | A | T | G | A | T |
| --- | --- | --- | --- | --- | --- | --- | --- | --- | --- | --- | --- | --- | --- | --- | --- | --- | --- | --- | --- | --- | --- | --- | --- | --- | --- | --- | --- | --- | --- | --- | --- | --- | --- | --- | --- | --- | --- | --- | --- | --- | --- | --- | --- | --- | --- | --- | --- | --- | --- | --- | --- | --- | --- | --- | --- | --- | --- | --- | --- | --- |
| Gene_G3H12 | A | A | A | A | T | C | G | G | A | G | A | A | T | C | G | A | T | A | C | G | - | - | - | - | - | - | - | - | T | A | T | G | T | A | C | G | G | A | G | A | - | - | T | T | T | A | T | G | T | A | T | G | C | C | G | T | A | C | A | T |

**┤ ├ Intron 37**

| Gene_G2D9 | T | C | **A** | **G** | T | A | C | A | C | C | A | G | A | G | G | A | G | A | G | G | A | A | T | A | C | T | T | T | A | A | T | G | A | A | **G** | **T** | A | A | G | T | T | T | G | A | T | A | A | T | T | T | T | C | A | T | A | T | A | T | T | T |
| --- | --- | --- | --- | --- | --- | --- | --- | --- | --- | --- | --- | --- | --- | --- | --- | --- | --- | --- | --- | --- | --- | --- | --- | --- | --- | --- | --- | --- | --- | --- | --- | --- | --- | --- | --- | --- | --- | --- | --- | --- | --- | --- | --- | --- | --- | --- | --- | --- | --- | --- | --- | --- | --- | --- | --- | --- | --- | --- | --- | --- |
| Gene_G3H12 | T | T | G | C | A | A | T | T | T | - | - | - | - | - | - | - | - | A | T | G | C | A | T | A | C | A | T | T | T | G | T | A | C | C | G | A | T | T | C | T | C | C | G | A | T | T | - | T | T | T | C | C | G | T | A | C | G | G | A | T |

**┤**

| Gene_G2D9 | T | T | C | C | G | A | A | T | T | T | T | A | T | G | A | A | A | A | A | T | C | G | A | T | T | C | T | A | A | A | T | T | T | G | T | T | T | A | T | T | C | C | **A** | **G** | G | G | C | T | C | T | C | C | G | A | A | T | T | T | T | T |
| --- | --- | --- | --- | --- | --- | --- | --- | --- | --- | --- | --- | --- | --- | --- | --- | --- | --- | --- | --- | --- | --- | --- | --- | --- | --- | --- | --- | --- | --- | --- | --- | --- | --- | --- | --- | --- | --- | --- | --- | --- | --- | --- | --- | --- | --- | --- | --- | --- | --- | --- | --- | --- | --- | --- | --- | --- | --- | --- | --- | --- |
| Gene_G3H12 | T | T | C | - | G | T | A | T | C | G | T | T | T | G | C | G | G | T | - | C | C | T | G | T | T | C | T | A | A | A | T | T | T | C | T | T | T | G | T | T | T | C | **A** | **G** | T | G | T | T | C | T | T | C | G | G | A | T | T | T | T | T |

**├ Intron 38**

| Gene_G2D9 | A | T | T | A | G | T | C | C | C | C | A | A | A | A | A | A | A | T | G | A | T | T | T | C | A | A | G | A | G | A | A | T | **G** | **T** | A | A | A | T | T | T | T | A | T | T | T | T | C | C | C | C | A | C | C | C | G | C | A | G | A | T |
| --- | --- | --- | --- | --- | --- | --- | --- | --- | --- | --- | --- | --- | --- | --- | --- | --- | --- | --- | --- | --- | --- | --- | --- | --- | --- | --- | --- | --- | --- | --- | --- | --- | --- | --- | --- | --- | --- | --- | --- | --- | --- | --- | --- | --- | --- | --- | --- | --- | --- | --- | --- | --- | --- | --- | --- | --- | --- | --- | --- | --- |
| Gene_G3H12 | A | T | T | G | G | T | C | C | C | A | G | A | A | A | A | G | G | A | G | A | T | T | T | C | A | A | G | A | G | A | A | T | **G** | **T** | A | A | A | T | T | T | T | G | T | T | T | T | T | C | C | C | A | A | C | C | G | C | A | T | A | T |

**┤**

| Gene_G2D9 | A | A | T | T | G | C | A | A | T | T | A | T | T | T | C | A | T | T | A | C | A | T | T | T | T | T | - | - | T | A | T | T | C | T | T | T | T | T | T | **A** | **G** | T | T | T | G | A | T | G | G | A | T | A | C | A | A | G | C | G | T | T |
| --- | --- | --- | --- | --- | --- | --- | --- | --- | --- | --- | --- | --- | --- | --- | --- | --- | --- | --- | --- | --- | --- | --- | --- | --- | --- | --- | --- | --- | --- | --- | --- | --- | --- | --- | --- | --- | --- | --- | --- | --- | --- | --- | --- | --- | --- | --- | --- | --- | --- | --- | --- | --- | --- | --- | --- | --- | --- | --- | --- | --- |
| Gene_G3H12 | A | A | T | T | G | C | A | A | T | T | A | T | T | T | G | A | T | T | A | T | A | T | T | T | T | T | A | T | T | T | T | C | T | T | T | T | T | C | T | **A** | **G** | T | T | T | G | A | T | T | A | A | C | T | G | T | G | G | C | A | T | T |

**├ Intron 39**

| Gene_G2D9 | T | G | C | A | A | **G** | **T** | A | A | G | T | T | T | A | T | A | A | C | A | G | A | A | T | A | T | A | A | A | T | T | T | A | A | A | T | T | C | A | A | A | A | C | T | G | A | T | A | G | A | A | A | C | A | T | T | C | A | T | C | A |
| --- | --- | --- | --- | --- | --- | --- | --- | --- | --- | --- | --- | --- | --- | --- | --- | --- | --- | --- | --- | --- | --- | --- | --- | --- | --- | --- | --- | --- | --- | --- | --- | --- | --- | --- | --- | --- | --- | --- | --- | --- | --- | --- | --- | --- | --- | --- | --- | --- | --- | --- | --- | --- | --- | --- | --- | --- | --- | --- | --- | --- |
| Gene_G3H12 | T | G | C | G | A | **G** | **T** | A | A | G | T | T | T | A | T | G | A | C | A | A | A | A | G | A | T | A | A | A | T | T | A | A | A | A | T | T | C | A | A | A | A | C | T | G | A | T | A | - | - | A | A | C | A | T | T | C | A | T | C | A |

**┤ ├Intron 40**

| Gene_G2D9 | C | A | A | A | T | - | - | T | A | A | T | T | T | T | T | G | T | T | T | C | C | G | T | T | C | G | G | T | G | C | G | T | T | G | C | **A** | **G** | A | G | A | G | A | C | A | G | A | T | T | T | C | G | A | T | T | A | C | A | T | **G** | **T** |
| --- | --- | --- | --- | --- | --- | --- | --- | --- | --- | --- | --- | --- | --- | --- | --- | --- | --- | --- | --- | --- | --- | --- | --- | --- | --- | --- | --- | --- | --- | --- | --- | --- | --- | --- | --- | --- | --- | --- | --- | --- | --- | --- | --- | --- | --- | --- | --- | --- | --- | --- | --- | --- | --- | --- | --- | --- | --- | --- | --- | --- |
| Gene_G3H12 | T | C | A | A | T | G | A | T | A | A | T | T | T | T | G | G | T | T | T | G | C | G | T | T | C | G | G | T | A | C | G | T | T | G | C | **A** | **G** | A | G | G | A | A | T | A | G | A | T | C | C | C | G | A | T | T | A | C | G | A | **G** | **T** |

| Gene_G2D9 | A | A | G | T | T | C | A | A | T | A | A | T | G | A | C | G | A | C | C | A | T | A | A | T | T | T | A | G | G | C | C | A | T | T | T | T | A | A | A | A | A | C | T | C | A | T | T | C | T | T | T | T | T | T | A | A | T | T | C | T |
| --- | --- | --- | --- | --- | --- | --- | --- | --- | --- | --- | --- | --- | --- | --- | --- | --- | --- | --- | --- | --- | --- | --- | --- | --- | --- | --- | --- | --- | --- | --- | --- | --- | --- | --- | --- | --- | --- | --- | --- | --- | --- | --- | --- | --- | --- | --- | --- | --- | --- | --- | --- | --- | --- | --- | --- | --- | --- | --- | --- | --- |
| Gene_G3H12 | A | T | G | T | T | C | A | A | T | A | A | T | G | A | T | G | A | T | C | A | T | A | A | T | T | T | A | G | G | C | C | A | T | T | T | T | A | A | A | T | A | C | T | C | A | T | A | C | T | T | T | T | T | T | A | A | T | T | C | T |

**┤ ├ Intron 41**

| Gene_G2D9 | T | T | T | T | A | T | C | **A** | **G** | A | A | T | A | G | C | G | G | A | G | A | A | G | A | T | T | T | G | G | G | A | C | G | C | A | T | C | A | C | A | T | G | A | **G** | **T** | G | A | G | T | A | T | T | A | T | T | T | T | C | A | G | T |
| --- | --- | --- | --- | --- | --- | --- | --- | --- | --- | --- | --- | --- | --- | --- | --- | --- | --- | --- | --- | --- | --- | --- | --- | --- | --- | --- | --- | --- | --- | --- | --- | --- | --- | --- | --- | --- | --- | --- | --- | --- | --- | --- | --- | --- | --- | --- | --- | --- | --- | --- | --- | --- | --- | --- | --- | --- | --- | --- | --- | --- |
| Gene_G3H12 | T | T | T | T | A | T | C | **A** | **G** | A | A | T | G | G | C | A | G | A | G | A | A | G | A | T | C | T | G | G | G | A | C | G | C | G | C | C | A | C | T | T | T | A | **G** | **T** | G | T | G | T | A | T | T | T | T | T | C | T | C | A | A | A |

**┤**

| Gene_G2D9 | T | T | T | A | G | G | C | T | A | A | A | T | G | A | C | G | T | C | A | A | A | A | A | T | T | G | A | A | G | T | T | T | T | C | T | T | A | C | A | T | T | C | T | T | T | A | T | T | T | T | A | T | **A** | **G** | C | C | G | T | C | T |
| --- | --- | --- | --- | --- | --- | --- | --- | --- | --- | --- | --- | --- | --- | --- | --- | --- | --- | --- | --- | --- | --- | --- | --- | --- | --- | --- | --- | --- | --- | --- | --- | --- | --- | --- | --- | --- | --- | --- | --- | --- | --- | --- | --- | --- | --- | --- | --- | --- | --- | --- | --- | --- | --- | --- | --- | --- | --- | --- | --- | --- |
| Gene_G3H12 | T | T | A | A | G | A | T | T | A | A | A | T | G | A | C | G | T | C | A | A | A | A | A | T | T | G | A | A | G | T | T | T | T | C | T | T | A | C | A | T | T | C | T | T | T | A | T | T | T | T | A | T | **A** | **G** | C | A | A | G | C | T |

| Gene_G2D9 | T | A | A | A | G | A | T | G | C | A | T | T | G | A | G | G | G | A | A | G | C | A | T | C | T | A | G | C | C | G | T | G | G | A | A | T | T | G | G | A | A | T | A | T | C | C | A | A | C | **T** | **G** | **A** | A | A | T | T | T | T | C | A |
| --- | --- | --- | --- | --- | --- | --- | --- | --- | --- | --- | --- | --- | --- | --- | --- | --- | --- | --- | --- | --- | --- | --- | --- | --- | --- | --- | --- | --- | --- | --- | --- | --- | --- | --- | --- | --- | --- | --- | --- | --- | --- | --- | --- | --- | --- | --- | --- | --- | --- | --- | --- | --- | --- | --- | --- | --- | --- | --- | --- | --- |
| Gene_G3H12 | T | C | A | A | A | A | T | G | C | A | T | T | T | A | T | C | A | C | A | G | C | A | C | - | - | - | - | - | - | - | - | - | - | - | - | - | - | - | - | - | - | - | A | T | C | A | A | G | C | T | G | A | A | C | T | A | T | T | A | A |

**├ 3’-UTR**

| Gene_G2D9 | A | T | T | T | A | A | C | A | T | T | C | A | C | A | A | A | T | G | A | A | T | C | A | C | T | A | A | C | A | T | T | C | A | A | T | G | T | A | A | T | A | A | T | T | T | T | T | T | T | T | G | C | G | A | A | A | A | T | A | T |
| --- | --- | --- | --- | --- | --- | --- | --- | --- | --- | --- | --- | --- | --- | --- | --- | --- | --- | --- | --- | --- | --- | --- | --- | --- | --- | --- | --- | --- | --- | --- | --- | --- | --- | --- | --- | --- | --- | --- | --- | --- | --- | --- | --- | --- | --- | --- | --- | --- | --- | --- | --- | --- | --- | --- | --- | --- | --- | --- | --- | --- |
| Gene_G3H12 | A | T | **T** | **A** | **A** | A | T | C | T | T | C | A | C | A | A | A | T | G | T | A | T | C | A | C | T | A | A | C | A | T | T | C | A | A | T | G | T | A | A | T | A | A | T | T | T | - | - | - | T | T | G | G | G | A | A | A | A | T | A | T |

| Gene_G2D9 | A | A | T | A | C | G | A | A | A | T | T | G | A | A | T | T | G | T | T | A | A | C | T | T | C | T | G | A | T | A | T | A | A | A | A | A | A | A | T | T | A | A | A | T | A | T | T | T | T | T | G | A | A | T | G | T | T | C | A | C |
| --- | --- | --- | --- | --- | --- | --- | --- | --- | --- | --- | --- | --- | --- | --- | --- | --- | --- | --- | --- | --- | --- | --- | --- | --- | --- | --- | --- | --- | --- | --- | --- | --- | --- | --- | --- | --- | --- | --- | --- | --- | --- | --- | --- | --- | --- | --- | --- | --- | --- | --- | --- | --- | --- | --- | --- | --- | --- | --- | --- | --- |
| Gene_G3H12 | A | A | T | A | C | G | A | A | A | T | T | G | A | A | T | T | G | T | C | T | A | C | T | T | C | T | G | A | T | A | T | A | A | A | G | A | A | A | T | T | A | A | A | T | A | T | T | T | T | T | G | A | A | T | G | T | G | C | A | C |

| Gene_G2D9 | A | T | A | T | A | G | A | A | T | T | T | A | A | T | C | T | T | T | G | T | T | T | T | A | A | T | G | T | C | T | A | T | A | A | A | A | T | T | A | C | G | T | T | T | T | T | A | T | C | A | A | A | A | C | T | T | T | T | G | C |
| --- | --- | --- | --- | --- | --- | --- | --- | --- | --- | --- | --- | --- | --- | --- | --- | --- | --- | --- | --- | --- | --- | --- | --- | --- | --- | --- | --- | --- | --- | --- | --- | --- | --- | --- | --- | --- | --- | --- | --- | --- | --- | --- | --- | --- | --- | --- | --- | --- | --- | --- | --- | --- | --- | --- | --- | --- | --- | --- | --- | --- |
| Gene_G3H12 | A | C | A | T | C | G | A | A | T | C | A | A | A | T | T | T | T | T | G | T | T | T | C | A | A | T | G | T | C | T | A | T | A | G | A | A | A | - | A | C | - | - | - | - | - | - | - | T | C | A | A | A | A | C | G | T | T | T | G | C |

| Gene_G2D9 | G | A | C | A | A | A | A | C | G | C | A | A | A | A | A | A | A | A | A | A | T | C | A | T | G | C | A | A | A | T | G | T | T | C | A | T | T | G | T | T | C | A | T | T | T | A | A | T | C | G | G | A | A | A | C | G | T | G | A | A |
| --- | --- | --- | --- | --- | --- | --- | --- | --- | --- | --- | --- | --- | --- | --- | --- | --- | --- | --- | --- | --- | --- | --- | --- | --- | --- | --- | --- | --- | --- | --- | --- | --- | --- | --- | --- | --- | --- | --- | --- | --- | --- | --- | --- | --- | --- | --- | --- | --- | --- | --- | --- | --- | --- | --- | --- | --- | --- | --- | --- | --- |
| Gene_G3H12 | G | A | C | A | A | A | T | C | G | T | A | T | A | A | A | A | A | - | - | A | T | C | A | T | G | C | A | A | A | T | G | T | T | C | A | T | T | G | T | T | T | A | T | T | T | C | A | T | C | A | G | T | A | A | C | A | C | G | A | A |

| Gene_G2D9 | A | C | C | A | T | A | G | A | A | A | C | C | A | C | A | C | T | T | T | T | A | T | C | G | A | C | T | C | T | T | T | T | T | A | G | G | T | T | T | G | A | G | T | T | T | G | A | G | T | A | C | T | T | T | A | A | A | G | C | T |
| --- | --- | --- | --- | --- | --- | --- | --- | --- | --- | --- | --- | --- | --- | --- | --- | --- | --- | --- | --- | --- | --- | --- | --- | --- | --- | --- | --- | --- | --- | --- | --- | --- | --- | --- | --- | --- | --- | --- | --- | --- | --- | --- | --- | --- | --- | --- | --- | --- | --- | --- | --- | --- | --- | --- | --- | --- | --- | --- | --- | --- |
| Gene_G3H12 | A | C | C | A | T | A | G | A | A | A | C | C | A | C | A | C | T | T | T | C | A | T | C | G | A | C | T | C | T | C | T | T | T | C | G | A | T | T | T | G | T | G | T | T | T | G | A | G | - | - | - | - | - | - | - | - | - | - | - | - |

| Gene_G2D9 | T | A | A | G | T | T | T | C | A | G | T | T | T | C | T | T | A | T | C | T | A | A | C | A | A | A | A | T | T | T | G | A | T | G | T | T | T | G | A | - | - | - | - | T | T | T | T | T | G | A | A | T | T | A | G | C | G | T | T | C |
| --- | --- | --- | --- | --- | --- | --- | --- | --- | --- | --- | --- | --- | --- | --- | --- | --- | --- | --- | --- | --- | --- | --- | --- | --- | --- | --- | --- | --- | --- | --- | --- | --- | --- | --- | --- | --- | --- | --- | --- | --- | --- | --- | --- | --- | --- | --- | --- | --- | --- | --- | --- | --- | --- | --- | --- | --- | --- | --- | --- | --- |
| Gene_G3H12 | - | - | - | G | A | T | T | C | A | G | T | T | T | C | T | T | A | T | C | T | A | A | C | A | A | A | A | T | T | T | G | A | G | G | T | T | T | G | A | A | T | T | G | T | T | T | G | T | G | A | C | T | C | A | G | C | A | A | T | C |

| Gene_G2D9 | A | A | T | C | G | C | G | T | T | A | A | T | T | T | T | C | G | C | C | T | G | A | A | A | C | A | G | C | T | T | T | G | T | G | A | A | T | T | G | A | T | C | G | G | C | C | A | A | T | A | T | T | G | A | A | A | A | A | T | G |
| --- | --- | --- | --- | --- | --- | --- | --- | --- | --- | --- | --- | --- | --- | --- | --- | --- | --- | --- | --- | --- | --- | --- | --- | --- | --- | --- | --- | --- | --- | --- | --- | --- | --- | --- | --- | --- | --- | --- | --- | --- | --- | --- | --- | --- | --- | --- | --- | --- | --- | --- | --- | --- | --- | --- | --- | --- | --- | --- | --- | --- |
| Gene_G3H12 | A | A | T | T | G | C | G | T | T | A | A | T | T | T | T | C | G | C | T | T | G | T | A | A | C | A | G | C | T | T | T | G | T | G | A | A | T | C | G | A | T | C | G | G | T | C | A | A | T | A | T | T | G | G | A | A | T | A | T | T |

| Gene_G2D9 | A | T | G | T | - | C | G | A | T | T | G | T | A | T | C | G | A | A | T | G | G | T | T | T | C | G | T | C | G | C | C | T | T | T | T | T | G | A | A | A | T | G | G | T | A | A | A | T | G | T | C | A | G | T | G | A | A | C | A | A |
| --- | --- | --- | --- | --- | --- | --- | --- | --- | --- | --- | --- | --- | --- | --- | --- | --- | --- | --- | --- | --- | --- | --- | --- | --- | --- | --- | --- | --- | --- | --- | --- | --- | --- | --- | --- | --- | --- | --- | --- | --- | --- | --- | --- | --- | --- | --- | --- | --- | --- | --- | --- | --- | --- | --- | --- | --- | --- | --- | --- | --- |
| Gene_G3H12 | A | A | A | T | T | C | G | A | T | G | C | C | A | T | C | T | T | T | T | T | G | A | A | T | T | G | G | T | T | C | T | T | T | C | T | T | G | G | A | A | - | - | G | T | T | G | A | A | A | A | T | A | A | T | G | A | A | T | C | A |

| Gene_G2D9 | G | A | C | A | C | T | T | G | A | C | A | C | T | G | T | G | A | G | C | T | A | C | C | A | G | A | A | A | T | C | A | - | - | - | G | G | C | A | C | T | T | C | G | A | T | G | C | T | T | A | A | T | T | - | T | T | A | C | C | C |
| --- | --- | --- | --- | --- | --- | --- | --- | --- | --- | --- | --- | --- | --- | --- | --- | --- | --- | --- | --- | --- | --- | --- | --- | --- | --- | --- | --- | --- | --- | --- | --- | --- | --- | --- | --- | --- | --- | --- | --- | --- | --- | --- | --- | --- | --- | --- | --- | --- | --- | --- | --- | --- | --- | --- | --- | --- | --- | --- | --- | --- |
| Gene_G3H12 | A | A | C | G | A | T | A | C | C | G | A | T | T | G | A | A | T | G | A | C | - | T | C | A | G | A | A | A | T | C | A | A | A | T | G | G | C | C | C | T | T | T | C | A | A | G | A | T | T | G | G | T | A | A | T | T | A | A | A | A |

| Gene_G2D9 | G | G | A | A | T | A | C | G | A | G | G | C | C | T | G | A | A | T | T | G | C | A | T | A | A | T | T | T | G | T | G | T | T | A | T | C | T | G | T | G | A | T | A | T | A | T | T | T | T | A | T | A | A | T | A | T | T | A | T | T |
| --- | --- | --- | --- | --- | --- | --- | --- | --- | --- | --- | --- | --- | --- | --- | --- | --- | --- | --- | --- | --- | --- | --- | --- | --- | --- | --- | --- | --- | --- | --- | --- | --- | --- | --- | --- | --- | --- | --- | --- | --- | --- | --- | --- | --- | --- | --- | --- | --- | --- | --- | --- | --- | --- | --- | --- | --- | --- | --- | --- | --- |
| Gene_G3H12 | G | T | G | T | A | A | T | T | C | T | T | C | T | C | A | A | A | C | T | T | C | A | G | A | A | T | A | T | A | T | G | T | C | A | A | T | C | G | C | G | A | A | T | G | A | T | T | T | C | A | A | A | - | - | - | T | T | T | T | C |

| Gene_G2D9 | T | C | A | G | T | A | T | T | C | G | T | A | G | T | T | G | T | T | T | A | G | G | A | C | C | A | T | C | A | T | A | C | G | T | A | A | T | G | A | T | G | C | C | A | A | C | T | G | T | A | C | T | G | G | A | T | T | T | T | C |
| --- | --- | --- | --- | --- | --- | --- | --- | --- | --- | --- | --- | --- | --- | --- | --- | --- | --- | --- | --- | --- | --- | --- | --- | --- | --- | --- | --- | --- | --- | --- | --- | --- | --- | --- | --- | --- | --- | --- | --- | --- | --- | --- | --- | --- | --- | --- | --- | --- | --- | --- | --- | --- | --- | --- | --- | --- | --- | --- | --- | --- |
| Gene_G3H12 | T | C | A | A | T | C | C | T | C | T | G | A | C | C | G | A | A | T | T | T | T | G | - | - | T | A | G | T | G | T | A | A | C | T | A | G | A | G | A | A | A | T | C | C | G | C | C | - | T | A | T | A | G | - | - | - | - | - | - | C |

| Gene_G2D9 | C | A | G | T | A | T | C | A | T | A | C | T | G | A | A | T | T | G | T | A | C | C | C | C | A | T | A | C | T | G | G | A | T | T | T | G | A | T | A | A | T | G | G | A | T | T | T | T | G | A | T | G | A | A | A | T | T | T | T | C |
| --- | --- | --- | --- | --- | --- | --- | --- | --- | --- | --- | --- | --- | --- | --- | --- | --- | --- | --- | --- | --- | --- | --- | --- | --- | --- | --- | --- | --- | --- | --- | --- | --- | --- | --- | --- | --- | --- | --- | --- | --- | --- | --- | --- | --- | --- | --- | --- | --- | --- | --- | --- | --- | --- | --- | --- | --- | --- | --- | --- | --- |
| Gene_G3H12 | T | A | G | G | C | A | A | A | A | A | A | A | G | G | G | G | C | C | T | T | T | T | G | G | A | T | A | C | T | G | A | C | T | A | T | A | A | T | A | A | T | G | A | G | A | T | C | - | G | T | T | A | G | C | A | T | T | C | G | A |

| Gene_G2D9 | T | T | A | A | A | A | A | T | T | C | A | A | A | T | T | T | T | T | A | C | A | A | T | C | A | A | A | A | T | G | A | A | A | A | A | T | A | A | C | T | C | A | A | A | A | T | A | C | T | T | T | T | A | A | T | C | C | A | G | G |
| --- | --- | --- | --- | --- | --- | --- | --- | --- | --- | --- | --- | --- | --- | --- | --- | --- | --- | --- | --- | --- | --- | --- | --- | --- | --- | --- | --- | --- | --- | --- | --- | --- | --- | --- | --- | --- | --- | --- | --- | --- | --- | --- | --- | --- | --- | --- | --- | --- | --- | --- | --- | --- | --- | --- | --- | --- | --- | --- | --- | --- |
| Gene_G3H12 | C | T | A | A | A | C | G | C | T | C | - | - | - | - | T | T | G | T | G | C | G | - | - | - | - | - | - | T | T | G | G | G | C | A | T | T | T | G | C | T | A | T | T | G | G | G | A | T | T | G | T | T | A | G | C | C | C | A | G | T |

| Gene_G2D9 | A | A | A | A | A | A | T | C | T | G | T | T | T | C | T | T | A | T | G | A | G | C | G | - | - | - | - | - | - | - | T | T | T | T | A | G | A | A | T | G | G | T | A | T | G | T | G | T | T | T | T | C | G | T | T | A | A | C | G | T |
| --- | --- | --- | --- | --- | --- | --- | --- | --- | --- | --- | --- | --- | --- | --- | --- | --- | --- | --- | --- | --- | --- | --- | --- | --- | --- | --- | --- | --- | --- | --- | --- | --- | --- | --- | --- | --- | --- | --- | --- | --- | --- | --- | --- | --- | --- | --- | --- | --- | --- | --- | --- | --- | --- | --- | --- | --- | --- | --- | --- | --- |
| Gene_G3H12 | A | A | T | G | G | A | T | A | A | G | C | T | C | G | T | T | A | T | A | A | G | C | G | G | T | G | A | G | G | T | T | T | T | A | A | A | A | A | T | G | G | A | C | G | G | T | A | C | T | T | T | C | A | T | C | C | A | A | A | T |

| Gene_G2D9 | T | C | T | C | T | C | T | G | A | A | A | A | A | T | G | A | T | C | C | T | A | C | G | T | G | T | T | C | C | A | A | T | C | G | A | G | T | T | G | C | T | T | G | T | T | T | T | T | G | A | T | C | T | T | G | G | T | G | T | G |
| --- | --- | --- | --- | --- | --- | --- | --- | --- | --- | --- | --- | --- | --- | --- | --- | --- | --- | --- | --- | --- | --- | --- | --- | --- | --- | --- | --- | --- | --- | --- | --- | --- | --- | --- | --- | --- | --- | --- | --- | --- | --- | --- | --- | --- | --- | --- | --- | --- | --- | --- | --- | --- | --- | --- | --- | --- | --- | --- | --- | --- |
| Gene_G3H12 | T | - | - | - | - | - | - | - | A | G | A | A | A | C | A | C | T | C | G | A | T | C | A | T | - | T | T | G | T | A | A | T | G | G | G | C | C | C | A | T | G | C | A | T | A | T | G | G | A | A | T | T | T | C | T | A | A | G | C | A |

| Gene_G2D9 | C | G | A | G | A | C | A | A | T | A | A | T | T | T | A | G | G | A | A | T | A | C | A | A | T | G | A | A | A | C | C | A | A | C | T | A | A | C | A | G | G | A | T | T | C | G | C | T | T | C | G | A | G | G | A | T | A | C | A | T |
| --- | --- | --- | --- | --- | --- | --- | --- | --- | --- | --- | --- | --- | --- | --- | --- | --- | --- | --- | --- | --- | --- | --- | --- | --- | --- | --- | --- | --- | --- | --- | --- | --- | --- | --- | --- | --- | --- | --- | --- | --- | --- | --- | --- | --- | --- | --- | --- | --- | --- | --- | --- | --- | --- | --- | --- | --- | --- | --- | --- | --- |
| Gene_G3H12 | T | G | A | - | - | - | - | A | T | C | A | T | T | C | A | T | C | G | T | T | T | C | G | C | T | T | G | A | A | G | A | T | A | A | A | A | A | A | A | A | A | G | T | C | A | A | A | T | T | C | A | A | C | G | C | T | G | T | C | A |

| Gene_G2D9 | A | T | G | T | A | T | T | T | G | A | T | G | G | A | A | G | T | T | T | C | C | T | T | T | T | C | G | A | A | T | A | A | T | C | T | T | C | T | T | T | T | G | G | A | A | T | T | G | T | A | T | T | T | G | A | A | T | T | A | C |
| --- | --- | --- | --- | --- | --- | --- | --- | --- | --- | --- | --- | --- | --- | --- | --- | --- | --- | --- | --- | --- | --- | --- | --- | --- | --- | --- | --- | --- | --- | --- | --- | --- | --- | --- | --- | --- | --- | --- | --- | --- | --- | --- | --- | --- | --- | --- | --- | --- | --- | --- | --- | --- | --- | --- | --- | --- | --- | --- | --- | --- |
| Gene_G3H12 | C | T | G | A | G | A | T | T | T | T | T | C | T | A | T | G | A | G | A | T | T | T | T | C | T | C | A | A | A | A | T | T | T | G | G | T | T | T | G | T | T | A | A | A | T | A | G | G | G | T | C | C | T | G | G | T | T | G | G | C |

| Gene_G2D9 | A | T | G | A | A | A | T | G | T | A | A | T | T | T | G | T | C | T | G | G | C | A | A | G | T | T | T | T | G | A | A | A | T | G | G | A | T | A | A | T | T | T | C | C | A | A | C | T | C | C | A | A | T | T | C | C | A | A | G | G |
| --- | --- | --- | --- | --- | --- | --- | --- | --- | --- | --- | --- | --- | --- | --- | --- | --- | --- | --- | --- | --- | --- | --- | --- | --- | --- | --- | --- | --- | --- | --- | --- | --- | --- | --- | --- | --- | --- | --- | --- | --- | --- | --- | --- | --- | --- | --- | --- | --- | --- | --- | --- | --- | --- | --- | --- | --- | --- | --- | --- | --- |
| Gene_G3H12 | A | T | C | A | G | A | - | - | - | - | - | - | - | - | G | C | T | G | G | G | C | A | C | A | G | C | A | C | T | T | A | A | T | G | C | G | A | A | A | T | T | T | T | G | G | A | T | T | C | - | A | G | C | T | C | T | G | A | A | A |

| Gene_G2D9 | C | T | A | C | G | T | T | T | C | A | A | T | A | A | C | G | A | T | T | T | T | T | C | A | T | A | A | T | A | A | A | G | A | C | T | A | A | A | G | A | C | C | G | A | G | T | T | A | T | T | T | A | T | A | A | A | T | A | T | C |
| --- | --- | --- | --- | --- | --- | --- | --- | --- | --- | --- | --- | --- | --- | --- | --- | --- | --- | --- | --- | --- | --- | --- | --- | --- | --- | --- | --- | --- | --- | --- | --- | --- | --- | --- | --- | --- | --- | --- | --- | --- | --- | --- | --- | --- | --- | --- | --- | --- | --- | --- | --- | --- | --- | --- | --- | --- | --- | --- | --- | --- |
| Gene_G3H12 | T | T | C | G | A | A | G | C | G | A | A | T | A | A | A | G | - | - | - | - | - | - | - | - | - | - | - | - | - | - | - | - | - | - | - | - | - | - | - | - | - | - | - | - | - | - | - | - | - | - | - | - | - | - | - | - | - | - | - | - |

**Protein alignment:**

Intron/exon boundaries

**▼ ▼ ▼ ▼ ▼ ▼ ▼ ▼ ▼**

| G2D9 | ***M*** | ***K*** | ***F*** | ***T*** | ***L*** | ***G*** | ***F*** | ***L*** | ***I*** | ***V*** | ***A*** | ***A*** | ***T*** | ***A*** | ***I*** | ***L*** | ***V*** | ***A*** | ***S*** | D | A | P | T | M | S | E | L | F | Q | E | R | L | K | L | V | T | E | F | T | V | L | M | T | K | L | D | A | N | A | M | Q | R | A | A | G | N | C | P | Q | L |
| --- | --- | --- | --- | --- | --- | --- | --- | --- | --- | --- | --- | --- | --- | --- | --- | --- | --- | --- | --- | --- | --- | --- | --- | --- | --- | --- | --- | --- | --- | --- | --- | --- | --- | --- | --- | --- | --- | --- | --- | --- | --- | --- | --- | --- | --- | --- | --- | --- | --- | --- | --- | --- | --- | --- | --- | --- | --- | --- | --- | --- |
| G3H12 | ***M*** | ***K*** | ***Y*** | ***S*** | ***L*** | ***A*** | ***F*** | ***L*** | ***F*** | ***V*** | ***A*** | ***S*** | ***T*** | ***A*** | ***F*** | ***L*** | ***V*** | ***A*** | ***S*** | A | P | P | T | S | - | - | - | - | V | D | R | A | R | T | V | A | R | L | N | Q | L | L | D | R | V | E | R | N | V | A | Q | R | L | R | - | - | - | - | - | - |

**▼ ▼ ▼ ▼ ▼ ▼ ▼ ▼ ▼ ▼ ▼ ▼ ▼**

| G2D9 | D | P | N | A | M | H | R | Y | E | S | R | R | N | I | L | L | N | E | I | Q | F | Y | E | S | Q | R | A | E | L | L | D | E | A | R | S | A | Q | G | T | P | H | F | Y | M | R | L | V | P | K | L | V | R | N | A | C | D | L | F | Q | L |
| --- | --- | --- | --- | --- | --- | --- | --- | --- | --- | --- | --- | --- | --- | --- | --- | --- | --- | --- | --- | --- | --- | --- | --- | --- | --- | --- | --- | --- | --- | --- | --- | --- | --- | --- | --- | --- | --- | --- | --- | --- | --- | --- | --- | --- | --- | --- | --- | --- | --- | --- | --- | --- | --- | --- | --- | --- | --- | --- | --- | --- |
| G3H12 | - | - | - | - | L | G | S | N | A | A | R | N | N | Q | L | I | N | A | A | Q | N | P | N | L | N | - | - | - | - | - | - | - | - | - | - | - | - | - | H | R | V | F | V | N | E | I | L | P | E | L | Q | R | - | - | - | - | - | - | - | - |

**▼ ▼ ▼ ▼ ▼ ▼ ▼ ▼ ▼ ▼ ▼ ▼ ▼**

| G2D9 | R | D | I | R | A | K | H | F | E | T | Q | S | P | A | A | Q | S | V | E | C | D | L | S | E | L | F | F | V | L | A | R | N | A | A | Q | R | R | A | I | L | A | S | R | E | L | T | R | Q | H | K | T | D | L | G | L | Y | T | T | P | R |
| --- | --- | --- | --- | --- | --- | --- | --- | --- | --- | --- | --- | --- | --- | --- | --- | --- | --- | --- | --- | --- | --- | --- | --- | --- | --- | --- | --- | --- | --- | --- | --- | --- | --- | --- | --- | --- | --- | --- | --- | --- | --- | --- | --- | --- | --- | --- | --- | --- | --- | --- | --- | --- | --- | --- | --- | --- | --- | --- | --- | --- |
| G3H12 | - | - | L | L | L | E | R | A | Q | L | E | R | S | V | V | S | T | A | E | G | D | L | A | E | L | L | V | E | L | S | R | S | A | A | E | R | - | - | - | - | - | - | - | A | Q | L | K | I | Y | F | P | A | L | Y | T | R | T | P | K | D |

**▼ ▼ ▼ ▼ ▼ ▼**

| G2D9 | D | L | T | V | I | P | T | P | E | E | R | N | T | L | M | K | A | L | R | I | F | I | S | P | Q | K | N | D | F | K | R | I | L | M | D | T | S | V | C | K | E | T | D | F | D | Y | I | I | A | E | K | I | W | D | A | S | H | D | R | L |
| --- | --- | --- | --- | --- | --- | --- | --- | --- | --- | --- | --- | --- | --- | --- | --- | --- | --- | --- | --- | --- | --- | --- | --- | --- | --- | --- | --- | --- | --- | --- | --- | --- | --- | --- | --- | --- | --- | --- | --- | --- | --- | --- | --- | --- | --- | --- | --- | --- | --- | --- | --- | --- | --- | --- | --- | --- | --- | --- | --- | --- |
| G3H12 | I | L | I | F | L | D | S | R | V | E | L | S | A | L | K | D | V | L | R | I | F | I | G | P | R | K | G | D | F | K | R | I | L | I | N | C | G | I | C | E | G | I | D | P | D | Y | E | M | A | E | K | I | W | D | A | P | L | Y | K | L |

| G2D9 | K | D | A | L | R | E | A | S | S | R | G | I | G | I | S | Y |  |  |  |  |  |  |  |  |  |  |  |  |  |  |  |  |  |  |  |  |  |  |  |  |  |  |  |  |  |  |  |  |  |  |  |  |  |  |  |  |  |  |  |  |
| --- | --- | --- | --- | --- | --- | --- | --- | --- | --- | --- | --- | --- | --- | --- | --- | --- | --- | --- | --- | --- | --- | --- | --- | --- | --- | --- | --- | --- | --- | --- | --- | --- | --- | --- | --- | --- | --- | --- | --- | --- | --- | --- | --- | --- | --- | --- | --- | --- | --- | --- | --- | --- | --- | --- | --- | --- | --- | --- | --- | --- |
| G3H12 | Q | N | A | F | I | T | A | H | Q | A | E | L | L | N | - | - |  |  |  |  |  |  |  |  |  |  |  |  |  |  |  |  |  |  |  |  |  |  |  |  |  |  |  |  |  |  |  |  |  |  |  |  |  |  |  |  |  |  |  |  |

Figure S1. Nucleotide and predicted amino acid sequence alignments of genes from families *SSSGP-1* (**A**), *SSSGP-31* (**B**)*, SSSGP-3* (**C**)*, SSSGP-5* (**D**), and *SSSGP-2* (**E**). Putative TATA box, transcription starting site, ATG initiation codon, intron/exon splicing donor and receptor, translation stop codon, and the residue where the cDNA ends are in bold red font. Starting positions for 5’-UTR, SPCR, MPCR, intron, and 3’UTR are indicated above the nucleotide alignment. Putative secretion signal peptides in the amino acid alignments are in bold italic font. Intron/exon boundaries were also indicated in the amino acid alignment. Both nucleotide and amino acid sequence alignments were produced using ClusterW2 initially and were marked using boxshade (<http://mobyle.pasteur.fr/cgi-bin/portal.py?form=boxshade>). In panel **E**, full length sequence was obtained for the gene corresponding to the cDNA G3H12, but only partial sequence was obtained for the cDNA G2D9. The numbers of introns was determined by comparing the gene and cDNA sequences of G3H12. Full cDNA sequences for both G2D9 and G3H12 were given in panel **A** of Figure S2.

Sequence curation: To avoid sequence assembly errors of the BAC clone, primers were designed according to the assembled BAC sequence and used to amplify DNA fragments around 3 kB in size using the original BAC DNA as template. The amplified DNA fragments were sequenced directly. No assembly error was found in the assembled BAC sequences.
