# Supplementary material for: Unusual conservation among genes encoding small secreted salivary gland proteins from a gall midge
Source: BMC Evol Biol. 2010 Sep 28;10:296. doi: 10.1186/1471-2148-10-296 (PMC2955719; doi:10.1186/1471-2148-10-296)
Supplement: Additional file 2 — Figure S2: Sequence alignments of different groups of SSSGP-encoding cDNAs. [file 1471-2148-10-296-S2.DOC]

**A**

**├ 5’-UTR ├ SPCR**

| L5E4 | - | - | - | - | - | - | - | - | - | - | - | - | - | - | - | - | - | - | - | - | - | - | - | - | - | - | - | - | - | - | - | - | - | - | - | - | - | - | - | - | - | - | - | A | T | C | T | C | A | T | T | T | A | A | A | **A** | **T** | **G** | A | A |
| --- | --- | --- | --- | --- | --- | --- | --- | --- | --- | --- | --- | --- | --- | --- | --- | --- | --- | --- | --- | --- | --- | --- | --- | --- | --- | --- | --- | --- | --- | --- | --- | --- | --- | --- | --- | --- | --- | --- | --- | --- | --- | --- | --- | --- | --- | --- | --- | --- | --- | --- | --- | --- | --- | --- | --- | --- | --- | --- | --- | --- |
| W12F4 | - | - | - | - | - | - | - | - | - | - | - | - | - | - | - | - | - | - | - | - | A | A | C | T | T | T | G | T | T | C | C | A | G | A | A | A | A | G | A | A | - | - | T | A | A | C | T | C | A | T | T | C | A | A | A | **A** | **T** | **G** | A | A |
| G2D9 | - | - | - | - | - | - | - | - | - | - | - | - | - | - | - | - | - | - | - | - | A | A | C | T | T | T | G | T | T | C | A | A | G | A | A | A | A | A | A | A | A | A | C | A | A | C | T | C | A | T | T | T | A | A | A | **A** | **T** | **G** | A | A |
| G3H12 | - | - | - | - | - | - | - | - | - | - | - | - | - | - | - | - | - | - | - | - | - | A | C | T | T | T | G | T | T | T | C | A | G | A | A | A | A | A | A | C | A | - | - | A | T | C | - | C | A | T | T | T | A | A | A | **A** | **T** | **G** | A | A |
| W8E4 | - | - | - | - | - | - | - | - | - | - | - | - | - | - | - | - | - | - | - | - | A | A | C | T | T | T | G | T | T | C | A | A | G | A | A | A | A | A | A | T | A | - | - | A | A | C | T | C | A | T | T | T | A | A | A | **A** | **T** | **G** | A | A |
| G12G1 | - | - | - | - | - | - | - | - | - | - | - | T | T | T | T | G | A | A | T | C | A | A | C | T | T | T | G | T | T | C | C | A | G | A | A | A | A | A | A | A | A | A | A | A | - | C | T | C | A | T | T | T | A | A | A | **A** | **T** | **G** | A | A |
| S18E7 | - | - | - | - | - | - | - | - | - | - | - | T | T | C | T | G | A | A | T | C | A | A | T | T | T | T | G | T | T | C | C | A | G | A | A | A | A | G | A | A | A | A | A | - | - | C | T | C | A | T | T | C | A | A | A | **A** | **T** | **G** | A | A |
| G21F11 | - | - | - | - | - | - | - | - | - | - | - | T | T | T | T | G | A | A | T | C | A | A | C | T | T | T | G | T | T | C | C | A | T | A | G | A | T | A | A | A | A | A | C | T | A | A | T | - | - | - | T | T | A | A | A | **A** | **T** | **G** | A | A |
| G7F4 | - | - | - | - | - | - | - | - | - | - | - | T | T | T | T | G | A | C | T | C | A | A | C | T | T | C | G | T | T | C | C | A | G | A | A | A | A | A | A | A | A | A | A | G | A | A | T | C | A | T | T | T | A | A | A | **A** | **T** | **G** | A | A |
| G10H7 | - | - | - | - | - | - | - | - | - | - | - | T | T | T | T | G | A | A | T | C | A | A | T | T | T | T | G | T | T | C | A | A | G | A | A | A | A | A | A | A | - | - | - | G | A | C | T | A | A | T | T | T | G | A | A | **A** | **T** | **G** | A | A |
| W7F8 | - | - | - | - | - | - | - | - | - | - | - | - | - | - | - | - | - | - | - | - | - | - | C | T | T | T | G | T | T | T | C | A | G | A | A | A | A | A | A | A | A | A | - | - | - | C | T | A | A | T | T | T | A | A | A | **A** | **T** | **G** | A | A |
| W16A21 | G | A | A | T | T | T | A | T | C | A | G | T | T | C | T | G | A | A | T | C | A | A | C | T | T | T | G | C | T | C | C | A | G | A | A | A | A | A | A | A | A | A | - | - | - | C | T | C | A | T | T | T | A | A | A | **A** | **T** | **G** | A | A |
| W6C7 | - | - | - | - | - | - | - | - | - | - | - | - | - | - | - | - | - | - | - | - | A | A | C | T | T | T | G | T | T | C | C | A | G | A | A | A | A | A | A | A | A | A | A | - | - | C | T | A | A | T | T | T | A | A | A | **A** | **T** | **G** | A | A |
| G12H12 | - | - | - | - | - | - | - | - | - | - | - | - | - | - | - | - | - | - | - | - | - | - | - | - | - | - | - | - | - | - | - | - | - | - | - | - | A | A | A | C | A | A | A | - | - | C | T | A | A | A | T | T | A | A | A | **A** | **T** | **G** | A | A |
| W1C2 | - | - | - | - | - | - | - | - | - | - | - | - | - | - | - | - | - | - | - | - | - | - | - | - | - | - | - | - | - | - | - | - | - | - | - | - | - | - | - | - | - | - | - | - | - | - | - | - | - | A | T | T | A | A | A | **A** | **T** | **G** | A | A |

**├ MPCR**

| L5E4 | G | T | T | C | T | T | G | T | T | A | G | C | T | T | T | C | C | T | T | T | T | T | G | T | A | G | T | T | T | C | C | A | C | A | G | C | T | A | T | T | T | T | G | G | T | G | G | A | C | A | G | C | G | C | T | - | G | G | C | G |
| --- | --- | --- | --- | --- | --- | --- | --- | --- | --- | --- | --- | --- | --- | --- | --- | --- | --- | --- | --- | --- | --- | --- | --- | --- | --- | --- | --- | --- | --- | --- | --- | --- | --- | --- | --- | --- | --- | --- | --- | --- | --- | --- | --- | --- | --- | --- | --- | --- | --- | --- | --- | --- | --- | --- | --- | --- | --- | --- | --- | --- |
| W12F4 | G | T | T | T | T | T | G | T | T | A | G | C | T | T | T | C | C | T | T | T | T | T | G | T | A | G | C | T | T | C | C | A | T | A | G | C | T | A | T | T | T | T | G | G | T | G | G | C | C | A | G | C | G | G | T | C | G | A | C | C |
| G2D9 | G | T | T | C | A | C | G | T | T | A | G | G | T | T | T | C | C | T | T | A | T | C | G | T | A | G | C | T | G | C | C | A | C | A | G | C | T | A | T | T | T | T | A | G | T | T | G | C | C | A | G | C | G | A | T | G | C | A | C | C |
| G3H12 | G | T | A | C | T | C | G | T | T | A | G | C | T | T | T | C | C | T | T | T | T | C | G | T | A | G | C | T | T | C | C | A | C | A | G | C | T | T | T | T | T | T | G | G | T | G | G | C | C | A | G | C | G | C | T | C | C | A | C | C |
| W8E4 | G | T | A | C | T | C | G | T | T | A | G | C | T | T | T | C | C | T | T | T | T | C | G | T | A | G | C | T | G | C | C | A | C | A | G | C | T | A | T | T | T | T | G | G | C | G | G | C | C | A | G | C | G | C | T | T | T | A | C | C |
| G12G1 | G | T | A | C | T | C | G | T | T | A | G | C | T | T | T | C | C | T | T | G | T | C | G | T | A | G | C | T | T | C | C | A | C | A | G | C | T | A | T | T | T | T | G | G | T | G | G | C | C | A | G | C | A | T | G | A | A | T | C | C |
| S18E7 | G | T | T | C | T | T | G | T | T | T | G | C | T | T | T | C | G | T | T | T | T | T | G | T | A | G | C | T | T | C | C | A | C | A | G | T | T | T | T | T | T | T | G | G | T | G | G | A | C | A | G | C | A | C | T | G | G | A | C | C |
| G21F11 | G | T | A | C | T | C | A | T | T | A | G | C | T | T | T | C | C | T | T | T | T | C | G | T | A | G | C | T | G | C | C | A | C | A | T | T | T | A | T | T | T | T | G | G | T | G | G | A | C | A | G | T | G | G | C | A | C | C | A | T |
| G7F4 | G | T | A | C | T | C | G | T | T | A | G | C | T | T | T | C | C | T | T | T | T | G | G | C | A | G | C | T | G | C | C | A | C | A | G | T | T | A | T | T | T | C | G | G | T | G | G | C | C | A | G | C | G | C | T | G | T | A | G | G |
| G10H7 | G | T | A | C | T | C | G | T | T | A | G | C | T | T | T | C | C | T | T | T | T | C | G | T | A | G | C | T | G | C | C | A | C | A | G | C | T | A | T | T | T | T | G | G | T | G | G | C | C | A | G | C | G | T | G | G | A | A | G | C |
| W7F8 | G | T | A | C | T | T | G | T | T | A | G | C | T | T | T | C | C | T | T | A | T | T | G | T | A | G | C | T | T | C | C | A | C | A | G | C | T | A | T | T | T | T | G | G | T | G | G | C | C | A | G | T | A | C | T | G | G | A | G | G |
| W16A21 | G | T | G | C | T | T | G | T | T | A | A | C | T | T | T | C | C | T | T | T | T | C | G | T | A | G | C | T | T | C | C | A | C | A | G | C | T | A | T | T | T | T | G | G | T | G | G | C | C | A | G | T | G | C | T | C | C | A | C | C |
| W6C7 | G | T | A | C | T | C | G | T | T | T | G | C | T | T | T | C | C | T | T | T | T | C | G | T | A | G | C | T | G | C | C | A | C | A | G | C | T | A | T | T | T | C | G | A | T | G | G | C | C | A | G | C | G | C | T | G | C | A | G | G |
| G12H12 | G | T | A | T | T | C | G | T | T | A | G | C | T | T | T | C | C | T | T | T | T | C | G | T | A | G | C | T | T | C | T | A | C | A | G | C | T | T | T | T | T | T | G | A | T | G | G | C | C | A | G | C | G | C | T | G | C | A | C | C |
| W1C2 | G | T | A | T | T | C | G | T | T | A | G | C | T | T | T | C | C | T | T | T | T | C | G | T | A | G | C | T | T | C | T | A | C | A | G | C | T | T | T | T | T | T | G | A | T | G | G | C | C | A | G | C | G | C | T | G | C | A | C | C |

| L5E4 | A | T | C | A | A | A | A | C | A | C | - | - | - | - | - | T | G | A | C | G | C | T | G | A | A | C | T | G | A | T | A | C | A | G | G | C | T | G | A | A | C | T | G | A | T | A | C | A | A | C | G | T | G | A | A | C | A | G | C | A |
| --- | --- | --- | --- | --- | --- | --- | --- | --- | --- | --- | --- | --- | --- | --- | --- | --- | --- | --- | --- | --- | --- | --- | --- | --- | --- | --- | --- | --- | --- | --- | --- | --- | --- | --- | --- | --- | --- | --- | --- | --- | --- | --- | --- | --- | --- | --- | --- | --- | --- | --- | --- | --- | --- | --- | --- | --- | --- | --- | --- | --- |
| W12F4 | G | T | C | G | A | A | A | C | G | - | - | - | - | - | - | - | - | - | - | - | - | T | G | A | A | C | A | G | C | A | A | C | T | A | C | G | T | G | A | A | C | A | G | C | A | A | C | T | A | C | G | T | G | A | A | G | A | A | C | A |
| G2D9 | G | A | C | A | A | T | G | T | C | - | - | - | - | - | - | - | - | - | - | - | - | T | G | A | A | T | T | A | T | T | C | C | A | G | - | - | - | G | A | A | C | G | G | T | T | A | A | A | A | C | T | T | G | T | A | A | C | G | G | A |
| G3H12 | G | A | C | - | - | - | - | - | - | - | - | - | - | - | - | C | A | G | T | G | T | A | G | A | T | C | G | G | G | C | A | A | G | A | A | C | T | G | T | A | G | C | G | A | G | A | C | T | C | A | A | T | C | A | A | T | T | - | - | - |
| W8E4 | G | T | C | A | A | C | T | T | A | - | - | - | - | - | - | T | A | A | C | T | T | A | G | A | A | C | T | G | G | A | A | C | T | C | A | A | T | A | G | A | C | T | G | A | A | A | C | T | C | C | A | T | G | G | A | A | T | - | - | - |
| G12G1 | A | - | - | - | - | - | - | - | - | - | - | - | - | - | - | - | T | C | C | G | C | T | G | A | A | C | G | G | G | A | - | - | - | - | A | C | G | C | C | - | - | - | G | T | G | A | A | T | T | A | C | T | G | G | C | T | G | C | A | C |
| S18E7 | G | C | A | - | - | - | - | - | - | - | - | - | - | - | - | C | T | C | C | A | A | T | G | C | A | C | C | G | A | A | - | - | - | - | A | C | T | C | A | C | A | G | A | C | A | A | A | G | A | A | C | T | A | G | C | T | G | C | - | T |
| G21F11 | T | C | - | - | - | - | - | - | - | - | - | - | - | - | - | C | A | T | C | G | G | C | A | A | A | C | G | C | T | G | C | - | - | - | A | C | C | G | C | A | A | C | G | G | G | T | A | C | T | A | T | T | G | C | A | A | A | T | G | A |
| G7F4 | T | T | - | - | - | - | - | - | - | - | - | - | - | - | - | C | C | G | G | G | C | C | A | C | G | C | A | G | T | T | C | - | - | - | A | T | C | G | A | A | A | C | G | G | G | A | A | C | A | A | C | T | T | C | G | A | A | C | C | G |
| G10H7 | A | A | - | - | - | - | - | - | - | - | - | - | - | - | - | G | T | G | G | A | T | C | A | C | C | C | G | G | T | T | C | C | - | - | G | C | C | G | G | T | T | C | C | A | G | A | A | T | A | T | C | C | G | G | T | C | C | C | A | G |
| W7F8 | A | A | A | - | - | - | - | - | - | - | - | - | - | - | - | C | G | G | C | G | C | T | G | G | A | T | C | T | T | C | A | G | G | C | G | C | T | G | G | A | T | T | G | T | C | A | G | A | C | G | A | G | G | A | A | A | A | G | G | C |
| W16A21 | G | C | C | - | - | - | - | - | - | - | - | - | - | - | - | A | C | C | T | G | G | T | G | G | A | T | C | G | T | T | A | A | G | T | G | C | T | G | G | A | T | C | G | T | C | A | A | G | T | G | C | T | G | G | A | T | C | G | T | C |
| W6C7 | T | G | A | - | - | - | - | - | - | - | - | - | - | - | - | A | T | T | C | G | A | T | G | G | G | T | T | C | T | A | T | G | A | A | A | C | T | A | A | T | A | C | A | G | A | A | T | T | C | G | C | T | G | A | A | G | G | T | G | A |
| G12H12 | G | A | A | - | - | - | - | - | - | - | - | - | - | - | - | A | C | G | C | G | C | T | G | C | T | C | A | G | C | A | A | A | G | C | G | C | T | G | A | A | G | G | G | T | T | G | C | C | C | T | C | T | G | C | T | C | C | A | C | G |
| W1C2 | G | A | G | A | A | T | C | A | A | T | G | A | G | A | T | A | A | A | C | C | G | A | G | C | A | C | A | G | G | C | A | A | C | C | G | T | T | G | A | C | C | G | G | T | T | A | A | G | C | G | G | G | G | A | A | C | T | G | G | C |

| L5E4 | A | C | T | C | G | C | T | G | G | G | A | T | G | C | C | C | G | C | C | A | A | T | G | C | A | C | G | G | C | G | A | A | G | A | G | C | T | G | C | A | G | A | G | G | A | A | C | - | - | - | - | - | - | - | - | - | - | - | - | - |
| --- | --- | --- | --- | --- | --- | --- | --- | --- | --- | --- | --- | --- | --- | --- | --- | --- | --- | --- | --- | --- | --- | --- | --- | --- | --- | --- | --- | --- | --- | --- | --- | --- | --- | --- | --- | --- | --- | --- | --- | --- | --- | --- | --- | --- | --- | --- | --- | --- | --- | --- | --- | --- | --- | --- | --- | --- | --- | --- | --- | --- |
| W12F4 | A | C | T | C | G | C | T | G | A | A | G | A | G | C | - | - | A | A | C | T | A | C | G | T | A | - | - | - | - | - | - | - | - | - | - | - | - | - | - | - | - | - | - | - | - | - | - | - | - | - | - | - | - | - | - | - | - | - | - | - |
| G2D9 | A | T | T | C | A | C | T | G | T | A | T | T | A | A | T | G | A | C | T | A | A | A | - | - | - | - | - | - | - | - | - | - | - | - | - | - | - | - | - | - | - | - | - | - | - | - | - | - | C | T | G | G | A | C | G | C | C | A | A | T |
| G3H12 | A | C | T | C | G | A | T | A | G | A | G | T | G | G | A | A | C | G | C | A | A | T | G | T | A | G | C | G | C | - | - | - | - | - | - | - | - | - | - | - | - | - | - | - | - | - | - | - | - | - | - | - | - | - | - | - | - | - | - | - |
| W8E4 | A | C | T | C | T | C | T | A | G | A | C | T | G | G | A | A | C | T | C | C | - | - | - | - | - | - | - | - | - | - | - | - | - | - | - | - | - | - | - | - | - | - | - | - | - | - | - | - | - | - | - | - | - | - | - | - | - | - | - | - |
| G12G1 | T | G | A | A | T | A | G | G | G | C | T | G | G | A | G | G | A | A | A | T | C | C | C | G | C | T | G | G | - | - | - | - | - | - | - | - | - | - | - | - | - | - | - | - | - | - | - | - | - | - | - | - | - | - | - | - | - | - | - | - |
| S18E7 | T | A | T | C | T | T | G | C | G | T | T | A | A | A | A | G | A | A | A | C | A | - | - | - | - | - | - | - | - | - | - | - | - | - | - | - | - | - | - | - | - | - | - | - | - | - | - | - | - | - | - | - | - | - | - | - | - | - | - | - |
| G21F11 | G | T | C | A | T | G | A | A | A | T | T | C | G | C | G | A | C | G | C | G | A | T | A | T | G | - | - | - | - | - | - | - | - | - | - | - | - | - | - | - | - | - | - | - | - | - | - | - | - | - | - | - | - | - | - | - | - | - | - | - |
| G7F4 | C | T | G | A | A | C | G | G | G | A | T | C | G | C | A | A | A | C | - | - | - | - | - | - | - | - | - | - | - | - | - | - | - | - | - | - | - | - | - | - | - | - | - | - | - | - | - | - | - | - | - | - | - | - | - | - | - | - | - | - |
| G10H7 | G | G | G | A | T | C | G | G | G | T | T | C | - | C | G | G | G | G | G | A | T | C | A | - | - | - | - | - | - | - | - | - | - | - | - | - | - | - | - | - | - | - | - | - | - | - | - | - | - | - | - | - | - | - | - | - | - | - | - | - |
| W7F8 | T | G | A | A | - | A | A | G | A | T | G | A | T | T | G | C | T | A | C | A | C | T | G | A | A | T | G | C | - | - | - | - | - | - | - | - | - | - | - | - | - | - | - | - | - | - | - | - | - | - | - | - | - | - | - | - | - | - | - | - |
| W16A21 | A | A | G | C | C | A | A | G | G | G | G | A | A | - | - | - | - | - | - | - | - | - | - | - | - | - | - | - | - | - | - | - | - | - | - | - | - | - | - | - | - | - | - | - | - | - | - | - | - | - | - | - | - | - | - | - | - | - | - | - |
| W6C7 | A | T | C | T | T | C | A | G | A | C | G | A | T | G | T | G | T | T | C | T | A | T | G | - | - | - | - | - | - | - | - | - | - | - | - | - | - | - | - | - | - | - | - | - | - | - | - | - | - | - | - | - | - | - | - | - | - | - | - | - |
| G12H12 | A | A | C | C | G | A | A | G | C | A | C | A | G | T | T | G | C | T | C | A | A | T | G | A | A | C | T | G | A | G | A | C | T | G | G | C | T | C | A | A | A | A | T | G | C | A | C | T | - | - | - | - | - | - | - | - | - | - | - | - |
| W1C2 | A | G | C | C | C | T | T | C | A | A | C | G | G | C | A | A | C | T | C | C | G | C | G | G | A | C | T | G | G | A | C | G | A | C | G | C | T | G | T | A | C | C | - | - | - | - | - | - | - | - | - | - | - | - | - | - | - | - | - | - |

| L5E4 | - | - | - | - | - | - | - | - | - | - | - | - | - | - | - | - | - | - | - | - | - | - | - | - | - | - | - | - | - | - | - | - | - | - | - | - | - | - | - | - | - | - | - | - | - | - | - | - | - | - | - | - | - | - | - | - | - | - | - | - |
| --- | --- | --- | --- | --- | --- | --- | --- | --- | --- | --- | --- | --- | --- | --- | --- | --- | --- | --- | --- | --- | --- | --- | --- | --- | --- | --- | --- | --- | --- | --- | --- | --- | --- | --- | --- | --- | --- | --- | --- | --- | --- | --- | --- | --- | --- | --- | --- | --- | --- | --- | --- | --- | --- | --- | --- | --- | --- | --- | --- | --- |
| W12F4 | - | - | - | - | - | - | - | - | - | - | - | - | - | - | - | - | - | - | - | - | - | - | - | - | - | - | - | - | - | - | - | - | - | - | - | - | - | - | - | - | - | - | - | - | - | - | - | - | - | - | - | - | - | - | - | - | - | - | - | - |
| G2D9 | G | C | A | A | T | G | C | A | A | C | G | T | G | C | T | G | C | A | G | G | G | A | A | T | T | G | C | C | C | C | C | A | A | C | T | G | G | A | T | C | C | C | A | A | T | G | C | A | A | T | G | C | A | C | C | G | C | T | A | T |
| G3H12 | - | - | - | - | - | - | - | - | - | - | - | - | - | - | - | - | - | - | - | - | - | - | - | - | - | - | - | - | - | - | - | - | - | - | - | - | - | - | - | - | - | - | - | - | - | - | - | - | - | - | - | - | - | - | - | - | - | - | - | - |
| W8E4 | - | - | - | - | - | - | - | - | - | - | - | - | - | - | - | - | - | - | - | - | - | - | - | - | - | - | - | - | - | - | - | - | - | - | - | - | - | - | - | - | - | - | - | - | - | - | - | - | - | - | - | - | - | - | - | - | - | - | - | - |
| G12G1 | - | - | - | - | - | - | - | - | - | - | - | - | - | - | - | - | - | - | - | - | - | - | - | - | - | - | - | - | - | - | - | - | - | - | - | - | - | - | - | - | - | - | - | - | - | - | - | - | - | - | - | - | - | - | - | - | - | - | - | - |
| S18E7 | - | - | - | - | - | - | - | - | - | - | - | - | - | - | - | - | - | - | - | - | - | - | - | - | - | - | - | - | - | - | - | - | - | - | - | - | - | - | - | - | - | - | - | - | - | - | - | - | - | - | - | - | - | - | - | - | - | - | - | - |
| G21F11 | - | - | - | - | - | - | - | - | - | - | - | - | - | - | - | - | - | - | - | - | - | - | - | - | - | - | - | - | - | - | - | - | - | - | - | - | - | - | - | - | - | - | - | - | - | - | - | - | - | - | - | - | - | - | - | - | - | - | - | - |
| G7F4 | - | - | - | - | - | - | - | - | - | - | - | - | - | - | - | - | - | - | - | - | - | - | - | - | - | - | - | - | - | - | - | - | - | - | - | - | - | - | - | - | - | - | - | - | - | - | - | - | - | - | - | - | - | - | - | - | - | - | - | - |
| G10H7 | - | - | - | - | - | - | - | - | - | - | - | - | - | - | - | - | - | - | - | - | - | - | - | - | - | - | - | - | - | - | - | - | - | - | - | - | - | - | - | - | - | - | - | - | - | - | - | - | - | - | - | - | - | - | - | - | - | - | - | - |
| W7F8 | - | - | - | - | - | - | - | - | - | - | - | - | - | - | - | - | - | - | - | - | - | - | - | - | - | - | - | - | - | - | - | - | - | - | - | - | - | - | - | - | - | - | - | - | - | - | - | - | - | - | - | - | - | - | - | - | - | - | - | - |
| W16A21 | - | - | - | - | - | - | - | - | - | - | - | - | - | - | - | - | - | - | - | - | - | - | - | - | - | - | - | - | - | - | - | - | - | - | - | - | - | - | - | - | - | - | - | - | - | - | - | - | - | - | - | - | - | - | - | - | - | - | - | - |
| W6C7 | - | - | - | - | - | - | - | - | - | - | - | - | - | - | - | - | - | - | - | - | - | - | - | - | - | - | - | - | - | - | - | - | - | - | - | - | - | - | - | - | - | - | - | - | - | - | - | - | - | - | - | - | - | - | - | - | - | - | - | - |
| G12H12 | - | - | - | - | - | - | - | - | - | - | - | - | - | - | - | - | - | - | - | - | - | - | - | - | - | - | - | - | - | - | - | - | - | - | - | - | - | - | - | - | - | - | - | - | - | - | - | - | - | - | - | - | - | - | - | - | - | - | - | - |
| W1C2 | - | - | - | - | - | - | - | - | - | - | - | - | - | - | - | - | - | - | - | - | - | - | - | - | - | - | - | - | - | - | - | - | - | - | - | - | - | - | - | - | - | - | - | - | - | - | - | - | - | - | - | - | - | - | - | - | - | - | - | - |

| L5E4 | - | - | - | - | - | - | - | - | - | - | - | - | - | - | - | - | - | - | - | - | - | - | - | - | - | - | - | - | - | - | - | - | - | - | - | - | - | - | - | - | - | - | - | - | - | - | - | - | - | - | - | - | - | - | - | - | - | - | - | - |
| --- | --- | --- | --- | --- | --- | --- | --- | --- | --- | --- | --- | --- | --- | --- | --- | --- | --- | --- | --- | --- | --- | --- | --- | --- | --- | --- | --- | --- | --- | --- | --- | --- | --- | --- | --- | --- | --- | --- | --- | --- | --- | --- | --- | --- | --- | --- | --- | --- | --- | --- | --- | --- | --- | --- | --- | --- | --- | --- | --- | --- |
| W12F4 | - | - | - | - | - | - | - | - | - | - | - | - | - | - | - | - | - | - | - | - | - | - | - | - | - | - | - | - | - | - | - | - | - | - | - | - | - | - | - | - | - | - | - | - | - | - | - | - | - | - | - | - | - | - | - | - | - | - | - | - |
| G2D9 | G | A | A | A | G | C | C | G | A | C | G | C | A | A | T | A | T | T | T | T | A | C | T | C | A | A | T | G | A | A | A | T | C | C | A | A | T | T | C | T | A | T | G | A | A | A | G | C | C | A | A | C | G | C | G | C | G | G | A | A |
| G3H12 | - | - | - | - | - | - | - | - | - | - | - | - | - | - | - | - | - | - | - | - | - | - | - | - | - | - | - | - | - | - | - | - | - | - | - | - | - | - | - | - | - | - | - | - | - | - | - | - | - | A | A | C | G | C | C | T | T | A | G | A |
| W8E4 | - | - | - | - | - | - | - | - | - | - | - | - | - | - | - | - | - | - | - | - | - | - | - | - | - | - | - | - | - | - | - | - | - | - | - | - | - | - | - | - | - | - | - | - | - | - | - | - | - | - | - | - | - | - | - | - | - | - | - | - |
| G12G1 | - | - | - | - | - | - | - | - | - | - | - | - | - | - | - | - | - | - | - | - | - | - | - | - | - | - | - | - | - | - | - | - | - | - | - | - | - | - | - | - | - | - | - | - | - | - | - | - | - | - | - | - | - | - | - | - | - | - | - | - |
| S18E7 | - | - | - | - | - | - | - | - | - | - | - | - | - | - | - | - | - | - | - | - | - | - | - | - | - | - | - | - | - | - | - | - | - | - | - | - | - | - | - | - | - | - | - | - | - | - | - | - | - | - | - | - | - | - | - | - | - | - | - | - |
| G21F11 | - | - | - | - | - | - | - | - | - | - | - | - | - | - | - | - | - | - | - | - | - | - | - | - | - | - | - | - | - | - | - | - | - | - | - | - | - | - | - | - | - | - | - | - | - | - | - | - | - | - | - | - | - | - | - | - | - | - | - | - |
| G7F4 | - | - | - | - | - | - | - | - | - | - | - | - | - | - | - | - | - | - | - | - | - | - | - | - | - | - | - | - | - | - | - | - | - | - | - | - | - | - | - | - | - | - | - | - | - | - | - | - | - | - | - | - | - | - | - | - | - | - | - | - |
| G10H7 | - | - | - | - | - | - | - | - | - | - | - | - | - | - | - | - | - | - | - | - | - | - | - | - | - | - | - | - | - | - | - | - | - | - | - | - | - | - | - | - | - | - | - | - | - | - | - | - | - | - | - | - | - | - | - | - | - | - | - | - |
| W7F8 | - | - | - | - | - | - | - | - | - | - | - | - | - | - | - | - | - | - | - | - | - | - | - | - | - | - | - | - | - | - | - | - | - | - | - | - | - | - | - | - | - | - | - | - | - | - | - | - | - | - | - | - | - | - | - | - | - | - | - | - |
| W16A21 | - | - | - | - | - | - | - | - | - | - | - | - | - | - | - | - | - | - | - | - | - | - | - | - | - | - | - | - | - | - | - | - | - | - | - | - | - | - | - | - | - | - | - | - | - | - | - | - | G | C | A | C | G | A | G | G | C | C | T | C |
| W6C7 | - | - | - | - | - | - | - | - | - | - | - | - | - | - | - | - | - | - | - | - | - | - | - | - | - | - | - | - | - | - | - | - | - | - | - | - | - | - | - | - | - | - | - | - | - | - | - | - | - | - | - | - | - | - | - | - | - | - | - | - |
| G12H12 | - | - | - | - | - | - | - | - | - | - | - | - | - | - | - | - | - | - | - | - | - | - | - | - | - | - | - | - | - | - | - | - | - | - | - | - | - | - | - | - | - | - | - | - | - | - | - | - | - | - | - | - | - | - | - | - | - | - | - | - |
| W1C2 | - | - | - | - | - | - | - | - | - | - | - | - | - | - | - | - | - | - | - | - | - | - | - | - | - | - | - | - | - | - | - | - | - | - | - | - | - | - | - | - | - | - | - | - | - | - | - | - | - | - | - | - | - | - | - | - | - | - | - | - |

| L5E4 | - | - | - | - | - | - | - | - | - | - | - | - | - | - | - | - | - | - | - | - | - | - | - | - | - | - | - | - | - | - | - | - | - | - | - | - | - | - | - | - | - | - | - | - | - | - | - | - | - | - | - | - | - | - | - | - | - | - | - | - |
| --- | --- | --- | --- | --- | --- | --- | --- | --- | --- | --- | --- | --- | --- | --- | --- | --- | --- | --- | --- | --- | --- | --- | --- | --- | --- | --- | --- | --- | --- | --- | --- | --- | --- | --- | --- | --- | --- | --- | --- | --- | --- | --- | --- | --- | --- | --- | --- | --- | --- | --- | --- | --- | --- | --- | --- | --- | --- | --- | --- | --- |
| W12F4 | - | - | - | - | - | - | - | - | - | - | - | - | - | - | - | - | - | - | - | - | - | - | - | - | - | - | - | - | - | - | - | - | - | - | - | - | - | - | - | - | - | - | - | - | - | - | - | - | - | - | - | - | - | - | - | - | - | - | - | - |
| G2D9 | T | T | A | C | T | C | G | A | T | G | A | A | G | C | G | C | G | C | T | C | A | G | C | A | C | A | G | G | G | A | A | C | G | C | C | T | C | A | T | T | T | T | T | A | C | A | T | G | C | G | A | C | T | G | G | T | C | C | C | T |
| G3H12 | C | T | G | G | G | A | A | G | C | A | A | T | G | C | A | - | G | C | G | C | G | A | A | A | C | A | A | T | C | A | A | T | T | A | A | T | C | A | A | T | G | C | A | G | C | G | C | A | A | A | A | C | C | C | T | A | A | C | C | T |
| W8E4 | - | - | - | - | - | - | - | - | - | - | - | - | - | - | - | - | - | - | - | - | - | - | - | - | - | - | - | - | - | - | - | - | - | - | - | - | - | - | - | - | - | - | - | - | - | - | - | - | - | - | - | - | - | - | - | - | - | - | - | - |
| G12G1 | - | - | - | - | - | - | - | - | - | - | - | - | - | - | - | - | - | - | - | - | - | - | - | - | - | - | - | - | - | - | - | - | - | - | - | - | - | - | - | - | - | - | - | - | - | - | - | - | - | - | - | - | - | - | - | - | - | - | - | - |
| S18E7 | - | - | - | - | - | - | - | - | - | - | - | - | - | - | - | - | - | - | - | - | - | - | - | - | - | - | - | - | - | - | - | - | - | - | - | - | - | - | - | - | - | - | - | - | - | - | - | - | - | - | - | - | - | - | - | - | - | - | - | - |
| G21F11 | - | - | - | - | - | - | - | - | - | - | - | - | - | - | - | - | - | - | - | - | - | - | - | - | - | - | - | - | - | - | - | - | - | - | - | - | - | - | - | - | - | - | - | - | - | - | - | - | - | - | - | - | - | - | - | - | - | - | - | - |
| G7F4 | - | - | - | - | - | - | - | - | - | - | - | - | - | - | - | - | - | - | - | - | - | - | - | - | - | - | - | - | - | - | - | - | - | - | - | - | - | - | - | - | - | - | - | - | - | - | - | - | - | - | - | - | - | - | - | - | - | - | - | - |
| G10H7 | - | - | - | - | - | - | - | - | - | - | - | - | - | - | - | - | - | - | - | - | - | - | - | - | - | - | - | - | - | - | - | - | - | - | - | - | - | - | - | - | - | - | - | - | - | - | - | - | - | - | - | - | - | - | - | - | - | - | - | - |
| W7F8 | - | - | - | - | - | - | - | - | - | - | - | - | - | - | - | - | - | - | - | - | - | - | - | - | - | - | - | - | - | - | - | - | - | - | - | - | - | - | - | - | - | - | - | - | - | - | - | - | - | - | - | - | - | - | - | - | - | - | - | - |
| W16A21 | G | T | G | C | C | G | A | A | T | T | C | G | G | C | A | C | G | A | G | G | T | A | T | A | A | C | G | A | C | A | A | C | A | T | G | T | T | T | T | C | A | T | T | T | C | A | A | T | G | A | A | T | T | A | T | T | A | T | T | T |
| W6C7 | - | - | - | - | - | - | - | - | - | - | - | - | - | - | - | - | - | - | - | - | - | - | - | - | - | - | - | - | - | - | - | - | - | - | - | - | - | - | - | - | - | - | - | - | - | - | - | - | - | - | - | - | - | - | - | - | - | - | - | - |
| G12H12 | - | - | - | - | - | - | - | - | - | - | - | - | - | - | - | - | - | - | - | - | - | - | - | - | - | - | - | - | - | - | - | - | - | - | - | - | - | - | - | - | - | - | - | - | - | - | - | - | - | - | - | - | - | - | - | - | - | - | - | - |
| W1C2 | - | - | - | - | - | - | - | - | - | - | - | - | - | - | - | - | - | - | - | - | - | - | - | - | - | - | - | G | G | A | T | C | A | C | T | A | T | A | A | C | C | C | G | A | A | C | C | C | G | C | T | T | A | C | T | G | C | A | G | C |

| L5E4 | - | - | - | - | - | - | - | - | - | - | - | - | - | - | - | - | - | - | - | - | - | - | - | - | - | - | - | - | - | - | - | - | - | - | - | - | - | - | - | - | - | - | - | - | - | - | - | - | - | - | - | - | - | - | - | - | - | - | - | - |
| --- | --- | --- | --- | --- | --- | --- | --- | --- | --- | --- | --- | --- | --- | --- | --- | --- | --- | --- | --- | --- | --- | --- | --- | --- | --- | --- | --- | --- | --- | --- | --- | --- | --- | --- | --- | --- | --- | --- | --- | --- | --- | --- | --- | --- | --- | --- | --- | --- | --- | --- | --- | --- | --- | --- | --- | --- | --- | --- | --- | --- |
| W12F4 | - | - | - | - | - | - | - | - | - | - | - | - | - | - | - | - | - | - | - | - | G | A | C | A | G | C | A | A | C | T | C | C | T | T | A | C | C | A | A | T | G | A | G | G | T | C | G | C | T | A | C | A | C | G | G | C | T | T | A | A |
| G2D9 | A | A | A | - | - | - | C | T | G | G | T | T | C | G | C | A | A | T | G | C | A | T | G | T | G | A | T | - | C | T | C | T | T | T | C | A | A | C | T | G | A | G | A | G | A | C | A | T | T | A | G | A | G | C | G | A | A | A | C | A |
| G3H12 | G | A | A | T | C | A | C | C | G | T | G | T | T | T | T | T | G | T | C | A | A | T | G | A | G | A | T | A | C | T | C | C | C | T | G | A | A | C | T | G | C | A | A | C | G | C | C | T | T | T | T | A | C | T | G | G | A | A | C | G |
| W8E4 | - | - | - | - | - | - | - | - | - | - | - | - | - | - | - | - | A | T | G | C | A | T | G | G | G | A | T | - | C | C | C | - | - | - | - | A | A | T | C | T | G | A | A | T | G | C | A | G | C | G | C | G | A | C | G | G | A | A | T | G |
| G12G1 | - | - | - | - | - | - | - | - | - | - | - | - | - | - | - | - | - | - | - | - | - | - | - | - | - | - | - | - | - | - | - | - | - | - | - | - | - | - | - | - | - | - | - | - | - | - | - | - | - | - | - | - | - | - | - | - | - | - | - | - |
| S18E7 | - | - | - | - | - | - | - | - | - | - | - | - | - | - | - | - | - | - | - | - | - | - | - | - | - | - | - | - | - | - | - | - | - | - | - | - | - | - | - | - | - | - | - | - | - | - | - | - | - | - | - | - | - | - | - | - | - | - | - | - |
| G21F11 | - | - | - | - | - | - | - | - | - | - | - | - | - | - | - | - | - | - | - | - | - | - | - | - | - | - | - | - | - | - | - | - | - | - | - | - | - | - | - | - | - | - | - | - | - | - | - | - | - | - | - | - | - | - | - | - | - | - | - | - |
| G7F4 | - | - | - | - | - | - | - | - | - | - | - | - | - | - | - | - | - | - | - | - | - | - | - | - | - | - | - | - | - | - | - | - | - | - | - | - | - | - | - | - | - | - | - | - | - | - | - | - | - | - | - | - | - | - | - | - | - | - | - | - |
| G10H7 | - | - | - | - | - | - | - | - | - | - | - | - | - | - | - | - | - | - | - | - | - | - | - | - | - | - | - | - | - | - | - | - | - | - | - | - | - | - | - | - | - | - | - | - | - | - | - | - | - | - | - | - | - | - | - | - | - | - | - | - |
| W7F8 | - | - | - | - | - | - | - | - | - | - | - | - | - | - | - | - | - | - | - | - | - | - | - | - | - | - | - | - | - | - | - | - | - | - | - | - | - | - | - | - | - | - | - | - | - | - | - | - | - | - | - | - | - | - | - | - | - | - | - | - |
| W16A21 | T | T | G | A | A | A | A | T | A | A | C | A | A | G | A | T | C | G | A | C | A | C | G | T | T | T | T | C | A | A | T | T | G | A | A | T | T | T | C | C | A | A | G | A | C | A | G | G | T | T | G | T | T | T | T | T | T | G | G | T |
| W6C7 | - | - | - | - | - | - | - | - | - | - | - | - | - | - | - | - | - | - | - | - | - | - | - | - | - | - | - | - | - | - | - | - | - | - | - | - | - | - | - | - | - | - | - | - | - | - | - | - | - | - | - | - | - | - | - | - | - | - | - | - |
| G12H12 | - | - | - | - | - | - | - | - | - | - | - | - | - | - | - | - | - | - | - | - | - | - | - | - | - | - | - | - | - | - | - | - | - | - | - | - | - | - | - | - | - | - | - | - | - | - | - | - | - | - | - | - | - | - | - | - | - | - | - | - |
| W1C2 | C | G | C | T | C | T | A | C | G | A | A | A | C | C | T | C | A | T | T | C | A | A | C | A | G | A | A | A | C | G | C | A | C | T | C | A | A | C | T | G | A | A | C | A | C | T | G | C | T | G | T | A | C | A | G | A | C | T | C | T |

| L5E4 | - | - | - | - | - | - | - | - | - | - | - | - | - | - | - | - | - | - | - | - | - | - | - | - | - | - | - | - | - | - | - | - | - | - | - | - | - | - | - | - | - | - | - | - | - | - | - | - | - | - | - | - | - | - | - | - | - | - | - | - |
| --- | --- | --- | --- | --- | --- | --- | --- | --- | --- | --- | --- | --- | --- | --- | --- | --- | --- | --- | --- | --- | --- | --- | --- | --- | --- | --- | --- | --- | --- | --- | --- | --- | --- | --- | --- | --- | --- | --- | --- | --- | --- | --- | --- | --- | --- | --- | --- | --- | --- | --- | --- | --- | --- | --- | --- | --- | --- | --- | --- | --- |
| W12F4 | C | A | C | A | A | G | A | C | T | G | C | A | A | C | T | C | T | C | T | G | G | C | C | C | T | G | G | G | G | G | A | G | T | A | T | T | A | T | C | A | G | A | C | G | A | T | A | A | A | G | T | A | A | T | C | C | G | C | T | T |
| G2D9 | C | T | T | T | G | A | A | A | C | G | C | A | A | A | G | C | C | C | T | G | C | A | G | C | G | C | A | A | A | G | C | G | T | T | G | A | A | T | G | T | G | A | T | C | T | C | T | C | T | G | A | A | T | T | A | T | T | C | T | T |
| G3H12 | C | G | C | T | C | A | A | C | T | G | G | A | A | C | G | C | A | G | T | G | T | A | G | T | G | T | C | A | A | C | C | G | C | T | G | A | A | G | G | T | G | A | T | C | T | C | G | C | T | G | A | A | T | T | A | C | T | C | G | T |
| W8E4 | C | - | - | - | - | - | A | G | C | G | C | A | A | C | G | C | A | C | T | A | C | A | A | G | G | G | G | A | G | A | C | C | - | - | - | - | A | T | G | C | A | A | C | G | A | C | A | T | C | G | A | A | T | - | - | - | - | - | - | - |
| G12G1 | - | - | - | - | - | - | - | - | - | - | - | - | - | - | - | - | - | - | - | - | - | - | - | - | - | - | - | - | - | - | - | - | - | - | - | - | - | - | - | - | - | - | - | - | - | - | - | - | - | - | - | - | - | - | - | - | - | - | - | - |
| S18E7 | - | - | - | - | - | - | - | - | - | - | - | - | - | - | - | - | - | - | - | - | - | - | - | - | - | - | - | - | - | - | - | - | - | - | - | - | - | - | - | - | - | - | - | - | - | - | - | - | - | - | - | - | - | - | - | - | - | - | - | - |
| G21F11 | - | - | - | - | - | - | - | - | - | - | - | - | - | - | - | - | - | - | - | - | - | - | - | - | - | - | - | - | - | - | - | - | - | - | - | - | - | - | - | - | - | - | - | - | - | - | - | - | - | - | - | - | - | - | - | - | - | - | - | - |
| G7F4 | - | - | - | - | - | - | - | - | - | - | - | - | - | - | - | - | - | - | - | - | - | - | - | - | - | - | - | - | - | - | - | - | - | - | - | - | - | - | - | - | - | - | - | - | - | - | - | - | - | - | - | - | - | - | - | - | - | - | - | - |
| G10H7 | - | - | - | - | - | - | - | - | - | - | - | - | - | - | - | - | - | - | - | - | - | - | - | - | - | - | - | - | - | - | - | - | - | - | - | - | - | - | - | - | - | - | - | - | - | - | - | - | - | - | - | - | - | - | - | - | - | - | - | - |
| W7F8 | - | - | - | - | - | - | - | - | - | - | - | - | - | - | - | - | - | - | - | - | - | - | - | - | - | - | - | - | - | - | - | - | - | - | - | - | - | - | - | - | - | - | - | - | - | - | - | - | - | - | - | - | - | - | - | - | - | - | - | - |
| W16A21 | T | T | T | T | A | T | A | C | A | A | A | A | A | A | A | A | T | T | T | C | C | T | A | T | A | A | A | A | A | G | C | G | G | C | C | G | A | A | G | C | T | G | A | T | C | C | C | T | C | A | A | T | G | A | C | T | T | C | A | A |
| W6C7 | - | - | - | - | - | - | - | - | - | - | - | - | - | - | - | - | - | - | - | - | - | - | - | - | - | - | - | - | - | - | - | - | - | - | - | - | - | - | - | - | - | - | - | - | - | - | - | - | - | - | - | - | - | - | - | - | - | - | - | - |
| G12H12 | - | - | - | - | - | - | - | - | - | - | - | - | - | - | - | - | - | - | - | - | - | - | - | - | - | - | - | - | - | - | - | - | - | - | - | - | - | - | - | - | - | - | - | - | - | - | - | - | - | - | - | - | - | - | - | - | - | - | - | - |
| W1C2 | T | G | G | T | A | A | C | C | T | G | C | A | A | T | T | C | A | A | T | G | T | A | C | C | G | G | A | A | A | T | C | G | C | T | G | C | A | C | C | G | C | T | C | A | A | T | G | C | A | A | T | G | G | C | A | C | A | A | G | C |

| L5E4 | - | - | - | A | A | C | A | T | G | A | A | A | A | G | C | A | A | - | - | - | C | T | C | G | C | T | C | A | A | C | T | - | G | G | G | C | C | T | C | A | C | T | G | A | A | C | A | G | A | A | - | A | C | T | C | G | C | T | G | A |
| --- | --- | --- | --- | --- | --- | --- | --- | --- | --- | --- | --- | --- | --- | --- | --- | --- | --- | --- | --- | --- | --- | --- | --- | --- | --- | --- | --- | --- | --- | --- | --- | --- | --- | --- | --- | --- | --- | --- | --- | --- | --- | --- | --- | --- | --- | --- | --- | --- | --- | --- | --- | --- | --- | --- | --- | --- | --- | --- | --- | --- |
| W12F4 | T | G | T | A | G | C | G | G | C | A | A | G | A | A | A | T | G | - | - | - | C | T | T | A | T | T | C | C | G | C | - | - | A | G | C | T | C | T | A | G | C | C | A | G | A | C | A | A | A | A | T | G | C | T | T | A | T | T | C | C |
| G2D9 | T | G | T | A | C | T | G | G | C | A | C | G | C | A | A | T | G | - | - | - | C | A | G | C | G | C | A | A | C | G | C | C | G | T | G | C | G | A | T | A | C | T | C | G | C | T | T | C | A | C | G | G | G | A | A | C | T | T | A | C |
| G3H12 | T | G | A | A | C | T | G | T | C | A | C | G | T | T | C | T | G | - | - | - | C | A | G | C | G | G | A | A | C | G | C | G | C | T | C | A | A | C | T | - | - | - | - | - | - | - | - | - | - | - | G | A | A | A | A | T | C | T | A | T |
| W8E4 | - | G | C | A | G | C | G | C | A | A | C | G | G | A | A | T | G | - | - | - | C | A | G | C | G | C | A | A | C | A | C | A | C | T | G | A | A | T | C | A | C | T | C | A | T | T | G | A | A | G | A | G | G | A | A | C | T | C | A | C |
| G12G1 | - | - | - | A | A | C | G | T | C | A | A | G | C | A | A | T | G | - | - | - | T | T | G | A | C | A | G | A | G | T | C | G | C | T | G | A | A | G | A | G | T | T | A | A | A | C | A | A | T | A | A | T | G | A | C | A | A | A | G | T |
| S18E7 | - | - | - | - | - | - | - | - | - | - | - | - | - | - | - | - | - | - | - | - | - | - | - | - | - | - | - | - | - | - | - | T | T | C | G | G | T | G | A | A | A | T | G | A | - | - | - | G | C | G | A | G | A | A | T | C | A | A | G | A |
| G21F11 | - | - | - | - | - | - | - | - | - | - | - | - | - | - | - | - | - | - | - | - | - | - | - | - | - | - | - | - | - | - | - | - | - | - | - | - | - | - | - | - | - | - | - | - | - | - | - | - | - | - | - | - | - | - | - | - | - | - | - | - |
| G7F4 | - | - | - | - | - | - | - | - | - | - | - | - | - | - | - | - | - | - | - | - | - | - | - | - | - | T | C | G | A | A | G | A | A | G | C | C | G | C | T | G | T | A | A | A | A | C | A | T | T | C | A | T | T | G | C | T | A | C | G | C |
| G10H7 | C | C | C | G | G | T | T | C | C | G | C | G | G | G | A | T | C | A | T | C | C | G | G | T | T | C | C | A | G | A | G | T | A | T | C | G | G | G | T | T | C | C | G | G | G | G | G | A | T | C | A | C | C | C | A | C | T | A | A | T |
| W7F8 | - | - | - | - | - | - | - | - | - | - | - | - | - | - | - | - | - | - | - | - | - | - | - | - | - | - | - | A | T | T | T | T | C | G | G | G | G | C | A | A | C | T | C | G | A | C | G | C | A | A | T | T | A | G | T | A | G | C | G | A |
| W16A21 | C | T | T | T | T | T | G | T | C | C | A | A | T | A | C | T | C | G | - | A | T | G | G | A | C | A | A | C | A | T | A | T | A | T | G | T | A | C | A | A | C | C | A | A | G | A | G | G | A | A | G | T | C | T | C | T | A | G | C | A |
| W6C7 | - | - | - | - | - | - | - | - | - | - | - | A | C | A | C | T | A | A | T | A | C | A | G | G | A | T | T | T | C | C | T | C | G | C | A | G | G | G | A | A | G | T | C | G | T | T | C | A | C | A | A | G | C | A | T | T | T | C | G | A |
| G12H12 | - | - | - | - | - | - | - | - | - | - | - | - | - | - | - | - | - | - | - | - | - | - | C | G | A | G | G | G | A | C | T | T | G | G | A | C | G | C | C | G | C | G | T | A | C | C | G | G | A | T | A | C | C | G | A | - | T | C | T | A |
| W1C2 | T | G | G | A | C | T | G | T | T | G | A | G | C | G | C | T | G | A | A | G | T | G | A | A | T | G | A | G | G | C | T | C | A | A | G | A | G | G | C | A | T | T | C | G | A | T | A | G | A | C | T | T | G | A | A | G | C | C | C | G |

| L5E4 | A | C | T | A | A | T | A | C | A | A | C | A | T | G | A | A | A | A | G | C | A | - | A | C | T | C | G | C | - | - | - | - | - | - | T | A | G | G | C | T | G | C | C | C | C | T | C | C | A | - | - | T | G | A | A | C | A | G | C | A |
| --- | --- | --- | --- | --- | --- | --- | --- | --- | --- | --- | --- | --- | --- | --- | --- | --- | --- | --- | --- | --- | --- | --- | --- | --- | --- | --- | --- | --- | --- | --- | --- | --- | --- | --- | --- | --- | --- | --- | --- | --- | --- | --- | --- | --- | --- | --- | --- | --- | --- | --- | --- | --- | --- | --- | --- | --- | --- | --- | --- | --- |
| W12F4 | G | C | C | G | T | T | G | T | C | G | A | T | C | A | A | G | C | A | G | C | T | - | G | T | T | C | A | T | C | T | T | G | C | A | T | A | T | G | C | A | G | A | A | T | T | T | C | A | A | G | C | T | G | C | T | T | A | T | C | A |
| G2D9 | T | C | G | A | C | A | G | C | A | T | A | A | G | A | C | T | G | A | C | T | T | G | G | G | T | C | T | T | T | A | C | A | C | C | A | C | T | C | C | A | A | G | G | G | A | T | C | T | C | A | C | T | G | T | G | A | T | A | C | C |
| G3H12 | T | T | T | C | C | G | G | C | T | C | T | - | - | - | T | T | A | C | A | C | T | A | G | G | A | C | T | C | C | A | A | A | G | G | A | T | A | T | C | T | T | A | A | T | A | T | - | - | - | - | - | T | C | C | T | A | G | A | T | T |
| W8E4 | T | G | A | A | T | T | A | C | T | C | A | - | - | - | C | T | G | C | A | C | T | G | G | C | G | G | A | G | A | A | T | G | C | A | G | T | G | C | T | A | C | G | G | A | A | T | G | - | C | A | C | C | G | C | A | A | C | G | T | T |
| G12G1 | C | G | C | T | G | A | A | C | G | G | A | T | A | A | A | C | A | G | A | C | T | - | G | A | C | G | G | G | A | A | C | - | - | - | G | G | A | T | C | A | G | G | A | A | C | T | C | G | A | A | T | T | A | C | T | G | G | A | A | A |
| S18E7 | T | A | T | A | C | T | C | G | A | T | A | A | A | T | C | G | G | G | C | C | A | C | A | A | T | G | T | G | G | G | C | C | T | C | A | A | T | G | T | A | A | A | C | C | T | T | G | A | A | - | C | G | G | A | C | A | A | G | C | T |
| G21F11 | - | - | - | - | - | - | - | - | - | - | - | - | - | - | - | - | - | - | - | - | - | - | - | - | - | - | - | - | - | - | - | - | - | - | - | - | - | - | - | - | - | - | - | - | - | - | - | - | - | - | - | C | G | A | A | A | A | A | A | T |
| G7F4 | C | A | A | A | G | A | A | T | A | C | G | A | G | C | C | A | A | T | T | T | G | G | G | A | T | T | A | G | A | A | C | C | T | T | C | A | A | C | C | G | C | A | T | T | G | G | T | A | T | T | A | G | G | A | G | A | A | G | A | A |
| G10H7 | A | T | T | C | A | A | G | C | C | C | A | G | G | A | T | G | A | C | A | T | G | G | T | G | C | G | T | A | A | A | G | T | G | G | A | A | A | G | C | G | C | C | T | T | T | G | C | C | G | C | A | T | C | G | T | C | A | A | A | T |
| W7F8 | T | C | C | A | G | T | A | T | T | C | G | T | C | A | C | C | G | T | A | C | T | - | G | A | A | T | A | A | A | A | - | - | - | - | - | - | A | T | A | A | A | A | A | A | C | T | G | C | A | - | - | T | G | A | T | G | A | A | - | - |
| W16A21 | G | G | C | T | C | A | A | A | A | A | A | T | T | G | A | A | A | T | T | G | G | C | T | G | A | G | A | A | G | C | T | C | G | T | G | G | A | A | C | T | G | A | A | T | G | T | C | G | A | T | C | C | G | G | A | A | G | A | T | T |
| W6C7 | A | G | A | C | T | T | T | T | A | T | A | A | G | G | C | T | A | A | T | A | A | - | A | G | C | A | C | T | A | A | C | T | C | A | T | G | A | G | C | A | A | G | T | T | T | T | A | - | - | - | - | - | G | G | A | A | A | A | A | T |
| G12H12 | C | C | T | A | C | G | G | A | A | C | A | G | - | G | A | G | A | A | A | T | T | G | G | C | A | G | A | T | G | C | T | G | A | A | C | A | G | T | T | G | C | G | C | G | C | T | G | C | A | - | - | C | G | A | T | T | G | C | G | C |
| W1C2 | C | G | T | A | C | C | G | G | A | T | A | C | C | G | A | T | G | A | A | C | C | G | G | A | A | A | A | C | G | G | T | A | C | T | - | - | - | T | T | A | C | G | C | T | C | T | G | C | A | - | - | C | G | A | T | T | G | C | G | C |

| L5E4 | - | A | A | G | A | G | C | T | G | C | A | G | A | G | G | A | A | C | A | C | G | C | T | C | A | A | A | G | G | G | A | A | G | C | C | A | A | T | G | C | A | C | - | C | G | T | C | A | C | C | T | T | T | T | A | C | A | T | T | G |
| --- | --- | --- | --- | --- | --- | --- | --- | --- | --- | --- | --- | --- | --- | --- | --- | --- | --- | --- | --- | --- | --- | --- | --- | --- | --- | --- | --- | --- | --- | --- | --- | --- | --- | --- | --- | --- | --- | --- | --- | --- | --- | --- | --- | --- | --- | --- | --- | --- | --- | --- | --- | --- | --- | --- | --- | --- | --- | --- | --- | --- |
| W12F4 | - | T | G | C | G | T | T | A | C | T | A | C | T | G | C | A | A | C | A | A | G | C | T | G | A | C | A | T | A | G | A | T | A | A | C | A | T | T | C | C | A | C | - | A | T | A | C | A | G | A | A | A | T | A | A | T | A | T | T | G |
| G2D9 | T | A | C | A | C | C | A | G | A | G | G | A | G | A | G | G | A | A | T | A | C | T | T | T | A | A | T | G | A | A | G | G | C | T | C | T | C | C | G | A | A | T | - | T | T | T | T | A | T | T | A | G | T | C | C | C | C | A | A | A |
| G3H12 | - | - | C | A | C | G | A | G | T | G | G | A | A | T | T | G | A | G | T | G | C | T | T | T | A | A | A | A | G | A | T | G | T | T | C | T | T | C | G | G | A | T | - | T | T | T | T | A | T | T | G | G | T | C | C | C | A | G | A | A |
| W8E4 | - | - | C | A | C | C | A | A | A | T | G | A | T | T | T | G | G | T | T | G | T | T | A | T | A | A | A | A | C | G | T | G | T | T | C | T | A | C | G | A | A | T | - | T | T | T | T | A | T | T | G | G | T | C | C | C | A | A | A | A |
| G12G1 | C | T | C | C | A | G | T | G | T | T | T | C | T | T | C | A | G | A | T | G | T | T | T | A | A | A | G | A | T | T | G | T | G | C | C | A | A | C | G | T | A | T | - | A | C | T | T | - | C | A | A | A | T | - | A | C | G | A | T | A |
| S18E7 | A | T | A | A | A | G | C | G | A | A | T | A | A | A | G | A | C | C | G | A | G | C | T | G | T | T | G | A | A | - | A | T | G | T | T | G | T | T | G | T | G | - | - | - | - | T | C | C | G | A | A | A | A | A | A | A | G | A | G | A |
| G21F11 | G | G | A | A | A | T | C | G | C | T | G | A | A | T | C | G | C | A | A | T | T | C | C | T | G | C | C | G | A | T | G | A | T | C | G | G | G | A | T | G | A | T | - | T | T | T | C | G | T | T | C | G | T | G | G | T | G | G | A | T |
| G7F4 | G | C | C | C | C | T | A | T | G | A | C | C | C | A | T | G | A | A | G | A | A | T | A | C | A | A | A | G | A | T | G | C | A | A | T | T | C | T | C | A | G | T | - | T | T | T | C | A | T | G | C | A | T | T | - | C | G | A | T | C |
| G10H7 | C | C | A | A | G | T | G | T | G | G | C | T | C | C | T | A | G | A | A | G | G | A | C | A | C | T | T | G | T | C | T | C | T | T | T | T | T | C | C | A | A | C | C | T | A | C | C | A | C | G | G | A | T | G | A | A | T | A | A | A |
| W7F8 | - | - | - | - | - | - | - | - | - | T | T | T | C | T | T | G | G | C | C | A | G | G | C | C | A | A | G | T | T | T | C | T | T | G | C | T | T | C | A | T | A | T | - | T | T | C | T | - | T | A | G | A | T | C | A | C | A | A | A | A |
| W16A21 | T | A | A | A | G | T | G | G | G | C | A | C | T | A | A | G | T | T | T | A | T | C | T | G | A | C | T | T | C | A | C | T | C | C | T | A | A | T | G | A | A | A | T | C | A | T | T | T | C | A | C | T | G | A | G | A | G | G | T | G |
| W6C7 | G | T | C | A | C | G | A | A | A | A | T | T | T | A | A | A | C | - | A | A | G | A | T | G | T | T | G | T | A | C | A | T | C | T | T | T | T | T | A | A | A | C | A | C | T | T | C | C | T | T | A | A | T | A | A | C | G | A | A | A |
| G12H12 | C | T | T | G | C | C | C | G | G | G | C | A | C | G | C | G | C | T | G | C | A | T | T | G | A | A | T | C | T | C | C | C | T | A | T | A | T | T | G | G | A | T | - | T | T | C | G | A | T | A | C | A | A | G | A | C | G | C | C | G |
| W1C2 | C | T | T | G | C | C | C | G | G | G | C | A | C | G | C | G | C | T | G | C | A | T | T | G | A | A | T | C | G | G | T | T | T | A | C | A | C | C | G | - | - | - | - | - | - | - | - | - | - | - | - | - | - | - | - | T | T | C | C | A |

| L5E4 | A | C | C | C | G | A | G | C | T | G | C | A | C | G | G | G | A | A | C | A | C | A | A | G | A | A | A | C | C | G | C | C | A | C | C | C | T | T | T | A | C | A | T | T | G | A | C | C | C | G | C | G | G | T | G | G | A | C | G | G |
| --- | --- | --- | --- | --- | --- | --- | --- | --- | --- | --- | --- | --- | --- | --- | --- | --- | --- | --- | --- | --- | --- | --- | --- | --- | --- | --- | --- | --- | --- | --- | --- | --- | --- | --- | --- | --- | --- | --- | --- | --- | --- | --- | --- | --- | --- | --- | --- | --- | --- | --- | --- | --- | --- | --- | --- | --- | --- | --- | --- | --- |
| W12F4 | C | A | G | G | C | C | G | T | T | C | C | A | C | T | G | A | C | A | C | A | A | T | T | T | T | T | T | C | C | G | C | C | A | C | C | A | A | C | T | G | G | A | C | C | G | T | C | C | C | - | - | - | G | T | G | G | A | C | G | G |
| G2D9 | A | A | A | A | T | G | A | T | T | T | C | A | A | G | A | G | A | A | T | T | T | T | G | A | T | G | G | A | T | A | C | A | A | G | C | G | T | T | T | G | C | A | A | A | G | A | - | - | - | - | - | - | - | - | G | A | C | A | G | A |
| G3H12 | A | A | G | G | A | G | A | T | T | T | C | A | A | G | A | G | A | A | T | T | T | T | G | A | T | T | A | A | C | T | G | T | G | G | C | A | T | T | T | G | C | G | A | A | G | G | - | - | - | - | - | - | - | - | A | A | T | A | G | A |
| W8E4 | A | G | A | G | A | G | C | T | T | T | C | G | A | G | A | G | A | A | C | T | T | T | G | G | C | T | G | G | A | G | C | A | A | G | T | G | T | T | T | G | C | G | A | C | G | A | - | - | - | - | - | - | - | - | T | A | T | T | G | A |
| G12G1 | C | A | A | A | T | T | G | G | T | T | C | A | A | G | G | A | T | A | T | T | T | T | G | G | T | T | A | G | A | G | C | A | A | A | T | A | T | T | T | G | C | A | A | A | A | - | - | - | - | - | - | - | - | - | G | G | A | A | A | A |
| S18E7 | A | - | - | - | C | A | G | C | T | C | G | T | A | T | G | C | G | G | A | T | T | G | A | A | G | C | A | A | G | C | A | C | A | C | C | T | T | T | G | C | C | C | A | C | C | A | - | - | - | - | - | - | G | C | C | A | T | G | A | A |
| G21F11 | T | C | A | C | T | A | A | T | G | A | T | G | A | A | A | T | C | A | G | A | G | C | G | A | T | A | A | A | C | G | C | T | G | C | A | G | C | G | G | G | A | G | C | C | G | A | T | - | - | - | - | - | - | G | C | G | A | A | A | C |
| G7F4 | A | A | G | T | C | A | A | C | T | T | C | G | A | A | A | G | C | T | T | T | T | T | G | A | A | A | A | A | C | G | C | A | G | G | C | G | T | T | C | C | C | T | T | A | G | G | A | C | T | - | - | T | A | A | T | G | G | A | G | G |
| G10H7 | T | C | A | T | T | G | C | A | A | G | C | A | G | C | A | G | G | C | A | T | T | G | T | C | A | T | A | C | A | G | G | G | A | A | G | C | T | T | A | G | A | G | G | A | T | T | C | T | A | T | C | A | A | T | G | G | A | G | A | A |
| W7F8 | A | A | G | A | A | A | T | A | T | T | C | A | T | T | C | C | C | T | G | T | T | T | G | G | T | T | A | A | A | G | C | A | C | A | A | C | T | T | T | G | C | C | T | A | A | - | - | - | - | - | - | - | - | - | G | G | A | T | A | A |
| W16A21 | C | G | A | A | A | A | A | C | A | T | T | G | A | A | A | A | A | G | T | T | A | T | T | G | A | G | A | T | A | T | T | C | G | A | A | A | T | A | C | A | T | T | T | C | A | G | A | A | T | T | T | T | T | T | G | T | C | A | A | A |
| W6C7 | A | A | T | A | C | A | A | A | T | T | A | G | T | G | A | T | G | A | G | T | T | T | G | G | T | T | A | A | A | A | A | T | G | G | A | A | T | G | A | T | T | - | - | C | C | A | - | - | - | - | - | - | A | A | G | G | C | G | A | C |
| G12H12 | A | **T** | **A** | **A** | A | A | T | A | T | T | - | - | G | T | A | C | T | T | A | T | A | T | A | A | T | A | G | C | G | G | A | A | A | A | G | A | T | A | T | T | G | A | A | C | G | C | - | - | - | - | - | - | - | - | - | G | C | C | A | C |
| W1C2 | A | **T** | **A** | **A** | A | A | T | A | T | T | - | - | G | T | A | C | T | T | A | T | A | T | A | A | T | A | G | C | G | G | A | A | A | A | A | A | T | A | T | G | G | A | A | C | G | A | - | - | - | - | - | - | - | - | - | G | C | C | A | C |

| L5E4 | G | G | A | C | A | G | **T** | **A** | **A** | A | A | G | A | A | T | A | T | A | A | G | A | G | - | A | A | A | A | T | A | T | C | G | A | A | G | A | C | G | T | G | A | C | A | T | T | A | G | T | A | C | A | A | C | C | T | G | - | - | - | - |
| --- | --- | --- | --- | --- | --- | --- | --- | --- | --- | --- | --- | --- | --- | --- | --- | --- | --- | --- | --- | --- | --- | --- | --- | --- | --- | --- | --- | --- | --- | --- | --- | --- | --- | --- | --- | --- | --- | --- | --- | --- | --- | --- | --- | --- | --- | --- | --- | --- | --- | --- | --- | --- | --- | --- | --- | --- | --- | --- | --- | --- |
| W12F4 | A | G | A | C | A | A | **T** | **A** | **A** | G | A | G | A | T | C | A | C | G | A | G | A | G | G | A | A | A | A | A | A | T | C | G | A | A | G | A | C | G | C | G | A | C | A | T | T | A | G | T | A | C | A | A | C | T | T | G | - | - | - | - |
| G2D9 | T | T | T | C | G | A | T | T | A | C | A | T | A | A | T | A | G | C | G | G | A | G | - | - | - | A | A | G | A | T | T | T | G | G | G | A | C | G | C | A | T | C | A | C | A | T | G | A | C | C | G | T | C | T | T | A | - | - | - | - |
| G3H12 | T | C | C | C | G | A | T | T | A | C | G | A | A | A | T | G | G | C | A | G | A | G | - | - | - | A | A | G | A | T | C | T | G | G | G | A | C | G | C | G | C | C | A | C | T | T | T | A | C | A | A | G | C | T | T | C | - | - | - | - |
| W8E4 | T | G | A | A | A | A | T | A | T | A | A | C | G | A | A | A | G | C | G | G | A | G | - | - | - | A | A | G | A | T | T | T | A | T | G | A | C | G | C | G | C | C | A | C | T | C | T | C | C | C | G | T | C | T | T | A | - | - | - | - |
| G12G1 | C | T | G | C | G | T | T | T | G | T | A | - | A | G | A | A | T | A | C | C | G | G | - | G | A | A | T | T | A | T | T | G | A | T | A | A | A | G | C | A | A | A | A | T | T | T | G | T | G | C | C | T | C | T | T | T | T | - | - | A |
| S18E7 | A | C | C | T | A | C | T | G | A | C | A | A | C | T | T | C | A | T | A | A | G | G | A | A | C | G | T | C | G | T | C | G | A | A | A | A | T | C | C | A | A | T | A | T | T | T | G | C | T | G | G | T | T | T | T | G | T | - | - | T |
| G21F11 | T | G | T | C | A | T | T | A | A | T | G | G | A | T | G | G | G | C | G | T | C | T | T | A | G | A | C | A | T | T | T | G | G | C | T | A | C | T | G | A | G | A | T | T | T | T | G | A | G | C | G | A | T | T | T | A | G | - | G | A |
| G7F4 | T | T | T | G | C | G | T | G | C | T | G | C | T | A | T | A | A | A | A | C | A | T | G | A | A | G | G | A | G | T | T | T | A | T | A | A | C | - | C | A | A | T | G | T | T | T | A | G | C | G | G | C | C | T | T | A | - | - | - | A |
| G10H7 | A | C | A | G | A | T | G | A | G | C | G | T | A | A | A | C | T | T | A | T | G | G | C | G | G | C | T | C | T | A | C | G | A | C | A | T | T | T | T | G | T | A | G | A | C | C | A | T | T | A | T | T | C | T | T | A | T | A | G | T |
| W7F8 | C | T | C | C | A | G | T | T | T | C | C | - | A | A | C | A | T | A | A | A | A | C | - | T | A | A | A | T | A | T | C | G | A | T | A | A | G | G | C | G | G | A | A | G | C | T | G | T | A | A | C | A | C | C | T | G | A | A | A | T |
| W16A21 | A | T | T | T | C | T | T | C | A | A | A | T | A | T | C | A | T | C | A | G | G | A | T | T | C | G | A | C | G | C | T | G | A | T | T | G | C | T | T | T | G | T | T | T | A | A | A | G | T | A | G | A | T | G | G | T | T | C | C | T |
| W6C7 | T | A | A | T | G | A | T | G | A | T | A | A | A | G | C | G | A | T | A | A | A | A | A | A | A | G | C | A | G | C | C | C | A | A | A | A | T | G | C | G | - | T | A | T | - | - | - | - | - | G | A | G | A | C | T | A | A | - | - | C |
| G12H12 | T | A | - | T | G | A | C | G | T | C | A | - | A | A | A | A | T | T | G | A | A | G | - | - | - | - | T | T | T | T | C | T | T | A | C | A | C | T | C | T | T | T | A | T | T | T | T | A | T | A | G | C | C | G | T | C | T | - | - | T |
| W1C2 | A | A | A | T | G | A | C | G | T | C | A | - | A | A | A | A | T | T | G | A | A | G | - | - | - | - | T | T | T | T | C | T | T | A | C | A | T | T | C | T | T | T | A | T | T | T | T | A | T | A | G | C | C | G | T | C | T | - | - | T |

**├ 3’-UTR**

| L5E4 | - | A | A | G | C | T | A | T | A | T | T | T | C | A | C | - | - | - | - | G | C | A | G | C | A | G | C | T | A | C | A | G | C | T | A | G | - | - | A | A | T | T | A | A | T | G | T | T | A | A | C | A | T | C | T | G | A | A | C | T |
| --- | --- | --- | --- | --- | --- | --- | --- | --- | --- | --- | --- | --- | --- | --- | --- | --- | --- | --- | --- | --- | --- | --- | --- | --- | --- | --- | --- | --- | --- | --- | --- | --- | --- | --- | --- | --- | --- | --- | --- | --- | --- | --- | --- | --- | --- | --- | --- | --- | --- | --- | --- | --- | --- | --- | --- | --- | --- | --- | --- | --- |
| W12F4 | - | A | A | G | C | T | A | T | A | T | T | T | C | G | C | - | - | - | - | G | C | A | G | C | A | G | C | T | A | C | A | G | C | A | A | G | - | - | A | A | T | T | A | A | T | G | T | T | A | A | C | A | A | C | T | G | A | A | C | T |
| G2D9 | - | A | A | G | A | T | G | C | A | T | T | G | A | G | G | - | - | - | - | G | A | A | G | C | A | T | C | T | A | G | C | C | G | T | G | G | - | - | A | A | T | T | G | G | A | A | T | A | T | C | C | T | A | C | **T** | **G** | **A** | A | A | T |
| G3H12 | - | A | A | A | A | T | G | C | A | T | T | T | A | T | C | - | - | - | - | A | C | A | G | C | A | C | - | - | - | - | - | - | - | - | - | - | - | - | - | - | - | - | - | - | - | - | - | A | T | C | A | A | G | C | T | G | A | A | C | T |
| W8E4 | - | C | A | A | A | T | G | C | A | T | T | G | A | A | T | - | - | - | - | G | A | A | G | C | A | C | C | T | **T** | **A** | **G** | G | C | T | G | G | - | - | A | A | T | T | C | G | A | A | T | A | T | C | C | A | A | C | T | A | A | A | A | T |
| G12G1 | A | A | T | G | C | T | T | T | A | - | - | - | A | A | A | - | - | - | - | T | T | A | G | C | A | T | A | T | T | C | G | G | A | G | A | G | - | - | A | G | C | A | A | C | T | C | G | A | A | A | C | A | A | C | **T** | **G** | **A** | A | A | T |
| S18E7 | G | C | G | T | T | T | G | C | A | C | T | T | A | A | A | - | - | - | - | A | A | A | G | C | T | - | - | A | G | C | G | A | - | - | - | - | - | - | - | - | - | - | - | T | **T** | **G** | **A** | T | A | G | C | T | A | C | T | G | A | A | A | T |
| G21F11 | T | T | T | C | C | C | A | T | A | T | T | A | G | G | G | C | G | T | C | A | A | A | C | C | G | C | G | G | G | T | T | T | T | C | T | A | - | - | - | - | T | A | T | C | C | C | G | C | T | C | T | T | G | C | A | G | A | A | G | T |
| G7F4 | A | A | A | A | A | T | A | T | G | T | T | T | C | T | G | - | - | A | G | G | A | A | G | G | A | A | C | T | T | C | - | - | - | - | - | - | - | - | - | - | - | - | - | - | - | - | - | T | A | C | T | A | G | C | **T** | **G** | **A** | A | A | T |
| G10H7 | A | C | A | T | C | T | C | C | A | C | C | G | A | G | G | G | - | A | T | G | G | T | G | C | A | T | C | T | A | C | T | A | G | C | A | A | - | G | A | G | A | A | G | A | **T** | **A** | **A** | A | A | A | A | A | A | C | T | G | A | A | A | T |
| W7F8 | - | - | A | T | T | C | A | A | T | T | A | A | T | T | - | - | - | - | - | - | - | - | A | T | T | - | C | A | C | A | - | A | A | T | G | T | A | T | C | - | A | A | T | A | T | C | A | T | C | C | C | T | C | T | T | G | C | - | - | A |
| W16A21 | C | G | T | T | A | G | A | A | A | T | C | G | A | G | A | A | A | A | T | G | G | A | A | T | T | G | C | C | C | C | G | A | C | T | T | G | T | A | A | A | A | A | T | T | G | T | G | C | A | C | G | A | A | G | T | A | A | T | G | T |
| W6C7 | A | T | A | C | T | T | A | A | A | C | T | T | A | T | A | - | - | - | - | A | A | A | G | C | T | T | T | A | G | T | G | G | A | T | G | T | - | - | - | - | - | A | C | C | T | G | A | T | A | T | G | A | A | A | **T** | **G** | **A** | A | A | T |
| G12H12 | A | C | A | A | A | T | G | C | A | T | T | G | A | T | G | T | T | G | T | A | T | A | A | T | A | T | C | T | T | C | C | G | C | T | A | A | - | - | A | A | T | T | G | G | T | A | T | A | C | C | C | A | A | C | C | G | A | A | A | T |
| W1C2 | T | C | A | A | A | T | G | C | A | T | T | G | A | T | G | - | - | - | - | G | C | A | G | C | T | T | C | C | T | C | C | G | C | T | A | A | - | - | T | A | T | T | T | G | T | A | T | A | C | C | C | A | A | C | T | A | A | A | C | T |

| L5E4 | C | - | T | T | T | C | A | A | T | T | A | A | A | A | - | - | - | - | - | - | - | - | A | T | T | - | - | G | C | A | - | A | A | T | T | T | A | T | C | - | A | C | T | A | A | C | A | T | T | C | A | A | T | G | T | A | A | T | A | A |
| --- | --- | --- | --- | --- | --- | --- | --- | --- | --- | --- | --- | --- | --- | --- | --- | --- | --- | --- | --- | --- | --- | --- | --- | --- | --- | --- | --- | --- | --- | --- | --- | --- | --- | --- | --- | --- | --- | --- | --- | --- | --- | --- | --- | --- | --- | --- | --- | --- | --- | --- | --- | --- | --- | --- | --- | --- | --- | --- | --- | --- |
| W12F4 | - | - | T | T | T | C | A | A | T | T | A | A | A | C | - | - | - | - | - | - | - | - | A | T | T | - | C | A | C | A | - | A | A | T | G | T | A | T | C | - | A | T | T | T | A | C | A | T | T | C | A | A | T | G | T | A | A | T | A | A |
| G2D9 | - | - | T | T | T | C | A | A | T | T | T | A | A | C | - | - | - | - | - | - | - | - | A | T | T | - | C | A | C | A | - | A | A | T | G | A | A | T | C | - | A | C | T | A | A | C | A | T | T | C | T | A | T | G | T | A | A | T | A | A |
| G3H12 | - | - | A | T | **T** | **A** | **A** | A | T | T | A | A | A | T | - | - | - | - | - | - | - | - | C | T | T | - | C | A | C | A | - | A | A | T | G | T | A | T | C | - | A | C | T | A | A | C | A | T | T | C | A | A | T | G | T | A | A | T | A | A |
| W8E4 | - | - | A | A | T | G | A | A | T | T | A | A | C | C | - | - | - | - | - | - | - | - | G | T | T | - | C | A | C | A | - | A | A | T | G | T | A | T | C | - | A | C | T | A | A | C | A | T | T | C | A | A | T | G | T | A | A | A | G | A |
| G12G1 | - | - | A | T | T | T | A | A | T | T | A | A | A | C | A | T | T | A | A | A | C | - | A | T | T | - | C | A | C | A | - | A | A | T | G | T | A | T | C | - | G | A | T | A | A | C | A | T | T | C | A | A | T | G | T | A | A | T | A | A |
| S18E7 | - | - | A | T | T | C | A | A | T | T | A | A | A | C | - | - | - | - | - | - | - | - | A | T | T | G | C | A | A | A | - | A | A | T | G | T | A | T | C | - | A | C | T | A | A | C | A | T | T | C | A | T | C | G | T | A | A | T | A | A |
| G21F11 | T | C | T | G | T | T | G | A | G | T | G | C | A | C | - | - | - | - | - | - | - | - | C | T | T | C | T | A | C | G | G | A | A | T | C | C | A | T | C | G | A | C | C | A | T | C | A | T | **T** | **G** | **A** | A | A | G | T | A | A | T | A | A |
| G7F4 | - | - | A | T | T | C | A | A | T | T | A | A | A | C | - | - | - | - | - | - | - | - | A | T | T | - | C | G | C | A | - | A | A | C | G | T | A | T | C | - | A | T | T | A | A | C | A | T | T | C | A | A | T | G | T | A | A | T | A | A |
| G10H7 | - | - | A | T | T | C | A | A | T | C | A | A | A | C | - | - | - | - | - | - | - | - | A | T | T | - | C | A | C | A | - | A | A | T | G | T | C | A | C | - | A | C | T | A | A | T | A | T | T | C | A | A | T | G | T | A | A | T | A | A |
| W7F8 | A | A | A | G | C | T | T | T | A | T | C | C | G | A | A | - | - | - | - | T | T | G | G | T | T | A | A | A | A | C | A | A | **T** | **A** | **G** | G | C | A | A | C | T | G | A | T | T | G | G | A | - | C | A | A | T | G | T | A | A | T | A | A |
| W16A21 | T | G | A | G | A | G | A | A | T | A | T | A | A | C | A | C | C | A | A | A | **T** | **A** | **A** | T | T | T | T | A | A | A | T | C | A | A | A | T | T | T | T | C | A | C | C | A | A | C | A | T | T | C | A | A | A | G | T | A | A | T | A | T |
| W6C7 | - | - | A | T | T | C | A | A | T | T | A | A | A | C | - | - | - | - | - | - | - | - | A | T | T | - | C | A | A | A | - | A | A | T | G | T | A | T | C | - | A | C | T | A | A | C | A | T | T | C | A | A | T | G | T | A | A | T | A | A |
| G12H12 | - | - | A | T | T | C | A | A | T | - | A | A | A | C | - | - | - | - | - | - | - | - | A | C | T | - | C | A | T | A | - | A | A | T | T | T | A | T | C | - | A | C | T | T | A | C | A | T | T | C | A | A | T | G | T | A | A | T | T | A |
| W1C2 | C | A | A | T | T | C | A | A | T | T | A | A | A | C | - | - | - | - | - | - | - | - | A | T | T | - | C | A | G | A | - | A | A | T | T | T | A | T | C | - | A | C | T | A | A | C | A | T | T | C | A | A | T | G | T | A | A | T | A | A |

| L5E4 | T | T | T | T | T | G | T | - | - | - | G | A | A | G | A | T | A | A | A | G | - | T | A | C | G | A | A | A | A | T | G | A | A | T | T | G | T | T | A | A | C | - | T | T | C | T | G | A | T | G | T | G | A | A | G | A | A | A | T | A |
| --- | --- | --- | --- | --- | --- | --- | --- | --- | --- | --- | --- | --- | --- | --- | --- | --- | --- | --- | --- | --- | --- | --- | --- | --- | --- | --- | --- | --- | --- | --- | --- | --- | --- | --- | --- | --- | --- | --- | --- | --- | --- | --- | --- | --- | --- | --- | --- | --- | --- | --- | --- | --- | --- | --- | --- | --- | --- | --- | --- | --- |
| W12F4 | T | T | T | T | T | G | C | - | - | - | G | A | A | A | A | T | A | A | A | A | - | T | A | C | G | A | A | A | A | T | G | A | A | T | T | G | T | T | A | A | C | - | T | T | C | T | C | A | T | A | T | A | A | A | G | A | A | A | T | A |
| G2D9 | T | T | T | T | T | T | T | T | G | C | G | A | A | A | A | T | A | T | A | A | - | T | A | C | G | A | A | A | T | T | G | A | A | T | T | G | T | T | A | A | C | - | T | T | C | T | G | A | T | A | T | A | A | A | A | A | A | A | T | T |
| G3H12 | T | T | T | T | T | G | G | - | - | - | G | A | A | A | A | T | A | T | A | A | - | T | A | C | G | A | A | A | T | T | G | A | A | T | T | G | T | C | T | A | C | A | T | T | C | T | G | A | T | A | T | A | A | A | G | A | A | A | T | T |
| W8E4 | T | T | T | T | T | T | G | - | - | - | G | A | A | A | A | T | A | T | A | A | - | T | A | C | A | A | A | A | T | T | G | A | A | T | T | G | T | T | T | A | C | - | T | T | C | T | G | A | A | A | T | A | A | A | G | A | A | A | T | - |
| G12G1 | T | T | T | T | T | G | G | - | - | - | G | A | A | A | A | T | A | T | A | A | - | T | A | C | G | A | A | A | T | T | G | A | A | T | T | G | T | T | A | A | T | - | T | T | C | T | G | A | T | A | T | A | A | A | G | A | A | A | T | A |
| S18E7 | T | T | T | T | T | G | G | - | - | - | A | A | A | A | A | T | A | T | T | A | - | T | A | C | A | A | A | A | T | T | G | A | A | T | T | G | T | T | A | A | C | - | T | T | C | T | G | A | T | A | T | A | A | A | A | A | A | A | T | A |
| G21F11 | T | T | T | T | T | G | T | - | - | - | G | A | A | A | A | T | A | T | A | A | - | T | G | C | G | A | A | A | T | T | G | A | A | T | T | G | T | T | T | A | C | - | T | T | C | T | G | A | T | A | T | A | A | A | G | A | A | A | T | A |
| G7F4 | T | T | T | T | T | A | T | - | - | - | - | - | - | - | - | - | - | T | A | A | - | T | A | C | G | A | A | A | T | T | G | A | A | T | T | G | T | T | A | A | C | - | T | T | C | T | G | A | T | A | T | A | A | A | G | A | A | A | T | A |
| G10H7 | T | T | T | T | T | T | T | - | - | - | - | - | - | - | - | - | - | T | A | A | - | T | A | C | A | A | A | A | T | T | G | A | A | T | T | G | G | T | A | A | C | - | T | T | C | T | G | A | T | A | T | A | A | A | G | A | A | A | T | A |
| W7F8 | T | T | T | T | T | G | G | - | - | - | G | A | A | A | A | T | A | T | A | A | - | T | A | C | A | A | A | A | T | T | G | A | A | T | T | G | T | A | A | A | C | - | T | T | T | T | T | - | T | G | T | A | A | A | A | A | A | A | T | A |
| W16A21 | T | T | T | T | T | T | G | - | - | G | A | A | A | A | A | T | A | T | A | A | A | T | A | C | G | A | A | A | T | T | G | A | A | T | T | G | T | T | A | A | C | - | T | T | C | T | G | A | T | A | T | G | A | A | G | A | A | - | T | A |
| W6C7 | T | T | T | T | T | G | G | - | - | - | A | A | A | A | A | T | A | T | A | A | - | T | A | C | G | A | A | A | T | T | G | A | A | T | T | G | T | T | A | A | T | - | T | T | C | T | G | A | T | A | A | A | G | A | G | A | A | A | T | A |
| G12H12 | T | T | T | T | T | C | G | - | - | - | G | A | A | A | A | T | A | T | A | A | - | T | A | C | G | A | A | A | T | T | G | A | A | T | T | G | T | T | A | A | C | - | T | T | C | T | G | A | T | A | T | A | A | A | G | A | A | A | A | T |
| W1C2 | T | T | T | T | T | G | G | - | - | - | A | A | A | A | A | T | A | T | A | A | - | T | A | C | G | A | A | A | T | T | G | A | A | T | T | G | T | T | A | A | C | - | T | T | C | T | G | A | T | A | T | A | A | A | G | A | A | A | A | T |

| L5E4 | A | A | A | T | A | T | T | T | T | T |  |  |  |  |  |  |  |  |  |  |  |  |  |  |  |  |  |  |  |  |  |  |  |  |  |  |  |  |  |  |  |  |  |  |  |  |  |  |  |  |  |  |  |  |  |  |  |  |  |  |
| --- | --- | --- | --- | --- | --- | --- | --- | --- | --- | --- | --- | --- | --- | --- | --- | --- | --- | --- | --- | --- | --- | --- | --- | --- | --- | --- | --- | --- | --- | --- | --- | --- | --- | --- | --- | --- | --- | --- | --- | --- | --- | --- | --- | --- | --- | --- | --- | --- | --- | --- | --- | --- | --- | --- | --- | --- | --- | --- | --- | --- |
| W12F4 | A | A | A | T | A | T | T | T | T | T |  |  |  |  |  |  |  |  |  |  |  |  |  |  |  |  |  |  |  |  |  |  |  |  |  |  |  |  |  |  |  |  |  |  |  |  |  |  |  |  |  |  |  |  |  |  |  |  |  |  |
| G2D9 | A | A | A | T | A | T | T | T | T | T |  |  |  |  |  |  |  |  |  |  |  |  |  |  |  |  |  |  |  |  |  |  |  |  |  |  |  |  |  |  |  |  |  |  |  |  |  |  |  |  |  |  |  |  |  |  |  |  |  |  |
| G3H12 | A | A | A | T | A | T | T | T | T | T |  |  |  |  |  |  |  |  |  |  |  |  |  |  |  |  |  |  |  |  |  |  |  |  |  |  |  |  |  |  |  |  |  |  |  |  |  |  |  |  |  |  |  |  |  |  |  |  |  |  |
| W8E4 | - | - | A | T | G | T | - | - | - | - |  |  |  |  |  |  |  |  |  |  |  |  |  |  |  |  |  |  |  |  |  |  |  |  |  |  |  |  |  |  |  |  |  |  |  |  |  |  |  |  |  |  |  |  |  |  |  |  |  |  |
| G12G1 | A | A | A | T | A | T | T | T | T | T |  |  |  |  |  |  |  |  |  |  |  |  |  |  |  |  |  |  |  |  |  |  |  |  |  |  |  |  |  |  |  |  |  |  |  |  |  |  |  |  |  |  |  |  |  |  |  |  |  |  |
| S18E7 | A | A | A | T | A | T | T | T | T | T |  |  |  |  |  |  |  |  |  |  |  |  |  |  |  |  |  |  |  |  |  |  |  |  |  |  |  |  |  |  |  |  |  |  |  |  |  |  |  |  |  |  |  |  |  |  |  |  |  |  |
| G21F11 | A | A | A | T | A | T | T | T | T | T |  |  |  |  |  |  |  |  |  |  |  |  |  |  |  |  |  |  |  |  |  |  |  |  |  |  |  |  |  |  |  |  |  |  |  |  |  |  |  |  |  |  |  |  |  |  |  |  |  |  |
| G7F4 | A | A | A | T | A | T | T | T | T | T |  |  |  |  |  |  |  |  |  |  |  |  |  |  |  |  |  |  |  |  |  |  |  |  |  |  |  |  |  |  |  |  |  |  |  |  |  |  |  |  |  |  |  |  |  |  |  |  |  |  |
| G10H7 | A | A | A | T | A | T | T | T | T | T |  |  |  |  |  |  |  |  |  |  |  |  |  |  |  |  |  |  |  |  |  |  |  |  |  |  |  |  |  |  |  |  |  |  |  |  |  |  |  |  |  |  |  |  |  |  |  |  |  |  |
| W7F8 | A | A | A | T | A | T | T | C | T | T |  |  |  |  |  |  |  |  |  |  |  |  |  |  |  |  |  |  |  |  |  |  |  |  |  |  |  |  |  |  |  |  |  |  |  |  |  |  |  |  |  |  |  |  |  |  |  |  |  |  |
| W16A21 | A | A | A | T | A | T | T | T | T | T |  |  |  |  |  |  |  |  |  |  |  |  |  |  |  |  |  |  |  |  |  |  |  |  |  |  |  |  |  |  |  |  |  |  |  |  |  |  |  |  |  |  |  |  |  |  |  |  |  |  |
| W6C7 | A | A | A | T | A | T | T | T | T | C |  |  |  |  |  |  |  |  |  |  |  |  |  |  |  |  |  |  |  |  |  |  |  |  |  |  |  |  |  |  |  |  |  |  |  |  |  |  |  |  |  |  |  |  |  |  |  |  |  |  |
| G12H12 | A | A | A | T | A | T | T | T | T | T |  |  |  |  |  |  |  |  |  |  |  |  |  |  |  |  |  |  |  |  |  |  |  |  |  |  |  |  |  |  |  |  |  |  |  |  |  |  |  |  |  |  |  |  |  |  |  |  |  |  |
| W1C2 | A | A | A | T | A | T | T | T | T | T |  |  |  |  |  |  |  |  |  |  |  |  |  |  |  |  |  |  |  |  |  |  |  |  |  |  |  |  |  |  |  |  |  |  |  |  |  |  |  |  |  |  |  |  |  |  |  |  |  |  |

**Protein alignment**

| L5E4 | M | K | F | L | L | A | F | L | F | V | V | S | T | A | I | L | V | D | S | A | G | D | Q | N | T | D | A | E | L | I | Q | A | E | L | I | Q | - | - | - | - | - | - | - | - | - | - | - | - | - | - | - | - | - | - | - | - | - | - | R | E |
| --- | --- | --- | --- | --- | --- | --- | --- | --- | --- | --- | --- | --- | --- | --- | --- | --- | --- | --- | --- | --- | --- | --- | --- | --- | --- | --- | --- | --- | --- | --- | --- | --- | --- | --- | --- | --- | --- | --- | --- | --- | --- | --- | --- | --- | --- | --- | --- | --- | --- | --- | --- | --- | --- | --- | --- | --- | --- | --- | --- | --- |
| W12F4 | M | K | F | L | L | A | F | L | F | V | A | S | I | A | I | L | V | A | S | G | R | P | S | K | R | E | Q | Q | L | R | E | Q | Q | L | R | E | - | - | - | - | - | - | E | Q | L | A | E | E | Q | L | R | R | Q | Q | L | L | T | N | E | V |
| G2D9 | M | K | F | T | L | G | F | L | I | V | A | A | T | A | I | L | V | A | S | D | A | P | T | M | S | E | L | F | Q | E | R | L | K | L | V | T | E | F | T | V | L | M | T | K | L | D | A | N | A | M | Q | R | A | A | G | N | C | P | Q | L |
| G3H12 | M | K | Y | S | L | A | F | L | F | V | A | S | T | A | F | L | V | A | S | A | P | P | T | S | V | D | R | A | R | T | V | A | R | L | N | - | - | - | - | Q | L | L | D | R | V | E | R | N | V | A | Q | R | - | - | - | - | - | L | R | L |
| W8E4 | M | K | Y | S | L | A | F | L | F | V | A | A | T | A | I | L | A | A | S | A | L | P | S | T | Y | N | L | E | L | E | L | N | R | L | K | - | - | L | H | G | I | L | S | R | L | E | L | H | A | W | D | - | - | - | - | - | - | P | N | L |
| G12G1 | M | K | Y | S | L | A | F | L | V | V | A | S | T | A | I | L | V | A | S | M | N | P | S | A | E | R | E | R | R | E | L | L | A | A | L | - | - | - | - | - | - | - | N | R | A | G | G | N | P | A | G | - | - | - | - | - | - | - | T | S |
| S18E7 | M | K | F | L | F | A | F | V | F | V | A | S | T | V | F | L | V | D | S | T | G | P | H | S | N | A | P | K | L | T | D | K | E | L | A | - | - | - | - | - | - | - | - | - | - | - | - | - | - | - | - | - | - | - | - | - | - | - | A | Y |
| G21F11 | M | K | Y | S | L | A | F | L | F | V | A | A | T | F | I | L | V | D | S | G | T | I | P | S | A | N | A | A | P | Q | R | V | L | L | Q | M | S | - | - | - | - | - | - | - | - | - | - | - | - | - | - | - | - | - | - | - | - | H | E | I |
| G7F4 | M | K | Y | S | L | A | F | L | L | A | A | A | T | V | I | S | V | A | S | A | V | G | S | G | P | R | S | S | S | K | R | E | Q | L | R | - | - | - | - | - | - | - | - | - | - | - | - | - | - | - | - | - | - | - | - | - | - | T | A | E |
| G10H7 | M | K | Y | S | L | A | F | L | F | V | A | A | T | A | I | L | V | A | S | V | E | A | S | G | S | P | G | S | A | G | S | R | I | S | G | P | R | - | - | - | - | - | G | S | G | S | G | G | S | P | G | S | A | G | S | S | G | S | R | V |
| W7F8 | M | K | Y | L | L | A | F | L | I | V | A | S | T | A | I | L | V | A | S | T | G | G | N | G | A | G | S | S | G | A | G | L | S | D | E | - | - | - | - | - | - | - | E | K | A | E | K | M | I | A | - | - | - | - | - | - | - | - | T | L |
| W16A21 | M | K | C | L | L | T | F | L | F | V | A | S | T | A | I | L | V | A | S | A | P | P | P | P | G | G | S | L | S | A | G | S | S | S | A | G | - | - | - | - | - | - | S | S | S | Q | G | E | L | N | D | F | N | F | L | S | N | T | R | W |
| W6C7 | M | K | Y | S | F | A | F | L | F | V | A | A | T | A | I | S | M | A | S | A | A | G | E | F | D | G | F | Y | E | T | N | T | E | F | A | E | G | - | - | - | - | - | - | - | - | - | - | - | - | - | - | - | - | - | - | - | E | S | S | D |
| G12H12 | M | K | Y | S | L | A | F | L | F | V | A | S | T | A | F | L | M | A | S | A | A | P | K | R | - | - | - | - | A | A | Q | Q | S | A | E | - | G | L | P | - | - | - | - | - | - | - | - | - | - | - | - | - | - | - | - | - | - | - | - | - |
| W1C2 | M | K | Y | S | L | A | F | L | F | V | A | S | T | A | F | L | M | A | S | A | A | P | R | I | N | E | I | N | R | A | Q | A | T | V | D | - | R | L | S | G | E | L | A | A | L | Q | R | Q | L | R | G | L | D | D | A | V | P | D | H | Y |
|  |  |  |  |  |  |  |  |  |  |  |  |  |  |  |  |  |  |  |  |  |  |  |  |  |  |  |  |  |  |  |  |  |  |  |  |  |  |  |  |  |  |  |  |  |  |  |  |  |  |  |  |  |  |  |  |  |  |  |  |  |
| L5E4 | Q | Q | L | A | G | M | P | A | N | A | R | R | - | - | - | - | - | - | - | - | - | - | R | A | A | E | E | Q | H | E | K | Q | L | A | Q | L | G | - | - | - | - | - | - | - | - | - | - | - | - | - | - | - | - | - | - | - | - | - | - | - |
| W12F4 | A | T | R | L | N | T | R | L | Q | L | S | G | P | G | - | - | - | - | - | - | - | - | G | V | L | S | D | D | K | V | I | R | F | V | A | A | R | N | - | - | - | - | - | - | - | - | - | - | - | - | - | - | - | - | - | - | - | - | - | - |
| G2D9 | D | P | N | A | M | H | R | Y | E | S | R | R | N | I | L | L | N | E | I | Q | F | Y | E | S | Q | R | A | E | L | L | D | E | A | R | S | A | Q | G | T | P | H | F | Y | M | R | L | V | P | K | L | V | R | N | A | C | D | L | F | Q | L |
| G3H12 | G | S | N | A | A | R | N | N | Q | L | I | N | - | - | - | - | - | - | - | - | - | - | A | A | Q | N | P | N | L | N | H | R | V | F | V | N | E | I | L | P | - | - | - | - | - | - | - | - | - | - | - | - | - | - | - | - | - | - | E | L |
| W8E4 | N | - | - | A | A | R | R | N | A | A | Q | R | - | - | - | - | - | - | - | - | - | - | - | T | T | R | G | D | H | A | T | T | S | N | A | A | Q | - | - | - | - | - | - | - | - | - | - | - | - | - | - | R | N | A | - | - | - | - | - | - |
| G12G1 | S | N | V | D | R | V | A | E | E | L | N | N | - | - | - | - | - | - | - | - | - | - | N | D | K | V | A | E | R | I | N | R | L | T | G | T | D | - | - | - | - | - | - | - | - | - | - | - | - | - | - | - | - | - | - | - | - | - | - | - |
| S18E7 | L | A | L | K | E | T | F | G | E | M | S | E | - | - | - | - | - | - | - | - | - | - | - | - | - | N | Q | D | I | L | D | K | S | G | H | N | V | G | - | - | - | - | - | - | - | - | - | - | - | - | - | - | - | - | - | - | - | - | - | - |
| G21F11 | R | D | A | I | C | E | K | M | E | I | A | E | S | - | - | - | - | - | - | - | - | - | - | - | Q | F | L | P | M | I | G | M | I | F | V | R | G | G | - | - | - | - | - | - | - | - | - | - | - | - | - | - | - | - | - | - | - | - | - | - |
| G7F4 | R | D | R | K | L | E | E | A | A | V | K | H | S | - | - | - | - | - | - | - | - | - | - | L | L | R | Q | R | I | R | A | N | L | G | L | E | P | S | - | - | - | - | - | - | - | - | - | - | - | - | - | - | - | - | - | - | - | - | - | - |
| G10H7 | S | G | S | G | G | S | P | T | N | I | Q | A | Q | - | - | - | - | - | - | - | - | - | D | D | M | V | R | K | V | E | S | A | F | A | A | S | S | N | P | S | - | - | - | - | - | - | - | - | - | - | - | - | - | - | - | - | - | - | - | - |
| W7F8 | N | A | F | S | G | Q | L | D | A | I | S | S | - | - | - | - | - | - | - | - | - | - | D | P | V | F | V | T | V | L | N | K | N | K | - | - | - | - | - | - | - | - | - | - | - | - | - | - | - | - | - | - | - | - | - | - | - | - | - | - |
| W16A21 | T | T | Y | M | Y | N | Q | E | E | V | S | S | - | - | - | - | - | - | - | - | - | - | R | L | K | K | L | K | L | A | E | K | L | V | E | L | N | V | D | P | - | - | - | - | - | - | - | - | - | - | - | - | - | - | - | - | - | - | - | - |
| W6C7 | D | V | F | Y | D | T | N | T | G | F | P | R | R | - | - | - | - | - | - | - | - | - | E | V | V | H | K | H | F | E | D | F | Y | K | A | N | K | A | L | T | - | - | - | - | - | - | - | - | - | - | - | - | - | - | - | - | - | - | - | - |
| G12H12 | - | S | A | P | R | T | E | A | Q | L | L | N | - | - | - | - | - | - | - | - | - | - | - | - | - | - | - | E | L | R | L | A | Q | N | A | L | E | G | - | - | - | - | - | - | - | - | - | - | - | - | - | - | - | - | - | - | - | - | - | - |
| W1C2 | N | P | N | P | L | T | A | A | A | L | R | N | L | I | - | - | - | - | - | - | - | - | Q | Q | K | R | T | Q | L | N | T | A | V | Q | T | L | G | N | - | - | - | - | - | - | - | - | - | - | - | - | - | - | - | - | - | - | - | - | - | - |
|  |  |  |  |  |  |  |  |  |  |  |  |  |  |  |  |  |  |  |  |  |  |  |  |  |  |  |  |  |  |  |  |  |  |  |  |  |  |  |  |  |  |  |  |  |  |  |  |  |  |  |  |  |  |  |  |  |  |  |  |  |
| L5E4 | - | - | - | - | - | - | - | - | - | - | - | - | - | - | - | - | L | T | E | Q | K | L | A | E | L | I | Q | H | E | K | Q | L | A | R | L | P | L | H | E | - | - | - | - | - | - | - | - | - | - | - | - | - | - | - | - | - | - | - | - | - |
| W12F4 | - | - | - | - | - | - | - | - | - | - | - | - | - | - | - | - | A | Y | S | A | A | L | A | R | Q | N | A | Y | S | A | V | V | D | Q | A | A | V | H | - | - | - | - | - | - | - | - | - | - | - | - | - | - | - | - | - | - | - | - | - | - |
| G2D9 | R | D | I | R | A | K | H | F | E | T | Q | S | P | A | A | Q | S | V | E | C | D | L | S | E | L | F | F | V | L | A | R | N | A | A | Q | R | R | A | I | L | A | S | R | E | L | T | R | Q | H | K | T | D | L | G | L | Y | T | T | P | R |
| G3H12 | Q | R | L | L | L | E | R | A | Q | L | E | R | S | V | V | S | T | A | E | G | D | L | A | E | L | L | V | E | L | S | R | S | A | A | E | R | A | Q | L | K | I | Y | F | P | A | L | Y | T | R | - | - | - | - | - | - | - | - | T | P | K |
| W8E4 | - | - | - | - | A | Q | H | T | E | S | L | - | - | - | - | - | - | I | E | E | E | L | T | E | L | L | T | A | L | A | E | N | A | V | L | R | N | A | P | Q | R | S | - | - | - | - | - | - | - | - | - | - | - | - | - | - | - | - | P | N |
| G12G1 | - | - | - | - | - | - | - | - | - | - | - | - | - | - | - | - | - | Q | E | L | E | L | L | E | T | P | V | F | L | Q | M | F | K | D | C | A | N | - | - | - | - | - | - | - | - | - | - | - | - | - | - | - | - | - | - | - | - | - | - | - |
| S18E7 | - | - | - | - | - | - | - | - | - | - | - | - | - | - | - | - | - | L | N | V | N | L | E | R | T | S | Y | K | A | N | K | D | R | A | V | E | M | L | L | C | P | - | - | - | - | - | - | - | - | - | - | - | - | - | - | - | - | - | - | - |
| G21F11 | - | - | - | - | - | - | - | - | - | - | - | - | - | - | - | - | F | T | N | D | E | I | R | A | I | N | A | A | A | G | A | D | A | K | L | S | L | M | D | G | - | - | - | - | - | - | - | - | - | - | - | - | - | - | - | - | - | - | - | - |
| G7F4 | - | - | - | - | - | - | - | - | - | - | - | - | - | - | - | - | - | T | A | L | V | L | G | E | E | A | P | M | T | H | E | E | Y | K | D | A | I | L | S | - | - | - | - | - | - | - | - | - | - | - | - | - | - | - | - | - | - | - | - | - |
| G10H7 | - | - | - | - | - | - | - | - | - | - | - | - | - | - | - | V | A | P | R | R | T | L | V | S | F | S | N | L | P | R | M | N | K | S | L | Q | A | A | G | - | - | - | - | - | - | - | - | - | - | - | - | - | - | - | - | - | - | - | - | - |
| W7F8 | - | - | - | - | - | - | - | - | - | - | - | - | - | - | - | - | - | - | - | - | K | L | H | D | - | - | E | F | L | G | Q | A | K | F | L | A | S | - | - | - | - | - | - | - | - | - | - | - | - | - | - | - | - | - | - | - | - | - | - | - |
| W16A21 | - | - | - | - | - | - | - | - | - | - | - | - | - | - | - | E | D | L | K | W | A | L | S | L | S | D | F | T | P | N | E | I | I | S | L | R | G | A | K | N | - | - | - | - | - | - | - | - | - | - | - | - | - | - | - | - | - | - | - | - |
| W6C7 | - | - | - | - | - | - | - | - | - | - | - | - | - | - | - | - | - | H | E | Q | V | L | G | K | M | S | R | K | F | K | Q | D | V | V | H | L | F | K | H | - | - | - | - | - | - | - | - | - | - | - | - | - | - | - | - | - | - | - | - | - |
| G12H12 | - | - | - | - | - | - | - | - | - | - | - | - | - | - | - | - | - | L | G | R | R | V | P | D | T | D | L | P | T | - | - | - | E | Q | E | K | L | A | D | - | - | - | - | - | - | - | - | - | - | - | - | - | - | - | - | - | - | - | - | - |
| W1C2 | - | - | - | - | - | - | - | - | - | - | - | - | - | - | - | - | - | L | Q | F | N | V | P | E | I | A | A | P | L | N | A | M | A | Q | A | G | L | L | S | - | - | - | - | - | - | - | - | - | - | - | - | - | - | - | - | - | - | - | - | - |
|  |  |  |  |  |  |  |  |  |  |  |  |  |  |  |  |  |  |  |  |  |  |  |  |  |  |  |  |  |  |  |  |  |  |  |  |  |  |  |  |  |  |  |  |  |  |  |  |  |  |  |  |  |  |  |  |  |  |  |  |  |
| L5E4 | - | - | - | - | - | - | - | - | - | - | - | - | - | - | - | - | - | - | - | - | - | - | - | - | - | Q | Q | R | A | A | E | E | H | A | Q | R | E | A | N | A | P | S | P | F | T | L | T | R | A | A | R | E | H | K | K | P | P | P | F | T |
| W12F4 | - | - | - | - | - | - | - | - | - | - | - | - | - | - | - | - | - | - | - | - | L | A | Y | A | E | F | Q | A | A | Y | H | A | L | L | L | Q | Q | A | D | I | D | N | I | P | H | T | E | I | I | L | Q | A | V | P | L | T | Q | F | F | P |
| G2D9 | D | L | T | V | I | P | T | P | - | E | E | R | N | T | L | M | K | A | L | R | I | F | I | S | P | Q | K | N | D | F | K | R | I | L | M | D | T | S | V | C | K | E | T | D | F | D | Y | I | I | A | E | K | I | W | D | A | S | H | D | R |
| G3H12 | D | I | L | I | F | L | D | S | R | V | E | L | S | A | L | K | D | V | L | R | I | F | I | G | P | R | K | G | D | F | K | R | I | L | I | N | C | G | I | C | E | G | I | D | P | D | Y | E | M | A | E | K | I | W | D | A | P | L | Y | K |
| W8E4 | D | L | V | V | I | K | R | - | - | - | - | - | - | - | - | - | - | V | L | R | I | F | I | G | P | K | K | R | A | F | E | R | T | L | A | G | A | S | V | C | D | D | I | D | E | N | I | T | K | A | E | K | I | Y | D | A | P | L | S | R |
| G12G1 | - | - | - | - | - | - | - | - | - | - | - | - | - | - | - | - | - | - | - | - | V | Y | F | K | Y | D | T | N | W | F | K | D | I | L | V | R | A | N | I | C | K | R | K | T | A | F | V | R | I | P | G | I | I | D | K | A | K | F | V | P |
| S18E7 | - | - | - | - | - | - | - | - | - | - | - | - | - | - | - | - | - | - | - | - | - | - | - | K | K | R | E | Q | L | V | C | G | L | K | Q | A | H | L | C | P | P | A | M | K | P | T | D | N | F | I | R | N | V | V | E | N | P | I | F | A |
| G21F11 | - | - | - | - | - | - | - | - | - | - | - | - | - | - | - | - | - | - | - | - | - | - | - | - | R | L | R | H | L | A | T | E | I | L | S | D | L | G | F | P | I | L | G | R | Q | T | A | G | F | L | Y | P | A | L | A | E | V | L | L | S |
| G7F4 | - | - | - | - | - | - | - | - | - | - | - | - | - | - | - | - | - | - | - | - | - | F | H | A | F | D | Q | V | N | F | E | S | F | L | K | N | A | G | V | P | L | G | L | N | G | G | L | R | A | A | I | K | H | E | G | V | Y | N | Q | C |
| G10H7 | - | - | - | - | - | - | - | - | - | - | - | - | - | - | - | - | - | - | - | - | - | - | - | I | V | I | Q | G | S | L | E | D | S | I | N | G | E | T | D | E | R | K | L | M | A | A | L | R | H | F | V | D | H | Y | S | Y | S | T | S | P |
| W7F8 | - | - | - | - | - | - | - | - | - | - | - | - | - | - | - | - | - | - | - | - | Y | F | L | D | H | K | K | E | I | F | I | P | C | L | V | K | A | Q | L | C | L | R | I | T | P | V | S | N | I | K | L | N | I | D | K | A | E | A | V | P |
| W16A21 | - | - | - | - | - | - | - | - | - | - | - | - | - | - | - | I | E | K | V | I | E | I | F | E | I | H | F | R | I | F | C | Q | N | F | F | K | Y | H | Q | D | S | T | L | I | A | L | F | K | V | D | G | S | S | L | E | I | E | K | M | E |
| W6C7 | - | - | - | - | - | - | - | - | - | - | - | - | - | - | - | - | - | - | - | - | F | L | N | N | E | K | Y | K | L | V | M | S | L | V | K | N | G | M | I | P | K | A | T | N | D | D | K | A | I | K | K | A | A | Q | N | A | Y | E | T | N |
| G12H12 | - | - | - | - | - | - | - | - | - | - | - | - | - | - | - | - | - | - | - | - | A | E | Q | - | - | - | - | - | - | - | - | - | - | - | - | - | - | - | - | - | - | - | - | - | - | - | - | L | R | A | A | R | L | R | L | A | R | A | R | A |
| W1C2 | - | - | - | - | - | - | - | - | - | - | - | - | - | - | - | - | - | - | - | - | A | E | V | N | E | A | Q | E | A | F | D | R | L | E | A | R | V | P | D | T | D | E | P | E | N | G | T | L | R | S | A | R | L | R | L | A | R | A | R | A |
|  |  |  |  |  |  |  |  |  |  |  |  |  |  |  |  |  |  |  |  |  |  |  |  |  |  |  |  |  |  |  |  |  |  |  |  |  |  |  |  |  |  |  |  |  |  |  |  |  |  |  |  |  |  |  |  |  |  |  |  |  |
| L5E4 | L | T | R | G | G | R | G | Q | - | - | - | - | - | - | - | - | - | - | - |  |  |  |  |  |  |  |  |  |  |  |  |  |  |  |  |  |  |  |  |  |  |  |  |  |  |  |  |  |  |  |  |  |  |  |  |  |  |  |  |  |
| W12F4 | P | P | T | G | P | S | R | G | R | R | Q | - | - | - | - | - | - | - | - |  |  |  |  |  |  |  |  |  |  |  |  |  |  |  |  |  |  |  |  |  |  |  |  |  |  |  |  |  |  |  |  |  |  |  |  |  |  |  |  |  |
| G2D9 | L | K | D | A | L | R | E | A | S | S | R | G | I | G | I | S | Y | - | - |  |  |  |  |  |  |  |  |  |  |  |  |  |  |  |  |  |  |  |  |  |  |  |  |  |  |  |  |  |  |  |  |  |  |  |  |  |  |  |  |  |
| G3H12 | L | Q | N | A | F | I | T | A | H | Q | A | E | L | L | N | - | - | - | - |  |  |  |  |  |  |  |  |  |  |  |  |  |  |  |  |  |  |  |  |  |  |  |  |  |  |  |  |  |  |  |  |  |  |  |  |  |  |  |  |  |
| W8E4 | L | T | N | A | L | N | E | A | P | - | - | - | - | - | - | - | - | - | - |  |  |  |  |  |  |  |  |  |  |  |  |  |  |  |  |  |  |  |  |  |  |  |  |  |  |  |  |  |  |  |  |  |  |  |  |  |  |  |  |  |
| G12G1 | L | L | N | A | L | K | L | A | Y | S | E | R | A | T | R | N | N | - | - |  |  |  |  |  |  |  |  |  |  |  |  |  |  |  |  |  |  |  |  |  |  |  |  |  |  |  |  |  |  |  |  |  |  |  |  |  |  |  |  |  |
| S18E7 | G | F | V | A | F | A | L | K | K | A | S | D | - | - | - | - | - | - | - |  |  |  |  |  |  |  |  |  |  |  |  |  |  |  |  |  |  |  |  |  |  |  |  |  |  |  |  |  |  |  |  |  |  |  |  |  |  |  |  |  |
| G21F11 | A | P | S | T | E | S | I | D | H | H | - | - | - | - | - | - | - | - | - |  |  |  |  |  |  |  |  |  |  |  |  |  |  |  |  |  |  |  |  |  |  |  |  |  |  |  |  |  |  |  |  |  |  |  |  |  |  |  |  |  |
| G7F4 | L | A | A | L | K | K | Y | V | S | E | E | G | T | S | T | S | - | - | - |  |  |  |  |  |  |  |  |  |  |  |  |  |  |  |  |  |  |  |  |  |  |  |  |  |  |  |  |  |  |  |  |  |  |  |  |  |  |  |  |  |
| G10H7 | P | R | D | G | A | S | T | S | K | R | R | - | - | - | - | - | - | - | - |  |  |  |  |  |  |  |  |  |  |  |  |  |  |  |  |  |  |  |  |  |  |  |  |  |  |  |  |  |  |  |  |  |  |  |  |  |  |  |  |  |
| W7F8 | L | A | K | A | L | S | E | L | V | K | T | I | G | N | - | - | - | - | - |  |  |  |  |  |  |  |  |  |  |  |  |  |  |  |  |  |  |  |  |  |  |  |  |  |  |  |  |  |  |  |  |  |  |  |  |  |  |  |  |  |
| W16A21 | L | P | R | L | V | K | I | V | H | E | V | M | L | R | E | Y | N | T | K |  |  |  |  |  |  |  |  |  |  |  |  |  |  |  |  |  |  |  |  |  |  |  |  |  |  |  |  |  |  |  |  |  |  |  |  |  |  |  |  |  |
| W6C7 | I | L | K | L | I | K | A | L | V | D | V | P | D | M | K | - | - | - | - |  |  |  |  |  |  |  |  |  |  |  |  |  |  |  |  |  |  |  |  |  |  |  |  |  |  |  |  |  |  |  |  |  |  |  |  |  |  |  |  |  |
| G12H12 | A | L | N | L | P | I | L | D | F | D | T | R | R | R | - | - | - | - | - |  |  |  |  |  |  |  |  |  |  |  |  |  |  |  |  |  |  |  |  |  |  |  |  |  |  |  |  |  |  |  |  |  |  |  |  |  |  |  |  |  |
| W1C2 | A | L | N | R | - | F | T | P | F | Q | - | - | - | - | - | - | - | - | - |  |  |  |  |  |  |  |  |  |  |  |  |  |  |  |  |  |  |  |  |  |  |  |  |  |  |  |  |  |  |  |  |  |  |  |  |  |  |  |  |  |
|  |  |  |  |  |  |  |  |  |  |  |  |  |  |  |  |  |  |  |  |  |  |  |  |  |  |  |  |  |  |  |  |  |  |  |  |  |  |  |  |  |  |  |  |  |  |  |  |  |  |  |  |  |  |  |  |  |  |  |  |  |

**B**

**├ 5’-UTR ├ SPCR**

| G12C4 |  | - | - | - | - | A | T | T | C | T | C | T | C | C | T | G | A | A | A | A | A | - | G | A | A | A | C | A | A | G | C | T | C | T | A | C | A | A | C | G | **A** | **T** | **G** | G | C | A | A | A | T | A | A | A | A | T | T | T | T | T | G | T | T |
| --- | --- | --- | --- | --- | --- | --- | --- | --- | --- | --- | --- | --- | --- | --- | --- | --- | --- | --- | --- | --- | --- | --- | --- | --- | --- | --- | --- | --- | --- | --- | --- | --- | --- | --- | --- | --- | --- | --- | --- | --- | --- | --- | --- | --- | --- | --- | --- | --- | --- | --- | --- | --- | --- | --- | --- | --- | --- | --- | --- | --- | --- |
| G21F1 |  | - | - | - | T | A | T | T | C | T | C | T | C | C | T | G | A | A | A | A | A | A | G | A | A | A | C | A | A | G | C | T | C | T | A | C | A | A | C | G | **A** | **T** | **G** | G | C | A | A | A | T | A | A | A | A | T | T | T | T | T | G | T | T |
| G8B10 |  | G | A | A | T | A | T | T | C | T | C | G | A | C | T | G | A | A | A | A | A | - | - | - | A | A | C | A | C | G | C | T | C | T | A | T | A | A | C | G | **A** | **T** | **G** | G | C | A | A | A | T | A | A | A | T | T | C | T | T | T | G | T | T |
| G15B4 |  | - | - | - | - | - | T | T | C | T | C | G | A | C | T | G | A | A | T | A | A | - | - | - | A | A | C | A | C | G | C | T | C | T | A | T | A | A | C | G | **A** | **T** | **G** | G | C | A | A | A | T | A | A | A | T | T | T | T | T | T | G | T | T |

**├ MPCR**

| G12C4 |  | T | T | T | T | T | G | G | C | A | T | T | C | G | C | C | G | C | A | T | T | G | G | C | C | T | A | C | G | T | G | G | C | C | A | C | G | G | C | G | A | T | G | A | T | G | G | - | - | - | - | - | A | A | C | C | A | - | A | A | T |
| --- | --- | --- | --- | --- | --- | --- | --- | --- | --- | --- | --- | --- | --- | --- | --- | --- | --- | --- | --- | --- | --- | --- | --- | --- | --- | --- | --- | --- | --- | --- | --- | --- | --- | --- | --- | --- | --- | --- | --- | --- | --- | --- | --- | --- | --- | --- | --- | --- | --- | --- | --- | --- | --- | --- | --- | --- | --- | --- | --- | --- | --- |
| G21F1 |  | T | T | T | C | T | G | G | C | A | T | T | C | G | C | C | G | C | A | T | T | A | G | C | C | T | A | C | G | T | G | G | C | C | A | C | G | G | C | A | A | T | G | A | T | G | G | G | C | G | C | A | A | A | T | C | A | T | A | A | T |
| G8B10 |  | T | T | T | C | T | G | G | C | A | T | T | T | G | C | C | G | C | A | T | T | A | G | T | G | G | C | C | A | G | T | G | - | - | - | C | A | T | G | T | G | G | G | G | C | A | C | C | T | A | A | A | A | T | A | C | A | T | C | C | T |
| G15B4 |  | T | T | T | C | T | G | G | C | A | T | T | T | G | C | C | G | C | A | T | T | A | G | T | G | G | C | C | A | G | C | G | G | C | G | C | A | T | G | T | G | G | G | G | G | A | C | C | T | A | - | - | A | T | C | C | A | G | A | A | T |

| G12C4 |  | A | A | G | C | C | C | A | A | A | C | C | T | T | C | T | A | T | A | T | C | T | G | C | C | A | A | A | C | C | T | G | A | A | G | C | G | A | G | T | A | C | A | C | G | A | A | A | A | G | A | A | A | G | A | G | A | A | G | C | C |
| --- | --- | --- | --- | --- | --- | --- | --- | --- | --- | --- | --- | --- | --- | --- | --- | --- | --- | --- | --- | --- | --- | --- | --- | --- | --- | --- | --- | --- | --- | --- | --- | --- | --- | --- | --- | --- | --- | --- | --- | --- | --- | --- | --- | --- | --- | --- | --- | --- | --- | --- | --- | --- | --- | --- | --- | --- | --- | --- | --- | --- | --- |
| G21F1 |  | A | A | G | C | A | G | A | A | A | G | C | T | A | C | T | G | T | A | G | C | T | C | C | C | A | T | A | C | C | T | C | A | G | C | C | G | A | G | G | T | C | A | T | T | A | A | G | A | G | A | A | A | A | C | G | A | A | G | C | C |
| G8B10 |  | T | C | G | G | A | A | A | C | A | A | C | T | C | A | A | A | C | A | G | C | T | C | C | G | C | A | A | C | C | A | G | G | A | C | A | A | A | T | G | A | C | T | A | C | C | G | A | A | C | A | C | C | A | C | C | A | C | A | A | T |
| G15B4 |  | T | G | G | G | A | A | A | - | - | - | - | - | - | A | C | A | T | G | G | C | G | G | G | G | G | A | A | A | T | G | C | T | G | G | T | G | C | A | G | C | C | A | C | C | C | A | A | A | T | G | C | C | A | C | C | C | A | A | A | G |

| G12C4 |  | G | C | A | T | C | G | A | C | C | G | T | G | G | A | A | A | C | C | - | A | A | C | A | A | T | G | C | C | A | G | T | C | C | A | G | C | A | A | - | - | - | - | - | - | A | A | G | G | A | A | A | G | G | A | A | A | G | T | G | G |
| --- | --- | --- | --- | --- | --- | --- | --- | --- | --- | --- | --- | --- | --- | --- | --- | --- | --- | --- | --- | --- | --- | --- | --- | --- | --- | --- | --- | --- | --- | --- | --- | --- | --- | --- | --- | --- | --- | --- | --- | --- | --- | --- | --- | --- | --- | --- | --- | --- | --- | --- | --- | --- | --- | --- | --- | --- | --- | --- | --- | --- | --- |
| G21F1 |  | G | C | A | T | C | G | A | C | C | A | T | G | G | A | A | A | C | C | - | A | A | C | A | A | T | G | C | C | A | G | T | C | C | A | G | C | A | G | - | - | - | - | - | - | A | A | G | - | A | A | C | G | C | A | A | A | T | T | G | G |
| G8B10 |  | C | C | A | C | C | A | A | C | T | G | T | T | G | A | A | G | C | A | G | A | A | A | A | G | T | G | T | T | G | C | T | C | C | T | G | T | T | T | T | T | G | T | A | A | A | G | G | T | T | G | T | G | G | A | A | A | A | T | G | T |
| G15B4 |  | A | A | G | C | T | T | G | G | A | A | A | C | C | A | A | C | T | G | A | A | A | G | A | G | C | T | C | C | G | C | A | C | C | C | A | A | A | T | C | - | - | - | - | - | A | A | A | T | G | A | C | A | A | A | A | T | T | T | G | A |

| G12C4 |  | T | G | G | A | A | G | T | T | G | T | C | T | T | G | G | G | G | G | A | T | G | - | - | - | - | - | - | - | - | - | C | T | T | C | A | A | G | G | G | T | A | T | - | - | - | - | - | A | T | T | A | C | A | T | C | A | T | A | A | A |
| --- | --- | --- | --- | --- | --- | --- | --- | --- | --- | --- | --- | --- | --- | --- | --- | --- | --- | --- | --- | --- | --- | --- | --- | --- | --- | --- | --- | --- | --- | --- | --- | --- | --- | --- | --- | --- | --- | --- | --- | --- | --- | --- | --- | --- | --- | --- | --- | --- | --- | --- | --- | --- | --- | --- | --- | --- | --- | --- | --- | --- | --- |
| G21F1 |  | - | - | - | - | - | A | T | T | G | T | T | T | C | G | G | G | C | A | A | T | G | - | - | - | - | - | - | - | - | - | C | A | T | A | A | A | G | G | A | T | T | T | - | - | - | - | - | T | A | T | A | A | C | T | C | C | T | A | G | A |
| G8B10 |  | T | G | - | C | A | C | C | G | G | T | T | T | A | G | G | A | A | A | A | T | G | T | T | G | C | G | A | C | A | G | T | T | T | A | G | G | A | A | A | T | T | G | T | T | G | C | G | C | A | A | A | C | T | G | T | T | G | C | C | A |
| G15B4 |  | T | - | - | C | A | T | C | G | G | - | - | - | - | G | G | A | C | C | A | T | - | - | - | - | C | G | T | C | A | T | C | T | T | G | T | G | A | A | A | A | T | A | - | - | A | G | C | C | A | G | G | T | T | G | T | T | T | A | G | A |

| G12C4 |  | A | A | G | T | C | A | A | C | A | G | A | A | A | G | A | C | C | A | C | A | T | G | G | T | A | C | - | - | - | - | - | - | G | G | A | - | A | G | G | A | A | G | T | G | A | A | G | - | - | G | C | G | A | - | T | T | T | G | T | C |
| --- | --- | --- | --- | --- | --- | --- | --- | --- | --- | --- | --- | --- | --- | --- | --- | --- | --- | --- | --- | --- | --- | --- | --- | --- | --- | --- | --- | --- | --- | --- | --- | --- | --- | --- | --- | --- | --- | --- | --- | --- | --- | --- | --- | --- | --- | --- | --- | --- | --- | --- | --- | --- | --- | --- | --- | --- | --- | --- | --- | --- | --- |
| G21F1 |  | A | A | - | - | - | - | - | - | - | - | - | - | - | - | - | - | - | - | - | - | - | - | - | T | A | G | - | - | - | - | - | - | G | T | G | - | A | G | A | A | A | G | T | C | A | A | G | - | - | G | C | G | A | G | T | T | T | G | T | C |
| G8B10 |  | C | A | A | T | T | G | T | T | G | C | T | G | T | T | G | T | G | G | A | G | A | T | C | C | A | G | A | A | C | G | T | T | G | T | G | T | A | G | C | A | T | G | T | T | G | T | G | T | A | G | C | A | G | G | C | T | G | T | A | T |
| G15B4 |  | A | A | A | A | T | C | T | T | G | C | T | G | C | T | G | T | - | - | - | - | - | - | - | - | - | - | - | - | - | - | - | T | G | T | G | T | A | A | A | A | T | G | C | T | G | T | G | - | - | - | C | A | A | A | A | T | T | T | C | C |

| G12C4 |  | A | T | G | G | C | T | C | A | A | A | G | - | A | T | C | C | G | G | T | G | C | A | A | G | C | T | A | A | T | T | A | T | A | A | T | A | G | A | C | G | G | C | G | C | - | - | - | - | - | - | - | - | - | **T** | **A** | **G** | A | A | T | A |
| --- | --- | --- | --- | --- | --- | --- | --- | --- | --- | --- | --- | --- | --- | --- | --- | --- | --- | --- | --- | --- | --- | --- | --- | --- | --- | --- | --- | --- | --- | --- | --- | --- | --- | --- | --- | --- | --- | --- | --- | --- | --- | --- | --- | --- | --- | --- | --- | --- | --- | --- | --- | --- | --- | --- | --- | --- | --- | --- | --- | --- | --- |
| G21F1 |  | A | C | G | G | C | T | C | C | A | A | G | - | A | T | C | C | G | G | T | G | C | T | C | A | A | T | A | A | T | T | A | T | A | A | T | A | G | A | C | G | G | C | A | C | - | - | - | - | - | - | - | - | - | **T** | **A** | **G** | A | A | T | A |
| G8B10 |  | A | T | G | T | T | T | T | T | G | G | G | G | A | G | T | A | G | G | T | A | C | C | G | T | T | G | T | T | G | C | T | T | C | A | T | G | C | C | T | T | A | A | T | C | A | A | T | A | A | T | T | T | G | A | A | G | T | G | T | T |
| G15B4 |  | A | G | A | A | C | T | T | T | G | T | G | T | A | T | T | A | T | G | T | G | G | T | G | T | T | G | G | A | G | T | - | - | - | A | T | G | T | - | - | - | - | - | - | - | - | - | - | - | - | - | - | - | - | A | A | G | T | G | C | G |

**├** 3’-UTR

| G12C4 |  | G | A | G | T | A | C | T | A | T | A | T | T | T | T | C | G | C | C | T | T | T | T | A | A | - | - | T | T | T | A | T | T | A | A | C | A | C | C | C | A | G | A | A | A | T | T | G | T | T | G | A | T | T | G | C | G | A | A | A | A |
| --- | --- | --- | --- | --- | --- | --- | --- | --- | --- | --- | --- | --- | --- | --- | --- | --- | --- | --- | --- | --- | --- | --- | --- | --- | --- | --- | --- | --- | --- | --- | --- | --- | --- | --- | --- | --- | --- | --- | --- | --- | --- | --- | --- | --- | --- | --- | --- | --- | --- | --- | --- | --- | --- | --- | --- | --- | --- | --- | --- | --- | --- |
| G21F1 |  | G | A | A | T | A | C | T | A | T | A | T | T | T | T | C | G | C | C | T | T | T | T | A | A | - | - | T | T | T | A | T | T | A | A | C | A | C | C | C | A | G | A | A | A | T | T | G | T | T | G | A | T | T | G | T | G | A | A | A | A |
| G8B10 |  | C | C | A | G | A | A | T | T | T | A | C | T | G | T | T | A | A | T | A | A | C | A | A | A | G | C | C | **T** | **A** | **A** | A | T | G | T | T | T | C | G | C | A | G | A | A | A | T | T | A | T | T | G | A | T | T | G | T | G | A | A | A | A |
| G15B4 |  | T | - | A | C | A | A | T | G | T | - | C | T | A | T | - | - | G | C | G | A | A | G | A | A | G | C | C | **T** | **A** | **A** | A | T | G | T | T | T | C | G | C | A | G | A | A | A | T | T | A | T | T | G | A | T | T | G | T | G | A | A | A | A |

| G12C4 |  | T | A | T | T | G | A | A | G | A | T | T | T | T | A | T | C | G | A | A | A | T | A | A | A | A | T | C | A | C | T | A | T | A | G | T | C | T | G | A | A | A | A | A | A | T | T | C | A | T | T | T | T | T | T | C | T | C | A | T | G |
| --- | --- | --- | --- | --- | --- | --- | --- | --- | --- | --- | --- | --- | --- | --- | --- | --- | --- | --- | --- | --- | --- | --- | --- | --- | --- | --- | --- | --- | --- | --- | --- | --- | --- | --- | --- | --- | --- | --- | --- | --- | --- | --- | --- | --- | --- | --- | --- | --- | --- | --- | --- | --- | --- | --- | --- | --- | --- | --- | --- | --- | --- |
| G21F1 |  | T | A | T | T | G | A | G | G | A | T | T | T | T | A | T | C | G | A | A | A | T | A | A | A | T | T | C | A | C | T | A | T | A | G | T | C | T | G | A | A | A | A | A | A | T | T | C | A | T | T | T | T | T | T | C | T | C | A | T | G |
| G8B10 |  | T | A | T | T | G | T | G | G | A | T | T | T | T | A | T | C | G | A | A | A | T | A | A | A | A | T | T | A | C | T | G | T | A | T | T | C | C | G | - | - | - | - | - | - | - | - | - | - | - | - | - | - | - | - | - | - | - | - | - | - |
| G15B4 |  | T | A | T | T | G | T | G | G | A | T | T | T | T | A | T | C | G | A | A | A | T | A | A | A | A | T | T | A | C | T | G | G | A | T | - | - | - | - | - | - | - | - | - | - | - | - | - | - | - | - | - | - | - | - | - | - | - | - | - | - |

| G12C4 |  | A | C | T | A | A | T | G | C | T | C | A | T | T | G | C | T | G | C | A | T | C | A |  |  |  |  |  |  |  |  |  |  |  |  |  |  |  |  |  |  |  |  |  |  |  |  |  |  |  |  |  |  |  |  |  |  |  |  |  |  |
| --- | --- | --- | --- | --- | --- | --- | --- | --- | --- | --- | --- | --- | --- | --- | --- | --- | --- | --- | --- | --- | --- | --- | --- | --- | --- | --- | --- | --- | --- | --- | --- | --- | --- | --- | --- | --- | --- | --- | --- | --- | --- | --- | --- | --- | --- | --- | --- | --- | --- | --- | --- | --- | --- | --- | --- | --- | --- | --- | --- | --- | --- |
| G21F1 |  | A | C | T | A | A | T | G | C | T | C | G | T | T | G | C | T | A | C | A | C | C | - |  |  |  |  |  |  |  |  |  |  |  |  |  |  |  |  |  |  |  |  |  |  |  |  |  |  |  |  |  |  |  |  |  |  |  |  |  |  |
| G8B10 |  | - | - | - | - | - | - | - | - | - | - | - | - | - | - | - | - | - | - | - | - | - | - |  |  |  |  |  |  |  |  |  |  |  |  |  |  |  |  |  |  |  |  |  |  |  |  |  |  |  |  |  |  |  |  |  |  |  |  |  |  |
| G15B4 |  | - | - | - | - | - | - | - | - | - | - | - | - | - | - | - | - | - | - | - | - | - | - |  |  |  |  |  |  |  |  |  |  |  |  |  |  |  |  |  |  |  |  |  |  |  |  |  |  |  |  |  |  |  |  |  |  |  |  |  |  |

**Protein alignment**

| G15B4 | M | A | N | K | F | F | V | F | L | A | F | A | A | L | V | A | S | G | A | C | G | G | P | N | P | E | L | G | K | H | G | G | G | N | A | G | A | A | T | Q | M | P | P | K | E | A | W | K | P | T | E | R | A | P | H | P | N | Q | M | T |
| --- | --- | --- | --- | --- | --- | --- | --- | --- | --- | --- | --- | --- | --- | --- | --- | --- | --- | --- | --- | --- | --- | --- | --- | --- | --- | --- | --- | --- | --- | --- | --- | --- | --- | --- | --- | --- | --- | --- | --- | --- | --- | --- | --- | --- | --- | --- | --- | --- | --- | --- | --- | --- | --- | --- | --- | --- | --- | --- | --- | --- |
| G8B10 | M | A | N | K | F | F | V | F | L | A | F | A | A | L | V | A | S | - | A | C | G | A | P | K | I | H | P | S | E | T | T | - | Q | T | A | P | Q | P | G | Q | M | T | T | E | H | H | H | N | P | P | T | - | - | V | E | A | E | K | C | C |
| G21F1 | M | A | N | K | I | F | V | F | L | A | F | A | A | L | A | Y | V | - | A | T | A | M | M | G | A | N | H | N | K | Q | K | A | T | V | A | P | I | P | Q | P | R | S | L | R | E | N | E | A | A | S | T | - | - | M | E | T | N | N | A | S |
| G12C4 | M | A | N | K | I | F | V | F | L | A | F | A | A | L | A | Y | V | - | A | T | A | M | M | E | P | - | - | N | K | P | K | P | S | I | S | A | K | P | E | A | S | T | R | K | E | R | E | A | A | S | T | - | - | V | E | T | N | N | A | S |
|  |  |  |  |  |  |  |  |  |  |  |  |  |  |  |  |  |  |  |  |  |  |  |  |  |  |  |  |  |  |  |  |  |  |  |  |  |  |  |  |  |  |  |  |  |  |  |  |  |  |  |  |  |  |  |  |  |  |  |  |  |
| G15B4 | K | F | D | H | R | G | P | S | S | S | C | E | N | K | P | G | C | L | E | K | S | C | C | C | C | V | K | C | C | A | K | F | - | - | - | - | - | P | E | L | C | V | L | C | G | V | G | V | C | K | C | - | - | - | - | - | - | V | Q | C |
| G8B10 | S | C | F | C | K | G | C | G | K | C | C | T | G | L | G | K | C | C | D | S | L | G | N | C | C | A | N | C | C | H | N | C | C | C | C | G | D | P | E | R | C | V | A | C | C | V | A | G | C | I | C | F | W | G | V | G | T | V | V | A |
| G21F1 | P | A | E | - | - | E | R | K | L | D | C | F | G | - | - | Q | C | I | K | D | F | I | T | P | R | - | - | - | - | - | - | - | - | - | - | - | - | - | - | - | - | - | - | - | - | - | - | - | - | - | - | - | - | - | - | - | - | - | - | - |
| G12C4 | P | A | K | G | K | E | S | G | G | S | C | L | G | - | - | G | C | F | K | G | I | L | H | H | K | K | S | T | E | R | P | H | - | - | - | - | - | G | T | E | G | S | E | G | D | L | S | W | L | K | D | - | - | - | - | - | P | V | Q | A |
|  |  |  |  |  |  |  |  |  |  |  |  |  |  |  |  |  |  |  |  |  |  |  |  |  |  |  |  |  |  |  |  |  |  |  |  |  |  |  |  |  |  |  |  |  |  |  |  |  |  |  |  |  |  |  |  |  |  |  |  |  |
| G15B4 | L | C | E | E | A | - |  |  |  |  |  |  |  |  |  |  |  |  |  |  |  |  |  |  |  |  |  |  |  |  |  |  |  |  |  |  |  |  |  |  |  |  |  |  |  |  |  |  |  |  |  |  |  |  |  |  |  |  |  |  |
| G8B10 | S | C | L | N | Q | - |  |  |  |  |  |  |  |  |  |  |  |  |  |  |  |  |  |  |  |  |  |  |  |  |  |  |  |  |  |  |  |  |  |  |  |  |  |  |  |  |  |  |  |  |  |  |  |  |  |  |  |  |  |  |
| G21F1 | - | - | N | R | - | - |  |  |  |  |  |  |  |  |  |  |  |  |  |  |  |  |  |  |  |  |  |  |  |  |  |  |  |  |  |  |  |  |  |  |  |  |  |  |  |  |  |  |  |  |  |  |  |  |  |  |  |  |  |  |
| G12C4 | N | Y | N | R | R | R |  |  |  |  |  |  |  |  |  |  |  |  |  |  |  |  |  |  |  |  |  |  |  |  |  |  |  |  |  |  |  |  |  |  |  |  |  |  |  |  |  |  |  |  |  |  |  |  |  |  |  |  |  |  |

**C**

**├ 5’-UTR ├ SPCR**

| G15B9 | - | - | - | T | T | C | A | A | A | A | C | T | T | G | A | A | T | A | T | T | A | A | T | A | A | G | T | G | A | A | G | T | G | A | A | G | A | A | T | T | G | T | T | G | C | A | A | A | **A** | **T** | **G** | T | T | T | A | A | C | T | C | T |
| --- | --- | --- | --- | --- | --- | --- | --- | --- | --- | --- | --- | --- | --- | --- | --- | --- | --- | --- | --- | --- | --- | --- | --- | --- | --- | --- | --- | --- | --- | --- | --- | --- | --- | --- | --- | --- | --- | --- | --- | --- | --- | --- | --- | --- | --- | --- | --- | --- | --- | --- | --- | --- | --- | --- | --- | --- | --- | --- | --- | --- |
| G9A12 | - | - | - | - | - | - | A | A | A | A | C | T | T | G | A | A | T | A | C | T | A | A | T | A | A | G | T | G | A | A | G | T | G | A | A | G | A | A | T | T | A | T | T | G | C | A | A | A | **A** | **T** | **G** | T | T | G | A | A | C | T | C | T |
| G11C8 | - | - | - | - | - | - | A | A | A | A | C | T | T | G | A | A | A | A | C | T | A | A | T | A | A | G | T | G | A | A | G | - | - | - | - | - | A | A | T | T | G | T | T | G | C | A | A | A | **A** | **T** | **G** | T | T | T | A | A | C | T | C | T |
| G28G10 | - | - | - | T | T | C | A | A | A | A | C | T | T | G | A | A | T | A | C | T | A | A | T | A | A | G | T | G | A | A | G | T | G | A | A | G | A | A | T | T | G | T | T | G | C | A | A | A | **A** | **T** | **G** | T | T | T | A | A | C | T | C | T |
| S19E7 | - | G | A | T | T | C | A | A | A | A | C | T | T | G | A | A | T | A | C | T | A | A | T | A | A | G | T | G | A | A | G | T | G | A | A | G | A | A | T | T | G | T | T | G | C | A | A | A | **A** | **T** | **G** | T | T | T | A | A | C | T | C | T |
| G6D3 | - | - | - | T | T | C | A | A | A | A | C | T | T | G | A | A | A | A | C | T | A | A | T | A | A | G | T | G | A | A | G | - | - | - | - | - | A | A | T | T | A | T | T | G | C | A | A | A | **A** | **T** | **G** | T | T | A | A | A | C | T | C | T |
| S10A9 | G | G | A | T | T | C | A | A | A | A | C | T | T | G | A | A | T | A | C | T | A | A | T | A | A | G | T | A | A | A | G | - | - | - | - | - | A | A | T | T | T | T | T | G | C | A | A | A | **A** | **T** | **G** | T | T | A | A | A | T | T | C | T |

**├MPCR**

| G15B9 | C | A | A | A | A | G | T | T | G | A | T | C | A | T | T | T | G | T | T | G | T | C | T | G | T | T | A | T | T | T | G | C | T | G | T | C | G | T | A | T | T | G | T | T | G | C | A | A | T | C | A | T | T | G | G | A | A | G | C | C |
| --- | --- | --- | --- | --- | --- | --- | --- | --- | --- | --- | --- | --- | --- | --- | --- | --- | --- | --- | --- | --- | --- | --- | --- | --- | --- | --- | --- | --- | --- | --- | --- | --- | --- | --- | --- | --- | --- | --- | --- | --- | --- | --- | --- | --- | --- | --- | --- | --- | --- | --- | --- | --- | --- | --- | --- | --- | --- | --- | --- | --- |
| G9A12 | C | A | A | A | A | G | T | T | G | A | T | C | A | T | C | T | G | T | T | G | T | C | T | A | T | T | A | T | T | T | G | C | T | G | C | C | G | T | A | T | T | G | T | T | G | C | A | A | T | C | A | T | T | A | A | A | A | G | C | C |
| G11C8 | C | A | A | A | A | G | T | T | G | A | T | C | A | T | T | T | T | T | A | G | T | C | T | G | T | T | A | T | T | T | G | C | T | G | C | C | G | T | A | T | G | G | G | T | G | C | A | A | T | T | T | T | C | A | A | A | A | G | C | C |
| G28G10 | C | A | A | A | A | G | T | T | A | A | G | C | A | T | T | T | G | T | T | G | T | C | T | G | T | T | A | T | T | T | G | C | T | G | C | C | G | T | A | T | G | G | T | T | G | C | A | A | T | C | A | T | T | A | A | A | A | G | C | C |
| S19E7 | C | A | A | A | A | G | T | T | G | A | T | C | A | T | T | T | G | T | T | G | T | C | T | G | T | T | A | T | T | T | G | C | T | G | C | C | G | T | A | T | G | G | G | T | G | C | A | G | T | C | A | T | T | A | A | A | A | G | C | C |
| G6D3 | C | A | A | A | G | C | T | T | G | A | T | C | G | T | T | T | G | T | T | G | T | T | T | G | T | T | A | T | T | T | G | C | T | A | C | T | G | T | A | T | G | G | G | C | A | A | C | A | G | A | A | G | - | A | G | C | A | G | C | C |
| S10A9 | C | A | A | A | A | G | T | T | A | A | T | C | A | T | T | T | G | T | T | G | T | T | T | G | G | T | A | T | T | T | G | C | T | G | C | C | A | T | A | T | G | G | G | T | G | C | A | A | T | C | A | T | T | A | A | A | A | G | C | C |

| G15B9 | G | G | A | T | C | A | A | A | - | - | - | - | - | - | - | - | - | - | - | - | - | - | - | - | - | - | - | - | - | - | - | - | - | - | - | - | - | - | - | - | - | - | - | - | - | - | - | - | - | - | - | - | - | - | - | - | - | - | - | - |
| --- | --- | --- | --- | --- | --- | --- | --- | --- | --- | --- | --- | --- | --- | --- | --- | --- | --- | --- | --- | --- | --- | --- | --- | --- | --- | --- | --- | --- | --- | --- | --- | --- | --- | --- | --- | --- | --- | --- | --- | --- | --- | --- | --- | --- | --- | --- | --- | --- | --- | --- | --- | --- | --- | --- | --- | --- | --- | --- | --- | --- |
| G9A12 | A | C | A | C | C | A | A | A | - | - | - | - | - | - | - | - | - | - | - | - | - | - | - | - | - | - | - | - | - | - | - | - | - | - | - | - | - | - | - | - | - | - | - | - | - | - | - | - | - | - | - | - | - | - | - | - | - | - | - | - |
| G11C8 | G | C | A | C | C | A | A | A | - | - | - | - | - | - | - | - | - | - | - | - | - | - | - | - | - | - | - | - | - | - | - | - | - | - | - | - | - | - | - | - | - | - | - | - | - | - | - | - | - | - | - | - | - | - | - | - | - | - | - | - |
| G28G10 | A | C | A | C | C | A | A | C | G | G | G | C | A | A | T | G | G | T | G | A | A | G | A | C | G | C | A | T | C | C | C | A | G | C | C | A | T | T | A | C | T | A | G | G | T | C | G | A | T | T | A | G | C | A | T | A | C | G | A | A |
| S19E7 | G | C | A | C | T | A | A | C | - | - | - | - | - | - | - | - | - | - | - | - | - | - | - | - | - | - | - | - | - | - | - | - | - | - | - | - | - | - | - | - | - | - | - | - | - | - | - | - | - | - | - | - | - | - | - | - | - | - | A | C |
| G6D3 | A | C | C | A | G | C | G | C | A | A | A | A | A | A | - | - | - | - | - | - | - | - | - | - | - | - | - | - | - | - | - | - | - | - | - | - | - | - | - | - | - | - | - | - | - | - | - | - | - | - | - | - | - | - | - | - | - | A | A | A |
| S10A9 | G | T | A | C | A | A | A | T | G | A | G | C | A | A | - | - | - | - | - | - | - | - | - | - | - | - | - | - | - | - | - | - | - | - | - | - | - | - | - | - | - | - | - | - | - | - | - | - | - | - | - | - | - | - | - | - | - | G | C | A |

| G15B9 | - | - | - | - | - | - | - | - | C | - | - | - | - | - | - | - | - | - | - | - | - | - | - | - | - | - | - | - | - | - | - | - | - | - | - | - | - | - | - | - | - | - | - | - | - | - | - | - | - | - | - | - | - | - | - | - | - | G | G | A |
| --- | --- | --- | --- | --- | --- | --- | --- | --- | --- | --- | --- | --- | --- | --- | --- | --- | --- | --- | --- | --- | --- | --- | --- | --- | --- | --- | --- | --- | --- | --- | --- | --- | --- | --- | --- | --- | --- | --- | --- | --- | --- | --- | --- | --- | --- | --- | --- | --- | --- | --- | --- | --- | --- | --- | --- | --- | --- | --- | --- | --- |
| G9A12 | - | - | - | - | - | - | - | - | A | A | G | A | A | A | - | - | - | - | - | - | - | - | - | - | - | - | - | - | - | - | - | - | - | - | - | - | - | - | - | - | - | - | - | - | - | - | - | - | - | - | - | - | - | - | - | - | G | G | G | A |
| G11C8 | - | - | - | - | - | - | - | - | A | A | G | A | A | G | - | - | - | - | - | - | - | - | - | - | - | - | - | - | - | - | - | - | - | - | - | - | - | - | - | - | - | - | - | - | - | - | - | - | - | - | - | - | - | - | - | - | A | C | C | T |
| G28G10 | C | A | G | G | T | G | T | A | C | C | A | A | C | A | C | G | C | A | C | C | A | C | A | A | C | C | A | C | C | A | T | C | A | C | C | A | C | C | A | C | A | A | C | C | A | C | C | A | C | T | A | T | C | T | T | A | C | A | G | T |
| S19E7 | C | A | G | G | T | T | T | A | T | C | G | A | T | A | - | - | - | - | - | - | - | - | - | - | - | - | - | - | - | - | - | - | - | - | - | - | - | - | - | - | - | - | - | - | - | - | - | - | - | T | A | T | C | T | - | - | C | G | A | T |
| G6D3 | T | A | G | A | G | T | C | A | C | A | G | G | G | G | C | T | G | G | A | A | - | - | - | - | - | - | - | - | - | - | - | - | - | - | - | - | - | - | - | - | - | - | - | - | - | - | - | - | - | - | - | - | T | T | T | T | T | C | G | A |
| S10A9 | T | T | A | T | G | A | T | G | A | A | G | A | A | A | T | C | G | C | C | - | - | - | - | - | - | - | - | - | - | - | - | - | - | - | - | - | - | - | - | - | - | - | - | - | - | - | - | - | - | - | - | - | C | A | T | T | T | T | G | A |

| G15B9 | A | A | G | G | A | A | A | T | T | G | A | T | C | T | C | A | A | T | T | T | A | T | C | C | A | T | A | G | C | C | C | A | A | C | C | A | G | G | - | - | - | - | - | - | - | - | - | - | - | - | - | - | - | - | - | - | - | C | T | C |
| --- | --- | --- | --- | --- | --- | --- | --- | --- | --- | --- | --- | --- | --- | --- | --- | --- | --- | --- | --- | --- | --- | --- | --- | --- | --- | --- | --- | --- | --- | --- | --- | --- | --- | --- | --- | --- | --- | --- | --- | --- | --- | --- | --- | --- | --- | --- | --- | --- | --- | --- | --- | --- | --- | --- | --- | --- | --- | --- | --- | --- |
| G9A12 | A | A | G | G | A | A | A | T | T | G | A | T | C | T | C | A | A | T | T | T | G | A | C | A | A | T | G | G | A | G | C | A | A | C | C | A | G | G | - | - | - | - | - | - | - | - | - | - | - | - | - | - | - | - | - | - | - | C | A | C |
| G11C8 | C | A | G | G | A | T | G | T | A | G | A | T | C | T | C | A | C | C | T | T | A | A | G | T | C | T | A | G | C | C | C | A | A | G | C | A | A | C | - | - | - | - | - | - | - | - | - | - | - | - | - | - | - | - | - | - | - | C | C | C |
| G28G10 | C | A | G | G | A | T | T | T | A | G | A | T | C | T | C | G | G | T | T | T | A | C | A | C | C | T | A | G | C | C | C | C | A | T | C | A | G | A | G | A | G | C | C | G | T | G | A | G | G | G | A | A | T | A | A | A | T | C | T | C |
| S19E7 | G | A | G | G | A | T | A | A | A | G | A | T | C | G | C | G | A | C | T | T | A | - | - | - | C | T | A | G | T | T | C | C | A | C | C | A | G | G | G | A | G | C | C | A | A | T | A | T | G | A | A | A | A | A | T | A | T | C | T | C |
| G6D3 | A | A | A | A | T | G | T | T | T | A | G | G | C | C | A | T | T | T | A | A | A | A | A | G | C | C | A | G | G | A | T | - | - | T | T | T | G | A | C | - | - | - | - | - | - | - | - | - | - | G | A | A | A | A | A | A | G | C | T | T |
| S10A9 | A | A | A | A | T | A | C | T | T | A | A | C | C | T | T | T | G | T | A | A | A | A | A | A | C | A | A | T | G | C | T | G | A | T | T | T | G | A | T | - | - | - | - | - | - | - | - | - | - | A | C | C | G | C | A | G | G | A | T | C |

| G15B9 | A | - | - | - | - | - | - | - | - | - | - | - | - | C | C | A | A | A | T | A | C | C | C | A | T | G | C | A | C | T | T | T | T | G | A | G | T | C | C | A | G | A | A | A | A | C | G | T | G | G | A | - | - | - | - | - | - | - | - | G |
| --- | --- | --- | --- | --- | --- | --- | --- | --- | --- | --- | --- | --- | --- | --- | --- | --- | --- | --- | --- | --- | --- | --- | --- | --- | --- | --- | --- | --- | --- | --- | --- | --- | --- | --- | --- | --- | --- | --- | --- | --- | --- | --- | --- | --- | --- | --- | --- | --- | --- | --- | --- | --- | --- | --- | --- | --- | --- | --- | --- | --- |
| G9A12 | A | - | - | - | - | - | - | - | - | - | - | - | - | T | C | C | A | A | - | - | - | - | - | - | - | - | - | - | - | - | - | - | - | - | - | - | - | - | - | - | - | - | - | - | - | - | - | - | G | A | G | - | - | - | - | - | - | - | - | G |
| G11C8 | A | - | - | - | - | - | - | - | - | - | - | - | - | C | C | A | G | T | A | T | C | C | G | A | T | G | A | A | C | - | - | - | - | - | - | - | - | - | - | - | - | - | - | - | A | G | A | G | T | C | G | - | - | - | - | - | - | - | - | T |
| G28G10 | A | A | C | T | T | A | C | G | C | A | T | A | G | C | C | C | G | A | C | C | A | A | C | C | C | C | A | C | C | A | C | C | A | G | A | G | A | C | C | G | A | A | T | A | T | G | A | G | A | A | A | T | A | T | G | T | C | G | A | A |
| S19E7 | A | C | A | T | C | A | T | C | C | G | C | C | A | A | T | C | G | A | T | A | T | T | C | G | A | A | A | A | C | A | T | T | C | C | A | T | C | G | C | G | A | G | C | A | A | C | A | T | G | A | A | - | - | - | A | T | A | A | A | G |
| G6D3 | T | - | - | - | - | - | - | - | - | - | C | C | G | G | C | T | G | T | T | A | A | A | T | T | G | G | A | T | T | C | C | G | T | T | T | G | T | T | T | A | G | A | C | G | A | C | T | G | G | A | A | - | - | - | - | - | - | A | A | G |
| S10A9 | G | T | G | T | C | A | - | - | - | A | C | C | A | T | T | T | A | T | T | A | T | C | T | T | G | G | G | A | T | C | G | C | T | G | G | G | G | A | G | G | T | A | T | A | T | C | T | A | A | A | T | - | - | - | - | - | - | A | T | G |

| G15B9 | T | C | A | G | T | C | G | A | G | C | T | A | T | T | G | G | A | A | C | A | A | G | G | T | A | T | A | A | T | G | T | G | T | G | T | T | G | C | - | - | G | A | T | G | A | A | A | T | A | T | T | G | G | G | A | A | C | G | T | A |
| --- | --- | --- | --- | --- | --- | --- | --- | --- | --- | --- | --- | --- | --- | --- | --- | --- | --- | --- | --- | --- | --- | --- | --- | --- | --- | --- | --- | --- | --- | --- | --- | --- | --- | --- | --- | --- | --- | --- | --- | --- | --- | --- | --- | --- | --- | --- | --- | --- | --- | --- | --- | --- | --- | --- | --- | --- | --- | --- | --- | --- |
| G9A12 | C | T | A | A | T | C | A | A | G | G | C | A | T | T | T | A | A | A | G | C | A | G | C | T | T | T | G | G | G | G | G | C | C | G | T | T | C | G | - | - | C | T | C | G | G | A | C | A | A | C | C | T | T | G | A | C | C | - | - | - |
| G11C8 | C | A | A | A | T | C | A | T | G | T | T | T | T | T | G | G | T | A | C | A | A | C | A | T | T | T | A | G | A | C | G | C | T | G | C | A | G | T | - | - | T | T | T | G | G | C | T | T | A | T | A | T | A | G | A | T | T | - | T | A |
| G28G10 | T | T | A | T | C | C | A | A | G | A | A | T | C | T | T | T | A | T | T | C | G | A | C | T | T | T | A | C | C | C | G | C | C | A | A | C | T | C | C | G | T | A | G | G | A | A | A | A | A | T | C | A | G | G | G | A | A | T | T | G |
| S19E7 | T | C | A | A | T | C | A | G | A | T | G | G | C | T | T | G | A | A | T | A | T | T | A | T | T | T | A | C | T | T | G | T | T | A | A | T | T | T | - | - | T | T | A | T | A | A | T | T | A | T | A | A | A | G | A | T | A | C | G | G |
| G6D3 | A | A | T | A | C | C | A | A | A | C | G | T | T | C | A | G | A | A | A | G | T | G | T | T | C | G | T | T | T | T | G | C | C | T | G | C | T | T | T | T | G | T | G | G | G | A | A | C | G | T | T | G | A | G | A | C | T | - | - | - |
| S10A9 | A | A | A | A | A | A | A | T | A | C | A | A | T | - | G | T | T | A | T | G | G | A | T | T | T | A | T | G | T | A | G | C | T | G | T | A | T | T | G | C | G | T | A | T | G | A | A | C | G | A | T | T | T | A | A | T | T | G | A | T |

| G15B9 | - | - | - | - | - | - | - | - | - | T | G | A | A | A | C | T | C | G | A | T | C | G | T | T | C | G | C | A | A | T | T | - | T | G | A | G | G | - | - | - | - | - | - | - | - | - | - | - | - | - | - | - | - | A | T | T | G | G | T | G |
| --- | --- | --- | --- | --- | --- | --- | --- | --- | --- | --- | --- | --- | --- | --- | --- | --- | --- | --- | --- | --- | --- | --- | --- | --- | --- | --- | --- | --- | --- | --- | --- | --- | --- | --- | --- | --- | --- | --- | --- | --- | --- | --- | --- | --- | --- | --- | --- | --- | --- | --- | --- | --- | --- | --- | --- | --- | --- | --- | --- | --- |
| G9A12 | - | - | - | - | - | - | - | - | - | - | - | A | A | A | A | T | G | C | A | T | T | A | T | T | G | G | C | A | T | A | T | - | T | G | G | A | A | - | - | - | - | - | - | - | - | - | - | - | - | - | - | - | - | A | A | G | T | T | T | A |
| G11C8 | - | - | - | - | - | - | - | - | - | T | T | G | G | G | T | T | A | C | A | A | T | G | T | T | G | A | T | A | A | T | T | A | T | G | T | G | C | - | - | - | - | - | - | - | - | - | - | - | - | - | - | - | - | A | T | T | G | G | T | G |
| G28G10 | G | A | A | A | G | A | A | A | T | T | T | A | G | A | C | C | G | T | G | C | A | A | T | T | T | G | G | T | T | T | T | T | T | A | A | A | T | C | G | A | G | G | C | A | A | C | C | A | C | A | T | T | T | G | A | T | G | T | T | C |
| S19E7 | C | G | A | A | A | G | A | T | T | A | C | G | G | A | T | T | C | G | A | C | G | A | T | A | C | A | A | A | A | T | T | T | T | G | G | A | G | - | - | - | - | - | - | - | - | - | - | - | - | - | - | T | T | G | G | T | G | G | G | G |
| G6D3 | - | - | - | - | - | - | - | - | - | T | T | A | T | A | A | A | T | T | A | T | C | T | T | T | C | G | A | A | T | C | G | G | C | A | G | T | T | - | - | - | - | - | - | - | - | - | - | - | - | - | - | - | T | C | T | G | A | T | C | A |
| S10A9 | - | - | - | - | - | - | - | - | - | T | C | G | G | G | A | A | C | C | A | T | C | A | A | A | C | C | G | G | A | C | A | A | C | G | G | T | G | - | - | - | - | - | - | - | - | - | - | - | - | - | - | - | G | A | A | A | G | T | C | A |

| G15B9 | G | C | T | G | A | A | G | T | T | A | G | A | T | T | C | C | A | A | A | A | C | C | - | - | - | - | - | A | A | A | T | T | G | A | A | A | T | T | G | G | T | G | C | A | A | C | G | A | C | A | T | C | A | G | G | C | T | T | G | T |
| --- | --- | --- | --- | --- | --- | --- | --- | --- | --- | --- | --- | --- | --- | --- | --- | --- | --- | --- | --- | --- | --- | --- | --- | --- | --- | --- | --- | --- | --- | --- | --- | --- | --- | --- | --- | --- | --- | --- | --- | --- | --- | --- | --- | --- | --- | --- | --- | --- | --- | --- | --- | --- | --- | --- | --- | --- | --- | --- | --- | --- |
| G9A12 | T | C | T | A | - | - | - | - | T | A | G | A | - | - | - | - | A | A | A | A | C | - | - | - | - | - | - | - | A | A | T | T | G | G | A | A | T | T | C | G | C | G | A | C | A | A | A | A | T | A | T | G | A | A | G | A | G | T | G | T |
| G11C8 | G | A | A | A | G | G | T | A | T | A | T | C | T | G | A | C | A | A | T | G | T | T | - | - | - | - | - | A | A | A | G | C | G | A | A | A | A | T | T | G | C | A | T | C | A | A | A | A | T | T | T | C | A | T | T | C | C | G | A | C |
| G28G10 | G | A | A | G | A | T | G | A | T | A | A | A | T | T | T | T | G | G | A | A | A | T | G | G | T | G | G | A | A | A | G | A | A | G | A | A | C | T | T | A | C | C | A | T | C | G | A | T | A | T | A | A | A | A | C | T | A | G | T | A |
| S19E7 | A | G | A | C | C | A | A | A | T | T | G | G | T | T | T | C | G | A | T | A | A | - | - | - | - | - | A | A | A | A | T | C | G | G | A | G | C | T | A | G | G | T | G | C | T | G | C | A | T | T | T | G | A | G | T | T | G | G | A | A |
| G6D3 | G | G | C | A | G | C | T | T | T | T | T | G | - | - | - | - | - | - | - | - | T | G | C | T | T | - | - | - | A | C | T | T | A | G | C | A | T | A | C | A | G | C | G | T | C | T | T | T | G | T | C | A | A | T | C | A | A | C | A | A |
| S10A9 | G | A | T | G | A | T | G | T | T | G | T | G | G | C | T | G | G | A | T | A | C | G | C | T | T | - | - | - | A | T | A | A | G | G | C | A | T | T | G | A | A | T | A | A | A | T | A | T | A | T | C | A | A | G | C | A | A | C | A | A |

| G15B9 | T | T | G | A | A | G | C | G | T | A | T | A | G | C | T | A | C | A | G | A | T | A | T | T | A | T | G | A | G | A | G | T | C | G | C | T | G | A | T | A | T | G | C | G | T | - | - | - | - | C | T | G | T | C | A | A | A | A | G | T |
| --- | --- | --- | --- | --- | --- | --- | --- | --- | --- | --- | --- | --- | --- | --- | --- | --- | --- | --- | --- | --- | --- | --- | --- | --- | --- | --- | --- | --- | --- | --- | --- | --- | --- | --- | --- | --- | --- | --- | --- | --- | --- | --- | --- | --- | --- | --- | --- | --- | --- | --- | --- | --- | --- | --- | --- | --- | --- | --- | --- | --- |
| G9A12 | G | T | G | G | A | G | C | G | T | A | G | A | A | C | A | G | A | A | A | G | G | G | C | T | G | C | A | A | G | T | T | T | C | G | C | T | G | A | A | G | T | G | C | - | - | - | - | - | - | - | - | - | C | G | G | A | C | A | G | T |
| G11C8 | G | C | G | A | A | G | A | A | T | A | A | A | C | A | A | A | C | A | G | T | G | G | G | T | T | C | G | A | G | T | A | A | T | A | A | T | G | T | A | G | G | G | C | G | T | - | - | - | - | G | A | A | A | A | A | G | A | G | C | T |
| G28G10 | A | T | G | G | C | G | C | A | C | A | T | G | T | A | T | G | A | G | A | C | C | G | A | T | A | T | G | T | C | G | G | C | C | A | T | A | A | A | G | A | G | G | C | T | G | C | G | T | C | G | G | C | A | A | G | A | G | A | T | T |
| S19E7 | G | C | T | A | T | A | A | A | A | A | T | A | C | C | A | A | A | G | A | T | T | G | A | T | C | T | G | C | G | A | A | C | C | G | A | T | G | A | G | A | A | T | T | T | A | - | - | - | - | G | A | C | A | A | G | C | A | A | T | T |
| G6D3 | T | G | G | T | T | A | A | C | T | T | T | C | G | A | A | A | A | T | T | A | T | A | T | G | T | G | G | A | A | C | A | A | T | A | T | G | G | G | C | T | T | C | C | T | G | - | - | - | - | - | - | - | A | A | A | G | T | A | A | T |
| S10A9 | A | T | G | G | A | A | G | A | T | A | T | C | G | T | T | A | A | A | T | A | T | A | T | T | A | C | A | T | G | G | C | A | A | A | A | G | G | A | A | G | A | C | T | T | G | - | - | - | - | - | - | - | A | A | G | C | A | A | T | T |

| G15B9 | A | T | G | C | T | T | T | T | G | C | G | C | T | C | A | T | G | A | A | G | T | A | C | T | T | G | A | T | G | C | A | G | T | T | T | T | C | A | G | - | - | - | - | - | - | - | - | - | - | - | - | - | - | G | C | C | A | A | C | - |
| --- | --- | --- | --- | --- | --- | --- | --- | --- | --- | --- | --- | --- | --- | --- | --- | --- | --- | --- | --- | --- | --- | --- | --- | --- | --- | --- | --- | --- | --- | --- | --- | --- | --- | --- | --- | --- | --- | --- | --- | --- | --- | --- | --- | --- | --- | --- | --- | --- | --- | --- | --- | --- | --- | --- | --- | --- | --- | --- | --- | --- |
| G9A12 | G | A | A | A | C | C | G | A | A | A | G | G | - | C | A | T | G | C | G | G | T | A | T | C | A | G | A | T | G | C | A | T | G | C | G | T | T | A | T | - | - | - | - | - | - | - | - | - | - | - | - | - | - | - | T | G | A | A | A | - |
| G11C8 | A | G | A | C | C | A | A | C | T | C | G | C | T | T | T | T | G | A | G | A | C | A | G | T | G | C | G | T | T | C | A | T | T | T | A | T | C | A | A | - | - | - | - | - | - | - | - | - | - | - | - | - | - | - | G | G | A | C | G | - |
| G28G10 | C | C | G | C | A | G | G | T | A | G | A | A | G | A | C | A | G | A | T | A | C | G | C | C | G | C | T | T | C | T | G | C | T | C | G | T | A | A | T | A | T | A | T | T | G | G | A | T | G | G | A | T | T | T | A | T | A | A | A | - |
| S19E7 | T | G | T | C | A | G | A | T | T | C | G | C | T | C | G | T | G | A | A | A | C | A | T | T | G | G | A | T | T | C | A | T | A | T | A | T | A | A | - | - | - | G | T | A | A | A | A | T | G | A | A | - | - | T | A | A | G | G | A | - |
| G6D3 | T | C | G | T | A | A | G | T | T | A | A | A | T | G | A | A | G | A | A | G | C | A | A | A | A | C | T | T | T | T | G | G | A | T | T | T | T | A | T | T | G | C | T | C | C | T | T | T | C | T | A | T | G | C | C | A | A | A | T | - |
| S10A9 | T | C | G | A | G | A | T | A | T | A | G | T | A | A | A | T | A | A | T | G | G | A | G | G | A | T | T | T | T | T | G | G | A | T | T | A | T | C | T | T | G | C | T | T | T | T | T | T | C | T | G | T | G | A | A | A | A | A | T | C |

| G15B9 | T | C | C | C | C | G | T | C | G | A | G | T | C | A | A | A | A | - | - | - | - | - | - | - | - | - | T | A | G | A | A | G | A | A | A | T | A | A | C | C | G | A | G | C | A | A | T | G | G | G | A | T | G | C | C | **T** | **G** | **A** | T | C |
| --- | --- | --- | --- | --- | --- | --- | --- | --- | --- | --- | --- | --- | --- | --- | --- | --- | --- | --- | --- | --- | --- | --- | --- | --- | --- | --- | --- | --- | --- | --- | --- | --- | --- | --- | --- | --- | --- | --- | --- | --- | --- | --- | --- | --- | --- | --- | --- | --- | --- | --- | --- | --- | --- | --- | --- | --- | --- | --- | --- | --- |
| G9A12 | T | A | A | T | A | G | A | T | G | A | G | - | C | A | T | T | T | - | - | - | - | - | - | - | - | - | C | G | G | A | T | T | G | G | A | T | A | A | T | C | C | T | C | A | G | T | T | A | A | A | A | A | C | C | C | T | G | T | T | C |
| G11C8 | G | A | A | T | A | A | T | C | C | - | - | - | C | A | C | C | A | - | - | - | - | - | - | - | - | - | C | A | G | G | A | A | T | G | C | T | G | G | T | T | C | **T** | **G** | **A** | G | C | C | C | A | A | A | T | C | G | C | C | G | A | T | C |
| G28G10 | T | A | A | C | G | A | T | T | T | A | A | T | C | A | A | A | A | G | T | C | C | C | C | - | - | - | T | C | G | A | T | T | G | G | G | T | C | A | A | T | C | G | C | C | A | A | C | G | C | G | A | G | C | T | T | C | A | G | C | T |
| S19E7 | A | A | A | C | A | A | T | A | T | C | G | T | T | A | C | A | C | C | T | T | T | C | C | A | A | A | T | A | G | A | G | T | G | G | T | A | C | A | A | A | T | T | C | C | A | A | A | C | A | G | C | T | G | T | T | A | A | T | G | C |
| G6D3 | T | A | G | A | A | A | C | A | G | A | G | A | A | A | C | G | A | A | - | - | - | - | - | - | - | - | A | A | G | C | G | A | A | C | T | T | C | A | G | T | T | C | T | C | A | A | G | A | A | G | A | T | T | C | T | - | - | - | A | T |
| S10A9 | T | A | A | A | G | A | T | C | C | A | G | A | A | A | T | A | A | T | T | C | - | - | - | - | - | - | G | G | G | C | G | A | T | T | G | A | A | A | T | T | T | T | T | G | A | A | A | G | A | A | C | T | T | C | T | C | C | A | A | C |

**├ 3’-UTR**

| G15B9 | T | G | A | A | T | G | G | - | - | - | - | - | - | - | - | - | - | - | - | - | - | - | - | - | - | - | - | - | - | - | - | - | - | - | - | - | - | - | - | - | - | - | - | - | - | - | - | - | - | A | G | A | A | T | T | A | A | A | A | A |
| --- | --- | --- | --- | --- | --- | --- | --- | --- | --- | --- | --- | --- | --- | --- | --- | --- | --- | --- | --- | --- | --- | --- | --- | --- | --- | --- | --- | --- | --- | --- | --- | --- | --- | --- | --- | --- | --- | --- | --- | --- | --- | --- | --- | --- | --- | --- | --- | --- | --- | --- | --- | --- | --- | --- | --- | --- | --- | --- | --- | --- |
| G9A12 | C | A | G | T | T | G | C | - | - | - | - | - | - | - | - | - | - | - | - | - | - | - | - | - | - | - | - | - | - | - | - | - | - | - | - | - | - | - | - | - | - | - | - | - | - | - | - | - | - | A | G | A | A | T | - | A | A | A | A | A |
| G11C8 | G | G | A | A | T | G | C | - | - | - | - | - | - | - | - | - | - | - | - | - | - | - | - | - | - | - | - | - | - | - | - | - | - | - | - | - | - | - | - | - | - | - | - | - | - | - | - | - | - | A | G | A | A | T | G | A | A | A | A | A |
| G28G10 | T | C | A | A | C | G | T | C | A | G | C | A | A | A | T | C | G | G | G | T | C | A | A | C | A | T | C | G | A | G | G | C | A | A | C | A | T | C | A | C | **T** | **G** | - | - | - | - | **A** | T | C | A | G | A | A | T | G | C | A | G | A | A |
| S19E7 | A | C | A | G | T | T | C | C | A | G | A | T | C | A | A | T | A | A | G | C | C | A | A | C | A | T | C | A | A | G | T | G | A | A | C | A | T | C | G | G | T | T | C | A | A | C | A | T | C | G | G | G | A | T | G | C | A | A | A | A |
| G6D3 | G | G | A | T | T | G | T | T | C | A | T | T | T | A | A | A | C | G | A | T | T | A | G | C | T | G | T | A | G | A | C | T | T | - | - | - | - | - | - | - | - | - | - | - | - | - | - | T | T | T | G | C | G | C | G | A | G | T | A | T |
| S10A9 | A | A | A | G | T | G | C | G | A | A | G | A | T | T | A | T | T | C | A | C | C | A | G | T | T | C | G | G | G | T | A | A | T | - | - | - | - | - | - | - | - | - | - | - | - | - | - | T | A | T | G | C | C | T | T | T | G | T | A | T |

| G15B9 | A | T | A | C | A | A | T | A | A | A | - | - | - | - | - | T | T | T | C | A | T | T | C | - | - | - | C | A | A | A | T | G | T | C | C | - | A | C | G | A | T | A | T | A | T | T | T | C | A | T | A | A | A | T | T | T | A | C | A | T |
| --- | --- | --- | --- | --- | --- | --- | --- | --- | --- | --- | --- | --- | --- | --- | --- | --- | --- | --- | --- | --- | --- | --- | --- | --- | --- | --- | --- | --- | --- | --- | --- | --- | --- | --- | --- | --- | --- | --- | --- | --- | --- | --- | --- | --- | --- | --- | --- | --- | --- | --- | --- | --- | --- | --- | --- | --- | --- | --- | --- | --- |
| G9A12 | A | **T** | **A** | **A** | A | T | A | A | A | A | - | - | - | - | - | T | T | C | A | A | T | T | A | - | - | - | C | A | A | A | T | G | T | C | G | - | A | C | G | A | T | A | T | A | T | T | T | C | A | T | A | A | A | T | T | C | C | C | A | T |
| G11C8 | A | A | A | A | A | A | A | A | A | A | A | T | C | A | A | T | T | C | A | A | T | T | C | - | - | - | C | A | A | A | T | G | T | C | G | - | A | C | G | A | T | A | T | A | T | T | T | C | A | T | G | A | A | T | T | C | C | C | A | T |
| G28G10 | T | T | A | A | T | A | A | A | A | A | T | A | A | A | A | T | T | C | A | A | T | T | C | - | - | - | C | A | A | A | T | G | T | T | C | - | A | C | G | A | C | A | A | A | T | T | T | C | A | T | A | A | A | T | T | T | C | C | A | T |
| S19E7 | T | A | A | A | **T** | **A** | **A** | A | A | A | T | A | A | A | A | T | T | C | A | A | T | T | C | G | C | G | T | G | G | A | C | A | T | T | C | C | A | C | G | A | C | A | T | A | T | T | T | C | A | T | A | G | A | T | T | T | C | C | A | T |
| G6D3 | C | T | T | C | A | A | A | A | A | A | G | C | A | A | A | A | C | C | T | G | C | - | - | - | - | - | - | - | G | G | T | A | C | T | T | C | A | A | C | A | T | C | A | T | C | C | **T** | **G** | **A** | T | C | A | T | T | T | T | C | A | A | G |
| S10A9 | C | T | T | - | - | - | G | C | G | G | C | C | A | A | A | T | C | C | T | G | T | T | C | - | - | - | - | T | G | G | T | G | C | T | T | C | A | T | C | A | T | C | A | A | C | C | **T** | **G** | **A** | T | C | A | T | T | T | T | C | A | A | G |

| G15B9 | T | C | C | C | C | G | T | G | G | A | A | T | A | T | A | T | T | A | T | T | A | A | C | A | A | T | T | - | - | - | - | - | T | C | C | C | A | T | T | T | C | A | T | T | T | G | T | A | C | A | C | C | T | A | A | T | A | T | A | A |
| --- | --- | --- | --- | --- | --- | --- | --- | --- | --- | --- | --- | --- | --- | --- | --- | --- | --- | --- | --- | --- | --- | --- | --- | --- | --- | --- | --- | --- | --- | --- | --- | --- | --- | --- | --- | --- | --- | --- | --- | --- | --- | --- | --- | --- | --- | --- | --- | --- | --- | --- | --- | --- | --- | --- | --- | --- | --- | --- | --- | --- |
| G9A12 | T | C | C | C | - | A | T | G | A | A | A | T | A | T | A | T | T | G | T | T | A | A | C | A | A | T | A | - | - | - | - | - | T | T | C | C | A | T | T | G | C | A | T | T | G | T | A | A | C | A | C | C | T | A | A | T | A | T | A | A |
| G11C8 | T | C | C | C | - | A | T | G | G | A | A | T | A | T | A | T | T | G | T | T | A | A | C | A | A | T | A | - | - | - | - | - | T | C | C | C | A | T | T | T | C | A | T | A | G | T | A | A | C | A | C | C | T | A | C | A | A | - | - | G |
| G28G10 | T | C | C | C | - | A | T | G | G | A | A | T | A | - | - | T | T | G | T | G | A | A | C | A | A | T | A | C | T | T | T | T | T | C | C | C | A | T | T | T | C | A | T | T | G | T | A | A | C | A | C | C | T | A | T | A | A | T | - | - |
| S19E7 | T | T | T | T | - | G | T | G | G | A | A | T | A | - | - | T | T | G | T | G | A | A | T | A | A | T | A | - | - | - | - | - | T | C | T | C | A | T | T | T | C | A | T | T | G | T | A | A | C | A | C | C | T | A | - | - | - | - | - | - |
| G6D3 | A | T | T | C | - | - | T | G | G | - | G | T | T | T | - | T | T | G | G | T | G | A | T | T | G | T | C | - | - | - | - | - | - | T | T | G | G | G | T | A | T | C | T | T | G | T | G | G | C | G | T | T | A | G | A | C | A | G | - | C |
| S10A9 | A | T | T | A | - | - | T | G | G | T | A | T | T | T | - | T | G | G | G | T | G | A | T | T | G | T | C | - | - | - | - | - | - | T | T | T | G | G | T | A | T | G | T | T | G | T | G | G | C | C | T | T | A | G | - | - | - | - | - | - |

| G15B9 | A | C | A | A | A | A | A | - | T | G | T | A | T | T | A | G | G | A | A | T | G | A | T | G | G | A | T | A | A | A | - | - | - | G | A | A | G | A | A | T | A | T | T | C | A | G | T | T | A | T | T | G | A | G | G | C | - | - | - | - |
| --- | --- | --- | --- | --- | --- | --- | --- | --- | --- | --- | --- | --- | --- | --- | --- | --- | --- | --- | --- | --- | --- | --- | --- | --- | --- | --- | --- | --- | --- | --- | --- | --- | --- | --- | --- | --- | --- | --- | --- | --- | --- | --- | --- | --- | --- | --- | --- | --- | --- | --- | --- | --- | --- | --- | --- | --- | --- | --- | --- | --- |
| G9A12 | A | A | A | A | A | A | A | A | T | G | T | A | T | A | A | T | G | A | A | T | G | A | T | G | A | A | T | A | A | A | - | - | - | G | A | A | G | G | A | T | A | T | T | C | A | G | T | T | A | T | T | G | A | G | A | A | A | C | T | C |
| G11C8 | T | T | A | A | G | G | A | A | T | G | T | A | T | A | A | G | G | A | A | T | G | A | T | G | A | A | T | A | A | A | - | - | - | A | A | A | G | A | A | T | A | T | T | T | A | A | T | T | A | T | C | - | - | - | - | - | - | - | - | - |
| G28G10 | A | T | G | A | A | A | A | A | T | G | T | A | A | A | A | G | G | A | A | T | G | A | T | A | A | A | T | A | A | A | G | - | - | A | A | A | A | A | A | A | A | A | T | C | T | G | T | T | T | T | T | G | A | G | T | A | A | T | T | C |
| S19E7 | - | - | - | - | - | - | - | - | - | A | T | A | T | A | A | G | A | A | A | T | G | A | T | G | A | A | T | A | A | A | - | - | - | A | A | A | G | A | A | T | A | T | T | C | A | G | T | T | A | T | T | G | A | A | C | A | A | T | T | C |
| G6D3 | A | T | C | T | G | A | T | G | G | C | T | G | T | T | C | G | G | G | A | T | G | G | A | A | G | A | T | A | A | A | A | T | A | A | A | A | T | G | A | A | A | T | T | C | A | A | T | T | T | C | A | A | A | T | A | T | A | T | T | T |
| S10A9 | - | - | - | - | - | - | - | - | - | C | T | T | A | T | C | G | G | A | A | T | G | A | A | A | A | A | T | G | A | A | A | T | A | A | A | A | T | A | A | A | A | T | T | C | A | A | T | T | C | T | A | A | A | C | A | T | G | - | - | - |

| G15B9 | - | - | - | - | - | - | - | - | - | - |  |  |  |  |  |  |  |  |  |  |  |  |  |  |  |  |  |  |  |  |  |  |  |  |  |  |  |  |  |  |  |  |  |  |  |  |  |  |  |  |  |  |  |  |  |  |  |  |  |  |
| --- | --- | --- | --- | --- | --- | --- | --- | --- | --- | --- | --- | --- | --- | --- | --- | --- | --- | --- | --- | --- | --- | --- | --- | --- | --- | --- | --- | --- | --- | --- | --- | --- | --- | --- | --- | --- | --- | --- | --- | --- | --- | --- | --- | --- | --- | --- | --- | --- | --- | --- | --- | --- | --- | --- | --- | --- | --- | --- | --- | --- |
| G9A12 | T | C | T | C | T | C | G | C | A | C |  |  |  |  |  |  |  |  |  |  |  |  |  |  |  |  |  |  |  |  |  |  |  |  |  |  |  |  |  |  |  |  |  |  |  |  |  |  |  |  |  |  |  |  |  |  |  |  |  |  |
| G11C8 | - | - | - | - | - | - | - | - | - | - |  |  |  |  |  |  |  |  |  |  |  |  |  |  |  |  |  |  |  |  |  |  |  |  |  |  |  |  |  |  |  |  |  |  |  |  |  |  |  |  |  |  |  |  |  |  |  |  |  |  |
| G28G10 | T | C | T | C | T | T | C | G | C | - |  |  |  |  |  |  |  |  |  |  |  |  |  |  |  |  |  |  |  |  |  |  |  |  |  |  |  |  |  |  |  |  |  |  |  |  |  |  |  |  |  |  |  |  |  |  |  |  |  |  |
| S19E7 | T | C | T | C | T | T | C | C | - | - |  |  |  |  |  |  |  |  |  |  |  |  |  |  |  |  |  |  |  |  |  |  |  |  |  |  |  |  |  |  |  |  |  |  |  |  |  |  |  |  |  |  |  |  |  |  |  |  |  |  |
| G6D3 | G | - | - | - | - | - | - | - | - | - |  |  |  |  |  |  |  |  |  |  |  |  |  |  |  |  |  |  |  |  |  |  |  |  |  |  |  |  |  |  |  |  |  |  |  |  |  |  |  |  |  |  |  |  |  |  |  |  |  |  |
| S10A9 | - | - | - | - | - | - | - | - | - | - |  |  |  |  |  |  |  |  |  |  |  |  |  |  |  |  |  |  |  |  |  |  |  |  |  |  |  |  |  |  |  |  |  |  |  |  |  |  |  |  |  |  |  |  |  |  |  |  |  |  |

**Protein alignment**

| G15B9 | M | F | N | S | Q | K | L | I | I | C | C | L | L | F | A | V | V | L | L | Q | S | L | E | A | G | S | N | - | - | - | - | - | - | - | - | - | - | - | - | - | - | - | - | - | - | - | - | - | - | - | - | - | - | - | - | - | - | - | - | - |
| --- | --- | --- | --- | --- | --- | --- | --- | --- | --- | --- | --- | --- | --- | --- | --- | --- | --- | --- | --- | --- | --- | --- | --- | --- | --- | --- | --- | --- | --- | --- | --- | --- | --- | --- | --- | --- | --- | --- | --- | --- | --- | --- | --- | --- | --- | --- | --- | --- | --- | --- | --- | --- | --- | --- | --- | --- | --- | --- | --- | --- |
| G9A12 | M | L | N | S | Q | K | L | I | I | C | C | L | L | F | A | A | V | L | L | Q | S | L | K | A | T | P | K | R | K | - | - | - | - | - | - | - | - | - | - | - | - | - | - | - | - | - | - | - | - | - | - | - | - | - | - | - | - | - | - | - |
| G11C8 | M | F | N | S | Q | K | L | I | I | F | S | L | L | F | A | A | V | W | V | Q | F | S | K | A | A | P | K | R | R | - | - | - | - | - | - | - | - | - | - | - | - | - | - | - | - | - | - | - | - | - | - | - | - | - | - | - | - | - | - | - |
| G28G10 | M | F | N | S | Q | K | L | S | I | C | C | L | L | F | A | A | V | W | L | Q | S | L | K | A | T | P | T | G | N | G | E | D | A | S | Q | P | L | L | G | R | L | A | Y | E | Q | V | Y | Q | H | A | P | Q | P | P | S | P | P | Q | P | P |
| S19E7 | M | F | N | S | Q | K | L | I | I | C | C | L | L | F | A | A | V | W | V | Q | S | L | K | A | A | L | T | P | G | - | - | - | - | - | - | - | - | - | - | - | - | - | - | - | - | - | - | - | - | - | - | - | - | - | - | - | - | - | - | - |
| G6D3 | M | L | N | S | Q | S | L | I | V | C | C | L | L | F | A | T | V | W | A | T | E | E | Q | P | P | A | Q | K | K | I | E | S | Q | G | L | E | F | F | E | K | C | L | G | - | - | - | - | - | - | - | - | - | - | - | - | - | - | - | - | - |
| S10A9 | M | L | N | S | Q | K | L | I | I | C | C | L | V | F | A | A | I | W | V | Q | S | L | K | A | V | Q | M | S | K | H | Y | D | E | E | I | A | H | F | E | K | Y | L | T | F | V | K | N | - | - | - | - | - | - | - | - | - | - | - | - | - |
|  |  |  |  |  |  |  |  |  |  |  |  |  |  |  |  |  |  |  |  |  |  |  |  |  |  |  |  |  |  |  |  |  |  |  |  |  |  |  |  |  |  |  |  |  |  |  |  |  |  |  |  |  |  |  |  |  |  |  |  |  |
| G15B9 | - | - | - | - | - | - | - | - | - | G | K | E | I | D | L | N | L | S | I | A | - | - | - | - | - | - | - | - | - | - | - | - | - | Q | P | G | S | P | N | - | - | T | H | A | L | L | S | - | - | - | - | - | - | - | - | - | - | - | - | P |
| G9A12 | - | - | - | - | - | - | - | - | - | G | K | E | I | D | L | N | L | T | M | E | - | - | - | - | - | - | - | - | - | - | - | - | - | Q | P | G | T | S | K | R | L | I | K | A | F | K | A | - | - | - | - | - | - | - | - | - | - | - | - | A |
| G11C8 | - | - | - | - | - | - | - | - | - | P | Q | D | V | D | L | T | L | S | L | A | - | - | - | - | - | - | - | - | - | - | - | - | - | - | - | - | - | - | - | Q | A | T | P | P | V | S | D | - | - | - | - | - | - | - | - | - | - | - | - | - |
| G28G10 | L | S | Y | S | Q | D | L | D | L | G | L | H | L | A | P | S | E | S | R | E | G | I | N | L | N | L | R | I | A | R | P | T | P | P | P | E | T | E | Y | E | K | Y | V | E | L | S | K | N | L | Y | S | - | - | - | - | - | T | L | P | A |
| S19E7 | - | - | - | - | - | - | - | - | L | S | I | Y | L | D | E | D | K | D | R | D | - | - | - | - | - | - | - | - | - | - | L | L | V | P | P | G | S | Q | Y | E | K | Y | L | T | S | S | A | N | R | Y | S | K | T | F | - | - | H | R | E | Q |
| G6D3 | - | - | - | - | - | - | H | L | K | S | Q | D | F | D | E | K | S | F | P | A | V | K | L | D | S | V | C | L | D | D | W | K | R | I | P | N | V | Q | K | V | F | V | L | P | A | F | V | G | - | - | - | - | - | - | - | - | - | - | T | L |
| S10A9 | - | - | - | N | A | D | L | I | P | Q | D | R | V | N | H | L | L | S | W | D | R | W | G | - | - | - | - | - | G | I | S | K | Y | E | K | N | T | M | L | W | I | Y | V | A | V | L | R | M | N | D | L | I | D | S | G | T | I | K | P | D |
|  |  |  |  |  |  |  |  |  |  |  |  |  |  |  |  |  |  |  |  |  |  |  |  |  |  |  |  |  |  |  |  |  |  |  |  |  |  |  |  |  |  |  |  |  |  |  |  |  |  |  |  |  |  |  |  |  |  |  |  |  |
| G15B9 | E | N | V | E | S | V | E | L | L | E | Q | G | I | M | C | V | A | M | K | Y | W | E | R | - | - | - | M | K | L | D | R | S | Q | F | E | D | W | W | - | L | K | L | D | S | K | T | K | L | K | L | V | Q | R | H | Q | A | C | L | K | R |
| G9A12 | L | G | A | V | R | S | D | N | L | D | Q | - | - | - | N | A | L | L | A | Y | W | K | S | - | - | - | L | S | I | E | K | - | - | - | - | - | - | - | - | - | Q | L | E | F | A | T | K | Y | E | E | C | V | E | R | R | T | E | R | A | A |
| G11C8 | E | Q | S | R | Q | I | M | F | L | V | Q | H | L | D | A | A | V | L | A | Y | I | D | L | - | - | - | L | G | Y | N | V | D | N | Y | V | H | W | W | - | K | G | I | S | D | N | V | K | A | K | I | A | S | K | F | H | S | D | A | K | N |
| G28G10 | N | S | V | G | K | I | R | E | L | E | R | N | L | D | R | A | I | W | F | F | K | S | R | Q | P | H | L | M | F | E | D | D | K | F | W | K | W | W | K | E | E | L | T | I | D | I | K | L | V | M | A | H | M | Y | E | T | D | M | S | A |
| S19E7 | H | E | I | K | S | I | R | W | L | E | Y | Y | L | L | V | N | F | Y | N | Y | K | D | T | A | K | D | Y | G | F | D | D | T | K | F | W | S | W | W | G | D | Q | I | G | F | D | K | K | S | E | L | G | A | A | F | E | L | E | A | I | K |
| G6D3 | R | L | Y | K | L | S | F | E | S | A | V | S | D | Q | A | A | F | C | A | Y | L | A | Y | S | V | F | V | N | Q | Q | W | L | T | F | E | N | Y | M | W | N | N | M | G | F | L | K | V | I | R | K | L | N | E | E | A | K | L | L | D | F |
| S10A9 | N | G | G | K | S | D | D | V | V | A | G | Y | A | Y | K | A | L | N | K | Y | I | K | Q | - | - | - | Q | M | E | D | I | V | K | Y | I | T | W | Q | K | E | D | L | K | Q | F | R | D | I | V | N | N | G | G | F | L | D | Y | L | A | F |
|  |  |  |  |  |  |  |  |  |  |  |  |  |  |  |  |  |  |  |  |  |  |  |  |  |  |  |  |  |  |  |  |  |  |  |  |  |  |  |  |  |  |  |  |  |  |  |  |  |  |  |  |  |  |  |  |  |  |  |  |  |
| G15B9 | I | A | T | D | I | M | R | V | A | D | M | R | L | S | K | V | C | F | C | A | H | E | V | L | D | A | V | F | R | - | - | - | - | - | - | - | - | - | - | - | - | - | - | - | - | - | P | T | P | R | R | V | K | I | E | E | I | T | E | Q |
| G9A12 | S | F | A | E | V | P | D | S | E | T | E | R | H | A | V | S | D | A | C | V | I | E | I | I | D | E | H | F | G | - | - | - | - | - | - | - | - | - | - | - | - | - | - | - | - | - | L | D | N | P | Q | L | K | T | L | F | Q | L | Q | N |
| G11C8 | K | Q | T | V | G | S | S | N | N | V | G | R | E | K | E | L | D | Q | L | A | F | E | T | V | R | S | F | I | K | - | - | - | - | - | - | - | - | - | - | - | - | - | - | - | - | - | - | - | D | G | I | I | P | P | Q | E | C | W | F | - |
| G28G10 | I | K | R | L | R | R | Q | E | I | P | Q | V | E | D | R | Y | A | A | S | A | R | N | I | L | D | G | F | I | N | - | - | - | - | - | - | N | D | L | I | K | S | P | L | D | W | V | N | R | Q | R | E | L | Q | L | Q | R | Q | Q | I | G |
| S19E7 | I | P | K | I | D | L | R | T | D | E | N | L | D | K | Q | F | V | R | F | A | R | E | T | L | D | S | Y | I | S | K | M | N | K | E | N | N | I | V | T | P | F | Q | I | E | W | Y | K | F | Q | T | A | V | N | A | Q | F | Q | I | N | K |
| G6D3 | I | A | P | F | Y | A | K | L | E | T | E | K | R | K | A | N | F | S | S | Q | E | D | S | M | D | C | S | F | K | R | - | - | - | - | - | - | - | - | - | - | - | - | - | - | - | - | L | A | V | D | F | L | R | E | Y | L | Q | K | S | K |
| S10A9 | F | C | E | K | S | K | D | P | E | I | I | R | A | I | E | I | F | E | R | T | S | P | T | K | C | E | D | Y | S | - | - | - | - | - | - | - | - | - | - | - | - | - | - | - | - | - | P | V | R | V | I | M | P | L | Y | L | A | A | K | S |
|  |  |  |  |  |  |  |  |  |  |  |  |  |  |  |  |  |  |  |  |  |  |  |  |  |  |  |  |  |  |  |  |  |  |  |  |  |  |  |  |  |  |  |  |  |  |  |  |  |  |  |  |  |  |  |  |  |  |  |  |  |
| G15B9 | W | D | A | - | - | - | - | - | - | - | - | - | - | - | - |  |  |  |  |  |  |  |  |  |  |  |  |  |  |  |  |  |  |  |  |  |  |  |  |  |  |  |  |  |  |  |  |  |  |  |  |  |  |  |  |  |  |  |  |  |
| G9A12 | K | K | - | - | - | - | - | - | - | - | - | - | - | - | - |  |  |  |  |  |  |  |  |  |  |  |  |  |  |  |  |  |  |  |  |  |  |  |  |  |  |  |  |  |  |  |  |  |  |  |  |  |  |  |  |  |  |  |  |  |
| G11C8 | - | - | - | - | - | - | - | - | - | - | - | - | - | - | - |  |  |  |  |  |  |  |  |  |  |  |  |  |  |  |  |  |  |  |  |  |  |  |  |  |  |  |  |  |  |  |  |  |  |  |  |  |  |  |  |  |  |  |  |  |
| G28G10 | S | T | S | R | Q | H | H | - | - | - | - | - | - | - | - |  |  |  |  |  |  |  |  |  |  |  |  |  |  |  |  |  |  |  |  |  |  |  |  |  |  |  |  |  |  |  |  |  |  |  |  |  |  |  |  |  |  |  |  |  |
| S19E7 | P | T | S | S | E | H | R | F | N | I | G | M | Q | N | K |  |  |  |  |  |  |  |  |  |  |  |  |  |  |  |  |  |  |  |  |  |  |  |  |  |  |  |  |  |  |  |  |  |  |  |  |  |  |  |  |  |  |  |  |  |
| G6D3 | T | C | G | T | S | T | S | S | - | - | - | - | - | - | - |  |  |  |  |  |  |  |  |  |  |  |  |  |  |  |  |  |  |  |  |  |  |  |  |  |  |  |  |  |  |  |  |  |  |  |  |  |  |  |  |  |  |  |  |  |
| S10A9 | C | S | G | A | S | S | S | T | - | - | - | - | - | - | - |  |  |  |  |  |  |  |  |  |  |  |  |  |  |  |  |  |  |  |  |  |  |  |  |  |  |  |  |  |  |  |  |  |  |  |  |  |  |  |  |  |  |  |  |  |

**D**

**├ 5’-UTR**

| G13E11 | - | - | - | - | - | - | G | A | A | G | T | T | A | A | C | T | G | T | T | T | A | T | T | C | T | T | C | A | G | T | A | A | A | C | G | A | C | A | T | A | A | A | T | A | C | T | A | A | A | T | A | C | T | T | C | A | G | A | T | A |
| --- | --- | --- | --- | --- | --- | --- | --- | --- | --- | --- | --- | --- | --- | --- | --- | --- | --- | --- | --- | --- | --- | --- | --- | --- | --- | --- | --- | --- | --- | --- | --- | --- | --- | --- | --- | --- | --- | --- | --- | --- | --- | --- | --- | --- | --- | --- | --- | --- | --- | --- | --- | --- | --- | --- | --- | --- | --- | --- | --- | --- |
| G16A3 | - | - | - | - | - | - | G | A | A | G | T | T | A | A | C | T | G | T | T | T | A | T | T | C | T | T | C | A | G | T | A | A | A | C | G | A | C | C | T | A | A | A | T | A | C | T | A | A | A | T | A | C | T | T | C | A | G | A | T | A |
| G11A3 | C | A | G | A | A | G | T | C | A | G | T | T | A | A | C | T | G | T | T | T | A | T | T | C | T | T | T | A | G | T | A | A | A | C | G | A | C | A | T | A | A | A | T | A | C | T | A | A | A | T | A | C | T | T | C | A | G | A | T | A |

**├ SPCR**

| G13E11 | T | C | T | T | A | A | A | A | C | A | G | A | A | G | C | C | A | A | A | A | C | C | T | G | A | A | G | **A** | **T** | **G** | A | A | A | T | T | A | T | T | T | T | T | C | T | G | G | T | T | G | T | T | T | G | C | G | A | T | T | T | T | G |
| --- | --- | --- | --- | --- | --- | --- | --- | --- | --- | --- | --- | --- | --- | --- | --- | --- | --- | --- | --- | --- | --- | --- | --- | --- | --- | --- | --- | --- | --- | --- | --- | --- | --- | --- | --- | --- | --- | --- | --- | --- | --- | --- | --- | --- | --- | --- | --- | --- | --- | --- | --- | --- | --- | --- | --- | --- | --- | --- | --- | --- |
| G16A3 | T | A | T | T | A | A | A | A | C | A | G | A | A | G | C | C | A | A | A | G | C | C | T | A | A | A | G | **A** | **T** | **G** | A | A | A | T | T | A | A | T | T | T | T | C | T | G | G | T | T | G | T | T | T | G | C | A | A | T | T | T | T | G |
| G11A3 | T | C | T | T | A | A | A | A | C | A | G | A | A | G | C | C | A | A | A | G | C | C | T | A | A | A | G | **A** | **T** | **G** | A | A | A | T | T | A | T | T | T | T | T | C | T | G | G | T | T | G | T | T | T | G | T | A | A | T | T | A | T | G |

**├ MPCR**

| G13E11 | G | T | T | G | T | T | G | T | G | C | A | G | G | T | A | C | T | C | G | T | T | C | C | A | A | T | A | G | C | C | T | C | G | G | C | T | A | A | T | C | C | T | G | G | C | A | G | - | - | - | - | A | G | C | A | T | C | A | A | A |
| --- | --- | --- | --- | --- | --- | --- | --- | --- | --- | --- | --- | --- | --- | --- | --- | --- | --- | --- | --- | --- | --- | --- | --- | --- | --- | --- | --- | --- | --- | --- | --- | --- | --- | --- | --- | --- | --- | --- | --- | --- | --- | --- | --- | --- | --- | --- | --- | --- | --- | --- | --- | --- | --- | --- | --- | --- | --- | --- | --- | --- |
| G16A3 | G | T | T | G | T | T | G | T | G | C | A | G | G | T | A | C | T | C | G | T | T | C | C | A | A | T | A | G | C | C | T | C | G | G | C | T | C | A | T | G | G | T | G | G | T | G | G | T | G | A | - | G | G | C | A | T | C | A | C | A |
| G11A3 | G | T | T | G | T | T | G | T | G | C | A | G | G | T | A | C | T | C | G | T | T | C | C | A | A | T | A | G | C | C | T | C | G | G | C | T | G | G | T | G | G | T | G | G | T | G | G | T | G | G | T | G | G | T | G | G | T | C | A | T |

| G13E11 | A | A | A | A | A | C | G | A | C | G | G | T | T | G | G | T | G | G | T | G | G | T | G | - | - | - | - | - | - | - | - | - | A | G | G | C | A | T | C | A | C | C | - | - | A | A | A | A | A | C | A | C | C | G | G | C | T | G | G | T |
| --- | --- | --- | --- | --- | --- | --- | --- | --- | --- | --- | --- | --- | --- | --- | --- | --- | --- | --- | --- | --- | --- | --- | --- | --- | --- | --- | --- | --- | --- | --- | --- | --- | --- | --- | --- | --- | --- | --- | --- | --- | --- | --- | --- | --- | --- | --- | --- | --- | --- | --- | --- | --- | --- | --- | --- | --- | --- | --- | --- | --- |
| G16A3 | A | A | A | A | A | C | G | C | C | G | G | C | T | G | G | T | G | G | C | G | G | A | G | G | A | G | - | - | - | - | - | - | A | G | G | G | A | A | C | G | T | C | - | - | A | G | G | A | T | C | A | A | G | C | A | T | T | A | G | A |
| G11A3 | G | G | A | C | A | T | G | - | C | G | G | C | T | G | G | T | G | G | T | A | G | T | G | G | A | C | A | T | T | C | A | A | A | A | G | T | G | T | C | G | G | C | T | G | A | C | A | A | A | C | A | T | C | A | G | A | C | A | A | C |

| G13E11 | C | G | T | G | G | T | - | - | - | - | - | - | - | - | - | G | G | T | G | A | A | A | - | - | - | - | - | - | G | T | A | C | A | G | A | A | G | - | - | - | - | - | - | - | - | - | - | - | - | - | T | C | A | A | A | G | A | T | C | - |
| --- | --- | --- | --- | --- | --- | --- | --- | --- | --- | --- | --- | --- | --- | --- | --- | --- | --- | --- | --- | --- | --- | --- | --- | --- | --- | --- | --- | --- | --- | --- | --- | --- | --- | --- | --- | --- | --- | --- | --- | --- | --- | --- | --- | --- | --- | --- | --- | --- | --- | --- | --- | --- | --- | --- | --- | --- | --- | --- | --- | --- |
| G16A3 | G | A | A | A | G | A | - | - | - | - | - | - | - | - | - | A | A | A | T | T | G | G | - | - | - | - | - | - | A | T | T | C | G | A | A | G | A | G | G | C | C | C | A | C | T | T | C | C | C | T | T | C | A | A | A | T | A | C | C | - |
| G11A3 | G | C | C | A | G | T | C | C | A | T | T | C | C | C | C | G | A | T | A | A | A | A | C | C | T | G | G | A | A | C | A | T | C | A | G | G | G | C | C | C | C | C | A | C | C | A | A | T | A | G | G | T | G | A | A | T | A | T | T | A |

| G13E11 | - | - | - | - | T | A | - | - | - | - | - | A | G | A | G | A | A | C | T | G | C | A | C | C | A | T | A | C | A | G | C | T | A | A | G | - | - | - | - | A | G | C | A | A | G | A | A | G | A | T | T | C | T | T | C | C | T | C | G | G |
| --- | --- | --- | --- | --- | --- | --- | --- | --- | --- | --- | --- | --- | --- | --- | --- | --- | --- | --- | --- | --- | --- | --- | --- | --- | --- | --- | --- | --- | --- | --- | --- | --- | --- | --- | --- | --- | --- | --- | --- | --- | --- | --- | --- | --- | --- | --- | --- | --- | --- | --- | --- | --- | --- | --- | --- | --- | --- | --- | --- | --- |
| G16A3 | - | - | - | - | T | G | C | A | C | C | C | A | A | A | A | T | A | G | C | G | G | A | C | G | A | A | A | C | A | A | C | C | G | A | G | G | A | G | G | A | A | G | A | G | G | A | A | G | A | A | A | C | T | T | G | C | G | A | A | C |
| G11A3 | C | C | A | A | C | A | A | G | G | A | A | A | G | A | A | A | A | A | C | G | C | A | G | C | A | C | A | G | A | A | C | T | T | G | C | C | T | A | C | G | G | G | T | C | A | A | A | G | T | G | C | A | A | C | C | C | C | A | A | G |

| G13E11 | G | G | - | - | - | - | - | - | T | G | C | G | C | - | C | T | C | C | A | G | T | T | A | G | - | - | - | - | - | - | A | G | C | T | C | T | T | T | T | T | C | A | A | A | A | T | - | - | - | - | - | - | - | - | - | - | - | - | - | - |
| --- | --- | --- | --- | --- | --- | --- | --- | --- | --- | --- | --- | --- | --- | --- | --- | --- | --- | --- | --- | --- | --- | --- | --- | --- | --- | --- | --- | --- | --- | --- | --- | --- | --- | --- | --- | --- | --- | --- | --- | --- | --- | --- | --- | --- | --- | --- | --- | --- | --- | --- | --- | --- | --- | --- | --- | --- | --- | --- | --- | --- |
| G16A3 | C | A | - | - | - | - | - | - | T | T | A | A | T | - | C | C | G | A | A | A | A | A | A | T | - | - | - | - | - | - | A | G | C | A | A | A | A | T | A | G | C | A | A | A | A | T | C | A | C | A | A | A | G | C | G | A | A | A | C | A |
| G11A3 | A | A | C | A | G | T | A | A | T | A | C | G | T | G | T | T | C | C | G | G | A | T | A | A | T | T | T | A | G | A | A | G | A | T | G | T | T | T | T | T | G | C | T | C | C | T | A | A | A | G | A | A | C | A | A | A | C | G | G | G |

├ 3’-UTR

| G13E11 | - | - | - | - | - | - | A | T | A | G | C | A | G | - | - | - | - | - | - | - | - | - | C | G | A | A | T | G | - | A | A | G | A | A | G | - | - | - | - | A | T | A | A | - | - | T | A | A | - | - | G | G | A | T | T | C | T | - | - | - |
| --- | --- | --- | --- | --- | --- | --- | --- | --- | --- | --- | --- | --- | --- | --- | --- | --- | --- | --- | --- | --- | --- | --- | --- | --- | --- | --- | --- | --- | --- | --- | --- | --- | --- | --- | --- | --- | --- | --- | --- | --- | --- | --- | --- | --- | --- | --- | --- | --- | --- | --- | --- | --- | --- | --- | --- | --- | --- | --- | --- | --- |
| G16A3 | G | G | G | C | A | T | A | T | G | G | C | A | G | G | A | G | G | A | A | A | A | C | C | A | A | A | T | G | C | A | A | G | G | A | A | - | - | - | - | A | **T** | **A** | **A** | A | A | T | A | A | T | T | A | A | A | A | T | A | G | - | - | - |
| G11A3 | T | T | C | T | T | C | A | A | A | T | A | A | T | **T** | **G** | **A** | A | A | G | T | A | G | C | G | A | A | T | G | A | T | A | A | A | A | C | T | T | A | T | A | T | A | T | A | T | T | T | G | A | T | G | A | A | T | T | C | T | T | T | G |

| G13E11 | A | C | A | C | C | T | T | C | G | A | A | G | C | T | T | C | - | - | - | A | A | T | A | A | A | A | T | A | T | G | A | A | C | T | G | C | T | G | C | G | A | A | A | T | T | T | A | A | A | G | C | T | G | T | T | A | C | A | T | C |
| --- | --- | --- | --- | --- | --- | --- | --- | --- | --- | --- | --- | --- | --- | --- | --- | --- | --- | --- | --- | --- | --- | --- | --- | --- | --- | --- | --- | --- | --- | --- | --- | --- | --- | --- | --- | --- | --- | --- | --- | --- | --- | --- | --- | --- | --- | --- | --- | --- | --- | --- | --- | --- | --- | --- | --- | --- | --- | --- | --- | --- |
| G16A3 | G | A | A | A | A | T | T | G | G | A | A | T | C | T | T | C | C | T | C | A | A | T | G | A | A | A | T | A | T | G | A | A | C | T | G | A | T | G | C | G | A | A | A | T | T | C | A | A | A | T | C | T | G | T | T | A | C | A | T | T |
| G11A3 | G | A | A | T | C | T | T | T | G | A | A | T | C | C | T | C | - | - | - | A | A | **T** | **A** | **A** | A | A | T | A | T | G | A | A | A | T | G | C | T | G | C | G | A | A | G | T | T | T | A | A | A | G | C | T | G | T | T | A | C | A | T | T |

| G13E11 | T | G | C | T | A | A | T | A | A | A | T | T | T | T | C | T | T | A | A | T | T | T | C | A | T | C | A | C | A | A | T | G | T | C | C | - | - | - | - | - | - | - | - | - | - | - | - | - | - | - |  |  |  |  |  |  |  |  |  |  |
| --- | --- | --- | --- | --- | --- | --- | --- | --- | --- | --- | --- | --- | --- | --- | --- | --- | --- | --- | --- | --- | --- | --- | --- | --- | --- | --- | --- | --- | --- | --- | --- | --- | --- | --- | --- | --- | --- | --- | --- | --- | --- | --- | --- | --- | --- | --- | --- | --- | --- | --- | --- | --- | --- | --- | --- | --- | --- | --- | --- | --- |
| G16A3 | T | T | C | T | A | A | T | A | A | A | T | T | T | C | C | T | T | T | A | T | T | T | C | A | T | C | G | C | A | T | T | G | T | G | C | A | T | G | A | A | A | C | A | A | T | T | G | G | C | G |  |  |  |  |  |  |  |  |  |  |
| G11A3 | T | G | C | T | A | A | T | A | A | A | T | A | A | A | C | - | - | A | A | C | T | A | C | - | - | - | - | - | - | - | - | - | - | - | - | - | - | - | - | - | - | - | - | - | - | - | - | - | - | - |  |  |  |  |  |  |  |  |  |  |

**Protein alignment**

| G13E11 | M | K | L | F | F | W | L | F | A | I | L | V | V | V | Q | V | L | V | P | I | A | S | A | N | P | G | R | A | S | K | K | T | T | V | G | G | G | E | A | S | P | K | T | P | A | G | R | G | G | E | S | T | E | V | K | D | L | R | E | L |
| --- | --- | --- | --- | --- | --- | --- | --- | --- | --- | --- | --- | --- | --- | --- | --- | --- | --- | --- | --- | --- | --- | --- | --- | --- | --- | --- | --- | --- | --- | --- | --- | --- | --- | --- | --- | --- | --- | --- | --- | --- | --- | --- | --- | --- | --- | --- | --- | --- | --- | --- | --- | --- | --- | --- | --- | --- | --- | --- | --- | --- |
| G16A3 | M | K | L | I | F | W | L | F | A | I | L | V | V | V | Q | V | L | V | P | I | A | S | A | H | - | - | - | - | - | - | - | - | - | - | G | G | G | E | A | S | Q | K | T | P | A | G | G | G | G | E | G | T | S | G | S | S | I | R | E | R |
| G11A3 | M | K | L | F | F | W | L | F | V | I | M | V | V | V | Q | V | L | V | P | I | A | S | A | G | - | - | - | - | - | - | - | - | - | - | G | G | G | G | G | G | H | G | H | A | A | G | G | S | G | H | S | K | V | S | A | D | K | H | Q | T |
|  |  |  |  |  |  |  |  |  |  |  |  |  |  |  |  |  |  |  |  |  |  |  |  |  |  |  |  |  |  |  |  |  |  |  |  |  |  |  |  |  |  |  |  |  |  |  |  |  |  |  |  |  |  |  |  |  |  |  |  |  |
| G13E11 | H | H | T | A | K | S | K | K | I | L | P | R | G | A | P | P | V | R | - | - | - | - | - | - | - | - | - | - | - | - | - | - | - | - | - | - | - | A | L | F | Q | N | - | - | - | I | A | A | N | E | E | D | N | - | - | - | K | D | S | T |
| G16A3 | K | L | D | S | K | R | P | T | S | L | Q | I | P | A | P | K | I | A | D | E | T | T | E | E | E | E | E | E | T | C | - | - | - | - | - | - | E | P | L | I | R | K | N | S | K | I | A | K | S | Q | S | E | T | G | H | M | A | G | G | K |
| G11A3 | T | P | V | H | S | P | I | K | P | G | T | S | G | P | P | P | I | G | E | Y | Y | Q | Q | G | K | K | N | A | A | Q | N | L | P | T | G | Q | S | A | T | P | R | T | V | I | R | V | P | D | N | L | E | D | V | F | A | P | K | E | Q | T |
|  |  |  |  |  |  |  |  |  |  |  |  |  |  |  |  |  |  |  |  |  |  |  |  |  |  |  |  |  |  |  |  |  |  |  |  |  |  |  |  |  |  |  |  |  |  |  |  |  |  |  |  |  |  |  |  |  |  |  |  |  |
| G13E11 | P | S | K | L | Q |  |  |  |  |  |  |  |  |  |  |  |  |  |  |  |  |  |  |  |  |  |  |  |  |  |  |  |  |  |  |  |  |  |  |  |  |  |  |  |  |  |  |  |  |  |  |  |  |  |  |  |  |  |  |  |
| G16A3 | P | N | A | R | K |  |  |  |  |  |  |  |  |  |  |  |  |  |  |  |  |  |  |  |  |  |  |  |  |  |  |  |  |  |  |  |  |  |  |  |  |  |  |  |  |  |  |  |  |  |  |  |  |  |  |  |  |  |  |  |
| G11A3 | G | S | S | N | N |  |  |  |  |  |  |  |  |  |  |  |  |  |  |  |  |  |  |  |  |  |  |  |  |  |  |  |  |  |  |  |  |  |  |  |  |  |  |  |  |  |  |  |  |  |  |  |  |  |  |  |  |  |  |  |

**E**

**5’-UTR ├ SPCR**

G7H5 AATATGATTTTGAATTTGGTTCAAA**ATG**AAACCGTCTCCGTTCATTGTTTT---GATATT
G8C4 -GTATAATCTTGAATTTGGTTCAAA**ATG**AAACTGTCTCTGTTCATTATTTTTTTGATATT

**├MPCR**

G7H5 TGCTGTTATTATAGGCCTGTGTGGTTGTGCACCACCCAAGGCCGAAGAAACTCAATCTGC
G8C4 TGCTGTTATTATAGGCCTGTGTGGTTGTGCACCACCCAAGGCCGAAGGAACTAAATCTGG

G7H5 TACGAGTACGAAAGCCGAGTCTTCTAATGCGGGTCAGAGCGGAAATCGATA--------T
G8C4 TATGGGAACGCAAGCCGAGTCTTCTAATGCGGGTCAGAGAGGAAGTCGAAACAATGGCAT

G7H5 C-CACCGGTGAAGATGAATTTTGAAAAAGTGTTTACTCCTAGTTTTTGTAAAGGTTTGCA
G8C4 CTCATCGGCGGAGTTGAACTTTGACAGAAT---TTCTCCTGGTTTTATTAAAGGTTTGCG

G7H5 AGATCAGCAATCAAAAATTGAAGAACTTTCGGCAGA-CTTGGAGAGGTTTGAGGGTCAGG
G8C4 TGAAGATCAATCAGGATATGAAAAAGTTG-GAGAGATCTTGAAGAGGGCTCAGGATCAGC

**├ 3’-UTR**

G7H5 AATTGAAGTCAAATTATGGAACATATTCCGACAAAAAGGACCATAAA**TAA**AAATTTGTCC
G8C4 AATTGAAGTCAAATTATGGAAAATATTCCGACAAAAAGGCCCATAAT**TAA**AAATTTGTTC

G7H5 AGCAAAAGATATGGTTGCATAATAAACGCAAATATAATCATACACGCACA
G8C4 AGCAAAAAATTTGGTTGCATAATAAACCCAAAAATAATCATACACGC

**Protein alignment**

G7H5 MKPSPFIV-LIFAVIIGLCGCAPPKAEETQSATSTKAESSNAGQSGNR---YPPVKMNFE
G8C4 MKLSLFIIFLIFAVIIGLCGCAPPKAEGTKSGMGTQAESSNAGQRGSRNNGISSAELNFD

G7H5 KVFTPSFCKGLQDQQSKIEELSADLERFEGQELKSNYGTYSDKKDHK
G8C4 RIS-PGFIKGLREDQSGYEKVGEILKRAQDQQLKSNYGKYSDKKAHN

**F**

**├5’-UTR**

G7d10 -TCAGTTATTCAATCCATTTGAAACGAGTAACAGTTTAATATTT-GAGAAAAA-------
G10c9 -TCAGTTATTCAATTCATTCGAAACGAGTAACAGTTTAATATTTTGAAAAAAATAAAA--
G8a11 ATCAGTTATTCAATTCATTTGAAACGAGTAACAGTTTAATATTT-GAAAAAAATAAAAAA
G9e4 -CCAGTTATTCAATTCATTCGAAACGAGTAACAGTTTAATATTTGAAAAAAAATAAAAA-

**├ SPCR ├ MPCR**
G7d10 ------**ATG**AAAGTCATCATTTTAGCTTTGTTCGCAATCGTTGC-TGTAG-CCTGT-GTT
G10c9 ------**ATG**AAAGTCATCATTTTAGCTTTGTTCGCAATCATTGC-TGTGG-CCTGT-GTG
G8a11 ATAAAA**ATG**AAAGTCATCATTTTAGCTTTGTTCGCAATCATTGCCTGTGTATCTGGCGTA
G9e4 ------**ATG**AAAGTCATCATTTTAGCTTTGTTCGCAATCATTGC--------CTGT-GTA

G7d10 TCAGG---T-CAAGGAGCAGGACTCGCACAGG--ATCCTCTTAAAACAATATCTGAAGCT
G10c9 TCAGG---T-CTACCA-----ACTTCCACTCT---TCCTGTT---CCAGGTTTAGGAGC-
G8a11 CCAACCTTTTCACGTATAGGAACAACTGCTCTTAATACACTTTCATCAATTCCAAAAGTA
G9e4 TCAGG---T-CAAGGACCA--CCTGGTCCTGTTCCACAACCTAGTTTGGTTTTGCAACC-

G7d10 GGTAAATCAGCAACTGATGCAGCAACTGGAGGTGTTAAACCAGCAACTGA----------
G10c9 -----ATCCCTTACTGGTCCA------AG------TATAGAAGCATCTCT----------
G8a11 GAAGCAGCTGTTCCTGACCCAACA---TTACTTGTTCCAGGAGTAGATGCAAAAGT----
G9e4 --ACCACCACAAACCGGTCCT------GGCCCTGTTGCCCCCCCACCACCACCATT----

G7d10 -----------------------TGCAGCAACTG------------AAGCAG--------
G10c9 -----------------------TCCTTT---TG-----------CAAG-----------
G8a11 -----------------------TGCTGTTCCTA--------ATCCAAC----------A
G9e4 --------------------TGGCCCTGTTCCAG-----------CAACATGT-------

G7d10 -------------TTAAACCAGCAACTGATGCAGT------------TAAACCAGCAACT
G10c9 --------------TCTAGGAGCATCTCTTCCA-----------------------AGTT
G8a11 TTACTTGTTCCAGAAGTAGCTGCAAATGTTCCTGTTCCTGACCC-----AACAAA-ACTT
G9e4 GTTCCTGCTCCGTTACCACCATGTAATCTTGAATCATCTGGCTCTGGTCAACCAGCATCT

G7d10 GATGCAGT---------------------------------------------TAAACCA
G10c9 TAGG-AGT----------------------------------------------AAACC-
G8a11 GTTCCAGAA---------------------G----------------------TAGATG-
G9e4 AATTTAGTT-CCA-------TCATC-----------------------GGTTCCATGCCT

G7d10 GCAACCGATGCACTAACTGGAGCTGCTCAAC------------CACTAACTGCAGCTGCT
G10c9 -------------TTGCTGGTCCAAGTCAAG------------CAGCAACTCTTCCT--T
G8a11 -CAAATGTTTCTGTTCCTGACCCAACAAAACTTGT---TCCAAAAGTAAATGCAAATATT
G9e4 ACAAA-----CACCAATTGATGCAAGTCAAGTACCAGCTG-TTCAACAAC--CACCTGTT

G7d10 CAAC---------------------------------------------CACTAACTGCA
G10c9 CCTC-------------------------------------------------AAGTGCA
G8a11 CCTGTATCAG--------------------------TG--------------TAGGAGCT
G9e4 CCAGG------T---------------------------------------CAAGGACCA

**├ 3’-UTR**

G7d10 GCTCTTCCACAAAGCCCATAAACACCAGCATCTACTGACCTTGCA**TAA**AAACATTTCGAT
G10c9 G---AT-------GC-----ATCATCAACTGCTACCGCTATTTCA**TAA**AAACGATTCGAT
G8a11 GGTCTTGGTCGA-GC-----ACCATCAACAT-TCCCG------CA**TAA**AAACGATTCGAT
G9e4 TCTCTC--ACCG-GTCCGGCACCACCAACAACTCCAACCACTGCA**TAA**AAACGATTCGAT

G7d10 TTCAATTCAAAAGTAAAAATTTCATGGAAATCATTGAACTAAACTTTCAATAAAATAAAT
G10c9 TTCAATTCAAAGATAAAAATTTCATGTAATTCATTGAACTAAACTTTCAATAAAATAAAT
G8a11 TCCAATTCAAAGATAAAAATTTCATGTAATTCATTGAACTAACCCTTCAATAAAATAAAT
G9e4 TCCAATTCAAAGATAAAAATTTCATGTAATTCATTGAACTAAACTTTCAATAAAATAAAT

G7d10 TCGAAAGACGAAAATGTCTTTGCAC---
G10c9 TTAAAAGACGAAAATGTCTTTGCATGCT
G8a11 TTGAAAGACGAAAATGTCTTTGC-----
G9e4 TTGAAAGACGAAAATGTC----------

**Protein alignment**

| G7D10 | M | K | V | I | I | L | A | L | F | A | I | V | A | V | A | - | C | V | S | G | Q | G | A | G | L | P | Q | D | P | L | K | T | I | S | E | A | G | K | S | A | T | D | A | A | N | G | G | V | K | S | A | T | E | A | V | - | - | - | - | K |
| --- | --- | --- | --- | --- | --- | --- | --- | --- | --- | --- | --- | --- | --- | --- | --- | --- | --- | --- | --- | --- | --- | --- | --- | --- | --- | --- | --- | --- | --- | --- | --- | --- | --- | --- | --- | --- | --- | --- | --- | --- | --- | --- | --- | --- | --- | --- | --- | --- | --- | --- | --- | --- | --- | --- | --- | --- | --- | --- | --- | --- |
| G10C9 | M | K | V | I | I | L | A | L | F | A | I | I | A | V | A | - | C | V | S | G | - | - | - | - | - | - | L | P | T | S | T | L | P | V | - | - | - | - | - | P | G | L | G | A | S | L | T | G | P | S | I | E | A | S | L | P | F | A | S | L |
| G8A11 | M | K | V | I | I | L | A | L | F | A | I | I | A | - | - | - | C | V | S | G | - | - | - | - | - | - | V | P | T | F | S | R | I | G | - | - | - | - | T | T | A | L | N | T | L | S | S | I | P | K | V | E | A | A | V | P | D | P | T | L |
| G9E4 | M | K | V | I | I | L | A | L | F | A | I | I | A | - | - | - | C | V | S | G | Q | G | P | P | G | P | V | P | Q | P | S | L | V | L | Q | P | P | P | Q | T | G | P | G | P | V | A | P | P | P | P | P | F | G | P | V | P | A | T | C | V |
|  |  |  |  |  |  |  |  |  |  |  |  |  |  |  |  |  |  |  |  |  |  |  |  |  |  |  |  |  |  |  |  |  |  |  |  |  |  |  |  |  |  |  |  |  |  |  |  |  |  |  |  |  |  |  |  |  |  |  |  |  |
| G7D10 | P | A | T | D | A | A | T | E | A | - | - | - | - | - | - | - | - | - | - | - | V | K | P | A | T | E | A | V | K | P | A | T | D | A | L | T | G | G | V | K | S | A | T | D | A |  |  |  |  |  |  |  |  |  |  |  |  |  |  |  |
| G10C9 | G | A | S | - | - | - | - | - | - | - | - | - | - | - | - | - | - | - | - | - | - | L | P | S | L | G | V | N | - | - | - | - | - | - | - | - | - | - | L | A | G | P | S | Q | A |  |  |  |  |  |  |  |  |  |  |  |  |  |  |  |
| G9E4 | P | A | P | L | P | P | C | N | L | E | S | S | G | S | G | Q | P | A | S | N | L | V | P | S | S | V | P | C | - | - | - | - | - | - | - | - | - | - | L | Q | T | P | I | D | A |  |  |  |  |  |  |  |  |  |  |  |  |  |  |  |
| G8A11 | L | V | P | G | V | D | A | K | V | A | V | P | N | - | - | - | - | P | T | L | L | V | P | E | V | A | A | N | - | - | - | - | - | - | - | - | - | - | V | P | V | P | D | P | T |  |  |  |  |  |  |  |  |  |  |  |  |  |  |  |
|  |  |  |  |  |  |  |  |  |  |  |  |  |  |  |  |  |  |  |  |  |  |  |  |  |  |  |  |  |  |  |  |  |  |  |  |  |  |  |  |  |  |  |  |  |  |  |  |  |  |  |  |  |  |  |  |  |  |  |  |  |
| G7D10 | V | K | P | A | - | - | - | - | - | - | - | - | T | D | A | V | K | P | A | T | D | A | L | T | G | - | - | - | - | A | A | Q | P | L | T | A | A | L | P | Q | S | Q |  |  |  |  |  |  |  |  |  |  |  |  |  |  |  |  |  |  |
| G10C9 | A | T | L | P | - | - | - | - | - | - | - | - | - | - | - | - | S | S | S | A | D | A | S | - | - | - | - | - | - | - | - | - | - | - | S | T | A | T | A | I | S | - |  |  |  |  |  |  |  |  |  |  |  |  |  |  |  |  |  |  |
| G9E4 | S | Q | V | P | - | - | - | - | - | - | - | - | - | - | - | - | A | V | Q | Q | P | P | V | P | G | Q | G | P | S | L | T | G | P | A | P | P | T | T | P | T | T | A |  |  |  |  |  |  |  |  |  |  |  |  |  |  |  |  |  |  |
| G8A11 | K | L | V | P | - | - | - | - | - | - | - | - | - | - | - | - | E | V | D | A | N | V | S | V | P | D | P | T | K | L | V | P | K | V | N | A | N | I | P | V | S | V |  |  |  |  |  |  |  |  |  |  |  |  |  |  |  |  |  |  |

**G**

**├5’-UTR ├ SPCR**

| OO4O12 | - | - | - | - | - | - | - | - | - | - | - | - | - | - | - | - | - | - | - | - | - | - | - | - | - | - | - | - | - | - | - | - | - | - | - | - | - | - | - | - | - | A | T | T | T | T | G | T | G | C | T | C | A | A | C | A | A | T | T | T |
| --- | --- | --- | --- | --- | --- | --- | --- | --- | --- | --- | --- | --- | --- | --- | --- | --- | --- | --- | --- | --- | --- | --- | --- | --- | --- | --- | --- | --- | --- | --- | --- | --- | --- | --- | --- | --- | --- | --- | --- | --- | --- | --- | --- | --- | --- | --- | --- | --- | --- | --- | --- | --- | --- | --- | --- | --- | --- | --- | --- | --- |
| OO4N5 | C | A | A | A | A | A | A | A | A | A | A | A | A | A | A | C | A | A | A | A | A | T | T | G | T | C | **A** | **T** | **G** | A | A | A | T | T | T | C | C | C | A | C | C | A | T | T | T | T | G | G | G | C | G | C | A | G | C | G | A | T | T | T |

**├ MPCR**

| OO4O12 | T | G | C | T | T | C | T | A | G | T | A | G | G | A | A | T | T | T | G | T | G | A | A | G | G | A | G | C | A | A | G | C | T | C | A | A | G | T | T | C | A | A | G | C | A | T | A | A | A | A | C | G | G | A | G | A | C | A | T | A |
| --- | --- | --- | --- | --- | --- | --- | --- | --- | --- | --- | --- | --- | --- | --- | --- | --- | --- | --- | --- | --- | --- | --- | --- | --- | --- | --- | --- | --- | --- | --- | --- | --- | --- | --- | --- | --- | --- | --- | --- | --- | --- | --- | --- | --- | --- | --- | --- | --- | --- | --- | --- | --- | --- | --- | --- | --- | --- | --- | --- | --- |
| OO4N5 | T | G | C | T | T | C | T | A | G | T | A | G | G | C | A | T | A | T | G | T | G | A | A | G | G | A | G | C | A | C | G | C | T | C | A | A | G | T | T | C | A | G | G | C | A | T | A | A | G | A | C | G | G | A | A | G | G | G | T | A |

| OO4O12 | C | C | C | T | T | G | A | G | G | A | G | - | - | - | - | - | - | - | - | - | - | - | - | - | - | - | - | - | - | T | C | T | C | A | T | G | A | A | G | G | A | G | A | G | A | T | C | G | C | - | - | - | - | A | A | T | - | - | - | - |
| --- | --- | --- | --- | --- | --- | --- | --- | --- | --- | --- | --- | --- | --- | --- | --- | --- | --- | --- | --- | --- | --- | --- | --- | --- | --- | --- | --- | --- | --- | --- | --- | --- | --- | --- | --- | --- | --- | --- | --- | --- | --- | --- | --- | --- | --- | --- | --- | --- | --- | --- | --- | --- | --- | --- | --- | --- | --- | --- | --- | --- |
| OO4N5 | A | T | C | G | T | G | G | A | G | A | A | A | A | A | G | A | C | A | G | T | A | A | T | A | C | T | A | T | A | C | T | C | T | C | T | G | A | A | C | A | T | G | A | A | A | T | C | T | C | T | G | A | A | A | A | T | G | A | A | G |

| OO4O12 | - | - | - | - | T | G | A | A | A | T | T | G | A | A | G | T | C | C | C | T | G | A | T | C | G | C | C | C | T | A | A | T | G | T | T | A | A | A | G | A | - | - | - | G | G | T | C | T | G | C | T | G | T | A | A | G | G | A | T | A |
| --- | --- | --- | --- | --- | --- | --- | --- | --- | --- | --- | --- | --- | --- | --- | --- | --- | --- | --- | --- | --- | --- | --- | --- | --- | --- | --- | --- | --- | --- | --- | --- | --- | --- | --- | --- | --- | --- | --- | --- | --- | --- | --- | --- | --- | --- | --- | --- | --- | --- | --- | --- | --- | --- | --- | --- | --- | --- | --- | --- | --- |
| OO4N5 | T | C | T | C | T | G | A | A | A | C | G | G | A | A | G | T | C | T | C | T | G | A | C | A | A | T | G | A | A | G | T | C | C | C | T | G | A | A | A | A | T | A | T | A | G | T | C | T | G | T | C | C | T | G | T | G | A | A | T | G |

| OO4O12 | A | A | A | A | T | G | G | G | T | T | C | A | T | T | A | A | A | A | A | A | T | T | T | C | A | T | T | G | G | A | T | A | A | A | T | G | G | A | C | T | T | C | A | T | T | G | C | G | C | G | G | A | A | G | C | C | G | C | G | T |
| --- | --- | --- | --- | --- | --- | --- | --- | --- | --- | --- | --- | --- | --- | --- | --- | --- | --- | --- | --- | --- | --- | --- | --- | --- | --- | --- | --- | --- | --- | --- | --- | --- | --- | --- | --- | --- | --- | --- | --- | --- | --- | --- | --- | --- | --- | --- | --- | --- | --- | --- | --- | --- | --- | --- | --- | --- | --- | --- | --- | --- |
| OO4N5 | A | A | A | C | T | G | G | A | A | C | T | G | C | A | A | T | C | A | T | T | C | A | T | G | A | T | A | A | G | T | T | T | T | T | T | A | A | T | A | T | T | C | G | T | C | G | C | G | C | G | A | T | A | G | G | A | A | C | T | T |

| OO4O12 | G | C | G | T | C | A | C | A | T | T | T | T | C | T | G | C | T | A | T | G | G | T | C | G | C | A | G | C | C | A | A | A | T | A | C | A | C | A | G | T | G | G | A | T | T | T | C | T | G | T | G | G | A | A | A | T | C | C | G | A |
| --- | --- | --- | --- | --- | --- | --- | --- | --- | --- | --- | --- | --- | --- | --- | --- | --- | --- | --- | --- | --- | --- | --- | --- | --- | --- | --- | --- | --- | --- | --- | --- | --- | --- | --- | --- | --- | --- | --- | --- | --- | --- | --- | --- | --- | --- | --- | --- | --- | --- | --- | --- | --- | --- | --- | --- | --- | --- | --- | --- | --- |
| OO4N5 | G | C | T | C | A | G | T | A | A | T | T | T | T | A | G | C | T | G | C | A | G | C | A | A | C | A | G | G | T | G | A | A | C | A | A | T | T | C | A | T | T | A | A | G | T | T | C | T | G | T | C | A | A | A | A | T | T | T | G | G |

| OO4O12 | A | T | G | A | A | A | A | T | G | A | G | A | G | G | G | C | C | G | A | A | T | G | C | C | T | T | A | A | T | T | A | T | A | T | C | T | C | T | A | T | T | G | C | T | A | G | T | A | C | G | A | T | T | C | C | T | A | T | T | G |
| --- | --- | --- | --- | --- | --- | --- | --- | --- | --- | --- | --- | --- | --- | --- | --- | --- | --- | --- | --- | --- | --- | --- | --- | --- | --- | --- | --- | --- | --- | --- | --- | --- | --- | --- | --- | --- | --- | --- | --- | --- | --- | --- | --- | --- | --- | --- | --- | --- | --- | --- | --- | --- | --- | --- | --- | --- | --- | --- | --- | --- |
| OO4N5 | A | T | G | A | A | G | A | A | A | C | G | A | A | G | A | A | T | A | A | A | T | G | C | G | T | G | A | A | C | T | A | T | G | C | C | A | C | T | T | T | T | G | C | T | G | C | T | G | C | T | G | C | A | G | G | A | A | T | T | A |

| OO4O12 | C | C | T | C | C | T | C | T | A | T | G | A | A | C | A | A | T | T | T | G | G | T | A | G | G | A | G | A | A | C | C | A | A | G | C | G | G | C | C | T | C | G | T | G | C | G | T | A | A | G | G | - | - | - | T | G | A | A | G | C |
| --- | --- | --- | --- | --- | --- | --- | --- | --- | --- | --- | --- | --- | --- | --- | --- | --- | --- | --- | --- | --- | --- | --- | --- | --- | --- | --- | --- | --- | --- | --- | --- | --- | --- | --- | --- | --- | --- | --- | --- | --- | --- | --- | --- | --- | --- | --- | --- | --- | --- | --- | --- | --- | --- | --- | --- | --- | --- | --- | --- | --- |
| OO4N5 | C | A | A | C | G | T | T | T | T | C | G | A | A | T | A | T | T | T | G | G | G | A | A | T | C | A | A | T | G | G | G | T | A | A | T | G | A | A | A | C | T | T | T | T | C | G | T | A | A | G | C | G | T | C | T | C | C | C | G | C |

| OO4O12 | T | T | T | G | G | T | G | T | C | A | G | A | A | A | A | C | T | G | C | T | A | A | C | G | T | G | A | T | T | G | T | T | G | T | T | G | T | G | G | C | T | G | G | T | T | T | T | G | C | G | A | T | C | A | C | T | T | G | T | G |
| --- | --- | --- | --- | --- | --- | --- | --- | --- | --- | --- | --- | --- | --- | --- | --- | --- | --- | --- | --- | --- | --- | --- | --- | --- | --- | --- | --- | --- | --- | --- | --- | --- | --- | --- | --- | --- | --- | --- | --- | --- | --- | --- | --- | --- | --- | --- | --- | --- | --- | --- | --- | --- | --- | --- | --- | --- | --- | --- | --- | --- |
| OO4N5 | G | T | C | T | T | T | G | T | A | A | G | G | C | T | T | T | T | A | T | T | G | G | C | T | C | C | A | T | G | A | T | T | G | T | T | G | G | A | A | C | T | A | T | T | A | C | T | G | C | T | T | A | C | G | C | T | T | G | T | G |

**├ 3’-UTR**

| OO4O12 | A | G | C | T | C | T | T | G | A | A | G | C | C | A | C | A | T | T | T | T | T | A | C | G | G | A | **T** | **A** | **A** | G | C | A | T | A | G | A | T | A | T | C | A | G | A | T | T | G | A | T | A | A | A | C | A | T | T | T | A | C | A | T |
| --- | --- | --- | --- | --- | --- | --- | --- | --- | --- | --- | --- | --- | --- | --- | --- | --- | --- | --- | --- | --- | --- | --- | --- | --- | --- | --- | --- | --- | --- | --- | --- | --- | --- | --- | --- | --- | --- | --- | --- | --- | --- | --- | --- | --- | --- | --- | --- | --- | --- | --- | --- | --- | --- | --- | --- | --- | --- | --- | --- | --- |
| OO4N5 | A | C | A | G | G | A | T | A | A | A | A | C | A | A | C | A | A | G | C | T | A | A | C | G | - | - | - | - | - | - | - | - | - | - | - | - | - | - | - | C | A | G | G | T | A | G | A | **T** | **A** | **G** | A | C | A | T | C | T | - | - | - | - |

| OO4O12 | A | T | A | T | A | T | C | T | A | T | A | C | A | G | A | A | A | A | A | G | T | G | T | T | T | C | T | A | T | T | T | T | A | A | A | C | A | C | T | G | A | A | A | A | G | G | A | A | A | C | A | C | T | T | T | A | C | T | G | - |
| --- | --- | --- | --- | --- | --- | --- | --- | --- | --- | --- | --- | --- | --- | --- | --- | --- | --- | --- | --- | --- | --- | --- | --- | --- | --- | --- | --- | --- | --- | --- | --- | --- | --- | --- | --- | --- | --- | --- | --- | --- | --- | --- | --- | --- | --- | --- | --- | --- | --- | --- | --- | --- | --- | --- | --- | --- | --- | --- | --- | --- |
| OO4N5 | - | T | A | T | A | T | C | T | T | G | A | A | A | A | A | A | A | A | A | C | C | G | T | A | T | C | T | A | T | T | T | T | A | A | A | C | A | C | T | G | G | A | A | G | G | A | A | A | A | A | C | C | T | A | A | A | T | C | G | C |

| OO4O12 | - | A | C | A | G | - | - | - | A | T | C | A | G | A | A | A | A | T | A | A | C | A | T | G | A | T | T | C | T | A | A | C | C | T | T | T | T | A | C | C | C | A | T | G | A | G | T | T | T | T | G | T | A | T | C | T | T | A | A | A |
| --- | --- | --- | --- | --- | --- | --- | --- | --- | --- | --- | --- | --- | --- | --- | --- | --- | --- | --- | --- | --- | --- | --- | --- | --- | --- | --- | --- | --- | --- | --- | --- | --- | --- | --- | --- | --- | --- | --- | --- | --- | --- | --- | --- | --- | --- | --- | --- | --- | --- | --- | --- | --- | --- | --- | --- | --- | --- | --- | --- | --- |
| OO4N5 | G | A | C | A | G | T | A | T | A | T | C | A | G | A | A | A | A | T | A | - | C | A | T | A | G | T | T | C | T | A | A | C | C | T | T | T | T | A | C | C | C | A | T | G | A | T | T | T | T | T | G | A | A | T | T | T | T | A | A | A |

| OO4O12 | A | A | A | T | - | - | - | - | - | - | - | - | - | - | - | - | - | - | - | - | - | - | - | - | - | - | - | - | - | - | - | - | - | - | - | - |  |  |  |  |  |  |  |  |  |  |  |  |  |  |  |  |  |  |  |  |  |  |  |  |
| --- | --- | --- | --- | --- | --- | --- | --- | --- | --- | --- | --- | --- | --- | --- | --- | --- | --- | --- | --- | --- | --- | --- | --- | --- | --- | --- | --- | --- | --- | --- | --- | --- | --- | --- | --- | --- | --- | --- | --- | --- | --- | --- | --- | --- | --- | --- | --- | --- | --- | --- | --- | --- | --- | --- | --- | --- | --- | --- | --- | --- |
| OO4N5 | A | T | A | T | A | T | T | T | G | A | T | G | A | A | A | T | A | A | A | G | T | T | A | A | A | T | A | G | A | A | C | T | T | T | G | A |  |  |  |  |  |  |  |  |  |  |  |  |  |  |  |  |  |  |  |  |  |  |  |  |

**Protein alignment**

| OO4O12 | - | - | - | - | - | I | L | C | S | T | I | L | L | L | V | G | I | C | E | G | A | S | S | S | S | S | I | K | R | R | H | T | L | E | E | S | H | E | G | E | I | A | I | E | I | E | V | P | D | R | - | - | - | - | - | - | - | - | - | - |
| --- | --- | --- | --- | --- | --- | --- | --- | --- | --- | --- | --- | --- | --- | --- | --- | --- | --- | --- | --- | --- | --- | --- | --- | --- | --- | --- | --- | --- | --- | --- | --- | --- | --- | --- | --- | --- | --- | --- | --- | --- | --- | --- | --- | --- | --- | --- | --- | --- | --- | --- | --- | --- | --- | --- | --- | --- | --- | --- | --- | --- |
| OO4N5 | M | K | F | P | T | I | L | G | A | A | I | L | L | L | V | G | I | C | E | G | A | R | S | S | S | G | I | R | R | K | G | N | R | G | E | K | D | S | N | T | I | L | S | E | H | E | I | S | E | N | E | V | S | E | T | E | V | S | D | N |

| OO4O12 | - | P | N | V | K | E | V | C | C | K | D | K | N | G | F | I | K | K | F | H | W | I | N | G | L | H | C | A | E | A | A | C | V | T | F | S | A | M | V | A | A | K | Y | T | V | D | F | C | G | N | P | N | E | N | E | R | A | E | C | L |
| --- | --- | --- | --- | --- | --- | --- | --- | --- | --- | --- | --- | --- | --- | --- | --- | --- | --- | --- | --- | --- | --- | --- | --- | --- | --- | --- | --- | --- | --- | --- | --- | --- | --- | --- | --- | --- | --- | --- | --- | --- | --- | --- | --- | --- | --- | --- | --- | --- | --- | --- | --- | --- | --- | --- | --- | --- | --- | --- | --- | --- |
| OO4N5 | E | V | P | E | N | I | V | C | P | V | N | E | T | G | T | A | I | I | H | D | K | F | F | N | I | R | R | A | I | G | T | C | S | V | I | L | A | A | A | T | G | E | Q | F | I | K | F | C | Q | N | L | D | E | E | T | K | N | K | C | V |

| OO4O12 | N | Y | I | S | I | A | S | T | I | P | I | A | S | S | M | N | N | L | V | G | E | - | P | S | G | L | V | R | K | V | K | L | W | C | Q | K | T | A | N | V | I | V | V | V | A | G | F | A | I | T | C | E | L | L | K | P | H | F | Y | G |
| --- | --- | --- | --- | --- | --- | --- | --- | --- | --- | --- | --- | --- | --- | --- | --- | --- | --- | --- | --- | --- | --- | --- | --- | --- | --- | --- | --- | --- | --- | --- | --- | --- | --- | --- | --- | --- | --- | --- | --- | --- | --- | --- | --- | --- | --- | --- | --- | --- | --- | --- | --- | --- | --- | --- | --- | --- | --- | --- | --- | --- |
| OO4N5 | N | Y | A | T | F | A | A | A | A | G | I | T | T | F | S | N | I | W | E | S | M | G | N | E | T | F | R | K | R | L | P | R | L | C | K | A | F | I | G | S | M | I | V | G | T | I | T | A | Y | A | C | D | R | I | K | Q | Q | A | N | A |

| OO4O12 | - | - |  |  |  |  |  |  |  |  |  |  |  |  |  |  |  |  |  |  |  |  |  |  |  |  |  |  |  |  |  |  |  |  |  |  |  |  |  |  |  |  |  |  |  |  |  |  |  |  |  |  |  |  |  |  |  |  |  |  |
| --- | --- | --- | --- | --- | --- | --- | --- | --- | --- | --- | --- | --- | --- | --- | --- | --- | --- | --- | --- | --- | --- | --- | --- | --- | --- | --- | --- | --- | --- | --- | --- | --- | --- | --- | --- | --- | --- | --- | --- | --- | --- | --- | --- | --- | --- | --- | --- | --- | --- | --- | --- | --- | --- | --- | --- | --- | --- | --- | --- | --- |
| OO4N5 | G | R |  |  |  |  |  |  |  |  |  |  |  |  |  |  |  |  |  |  |  |  |  |  |  |  |  |  |  |  |  |  |  |  |  |  |  |  |  |  |  |  |  |  |  |  |  |  |  |  |  |  |  |  |  |  |  |  |  |  |

Figure S2. Nucleotide and predicted amino acid sequence alignments of SSSGP-encoding cDNAs from other SSSGP gene families. **A:** Alignments of cDNAs L5E4, W12F4, G2D9, G3H12, W8E4, G12G1, S18E7, G21F11, G7F4, G10H7, W7F8, W16A21, W6C7, G12H12, and W1C2, and their predicted proteins from the *SSSGP-2* family. **B:** Alignments of cDNAs G12C4, G21F1, G8B10, and G15B4, and their predicted proteins from the *SSSGP-3* family. **C:** Alignments of cDNAs G15B9, G9A12, G11C8, G28G10, S19E7, G6D3, and S10A9, and their predicted proteins from the *SSSGP-4* family. **D:** Alignments of cDNAs G13E11, G16A3, and G11A3, and their predicted proteins from the *SSSGP-5* family. **E:** Alignments of cDNAs G7H5 and G8C4, and their predicted proteins from the *SSSGP-6* family. **F**: Aligments of cDNAs G7D10, G10C9, G8A11, and G9E4, their predicted proteins from the *SSSGP-31* family. **G:** Alignments of two cDNAs OO4O12 and OO4N5, and their predicted proteins from the Asian rice midge. Abbreviations and methods for alignments were the same as in Fig. S1.
