# Supplementary material for: Unusual conservation among genes encoding small secreted salivary gland proteins from a gall midge
Source: BMC Evol Biol. 2010 Sep 28;10:296. doi: 10.1186/1471-2148-10-296 (PMC2955719; doi:10.1186/1471-2148-10-296)
Supplement: Additional file 3 — Figure S3: Evidence for clustered organization of SSSGP-encoding genes. [file 1471-2148-10-296-S3.DOC]

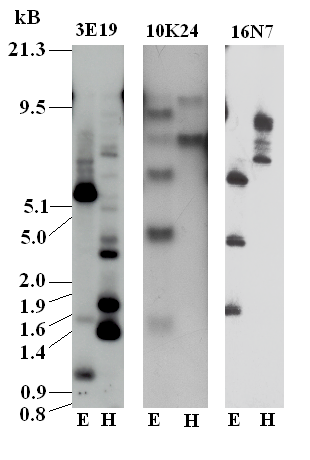


Figure S3. Southern blots of BAC clones 3E19, 10K24, and 16N7 that hybridized to cDNA probes corresponding to *SSSGP-4*, *SSSGP-31*, and *SSSGP-8*, respectively. BAC DNA samples were digested with EcoR1 (E) or Hind III (H), separated on a 0.8% agarose gel, and hybridized to a ^32^P-labelled cDNA probe. DNA marker sizes were given on the left.
