# Supplementary material for: Unusual conservation among genes encoding small secreted salivary gland proteins from a gall midge
Source: BMC Evol Biol. 2010 Sep 28;10:296. doi: 10.1186/1471-2148-10-296 (PMC2955719; doi:10.1186/1471-2148-10-296)
Supplement: Additional file 4 — Figure S4: Alignments of moderately diversified SSSGP group members (cDNAs). [file 1471-2148-10-296-S4.DOC]

**A**

| SSGP_1C1 | A | C | A | G | T | T | C | C | A | A | T | T | G | A | A | C | C | G | T | T | T | A | T | T | C | T | T | T | C | G | A | T | A | T | C | C | A | C | T | G | G | A | A | C | A | T | C | C | A | A | A | A | A | - | C | G | A | A | A | **A** |
| --- | --- | --- | --- | --- | --- | --- | --- | --- | --- | --- | --- | --- | --- | --- | --- | --- | --- | --- | --- | --- | --- | --- | --- | --- | --- | --- | --- | --- | --- | --- | --- | --- | --- | --- | --- | --- | --- | --- | --- | --- | --- | --- | --- | --- | --- | --- | --- | --- | --- | --- | --- | --- | --- | --- | --- | --- | --- | --- | --- | --- |
| SSGP_1C2 | A | C | A | G | T | T | C | G | A | A | T | T | A | A | A | C | C | G | T | T | A | A | T | T | C | T | T | T | C | A | A | T | A | T | C | C | A | C | T | G | G | A | A | C | A | T | C | C | A | A | A | A | A | A | C | G | A | A | A | **A** |

***M S K F F L A F A I I A V C L V A A*** *Q A*

| SSGP_1C1 | **T** | **G** | T | C | A | A | A | A | T | T | T | T | T | C | T | T | A | G | C | T | T | T | C | G | C | C | A | T | C | A | T | C | G | C | C | G | T | C | T | G | C | C | T | T | G | T | T | G | C | A | G | C | T | C | A | G | G | C | T | G |
| --- | --- | --- | --- | --- | --- | --- | --- | --- | --- | --- | --- | --- | --- | --- | --- | --- | --- | --- | --- | --- | --- | --- | --- | --- | --- | --- | --- | --- | --- | --- | --- | --- | --- | --- | --- | --- | --- | --- | --- | --- | --- | --- | --- | --- | --- | --- | --- | --- | --- | --- | --- | --- | --- | --- | --- | --- | --- | --- | --- | --- |
| SSGP_1C2 | **T** | **G** | T | C | A | A | A | A | T | T | T | T | T | A | T | T | A | G | C | T | T | T | C | G | C | C | A | T | C | A | T | C | G | C | C | G | T | C | T | G | C | C | T | T | G | T | T | G | C | A | G | C | T | C | A | G | G | C | T | G |

***M S K F L L A F A I I A V C L V A A*** Q A

V H S S H S T A A H **A** **G** **K** **T** H E P A K

| SSGP_1C1 | - | - | - | T | A | C | A | C | A | G | C | A | G | C | C | A | T | T | C | C | A | C | A | G | C | T | G | C | A | C | A | C | G | C | T | G | G | T | A | A | A | A | C | C | C | A | T | G | A | G | C | C | A | G | C | A | A | A | A | A |
| --- | --- | --- | --- | --- | --- | --- | --- | --- | --- | --- | --- | --- | --- | --- | --- | --- | --- | --- | --- | --- | --- | --- | --- | --- | --- | --- | --- | --- | --- | --- | --- | --- | --- | --- | --- | --- | --- | --- | --- | --- | --- | --- | --- | --- | --- | --- | --- | --- | --- | --- | --- | --- | --- | --- | --- | --- | --- | --- | --- | --- |
| SSGP_1C2 | C | T | G | T | A | C | A | C | A | G | C | A | G | C | C | A | T | T | C | C | A | C | A | G | C | T | G | C | A | C | A | C | G | C | A | G | G | A | A | A | T | A | T | C | C | A | T | G | - | - | - | - | - | - | C | A | G | C | A | A |

A V H S S H S T A A H **A** **G** **N** **I** H A A

T **Q** T A Q **P** **G** **V** **G** **D** **H** L D **D** **G** E H Y F D

| SSGP_1C1 | C | G | C | A | A | A | C | A | G | C | C | C | A | A | C | C | A | G | G | C | G | T | A | G | G | T | G | A | T | C | A | T | C | T | T | G | A | C | G | A | T | G | G | T | G | A | G | C | A | C | T | A | T | T | T | C | G | A | C | G |
| --- | --- | --- | --- | --- | --- | --- | --- | --- | --- | --- | --- | --- | --- | --- | --- | --- | --- | --- | --- | --- | --- | --- | --- | --- | --- | --- | --- | --- | --- | --- | --- | --- | --- | --- | --- | --- | --- | --- | --- | --- | --- | --- | --- | --- | --- | --- | --- | --- | --- | --- | --- | --- | --- | --- | --- | --- | --- | --- | --- | --- |
| SSGP_1C2 | C | G | C | A | T | A | C | A | G | C | C | C | A | A | C | T | A | A | G | C | C | T | A | G | C | T | G | A | C | G | A | T | C | T | T | G | A | C | G | A | A | G | A | T | G | A | G | C | A | C | T | G | G | T | T | C | G | A | C | G |

T **H** T A Q **L** **S** **L** **A** **D** **D** L D **E** **D** E H W F D

A S D K D F E S Y F E D D N E P N E A A

| SSGP_1C1 | C | A | T | C | G | G | A | C | A | A | A | G | A | T | T | T | C | G | A | A | T | C | A | T | A | T | T | T | C | G | A | A | G | A | T | G | A | T | A | A | T | G | A | G | C | C | T | A | A | T | G | A | A | G | C | A | G | C | C | G |
| --- | --- | --- | --- | --- | --- | --- | --- | --- | --- | --- | --- | --- | --- | --- | --- | --- | --- | --- | --- | --- | --- | --- | --- | --- | --- | --- | --- | --- | --- | --- | --- | --- | --- | --- | --- | --- | --- | --- | --- | --- | --- | --- | --- | --- | --- | --- | --- | --- | --- | --- | --- | --- | --- | --- | --- | --- | --- | --- | --- | --- |
| SSGP_1C2 | C | A | T | C | G | G | A | C | A | A | A | G | A | T | T | T | C | G | A | A | T | C | A | T | A | T | T | T | C | G | A | A | G | A | T | G | A | T | A | A | T | G | G | C | C | C | T | A | A | T | G | A | A | G | C | A | G | C | C | G |

A S D K D F E S Y F E D D N G P N E A A

V P A G K P T K P L K A K **T** S P K G K G

| SSGP_1C1 | T | A | C | C | G | G | C | A | G | G | A | A | A | A | C | C | A | A | C | A | A | A | A | C | C | A | T | T | G | A | A | G | G | C | C | A | A | A | A | C | A | T | C | G | C | C | A | A | A | A | G | G | A | A | A | A | G | G | C | C |
| --- | --- | --- | --- | --- | --- | --- | --- | --- | --- | --- | --- | --- | --- | --- | --- | --- | --- | --- | --- | --- | --- | --- | --- | --- | --- | --- | --- | --- | --- | --- | --- | --- | --- | --- | --- | --- | --- | --- | --- | --- | --- | --- | --- | --- | --- | --- | --- | --- | --- | --- | --- | --- | --- | --- | --- | --- | --- | --- | --- | --- |
| SSGP_1C2 | T | A | C | C | G | G | C | A | G | G | A | A | A | A | C | C | A | A | C | A | A | A | A | C | C | A | T | T | G | A | A | G | G | C | C | A | A | A | C | C | A | T | C | G | C | C | A | C | A | T | G | G | A | A | A | A | G | G | C | C |

V P A G K P T K P L K A K **P** S P H G K G

H Q K K

| SSGP_1C1 | A | C | C | A | A | A | A | A | A | A | A | **T** | **G** | **A** | T | C | A | T | T | T | C | A | T | T | C | A | A | T | T | G | A | A | A | G | A | A | C | A | T | T | T | G | G | A | G | G | C | G | T | C | G | C | G | T | G | T | A | A | C | C |
| --- | --- | --- | --- | --- | --- | --- | --- | --- | --- | --- | --- | --- | --- | --- | --- | --- | --- | --- | --- | --- | --- | --- | --- | --- | --- | --- | --- | --- | --- | --- | --- | --- | --- | --- | --- | --- | --- | --- | --- | --- | --- | --- | --- | --- | --- | --- | --- | --- | --- | --- | --- | --- | --- | --- | --- | --- | --- | --- | --- | --- |
| SSGP_1C2 | A | C | C | A | A | A | A | A | A | A | G | **T** | **A** | **A** | T | C | A | T | T | T | C | A | T | T | C | A | A | T | T | G | A | A | A | G | A | A | C | A | T | T | T | G | G | A | G | G | C | G | T | C | G | C | G | T | G | T | A | A | C | C |

H Q K K

| SSGP_1C1 | A | A | A | A | T | T | A | T | A | T | A | G | T | T | A | T | A | C | A | C | T | C | T | C | A | T | A | T | T | T | T | C | A | A | T | T | T | G | T | C | T | A | T | T | G | A | A | A | T | C | A | A | T | T | C | T | T | G | A | A |
| --- | --- | --- | --- | --- | --- | --- | --- | --- | --- | --- | --- | --- | --- | --- | --- | --- | --- | --- | --- | --- | --- | --- | --- | --- | --- | --- | --- | --- | --- | --- | --- | --- | --- | --- | --- | --- | --- | --- | --- | --- | --- | --- | --- | --- | --- | --- | --- | --- | --- | --- | --- | --- | --- | --- | --- | --- | --- | --- | --- | --- |
| SSGP_1C2 | A | A | A | A | T | T | A | A | A | T | A | G | T | T | A | T | A | T | G | C | T | C | T | C | A | T | A | A | A | T | T | C | A | A | T | T | T | G | T | C | A | A | T | T | G | A | A | A | T | C | A | A | T | T | C | T | T | G | A | A |

| SSGP_1C1 | A | T | T | A | A | A | T | T | C | T | T | G | A | A | T | G | T | A | A | A | A | A | A | A | A | A | A | A | - | - | - | - | - | - | - | - | - | - | - | - | A | T | A | A | T | T | G | A | G | T | A | T | T | T | T | A | T | T | C | A |
| --- | --- | --- | --- | --- | --- | --- | --- | --- | --- | --- | --- | --- | --- | --- | --- | --- | --- | --- | --- | --- | --- | --- | --- | --- | --- | --- | --- | --- | --- | --- | --- | --- | --- | --- | --- | --- | --- | --- | --- | --- | --- | --- | --- | --- | --- | --- | --- | --- | --- | --- | --- | --- | --- | --- | --- | --- | --- | --- | --- | --- |
| SSGP_1C2 | A | T | T | A | A | A | T | T | C | T | T | G | A | A | T | G | T | A | A | A | A | A | A | A | A | A | A | A | T | A | A | A | A | T | A | A | A | A | T | A | A | T | A | A | T | T | G | A | G | T | A | T | T | T | T | A | T | T | C | A |

| SSGP_1C1 | G | A | A | C | A | A | T | C | T | C | C | A | C | C | A | T | T | C | T | T | A | T | T | C | A | T | C | C | A | T | A | G | G | - | T | A | A | - | - | - | - | - | T | C | T | C | T | T | T | A | G | T | T | A | T | A | A | T | T | T |
| --- | --- | --- | --- | --- | --- | --- | --- | --- | --- | --- | --- | --- | --- | --- | --- | --- | --- | --- | --- | --- | --- | --- | --- | --- | --- | --- | --- | --- | --- | --- | --- | --- | --- | --- | --- | --- | --- | --- | --- | --- | --- | --- | --- | --- | --- | --- | --- | --- | --- | --- | --- | --- | --- | --- | --- | --- | --- | --- | --- | --- |
| SSGP_1C2 | G | A | A | C | G | A | T | C | T | C | C | A | C | C | A | T | T | C | T | T | A | T | T | C | T | T | C | C | A | T | A | G | G | A | T | A | A | G | G | T | C | T | T | T | C | C | T | T | T | A | G | T | T | G | T | T | A | T | T | T |

| SSGP_1C1 | T | G | T | A | C | A | A | A | T | C | A | T | G | A | A | C | A | A | G | T | T | C | G | A | T | G | A | T | T | A |  |  |  |  |  |  |  |  |  |  |  |  |  |  |  |  |  |  |  |  |  |  |  |  |  |  |  |  |  |  |
| --- | --- | --- | --- | --- | --- | --- | --- | --- | --- | --- | --- | --- | --- | --- | --- | --- | --- | --- | --- | --- | --- | --- | --- | --- | --- | --- | --- | --- | --- | --- | --- | --- | --- | --- | --- | --- | --- | --- | --- | --- | --- | --- | --- | --- | --- | --- | --- | --- | --- | --- | --- | --- | --- | --- | --- | --- | --- | --- | --- | --- |
| SSGP_1C2 | T | G | T | T | C | A | A | A | T | C | A | T | A | A | A | C | A | A | G | T | T | C | G | A | A | A | A | T | T | T |  |  |  |  |  |  |  |  |  |  |  |  |  |  |  |  |  |  |  |  |  |  |  |  |  |  |  |  |  |  |

**B**

M G K Y H

| G19B4 | G | T | T | C | A | T | C | A | G | A | A | C | A | T | T | C | T | A | T | T | C | A | T | T | G | C | A | G | T | A | T | A | A | C | A | G | T | T | G | T | C | G | A | A | **A** | **T** | **G** | G | G | A | A | A | A | T | A | T | C | A | T | C |
| --- | --- | --- | --- | --- | --- | --- | --- | --- | --- | --- | --- | --- | --- | --- | --- | --- | --- | --- | --- | --- | --- | --- | --- | --- | --- | --- | --- | --- | --- | --- | --- | --- | --- | --- | --- | --- | --- | --- | --- | --- | --- | --- | --- | --- | --- | --- | --- | --- | --- | --- | --- | --- | --- | --- | --- | --- | --- | --- | --- | --- |
| S21C6 | G | T | T | C | A | A | C | T | G | A | A | C | A | T | T | C | T | G | A | T | C | A | T | T | G | T | A | G | T | A | T | A | A | C | A | G | A | T | G | T | C | A | A | A | **A** | **T** | **G** | G | G | A | A | A | A | T | A | T | C | A | T | T |

***M G K Y H***

***L I V L L I F L V V C*** S **E** T L A T G D G

| G19B4 | T | A | A | T | A | G | T | T | T | T | G | T | T | A | A | T | T | T | T | T | T | T | G | G | T | C | G | T | C | T | G | C | T | C | T | G | A | G | A | C | A | T | T | A | G | C | A | A | C | T | G | G | A | G | A | T | G | G | T | A |
| --- | --- | --- | --- | --- | --- | --- | --- | --- | --- | --- | --- | --- | --- | --- | --- | --- | --- | --- | --- | --- | --- | --- | --- | --- | --- | --- | --- | --- | --- | --- | --- | --- | --- | --- | --- | --- | --- | --- | --- | --- | --- | --- | --- | --- | --- | --- | --- | --- | --- | --- | --- | --- | --- | --- | --- | --- | --- | --- | --- | --- |
| S21C6 | T | G | A | T | A | G | T | T | T | T | G | T | T | A | A | T | T | T | T | T | T | T | G | G | T | C | G | T | C | T | G | C | T | C | T | C | A | G | A | C | A | T | T | A | G | C | A | A | C | T | G | G | A | G | A | T | G | G | T | A |

***L I V L L I F L V V C*** S **Q** T L A T G D G

K Q E K K - - I A D - - - - W E K **K** **S** F

| G19B4 | A | A | C | A | G | G | A | A | A | A | G | A | A | A | A | - | - | - | - | - | - | T | A | G | C | T | G | A | - | - | - | - | - | - | - | - | - | - | - | - | T | T | G | G | G | A | G | A | A | A | A | A | A | T | C | A | T | T | T | G |
| --- | --- | --- | --- | --- | --- | --- | --- | --- | --- | --- | --- | --- | --- | --- | --- | --- | --- | --- | --- | --- | --- | --- | --- | --- | --- | --- | --- | --- | --- | --- | --- | --- | --- | --- | --- | --- | --- | --- | --- | --- | --- | --- | --- | --- | --- | --- | --- | --- | --- | --- | --- | --- | --- | --- | --- | --- | --- | --- | --- | --- |
| S21C6 | A | A | C | A | G | G | A | A | A | A | G | A | A | A | A | A | G | G | A | A | C | T | G | G | C | T | G | A | A | G | A | A | A | A | A | A | G | G | A | A | C | T | G | G | G | A | G | A | A | A | G | A | A | C | C | A | T | T | T | G |

K Q E K K K E L A E E K R N W E K **E** **P** F

D K F V **N** E I L **L** Q A N L H G D **W** **E** E T

| G19B4 | A | C | A | A | G | T | T | T | G | T | T | A | A | T | G | A | A | A | T | T | C | T | C | T | T | A | C | A | A | G | C | T | A | A | C | C | T | T | C | A | C | G | G | A | G | A | T | T | G | G | G | A | A | G | A | G | A | C | A | G |
| --- | --- | --- | --- | --- | --- | --- | --- | --- | --- | --- | --- | --- | --- | --- | --- | --- | --- | --- | --- | --- | --- | --- | --- | --- | --- | --- | --- | --- | --- | --- | --- | --- | --- | --- | --- | --- | --- | --- | --- | --- | --- | --- | --- | --- | --- | --- | --- | --- | --- | --- | --- | --- | --- | --- | --- | --- | --- | --- | --- | --- |
| S21C6 | A | C | A | A | G | T | T | T | G | T | T | C | A | T | G | A | A | A | T | T | C | T | C | T | T | T | C | A | A | G | C | T | A | A | C | C | T | T | C | A | C | G | G | A | G | A | T | C | G | G | A | A | A | G | A | G | G | G | A | G |

D K F V **H** E I L **F** Q A N L H G D **R** **K** E G

D H C Y P **K** P S **G** **S** D **E** R **S** S A N **S** **F** **S**

| G19B4 | A | T | C | A | C | T | G | T | T | A | T | C | C | C | A | A | A | C | C | A | A | G | T | G | G | A | A | G | T | G | A | C | G | A | A | A | G | G | A | G | T | T | C | T | G | C | A | A | A | T | T | C | G | T | T | C | T | C | A | A |
| --- | --- | --- | --- | --- | --- | --- | --- | --- | --- | --- | --- | --- | --- | --- | --- | --- | --- | --- | --- | --- | --- | --- | --- | --- | --- | --- | --- | --- | --- | --- | --- | --- | --- | --- | --- | --- | --- | --- | --- | --- | --- | --- | --- | --- | --- | --- | --- | --- | --- | --- | --- | --- | --- | --- | --- | --- | --- | --- | --- | --- |
| S21C6 | A | T | C | A | C | C | C | T | T | A | T | C | C | C | G | A | A | C | C | A | A | G | T | G | A | A | C | G | T | G | A | C | G | T | A | A | G | G | A | A | T | T | C | T | G | C | A | A | A | T | C | C | G | T | G | C | C | C | A | A |

D H P Y P **E** P S **E** **R** D **V** R **N** S A N **P** **C** **P**

T **K** S K S M Q I K G D **H** **F** Q **T** D L Q R **P**

| G19B4 | C | T | A | A | A | A | G | C | A | A | A | T | C | T | A | T | G | C | A | A | A | T | T | A | A | A | G | G | A | G | A | T | C | A | C | T | T | C | C | A | A | A | C | T | G | A | C | C | T | T | C | A | G | A | G | A | C | C | T | T |
| --- | --- | --- | --- | --- | --- | --- | --- | --- | --- | --- | --- | --- | --- | --- | --- | --- | --- | --- | --- | --- | --- | --- | --- | --- | --- | --- | --- | --- | --- | --- | --- | --- | --- | --- | --- | --- | --- | --- | --- | --- | --- | --- | --- | --- | --- | --- | --- | --- | --- | --- | --- | --- | --- | --- | --- | --- | --- | --- | --- | --- |
| S21C6 | C | T | A | G | A | A | G | C | A | A | A | T | C | T | A | T | G | C | A | A | A | T | T | A | A | A | G | G | A | G | A | T | C | C | C | C | T | C | C | A | A | G | C | T | G | A | C | C | T | T | C | A | G | A | G | A | C | T | T | T |

T **R** S K S M Q I K G D **P** **L** Q **A** D L Q R **L**

S R E E L **H** **Q** P D P K P S K **C** **D** **E** G N S

| G19B4 | C | C | A | G | A | G | A | A | G | A | G | T | T | A | C | A | T | C | A | A | C | C | T | G | A | T | C | C | C | A | A | A | C | C | A | A | G | T | A | A | A | T | G | T | G | A | C | G | A | A | G | G | G | A | A | T | T | C | T | G |
| --- | --- | --- | --- | --- | --- | --- | --- | --- | --- | --- | --- | --- | --- | --- | --- | --- | --- | --- | --- | --- | --- | --- | --- | --- | --- | --- | --- | --- | --- | --- | --- | --- | --- | --- | --- | --- | --- | --- | --- | --- | --- | --- | --- | --- | --- | --- | --- | --- | --- | --- | --- | --- | --- | --- | --- | --- | --- | --- | --- | --- |
| S21C6 | C | C | A | G | A | G | A | A | G | A | G | T | T | A | G | A | T | A | A | A | C | C | T | G | A | T | C | C | C | A | A | A | C | C | A | A | G | T | A | A | A | A | G | T | C | A | C | C | A | A | G | G | G | A | A | T | T | C | T | G |

S R E E L **D** **K** P D P K P S K **S** **H** **Q** G N S

A N Q Q Q **M** K L T R **G** K S N H K R A A **H**

| G19B4 | C | A | A | A | T | C | A | G | C | A | G | C | A | G | A | T | G | A | A | A | C | T | A | A | C | T | A | G | A | G | G | A | A | A | A | T | C | T | A | A | C | C | A | C | A | A | A | A | G | A | G | C | T | G | C | A | C | A | C | T |
| --- | --- | --- | --- | --- | --- | --- | --- | --- | --- | --- | --- | --- | --- | --- | --- | --- | --- | --- | --- | --- | --- | --- | --- | --- | --- | --- | --- | --- | --- | --- | --- | --- | --- | --- | --- | --- | --- | --- | --- | --- | --- | --- | --- | --- | --- | --- | --- | --- | --- | --- | --- | --- | --- | --- | --- | --- | --- | --- | --- | --- |
| S21C6 | C | A | A | A | T | C | A | G | C | A | G | C | A | G | A | A | G | A | A | A | C | T | A | A | C | T | A | G | A | G | A | A | A | A | A | T | C | T | A | A | C | C | A | C | A | A | A | A | G | A | G | C | T | G | C | A | C | G | C | T |

A N Q Q Q **K** K L T R **E** K S N H K R A A **R**

| G19B4 | A | A | T | T | G | T | C | A | A | A | T | T | G | A | T | G | A | A | A | T | G | A | A | A | T | T | C | A | A | A | A | C | G | A | A | T | A | A | T | G | A | A | A | C | G | T | T | T | C | A | A | T | G | T | T | C | C | A | A | C |
| --- | --- | --- | --- | --- | --- | --- | --- | --- | --- | --- | --- | --- | --- | --- | --- | --- | --- | --- | --- | --- | --- | --- | --- | --- | --- | --- | --- | --- | --- | --- | --- | --- | --- | --- | --- | --- | --- | --- | --- | --- | --- | --- | --- | --- | --- | --- | --- | --- | --- | --- | --- | --- | --- | --- | --- | --- | --- | --- | --- | --- |
| S21C6 | G | A | T | T | G | T | C | A | A | A | T | T | A | A | T | G | A | A | A | T | G | A | A | A | T | T | T | A | A | A | A | C | G | A | A | T | A | A | T | G | A | A | A | C | G | C | T | T | C | A | T | T | G | T | T | C | C | A | A | C |

| G19B4 | G | T | T | A | C | A | A | T | A | T | T | C | T | G | T | T | T | A | T | T | T | A | G | A | A | C | A | G | C | T | G | T | T | C | T | C | A | G | C | T | A | T | G | A | A | A | T | T | C | A | C | A | A | A | T | G | A | A | A | A |
| --- | --- | --- | --- | --- | --- | --- | --- | --- | --- | --- | --- | --- | --- | --- | --- | --- | --- | --- | --- | --- | --- | --- | --- | --- | --- | --- | --- | --- | --- | --- | --- | --- | --- | --- | --- | --- | --- | --- | --- | --- | --- | --- | --- | --- | --- | --- | --- | --- | --- | --- | --- | --- | --- | --- | --- | --- | --- | --- | --- | --- |
| S21C6 | G | T | T | A | C | A | A | T | A | T | T | C | T | G | T | T | T | A | T | T | T | G | A | G | A | A | T | T | T | C | G | T | T | T | - | - | - | A | T | T | T | G | A | A | A | A | T | T | C | A | C | A | A | A | T | G | A | A | A | A |

| G19B4 | A | T | A | T | G | G | G | C | T | C | C | A | G | G | C | T | T | A | A | A | A | T | T | T | C | A | T | T | A | G | A | C | G | G | A | T | T | T | A | A | A | A | T | A | C |  |  |  |  |  |  |  |  |  |  |  |  |  |  |  |
| --- | --- | --- | --- | --- | --- | --- | --- | --- | --- | --- | --- | --- | --- | --- | --- | --- | --- | --- | --- | --- | --- | --- | --- | --- | --- | --- | --- | --- | --- | --- | --- | --- | --- | --- | --- | --- | --- | --- | --- | --- | --- | --- | --- | --- | --- | --- | --- | --- | --- | --- | --- | --- | --- | --- | --- | --- | --- | --- | --- | --- |
| S21C6 | A | T | A | T | G | G | T | C | T | C | A | T | G | G | C | T | T | A | A | A | A | T | T | T | C | A | T | T | A | A | A | C | G | G | A | A | T | A | A | A | A | A | T | A | C |  |  |  |  |  |  |  |  |  |  |  |  |  |  |  |

**C**

M K Y S L

| G10H7 | A | T | C | A | G | T | T | T | T | G | A | A | T | C | A | A | T | T | T | T | G | T | T | C | A | A | G | A | A | A | A | A | A | A | G | A | C | T | A | A | T | T | T | G | A | A | **A** | **T** | **G** | A | A | G | T | A | C | T | C | G | T | T |
| --- | --- | --- | --- | --- | --- | --- | --- | --- | --- | --- | --- | --- | --- | --- | --- | --- | --- | --- | --- | --- | --- | --- | --- | --- | --- | --- | --- | --- | --- | --- | --- | --- | --- | --- | --- | --- | --- | --- | --- | --- | --- | --- | --- | --- | --- | --- | --- | --- | --- | --- | --- | --- | --- | --- | --- | --- | --- | --- | --- | --- |
| G14E6 | - | T | C | A | G | T | T | T | T | G | A | A | T | C | A | A | T | T | T | T | G | T | T | C | A | A | G | A | A | A | A | A | A | A | G | A | C | T | A | A | T | T | T | G | A | A | **A** | **T** | **G** | A | A | G | T | A | C | T | C | G | T | T |

***M K Y S L***

***A F L F V A A T A I L V A*** S V E A S G S

| G10H7 | A | G | C | T | T | T | C | C | T | T | T | T | C | G | T | A | G | C | T | G | C | C | A | C | A | G | C | T | A | T | T | T | T | G | G | T | G | G | C | C | A | G | C | G | T | G | G | A | A | G | C | A | A | G | T | G | G | A | T | C |
| --- | --- | --- | --- | --- | --- | --- | --- | --- | --- | --- | --- | --- | --- | --- | --- | --- | --- | --- | --- | --- | --- | --- | --- | --- | --- | --- | --- | --- | --- | --- | --- | --- | --- | --- | --- | --- | --- | --- | --- | --- | --- | --- | --- | --- | --- | --- | --- | --- | --- | --- | --- | --- | --- | --- | --- | --- | --- | --- | --- | --- |
| G14E6 | A | G | C | T | T | T | C | C | T | T | T | T | C | G | T | A | G | C | T | G | C | C | A | C | A | G | C | T | A | T | T | T | T | G | A | T | G | G | C | C | A | G | C | G | T | G | G | A | A | G | C | A | A | G | T | G | G | A | T | C |

***A F L F V A A T A I L M A*** S V E A S G S

P **G** S A G S R **I** S G P R G S G S G G S P

| G10H7 | A | C | C | C | G | G | T | T | C | C | G | C | C | G | G | T | T | C | C | A | G | A | A | T | A | T | C | C | G | G | T | C | C | C | A | G | G | G | G | A | T | C | G | G | G | T | T | C | C | G | G | G | G | G | A | T | C | A | C | C |
| --- | --- | --- | --- | --- | --- | --- | --- | --- | --- | --- | --- | --- | --- | --- | --- | --- | --- | --- | --- | --- | --- | --- | --- | --- | --- | --- | --- | --- | --- | --- | --- | --- | --- | --- | --- | --- | --- | --- | --- | --- | --- | --- | --- | --- | --- | --- | --- | --- | --- | --- | --- | --- | --- | --- | --- | --- | --- | --- | --- | --- |
| G14E6 | A | T | C | T | C | G | T | T | C | C | A | C | - | - | - | - | - | - | - | - | - | G | G | T | A | T | C | - | - | - | - | - | - | - | - | - | - | - | - | A | T | C | G | G | G | T | T | C | C | G | G | G | G | G | A | T | C | A | C | C |

S **R** S T - - - **V** S - - - - S G S G G S P

G S A G S S G S R V S G S G G S P **T** N **I**

| G10h7 | C | G | G | T | T | C | C | G | C | G | G | G | A | T | C | A | T | C | C | G | G | T | T | C | C | A | G | A | G | T | A | T | C | G | G | G | T | T | C | C | G | G | G | G | G | A | T | C | A | C | C | C | A | C | T | A | A | T | A | T |
| --- | --- | --- | --- | --- | --- | --- | --- | --- | --- | --- | --- | --- | --- | --- | --- | --- | --- | --- | --- | --- | --- | --- | --- | --- | --- | --- | --- | --- | --- | --- | --- | --- | --- | --- | --- | --- | --- | --- | --- | --- | --- | --- | --- | --- | --- | --- | --- | --- | --- | --- | --- | --- | --- | --- | --- | --- | --- | --- | --- | --- |
| G14E6 | C | G | G | T | T | C | C | G | C | G | G | G | A | T | C | A | T | C | C | G | G | T | T | C | C | A | G | A | G | T | A | T | C | G | G | G | T | T | C | C | G | G | - | - | - | - | - | - | - | - | - | C | T | C | T | C | C | T | A | C |

G S A G S S G S R V S G S G - - - **S** P **T**

Q A Q D D M V R **K** **V** E S A F **A** A S **S** N P

| G10H7 | T | C | A | A | G | C | C | C | A | G | G | A | T | G | A | C | A | T | G | G | T | G | C | G | T | A | A | A | G | T | G | G | A | A | A | G | C | G | C | C | T | T | T | G | C | C | G | C | A | T | C | G | T | C | A | A | A | T | C | C |
| --- | --- | --- | --- | --- | --- | --- | --- | --- | --- | --- | --- | --- | --- | --- | --- | --- | --- | --- | --- | --- | --- | --- | --- | --- | --- | --- | --- | --- | --- | --- | --- | --- | --- | --- | --- | --- | --- | --- | --- | --- | --- | --- | --- | --- | --- | --- | --- | --- | --- | --- | --- | --- | --- | --- | --- | --- | --- | --- | --- | --- |
| G14E6 | T | C | A | A | G | C | C | C | A | G | G | A | T | G | A | C | T | T | A | G | T | G | C | G | T | G | A | A | C | T | G | A | A | C | A | G | C | G | C | C | T | T | T | G | A | C | G | C | A | T | C | G | C | C | A | A | A | T | C | C |

Q A Q D D L V R **E** **L** N S A F **D** A S **P** N P

S V A P **R** R T L V S F S N L P R M N K S

| G10H7 | A | A | G | T | G | T | G | G | C | T | C | C | T | A | G | A | A | G | G | A | C | A | C | T | T | G | T | C | T | C | T | T | T | T | T | C | C | A | A | C | C | T | A | C | C | A | C | G | G | A | T | G | A | A | T | A | A | A | T | C |
| --- | --- | --- | --- | --- | --- | --- | --- | --- | --- | --- | --- | --- | --- | --- | --- | --- | --- | --- | --- | --- | --- | --- | --- | --- | --- | --- | --- | --- | --- | --- | --- | --- | --- | --- | --- | --- | --- | --- | --- | --- | --- | --- | --- | --- | --- | --- | --- | --- | --- | --- | --- | --- | --- | --- | --- | --- | --- | --- | --- | --- |
| G14E6 | A | A | G | T | G | T | G | G | C | T | C | C | T | A | G | T | A | G | G | A | C | A | C | T | T | G | T | C | T | C | T | T | T | T | T | C | C | A | A | C | C | T | A | C | C | A | C | G | G | A | T | G | A | A | T | A | A | A | T | C |

S V A P **S** R T L V S F S N L P R M N K S

L Q A A G I **V** I Q G S L E D S I N G E T

| G10H7 | A | T | T | G | C | A | A | G | C | A | G | C | A | G | G | C | A | T | T | G | T | C | A | T | A | C | A | G | G | G | A | A | G | C | T | T | A | G | A | G | G | A | T | T | C | T | A | T | C | A | A | T | G | G | A | G | A | A | A | C |
| --- | --- | --- | --- | --- | --- | --- | --- | --- | --- | --- | --- | --- | --- | --- | --- | --- | --- | --- | --- | --- | --- | --- | --- | --- | --- | --- | --- | --- | --- | --- | --- | --- | --- | --- | --- | --- | --- | --- | --- | --- | --- | --- | --- | --- | --- | --- | --- | --- | --- | --- | --- | --- | --- | --- | --- | --- | --- | --- | --- | --- |
| G14E6 | A | T | T | G | C | A | A | G | C | A | G | C | A | G | G | C | A | T | T | G | C | C | A | T | A | C | A | G | G | G | A | A | G | C | T | T | A | G | A | G | G | A | T | T | C | T | A | T | C | A | A | T | G | G | A | G | A | A | A | C |

L Q A A G I **A** I Q G S L E D S I N G E T

D E R K L M A A L R H F V D H Y S Y S T

| G10H7 | A | G | A | T | G | A | G | C | G | T | A | A | A | C | T | T | A | T | G | G | C | G | G | C | T | C | T | A | C | G | A | C | A | T | T | T | T | G | T | A | G | A | C | C | A | T | T | A | T | T | C | T | T | A | T | A | G | T | A | C |
| --- | --- | --- | --- | --- | --- | --- | --- | --- | --- | --- | --- | --- | --- | --- | --- | --- | --- | --- | --- | --- | --- | --- | --- | --- | --- | --- | --- | --- | --- | --- | --- | --- | --- | --- | --- | --- | --- | --- | --- | --- | --- | --- | --- | --- | --- | --- | --- | --- | --- | --- | --- | --- | --- | --- | --- | --- | --- | --- | --- | --- |
| G14E6 | A | G | A | T | G | A | G | C | G | T | A | A | A | C | T | T | A | T | G | G | C | G | G | C | T | C | T | A | C | G | A | C | A | T | T | T | T | G | T | A | A | A | A | C | A | T | T | A | T | T | C | T | T | A | T | A | G | T | A | C |

D E R K L M A A L R H F V K H Y S Y S T

S P P **R** D G A S T S K R R

| G10H7 | A | T | C | T | C | C | A | C | C | G | A | G | G | G | A | T | G | G | T | G | C | A | T | C | T | A | C | T | A | G | C | A | A | G | A | G | A | A | G | A | **T** | **A** | **A** | A | A | A | A | A | A | C | T | G | A | A | A | T | A | T | T | C |
| --- | --- | --- | --- | --- | --- | --- | --- | --- | --- | --- | --- | --- | --- | --- | --- | --- | --- | --- | --- | --- | --- | --- | --- | --- | --- | --- | --- | --- | --- | --- | --- | --- | --- | --- | --- | --- | --- | --- | --- | --- | --- | --- | --- | --- | --- | --- | --- | --- | --- | --- | --- | --- | --- | --- | --- | --- | --- | --- | --- | --- |
| G14E6 | A | T | C | T | C | C | A | C | C | G | A | A | G | G | A | T | G | G | T | G | C | A | T | C | T | A | C | T | A | G | C | A | A | G | A | G | A | A | G | A | **T** | **A** | **A** | A | A | A | A | A | - | C | T | G | A | A | A | T | A | T | T | C |

S P P **K** D G A S T S K R R

| G10H7 | A | A | T | C | A | A | A | C | A | T | T | C | A | C | A | A | A | T | G | T | C | A | C | A | C | T | A | A | T | A | T | T | C | A | A | T | G | T | A | A | T | A | A | T | T | T | T | T | T | T | T | A | A | T | A | C | A | A | A | A |
| --- | --- | --- | --- | --- | --- | --- | --- | --- | --- | --- | --- | --- | --- | --- | --- | --- | --- | --- | --- | --- | --- | --- | --- | --- | --- | --- | --- | --- | --- | --- | --- | --- | --- | --- | --- | --- | --- | --- | --- | --- | --- | --- | --- | --- | --- | --- | --- | --- | --- | --- | --- | --- | --- | --- | --- | --- | --- | --- | --- | --- |
| G14E6 | A | A | T | C | A | A | A | C | A | T | T | C | A | C | A | A | A | T | G | T | C | A | C | A | C | T | A | A | T | A | T | T | C | A | A | T | G | T | A | A | T | A | A | T | T | T | T | T | T | T | T | A | A | T | A | C | A | A | A | A |

| G10H7 | T | T | G | A | A | T | T | G | G | T | A | A | C | T | T | C | T | G | A | T | A | T | A | A | A | G | A | A | A | T | A | A | A | A | T | A | T | T | T | T | T | G | A | A | T | G | T | T | C | A | C | G | C |  |  |  |  |  |  |  |
| --- | --- | --- | --- | --- | --- | --- | --- | --- | --- | --- | --- | --- | --- | --- | --- | --- | --- | --- | --- | --- | --- | --- | --- | --- | --- | --- | --- | --- | --- | --- | --- | --- | --- | --- | --- | --- | --- | --- | --- | --- | --- | --- | --- | --- | --- | --- | --- | --- | --- | --- | --- | --- | --- | --- | --- | --- | --- | --- | --- | --- |
| G14E6 | T | T | G | A | A | T | T | G | G | T | A | A | C | T | T | C | T | G | A | T | A | T | A | A | A | G | A | A | A | T | A | A | A | A | T | A | T | T | T | T | T | A | G | A | T | G | T | T | C | A | C | G | C |  |  |  |  |  |  |  |

**D**

M K Y

| L4C4 | A | A | A | C | G | A | A | A | T | A | T | T | T | T | T | G | T | T | A | A | A | G | A | A | A | A | C | G | T | T | A | C | C | A | A | T | T | T | T | C | G | A | G | C | G | A | A | A | A | A | **A** | **T** | **G** | A | A | A | T | A | T | T |
| --- | --- | --- | --- | --- | --- | --- | --- | --- | --- | --- | --- | --- | --- | --- | --- | --- | --- | --- | --- | --- | --- | --- | --- | --- | --- | --- | --- | --- | --- | --- | --- | --- | --- | --- | --- | --- | --- | --- | --- | --- | --- | --- | --- | --- | --- | --- | --- | --- | --- | --- | --- | --- | --- | --- | --- | --- | --- | --- | --- | --- |
| G28G12 | - | A | A | C | G | A | A | A | T | A | T | T | T | T | T | G | T | T | T | A | A | G | A | A | A | A | C | G | T | T | A | C | C | A | A | T | T | T | T | C | G | A | G | C | G | A | A | A | A | **A** | - | **T** | **G** | A | A | A | T | A | T | T |

***M K Y***

***S L A C I F L I T F A V S*** I N V C S **S** **N**

| L4C4 | C | A | C | T | T | G | C | T | T | G | T | A | T | T | T | T | C | C | T | G | A | T | T | A | C | T | T | T | T | G | C | C | G | T | T | T | C | A | A | T | A | A | A | C | G | T | T | T | G | T | T | C | C | A | G | C | A | A | T | G |
| --- | --- | --- | --- | --- | --- | --- | --- | --- | --- | --- | --- | --- | --- | --- | --- | --- | --- | --- | --- | --- | --- | --- | --- | --- | --- | --- | --- | --- | --- | --- | --- | --- | --- | --- | --- | --- | --- | --- | --- | --- | --- | --- | --- | --- | --- | --- | --- | --- | --- | --- | --- | --- | --- | --- | --- | --- | --- | --- | --- | --- |
| G28G12 | C | A | C | T | T | G | C | T | T | G | T | A | T | T | T | T | C | C | T | G | A | T | T | A | C | T | T | T | T | G | C | C | G | T | T | T | C | T | A | T | A | A | A | C | G | T | T | T | G | T | T | C | C | A | G | T | G | A | T | G |

***S L A C I F L I T F A V S*** I N V C S **S** **D**

**G** E G - G L - D - L D S **L** R V Q **A** **I** T **I**

| L4C4 | G | A | G | A | A | G | G | A | G | - | - | G | G | T | T | - | - | - | - | G | G | A | T | - | - | - | T | T | G | G | A | C | A | G | T | T | T | G | A | G | A | G | T | T | C | A | A | G | C | T | A | T | T | A | C | G | A | T | T | G |
| --- | --- | --- | --- | --- | --- | --- | --- | --- | --- | --- | --- | --- | --- | --- | --- | --- | --- | --- | --- | --- | --- | --- | --- | --- | --- | --- | --- | --- | --- | --- | --- | --- | --- | --- | --- | --- | --- | --- | --- | --- | --- | --- | --- | --- | --- | --- | --- | --- | --- | --- | --- | --- | --- | --- | --- | --- | --- | --- | --- | --- |
| G28G12 | A | A | A | A | T | A | A | A | G | T | C | G | A | T | T | T | A | G | C | G | G | A | T | A | A | A | C | T | A | A | A | T | G | G | C | T | T | A | A | G | A | A | A | T | C | A | A | G | T | T | A | C | T | G | G | G | A | C | T | G |

**E** N K V D L A D K L N G **L** R N Q **V** **T** G **T**

V L K E E A T K S A Y F R N I L H H **F** **G**

| L4C4 | T | G | C | T | G | A | A | A | G | A | A | G | A | A | G | C | A | A | C | A | A | A | G | A | G | C | G | C | T | T | A | T | T | T | C | C | G | A | A | A | T | A | T | T | T | T | G | C | A | C | C | A | C | T | T | T | G | G | A | A |
| --- | --- | --- | --- | --- | --- | --- | --- | --- | --- | --- | --- | --- | --- | --- | --- | --- | --- | --- | --- | --- | --- | --- | --- | --- | --- | --- | --- | --- | --- | --- | --- | --- | --- | --- | --- | --- | --- | --- | --- | --- | --- | --- | --- | --- | --- | --- | --- | --- | --- | --- | --- | --- | --- | --- | --- | --- | --- | --- | --- | --- |
| G28G12 | T | G | C | T | G | A | A | A | G | A | A | G | A | A | G | C | A | A | C | A | A | A | G | A | G | C | G | C | T | T | A | T | T | T | C | C | G | A | A | A | T | A | T | T | T | T | G | C | A | C | A | G | C | C | T | T | G | G | T | A |

V L K E E A T K S A Y F R N I L H S **L** **G**

I L R E P G **N** I R K E **E** N E I **V** **N** **E** I N

| L4C4 | T | T | C | T | C | A | G | G | G | A | G | C | C | T | G | G | A | A | A | C | A | T | T | A | G | A | A | A | A | G | A | A | G | A | A | A | A | C | G | A | G | A | T | C | G | T | A | A | A | T | G | A | A | A | T | C | A | A | T | T |
| --- | --- | --- | --- | --- | --- | --- | --- | --- | --- | --- | --- | --- | --- | --- | --- | --- | --- | --- | --- | --- | --- | --- | --- | --- | --- | --- | --- | --- | --- | --- | --- | --- | --- | --- | --- | --- | --- | --- | --- | --- | --- | --- | --- | --- | --- | --- | --- | --- | --- | --- | --- | --- | --- | --- | --- | --- | --- | --- | --- | --- |
| G28G12 | T | T | C | T | C | A | G | G | G | A | G | C | C | T | G | G | A | A | A | T | A | T | T | A | G | A | A | A | A | G | A | A | G | C | A | A | A | C | G | A | G | A | T | C | G | A | A | G | A | T | C | A | A | A | T | C | - | - | - | T |

I L R E P G **N** I R K E **A** N E I **E** **D** **Q** I -

S **Y** **C** S N T G L R S **G** **I** V **E**  T **L** **K** K **A** **L**

| L4C4 | C | A | T | A | T | T | G | T | A | G | C | A | A | T | A | C | A | G | G | A | C | T | C | A | G | G | A | G | T | G | G | A | A | T | T | G | T | T | G | A | G | A | C | G | C | T | T | A | A | G | A | A | G | G | C | T | T | T | G | A |
| --- | --- | --- | --- | --- | --- | --- | --- | --- | --- | --- | --- | --- | --- | --- | --- | --- | --- | --- | --- | --- | --- | --- | --- | --- | --- | --- | --- | --- | --- | --- | --- | --- | --- | --- | --- | --- | --- | --- | --- | --- | --- | --- | --- | --- | --- | --- | --- | --- | --- | --- | --- | --- | --- | --- | --- | --- | --- | --- | --- | --- |
| G28G12 | C | A | A | A | T | T | A | T | T | G | T | G | - | - | A | C | A | A | A | A | C | T | - | A | G | G | A | G | T | A | G | A | A | C | T | G | T | T | C | A | G | A | C | A | C | T | A | A | C | G | A | C | C | G | G | T | T | T | A | G |

S **N** **Y** C - D K T R S **R** **T** V **Q** T **L** **T** T **G** **L**

**N** A **Y** **Y** **H** P **N** H **R** **A** **A** - **T** R S **G**

| L4C4 | A | T | G | C | A | T | A | T | T | A | C | C | A | C | C | C | A | A | A | T | C | A | C | A | G | G | G | C | A | G | C | C | - | - | - | A | C | C | A | G | A | A | G | C | G | G | T | **T** | **A** | **A** | T | C | C | A | G | A | A | A | A | T |
| --- | --- | --- | --- | --- | --- | --- | --- | --- | --- | --- | --- | --- | --- | --- | --- | --- | --- | --- | --- | --- | --- | --- | --- | --- | --- | --- | --- | --- | --- | --- | --- | --- | --- | --- | --- | --- | --- | --- | --- | --- | --- | --- | --- | --- | --- | --- | --- | --- | --- | --- | --- | --- | --- | --- | --- | --- | --- | --- | --- | --- |
| G28G12 | A | T | G | C | A | T | G | T | A | A | C | A | A | C | C | C | A | A | A | G | C | G | A | A | A | G | C | C | A | G | C | T | T | C | A | G | C | C | A | G | A | A | G | A | G | G | A | **T** | **A** | **A** | T | C | C | A | A | A | A | A | A | T |

**D** A **C** **N** **N** P **K** R **K** **P** **A** S **A** R **R** **G**

| L4C4 | T | G | A | G | G | A | A | A | C | G | A | A | A | T | C | T | T | T | G | T | T | T | T | T | T | G | G | A | C | C | T | T | T | T | A | A | A | A | T | G | A | T | A | C | T | T | T | G | A | C | A | A | T | C | T | G | C | A | T | C |
| --- | --- | --- | --- | --- | --- | --- | --- | --- | --- | --- | --- | --- | --- | --- | --- | --- | --- | --- | --- | --- | --- | --- | --- | --- | --- | --- | --- | --- | --- | --- | --- | --- | --- | --- | --- | --- | --- | --- | --- | --- | --- | --- | --- | --- | --- | --- | --- | --- | --- | --- | --- | --- | --- | --- | --- | --- | --- | --- | --- | --- |
| G28G12 | T | G | A | G | A | A | A | A | C | G | A | A | A | T | C | T | T | T | G | T | T | T | T | T | T | G | G | A | C | C | T | T | T | T | A | A | A | A | T | G | A | T | A | C | T | T | T | G | A | C | A | A | T | C | T | G | C | A | T | T |

| L4C4 | T | C | T | T | A | A | C | T | A | A | A | T | A | A | T | A | T | T | C | C | A | C | T | A | A | A | T | A | T | A | T | C | A | A | T | T | A | T | T | T | A | T | T | C | A | A | A | T | T | A | A | A | T | T | C | A | A | A | G | C |
| --- | --- | --- | --- | --- | --- | --- | --- | --- | --- | --- | --- | --- | --- | --- | --- | --- | --- | --- | --- | --- | --- | --- | --- | --- | --- | --- | --- | --- | --- | --- | --- | --- | --- | --- | --- | --- | --- | --- | --- | --- | --- | --- | --- | --- | --- | --- | --- | --- | --- | --- | --- | --- | --- | --- | --- | --- | --- | --- | --- | --- |
| G28G12 | T | C | T | T | A | A | C | T | A | A | A | T | G | A | A | A | T | T | C | C | A | C | T | A | A | A | T | A | T | A | T | C | A | A | T | T | A | T | T | T | A | T | T | C | A | A | A | T | T | A | A | A | T | T | C | A | A | A | G | C |

| L4C4 | A | A | T | A | A | A | T | A | T | T | T | T | T | A | T | T | T | T | T | T | T | T | T | - | - | G | T | C | A | G | - |  |  |  |  |  |  |  |  |  |  |  |  |  |  |  |  |  |  |  |  |  |  |  |  |  |  |  |  |  |
| --- | --- | --- | --- | --- | --- | --- | --- | --- | --- | --- | --- | --- | --- | --- | --- | --- | --- | --- | --- | --- | --- | --- | --- | --- | --- | --- | --- | --- | --- | --- | --- | --- | --- | --- | --- | --- | --- | --- | --- | --- | --- | --- | --- | --- | --- | --- | --- | --- | --- | --- | --- | --- | --- | --- | --- | --- | --- | --- | --- | --- |
| G28G12 | A | A | T | A | A | A | T | A | C | T | T | T | T | A | T | T | T | T | T | T | T | T | T | T | T | G | T | C | A | G | C |  |  |  |  |  |  |  |  |  |  |  |  |  |  |  |  |  |  |  |  |  |  |  |  |  |  |  |  |  |

**E**

***M L N S K S***

G22C4 1 ATTCAAAACTTGAATACCAAAAAGTGAAGTGAATTGTTTTAAA**ATG**TTAAACTCTAAAAG
G13E6 1 --------------TACCAAAAAGTGAAGTGAATTGTTTTAAA**ATG**TTAAACTCTAAAAG
 ***M L N S K S***

***W I I C S F L F V A V L A*** V G T D N S A

G22C4 61 TTGGATCATTTGTTCGTTTTTATTTGTTGCGGTATTGGCAGTGGGCACTGATAACTCAGC
G13E6 47 TTGGATCATTTGTTCGTTTTTATTTGCTGCGGTATTGGCAGTGGGCACTGATAACTCAGA
 ***W I I C S F L F A A V L A*** V G T D N S E

E N **E** N I S Y L **R**

G22C4 121 AGAAAATGAAAA---------------------------------TATATCCTATTTAAG
G13E6 107 AGAAAATGATGAAATGGCTAAATGGGTCAAGTCTCTTGATGGAAGTATATCCTATTTAAA
 E N **D** E M A K W V K S L D G S I S Y L **K**

S K N F K N **K** E F T D L N L Y **E** W S D W

G22C4 148 AAGCAAAAATTTTAAGAACAAAGAATTTACTGATTTAAATTTATATGAATGGAGTGACTG
G13E6 167 AAGCAAAAATTTTAAGAACACAGAATTTACTGATTTAAATTTATATGTATGGAGTGACTG
 S K N F K N **T** E F T D L N L Y **V** W S D W

E K **I** P Y R L K **A** A A S Y T Y K T E M L

G22C4 208 GGAAAAAATTCCTTACAGATTAAAAGCTGCTGCTTCGTATACATATAAGACAGAGATGCT
G13E6 227 GGAAAAAATGCCTTACAGATTAAAAGCATTTGCTTCGTATACATATAAGACAGAAATGCT
 E K **M** P Y R L K **A** F A S Y T Y K T E M L

K K **N** Y K **T** N R N A D Q F **S** A T C A Y K

G22C4 268 CAAGAAAAATTATAAAACAAATCGAAATGCTGATCAATTTAGCGCTACTTGTGCTTATAA
G13E6 287 CAAGAAAAAATATAAACCAAATCGAAATGCTGATCAATTTACCGCTACTTGTGCTTATAA
 K K **K** Y K **P** N R N A D Q F **T** A T C A Y K

A F K **E** F L **R** K R **N** **E** **F** **M** Y F H **L** T F H

G22C4 328 GGCATTCAAAGAGTTTCTCAGGAAACGTAATGAGTTTATGTATTTTCATCTGACTTTCCA
G13E6 347 GGCATTCAAAGATTTTCTCAAGAAACAAGATGAATTGATCTATTTTCATTTGACTTTCCA
 A F K **D** F L **K** K Q **D** **E** **L** **I** Y F H **L** T F H

**T** **D** L K M K Y D E I F N D K **V** **K** **F** L K F

G22C4 388 TACAGACCTCAAGATGAAATATGATGAAATATTTAATGATAAAGTAAAATTTTTGAAATT
G13E6 407 TAAAGCCCTCAAGGCGAAATATGATGAAATATTTAATGATAAAGCAAACTATTTGAAATT
 **K** **A** L K A K Y D E I F N D K **A** **N** **Y** L K F

**I** I D F **E** A **V** L E **E** **K** **D** A S F M D I L Y

G22C4 448 TATTATTGATTTCGAAGCCGTATTAGAAGAGAAAGATGCTTCTTTCATGGATATTTTATA
G13E6 467 TCTTATTGATTTCGAGGCCGAATTAGAAGACGAAAATG----------------------
 **L** I D F **E** A **E** L E **D** **E** **N** - - - - - - - -

I P F V A D V F R D Y F K G K A S G A G

G22C4 508 CATACCATTCGTTGCTGATGTTTTTCGCG**A**TTATTTTAAAGGCAAAGCCTCAGGGGCAGG
G13E6 505 ----------GTTGC**TAA**TGCTTTTCGC**A**GTTATCTTAAAGGCAAAGCCTCAGGGGCAGG
 - - G C

A S S S T S

G22C4 568 TGCTTCATCGTCAACCTCA**TGA**TTTTCAAGATTCTGGGTTTTCATCGATTTTCTTT-GGT
G13E6 555 TGCTTCATCGTCAACCTCATGATTTTCCAGATTCTGTGTTTTCATCGATTTTTTTTTGGT

G22C4 627 ATCTTCGGTGGCCTTAGACATCATTTGATCGCTAATCAGAATGAAAAA-TAAATAAAATA
G13E6 615 ATCTTCGGTGGCCTTAGACAGCATTTGTTCGCTAATCAGAATGAAAAACAAAATAAAATA

G22C4 686 AATTTCAATTCC-
G13E6 675 AAATTCAATTCCC

**F**

***M L N S K S***

| G2C8 | - | T | T | C | A | A | A | A | C | T | T | G | A | A | T | A | C | C | A | A | A | A | A | G | T | G | A | A | G | T | G | A | A | T | T | G | T | T | T | T | A | A | A | **A** | **T** | **G** | T | T | A | A | A | C | T | C | T | A | A | A | A | G |
| --- | --- | --- | --- | --- | --- | --- | --- | --- | --- | --- | --- | --- | --- | --- | --- | --- | --- | --- | --- | --- | --- | --- | --- | --- | --- | --- | --- | --- | --- | --- | --- | --- | --- | --- | --- | --- | --- | --- | --- | --- | --- | --- | --- | --- | --- | --- | --- | --- | --- | --- | --- | --- | --- | --- | --- | --- | --- | --- | --- | --- |
| L4H12 | T | T | C | A | A | A | A | A | C | T | T | G | A | A | T | A | C | C | A | A | A | A | A | G | T | G | A | A | G | T | G | A | A | T | T | G | T | T | T | T | A | A | A | **A** | **T** | **G** | T | T | A | A | A | C | T | C | T | A | A | A | A | G |

***M L N S K S***

***W I I C S F L F V A V L A*** **M** G **T** D N S A

| G2C8 | T | T | G | G | A | T | C | A | T | T | T | G | T | T | C | T | T | T | T | T | T | A | T | T | T | G | T | T | G | C | G | G | T | A | T | T | G | G | C | A | A | T | G | G | G | C | A | C | T | G | A | T | A | A | C | T | C | A | G | C |
| --- | --- | --- | --- | --- | --- | --- | --- | --- | --- | --- | --- | --- | --- | --- | --- | --- | --- | --- | --- | --- | --- | --- | --- | --- | --- | --- | --- | --- | --- | --- | --- | --- | --- | --- | --- | --- | --- | --- | --- | --- | --- | --- | --- | --- | --- | --- | --- | --- | --- | --- | --- | --- | --- | --- | --- | --- | --- | --- | --- | --- |
| L4H12 | T | T | G | G | A | T | C | A | T | T | T | G | T | T | C | T | T | T | T | T | T | A | T | T | T | G | C | T | G | C | G | G | T | A | T | T | G | G | C | A | G | T | G | G | G | C | A | C | C | G | A | T | A | A | C | T | C | A | G | C |

***W I I C S F L F A A V L A*** **V** G **T** D N S A

E N **D** K M A K W V K S L D G S I S Y L R

| G2C8 | A | G | A | A | A | A | T | G | A | T | A | A | A | A | T | G | G | C | T | A | A | A | T | G | G | G | T | C | A | A | G | T | C | T | C | T | T | G | A | T | G | G | A | A | G | T | A | T | A | T | C | C | T | A | T | T | T | A | A | G |
| --- | --- | --- | --- | --- | --- | --- | --- | --- | --- | --- | --- | --- | --- | --- | --- | --- | --- | --- | --- | --- | --- | --- | --- | --- | --- | --- | --- | --- | --- | --- | --- | --- | --- | --- | --- | --- | --- | --- | --- | --- | --- | --- | --- | --- | --- | --- | --- | --- | --- | --- | --- | --- | --- | --- | --- | --- | --- | --- | --- | --- |
| L4H12 | A | G | A | A | A | A | T | G | A | A | A | A | A | A | T | G | G | C | T | A | A | A | T | G | G | G | T | C | A | A | G | T | C | T | C | T | T | G | A | T | G | G | A | A | G | T | A | T | A | T | C | C | T | A | T | T | T | A | A | G |

E N **E** K M A K W V K S L D G S I S Y L R

S K N F K **N** K E F T D L N **S** Y E W **T** D W

| G2C8 | A | A | G | C | A | A | A | A | A | T | T | T | T | A | A | G | A | A | T | A | A | A | G | A | A | T | T | T | A | C | T | G | A | T | T | T | A | A | A | T | T | C | A | T | A | T | G | A | A | T | G | G | A | C | T | G | A | C | T | G |
| --- | --- | --- | --- | --- | --- | --- | --- | --- | --- | --- | --- | --- | --- | --- | --- | --- | --- | --- | --- | --- | --- | --- | --- | --- | --- | --- | --- | --- | --- | --- | --- | --- | --- | --- | --- | --- | --- | --- | --- | --- | --- | --- | --- | --- | --- | --- | --- | --- | --- | --- | --- | --- | --- | --- | --- | --- | --- | --- | --- | --- |
| L4H12 | A | A | G | C | A | A | A | A | A | T | T | T | T | A | A | G | A | A | C | A | A | A | G | A | A | T | T | T | A | C | T | G | A | T | T | T | A | A | A | T | T | T | A | T | A | T | G | A | A | T | G | G | A | G | T | G | A | C | T | G |

S K N F K **N** K E F T D L N **L** Y E W **S** D W

E K **M** P Y **K** L K A **S** A **A** Y T **Y** K T **E** M L

| G2C8 | G | G | A | A | A | A | A | A | T | G | C | C | T | T | A | C | A | A | A | T | T | A | A | A | A | G | C | T | T | C | T | G | C | T | G | C | G | T | A | T | A | C | A | T | A | C | A | A | G | A | C | A | G | A | A | A | T | G | C | T |
| --- | --- | --- | --- | --- | --- | --- | --- | --- | --- | --- | --- | --- | --- | --- | --- | --- | --- | --- | --- | --- | --- | --- | --- | --- | --- | --- | --- | --- | --- | --- | --- | --- | --- | --- | --- | --- | --- | --- | --- | --- | --- | --- | --- | --- | --- | --- | --- | --- | --- | --- | --- | --- | --- | --- | --- | --- | --- | --- | --- | --- |
| L4H12 | G | G | A | A | A | A | A | A | T | T | C | C | T | T | A | C | A | G | A | T | T | A | A | A | A | G | C | T | G | C | T | G | C | T | T | C | G | T | A | T | A | C | A | T | A | T | A | A | G | A | C | A | G | A | G | A | T | G | C | T |

E K **I** P Y **R** L K A **A** A **S** Y T **Y** K T **E** M L

K K N Y K T N **R** N A D Q F S A T **C** A Y K

| G2C8 | C | A | A | G | A | A | A | A | A | T | T | A | T | A | A | A | A | C | A | A | A | T | C | G | G | A | A | T | G | C | T | G | A | T | C | A | A | T | T | T | A | G | C | G | C | T | A | C | T | T | G | T | G | C | T | T | A | T | A | A |
| --- | --- | --- | --- | --- | --- | --- | --- | --- | --- | --- | --- | --- | --- | --- | --- | --- | --- | --- | --- | --- | --- | --- | --- | --- | --- | --- | --- | --- | --- | --- | --- | --- | --- | --- | --- | --- | --- | --- | --- | --- | --- | --- | --- | --- | --- | --- | --- | --- | --- | --- | --- | --- | --- | --- | --- | --- | --- | --- | --- | --- |
| L4H12 | C | A | A | G | A | A | A | A | A | T | T | A | T | A | A | A | A | C | A | A | A | T | C | G | A | A | A | T | G | C | T | G | A | T | C | A | A | T | T | T | A | G | C | G | C | T | A | C | T | C | G | T | G | C | T | T | A | T | A | A |

K K N Y K T N **R** N A D Q F S A T **R** A Y K

A F **K** E **L** L R K C D **D** I **I** Y F **Q** L T F H

| G2C8 | G | G | C | A | T | T | C | A | A | G | G | A | G | T | T | A | C | T | C | A | G | G | A | A | A | T | G | T | G | A | T | G | A | T | A | T | A | A | T | C | T | A | T | T | T | T | C | A | A | T | T | G | A | C | T | T | T | C | C | A |
| --- | --- | --- | --- | --- | --- | --- | --- | --- | --- | --- | --- | --- | --- | --- | --- | --- | --- | --- | --- | --- | --- | --- | --- | --- | --- | --- | --- | --- | --- | --- | --- | --- | --- | --- | --- | --- | --- | --- | --- | --- | --- | --- | --- | --- | --- | --- | --- | --- | --- | --- | --- | --- | --- | --- | --- | --- | --- | --- | --- | --- |
| L4H12 | G | G | C | A | T | T | C | A | A | A | G | A | G | T | T | T | C | T | C | A | G | G | A | A | A | C | G | A | G | A | T | G | A | A | T | T | T | A | T | G | T | A | T | T | T | T | C | A | T | T | T | G | A | C | T | T | T | C | C | A |

A F **K** E **F** L R K R D **E** F **M** Y F **H** L T F H

**K** D **I** **R** A K Y **N** E I F N D K **A** **N** **Y** L K F

| G2C8 | T | A | A | A | G | A | C | A | T | C | A | G | G | G | C | G | A | A | A | T | A | T | A | A | T | G | A | A | A | T | A | T | T | T | A | A | T | G | A | T | A | A | A | G | C | A | A | A | C | T | A | T | T | T | G | A | A | A | T | T |
| --- | --- | --- | --- | --- | --- | --- | --- | --- | --- | --- | --- | --- | --- | --- | --- | --- | --- | --- | --- | --- | --- | --- | --- | --- | --- | --- | --- | --- | --- | --- | --- | --- | --- | --- | --- | --- | --- | --- | --- | --- | --- | --- | --- | --- | --- | --- | --- | --- | --- | --- | --- | --- | --- | --- | --- | --- | --- | --- | --- | --- |
| L4H12 | T | A | C | A | G | A | C | C | T | C | A | A | G | A | T | G | A | A | A | T | A | T | G | A | T | G | A | A | A | T | A | T | T | T | A | A | T | G | A | T | A | A | A | G | T | A | A | A | A | T | T | T | T | T | G | A | A | A | T | T |

**T** D **L** **K** M K Y **D** E I F N D K **V** **K** **F** L K L

L I D F **V** A **G** L E **D** K D I F V P **E** **T** **S** K

| G2C8 | T | C | T | T | A | T | T | G | A | T | T | T | C | G | T | A | G | C | C | G | G | A | T | T | A | G | A | A | G | A | C | A | A | A | G | A | T | A | T | T | T | T | T | G | T | A | C | C | G | G | A | A | A | C | T | T | C | A | A | A |
| --- | --- | --- | --- | --- | --- | --- | --- | --- | --- | --- | --- | --- | --- | --- | --- | --- | --- | --- | --- | --- | --- | --- | --- | --- | --- | --- | --- | --- | --- | --- | --- | --- | --- | --- | --- | --- | --- | --- | --- | --- | --- | --- | --- | --- | --- | --- | --- | --- | --- | --- | --- | --- | --- | --- | --- | --- | --- | --- | --- | --- |
| L4H12 | A | A | T | T | A | T | T | G | A | T | T | T | C | G | A | A | G | C | C | G | T | A | T | T | A | G | A | A | G | A | G | A | A | A | G | A | T | G | C | T | T | C | A | T | T | C | A | T | G | G | A | T | A | T | T | T | T | A | T | A |

I I D F **E** A **V** L E **E** K D A S F M **D** **I** **L** Y

I P R L A **E** **A** F R **N** Y S K **S** K A S **G** **A** G

| G2C8 | A | A | T | A | C | C | A | C | G | A | C | T | T | G | C | T | G | A | A | G | C | T | T | T | T | C | G | C | A | A | T | T | A | T | T | C | T | A | A | A | A | G | C | A | A | A | G | C | C | T | C | A | G | G | G | G | C | A | G | G |
| --- | --- | --- | --- | --- | --- | --- | --- | --- | --- | --- | --- | --- | --- | --- | --- | --- | --- | --- | --- | --- | --- | --- | --- | --- | --- | --- | --- | --- | --- | --- | --- | --- | --- | --- | --- | --- | --- | --- | --- | --- | --- | --- | --- | --- | --- | --- | --- | --- | --- | --- | --- | --- | --- | --- | --- | --- | --- | --- | --- | --- |
| L4H12 | C | A | T | A | C | C | A | T | T | C | G | T | T | G | C | T | G | A | T | G | T | T | T | T | T | C | G | C | G | A | T | T | A | T | C | T | T | A | A | A | G | G | C | A | A | A | G | C | C | T | C | A | G | A | G | T | C | A | G | G |

I P F V A **D** **V** F R **D** Y L K **G** K A S **E** **S** G

**G** S S S T S

| G2C8 | T | G | G | T | T | C | A | T | C | G | T | C | A | A | C | C | T | C | A | **T** | **G** | **A** | T | T | T | T | C | - | - | A | A | G | A | T | T | C | T | G | A | T | T | T | T | T | C | A | T | C | G | A | T | T | T | T | C | T | T | T | G | G |
| --- | --- | --- | --- | --- | --- | --- | --- | --- | --- | --- | --- | --- | --- | --- | --- | --- | --- | --- | --- | --- | --- | --- | --- | --- | --- | --- | --- | --- | --- | --- | --- | --- | --- | --- | --- | --- | --- | --- | --- | --- | --- | --- | --- | --- | --- | --- | --- | --- | --- | --- | --- | --- | --- | --- | --- | --- | --- | --- | --- | --- |
| L4H12 | T | G | C | T | T | C | A | T | C | G | T | C | A | A | C | C | T | C | A | **T** | **G** | **A** | T | T | T | T | C | T | G | A | T | G | A | T | T | C | T | G | G | T | T | T | T | T | C | A | T | C | G | A | T | T | T | T | T | T | T | T | G | G |

**A** S S S T S

| G2C8 | T | A | T | C | T | T | G | T | G | G | C | C | T | T | A | G | A | C | A | G | C | A | T | T | T | G | A | T | C | G | C | T | A | A | T | C | A | G | A | A | T | G | A | A | A | A | A | T | A | A | A | A | T | A | A | A | A | T | A | A |
| --- | --- | --- | --- | --- | --- | --- | --- | --- | --- | --- | --- | --- | --- | --- | --- | --- | --- | --- | --- | --- | --- | --- | --- | --- | --- | --- | --- | --- | --- | --- | --- | --- | --- | --- | --- | --- | --- | --- | --- | --- | --- | --- | --- | --- | --- | --- | --- | --- | --- | --- | --- | --- | --- | --- | --- | --- | --- | --- | --- | --- |
| L4H12 | T | A | T | C | T | T | G | T | G | G | C | C | T | T | A | G | A | C | A | G | C | A | T | T | T | G | A | T | C | G | C | T | A | A | T | C | A | G | A | A | T | G | A | A | A | A | A | T | A | A | A | A | T | A | A | A | A | T | A | A |

| G2C8 | A | A | T | T | C | A | A | T | T |  |  |  |  |  |  |  |  |  |  |  |  |  |  |  |  |  |  |  |  |  |  |  |  |  |  |  |  |  |  |  |  |  |  |  |  |  |  |  |  |  |  |  |  |  |  |  |  |  |  |  |
| --- | --- | --- | --- | --- | --- | --- | --- | --- | --- | --- | --- | --- | --- | --- | --- | --- | --- | --- | --- | --- | --- | --- | --- | --- | --- | --- | --- | --- | --- | --- | --- | --- | --- | --- | --- | --- | --- | --- | --- | --- | --- | --- | --- | --- | --- | --- | --- | --- | --- | --- | --- | --- | --- | --- | --- | --- | --- | --- | --- | --- |
| L4H12 | A | T | T | T | C | A | A | T | T |  |  |  |  |  |  |  |  |  |  |  |  |  |  |  |  |  |  |  |  |  |  |  |  |  |  |  |  |  |  |  |  |  |  |  |  |  |  |  |  |  |  |  |  |  |  |  |  |  |  |  |

**G**

***M L N S***

| G16C10 | - | - | - | - | T | T | C | A | A | A | A | C | T | T | - | - | - | G | A | A | T | A | C | T | A | A | T | A | A | G | T | G | A | A | G | T | G | A | A | T | T | G | T | T | G | T | A | A | A | **A** | **T** | **G** | T | T | A | A | A | C | T | G |
| --- | --- | --- | --- | --- | --- | --- | --- | --- | --- | --- | --- | --- | --- | --- | --- | --- | --- | --- | --- | --- | --- | --- | --- | --- | --- | --- | --- | --- | --- | --- | --- | --- | --- | --- | --- | --- | --- | --- | --- | --- | --- | --- | --- | --- | --- | --- | --- | --- | --- | --- | --- | --- | --- | --- | --- | --- | --- | --- | --- | --- |
| G29D6 | A | T | A | T | T | T | A | A | A | A | A | C | T | T | T | A | A | A | A | A | T | T | C | T | A | A | T | A | A | G | T | A | A | A | A | - | A | A | A | T | T | A | T | T | T | C | A | A | A | **A** | **T** | **G** | T | T | A | A | A | C | T | C |

***M L N S***

***Q R L I I C C L L F A A*** V F S - - - -

| G16C10 | T | C | A | A | A | G | G | T | T | G | A | T | C | A | T | T | T | G | T | T | G | T | T | T | G | T | T | A | T | T | T | G | C | T | G | C | G | G | T | A | T | T | - | - | C | A | G | T | - | - | - | - | - | - | - | - | - | - | - | - |
| --- | --- | --- | --- | --- | --- | --- | --- | --- | --- | --- | --- | --- | --- | --- | --- | --- | --- | --- | --- | --- | --- | --- | --- | --- | --- | --- | --- | --- | --- | --- | --- | --- | --- | --- | --- | --- | --- | --- | --- | --- | --- | --- | --- | --- | --- | --- | --- | --- | --- | --- | --- | --- | --- | --- | --- | --- | --- | --- | --- | --- |
| G29D6 | C | C | A | A | A | G | G | T | T | G | A | T | C | G | T | T | T | G | T | T | G | T | T | T | G | T | T | A | T | T | T | G | C | T | G | C | G | G | T | A | T | T | G | G | C | A | A | T | G | G | C | C | A | C | A | G | A | A | G | A |

***Q R L I V C C L L F A A*** V L A M A T E E

- - - **K** **D** V W E Q S L D D T M **D** Y **I** K L

| G16C10 | - | - | - | - | - | - | - | - | - | - | A | A | A | G | A | T | G | T | A | T | G | G | G | A | A | C | A | G | T | C | T | C | T | T | G | A | T | G | A | T | A | C | T | A | T | G | G | A | C | T | A | T | A | T | A | A | A | A | C | T |
| --- | --- | --- | --- | --- | --- | --- | --- | --- | --- | --- | --- | --- | --- | --- | --- | --- | --- | --- | --- | --- | --- | --- | --- | --- | --- | --- | --- | --- | --- | --- | --- | --- | --- | --- | --- | --- | --- | --- | --- | --- | --- | --- | --- | --- | --- | --- | --- | --- | --- | --- | --- | --- | --- | --- | --- | --- | --- | --- | --- | --- |
| G29D6 | A | G | A | A | A | A | A | A | A | A | A | A | G | A | A | T | G | T | A | T | G | G | G | A | A | A | G | G | T | C | T | C | T | T | G | A | T | A | A | C | A | C | T | T | T | T | A | A | C | T | A | T | A | T | C | A | A | A | G | C |

E K K **K** **N** V W E R S L D N T F **N** Y **I** K A

**K** G L T Y E G N **A** R M **N** G F D W A D W K

| G16C10 | A | A | A | A | G | G | T | T | T | G | A | C | G | T | A | C | G | A | A | G | G | A | A | A | T | G | C | T | A | G | A | A | T | G | A | A | C | G | G | A | T | T | T | G | A | T | T | G | G | G | C | A | G | A | C | T | G | G | A | A |
| --- | --- | --- | --- | --- | --- | --- | --- | --- | --- | --- | --- | --- | --- | --- | --- | --- | --- | --- | --- | --- | --- | --- | --- | --- | --- | --- | --- | --- | --- | --- | --- | --- | --- | --- | --- | --- | --- | --- | --- | --- | --- | --- | --- | --- | --- | --- | --- | --- | --- | --- | --- | --- | --- | --- | --- | --- | --- | --- | --- | --- |
| G29D6 | C | A | A | C | A | A | T | T | T | G | A | C | G | T | A | C | G | A | A | A | A | A | A | A | T | G | A | T | A | A | T | T | A | T | A | A | A | G | G | A | T | T | T | A | A | G | T | G | G | G | C | A | G | A | C | T | G | G | G | T |

**N** N L T Y E K N **D** N Y **K** G F K W A D W V

**N** I P S E H K K I **A** **V** **T** A Y Q K **L** M **R** H

| G16C10 | A | A | A | T | A | T | A | C | C | T | T | C | C | G | A | A | C | A | T | A | A | A | A | A | G | A | T | T | G | C | C | G | T | G | A | C | G | G | C | A | T | A | T | C | A | G | A | A | A | T | T | G | A | T | G | A | G | A | C | A |
| --- | --- | --- | --- | --- | --- | --- | --- | --- | --- | --- | --- | --- | --- | --- | --- | --- | --- | --- | --- | --- | --- | --- | --- | --- | --- | --- | --- | --- | --- | --- | --- | --- | --- | --- | --- | --- | --- | --- | --- | --- | --- | --- | --- | --- | --- | --- | --- | --- | --- | --- | --- | --- | --- | --- | --- | --- | --- | --- | --- | --- |
| G29D6 | A | C | A | T | A | T | A | C | C | T | T | C | C | G | A | A | T | T | G | A | A | A | G | T | G | T | C | T | A | C | C | T | T | G | A | A | G | G | C | A | T | A | T | C | A | G | A | A | A | T | C | G | A | T | G | A | A | A | A | A |

**H** I P S E L K V S **T** **L** **K** A Y Q K **S** M **K** K

K **D** **P** **K** Q A **R** **S** **K** **Q** I A N Y A F **K** A F Q

| G16C10 | T | A | A | A | G | A | T | C | C | T | A | A | A | C | A | G | G | C | T | C | G | T | A | G | T | A | A | G | C | A | A | A | T | C | G | C | T | A | A | T | T | A | T | G | C | T | T | T | T | A | A | G | G | C | A | T | T | T | C | A |
| --- | --- | --- | --- | --- | --- | --- | --- | --- | --- | --- | --- | --- | --- | --- | --- | --- | --- | --- | --- | --- | --- | --- | --- | --- | --- | --- | --- | --- | --- | --- | --- | --- | --- | --- | --- | --- | --- | --- | --- | --- | --- | --- | --- | --- | --- | --- | --- | --- | --- | --- | --- | --- | --- | --- | --- | --- | --- | --- | --- | --- |
| G29D6 | A | A | A | A | A | A | T | C | C | A | A | A | T | C | A | G | G | C | T | C | C | T | A | A | T | C | A | G | C | G | A | A | T | C | G | C | T | A | A | T | T | A | T | G | C | T | T | T | T | G | A | G | G | C | A | T | T | C | G | A |

K **N** **P** **N** Q A **P** **N** **Q** **R** I A N Y A F **E** A F D

**E** F H **T** **A** Q S M Y **L** **G** P H **L** **L** T A D K E

| G16C10 | A | G | A | A | T | T | T | C | A | C | A | C | G | G | C | A | C | A | A | T | C | T | A | T | G | T | A | T | C | T | C | G | G | A | C | C | G | C | A | T | T | T | G | T | T | A | A | C | A | G | C | T | G | A | C | A | A | A | G | A |
| --- | --- | --- | --- | --- | --- | --- | --- | --- | --- | --- | --- | --- | --- | --- | --- | --- | --- | --- | --- | --- | --- | --- | --- | --- | --- | --- | --- | --- | --- | --- | --- | --- | --- | --- | --- | --- | --- | --- | --- | --- | --- | --- | --- | --- | --- | --- | --- | --- | --- | --- | --- | --- | --- | --- | --- | --- | --- | --- | --- | --- |
| G29D6 | C | A | A | A | T | T | T | C | A | C | T | C | G | G | A | A | C | A | A | A | T | T | G | A | A | T | A | T | A | T | C | A | G | A | T | T | G | C | A | T | T | G | G | T | C | A | A | C | A | G | C | T | G | A | C | G | C | T | T | A |

**K** F H **S** **E** Q I E Y **I** **R** L H **W** **S** T A D A Y

L K K R C L G E F E **N** K **Q** T F L K F I

| G16C10 | A | C | T | C | A | A | A | A | A | A | C | G | A | T | G | T | - | C | T | G | G | G | A | G | A | A | T | T | T | G | A | A | A | A | C | A | A | A | C | A | A | A | C | T | T | T | C | T | T | G | A | A | G | T | T | T | A | T | T | A |
| --- | --- | --- | --- | --- | --- | --- | --- | --- | --- | --- | --- | --- | --- | --- | --- | --- | --- | --- | --- | --- | --- | --- | --- | --- | --- | --- | --- | --- | --- | --- | --- | --- | --- | --- | --- | --- | --- | --- | --- | --- | --- | --- | --- | --- | --- | --- | --- | --- | --- | --- | --- | --- | --- | --- | --- | --- | --- | --- | --- | --- |
| G29D6 | T | C | T | C | A | A | A | G | T | - | C | A | A | T | A | T | A | C | T | G | G | G | A | G | A | A | T | T | T | G | A | A | A | G | C | A | A | A | C | C | A | A | C | T | T | T | C | T | T | G | A | A | G | T | T | T | A | T | T | A |

L K V N I L G E F E **S** K **P** T F L K F I

I **G** I R **T** A V K D K F G N Q D P I P **A** E

| G16C10 | T | T | G | G | T | A | T | T | C | G | T | A | C | C | G | C | A | G | T | G | A | A | A | G | A | T | A | A | A | T | T | T | G | G | C | A | A | T | C | A | A | G | A | T | C | C | A | A | T | A | C | C | A | G | C | A | G | A | A | T |
| --- | --- | --- | --- | --- | --- | --- | --- | --- | --- | --- | --- | --- | --- | --- | --- | --- | --- | --- | --- | --- | --- | --- | --- | --- | --- | --- | --- | --- | --- | --- | --- | --- | --- | --- | --- | --- | --- | --- | --- | --- | --- | --- | --- | --- | --- | --- | --- | --- | --- | --- | --- | --- | --- | --- | --- | --- | --- | --- | --- | --- |
| G29D6 | T | T | G | A | T | A | T | T | C | G | T | G | C | C | G | C | A | G | T | G | A | A | A | G | A | T | A | A | A | T | T | T | G | G | C | G | A | C | C | G | T | G | A | T | C | C | A | A | C | C | C | C | A | A | C | A | G | A | A | T |

I **D** I R **A** A V K D K F G D R D P T P **T** E

F A E V L A K Y Y K D K **D** S **G** A **G** A S S

| G16C10 | T | T | G | C | G | G | A | A | G | T | T | C | T | T | G | C | A | A | A | G | T | A | T | T | A | T | A | A | A | G | A | C | A | A | A | G | A | T | T | C | A | G | G | G | G | C | A | G | G | C | G | C | T | T | C | A | T | C | A | T |
| --- | --- | --- | --- | --- | --- | --- | --- | --- | --- | --- | --- | --- | --- | --- | --- | --- | --- | --- | --- | --- | --- | --- | --- | --- | --- | --- | --- | --- | --- | --- | --- | --- | --- | --- | --- | --- | --- | --- | --- | --- | --- | --- | --- | --- | --- | --- | --- | --- | --- | --- | --- | --- | --- | --- | --- | --- | --- | --- | --- | --- |
| G29D6 | T | T | G | C | G | G | A | A | G | T | T | C | T | T | G | C | A | A | A | G | T | A | T | T | A | T | A | A | A | G | A | C | A | A | A | G | A | G | T | C | A | G | A | G | G | C | A | G | A | C | G | C | T | T | C | A | T | C | A | T |

F A E V L A K Y Y K D K **E** S **E** A **D** A S S

S T **S**

| G16C10 | C | A | A | C | C | T | C | A | **T** | **G** | **A** | T | T | A | T | C | A | A | G | A | C | T | C | C | T | - | C | C | T | G | C | T | C | T | G | C | T | G | T | G | G | T | C | T | T | C | T | T | C | A | G | C | A | T | C | T | C | C | G | G |
| --- | --- | --- | --- | --- | --- | --- | --- | --- | --- | --- | --- | --- | --- | --- | --- | --- | --- | --- | --- | --- | --- | --- | --- | --- | --- | --- | --- | --- | --- | --- | --- | --- | --- | --- | --- | --- | --- | --- | --- | --- | --- | --- | --- | --- | --- | --- | --- | --- | --- | --- | --- | --- | --- | --- | --- | --- | --- | --- | --- | --- |
| G29D6 | C | A | A | C | C | T | T | A | **T** | **G** | **A** | T | T | T | T | C | A | A | G | G | C | T | C | T | G | G | C | C | T | G | G | T | C | T | G | T | T | T | T | G | G | T | C | T | - | - | - | T | C | G | G | T | A | T | C | T | T | C | G | G |

S T **L**

| G16C10 | G | G | G | C | C | T | C | A | G | A | C | A | - | C | A | T | A | T | G | A | T | C | G | T | - | - | - | - | - | - | - | A | A | T | G | A | G | A | A | A | T | - | - | A | A | A | A | T | G | A | G | A | T | A | T | A | A | T | T | T |
| --- | --- | --- | --- | --- | --- | --- | --- | --- | --- | --- | --- | --- | --- | --- | --- | --- | --- | --- | --- | --- | --- | --- | --- | --- | --- | --- | --- | --- | --- | --- | --- | --- | --- | --- | --- | --- | --- | --- | --- | --- | --- | --- | --- | --- | --- | --- | --- | --- | --- | --- | --- | --- | --- | --- | --- | --- | --- | --- | --- | --- |
| G29D6 | T | G | G | C | C | T | T | A | G | A | C | A | A | C | A | T | T | T | G | A | T | C | G | C | T | A | A | T | C | G | A | A | A | T | G | A | A | A | A | A | T | G | A | A | A | A | A | T | A | A | A | A | T | A | A | A | A | T | T | T |

| G16C10 | - | C | C | T | C | C | A | C | A | T | A | T | A | T | A | T | C | A |  |  |  |  |  |  |  |  |  |  |  |  |  |  |  |  |  |  |  |  |  |  |  |  |  |  |  |  |  |  |  |  |  |  |  |  |  |  |  |  |  |  |
| --- | --- | --- | --- | --- | --- | --- | --- | --- | --- | --- | --- | --- | --- | --- | --- | --- | --- | --- | --- | --- | --- | --- | --- | --- | --- | --- | --- | --- | --- | --- | --- | --- | --- | --- | --- | --- | --- | --- | --- | --- | --- | --- | --- | --- | --- | --- | --- | --- | --- | --- | --- | --- | --- | --- | --- | --- | --- | --- | --- | --- |
| G29D6 | A | C | T | T | C | C | A | A | A | T | A | C | A | T | T | T | C | A |  |  |  |  |  |  |  |  |  |  |  |  |  |  |  |  |  |  |  |  |  |  |  |  |  |  |  |  |  |  |  |  |  |  |  |  |  |  |  |  |  |  |

**H**

***M F N S***

| G9B3 | A | T | C | A | T | T | C | A | A | A | A | C | T | T | G | A | A | T | A | C | T | A | A | T | A | A | G | T | G | A | A | G | T | G | A | A | G | A | A | T | T | G | T | T | G | C | A | A | A | **A** | **T** | **G** | T | T | T | A | A | C | T | C |
| --- | --- | --- | --- | --- | --- | --- | --- | --- | --- | --- | --- | --- | --- | --- | --- | --- | --- | --- | --- | --- | --- | --- | --- | --- | --- | --- | --- | --- | --- | --- | --- | --- | --- | --- | --- | --- | --- | --- | --- | --- | --- | --- | --- | --- | --- | --- | --- | --- | --- | --- | --- | --- | --- | --- | --- | --- | --- | --- | --- | --- |
| L1C12 | - | - | - | - | T | T | C | A | A | A | A | C | T | T | G | A | A | T | A | C | T | A | A | T | A | A | G | T | G | A | A | G | T | G | A | A | G | A | A | T | T | G | T | C | G | C | A | A | A | **A** | **T** | **G** | T | T | T | A | A | C | T | C |

***M F N S***

***Q K L I I C C L L F A A*** V W V Q S L K A

| G9B3 | T | C | A | A | A | A | G | T | T | G | A | T | C | A | T | T | T | G | T | T | G | T | C | T | G | T | T | A | T | T | T | G | C | T | G | C | C | G | T | A | T | G | G | G | T | G | C | A | G | T | C | A | T | T | A | A | A | A | G | C |
| --- | --- | --- | --- | --- | --- | --- | --- | --- | --- | --- | --- | --- | --- | --- | --- | --- | --- | --- | --- | --- | --- | --- | --- | --- | --- | --- | --- | --- | --- | --- | --- | --- | --- | --- | --- | --- | --- | --- | --- | --- | --- | --- | --- | --- | --- | --- | --- | --- | --- | --- | --- | --- | --- | --- | --- | --- | --- | --- | --- | --- |
| L1C12 | T | C | A | A | A | A | G | T | T | G | A | T | C | A | T | T | T | G | T | T | G | T | C | T | G | T | T | A | T | T | T | G | C | T | G | C | C | G | T | A | T | G | G | G | T | G | C | A | G | T | C | A | T | T | A | A | A | A | G | C |

***Q K L I I C C L L F A A*** V W V Q S L K A

A L T P G L S I Y L D E D K D R D L L V

| G9B3 | C | G | C | A | C | T | A | A | C | A | C | C | A | G | G | T | T | T | A | T | C | G | A | T | A | T | A | T | C | T | C | G | A | T | G | A | G | G | A | T | A | A | A | G | A | T | C | G | C | G | A | C | T | T | A | C | T | A | G | T |
| --- | --- | --- | --- | --- | --- | --- | --- | --- | --- | --- | --- | --- | --- | --- | --- | --- | --- | --- | --- | --- | --- | --- | --- | --- | --- | --- | --- | --- | --- | --- | --- | --- | --- | --- | --- | --- | --- | --- | --- | --- | --- | --- | --- | --- | --- | --- | --- | --- | --- | --- | --- | --- | --- | --- | --- | --- | --- | --- | --- | --- |
| L1C12 | C | G | C | A | C | T | A | A | C | A | C | C | A | G | G | T | T | T | A | T | C | G | A | T | A | T | A | T | C | T | C | G | A | T | G | A | G | G | A | T | A | A | A | G | A | T | C | G | C | G | A | C | T | T | A | C | T | A | G | T |

A L T P G L S I Y L D E D K D R D L L V

P P G S Q Y E K Y L I S S A N R Y S K I

| G9B3 | T | C | C | A | C | C | A | G | G | G | A | G | C | C | A | A | T | A | T | G | A | A | A | A | A | T | A | T | C | T | C | A | T | A | T | C | A | T | C | C | G | C | C | A | A | T | C | G | A | T | A | T | T | C | G | A | A | A | A | T |
| --- | --- | --- | --- | --- | --- | --- | --- | --- | --- | --- | --- | --- | --- | --- | --- | --- | --- | --- | --- | --- | --- | --- | --- | --- | --- | --- | --- | --- | --- | --- | --- | --- | --- | --- | --- | --- | --- | --- | --- | --- | --- | --- | --- | --- | --- | --- | --- | --- | --- | --- | --- | --- | --- | --- | --- | --- | --- | --- | --- | --- |
| L1C12 | T | C | C | A | C | C | A | G | G | G | A | G | C | C | A | A | T | A | T | G | A | A | A | A | A | T | A | T | C | T | C | A | T | A | T | C | A | T | C | C | G | C | C | A | A | T | C | G | A | T | A | T | T | C | G | A | A | A | A | T |

P P G S Q Y E K Y L I S S A N R Y S K I

F H R G E H E I K S I R L I E Y Y L L **V**

| G9B3 | A | T | T | C | C | A | T | C | G | C | G | G | C | G | A | A | C | A | T | G | A | A | A | T | A | A | A | G | T | C | A | A | T | C | A | G | A | T | T | G | A | T | T | G | A | A | T | A | T | T | A | T | T | T | A | C | T | T | G | T |
| --- | --- | --- | --- | --- | --- | --- | --- | --- | --- | --- | --- | --- | --- | --- | --- | --- | --- | --- | --- | --- | --- | --- | --- | --- | --- | --- | --- | --- | --- | --- | --- | --- | --- | --- | --- | --- | --- | --- | --- | --- | --- | --- | --- | --- | --- | --- | --- | --- | --- | --- | --- | --- | --- | --- | --- | --- | --- | --- | --- | --- |
| L1C12 | A | T | T | C | C | A | T | C | G | C | G | G | C | G | A | A | C | A | T | G | A | A | A | T | A | A | A | G | T | C | A | A | T | C | A | G | A | T | T | G | A | T | T | G | A | A | T | A | T | T | A | T | T | T | A | C | T | T | T | T |

F H R G E H E I K S I R L I E Y Y L L **F**

N **F** **Y** N Y K **D** **T** A **K** D **F** **G** F D D T K F W

| G9B3 | T | A | A | T | T | T | T | T | A | T | A | A | T | T | A | T | A | A | A | G | A | T | A | C | G | G | C | G | A | A | A | G | A | T | T | T | C | G | G | A | T | T | C | G | A | C | G | A | T | A | C | A | A | A | A | T | T | T | T | G |
| --- | --- | --- | --- | --- | --- | --- | --- | --- | --- | --- | --- | --- | --- | --- | --- | --- | --- | --- | --- | --- | --- | --- | --- | --- | --- | --- | --- | --- | --- | --- | --- | --- | --- | --- | --- | --- | --- | --- | --- | --- | --- | --- | --- | --- | --- | --- | --- | --- | --- | --- | --- | --- | --- | --- | --- | --- | --- | --- | --- | --- |
| L1C12 | T | G | A | A | A | T | T | C | A | T | A | A | T | T | A | T | A | A | A | A | A | T | G | C | G | G | C | G | G | A | A | A | A | A | T | A | C | G | A | A | T | T | C | G | A | C | G | A | T | A | C | A | A | A | A | T | T | T | T | G |

E **I** **H** N Y K **N** **A** A **E** K **Y** **E**  F D D T K F W

S W W G **D** **Q** I G F D K K S E L **G** A A F E

| G9B3 | G | A | G | T | T | G | G | T | G | G | G | G | A | G | A | C | C | A | A | A | T | T | G | G | T | T | T | C | G | A | T | A | A | A | A | A | A | T | C | G | G | A | G | C | T | A | G | G | T | G | C | T | G | C | A | T | T | T | G | A |
| --- | --- | --- | --- | --- | --- | --- | --- | --- | --- | --- | --- | --- | --- | --- | --- | --- | --- | --- | --- | --- | --- | --- | --- | --- | --- | --- | --- | --- | --- | --- | --- | --- | --- | --- | --- | --- | --- | --- | --- | --- | --- | --- | --- | --- | --- | --- | --- | --- | --- | --- | --- | --- | --- | --- | --- | --- | --- | --- | --- | --- |
| L1C12 | G | A | G | T | T | G | G | T | G | G | G | G | A | A | A | C | G | A | A | A | T | T | G | G | T | T | T | C | G | A | T | A | A | A | A | A | A | T | C | G | G | A | G | C | T | A | G | G | C | G | C | T | G | C | A | T | T | T | G | A |

S W W G **N** **E** I G F D K K S E L **G** A A F E

**L** K A **I** **K** I P **K** I Q **L** **R** T D E **N** **L** D **K** **Q**

| G9B3 | G | T | T | G | A | A | A | G | C | G | A | T | A | A | A | A | A | T | A | C | C | A | A | A | G | A | T | T | C | A | A | C | T | G | C | G | A | A | C | C | G | A | T | G | A | G | A | A | T | T | T | A | G | A | C | A | A | G | C | A |
| --- | --- | --- | --- | --- | --- | --- | --- | --- | --- | --- | --- | --- | --- | --- | --- | --- | --- | --- | --- | --- | --- | --- | --- | --- | --- | --- | --- | --- | --- | --- | --- | --- | --- | --- | --- | --- | --- | --- | --- | --- | --- | --- | --- | --- | --- | --- | --- | --- | --- | --- | --- | --- | --- | --- | --- | --- | --- | --- | --- | --- |
| L1C12 | G | C | T | G | A | A | A | G | C | G | A | T | G | G | A | A | A | T | A | C | G | T | A | G | G | G | A | T | G | G | T | C | G | G | C | A | A | T | T | A | G | T | T | G | A | G | A | C | T | T | T | G | A | A | A | A | A | A | C | G |

**L** K A **M** **E** I R **R** D G **R** **Q** L V E **T** **L** K **K** **R**

F V **R** **F** A R E T L D S Y I S K I N **K** E N

| G9B3 | A | T | T | T | G | T | C | A | G | A | T | T | C | G | C | T | C | G | T | G | A | A | A | C | A | T | T | G | G | A | T | T | C | A | T | A | T | A | T | A | A | G | T | A | A | A | A | T | C | A | A | T | A | A | G | G | A | A | A | A |
| --- | --- | --- | --- | --- | --- | --- | --- | --- | --- | --- | --- | --- | --- | --- | --- | --- | --- | --- | --- | --- | --- | --- | --- | --- | --- | --- | --- | --- | --- | --- | --- | --- | --- | --- | --- | --- | --- | --- | --- | --- | --- | --- | --- | --- | --- | --- | --- | --- | --- | --- | --- | --- | --- | --- | --- | --- | --- | --- | --- | --- |
| L1C12 | A | T | T | T | T | C | C | A | A | A | T | G | C | G | C | T | C | G | T | G | A | A | A | C | A | T | T | G | G | A | T | T | C | A | T | A | T | A | T | A | A | G | T | A | A | A | A | T | G | G | A | A | G | A | G | G | A | A | A | A |

F S **K** **C** A R E T L D S Y I S K M E **E** E N

N I V **T** P F Q K E W Y K F **Q** **T** A V **N** A

| G9B3 | C | A | A | T | A | T | C | G | T | A | A | C | A | C | C | T | T | T | C | C | A | A | A | A | A | G | A | G | T | G | G | T | A | C | A | A | A | T | T | C | C | A | A | A | C | A | G | C | T | G | T | T | A | A | T | G | C | T | A | - |
| --- | --- | --- | --- | --- | --- | --- | --- | --- | --- | --- | --- | --- | --- | --- | --- | --- | --- | --- | --- | --- | --- | --- | --- | --- | --- | --- | --- | --- | --- | --- | --- | --- | --- | --- | --- | --- | --- | --- | --- | --- | --- | --- | --- | --- | --- | --- | --- | --- | --- | --- | --- | --- | --- | --- | --- | --- | --- | --- | --- | --- |
| L1C12 | C | A | A | T | A | T | C | G | T | A | A | C | G | C | C | T | T | T | C | C | A | A | A | A | A | G | A | G | T | G | G | T | A | C | A | A | A | T | T | C | C | G | A | A | A | A | G | C | T | G | T | T | G | A | G | G | C | T | G | C |

N I V **T** P F Q K E W Y K F **R** **K** A V **E** A A

K F Q I N N P T S - S E **H** **R** F **N** I **G** M Q N

| G9B3 | A | G | T | T | C | C | A | G | A | T | C | A | A | T | A | A | C | C | C | A | A | C | A | T | C | - | - | A | A | G | T | G | A | A | C | A | T | C | G | G | T | T | C | A | A | C | A | T | C | G | G | G | A | T | G | C | A | A | A | A |
| --- | --- | --- | --- | --- | --- | --- | --- | --- | --- | --- | --- | --- | --- | --- | --- | --- | --- | --- | --- | --- | --- | --- | --- | --- | --- | --- | --- | --- | --- | --- | --- | --- | --- | --- | --- | --- | --- | --- | --- | --- | --- | --- | --- | --- | --- | --- | --- | --- | --- | --- | --- | --- | --- | --- | --- | --- | --- | --- | --- | --- |
| L1C12 | A | G | A | T | C | C | G | G | T | T | C | T | A | C | A | A | C | A | C | A | A | T | A | T | C | C | C | A | A | C | A | T | C | A | A | A | T | C | G | A | T | T | C | T | A | C | A | T | C | G | G | A | A | T | G | C | A | A | A | A |

D P V L Q H N I P T S **N** **R** F **Y** I **G** M Q N

K

| G9B3 | T | A | A | A | **T** | **A** | **A** | A | A | A | T | A | A | A | A | T | T | C | A | A | T | T | C | G | C | G | T | G | G | A | C | A | T | T | C | C | A | C | G | A | C | A | T | A | T | T | T | C | A | T | A | G | A | T | T | T | C | C | A | T |
| --- | --- | --- | --- | --- | --- | --- | --- | --- | --- | --- | --- | --- | --- | --- | --- | --- | --- | --- | --- | --- | --- | --- | --- | --- | --- | --- | --- | --- | --- | --- | --- | --- | --- | --- | --- | --- | --- | --- | --- | --- | --- | --- | --- | --- | --- | --- | --- | --- | --- | --- | --- | --- | --- | --- | --- | --- | --- | --- | --- | --- |
| L1C12 | T | A | A | A | **T** | **A** | **A** | A | A | A | T | A | A | A | A | T | T | C | A | A | T | T | C | G | C | G | T | G | G | A | C | A | T | T | C | C | A | C | G | A | C | A | T | A | T | T | T | C | A | T | A | G | A | T | T | T | C | C | A | T |

K

| G9B3 | T | T | T | C | G | T | G | G | A | A | T | A | T | T | G | T | G | A | A | C | A | A | T | A | T | C | C | C | A | T | T | T | - | C | A | T | T | G | T | A | A | C | A | C | C | T | A | A | T | A | T | A | A | G | A | A | A | T | G | A |
| --- | --- | --- | --- | --- | --- | --- | --- | --- | --- | --- | --- | --- | --- | --- | --- | --- | --- | --- | --- | --- | --- | --- | --- | --- | --- | --- | --- | --- | --- | --- | --- | --- | --- | --- | --- | --- | --- | --- | --- | --- | --- | --- | --- | --- | --- | --- | --- | --- | --- | --- | --- | --- | --- | --- | --- | --- | --- | --- | --- | --- |
| L1C12 | T | T | T | C | G | T | G | G | A | A | T | A | T | T | G | T | G | A | A | C | A | A | T | A | T | C | C | C | A | T | T | T | T | C | A | T | T | G | T | A | A | C | A | C | C | T | A | A | T | A | T | A | A | G | G | A | A | T | G | A |

| G9B3 | T | G | A | A | T | A | A | A | A | A | A | G | A | A | T | A | T | T | C | A | G | - | T | T | A | T | T | G | A | A | T | A | A | C | T | C | T | C | T |  |  |  |  |  |  |  |  |  |  |  |  |  |  |  |  |  |  |  |  |  |
| --- | --- | --- | --- | --- | --- | --- | --- | --- | --- | --- | --- | --- | --- | --- | --- | --- | --- | --- | --- | --- | --- | --- | --- | --- | --- | --- | --- | --- | --- | --- | --- | --- | --- | --- | --- | --- | --- | --- | --- | --- | --- | --- | --- | --- | --- | --- | --- | --- | --- | --- | --- | --- | --- | --- | --- | --- | --- | --- | --- | --- |
| L1C12 | T | G | A | A | T | A | A | A | A | A | A | A | A | A | T | A | T | T | C | A | G | G | T | T | A | T | T | G | A | A | T | A | A | C | T | C | T | C | T |  |  |  |  |  |  |  |  |  |  |  |  |  |  |  |  |  |  |  |  |  |

**I**

*M K P S P F I V L - I F*

G7H5 1 AATATGATTTTGAATTTGGTTCAAA**ATG**AAACCGTCTCCGTTCATTGTTTT---GATATT
G8C4 1 -GTATAATCTTGAATTTGGTTCAAA**ATG**AAACTGTCTCTGTTCATTATTTTTTTGATATT
 *M K L S L F I I F L I F*

*A V I I G L C G C A* P P K A E E T Q S A

G7H5 58 TGCTGTTATTATAGGCCTGTGTGGTTGTGCACCACCCAAGGCCGAAGAAACTCAATCTGC
G8C4 60 TGCTGTTATTATAGGCCTGTGTGGTTGTGCACCACCCAAGGCCGAAGGAACTAAATCTGG
 *A* V I I G L C G C A P P K A E G T K S G

T S T K A E S S N A G Q S G N R Y - - -

G7H5 118 TACGAGTACGAAAGCCGAGTCTTCTAATGCGGGTCAGAGCGGAAATCGATA--------T
G8C4 120 TATGGGAACGCAAGCCGAGTCTTCTAATGCGGGTCAGAGAGGAAGTCGAAACAATGGCAT
 M G T Q A E S S N A G Q R G S R N N G I

P P V K M N F E K V F T P S F C K G L Q

G7H5 170 C-CACCGGTGAAGATGAATTTTGAAAAAGTGTTTACTCCTAGTTTTTGTAAAGGTTTGCA
G8C4 180 CTCATCGGCGGAGTTGAACTTTGACAGAAT---TTCTCCTGGTTTTATTAAAGGTTTGCG
 S S A E L N F D R I - S P G F I K G L R

D Q Q S K I E E L S A D L E R F E G Q

G7H5 229 AGATCAGCAATCAAAAATTGAAGAACTTTCGGCAGA-CTTGGAGAGGTTTGAGGGTCAGG
G8C4 237 TGAAGATCAATCAGGATATGAAAAAGTTG-GAGAGATCTTGAAGAGGGCTCAGGATCAGC
 E D Q S G Y E K V G E I L K R A Q D Q

E L K S N Y G T Y S D K K D H K

G7H5 288 AATTGAAGTCAAATTATGGAACATATTCCGACAAAAAGGACCATAAA**TAA**AAATTTGTCC
G8C4 296 AATTGAAGTCAAATTATGGAAAATATTCCGACAAAAAGGCCCATAAT**TAA**AAATTTGTTC
 Q L K S N Y G K Y S D K K A H N

G7H5 348 AGCAAAAGATATGGTTGCATAATAAACGCAAATATAATCATACACGCACA
G8C4 356 AGCAAAAAATTTGGTTGCATAATAAACCCAAAAATAATCATACACGC

**J**

M K

| G10C11 | G | T | C | A | G | T | T | A | A | C | G | T | T | T | A | A | C | T | T | T | C | G | A | A | A | G | A | G | C | A | A | A | C | T | T | A | C | G | A | A | A | A | G | A | A | A | C | T | A | T | C | G | C | G | **A** | **T** | **G** | A | A | G |
| --- | --- | --- | --- | --- | --- | --- | --- | --- | --- | --- | --- | --- | --- | --- | --- | --- | --- | --- | --- | --- | --- | --- | --- | --- | --- | --- | --- | --- | --- | --- | --- | --- | --- | --- | --- | --- | --- | --- | --- | --- | --- | --- | --- | --- | --- | --- | --- | --- | --- | --- | --- | --- | --- | --- | --- | --- | --- | --- | --- | --- |
| G15G6 | - | - | - | - | G | T | T | A | A | C | G | T | T | T | A | A | C | T | T | T | C | G | A | A | A | G | A | G | C | A | A | A | C | T | T | A | C | G | A | A | A | A | G | A | A | A | C | T | A | T | C | G | C | G | **A** | **T** | **G** | A | A | G |

***M K***

***K L F I L L F I T F I V I Q A*** V V P N V

| G10C11 | A | A | A | T | T | G | T | T | C | A | T | T | T | T | G | T | T | A | T | T | T | A | T | A | A | C | A | T | T | T | A | T | T | G | T | C | A | T | T | C | A | G | G | C | A | G | T | T | G | T | T | C | C | A | A | A | T | G | T | G |
| --- | --- | --- | --- | --- | --- | --- | --- | --- | --- | --- | --- | --- | --- | --- | --- | --- | --- | --- | --- | --- | --- | --- | --- | --- | --- | --- | --- | --- | --- | --- | --- | --- | --- | --- | --- | --- | --- | --- | --- | --- | --- | --- | --- | --- | --- | --- | --- | --- | --- | --- | --- | --- | --- | --- | --- | --- | --- | --- | --- | --- |
| G15G6 | A | A | A | T | T | T | T | T | C | T | T | T | T | T | G | T | T | A | T | T | T | A | T | A | A | C | A | T | T | T | A | T | T | G | T | C | A | T | T | C | A | G | G | C | A | G | T | T | G | T | T | C | C | A | A | A | T | G | T | G |

***K F F F L L F I T F I V I Q A*** V V P N V

S A G **K** T D D S G S S S **F** G **L** K K T - -

| G10C11 | T | C | T | G | C | T | G | G | G | A | A | A | A | C | T | G | A | T | G | A | T | A | G | T | G | G | C | A | G | T | T | C | G | A | G | T | T | T | T | G | G | G | C | T | A | A | A | A | A | A | A | A | C | A | - | - | - | - | - | - |
| --- | --- | --- | --- | --- | --- | --- | --- | --- | --- | --- | --- | --- | --- | --- | --- | --- | --- | --- | --- | --- | --- | --- | --- | --- | --- | --- | --- | --- | --- | --- | --- | --- | --- | --- | --- | --- | --- | --- | --- | --- | --- | --- | --- | --- | --- | --- | --- | --- | --- | --- | --- | --- | --- | --- | --- | --- | --- | --- | --- | --- |
| G15G6 | T | C | T | G | C | T | G | G | G | A | C | A | C | A | T | G | A | T | G | A | T | A | G | T | G | G | C | A | G | T | T | C | G | A | G | T | T | C | T | G | G | G | C | A | A | A | A | A | A | A | A | A | C | A | G | G | C | A | G | T |

S A G **T** H D D S G S S S **S** G **Q** K K T G S

- L **K** K **N** L S N **A** K Q G **F** **S** D W K N **S** P

| G10C11 | - | - | - | T | T | A | A | A | A | A | A | A | A | A | C | C | T | T | T | C | G | A | A | T | G | C | T | A | A | G | C | A | A | G | G | G | T | T | C | T | C | C | G | A | T | T | G | G | A | A | A | A | A | C | A | G | C | C | C | A |
| --- | --- | --- | --- | --- | --- | --- | --- | --- | --- | --- | --- | --- | --- | --- | --- | --- | --- | --- | --- | --- | --- | --- | --- | --- | --- | --- | --- | --- | --- | --- | --- | --- | --- | --- | --- | --- | --- | --- | --- | --- | --- | --- | --- | --- | --- | --- | --- | --- | --- | --- | --- | --- | --- | --- | --- | --- | --- | --- | --- | --- |
| G15G6 | A | C | A | T | T | A | A | T | A | A | A | A | A | A | G | C | T | T | T | C | G | G | G | T | G | T | T | A | A | G | C | A | A | G | G | G | T | A | C | T | C | T | G | A | T | T | G | G | A | A | A | A | A | C | G | G | C | C | C | A |

T L **I** K **K** L S G **V** K Q G **Y** **S** D W K N **G** P

T **K** L **S** **Q** A T V M T D S P E L N **S** **P** K A

| G10C11 | A | C | A | A | A | A | C | T | T | T | C | C | C | A | A | G | C | A | A | C | A | G | T | A | A | T | G | A | C | T | G | A | C | A | G | C | C | C | A | G | A | A | C | T | A | A | A | T | T | C | C | C | C | C | A | A | A | G | C | A |
| --- | --- | --- | --- | --- | --- | --- | --- | --- | --- | --- | --- | --- | --- | --- | --- | --- | --- | --- | --- | --- | --- | --- | --- | --- | --- | --- | --- | --- | --- | --- | --- | --- | --- | --- | --- | --- | --- | --- | --- | --- | --- | --- | --- | --- | --- | --- | --- | --- | --- | --- | --- | --- | --- | --- | --- | --- | --- | --- | --- | --- |
| G15G6 | A | C | A | A | G | A | C | T | T | T | C | A | C | G | A | G | C | A | A | C | A | G | T | A | A | T | G | A | C | T | G | A | C | A | G | C | C | C | A | G | A | A | C | T | A | A | A | T | T | C | A | C | A | C | A | A | A | G | C | A |

T **R** L **S** **R** A T V M T D S P E L N **S** **H** K A

**K** T W E **G** N **D** R K L K **V** W **Q** Q E E K N **F**

| G10C11 | A | A | A | A | C | T | T | G | G | G | A | G | G | G | C | A | A | T | G | A | T | A | G | A | A | A | A | T | T | G | A | A | A | G | T | G | T | G | G | C | A | G | C | A | A | G | A | A | G | A | A | A | A | G | A | A | C | T | T | T |
| --- | --- | --- | --- | --- | --- | --- | --- | --- | --- | --- | --- | --- | --- | --- | --- | --- | --- | --- | --- | --- | --- | --- | --- | --- | --- | --- | --- | --- | --- | --- | --- | --- | --- | --- | --- | --- | --- | --- | --- | --- | --- | --- | --- | --- | --- | --- | --- | --- | --- | --- | --- | --- | --- | --- | --- | --- | --- | --- | --- | --- |
| G15G6 | A | G | A | A | C | T | T | G | G | G | A | G | G | A | C | A | A | T | G | T | T | A | G | A | A | A | A | T | T | G | A | A | A | T | T | G | T | G | G | C | G | G | C | A | A | G | A | A | G | A | A | A | A | G | A | A | C | T | T | A |

**R** T W E **D** N **V** R K L K **L** W **R** Q E E K N **L**

L E M P **E** **Q** **N** E **E** R L Q **L** V R E M I N **K**

| G10C11 | C | T | A | G | A | A | A | T | G | C | C | C | G | A | A | C | A | A | A | A | T | G | A | G | G | A | A | C | G | A | T | T | G | C | A | A | C | T | A | G | T | A | C | G | T | G | A | A | A | T | G | A | T | T | A | A | C | A | A | A |
| --- | --- | --- | --- | --- | --- | --- | --- | --- | --- | --- | --- | --- | --- | --- | --- | --- | --- | --- | --- | --- | --- | --- | --- | --- | --- | --- | --- | --- | --- | --- | --- | --- | --- | --- | --- | --- | --- | --- | --- | --- | --- | --- | --- | --- | --- | --- | --- | --- | --- | --- | --- | --- | --- | --- | --- | --- | --- | --- | --- | --- |
| G15G6 | C | T | A | G | A | A | A | T | G | C | C | C | G | G | A | C | G | A | G | A | T | G | A | G | A | A | A | C | G | A | T | T | G | C | A | A | C | A | A | G | T | A | C | G | T | G | A | A | A | T | G | A | T | T | A | A | C | G | A | A |

L E M P **G** **R** **D** E **K** R L Q **Q** V R E M I N **E**

F **K** E V M S K N D P A D **P** **R** Y R L

| G10C11 | T | T | T | A | A | A | G | A | A | G | T | T | A | T | G | A | G | T | A | A | A | A | A | T | G | A | T | C | C | C | G | C | T | G | A | T | C | C | C | C | G | G | T | A | T | C | G | A | T | T | A | **T** | **G** | **A** | T | A | G | A | T | T |
| --- | --- | --- | --- | --- | --- | --- | --- | --- | --- | --- | --- | --- | --- | --- | --- | --- | --- | --- | --- | --- | --- | --- | --- | --- | --- | --- | --- | --- | --- | --- | --- | --- | --- | --- | --- | --- | --- | --- | --- | --- | --- | --- | --- | --- | --- | --- | --- | --- | --- | --- | --- | --- | --- | --- | --- | --- | --- | --- | --- | --- |
| G15G6 | T | T | T | A | T | A | G | A | A | G | T | T | A | T | G | A | G | T | A | A | A | A | A | T | G | A | T | C | C | C | G | C | T | G | A | T | C | C | A | C | G | T | T | A | T | C | G | A | T | T | A | **T** | **G** | **A** | T | A | G | A | T | T |

F **I** E V M S K N D P A D **P** **R** Y R L

| G10C11 | G | T | G | T | T | G | T | C | A | A | A | A | T | G | A | A | A | A | G | A | A | T | C | A | A | T | A | A | A | A | C | C | A | T | A | A | T | C | A | T | T | G | G | G | C | A | G | C | G | T | T | C | C | A | A | A | T | A | C | C |
| --- | --- | --- | --- | --- | --- | --- | --- | --- | --- | --- | --- | --- | --- | --- | --- | --- | --- | --- | --- | --- | --- | --- | --- | --- | --- | --- | --- | --- | --- | --- | --- | --- | --- | --- | --- | --- | --- | --- | --- | --- | --- | --- | --- | --- | --- | --- | --- | --- | --- | --- | --- | --- | --- | --- | --- | --- | --- | --- | --- | --- |
| G15G6 | G | T | G | T | T | G | T | C | A | A | A | A | T | G | A | A | A | A | G | A | A | T | C | A | A | T | A | A | A | A | C | C | A | T | A | A | T | C | A | T | T | G | G | A | C | A | G | C | G | T | T | C | C | A | A | A | T | A | C | C |

**K**

***M K Y S***

G8C1 1 ----------------------TCTAGAGAAAACAAACTAAATTAAA**ATG**AAGTATTCGT
G14G1 1 ATCAGTTTTGAATGAACTTTGTTCTAGAGAAAACAAACTAAATTAAA**ATG**AAGTATTCGT
 ***M K Y S***

***L A F L F V A S T A F L M A S A A*** P K R

G8C1 39 TAGCTTTCCTTTTCGTAGCTTCTACAGCTTTTTTGATGGCCAGCGCTGCACCGAAACGCG
G14G1 61 TAGCTTTCCTTTTCGTAGCTTCTACAGCTTTTTTAATGGCCAGCGCTGCACCGAAACGCG
 ***L A F L F V A S T A F L M A S A A*** P K R

A A Q Q S **A** **A** G **L** - - - - - - - - - - -

G8C1 99 CTGCTCAGCAAAGCGCTGCAGGGTTGC---------------------------------
G14G1 121 CTGCTCAGCAAAGCGGTGAAGGGTCGCACGTTGAAGGGTTGCGCCAGGATCTGGCAAACA
 A A Q Q S **G** **E** G **S** H V E G L R Q D L A N

- - - - P S A P R T E A Q L **L** N E L R **L**

G8C1 126 ------------CCTCTGCTCCACGAACCGAAGCACAGTTGCTCAATGAACTGAGACTGG
G14G1 181 TTAACCTTGAATCCTCTGCTCCACGAACCGAAGCACAGTTGCGCAATGAACTGAGACTCG
 I N L E S S A P R T E A Q L **R** N E L R **L**

A Q **N** A L **E** **G** L G R R V P D T **D** L P T **E**

G8C1 174 CTCAAAATGCACTCGAGGGACTTGGACGCCGCGTACCGGATACCGATCTACCTACGGAAC
G14G1 241 CTCAACATGCACTCGATGTACTTGGACGCCGCGTACCGGATCTCAATCTATC---GCAAC
 A Q **H** A L **D** **V** L G R R V P D L **N** L S - **Q**

**Q** E K L A D A E Q L R A A R L R **L** A R A

G8C1 234 AGGAGAAATTGGCAGATGCTGAACAGTTGCGCGCTGCACGATTGCGCCTTGCCCGGGCAC
G14G1 298 ACGAGAAATTGGCAGATGCTGAACAGTTGCGCAAAGCAAAGGAACGCGTTGAGCGGGCAA
 **H** E K L A D A E Q L R K A K E R **V** E R A

R A A L N **L** **P** **I** L D F D T R R Q
G8C1 294 GCGCTGCATTGAATCTCCCTATATTGGATTTCGATACAAGACGCCAA**TAA**AATATTGTAC
G14G1 358 GCGCTGCATTGAATCGCGCTAGA**T**--**GA**TT-------------CCAATAAAATATTGTAC
 S A A L N **R** **A** **R**

G8C1 354 TTATATAATAGCGGAAAAGATATTGAACGCGCCACTATGACGTCAAAAATTGAAGTTTTC
G14G1 403 TTATATAATAGCGGAAAAGATATTGAACGCGCCACTATGACGTCCAAAATTGAAGTTTTC

G8C1 414 TTACATTCTTTATTTTATAGCCGTCTTACAAATGCATTGATGTTGTATAATATCTTCCGC
G14G1 463 TTACATTCTTTATTTTATAGCCGTCTTACAAATGCATTGATGTTGTATAATATCTTCCGC

G8C1 474 TAAAATTGGTATACCCAACCGAAATATTCAATAAACACTCATAAATTTATCACTTACATT
G14G1 523 TAAAATTGGTATACCCAACCGAAATATTCAATAAACACTCATAAATTTATCACC------

**L**

M K L V L A I I

| G4C3 | - | - | C | A | A | T | A | A | A | A | T | A | A | T | T | G | T | T | C | A | A | C | G | A | T | T | G | A | A | A | A | C | G | A | A | A | **A** | **T** | **G** | A | A | G | T | T | A | G | T | T | T | T | G | G | C | G | A | T | A | A | T | T |
| --- | --- | --- | --- | --- | --- | --- | --- | --- | --- | --- | --- | --- | --- | --- | --- | --- | --- | --- | --- | --- | --- | --- | --- | --- | --- | --- | --- | --- | --- | --- | --- | --- | --- | --- | --- | --- | --- | --- | --- | --- | --- | --- | --- | --- | --- | --- | --- | --- | --- | --- | --- | --- | --- | --- | --- | --- | --- | --- | --- | --- |
| S22E4 | G | A | C | A | A | T | A | A | A | A | T | A | A | T | T | G | T | T | C | A | A | C | A | A | T | T | G | A | A | A | A | C | G | A | A | A | **A** | **T** | **G** | C | A | G | T | T | A | G | T | T | T | T | G | G | C | G | A | T | A | A | T | T |

***M Q L V L A I I***

***L S F G F Y L C A V S A*** T K P K K E L T

| G4C3 | T | T | G | T | C | C | T | T | T | G | G | T | T | T | T | T | A | T | T | T | A | T | G | T | G | C | A | G | T | C | A | G | T | G | C | G | A | C | G | A | A | A | C | C | A | A | A | A | A | A | A | G | A | A | C | T | T | A | C | C |
| --- | --- | --- | --- | --- | --- | --- | --- | --- | --- | --- | --- | --- | --- | --- | --- | --- | --- | --- | --- | --- | --- | --- | --- | --- | --- | --- | --- | --- | --- | --- | --- | --- | --- | --- | --- | --- | --- | --- | --- | --- | --- | --- | --- | --- | --- | --- | --- | --- | --- | --- | --- | --- | --- | --- | --- | --- | --- | --- | --- | --- |
| S22E4 | T | T | G | T | C | C | T | T | T | G | G | T | T | T | T | T | A | T | T | T | G | T | G | T | G | C | A | G | T | C | A | G | T | G | C | G | A | C | G | A | A | A | C | C | A | A | A | A | A | A | A | G | A | A | C | T | T | A | C | C |

***L S F G F Y L C A V S A*** T K P K K E L T

L E E K Y Q K F I S M **C** S L S D K R N D

| G4C3 | C | T | T | G | A | G | G | A | A | A | A | A | T | A | T | C | A | A | A | A | G | T | T | C | A | T | C | T | C | T | A | T | G | T | G | C | A | G | T | C | T | T | T | C | G | G | A | T | A | A | A | C | G | G | A | A | T | G | A | C |
| --- | --- | --- | --- | --- | --- | --- | --- | --- | --- | --- | --- | --- | --- | --- | --- | --- | --- | --- | --- | --- | --- | --- | --- | --- | --- | --- | --- | --- | --- | --- | --- | --- | --- | --- | --- | --- | --- | --- | --- | --- | --- | --- | --- | --- | --- | --- | --- | --- | --- | --- | --- | --- | --- | --- | --- | --- | --- | --- | --- | --- |
| S22E4 | C | T | T | G | A | G | G | A | A | A | A | A | T | A | T | C | A | A | A | A | G | T | T | C | A | T | C | T | C | T | A | T | G | C | G | C | A | G | T | C | T | T | T | C | G | G | A | T | A | A | A | C | G | G | A | A | T | G | A | C |

L E E K Y Q K F I S M **R** S L S D K R N D

K I Y F G S W T N L N D V G A D F K L S

| G4C3 | A | A | A | A | T | A | T | A | T | T | T | C | G | G | C | T | C | A | T | G | G | A | C | A | A | A | T | T | T | A | A | A | T | G | A | T | G | T | A | G | G | A | G | C | A | G | A | T | T | T | T | A | A | G | C | T | T | A | G | T |
| --- | --- | --- | --- | --- | --- | --- | --- | --- | --- | --- | --- | --- | --- | --- | --- | --- | --- | --- | --- | --- | --- | --- | --- | --- | --- | --- | --- | --- | --- | --- | --- | --- | --- | --- | --- | --- | --- | --- | --- | --- | --- | --- | --- | --- | --- | --- | --- | --- | --- | --- | --- | --- | --- | --- | --- | --- | --- | --- | --- | --- |
| S22E4 | A | A | A | A | T | A | T | A | T | T | T | C | G | G | C | T | C | A | T | G | G | A | C | A | A | A | T | T | T | A | A | A | T | G | A | T | G | T | A | G | G | A | G | C | A | G | A | T | T | T | T | A | A | G | C | T | T | A | G | T |

K I Y F G S W T N L N D V G A D F K L S

C **T** L E F **G** **T** K L K N S **I** D F N D **E** E **E**

| G4C3 | T | G | C | A | C | T | T | T | A | G | A | A | T | T | T | G | G | G | A | C | A | A | A | A | T | T | A | A | A | A | A | A | C | A | G | C | A | T | T | G | A | C | T | T | C | A | A | T | G | A | C | G | A | G | G | A | A | G | A | A |
| --- | --- | --- | --- | --- | --- | --- | --- | --- | --- | --- | --- | --- | --- | --- | --- | --- | --- | --- | --- | --- | --- | --- | --- | --- | --- | --- | --- | --- | --- | --- | --- | --- | --- | --- | --- | --- | --- | --- | --- | --- | --- | --- | --- | --- | --- | --- | --- | --- | --- | --- | --- | --- | --- | --- | --- | --- | --- | --- | --- | --- |
| S22E4 | T | G | C | A | T | T | T | T | A | G | A | A | T | T | T | G | G | A | A | T | A | A | A | A | T | T | A | A | A | A | A | A | C | A | G | C | A | T | G | G | A | C | T | T | C | A | A | T | G | A | C | A | A | G | G | A | A | A | A | A |

C **I** L E F **G** **I** K L K N S **M** D F N D **K** E **K**

**I** E W **N** C G **N** **T** G M C N A A Y D T L D E

| G4C3 | A | T | T | G | A | A | T | G | G | A | A | T | T | G | C | G | G | A | A | A | T | A | C | C | G | G | A | A | T | G | T | G | T | A | A | T | G | C | A | G | C | G | T | A | T | G | A | T | A | C | T | C | T | T | G | A | C | G | A | A |
| --- | --- | --- | --- | --- | --- | --- | --- | --- | --- | --- | --- | --- | --- | --- | --- | --- | --- | --- | --- | --- | --- | --- | --- | --- | --- | --- | --- | --- | --- | --- | --- | --- | --- | --- | --- | --- | --- | --- | --- | --- | --- | --- | --- | --- | --- | --- | --- | --- | --- | --- | --- | --- | --- | --- | --- | --- | --- | --- | --- | --- |
| S22E4 | A | A | T | G | A | A | T | G | G | G | A | T | T | G | C | G | G | A | A | A | A | A | T | C | G | G | A | A | T | G | T | G | T | A | A | T | G | C | A | G | C | G | T | A | T | G | A | T | A | C | T | C | T | T | G | A | C | G | A | A |

**N** E W **D** C G **K** **I** G M C N A A Y D T L D E

Q I K K F I D - - - - - - - - - - - - -

| G4C3 | C | A | A | A | T | A | A | A | A | A | A | A | T | T | T | A | T | C | G | A | T | G | - | - | - | - | - | - | - | - | - | - | - | - | - | - | - | - | - | - | - | - | - | - | - | - | - | - | - | - | - | - | - | - | - | - | - | - | - | - |
| --- | --- | --- | --- | --- | --- | --- | --- | --- | --- | --- | --- | --- | --- | --- | --- | --- | --- | --- | --- | --- | --- | --- | --- | --- | --- | --- | --- | --- | --- | --- | --- | --- | --- | --- | --- | --- | --- | --- | --- | --- | --- | --- | --- | --- | --- | --- | --- | --- | --- | --- | --- | --- | --- | --- | --- | --- | --- | --- | --- | --- |
| S22E4 | C | A | A | A | T | A | A | A | A | A | A | A | T | T | T | A | T | C | G | A | T | G | T | T | T | G | T | A | T | G | A | A | T | T | C | G | T | T | A | A | G | T | T | T | A | G | G | T | G | A | A | A | G | T | T | T | A | G | G | T |

Q I K K F I D V C M N S L S L G E S L G

- - - - - - - - - E G S K R Y D V L D F

| G4C3 | - | - | - | - | - | - | - | - | - | - | - | - | - | - | - | - | - | - | - | - | - | - | - | - | - | - | - | - | A | G | G | G | C | T | C | A | A | A | G | A | G | G | T | A | T | G | A | T | G | T | T | T | T | G | G | A | T | T | T | C |
| --- | --- | --- | --- | --- | --- | --- | --- | --- | --- | --- | --- | --- | --- | --- | --- | --- | --- | --- | --- | --- | --- | --- | --- | --- | --- | --- | --- | --- | --- | --- | --- | --- | --- | --- | --- | --- | --- | --- | --- | --- | --- | --- | --- | --- | --- | --- | --- | --- | --- | --- | --- | --- | --- | --- | --- | --- | --- | --- | --- | --- |
| S22E4 | G | A | A | A | G | T | T | T | T | G | T | T | T | T | A | A | T | T | G | A | A | A | T | G | A | A | G | G | A | G | G | G | C | T | C | A | A | A | G | A | G | G | T | A | T | G | A | T | G | T | T | T | T | G | G | A | T | T | T | C |

E S F V L I E M K E G S K R Y D V L D F

L S R W F T L R K L M K N N K E A S I K

| G4C3 | C | T | T | A | G | T | A | G | A | T | G | G | T | T | C | A | C | G | C | T | A | A | G | A | A | A | A | T | T | A | A | T | G | A | A | A | A | A | T | A | A | C | A | A | A | G | A | A | G | C | G | T | C | A | A | T | C | A | A | A |
| --- | --- | --- | --- | --- | --- | --- | --- | --- | --- | --- | --- | --- | --- | --- | --- | --- | --- | --- | --- | --- | --- | --- | --- | --- | --- | --- | --- | --- | --- | --- | --- | --- | --- | --- | --- | --- | --- | --- | --- | --- | --- | --- | --- | --- | --- | --- | --- | --- | --- | --- | --- | --- | --- | --- | --- | --- | --- | --- | --- | --- |
| S22E4 | C | T | T | A | G | T | A | G | A | T | G | G | T | T | C | A | C | G | C | T | A | A | G | A | A | A | A | T | T | A | A | T | G | A | A | A | A | A | T | A | A | C | A | A | A | G | A | A | G | C | G | T | C | A | A | T | C | A | A | A |

L S R W F T L R K L M K N N K E A S I K

S I T E **K** V L **I** D F A A E **K** T I K D H P

| G4C3 | A | G | T | A | T | A | A | C | C | G | A | G | A | A | A | G | T | T | T | T | G | A | T | A | G | A | T | T | T | C | G | C | T | G | C | T | G | A | A | A | A | A | A | C | G | A | T | T | A | A | A | G | A | T | C | A | T | C | C | G |
| --- | --- | --- | --- | --- | --- | --- | --- | --- | --- | --- | --- | --- | --- | --- | --- | --- | --- | --- | --- | --- | --- | --- | --- | --- | --- | --- | --- | --- | --- | --- | --- | --- | --- | --- | --- | --- | --- | --- | --- | --- | --- | --- | --- | --- | --- | --- | --- | --- | --- | --- | --- | --- | --- | --- | --- | --- | --- | --- | --- | --- |
| S22E4 | A | G | T | A | T | A | A | C | C | G | A | G | G | A | A | G | T | T | T | T | G | A | C | A | G | A | T | T | T | C | G | C | T | G | C | T | G | A | A | G | A | A | A | C | G | A | T | T | A | A | A | G | A | T | C | A | T | C | C | G |

S I T E **E** V L **T** D F A A E **E** T I K D H P

T E N E S N L D **K** E K P A N L N

| G4C3 | A | C | T | G | A | A | A | A | C | G | A | A | T | C | T | A | A | T | T | T | G | G | A | C | A | A | G | G | A | A | A | A | A | C | C | G | G | C | C | A | A | T | T | T | A | A | A | C | **T** | **A** | **A** | A | A | A | T | A | A | A | T | G |
| --- | --- | --- | --- | --- | --- | --- | --- | --- | --- | --- | --- | --- | --- | --- | --- | --- | --- | --- | --- | --- | --- | --- | --- | --- | --- | --- | --- | --- | --- | --- | --- | --- | --- | --- | --- | --- | --- | --- | --- | --- | --- | --- | --- | --- | --- | --- | --- | --- | --- | --- | --- | --- | --- | --- | --- | --- | --- | --- | --- | --- |
| S22E4 | A | C | T | G | A | A | A | A | C | G | A | A | T | C | T | A | A | T | T | T | G | G | A | C | A | T | G | G | A | A | A | A | A | C | C | G | G | C | C | A | A | T | T | T | A | A | A | C | **T** | **G** | **A** | A | A | A | T | A | A | A | T | G |

T E N E S N L D **M** E K P A N L N

| G4C3 | A | A | T | C | T | A | A | T | G | A | T | T | T | T | A | A | A | A | C | A | A | T | A | G | T | G | A | A | A | A | T | G | T | A | A | T | T | T | G | A | A | A | G | T | A | A | A | T | T | T | T | T | G | A | G | A | A | T | A | A |
| --- | --- | --- | --- | --- | --- | --- | --- | --- | --- | --- | --- | --- | --- | --- | --- | --- | --- | --- | --- | --- | --- | --- | --- | --- | --- | --- | --- | --- | --- | --- | --- | --- | --- | --- | --- | --- | --- | --- | --- | --- | --- | --- | --- | --- | --- | --- | --- | --- | --- | --- | --- | --- | --- | --- | --- | --- | --- | --- | --- | --- |
| S22E4 | A | A | T | C | T | A | A | T | G | A | T | T | T | T | A | A | A | A | C | A | A | T | A | T | T | G | A | A | A | A | T | G | T | A | A | T | T | T | G | A | A | A | G | T | A | A | A | T | T | T | T | T | G | A | G | A | A | T | A | A |

| G4C3 | A | A | T | A | T | T | A | T | A |  |  |  |  |  |  |  |  |  |  |  |  |  |  |  |  |  |  |  |  |  |  |  |  |  |  |  |  |  |  |  |  |  |  |  |  |  |  |  |  |  |  |  |  |  |  |  |  |  |  |  |
| --- | --- | --- | --- | --- | --- | --- | --- | --- | --- | --- | --- | --- | --- | --- | --- | --- | --- | --- | --- | --- | --- | --- | --- | --- | --- | --- | --- | --- | --- | --- | --- | --- | --- | --- | --- | --- | --- | --- | --- | --- | --- | --- | --- | --- | --- | --- | --- | --- | --- | --- | --- | --- | --- | --- | --- | --- | --- | --- | --- | --- |
| S22E4 | G | A | T | A | T | T | A | G | A |  |  |  |  |  |  |  |  |  |  |  |  |  |  |  |  |  |  |  |  |  |  |  |  |  |  |  |  |  |  |  |  |  |  |  |  |  |  |  |  |  |  |  |  |  |  |  |  |  |  |  |

**M**

***M K V***

| G7D10 | A | T | C | A | G | T | T | A | T | T | C | A | A | T | C | C | A | T | T | T | G | A | A | A | C | G | A | G | T | A | A | C | A | G | T | T | T | A | T | T | A | T | T | T | G | A | G | A | A | A | A | A | **A** | **T** | **G** | A | A | A | G | T |
| --- | --- | --- | --- | --- | --- | --- | --- | --- | --- | --- | --- | --- | --- | --- | --- | --- | --- | --- | --- | --- | --- | --- | --- | --- | --- | --- | --- | --- | --- | --- | --- | --- | --- | --- | --- | --- | --- | --- | --- | --- | --- | --- | --- | --- | --- | --- | --- | --- | --- | --- | --- | --- | --- | --- | --- | --- | --- | --- | --- | --- |
| Sg4A9 | - | - | - | - | - | - | - | - | - | - | - | - | - | - | - | - | - | - | - | - | G | A | A | A | C | G | A | G | T | A | A | C | A | G | T | T | T | A | A | T | A | T | T | T | G | A | G | A | A | A | A | A | **A** | **T** | **G** | A | A | A | G | T |

***M K V***

***I I L A L F A I V A V A C V S G*** **Q** G **A** G

| G7D10 | C | A | T | C | A | T | T | T | T | A | G | C | T | T | T | G | T | T | C | G | C | A | A | T | C | G | T | T | G | C | T | G | T | A | G | C | C | T | G | T | G | T | T | T | C | A | G | G | T | C | A | A | G | G | A | G | C | A | G | G |
| --- | --- | --- | --- | --- | --- | --- | --- | --- | --- | --- | --- | --- | --- | --- | --- | --- | --- | --- | --- | --- | --- | --- | --- | --- | --- | --- | --- | --- | --- | --- | --- | --- | --- | --- | --- | --- | --- | --- | --- | --- | --- | --- | --- | --- | --- | --- | --- | --- | --- | --- | --- | --- | --- | --- | --- | --- | --- | --- | --- | --- |
| Sg4A9 | C | A | T | C | A | T | T | T | T | A | G | C | T | T | T | G | T | T | C | G | C | A | A | T | C | G | T | T | G | C | T | G | T | A | G | C | C | T | G | T | G | T | A | T | C | A | G | G | T | G | A | A | G | G | A | G | T | A | G | G |

***I I L A L F A I V A V A*** C V S G **E** G **V** G

L P Q D P L K T I S E A G K S A T D A A

| G7D10 | A | C | T | C | C | C | A | C | A | G | G | A | T | C | C | T | C | T | T | A | A | A | A | C | A | A | T | A | T | C | T | G | A | A | G | C | T | G | G | T | A | A | A | T | C | A | G | C | A | A | C | T | G | A | T | G | C | A | G | C |
| --- | --- | --- | --- | --- | --- | --- | --- | --- | --- | --- | --- | --- | --- | --- | --- | --- | --- | --- | --- | --- | --- | --- | --- | --- | --- | --- | --- | --- | --- | --- | --- | --- | --- | --- | --- | --- | --- | --- | --- | --- | --- | --- | --- | --- | --- | --- | --- | --- | --- | --- | --- | --- | --- | --- | --- | --- | --- | --- | --- | --- |
| Sg4A9 | A | C | T | C | C | C | A | C | A | G | G | A | T | C | C | T | C | T | T | A | A | A | A | C | A | A | T | A | T | C | T | G | A | A | G | C | T | G | G | T | A | A | A | T | C | A | G | C | A | A | C | T | G | A | T | G | C | A | G | C |

L P Q D P L K T I S E A G K S A T D A A

N G G V K S A T **E** **A** V K P A T D A A T -

| G7D10 | A | A | A | T | G | G | A | G | G | T | G | T | T | A | A | A | T | C | C | G | C | A | A | C | T | G | A | A | G | C | A | G | T | T | A | A | A | C | C | A | G | C | A | A | C | T | G | A | T | G | C | A | G | C | A | A | C | T | G | - |
| --- | --- | --- | --- | --- | --- | --- | --- | --- | --- | --- | --- | --- | --- | --- | --- | --- | --- | --- | --- | --- | --- | --- | --- | --- | --- | --- | --- | --- | --- | --- | --- | --- | --- | --- | --- | --- | --- | --- | --- | --- | --- | --- | --- | --- | --- | --- | --- | --- | --- | --- | --- | --- | --- | --- | --- | --- | --- | --- | --- | --- |
| Sg4A9 | A | A | A | T | G | G | A | G | G | T | G | T | T | A | A | A | T | C | C | G | C | A | A | C | T | G | A | T | G | C | G | G | T | T | A | A | A | C | C | A | G | C | A | A | C | T | G | A | T | G | C | A | G | C | A | A | C | T | G | G |

N G G V K S A T **D** **A** V K P A T D A A T G

- - - - - - E A V K P A T **E** A - - - - -

| G7D10 | - | - | - | - | - | - | - | - | - | - | - | - | - | - | - | - | - | - | - | - | A | A | G | C | A | G | T | T | A | A | A | C | C | A | G | C | A | A | C | T | G | A | A | G | C | A | G | - | - | - | - | - | - | - | - | - | - | - | - | - |
| --- | --- | --- | --- | --- | --- | --- | --- | --- | --- | --- | --- | --- | --- | --- | --- | --- | --- | --- | --- | --- | --- | --- | --- | --- | --- | --- | --- | --- | --- | --- | --- | --- | --- | --- | --- | --- | --- | --- | --- | --- | --- | --- | --- | --- | --- | --- | --- | --- | --- | --- | --- | --- | --- | --- | --- | --- | --- | --- | --- | --- |
| Sg4A9 | A | G | G | T | C | T | T | A | A | T | T | C | A | G | C | A | A | C | T | G | A | A | G | C | A | G | T | T | A | A | A | C | C | A | G | C | A | A | C | T | G | A | T | G | C | A | G | C | G | A | C | T | G | A | A | G | G | T | C | T |

G L N S A T E A V K P A T **D** A A T E G L

- - - - - - - - - - V **K** P A T D A L T G

| G7D10 | - | - | - | - | - | - | - | - | - | - | - | - | - | - | - | - | - | - | - | - | - | - | - | - | - | - | - | - | - | - | - | - | T | T | A | A | A | C | C | A | G | C | A | A | C | T | G | A | T | G | C | A | C | T | A | A | C | T | G | G |
| --- | --- | --- | --- | --- | --- | --- | --- | --- | --- | --- | --- | --- | --- | --- | --- | --- | --- | --- | --- | --- | --- | --- | --- | --- | --- | --- | --- | --- | --- | --- | --- | --- | --- | --- | --- | --- | --- | --- | --- | --- | --- | --- | --- | --- | --- | --- | --- | --- | --- | --- | --- | --- | --- | --- | --- | --- | --- | --- | --- | --- |
| Sg4A9 | T | A | A | T | T | C | A | G | C | A | A | C | T | G | G | A | A | C | A | G | T | A | A | A | T | G | G | A | G | C | T | G | T | T | C | A | A | C | C | A | G | C | A | A | C | T | G | A | T | G | C | A | G | C | A | A | C | T | G | G |

N S A T G T V N G A V **Q** P A T D A A T G

G **V** K S A T D A V K P A T D A - - - - V

| G7D10 | A | G | G | T | G | T | T | A | A | A | T | C | C | G | C | A | A | C | T | G | A | T | G | C | A | G | T | T | A | A | A | C | C | A | G | C | A | A | C | T | G | A | T | G | C | A | G | - | - | - | - | - | - | - | - | - | - | - | - | T |
| --- | --- | --- | --- | --- | --- | --- | --- | --- | --- | --- | --- | --- | --- | --- | --- | --- | --- | --- | --- | --- | --- | --- | --- | --- | --- | --- | --- | --- | --- | --- | --- | --- | --- | --- | --- | --- | --- | --- | --- | --- | --- | --- | --- | --- | --- | --- | --- | --- | --- | --- | --- | --- | --- | --- | --- | --- | --- | --- | --- | --- |
| Sg4A9 | A | G | G | T | C | T | T | A | A | A | T | C | C | G | C | A | A | C | T | G | A | T | G | C | A | G | T | T | A | A | A | C | C | A | G | C | A | A | C | T | G | A | T | G | C | A | G | C | A | G | C | T | G | G | A | G | G | T | C | T |

G **L** K S A T D A V K P A T D A A A G G L

K **P** A T D **A** L T **G** A A Q P L T A A L P Q

| G7D10 | T | A | A | A | C | C | A | G | C | A | A | C | T | G | A | T | G | C | A | C | T | A | A | C | T | G | G | A | G | C | T | G | C | T | C | A | A | C | C | A | C | T | A | A | C | T | G | C | A | G | C | T | C | T | T | C | C | A | C | A |
| --- | --- | --- | --- | --- | --- | --- | --- | --- | --- | --- | --- | --- | --- | --- | --- | --- | --- | --- | --- | --- | --- | --- | --- | --- | --- | --- | --- | --- | --- | --- | --- | --- | --- | --- | --- | --- | --- | --- | --- | --- | --- | --- | --- | --- | --- | --- | --- | --- | --- | --- | --- | --- | --- | --- | --- | --- | --- | --- | --- | --- |
| Sg4A9 | T | A | A | A | T | C | A | G | C | A | A | C | T | G | G | A | C | C | A | C | T | A | A | C | T | G | C | A | G | C | T | G | C | T | C | A | A | C | C | A | C | T | A | A | C | T | G | C | A | G | C | T | C | T | T | C | C | A | C | A |

K **S** A T G **P** L T **A** A A Q P L T A A L P Q

S **Q**

| G7D10 | A | A | G | C | C | A | A | **T** | **A** | **A** | A | C | A | C | C | A | G | C | A | T | C | T | A | C | T | G | A | C | C | T | T | G | C | A | T | A | A | A | A | A | C | A | A | T | T | C | G | A | T | T | T | C | A | A | T | T | C | A | A | A |
| --- | --- | --- | --- | --- | --- | --- | --- | --- | --- | --- | --- | --- | --- | --- | --- | --- | --- | --- | --- | --- | --- | --- | --- | --- | --- | --- | --- | --- | --- | --- | --- | --- | --- | --- | --- | --- | --- | --- | --- | --- | --- | --- | --- | --- | --- | --- | --- | --- | --- | --- | --- | --- | --- | --- | --- | --- | --- | --- | --- | --- |
| Sg4A9 | A | A | G | C | C | T | A | **T** | **A** | **A** | A | C | A | C | C | A | G | C | A | T | C | T | A | C | T | G | A | C | C | T | T | G | C | A | T | A | A | A | A | A | C | A | A | T | T | C | G | A | T | T | T | C | A | A | T | T | C | A | A | A |

S **L**

| G7D10 | A | G | T | A | A | A | A | A | T | T | T | C | A | T | G | G | A | A | A | T | C | A | T | T | G | A | A | C | T | A | A | A | C | T | T | T | C | A | A | T | A | A | A | A | T | A | A | A | T | T | T | G | A | A | A | G | A | C | G | A |
| --- | --- | --- | --- | --- | --- | --- | --- | --- | --- | --- | --- | --- | --- | --- | --- | --- | --- | --- | --- | --- | --- | --- | --- | --- | --- | --- | --- | --- | --- | --- | --- | --- | --- | --- | --- | --- | --- | --- | --- | --- | --- | --- | --- | --- | --- | --- | --- | --- | --- | --- | --- | --- | --- | --- | --- | --- | --- | --- | --- | --- |
| Sg4A9 | A | G | T | A | A | A | A | A | T | T | T | C | A | T | G | G | A | A | A | T | C | A | T | T | G | A | A | C | T | A | A | A | C | T | T | T | C | A | A | T | A | A | A | A | T | A | A | A | T | T | T | G | G | A | A | G | A | C | G | A |

| G7D10 | A | A | A | T | G | T | C | T | T | T | G | C |  |  |  |  |  |  |  |  |  |  |  |  |  |  |  |  |  |  |  |  |  |  |  |  |  |  |  |  |  |  |  |  |  |  |  |  |  |  |  |  |  |  |  |  |  |  |  |  |
| --- | --- | --- | --- | --- | --- | --- | --- | --- | --- | --- | --- | --- | --- | --- | --- | --- | --- | --- | --- | --- | --- | --- | --- | --- | --- | --- | --- | --- | --- | --- | --- | --- | --- | --- | --- | --- | --- | --- | --- | --- | --- | --- | --- | --- | --- | --- | --- | --- | --- | --- | --- | --- | --- | --- | --- | --- | --- | --- | --- | --- |
| Sg4A9 | A | A | A | T | G | T | C | T | T | T | G | C |  |  |  |  |  |  |  |  |  |  |  |  |  |  |  |  |  |  |  |  |  |  |  |  |  |  |  |  |  |  |  |  |  |  |  |  |  |  |  |  |  |  |  |  |  |  |  |  |

**N**

M K Y L F

| S5E9 | - | A | T | A | G | T | C | A | G | C | T | T | A | A | A | G | C | A | T | T | T | C | G | A | C | T | G | A | A | G | A | C | A | C | T | A | A | A | C | T | T | T | C | A | A | A | **A** | **T** | **G** | A | A | A | T | A | T | T | T | G | T | T |
| --- | --- | --- | --- | --- | --- | --- | --- | --- | --- | --- | --- | --- | --- | --- | --- | --- | --- | --- | --- | --- | --- | --- | --- | --- | --- | --- | --- | --- | --- | --- | --- | --- | --- | --- | --- | --- | --- | --- | --- | --- | --- | --- | --- | --- | --- | --- | --- | --- | --- | --- | --- | --- | --- | --- | --- | --- | --- | --- | --- | --- |
| G9G9 | A | A | T | A | G | T | C | A | G | C | T | T | A | A | A | G | C | A | T | T | T | C | G | A | C | T | G | A | A | G | A | C | A | C | T | A | A | A | C | T | T | T | C | A | A | A | **A** | **T** | **G** | A | A | A | T | A | T | T | T | G | T | T |

M K Y L F

***V F F V I I A*** ***S L L G L S*** H G E I C A **I**

| S5E9 | C | G | T | A | T | T | T | T | T | C | G | T | T | A | T | T | A | C | T | G | C | A | A | G | T | C | T | T | T | T | G | G | G | A | C | T | T | T | C | T | C | A | C | G | G | A | A | C | A | A | T | C | T | G | T | G | C | C | A | G |
| --- | --- | --- | --- | --- | --- | --- | --- | --- | --- | --- | --- | --- | --- | --- | --- | --- | --- | --- | --- | --- | --- | --- | --- | --- | --- | --- | --- | --- | --- | --- | --- | --- | --- | --- | --- | --- | --- | --- | --- | --- | --- | --- | --- | --- | --- | --- | --- | --- | --- | --- | --- | --- | --- | --- | --- | --- | --- | --- | --- | --- |
| G9G9 | C | G | T | A | T | T | T | T | T | C | G | T | T | A | T | T | A | T | T | G | C | A | A | G | T | C | T | T | T | T | G | G | G | A | C | T | T | T | C | T | C | A | C | G | G | A | G | A | A | A | T | C | T | G | T | G | C | C | A | T |

V F F V I T A S L L G L S H G T I C A **R**

D H K S G G **I** Q N F P N I F A M M A E N

| S5E9 | A | G | A | T | G | G | A | C | G | A | A | G | T | G | G | T | G | G | A | A | A | A | C | A | A | A | A | T | T | T | T | C | C | T | A | A | T | A | T | A | T | T | T | G | C | A | A | T | G | A | T | G | G | C | T | G | A | A | A | A |
| --- | --- | --- | --- | --- | --- | --- | --- | --- | --- | --- | --- | --- | --- | --- | --- | --- | --- | --- | --- | --- | --- | --- | --- | --- | --- | --- | --- | --- | --- | --- | --- | --- | --- | --- | --- | --- | --- | --- | --- | --- | --- | --- | --- | --- | --- | --- | --- | --- | --- | --- | --- | --- | --- | --- | --- | --- | --- | --- | --- | --- |
| G9G9 | A | G | A | T | C | A | C | A | A | A | A | G | T | G | G | T | G | G | A | A | T | A | C | A | A | A | A | T | T | T | T | C | C | T | A | A | T | A | T | A | T | T | T | G | C | A | A | T | G | A | T | G | G | C | T | G | A | A | A | A |

D G R S G G **K** Q N F P N I F A M M A E N

G R G G R Y S **F** E **R** N G A C

| S5E9 | T | G | G | G | C | G | T | G | G | A | G | G | C | C | G | T | T | A | C | T | C | T | T | T | G | G | A | A | A | A | G | A | A | T | G | G | A | G | C | A | T | G | T | **T** | **A** | **A** | C | A | A | C | A | A | A | T | G | C | C | A | A | A |
| --- | --- | --- | --- | --- | --- | --- | --- | --- | --- | --- | --- | --- | --- | --- | --- | --- | --- | --- | --- | --- | --- | --- | --- | --- | --- | --- | --- | --- | --- | --- | --- | --- | --- | --- | --- | --- | --- | --- | --- | --- | --- | --- | --- | --- | --- | --- | --- | --- | --- | --- | --- | --- | --- | --- | --- | --- | --- | --- | --- | --- |
| G9G9 | T | G | G | G | C | G | T | G | G | A | G | G | C | C | G | T | T | A | C | T | C | T | T | T | C | G | A | A | A | G | G | A | A | T | G | G | A | G | C | A | T | G | T | **T** | **A** | **A** | C | A | A | C | A | A | T | T | G | C | C | A | A | A |

G R G G R Y S **L** E **K** N G A C

| S5E9 | A | G | A | A | G | A | C | T | T | C | G | A | G | C | A | C | T | A | T | G | G | A | C | T | A | T | G | G | A | A | A | C | C | G | G | T | T | G | C | T | T | A | G | T | C | A | T | A | A | T | T | C | C | G | T | A | T | T | A | T |
| --- | --- | --- | --- | --- | --- | --- | --- | --- | --- | --- | --- | --- | --- | --- | --- | --- | --- | --- | --- | --- | --- | --- | --- | --- | --- | --- | --- | --- | --- | --- | --- | --- | --- | --- | --- | --- | --- | --- | --- | --- | --- | --- | --- | --- | --- | --- | --- | --- | --- | --- | --- | --- | --- | --- | --- | --- | --- | --- | --- | --- |
| G9G9 | A | G | A | A | G | A | C | T | T | C | G | A | G | C | A | C | C | G | T | G | G | A | C | T | A | T | G | G | A | A | A | C | C | C | G | T | T | G | C | T | A | A | G | T | C | A | T | A | A | T | T | C | T | G | T | A | T | T | G | T |

| S5E9 | A | T | A | A | T | G | A | T | T | T | T | G | C | G | A | A | T | G | T | G | C | G | C | C | A | T | T | A | A | A | T | A | A | A | T | A | C | A | T | A | T | C | C | G | C | C | - |  |  |  |  |  |  |  |  |  |  |  |  |  |
| --- | --- | --- | --- | --- | --- | --- | --- | --- | --- | --- | --- | --- | --- | --- | --- | --- | --- | --- | --- | --- | --- | --- | --- | --- | --- | --- | --- | --- | --- | --- | --- | --- | --- | --- | --- | --- | --- | --- | --- | --- | --- | --- | --- | --- | --- | --- | --- | --- | --- | --- | --- | --- | --- | --- | --- | --- | --- | --- | --- | --- |
| G9G9 | A | T | G | A | T | G | A | T | T | T | T | G | C | G | A | A | T | G | T | G | C | A | A | C | T | T | T | A | A | A | T | A | A | A | T | A | C | A | T | A | T | A | C | G | C | C | G |  |  |  |  |  |  |  |  |  |  |  |  |  |

**O**

| S14G9 | G | G | A | T | A | T | C | A | G | T | T | T | G | A | T | T | C | G | A | G | T | T | G | T | T | G | A | G | A | A | A | T | C | A | C | C | A | A | G | T | T | A | A | A | G | A | C | T | A | A | A | A | A | C | A | A | A | A | A | A |
| --- | --- | --- | --- | --- | --- | --- | --- | --- | --- | --- | --- | --- | --- | --- | --- | --- | --- | --- | --- | --- | --- | --- | --- | --- | --- | --- | --- | --- | --- | --- | --- | --- | --- | --- | --- | --- | --- | --- | --- | --- | --- | --- | --- | --- | --- | --- | --- | --- | --- | --- | --- | --- | --- | --- | --- | --- | --- | --- | --- | --- |
| S22D12 | - | - | - | T | A | T | C | A | G | T | T | T | G | A | T | T | C | G | A | G | T | C | G | T | T | G | A | G | A | A | A | T | T | A | C | C | A | A | G | T | T | A | A | A | G | A | C | T | T | A | A | A | A | C | A | A | A | A | A | A |

***M***  ***K F F A V L L L C A V A I A C***

| S14G9 | C | A | T | T | T | T | T | T | T | C | A | A | A | **A** | **T** | **G** | A | A | A | T | T | C | T | T | T | G | C | C | G | T | T | T | T | A | T | T | G | T | T | G | T | G | C | G | C | C | G | T | T | G | C | C | A | T | C | G | C | C | T | G |
| --- | --- | --- | --- | --- | --- | --- | --- | --- | --- | --- | --- | --- | --- | --- | --- | --- | --- | --- | --- | --- | --- | --- | --- | --- | --- | --- | --- | --- | --- | --- | --- | --- | --- | --- | --- | --- | --- | --- | --- | --- | --- | --- | --- | --- | --- | --- | --- | --- | --- | --- | --- | --- | --- | --- | --- | --- | --- | --- | --- | --- |
| S22D12 | C | A | T | T | T | T | T | T | A | - | A | A | A | **A** | **T** | **G** | A | A | A | T | T | C | T | T | C | G | C | C | G | T | T | T | T | A | T | T | G | T | T | G | T | G | C | G | C | C | G | T | T | G | T | C | A | T | C | G | C | C | T | G |

***M K F F A V L L L C A V V I A C***

***V S A*** Q **G** S S P V N **A** G - - - - - - - -

| S14G9 | T | G | T | C | A | G | C | G | C | T | C | A | A | G | G | C | A | G | C | T | C | C | C | C | A | G | T | G | A | A | T | G | C | A | G | G | G | A | - | - | - | - | - | - | - | - | - | - | - | - | - | - | - | - | - | - | - | - | - | - |
| --- | --- | --- | --- | --- | --- | --- | --- | --- | --- | --- | --- | --- | --- | --- | --- | --- | --- | --- | --- | --- | --- | --- | --- | --- | --- | --- | --- | --- | --- | --- | --- | --- | --- | --- | --- | --- | --- | --- | --- | --- | --- | --- | --- | --- | --- | --- | --- | --- | --- | --- | --- | --- | --- | --- | --- | --- | --- | --- | --- | --- |
| S22D12 | T | G | T | C | A | G | C | G | C | T | C | A | A | G | G | T | A | G | C | T | C | C | C | C | A | G | T | G | A | A | T | G | G | A | G | G | G | A | A | T | G | G | C | G | C | C | C | A | A | G | A | C | C | C | A | T | T | G | A | C |

***V S A***  Q **G** S S P V N **G** G N G A Q D P L T

- - S S **M** **L** T **S** I M T Q L **E** **K** L W E Q **F**

| S14G9 | - | - | - | - | - | - | - | - | G | T | T | C | A | A | T | G | C | T | G | A | C | T | A | G | T | A | T | A | A | T | G | A | C | T | C | A | A | T | T | G | G | A | A | A | A | A | C | T | C | T | G | G | G | A | A | C | A | A | T | T |
| --- | --- | --- | --- | --- | --- | --- | --- | --- | --- | --- | --- | --- | --- | --- | --- | --- | --- | --- | --- | --- | --- | --- | --- | --- | --- | --- | --- | --- | --- | --- | --- | --- | --- | --- | --- | --- | --- | --- | --- | --- | --- | --- | --- | --- | --- | --- | --- | --- | --- | --- | --- | --- | --- | --- | --- | --- | --- | --- | --- | --- |
| S22D12 | T | G | C | A | G | C | G | A | G | T | T | C | A | G | T | G | G | T | G | A | C | T | G | G | T | A | T | A | A | T | G | A | C | T | C | A | A | T | T | G | C | A | A | C | A | A | C | T | C | A | T | T | G | C | A | C | A | A | T | T |

A A S S **V** **V** T **G** I M T Q L **Q** **Q** L I A Q **L**

**L** A A **V** Q K L F G S F D L S **S** L **T** **S** P A

| S14G9 | C | T | T | G | G | C | T | G | C | T | G | T | G | C | A | A | A | A | A | T | T | G | T | T | T | G | G | A | T | C | A | T | T | C | G | A | T | T | T | G | A | G | C | T | C | G | T | T | A | A | C | C | A | G | T | C | C | A | G | C |
| --- | --- | --- | --- | --- | --- | --- | --- | --- | --- | --- | --- | --- | --- | --- | --- | --- | --- | --- | --- | --- | --- | --- | --- | --- | --- | --- | --- | --- | --- | --- | --- | --- | --- | --- | --- | --- | --- | --- | --- | --- | --- | --- | --- | --- | --- | --- | --- | --- | --- | --- | --- | --- | --- | --- | --- | --- | --- | --- | --- | --- |
| S22D12 | A | A | T | G | G | C | T | G | C | T | T | T | G | C | A | A | A | A | A | T | T | G | G | T | G | G | G | A | C | A | A | A | T | G | A | G | T | T | T | G | A | G | C | G | C | G | T | T | A | A | A | C | A | T | T | C | C | A | G | C |

**M** A A **L** Q K L V G Q M S L S **A** L **N** **I** P A

S S L A S S **V** G S T L G G V G S S L T G

| S14G9 | T | T | C | A | T | C | G | C | T | T | G | C | T | T | C | C | A | G | T | G | T | T | G | G | A | T | C | T | A | C | T | T | T | G | G | G | T | G | G | T | G | T | G | G | G | A | T | C | C | A | G | T | T | T | G | A | C | T | G | G |
| --- | --- | --- | --- | --- | --- | --- | --- | --- | --- | --- | --- | --- | --- | --- | --- | --- | --- | --- | --- | --- | --- | --- | --- | --- | --- | --- | --- | --- | --- | --- | --- | --- | --- | --- | --- | --- | --- | --- | --- | --- | --- | --- | --- | --- | --- | --- | --- | --- | --- | --- | --- | --- | --- | --- | --- | --- | --- | --- | --- | --- |
| S22D12 | T | T | C | A | - | - | - | C | T | T | G | C | T | T | C | C | A | G | T | A | T | T | G | G | A | T | C | T | A | C | T | T | T | G | G | G | T | G | G | T | G | T | G | G | G | A | T | C | C | A | G | T | T | T | G | A | C | T | G | G |

S - L A S S **I** G S T L G G V G S S L T G

A V P K T A A

| S14G9 | A | G | C | T | G | T | A | C | C | A | A | A | A | A | C | C | G | C | A | G | C | T | **T** | **A** | **A** | A | C | A | T | T | C | A | A | A | A | C | T | C | T | G | G | A | A | C | A | A | C | A | G | A | A | A | A | T | C | T | G | A | G | C |
| --- | --- | --- | --- | --- | --- | --- | --- | --- | --- | --- | --- | --- | --- | --- | --- | --- | --- | --- | --- | --- | --- | --- | --- | --- | --- | --- | --- | --- | --- | --- | --- | --- | --- | --- | --- | --- | --- | --- | --- | --- | --- | --- | --- | --- | --- | --- | --- | --- | --- | --- | --- | --- | --- | --- | --- | --- | --- | --- | --- | --- |
| S22D12 | A | G | C | T | G | T | A | C | C | A | A | A | A | A | C | C | G | C | A | G | C | T | **T** | **A** | **A** | A | C | A | T | T | C | A | A | A | A | C | T | C | T | G | G | A | A | C | A | A | C | A | G | A | A | A | A | T | C | T | G | A | G | C |

A V P K T A A

| S14G9 | A | C | A | A | A | C | C | A | T | A | T | T | T | G | A | T | C | G | C | C | T | A | A | A | A | T | T | G | T | C | A | A | T | T | T | T | C | A | T | T | G | T | A | T | T | T | A | T | T | T | G | A | A | T | G | T | A | T | T | G |
| --- | --- | --- | --- | --- | --- | --- | --- | --- | --- | --- | --- | --- | --- | --- | --- | --- | --- | --- | --- | --- | --- | --- | --- | --- | --- | --- | --- | --- | --- | --- | --- | --- | --- | --- | --- | --- | --- | --- | --- | --- | --- | --- | --- | --- | --- | --- | --- | --- | --- | --- | --- | --- | --- | --- | --- | --- | --- | --- | --- | --- |
| S22D12 | A | C | A | A | A | C | C | A | T | A | T | T | T | G | A | T | C | G | C | C | A | A | A | A | A | T | T | G | T | C | A | A | T | T | T | C | C | A | T | T | G | A | A | T | T | T | A | T | C | T | G | A | A | T | G | C | A | T | T | G |

| S14G9 | T | G | T | T | G | G | A | C | T | A | T | G | A | A | G | T | A | A | A | G | A | A | A | A | T | A | T | C | T | T | A | T | T | C | C |  |  |  |  |  |  |  |  |  |  |  |  |  |  |  |  |  |  |  |  |  |  |  |  |  |
| --- | --- | --- | --- | --- | --- | --- | --- | --- | --- | --- | --- | --- | --- | --- | --- | --- | --- | --- | --- | --- | --- | --- | --- | --- | --- | --- | --- | --- | --- | --- | --- | --- | --- | --- | --- | --- | --- | --- | --- | --- | --- | --- | --- | --- | --- | --- | --- | --- | --- | --- | --- | --- | --- | --- | --- | --- | --- | --- | --- | --- |
| S22D12 | T | G | A | T | G | G | A | T | T | A | T | A | A | A | A | T | A | A | A | G | A | A | A | A | T | A | T | C | T | T | A | T | T | C | C |  |  |  |  |  |  |  |  |  |  |  |  |  |  |  |  |  |  |  |  |  |  |  |  |  |

**P**

M K L P I L F L

| L7h8 | - | - | - | - | - | - | - | - | - | - | A | A | A | C | G | A | T | A | C | A | G | T | A | A | T | C | C | A | A | A | C | G | A | G | A | A | G | **A** | **T** | **G** | A | A | G | C | T | T | C | C | A | A | T | C | C | T | C | T | T | T | C | T |
| --- | --- | --- | --- | --- | --- | --- | --- | --- | --- | --- | --- | --- | --- | --- | --- | --- | --- | --- | --- | --- | --- | --- | --- | --- | --- | --- | --- | --- | --- | --- | --- | --- | --- | --- | --- | --- | --- | --- | --- | --- | --- | --- | --- | --- | --- | --- | --- | --- | --- | --- | --- | --- | --- | --- | --- | --- | --- | --- | --- | --- |
| S8C9 | G | G | C | A | C | A | G | T | C | A | A | A | A | C | G | A | T | A | C | A | G | C | A | A | T | C | C | A | A | A | C | G | A | G | A | A | G | **A** | **T** | **G** | A | A | G | C | T | T | C | C | A | A | T | C | C | T | T | T | T | T | C | T |

***M K L P I L F L***

***L I L N L L A V F G*** E V R K V **N** Q K T **I**

| L7h8 | C | C | T | A | A | T | A | T | T | A | A | A | T | T | T | G | C | T | G | G | C | T | G | T | A | T | T | T | G | G | T | G | A | G | G | T | T | C | G | A | A | A | A | G | T | C | A | A | T | C | A | A | A | A | A | A | C | T | A | T |
| --- | --- | --- | --- | --- | --- | --- | --- | --- | --- | --- | --- | --- | --- | --- | --- | --- | --- | --- | --- | --- | --- | --- | --- | --- | --- | --- | --- | --- | --- | --- | --- | --- | --- | --- | --- | --- | --- | --- | --- | --- | --- | --- | --- | --- | --- | --- | --- | --- | --- | --- | --- | --- | --- | --- | --- | --- | --- | --- | --- | --- |
| S8C9 | G | C | T | A | A | T | A | T | T | A | A | A | T | T | T | G | C | T | G | G | G | C | G | T | A | T | T | T | G | G | T | G | A | G | G | T | T | C | G | A | A | A | A | G | T | C | G | A | T | C | A | A | A | A | A | A | C | T | C | T |

***L I L N L L G V F G*** E V R K V **D** Q K T **L**

D Y I K K R **G** K W M P **K** A Y D D G Y G **N**

| L7h8 | T | G | A | T | T | A | C | A | T | C | A | A | G | A | A | A | C | G | T | G | G | G | A | A | A | T | G | G | A | T | G | C | C | A | A | A | G | G | C | T | T | A | T | G | A | T | G | A | T | G | G | A | T | A | C | G | G | T | A | A |
| --- | --- | --- | --- | --- | --- | --- | --- | --- | --- | --- | --- | --- | --- | --- | --- | --- | --- | --- | --- | --- | --- | --- | --- | --- | --- | --- | --- | --- | --- | --- | --- | --- | --- | --- | --- | --- | --- | --- | --- | --- | --- | --- | --- | --- | --- | --- | --- | --- | --- | --- | --- | --- | --- | --- | --- | --- | --- | --- | --- | --- |
| S8C9 | T | G | A | T | T | C | G | A | T | C | A | A | G | A | A | A | C | G | T | G | A | G | A | A | A | T | G | G | A | T | G | C | C | A | A | C | G | G | C | T | T | A | T | G | A | T | G | A | T | G | G | A | T | A | C | G | G | T | A | A |

D S I K K R **E** K W M P **T** A Y D D G Y G **K**

**P** T I G Y G H L I K P G D G L H M G S T

| L7h8 | T | C | C | G | A | C | A | A | T | T | G | G | T | T | A | T | G | G | A | C | A | T | T | T | G | A | T | C | A | A | A | C | C | C | G | G | A | G | A | T | G | G | T | T | T | G | C | A | T | A | T | G | G | G | C | T | C | G | A | C |
| --- | --- | --- | --- | --- | --- | --- | --- | --- | --- | --- | --- | --- | --- | --- | --- | --- | --- | --- | --- | --- | --- | --- | --- | --- | --- | --- | --- | --- | --- | --- | --- | --- | --- | --- | --- | --- | --- | --- | --- | --- | --- | --- | --- | --- | --- | --- | --- | --- | --- | --- | --- | --- | --- | --- | --- | --- | --- | --- | --- | --- |
| S8C9 | A | C | T | G | A | C | A | A | T | T | G | G | T | T | A | T | G | G | A | C | A | T | T | T | G | A | T | C | A | A | A | C | C | C | G | G | A | G | A | T | G | G | T | T | T | G | C | A | T | A | T | G | G | G | C | T | C | G | A | C |

**L** T I G Y G H L I K P G D G L H M G S T

I T K K Q G E K L F Q D D L **V** V F E **R** C

| L7h8 | A | A | T | A | A | C | A | A | A | G | A | A | A | C | A | G | G | G | T | G | A | G | A | A | A | C | T | T | T | T | C | C | A | G | G | A | T | G | A | T | C | T | T | G | T | C | G | T | G | T | T | T | G | A | A | A | G | G | T | G |
| --- | --- | --- | --- | --- | --- | --- | --- | --- | --- | --- | --- | --- | --- | --- | --- | --- | --- | --- | --- | --- | --- | --- | --- | --- | --- | --- | --- | --- | --- | --- | --- | --- | --- | --- | --- | --- | --- | --- | --- | --- | --- | --- | --- | --- | --- | --- | --- | --- | --- | --- | --- | --- | --- | --- | --- | --- | --- | --- | --- | --- |
| S8C9 | A | A | T | A | A | C | A | A | A | G | A | A | A | C | A | G | G | G | T | G | A | G | A | A | A | C | T | T | T | T | C | C | A | G | G | A | T | G | A | T | C | T | T | G | C | C | G | T | G | T | T | T | G | A | A | A | G | T | T | G |

I T K K Q G E K L F Q D D L **A** V F E **S** C

V Q A F K P K E P L N D N E F G A L V S

| L7h8 | T | G | T | T | C | A | A | G | C | A | T | T | C | A | A | A | C | C | T | A | A | A | G | A | A | C | C | G | C | T | C | A | A | T | G | A | T | A | A | C | G | A | A | T | T | C | G | G | T | G | C | A | C | T | T | G | T | T | T | C |
| --- | --- | --- | --- | --- | --- | --- | --- | --- | --- | --- | --- | --- | --- | --- | --- | --- | --- | --- | --- | --- | --- | --- | --- | --- | --- | --- | --- | --- | --- | --- | --- | --- | --- | --- | --- | --- | --- | --- | --- | --- | --- | --- | --- | --- | --- | --- | --- | --- | --- | --- | --- | --- | --- | --- | --- | --- | --- | --- | --- | --- |
| S8C9 | T | G | T | T | C | A | A | G | C | A | T | T | C | A | A | A | C | C | T | A | A | A | G | A | A | C | C | G | C | T | C | A | A | T | G | A | T | A | A | C | G | A | A | T | T | C | G | G | T | G | C | A | C | T | T | G | T | T | T | C |

V Q A F K P K E P L N D N E F G A L V S

W S Y N I G C G **A** A T G S T L V K K L Q

| L7h8 | C | T | G | G | A | G | C | T | A | T | A | A | C | A | T | T | G | G | C | T | G | C | G | G | A | G | C | T | G | C | A | A | C | C | G | G | T | T | C | A | A | C | G | C | T | T | G | T | T | A | A | A | A | A | A | T | T | G | C | A |
| --- | --- | --- | --- | --- | --- | --- | --- | --- | --- | --- | --- | --- | --- | --- | --- | --- | --- | --- | --- | --- | --- | --- | --- | --- | --- | --- | --- | --- | --- | --- | --- | --- | --- | --- | --- | --- | --- | --- | --- | --- | --- | --- | --- | --- | --- | --- | --- | --- | --- | --- | --- | --- | --- | --- | --- | --- | --- | --- | --- | --- |
| S8C9 | C | T | G | G | A | G | C | T | A | T | A | A | C | A | T | T | G | G | C | T | G | C | C | C | A | G | C | A | G | C | A | A | C | C | G | G | T | T | C | A | A | C | G | C | T | T | G | T | T | A | A | A | A | A | A | T | T | G | C | A |

W S Y N I G C P **A** A T G S T L V K K L Q

E G K K D E V C K E L R R

| L7h8 | G | G | A | G | G | G | C | A | A | A | A | A | A | G | A | T | G | A | G | G | T | T | T | G | C | A | A | G | G | A | G | C | T | A | C | G | G | A | G | A | **T** | **A** | **G** | A | A | T | A | A | A | G | C | C | A | A | C | A | A | A | A | A |
| --- | --- | --- | --- | --- | --- | --- | --- | --- | --- | --- | --- | --- | --- | --- | --- | --- | --- | --- | --- | --- | --- | --- | --- | --- | --- | --- | --- | --- | --- | --- | --- | --- | --- | --- | --- | --- | --- | --- | --- | --- | --- | --- | --- | --- | --- | --- | --- | --- | --- | --- | --- | --- | --- | --- | --- | --- | --- | --- | --- | --- |
| S8C9 | G | G | A | G | G | G | C | A | A | A | A | A | A | G | A | T | G | A | G | G | T | T | T | G | C | G | A | T | G | A | G | C | T | A | C | G | G | A | G | A | T | G | G | A | A | T | A | A | A | G | C | C | A | A | C | A | A | A | A | A |

E G K K D E V C D E L R R W N K A N K K

| L7h8 | A | G | T | A | T | C | C | G | A | A | G | G | C | C | T | C | A | A | A | G | C | A | A | G | A | A | G | A | G | A | A | G | A | A | G | A | A | T | G | T | A | A | A | C | T | T | T | T | C | A | C | T | T | C | T | A | A | A | T | G |
| --- | --- | --- | --- | --- | --- | --- | --- | --- | --- | --- | --- | --- | --- | --- | --- | --- | --- | --- | --- | --- | --- | --- | --- | --- | --- | --- | --- | --- | --- | --- | --- | --- | --- | --- | --- | --- | --- | --- | --- | --- | --- | --- | --- | --- | --- | --- | --- | --- | --- | --- | --- | --- | --- | --- | --- | --- | --- | --- | --- | --- |
| S8C9 | A | G | T | A | T | C | C | G | A | A | G | G | C | C | T | C | A | A | A | G | C | A | A | G | A | A | G | A | G | A | A | G | A | A | G | A | A | T | G | T | G | C | A | C | T | T | T | T | C | A | C | T | T | C | T | C | A | A | **T** | **G** |

V S E G L K A R R E E E C A L F T S Q

| L7h8 | A | T | T | C | G | A | A | A | T | T | T | T | C | T | T | T | G | A | A | T | G | T | T | A | A | A | C | T | T | C | A | C | C | A | A | T | A | A | A | C | A | C | T | T | T | T | A | T | C | A | A | A | G | A | C | C | A | A | T |
| --- | --- | --- | --- | --- | --- | --- | --- | --- | --- | --- | --- | --- | --- | --- | --- | --- | --- | --- | --- | --- | --- | --- | --- | --- | --- | --- | --- | --- | --- | --- | --- | --- | --- | --- | --- | --- | --- | --- | --- | --- | --- | --- | --- | --- | --- | --- | --- | --- | --- | --- | --- | --- | --- | --- | --- | --- | --- | --- | --- |
| S8C9 | **A** | T | T | C | G | G | A | A | T | T | T | T | C | T | T | T | G | A | A | T | G | T | T | A | A | A | C | T | T | C | A | C | C | A | A | T | A | A | A | C | A | C | T | T | T | T | A | T | C | A | A | A | G | A | C | A | A | C | - |
|  |  |  |  |  |  |  |  |  |  |  |  |  |  |  |  |  |  |  |  |  |  |  |  |  |  |  |  |  |  |  |  |  |  |  |  |  |  |  |  |  |  |  |  |  |  |  |  |  |  |  |  |  |  |  |  |  |  |  |  |

Figure S4. Sequence alignments of cDNA pairs that share 80 to 95% sequence identity in their MPCR. cDNA pairs belong to groups *SSSGP-1* (A)**,** *SSSGP-2* (B, C, D), *SSSGP-4* (E, F, G, H), *SSSGP-6* (I), *SSSGP-7* (J), *SSSGP-10* (K), *SSSGP-26* (L), *SSSGP-31* (M), *SSSGP-37* (N), *SSSGP-79* (O), and *SSSGP-80* (P), respectively. Putative signal peptides are bold and italic. Amino acid changes resulting from nonsynonymous substitutions are in red whereas amino acids not affected by synonymous substitutions are in blue. Only single nucleotide substitutions in individual codons were counted.
