# Supplementary material for: Unusual conservation among genes encoding small secreted salivary gland proteins from a gall midge
Source: BMC Evol Biol. 2010 Sep 28;10:296. doi: 10.1186/1471-2148-10-296 (PMC2955719; doi:10.1186/1471-2148-10-296)
Supplement: Additional file 5 — Figure S5: Evidence for single location of genes in the SSSGP-1 family. [file 1471-2148-10-296-S5.DOC]

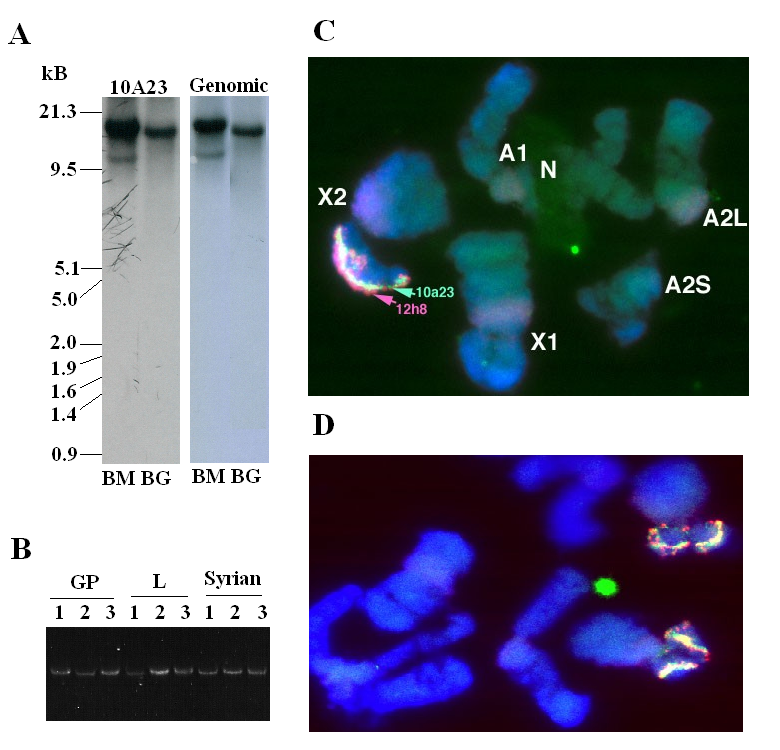


Figure S5. Evidence for a single copy of *SSSGP-1A2*, *1B1*, and *1C1*. **A:** Southern blots of BAC clone 10A23 (from which the sequences of the *SSSGP-1* gene family members were derived) and genomic DNA samples. DNA samples were digested with BamH1 (BM) and Bgl1 (BG) restriction enzymes and separated on a 0.8% agarose gel. The blot was hybridized to a ^32^P-labeled probe derived from the *SSSGP-1A1* cDNA. The same hybridization pattern was observed for both the BAC and genomic DNA samples. The single 15 kB Bgl I band in both BAC and genomic DNA samples suggests a single location of the BAC insert in Hessian fly genome. **B:** PCR analysis of genomic DNA samples isolated from individual insects from three different populations: biotype GP, biotype L, and a Syrian population. The primer pair for the PCR reaction covers the second exon of *SSSGP-1A2* (5’-CATGGAAAGATCGTCAAATTGGT) and second exon of *SSSGP-1C1* (5’-TTCGAAATATGATTCGAAATCTT). Each PCR reaction resulted in a single band with expected size (5.7 kB). The single PCR band from individual insects from different populations was also consistent with a single copy of the genes. **C:** *In situ* hybridization with the BAC clone 10A23 as a probe. A single hybridization band corresponding to 10A23 (green fluorescence) was observed, indicating a single location of the BAC. **D:** A different view of the same *in situ* hybridization. *In situ* hybridization was carried as described previously (Chen et al., 2004). The overall evidence suggests a single cluster of the seven genes in the Hessian fly genome.
