# Supplementary material for: Unusual conservation among genes encoding small secreted salivary gland proteins from a gall midge
Source: BMC Evol Biol. 2010 Sep 28;10:296. doi: 10.1186/1471-2148-10-296 (PMC2955719; doi:10.1186/1471-2148-10-296)
Supplement: Additional file 6 — Figure S6: Sequence alignment of similar SSSGP-encoding cDNAs (presumably derived from different alleles). [file 1471-2148-10-296-S6.DOC]

**A**

L9F5 CAGTTCAATTCGAACCATTAAT**A**CTTTCAATATCCACTGGAACATCCAAAAACGAAA**ATG** 60

G38F8 CAGTTCAATTCGAACCATTAATTCTTTCAATATCCACTGGAACATCCAAAAACGAAA**ATG** 60

S21E5 CAGTT**TG**A**A**T**T**GAACC**G**TTAATTCTTTCAATATCCACTGGAACATCCAAAAACGAAA**ATG** 60

G16C5 CAGTTCAATTCGAACCATTAATTCTTTCAATATCCACTGGAACATCCAAAAACGAAA**ATG** 60

S8C8 --GTT**TG**A**A**T**T**GAACC**G**TTAATTCTTTCAATATCCACTGGAACATCCAAAAACGAAA**ATG** 58

G16E1 CAGTTCAATTCGAACCATTAATTCTTTCAATATCCACTGGAACATCCAAAAACGAAA**ATG** 60

S4C12 CAGTT**TG**A**A**T**T**GAACC**G**TTAATTCTTTCAATATCCACTGGAACATCCAAAAACGAAA**ATG** 60

L6G1 CAGTTCAATTCGAACCATTAATTCTTTCAATATCCACT**A**GAACATCCAAAAACGAAA**ATG** 60

L6G2 CAGTTCAA**C**TCGAACC**G**TTAATTCTTTCAATATCCACTGGAACATCCAAAAACGAAA**ATG** 60

S8H3 CAGTT**TG**A**A**T**T**GAACC**G**TTAATTCTTTCAATATCCACTGGAACATCCAAAAACGAAA**ATG** 60

S19E10 --------------------------------------GGAACATCCAAAAACGAAA**ATG** 22

S21C5 CAGTT**TG**A**A**T**T**GAACC**G**TTAATTCTTTCAATATCCACTGGAACATCCAAAAACGAAA**ATG** 60

G8A6 CAGTTCAATTCGAACCATTAATTCTTTCAATATCCACTGGAACATCCAAAAACGAAA**ATG** 60

G28H7 CAGTTCAATTCGAACCATTAATTCTTTCAATATCCACTGGAACATCCAAAAACGAAA**ATG** 60

L6B5 CAGTTCAATTCGAACC**G**TTAATTCTTTCAATATCCACTGGAACATCCAAAAACGAAA**ATG** 60

L1H9 CAGTTCAATTCGAACCA**C**TAATTCTTTCAATATCCACTGGAACATCCAAAAACGAAA**ATG** 60

L6E2 CAGTTCAATTCGAACCA**C**TAATTCTTTCAATATCCACTGGAACATCCAAAAACGAAA**ATG** 60

G39G10 CAGTTCAATTCGAACCATTAATTCTTTCAATATCCACTGGAACATCCAAAAACGAAA**ATG** 60

G27G6 CAGTTCAATTCGAACCATTAATTCTTTCAATATCCACTGGAACATCCAAAAACGAAA**ATG** 60

L4E1 CAGTTCAATTCGAACCATTAATTCTTTCAATATCCACTGGAACATCCAAAAACGAAA**ATG** 60

G8H8 CAGTTCAATTCGAACCATTAATTCTTTCAATATCCACTGGAACATCCAAAAACGAAA**ATG** 60

G39B9 CAGTT**-**AATTCGAACC**T**TTAATTCTTTCAATATCCACTGGAACATCCAAAAACGAAA**ATG** 59

L2C8 CAGTTCAATTCGAACCATTAATTCTTTCAATATCCACTGGAACATCCAAAAACGAAA**ATG** 60

S11D7 --------------------------------------GGAACATCCAAAAACGAAA**ATG** 22

L6D6 CAGTTCAATTCGAACCATTAATTCTTTCAATATCCACT**A**GAACATCCAAAAACGAAA**ATG** 60

L9F12 CAGTTCAATTCGAACCATTAATTCTTTCAATATCCACTGGAACATCCAAAAACGAAA**ATG** 60

G8B4 CAGTTCAATTCGAACCATTAATTCTTTCAATATCCACTGGAACATCCAAAAACGAAA**ATG** 60

G3G5 CAGTTCAATTCGAACCATTAATTCTTTCAATATCCACTGGAACATCCAAAAACGAAA**ATG** 60

G27E6 CAGTTCAATTCGAACCATTAATTCTTTCAATATCCACTGGAACATCCAAAAACGAAA**ATG** 60

G5D4 CAGTTCAATTCGAACC**G**TTAATTCTTTCAATATCCACTGGAACATCCAAAAACGAAA**ATG** 60

G1A5 CAGTTCAATTCGAACCATTAAT**A**CTTTCAATATCCACTGGAACATCCAAAAACGAAA**ATG** 60

G6G2 CAGTTCAATTCGAACCATTAATTCTTTCAATATCCACTGGAACATCCAAAAACGAAA**ATG** 60

G34G4 CAGTTCAATTCGAACCATTAAT**A**CTTTCAATATCCACTGGAACATCCAAAAACGAAA**ATG** 60

G38B12 -AGTTCAATTCGAACCATTAATTCTTTCAATATCCACTGGAACATCCAAAAACGAAA**ATG** 59

G8B10 CAGTTCAATTCGAACCATTAATTCTTTCAATATCCACTGGAACATCCAAAAACGAAA**ATG** 60

G1H6 CAGTTCAATTCGAACCATTAATTCTTTCAATATCCACT**A**GAACATCCAAAAACGAAA**ATG** 60

G36E3 CAGTTCAATTCGAACCATTAATTCTTTCAATATCCACTGGAACATCCAAAAACGAAA**ATG** 60

G4H9 CAGTTCAATTCGAACCATTAATTCTTTCAATATCCACTGGAACATCCAAAAACGAAA**ATG** 60

G33B10 CAGTTCAATTCGAACCATTAATTCTTTCAATATCCACTGGAACATCCAAAAACGAAA**ATG** 60

L3A11 CAGTTCAATTCGAACCATTAATTCTTTCAATATCCACTGGAACATCCAAAAACGAAA**ATG** 60

L6H9 CAGTTCAATTCGAACC**G**TTAATTCTTTCAATATCCACTGGAACATCCAAAAACGAAA**ATG** 60

L5E1 CAGTTCAATTCGAACCATTAATTCTTTCAATATCCACTGGAACATCCAAAAACGAAA**ATG** 60

L3G10 CAGTTCAATTCGAACCATTAATTCTTTCAATATCCACT**A**GAACATCCAAAAACGAAA**ATG** 60

L7D10 CAGTTCAATTCGAACCATTAATTCTTTCAATATCCACTGGAACATCCAAAAACGAAA**ATG** 60

L7B7 CAGTTCAATTCGAACC**G**TTAATTCTTTCAATATCCACTGGAACATCCAAAAACGAAA**ATG** 60

L7G9 CAGTTCAATTCGAACCATTAATTCTTTCAATATCCACTGGAACATCCAAAAACGGAA**ATG** 60

L2F3 CAGTTCAATTCGAACCATTAATTCTTTCAATATCCACTGGAACATCCAAAAACGAAA**ATG** 60

L1F3 CAGTTCAATTCGAACC**G**TTAATTCTTTCAATATCCACTGGAACATCCAAAAACGAAA**ATG** 60

L1D3 CAGTTCAATTCGAACCATTAATTCTTTCAATATCCACT**A**GAACATCCAAAAACGAAA**ATG** 60

L4D7 CAGTTCAATTCGAACC**G**TTAATTCTTTCAATATCCACTGGAACATCCAAAAAC**T**AAA**ATG** 60

L10A5 CAGTTCAATTCGAACCATTAATTCTTTCAATATCCACTGGAACATCCAAAAACGAAA**ATG** 60

L3D4 CAGTTCAATTCGAACCATTAATTCTTTCAATATCCACT**A**GAACATCCAAAAACGAAA**ATG** 60

L8D6 CAGTTCAATTCGAACC**G**TTAATTCTTTCAATATCCACTGGAACATCCAAAAACGAAA**ATG** 60

S6F4 CAGTTCAATTCGAACCATTAATTCTTTCAATATCCACTGGAACATCCAAAAACGAAA**ATG** 60

S19H4 CAGTTCAATTCGAACCATTAAT**A**CTTTCAATATCC**G**CTGGAACATCCAAAAACGAAA**ATG** 60

S6C12 CAGTTCAATTCGAACCATTAATTCTTTCAATATCCACTGGAACATCCAAAAACGAAA**ATG** 60

L1H10 CAGTTCAATTCGAACC**G**TTAATTCTTTCAATATCCACTGGAACATCCAAAAAC**T**AAA**ATG** 60

L5G9 CAGTTCAATTCGAACCATTAATTCTTTCAATATCCACTGGAACATCCAAAAACGAAA**ATG** 60

S8E10 CAGTT**TG**A**A**T**T**GAACC**G**TTAATTCTTTCAATATCCACTGGAACATCCAAAAACGAAA**ATG** 60

S18B4 CAGTT**TG**A**A**T**T**GAACC**G**TTAATTCTTTCAATATCCACTGGAACATCCAAAAACGAAA**ATG** 60

G34G2 CAATTCAATTCGAACCATTAATTCTTTCAATATCCACTGGAACATCCAAAAACGAAA**ATG** 60

G16C1 CAGTTCAATTCGAACCATTAATTCTTTCAATATCCACTGGAACATCCAAAAACGAAA**ATG** 60

S18G4 CAGTT**TG**A**A**T**T**GAACC**G**TTAATTCTTTCAATATCCACTGGAACATCCAAAAACGAAA**ATG** 60

S16F12 CAGTT**TG**A**A**T**T**GAACC**G**TTAATTCTTTCAATATCCACTGGAACATCCAAAAACGAAA**ATG** 60

G27D12 CAGTTCAATTCGAACCATTAATTCTTTCAATATCCACTGGAACATCCAAAAACGAAA**ATG** 60

G2H6 CAGTTCAATTCGAACCATTAAT**A**CTTTCAATATCCACTGGAACATCCAAAAACGAAA**ATG** 60

L2F4 CAGTTCAATTCGAACC**G**TTAATTCTTTCAATATCCACTGGAACATCCAAAAACGAAA**ATG** 60

S21G8 CAGTTCAATTCGAACCATTAAT**A**CTTTCAATATCCACTGGAACATCCAAAAACGAAA**ATG** 60

G1C2 CAGTTCAATTCGAACCATTAATTCTTTCAATATCCACTGGAACATCCAAAAACGAAA**ATG** 60

S16A9 CAGTT**TG**A**A**T**T**GAACC**G**TTAATTCTTTCAATATCCACTGGAACATCCAAAAACGAAA**ATG** 60

G5G10 CAGTTCAATTCGAACCATTAATTCTTTCAATATCCACTGGAACATCCAAAAACGAAA**ATG** 60

L9F5 TCAAAATTTTTACTAGCTTTCGCCGTCATCGCCGTCTGCCTTGT**C**GCAGCTCAGGCTGCT 120

G38F8 TCAAAATTTTT**G**CTAGCTTTCGCCGTCATCGCCGTCTGCCTTGTTGCAGCTCAGGCTGCT 120

S21E5 TCAAAATTTTTACTAGCTTTCGCCGTCATCGCCGTCTGCCTTGTTGCAGCTCAGGCTGCT 120

G16C5 TCAAAATTTTTACTAGCTTTCGCCGTCATCGCCGTCTGCCTTGTTGCAGCTCAGGCTGCT 120

S8C8 TCAAAATTTTTACTAGCTTTCGCCGTCATCGCCGTCTGCCTTGTTGCAGCTCAGGCTGCT 118

G16E1 TCAAAATTTTTACTAGCTTTCGCCG**C**CATCGCCGTCTGCCTTGTTGCAGCTCAGGCTGCT 120

S4C12 TCAAAATTTTTACTAGCTTTCGCCG**C**CATCGCCGTCTGCCT**C**GTTGCAGCTCAGGCTGCT 120

L6G1 TCAAAATTTTTACTAGCTTTCGCCGTCATCGC**T**GTCTGCCTTGTTGCAGCTCAGGCTGCT 120

L6G2 TCAAAATTTTTACTAGCTTTCGCCGTCATCGCCGTCTGCCTTGTTGCAGCTCAGGCTGCT 120

S8H3 TCAAAATTTTTACTAGCTTTCGCCGTCATCGCCGTCTGCCTTGTTGCAGCTCAGGCTGCT 120

S19E10 TCAAAATTTTTACTAGCTTTCGCCGTCATCGCCGTCTGCCTTGTTGCAGCTCAGGCTGCT 82

S21C5 TCAAAATTTTTACTAGCTTTCGCCGTCATCGCCGTCTGCCTTGTTGCAGCTCAGGCTGCT 120

G8A6 TCAAAATTTTTACTAGCTTTCGCCGTCATCGCCGTCTGCCTTGTTGCAGCTCAGGCTGCT 120

G28H7 TCAAAATTTTTACTAGCTTTCGCCGTCATCGCCGTCTGCCTTGTTGCAGCTCAGGCTGCT 120

L6B5 TCAAAATTTTTACTAGCTTTCGCCGTCATCGCCGTCTGCCTTGTTGCAGCTCAGGCTGCT 120

L1H9 TCAAAATTTTTACTAGCTTTCGCCGTCATCGCCGTCTGCCT**C**GTTGCAGCTCAGGCTGCT 120

L6E2 TCAAAATTTTTACTAGCTTTCGCCGTCATCGCCGTCTGCCTTGTTGCAGCTCAGGCTGCT 120

G39G10 TCAAAATTTTTACTAGCTTTCGCCGTCATCGCCGTCTGCCTTGTTGCAGCTCAGGCTGCT 120

G27G6 TCAA**G**ATTTTTACTAGCTTTCGCCGTCATCGCCGTCTGCCTTGTTGCAGCTCAGGCTGCT 120

L4E1 TCAAAATTTTTACTAGCTTTCGCCGTCA**A**CGCCGTCTGCCTTGTTGCAGCTCAGGCTGCT 120

G8H8 TCAAAATTTTTACTAGCTTTCGCCGTCATCGCCGTCTGCCTTGTTGCAGCTCAGGCTGCT 120

G39B9 TCAAAATTTTTACTAG**G**TTTCGCCGT**G**ATCGCCGTCTGCCTTGTTGCAGCTCAGGCTGCT 119

L2C8 TCAAAATTTTT**G**CTAGC**A**TTCGCCGTCATCGCCGTCTGCCTTGTTGCAGCTCAGGCTGCT 120

S11D7 TCAAAATTTTTACTAGCT**C**TCGCCGTCATCGCCGTCTGCCTTGTTGCAGCTCAGGCCGCT 82

L6D6 TCAAAATTTTTACTAGCTTTCGCCGTCATCGCCGTCTGCCTTGTTGCAGCTCAGGCTGCT 120

L9F12 TCAAAATTTTTACTAGCTTTCGCCGTCATCGCCGTCTGCCTTGTTGCAGCTCAGGCTGCT 120

G8B4 TCAAAATTTTTACTAGCTTTCGCCGTCATCGCCGTCTGCCTTGTTGCAGCTCAGGCTGCT 120

G3G5 TCAAAATTTTTACTAGCTTTCGCCGTCATCGCCGTCTGCCTTGTTGCAGCTCAGGCTGCT 120

G27E6 TCAAAATTTTTACTAGCTTTCGCCGTCATCGCCGTCTGCCTTGTTGCAGC**A**CAGGCTGCT 120

G5D4 TCAAAATTTTTACTAGCTTTCGC**T**GTCATCGC**T**GTCTGCCTTGTTGCAGCTCAGGCTGCT 120

G1A5 TCAAAATTTTTACTAGCTTTCGCCGTCATCGC**T**GTCTGCCTTGTTGCAGCTCAGGCTGCT 120

G6G2 TCAAAATTTTTACTAGCTTTCGCCGTCATCGCCGTCTGCCTTGTTGCAGCTCAGGCTGCT 120

G34G4 TCAAAATTTTTACTAGCTTTCGCCGTCATCGC**T**GTCTGCCTTGTTGCAGCTCAGGCTGCT 120

G38B12 TCAAAATTTTTACTAGCTTTCGCCGTCATCGCCGTCTGCCTTGTTGCAGCTCAGGCTGCT 119

G8B10 TCAAAATTTTTACTAGCTTTCGCCGTCATCGCCGTCTGCCTTGTTGCAGCTCAGGCTGCT 120

G1H6 TCAAAATTTTTACTAGCTTTCGCCGTCATCGC**T**GTCTGCCTTGTTGCAGCTCAGGCTGCT 120

G36E3 TCAAAATTTTTACTAGCTTTCGCCGTCATCGCCGTCTGCCTTGTTGCAGCTCAGGCTGCT 120

G4H9 TCAAAATTTTTACTAGCTTTCGCCGTCATCGCCGTCTGCCTTGTTGCAGCTCAGGCTGCT 120

G33B10 TCAAAATTTTTACTAGCTTTCGCCGTCATCGCCGTCTGCCTTGTTGCAGCTCAGGCTGCT 120

L3A11 TCAAAATTTTTACTAGCTTTCGCCGTCATCGCCGTCTGCCTTGTTGCAGCTCAGGCTGCT 120

L6H9 TCAAAATTTTTACTAGCTTTCGCCGTCATCGCCGTCTGCCTTGTTGCAGCTCAGGCTGCT 120

L5E1 TCAAAATTTTTACTAGCTTTCGCCGTCATCGCCGTCTGCCTTGTTGCAGCTCAGGCTGCT 120

L3G10 TCAAAATTTTTACTAGCTTTCGCCGTCATCGC**T**GTCTGCCTTGTTGCAGCTCAGGCTGCT 120

L7D10 TCAAAATTTTTACTAGCTTTCGCCGTCATCGCCGTCTGCCTTGTTGCAGCTCAGGCTGCT 120

L7B7 TCAAAATTTTTACTAGCTTTCGCCGTCATCGCCGTCTGCCTTGTTGCAGCTCAGGCTGCT 120

L7G9 TCAAAATTTTTACTAGCTTTCGCCGTCATCGCCGTCTGCCTTGTTGCAGCTCAGGCTGCT 120

L2F3 TCAAAATTTTTACTAGCTTTCGCCGTCATCGCCGTCTGCCTTGTTGCAGCTCAGGCTGCT 120

L1F3 TCAAAATTTTTACTAGCTTTCGCCGTCATCGCCGTCTGCCTTGTTGCAGCTCAGGCTGCT 120

L1D3 TCAAAATTTTTACTAGCTTTCGCCGTCATCGC**T**GTCTGCCTTGTTGCAGCTCAGGCTGCT 120

L4D7 TCAAAATTTTTACTAGCTTTCGCCGTCATCGCCGTCTGCCTTGTTGCAGCTCAGGCTGCT 120

L10A5 TCAAAATTTTTACTAGCTTTCGCCGTCATCGCCGTCTGCCTTGTTGCAGCTCAGGCTGCT 120

L3D4 TCAAAATTTTTACTAGCTTTCGCCGTCATCGC**T**GTCTGCCTTGTTGCAGCTCAGGCTGCT 120

L8D6 TCAAAATTTTTACTAGCTTTCGCCGTCATCGCCGTCTGCCTTGTTGCAGCTCAGGCTGCT 120

S6F4 TCAAAATTTTTACTAGCTTTCGCCGTCATCGCCGTCTGCCTTGTTGCAGCTCAGGCTGCT 120

S19H4 TCAAAATTTTTACTAGCTTTCGCCGTCATCGCCGTCTGCCTTGTTGCAGCTCAGGCTGCT 120

S6C12 TCAAAATTTTTACTAGCTTTCGCCGTCATCGCCGTCTGCCTTGTTGCAGCTCAGGCTGCT 120

L1H10 TCAAAATTTTTACTAGCTTTCGCCGTCATCGCCGTCTGCCTTGTTGCAGCTCAGGCTGCT 120

L5G9 TCAAAATTTTTACTAGCTTTCGCCGTCATCGCCGTCTGCCTTGTTGCAGCTCAGGCTGCT 120

S8E10 TCAAAATTTTTACTAGCTTTCGCCGTCATCGCCGTCTGCCTTGTTGCAGCTCAGGCTGCT 120

S18B4 TCAAAATTTTTACTAGCTTTCGCCGTCATCGCCGTCTGCCTTGTTGCAGCTCAGGCTGCT 120

G34G2 TCAAAATTTTTACTAGCTTTCGCCGTCATCGCCGTCTGCCTTGTTGCAGCTCAGGCTGCT 120

G16C1 TCAAAATTTTTACTAGCTTTCGCCG**C**CATCGCCGTCTGCCTTGTTGCAGCTCAGGCTGCT 120

S18G4 TCAAAATTTTTACTAGCTTTCGCCGTCATCGCCGTCTGCCTTGTTGCAGCTCAGGCTGCT 120

S16F12 TCAAAATTTTTACTAGCTTTCGCCGTCATCGCCGTCTGCCTTGTTGCAGCTCAGGCTGCT 120

G27D12 TCAAAATTTTTACTAGCTTTCGCCGTCATCGCCGTCTGCCTTGTTGCAGCTCAGGCTGCT 120

G2H6 TCAAAATTTTTACTAGCTTTCGCCGTCATCGCCGTCTGCCTTGTTGCAGCTCAGGCTGCT 120

L2F4 TCAAAATTTTTACTAGCTTTCGCCGTCATCGCCGTCTGCCTTGTTGCAGCTCAGGCTGCT 120

S21G8 TCAAAATTTTTACTAGCTTTCGCCGTCATCGCCGTCTGCCTTGTTGCAGCTCAGGCTGCT 120

G1C2 TCAAAATTTTT**G**CTAGCTTTCGCCGTCATCGCCGTCTGCCTTGTTGCAGCTCAGGCTGCT 120

S16A9 TCAAAATTTTTACTAGCTTTCGCCGTCATCGCCGTCTGCCTTGTTGCAGCTCAGGCTGCT 120

G5G10 TCAAAATTTTTACTAGCTTTCGCCGTCATCGCCGTCTGCCTTGTTGCAGCTCAGGCTGCT 120

L9F5 **G**TAACTAAACATCCAGCAGGAAAAAAGTCCCCAGCTAAACC**C**GCAAGTCCAACCCATGCA 180

G38F8 **G**TAACTAAACATCCAGCAGGAAAAAAGTCCCCAGCTAAACCAGCAAGTCCAACCCATGCA 180

S21E5 **G**TAACTAAACATCCAGCAGGAAAAAAGTCCCCAGCTAAACCAGCAAGTCCAACCCATGCA 180

G16C5 **G**TAAC**C**AAACATCCAGCAGGAAAAAAGTCCCCAGCTAAACCAGCAAGTCCAACCCATGCA 180

S8C8 **GC**AA**A**TAAACATCCAGCAGGAAAAAAGTCCCCAGCTAAACCAGCAAGTCCAACCCATGCA 178

G16E1 **G**TAAC**C**AAACATCCAGCAGGAAAAAAGTCCCCAGCTAAACCAGCAAGTCCAACCCATGCA 180

S4C12 **G**TAACTAAACATCCAGCAGGAAAAAAGTCCCCAGCTAAACCAGCAAGTCCAACCCATGCA 180

L6G1 **G**TAACTAAACATCCAGCAGGAAAAA**G**GTCCCCAGCTAAACCAGCAAGTCCAACCCATGCA 180

L6G2 **G**TAACTAAACATCCAGCAGGAAAAA**G**GTCCCCAGCTAAACCAGCAAGTCCAACCCATGCA 180

S8H3 **GC**AA**A**TAAACATCCAGCAGGAAAAAAGTCCCCAGCTAAACCAGCAAGTCCAACCCATGCA 180

S19E10 **GC**AA**A**TAAACATCCAGCAGGAAAAAAGTCCCCAGCTAAACCAGCAAGTCCAACCCATGCA 142

S21C5 **GC**AA**A**TAAACATCCAGCAGGAAAAAAGTCCCCAGCTAAACCAGCAAGTCCAACCCATGCA 180

G8A6 **G**TAAC**C**AAACATCCAGCAGGAAAAAAGTCCCCAGCTAAACCAGCAAGTCCAACCCATGCA 180

G28H7 **G**TAACTAAACATCCAGCAGGAAAAAAGTCCCCAGCTAAACC**T**GCAAGTCCAACCCATGCA 180

L6B5 **G**TAACTAAACATCCAGCAGGAAAAAAGTCCCCAGCTAAACCAGCAAGTCCAACCCATGCA 180

L1H9 **GC**AA**A**TAAACATCCAGCAGGGAAAAAGTCCCCAGCTAAACCAGCAAGTCCAACCCATGCA 180

L6E2 **GC**AA**A**TAAACATCCAGCAGGGAAAAAGTCCCC**T**GCTAAACCAGCAAGTCCAACCCATGCA 180

G39G10 **G**TAACTAAACATCCAGCAGGAAAAAAGTCCCCAGCTAAACCAGCAAGTCCAACCCATGCA 180

G27G6 **G**TAAC**C**AAACATCCAGCAGGAAAAAAGTCCCCAGCTAAACCAG**T**AAGTCCAACCCATGCA 180

L4E1 **G**TAAC**C**AAACATCCAGCAGGAAAAAAGTCCCCAGCTAAACCAGCAAGTCCAACCCATGCA 180

G8H8 **G**TAAC**C**AAACATGCAGCAGGAAAAAAGTCCCCAGCTAAACCAGCAAGTCCAACCCATGCA 180

G39B9 **G**TAACTAAACATCCAGCAGGAAAAAAGTCCCCAGCTAAACC**T**GCAAGTCCAACCCATGCA 179

L2C8 **G**TAACTAAACATCCAGCAGGAAAAAAGTCCCCAGCTAAACCAGCAAGTCCAACCCATGCA 180

S11D7 **G**TAACTAAACATCCAGCAGGAAAAAAGTCCCCAGCTAAACCAGCAAGTCCAACCCATGCA 142

L6D6 **G**TAAC**C**AAACATCCAGCAGGAAAAAAGTCCCCAGCTAAACCAGCAAGTCCAACCCATGCA 180

L9F12 **G**TAAC**C**AAACATCCAGCAGGAAAAAAGTCCCCAGCTAAACCAGCAAGTCCAACCCATGCA 180

G8B4 **G**TAAC**C**AAACATCCAGCAGGAAAAAAGTCCCCAGCTAAACCAGCAAGTCCAACCCATGCA 180

G3G5 **G**TAACTAAACATCCAGCAGGAAAAAAGTCCCCAGCTAAACC**T**GCAAGTCCAACCCATGCA 180

G27E6 **G**TAAC**C**AAACATCCAGCAGGAAAAAAGTCCCCAGCTAAACCAGCAAGTCCAACCCATGCA 180

G5D4 **G**TAACTAAACATCCAGCAGGAAAAAAGTCCCCAGCTAAACCAGCAAGTCCAACCCATGCA 180

G1A5 **G**TAACTAAACATCCAGCAGGAAAAAA**A**TCCCCAGCTAAACC**T**GCAAGTCCAACCCATGCA 180

G6G2 **G**TAAC**C**AAACATCCAGCAGGAAAAAAGTCCCCAGCTAAACCAGCAAGTCCAACCCATGCA 180

G34G4 **G**TAACTAAACATCCAGCAGGAAAAAA**A**TCCCCAGCTAAACC**T**GCAAGTCCAACCCATGCA 180

G38B12 **G**TAAC**C**AAACATCCAGCAGGAAAAAAGTCCCCAGCTAAACCAGCAAGTCCAACCCATGCA 179

G8B10 **G**TAAC**C**AAACATCCAGCAGGAAAAAAGTCCCCAGCTAAACCAGCAAGTCCAACCCATGCA 180

G1H6 **G**TAACTAAACATCCAGCAGGAAAAAAGTCCCCAGCTAAACCAGCAAGTCCAACCCATGCA 180

G36E3 **G**TAAC**C**AAACATCCAGCAGGAAAAAAGTCCCCAGCTAAACCAGCAAGTCCAACCCATGCA 180

G4H9 **G**TAAC**C**AAACATCCAGCAGGAAAAAAGTCCCCAGCTAAACCAGCAAGTCCAACCCATGCA 180

G33B10 **G**TAAC**C**AAACATCCAGCAGGAAAAAAGTCCCCAGCTAAACCAGCAAGTCCAACCCATGCA 180

L3A11 **G**TAACTAAACATCCAGCAGGAAAAAAGTCCCCAGCTAAACCAGCAAGTCCAACCCATGCA 180

L6H9 **G**TAACTAAACATCCAGCAGGAAAAAA**A**TCCCCAGCTAAACC**T**GCAAGTCCAACCCATGCA 180

L5E1 **G**TAACTAAACATCCAGCAGGAAAAAAGTCCCCAGCTAAACC**T**GCAAGTCCAACCCATGCA 180

L3G10 **G**TAACTAAACATCCAGCAGGAAAAAAGTCCCCAGCTAAACCAGCAAGTCCAACCCATGCA 180

L7D10 **G**TAACTAAACATCCAGCAGGAAAAAA**A**TCCCCAGCTAAACC**T**GCAAGTCCAACCCATGCA 180

L7B7 **G**TAACTAAACATCCAGCAGGAAAAAA**A**TCCCCAGCTAAACC**T**GCAAGTCCAACCCATGCA 180

L7G9 **G**TAACTAAACATCCAGCAGGAAAAAAGTCCCCAGCTAAACC**T**GCAAGTCCAACCCATGCA 180

L2F3 **G**TAACTAAACATCCAGCAGGAAAAAAGTCCCCAGCTAAACC**T**GCAAGTCCAACCCATGCA 180

L1F3 **G**TAACTAAACATCCAGCAGGAAAAAAGTCCCCAGCTAAACCAGCAAGTCCAACCCATGCA 180

L1D3 **G**TAACTAAACATCCAGCAGGAAAAAAGTCCCCAGCTAAACCAGCAAGTCCAACCCATGCA 180

L4D7 **G**TAACTAAACATCCAGCAGGAAAAAAGTCCCCAGCTAAACCAGCAAGTCCAACCCATGCA 180

L10A5 **G**TAACTAAACATCCAGCAGGAAAAAAGTCCCCAGCTAAACC**T**GCAAGTCCAACCCATGCA 180

L3D4 **G**TAACTAAACATCCAGCAGGAAAAAAGTCCCCAGCTAAACCAGCAAGTCCAACCCATGCA 180

L8D6 **G**TAACTAAACATCCAGCAGGAAAAAAGTCCCCAGCTAAACCAGCAAGTCCAACCCATGCA 180

S6F4 **G**TAACTAAACATCCAGCAGGAAAAAAGTCCCCAGCTAAACCAGCAAGTCCAACCCATGCA 180

S19H4 **G**TAACTAAACATCCAGCAGGAAAAAAGTCCCCAGCTAAACCAGCAAGTCCAACCCATGCA 180

S6C12 **G**TAACTAAACATCCAGCAGGAAAAAAGTCCCCAGCTAAACCAGCAAGTCCAACCCATGCA 180

L1H10 **G**TAACTAAACATCCAGCAGGAAAAAAGTCCCCAGCTAAACCAGCAAGTCCAACCCATGCA 180

L5G9 **G**TAACTAAACATCCAGCAGGAAAAAAGTCCCCAGCTAAACCAGCAAGTCCAACCCATGCA 180

S8E10 **G**TAACTAAACATCCAGCAGGAAAAAAGTCCCCAGCTAAACCAGCAAGTCCAACCCATGCA 180

S18B4 **G**TAACTAAACATCCAGCAGGAAAAAAGTCCCCAGCTAAACCAGCAAGTCCAACCCATGCA 180

G34G2 **G**TAAC**C**AAACATCCAGCAGGAAAAAAGTCCCCAGCTAAACCAGCAAGTCCAACCCATGCA 180

G16C1 **G**TAAC**C**AAACATCCAGCAGGAAAAAAGTCCCCAGCTAAACCAGCAAGTCCAACCCATGCA 180

S18G4 **GC**AA**A**TAAACATCCAGCAGGAAAAAAGTCCCCAGCTAAACCAGCAAGTCCAACCCATGCA 180

S16F12 **GC**AA**A**TAAACATCCAGCAGGAAAAAAGTCCCCAGCTAAACCAGCAAGTCCAACCCATGCA 180

G27D12 **G**TAAC**C**AAACATCCAGCAGGAAAAAAGTCCCCAGCTAAACCAGCAAGTCCAACCCATGCA 180

G2H6 **G**TAACTAAACATCCAGCAGGAAAAAAGTCCCCAGCTAAACCAGCAAGTCCAACCCATGCA 180

L2F4 **G**TAACTAAACATCCAGCAGGAAAAAA**A**TCCCCAGCTAAACC**T**GCAAGTCCAACCCATGCA 180

S21G8 **G**TAACTAAACATCCAGCAGGAAAAAAGTCCCCAGCTAAACCAGCAAGTCCAACCCATGCA 180

G1C2 **G**TAACTAAACATCCAGCAGGAAAAAAGTCCCCAGCTAAACCAGCAAGTCCAACCCATGCA 180

S16A9 **G**TAACTAAACATCCAGCAGGAAAAAAGTCCCCAGCTAAACCAGCAAGTCCAACCCATGCA 180

G5G10 **G**TAAC**C**AAACATCCAGCAGGAAAAAAGTCCCCAGCTAAACCAGCAAGTCCAACCCATGCA 180

L9F5 GCAGCGCCAACAGC**T**CAATCAAACCCAGATGACCAATTTGACGATCTTTCCATGCTAGAT 240

G38F8 GCAGCGCCAACAGCCCAATCAAACCCAGATGACCAATTTGACGATCTTTCCATGCTAGAT 240

S21E5 GCAGCGCCAACAGCCCAATCAAACCCAGATGACCAATTTGACGAT**T**TTTCCATGCTAGAT 240

G16C5 GCAGCGCCAACAGCCCAATCAAACCCAGATGACCAATTTGACGATCTTTCCATGCTAGAT 240

S8C8 GCAGCGCCAACAGCCCAATCAAACCCAGATGACCAATTTGA**G**GAT**T**TTTCCATGCTAGAT 238

G16E1 GCAGCGCCAACAGCCCAATCAAACCCAGATGACCAATTTGACGATCTTTCCATGCTAGAT 240

S4C12 GCAGCGCCAACAGCCCAATCAAACCCAGATGACCAATTTGACGAT**T**TTTCCATGCTAGAT 240

L6G1 GCAGCGCCA**G**CAGCCCAATCAAACCCAGATGACCAATTTGACGATCTTTCCATGCTAGAT 240

L6G2 GCAGCGCCAACAGCCCAATCAAACCCAGATGACCAATTTGACGATCTTTCCATGCTAGAT 240

S8H3 GCAGCGCCAACAGCCCAATCA**G**ACCCAGATGACCAATTTGACGATCTTTCCATGCTAGAT 240

S19E10 GCAGCGCCAACAGCCCAATCAAACCCAGATGACCAATTTGA**G**GAT**T**TTTCCATGCTAGAT 202

S21C5 GCAGCGCCAACAGCCCAATCAAACCCAGATGACCAATTTGA**G**GAT**T**TTTCCATGCTAGAT 240

G8A6 GCAGCGCCA**G**CAGCCCAATCAAACCCAGATGACCAATTTGACGATCTTTCCATGCTAGAT 240

G28H7 GCAGCGCCAACAGC**T**CAATCAAACCCAGATG**G**CCAATTTGACGATCTTTCCATGCTAGAT 240

L6B5 GCAGCGCCAACAGCCCAATCAAACCCAGATG**G**CCAATTTGACGATCTTTCCATGCTAGAT 240

L1H9 GCAGCGCCAACAGCCCAATCAAACCCAGATGACCAATTTGACGATCTTTCCATGCTAGAT 240

L6E2 GCAGCGCCAACAGCCCAATCAAACCCAGATGACCAATTTGACGATCTTTCCATGCTAGAT 240

G39G10 GCAGCGCCAACAGCCCAATCAAACCCAGATGACCAATTTGACGATCTTTCCATGCTAGAT 240

G27G6 GCAGCGCCAACAGCCCAATCAAACCCAGATGACCAATTTGACGATCTTTCCATGCTAGAT 240

L4E1 GCAGCGCCAACAGCCCAATCAAACCCAGATGACCAATTTGACGATCTTTCCATGCTAGAT 240

G8H8 GCAGCGCCAACAGCCCAATCAAACCCAGATGACCAATTTGACGATCTTTCCATGCTAGAT 240

G39B9 GGAGCGGCAACAGC**T**GAATC**TG**ACCCAGATGACCAATTTGACGATCTTTCCATGCTAGAT 239

L2C8 GCAGCGCCAACAGCCCAATCAAACCCAGATGACCAATTTGACG**G**TCTTTCCATGCTAGAT 240

S11D7 GCAGCGCCAACAGCCCAATCAAACCCAGATGACCAATTTGACGAT**T**TTTCCATG**T**TAGAT 202

L6D6 GCAGCGCCAACAGCCCAATCAAACCCAGATGACCAATTTGACGATCTTTCCATGCTAGAT 240

L9F12 GCAGCGCCAACAGCCCAATC**C**AACCCAGATGACCAATTTGACGATCTTTCCATGCTAGAT 240

G8B4 GCAGCGCCAACAGCCCAATCAAACCCAGATGACCAATTTGACGATCTTTCCATGCTAGAT 240

G3G5 GCAGCGCCAACAGC**T**CAATCAAACCCAGATGACCAATTTGACGATCTTTCCATGCTAGAT 240

G27E6 GCAGCGCCAACAGCCCAATCAAACCCAGATGACCAATTTGACGATCTTTCCATGCTAGAT 240

G5D4 GCAGCGCCAACAGCCCAATCAAACCCAGATGACCAATTTGACGATCTTTCCATGCTAGAT 240

G1A5 GCAGCGCCAACAGCCCAATC**C**AACCCAGATGACCAATTTGACGATCTTTCCATGCTAGAT 240

G6G2 GCAGCGCCAACAGCCCAATCAAACCCAGATGACCAATTTGACGATCTTTCCATGCTAGAT 240

G34G4 GCAGCGCCAACAGCCCAATC**C**AACCCAGATGACCAATTTGACGATCTTTCCATGCTAGAT 240

G38B12 GCAGCGCC**G**ACAGCCCAATCAAACCCAGATGACCAATTTGACGATCTTTCCATGCTAGAT 239

G8B10 GCAGCGCCAACAGCCCAATCAAACCCAGATGACCAATTTGACGATCTTTCCATGCTAGAT 240

G1H6 GCAGCGCCAACAGCCCAATCAAACCCAGATGACCAATTTGACGATCTTTCCATGCTAGAT 240

G36E3 GCAGCGCCAACAGCCCAATCAAACCCAGATGACCAATTTGACGATCTTTCCATGCTAGAT 240

G4H9 GCAGCGCCAACAGCCCAATCAAACCCAGATGACCAATTTGACGATCTTTCCATGCTAGAT 240

G33B10 GCAGCGCCAACAGCCCAATCAAACCCAGATGACCAATTTGACGATCTTTCCATGCTAGAT 240

L3A11 GCAGCGCCAACAGCCCAATCAAACCCAGATGACCAATTTGACGATCTTTCCATGCTAGAT 240

L6H9 GCAGCGCCAACAGCCCAATCAAACCCAGATGACCAATTTGACGATCTTTCCATGCTAGAT 240

L5E1 GCAGCGCCAACAGC**T**CAATCAAACCCAGATGACCAATTTGACGATCTTTCCATGCTAGAT 240

L3G10 GCAGCGCCAACAGCCCAATCAAACCCAGATGACCAATTTGACGATCTTTCCATGCTAGAT 240

L7D10 GCAGCGCCAACAGCCCAATC**C**AACCCAGATGACCAATTTGACGATCTTTCCATGCTAGAT 240

L7B7 GCAGCGCCAACAGCCCAATC**C**AACCCAGATGACCAATTTGACGATCTTTCCATGCTAGAT 240

L7G9 GCAGCGCCAACAGC**T**CAATCAAACCCAGATGACCAATTTGACGATCTTTCCATGCTAGAT 240

L2F3 GCAGCGCCAACAGC**T**CAATCAAACCCAGATGACCAATTTGACGATCTTTCCATGCTAGAT 240

L1F3 GCAGCGCCAACAGCCCAATCAAACCCAGATGACCAATTTGACGATCTTTCCATGCTAGAT 240

L1D3 GCAGCGCCAACAGCCCAATCAAACCCAGATGACCAATTTGACGATCTTTCCATGCTAGAT 240

L4D7 GCAGCGCCAACAGCCCAATCAAACCCAGATGACCAATTTGACGATCTTTCCATGCTAGAT 240

L10A5 GCAGCGCCAACAGC**T**CAATCAAACCCAGATGACCAATTTGACGATCTTTCCATGCTAGAT 240

L3D4 GCAGCGCCAACAGCCCAATCAAACCCAGATGACCAATTTGACGATCTTTCCATGCTAGAT 240

L8D6 GCAGCGCCAACAGCCCAATCAAACCCAGATGACCAATTTGACGATCTTTCCATGCTAGAT 240

S6F4 GCAGCGCCAACAGCCCAATCAAACCCAGATGACCAATTTGACGATCTTTCCATGCTAGAT 240

S19H4 GCAGCGCCAACAGCCCAATC**C**AACCCAGATGACCAATTTGACGATCTTTCCATGCTAGAT 240

S6C12 GCAGCGCCAACAGCCCAATCAAACCCAGATGACCAATTTGACGATCTTTCCATGCTAGAT 240

L1H10 GCAGCGCCAACAGCCCAATCAAACCCAGATGACCAATTTGACGATCTTTCCATGCTAGAT 240

L5G9 GCAGCGCCAACAGCCCAATCAAACCCAGATGACCAATTTGACGATCTTTCCATGCTAGAT 240

S8E10 GCAGCGCCAACAGCCCAATCAAACCCAGATGACCAATTTGACGAT**T**TTTCCATGCTAGAT 240

S18B4 GCAGCGCCAACAGCCCAATCAAACCCAGATGACCAATTTGACGAT**T**TTTCCATGCTAGAT 240

G34G2 GCAGCGCCAACAGCCCAATCAAACCCAGATGACCAATTTGACGATCTTTCCATGCTAGAT 240

G16C1 GCAGCGCCAACAGCCCAATCAAACCCAGATGACCAATTTGACGATCTTTCCATGCTAGAT 240

S18G4 GCAGCGCCAACAGCCCAATCAAACCCAGATGACCAATTTGACGATCTTTCCATGCTAGAT 240

S16F12 GCAGCGCCAACAGCCCAATCAAACCCAGATGACCAATTTGACGAT**T**TTTCCATGCTAGAT 240

G27D12 GCAGCGCCAACAGCCCAATCAAACCCAGATGACCAATTTGACGATCTTTCCATGCTAGAT 240

G2H6 GCAGCGCCAACAGCCCAATCAAACCCAGATGACCAATTTGACGATCTTTCCATGCTAGAT 240

L2F4 GCAGCGCCAACAGCCCAATC**C**AACCCAGATGACCAATTTGACGATCTTTCCATGCTAGAT 240

S21G8 GCAGCGCCAACAGCCCAATC**C**AACCCAGATGACCAATTTGACGATCTTTCCATGCTAGAT 240

G1C2 GCAGCGCCAACAGCCCAATCAAACCCAGATGACCAATTTGACGATCTTTCCATGCTAGAT 240

S16A9 GCAGCGCCAACAGCCCAATCAAACCCAGATGACCAATTTGACGAT**T**TTTCCATGCTAGAT 240

G5G10 GCAGCGCCAACAGCCCAATCAAACCCAGATGACCAATTTGACGATCTTTCCATGCTAGAT 240

L9F5 TGGGAAGCTATTCTAGCAGACGATTCAGATTTCGGAAATTCTAATGGACCTGCCACACCA 300

G38F8 TGGGAAGCTATTCTAGCAGACGATTCAGATTTCGGAAATTCTAATGGACCTGCCACACCA 300

S21E5 TGGGAAGCTATTCTAGCAGACGATTCAGATTTCGGAAATTCTAATGGACCTGCCACACCA 300

G16C5 TGGGAAGCTATT**TC**AGCAGACGATTCAGATTTCGGAAATTCTAATGGACCTGCCACACCA 300

S8C8 TGGGAAGCTATTCTAGCAGACGATTCAGATTTCGGAAATTCTAATGGACCTGCCACACCA 298

G16E1 TGGGAAGCTATTCTAGCAGACGATTCAGATTTCGGAAATTCTAATGGACCTGCCACACCA 300

S4C12 TGGGAAGCTATTCTAGCAGACGATTCAGATTTCGGAAATTCTAATGGACCTGCCACACCA 300

L6G1 TGGGA**G**GCTATTCTAGCAGACGATTCAGATTTCGGAAATTCTAATGGACCTGCCACACCA 300

L6G2 TGGGA**G**GCTATTCTAGCAGACGATTCAGATTTCGGAAATTCTAATGGACCTGCCACACCA 300

S8H3 TGGGAAGCTATTCTAGCAGACGATTCAGATTTCGGAAATTCTAATGGACCTGCCACACCA 300

S19E10 TGGGAAGCTATTCTAGCAGACGATTCAGATTTCGGAAATTCT**G**ATGGACCTGCC**G**CACCA 262

S21C5 TGGGAAGCTATTCTAGCAGACGATTCAGATTTCGGAAATTCTAATGGACCTGCCACACCA 300

G8A6 TGGGAAGCTATTCTAGCAGACGATTCAGATTTCGGAAATTCTAATGGACCTGCCACACCA 300

G28H7 TGGGAAGCTATTCTAGCAGACGATTCAGATTTCGGAAATTCTAATGGACCTGCCACACCA 300

L6B5 TGGGA**G**GCTATTCTAGCAGACGATTCAGATTTCGGAAATTCTAATGGACCT**A**CCACACCA 300

L1H9 TGGGAAGCTATT**T**TAGCAGACGATTCAGATTTCGGAAATTCTAATGGACCTGCCACACCA 300

L6E2 TGGGAAGCTATTCTAGCAGACGATTCAGATTTCGGAAATTCTAATGGACCTGCCACACCA 300

G39G10 TGGGAAGCTATTCTAGCAGACGATTCAGATTTCGGAAATTCTAATGGACCTGCCACACCA 300

G27G6 TGGGAAGCTATT**TC**AGCAGACGATTCAGATTTCGGAAATTCTAATGGACCTGCCACACCA 300

L4E1 TGGGAAGCTATT**TC**AGCAGACGATTCAGATTTCGGAAATTCTAATGGACCTGCCACACCA 300

G8H8 TGGGAA**T**CTATTCTAGCAGACGATTCAGATTTCGGAAATTCTAATGGACCTGCCACACCA 300

G39B9 TGGGAAGCTATTCTA**AA**AGACGATTCAGATTTCGGAAATTCTAATGGACCTGCCACACCA 299

L2C8 TGGGAAGCTATTCTAGCAGACGATTCAGATTTCGGAAATTCTAATGGACCTGCCACACCA 300

S11D7 TGGGAAGCTATTCTAGCAGACGATTCAGATTTCGGAAATTCTAATGGACCTGCCACACCA 262

L6D6 TGGGAAGCTATT**TC**AGCAGACGATTCAGATTTCGGAAATTCTAATGGACCTGCCACACCA 300

L9F12 TGGGAAGCTATT**TC**AGCAGACGATTCAGATTTCGGAAATTCTAATGGACCTGCCACACCA 300

G8B4 TGGGAAGCTATTCTAGCAGACGATTCAGATTTCGGAAATTCTAATGGACCTGCCACACCA 300

G3G5 TGGGAAGCTATTCTAGCAGACGATTCAGATTTCGGAAATTCTAATGGACCTGCCACACCA 300

G27E6 TGGGAAGCTATTCTAGCAGACGATTCAGATTTCGGAAATTCTAATGGACCTGCCACACCA 300

G5D4 TGGGAAGCTATTCTAGCAGACGATTCAGATTTCGGAAATTCTAATGGACCTGCCACACCA 300

G1A5 TGGGAAGCTATTCTAGCAGACGATTCAGATTTCGGAAATTCTAATGGACCTGCCACACCA 300

G6G2 TGGGAAGCTATTCTAGCAGACGATTCAGATTTCGGAAATTCTAATGGACCTGCCACACCA 300

G34G4 TGGGAAGCTATTCTAGCAGACGATTCAGATTTCGGAAATTCTAATGGACCTGCCACACCA 300

G38B12 TGGGAAGCTATTCTAGCAGACGATTCAGATTTCGGAAATTCTAATGGACCTGCCACACCA 299

G8B10 TGGGAAGCTATTCTAGCAGACGATTCAGATTTCGGAAATTCTAATGGACCTGCCACACCA 300

G1H6 TGGGA**G**GCTATTCTAGCAGACGATTCAGATTTCGGAAATTCTAATGGACCTGCCACACCA 300

G36E3 TGGGAAGCTATTCTAGCAGACGATTCAGATTTCGGAAATTCTAATGGACCTGCCACACCA 300

G4H9 TGGGAAGCTATTCTAGCAGACGATTCAGATTTCGGAAATTCTAATGGACCTGCCACACCA 300

G33B10 TGGGAAGCTATTCTAGCAGACGATTCAGATTTCGGAAATTCTAATGGACCTGCCACACCA 300

L3A11 TGGGAAGCTATT**T**TAGCAGACGATTCAGATTTCGGAAATTCTAATGGACCTGCCACACCA 300

L6H9 TGGGAAGCTATTCTAGCAGACGATTCAGATTTCGGAAATTCTAATGGACCTGCCACACCA 300

L5E1 TGGGAAGCTATTCTAGCAGACGATTCAGATTTCGGAAATTCTAATGGACCTGCCACACCA 300

L3G10 TGGGA**G**GCTATTCTAGCAGACGATTCAGATTTCGGAAATTCTAATGGACCTGCCACACCA 300

L7D10 TGGGAAGCTATTCTAGCAGACGATTCAGATTTCGGAAATTCTAATGGACCTGCCACACCA 300

L7B7 TGGGAAGCTATTCTAGCAGACGATTCAGATTTCGGAAATTCTAATGGACCTGCCACACCA 300

L7G9 TGGGA**G**GCTATTCTAGCAGACGATTCAGATTTCGGAAATTCTAATGGACCTGCCACACCA 300

L2F3 TGGGAAGCTATTCTAGCAGACGATTCAGATTTCGGAAATTCTAATGGACCTGCCACACCA 300

L1F3 TGGGA**G**GCTATTCTAGCAGACGATTCAGATTTCGGAAATTCTAATGGACCTGCCACACCA 300

L1D3 TGGGA**G**GCTATTCTAGCAGACGATTCAGATTTCGGAAATTCTAATGGACCTGCCACACCA 300

L4D7 TGGGAAGCTATTCTAGCAGACGATTCAGATTTCGGAAATTCTAATGGACCTGCCACACCA 300

L10A5 TGGGAAGCTATTCTAGCAGACGATTCAGATTTCGGAAATTCTAATGGACCTGCCACACCA 300

L3D4 TGGGA**G**GCTATTCTAGCAGACGATTCAGATTTCGGAAATTCTAATGGACCTGCCACACCA 300

L8D6 TGGGA**G**GCTATTCTAGCAGACGATTCAGATTTCGGAAATTCTAATGGACCTGCCACACCA 300

S6F4 TGGGAAGCTATTCTAGCAGACGATTCAGATTTCGGAAATTCTAATGGACCTGCCACACCA 300

S19H4 TGGGAAGCTATTCTAGCAGACGATTCAGATTTCGGAAATTCTAATGGACCTGCCACACCA 300

S6C12 TGGGAAGCTATTCTAGCAGACGATTCAGATTTCGGAAATTCTAATGGACCTGCCACACCA 300

L1H10 TGGGAAGCTATTCTAGCAGACGATTCAGATTTCGGAAATTCTAATGGACCTGCCACACCA 300

L5G9 TGGGAAGCTATTCTAGCAGACGATTCAGATTTCGGAAATTCTAATGGACCTGCCACACCA 300

S8E10 TGGGAAGCTATTCTAGCAGACGATTCAGATTTCGGAAATTCTAATGGACCTGCCACACCA 300

S18B4 TGGGAAGCTATTCTAGCAGACGATTCAGATTTCGGAAATTCTAATGGACCTGCCACACCA 300

G34G2 TGGGAAGCTATT**TC**AGCAGACGATTCAGATTTCGGAAATTCTAATGGACCTGCCACACCA 300

G16C1 TGGGAAGCTATTCTAGCAGACGATTCAGATTTCGGAAATTCTAATGGACCTGCCACACCA 300

S18G4 TGGGAAGCTATTCTAGCAGACGATTCAGATTTCGGAAATTCTAATGGACCTGCCACACCA 300

S16F12 TGGGAAGCTATTCTAGCAGACGATTCAGATTTCGGAAATTCTAATGGACCTGCCACACCA 300

G27D12 TGGGAAGCTATT**TC**AGCAGACGATTCAGATTTCGGAAATTCTAATGGACCTGCCACACCA 300

G2H6 TGGGA**G**GCTATTCTAGCAGACGATTCAGATTTCGGAAATTCTAATGGACCTGCCACACCA 300

L2F4 TGGGAAGCTATTCTAGCAGACGATTCAGATTTCGGAAATTCTAATGGACCTGCCACACCA 300

S21G8 TGGGAAGCTATTCTAGCAGACGATTCAGATTTCGGAAATTCTAATGGACCTGCCACACCA 300

G1C2 TGGGAAGCTATTCTAGCAGACGATTCAGATTTCGGAAATTCTAATGGACCTGCCACACCA 300

S16A9 TGGGAAGCTATTCTAGCAGACGATTCAGATTTCGGAAATTCTAATGGACCTGCCACACCA 300

G5G10 TGGGAAGCTATT**TC**AGCAGACGATTCAGATTTCGGAAATTCTAATGGACCTGCCACACCA 300

L9F5 GCAGCTGCACCAGCTAAACCATCAAAAGGAAAACCAAAGAAGGCCGATT---------CA 351

G38F8 GCAGCTGCACCAGCTAAACCATCAAAAGGAAAACCAAAGAAGGCCGATTCACCAAAATCA 360

S21E5 GCAGCTGCACCAGCTAAACCATCAAA**G**G**C**AAAACCAAAGAAGGCCGATT---------CA 351

G16C5 GCAGCTGCACCAGCTAAACCATCAAAAGGAAAACCAAAGAAGGCCGATT---------CA 351

S8C8 GCAGCTGCACCAGCTAAACCATCAAA**G**G**C**AAAACCAAAGAAGGCCGATT---------CA 349

G16E1 GCAGCTGCACCAGCTAAACCATCAAAAGGAAAACCAAAGAAGGCCGATT---------CA 351

S4C12 GCAGCTGCACCAGCTAAACCATCAAA**G**G**C**AAAACCAAAGAAGGCCGATT---------CA 351

L6G1 GCAGCTGCACCAGCTAAACCATCAAAAGGAAAACCAAAGAAGGCCGATT---------CA 351

L6G2 GCAGCTGCACCAGCTAAACCATCAAAAGGAAAACCAAAGAAGGCCGATT---------CA 351

S8H3 GCAGCTGCACCAGCTAAACCATCAAA**G**G**C**AAAACCAAAGAAGGCCGATT---------CA 351

S19E10 GCAGCTGCACCAGCTAAACCATCAAA**G**G**C**AAAACCAAAGAAGGCCGATT---------CA 351

S21C5 GCAGCTGCACCAGCTAAACCATCAAA**G**G**C**AAAACCAAAGAAGGCC**A**ATT---------CA 313

G8A6 GCAGCTGCACCAGCTAAACCATCAAAAGGAAAACCAAAGAAGGCCGATT---------CA 351

G28H7 GCAGCTGCACCAGCTAAACCATCAAAAGGAAAACCAAAGAAGGCCGATT---------CA 351

L6B5 GCAGCTGCACCAGCTAAACCATCAAAAGGAAAACCAAAGAAGGCCGATT---------CA 351

L1H9 GCAGCTGCACCAGCTAAACCATCAAAAGGAAAACCAAAGAAGGCCGATT---------CA 351

L6E2 GCAGCTGCACCAGCTAAACCATCAAAAGGAAAACCAAAGAAGGCCGATT---------CA 351

G39G10 GCAGCTGCACCAGCTAAACCATCAAAAGGAAAACCAAAGAAGGCCGATTCAC**T**AAAATCA 360

G27G6 GCAGCTGCACCAGCTAAACCATCAAAAGGAAAACCAAAGAAGGCCGATT---------CA 351

L4E1 GCAGCTGCACCAGCT**G**AACCATCAAAAGGAAAACCAAAGAAGGCCGATT---------CA 351

G8H8 GCAGCTGCACCAGCTAAACCA**A**C**T**AAA**AT**AAA---A**TAG**AA------TT--------ACA 343

G39B9 GC**T**GCTGCACCAGCTAAACCATC**T**GAAGGAAAACCAAAGAA**T**GCCCATT---------TA 350

L2C8 GCAGCTGCACCAGCTAAACCATCAAAAGGAAAACCAAAGAAGGCCGATTCACCAAAATCA 360

S11D7 GCAGCTGCACCAGCTAAACCATCAAA**G**G**C**AAAACCAAAGAAGGCCGATT---------CA 313

L6D6 GCAGCTGCACCAGCTAAACCATCAAAAGGAAAACCAAAGAAGGCCGATT---------CA 351

L9F12 GCAGCTGCACCAGCTAAACCATCAAAAGGAAAACCAAAGAAGGCCGATT---------CA 351

G8B4 GCAGCTGCACCAGCTAAACCATCAAAAGGAAAACCAAAGAAGGCCGATT---------CA 351

G3G5 GCAGCTGCACCAGCTAAACCATCAAAAGGAAAACCAAAGAAGGCCGATT---------CA 351

G27E6 GCAGCTGCACCAGCTAAACCATCAAAAGGAAAACCAAAGAAGGCCGATT---------CA 351

G5D4 GCAGCTGCACCAGCTAAACCATCAAAAGGAAAACCAAAGAAGGCCGATT---------CA 351

G1A5 GCAGCTGCACCAGCTAAACCATCAAAAGGAAAACCAAAGAAGGCCGATT---------CA 351

G6G2 GCAGCTGCACCAGCTAAACCATCAAAAGGAAAACCAAAGAAGGCCGATT---------CA 351

G34G4 GCAGCTGCACCAGCTAAACCATCAAAAGGAAAACCAAAGAAGGCCGATT---------CA 351

G38B12 GCAGCTGCACCAGCTAAACCATCAAAAGGAAAACCAAAGAAGGCCGATT---------CA 350

G8B10 GCAGCTGCACCAGCTAAACCATCAAAAGGAAAACCAAAGAAGGCCGATT---------CA 351

G1H6 GCAGCTGCACCAGCTAAACCATCAAAAGGAAAACCAAAGAAGGCCGATT---------CA 351

G36E3 GCAGCTGCACCAGCTAAACCATCAAAAGGAAAACCAAAGAAGGCCGATT---------CA 351

G4H9 GCAGCTGCACCAGCTAAACCATCAAAAGGAAAACCAAAGAAGGCCGATT---------CA 351

G33B10 GCAGCTGCACCAGCTAAACC**G**TCAAAAGGAAA**G**CCAAAGAAGGCCGATT---------CA 351

L3A11 GCAGCTGCACCAGCTAAACCATCAAAAGGAAAACCAAAGAAGGCCGATT---------CA 351

L6H9 GCAGCTGCACCAGCTAAACCATCAAAAGGAAAACCAAAGAAGGCCGATT---------CA 351

L5E1 GCAGCTGCACCAGCTAAACCATCAAAAGGAAAACCAAAGAAGGCCGATT---------CA 351

L3G10 GCAGCTGCACCAGCTAAACCATCAAAAGGAAAACCAAAGAAGGCCGATT---------CA 351

L7D10 GCAGCTGCACCAGCTAAACCATCAAAAGGAAAACCAAAGAAGGCCGATT---------CA 351

L7B7 GCAGCTGCACCAGCTAAACCATCAAAAGGAAAACCAAAGAAGGCCGATT---------CA 351

L7G9 GCAGCTGCACCAGCTAAACCATCAAAAGGAAAACCAAAGAAGGCCGATT---------CA 351

L2F3 GCAGCTGCACCAGCTAAACCATCAAAAGGAAAACCAAAGAAGGCCGATT---------CA 351

L1F3 GCAGCTGCACCAGCTAAACCATCAAAAGGAAAACCAAAGAAGGCCGATT---------CA 351

L1D3 GCAGCTGCACCAGCTAAACCATCAAAAGGAAAACCAAAGAAGGCCGATT---------CA 351

L4D7 GCAGCTGCACCAGCTAAACCATCAAAAGGAAAACCAAAGAAGGCCGATTCACCAAAATCA 360

L10A5 GCAGCTGCACCAGCTAAACCATCAAAAGGAAAACCAAAGAAGGCCGATTCACCAAAATCA 360

L3D4 GCAGCTGCACCAGCTAAACCATCAAAAGGAAAACCAAAGAAGGCCGATT---------CA 351

L8D6 GCAGCTGCACCAGCTAAACCATCAAAAGGAAAACCAAAGAAGGCCGATT---------CA 351

S6F4 GCAGCTGCACCAGCTAAACCATCAAAAGGAAAACCAAAGAAGGCCGATT---------CA 351

S19H4 GCAGCTGCACCAGCTAAACCATCAAAAGGAAAACCAAAGAAGGCCGATT---------CA 351

S6C12 GCAGCTGCACCAGCTAAACCATCAAAAGGAAAACCAAAGAAGGCCGATT---------CA 351

L1H10 GCAGCTGCACCAGCTAAACCATCAAAAGGAAAACCAAAGAAGGCCGATTCACCAAAATCA 360

L5G9 GCAGCTGCACCAGCTAAACCATCAAAAGGAAAACCAAAGAAGGCCGATTCACCAAAATCA 360

S8E10 GCAGCTGCACCAGCTAAACCATCAAA**G**G**C**AAAACCAAAGAAGGCCGATT---------CA 351

S18B4 GCAGCTGCACCAGCTAAACCATCAAA**G**G**C**AAAACCAAAGAAGGCCGATT---------CA 351

G34G2 GCAGCTGCACCAGCTAAACCATCAAAAGGAAAACCAAAGAAGGCCGATT---------CA 351

G16C1 GCAGCTGCACCAGCTAAACCATCAAAAGGAAAACCAAAGAAGGCCGATT---------CA 351

S18G4 GCAGCTGCACCAGCTAAACCATCAAA**G**G**C**AAAACCAAAGAAGGCCGATT---------CA 351

S16F12 GCAGCTGCACCAGCTAAACCATCAAA**G**G**C**AAAACCAAAGAAGGCCGATT---------CA 351

G27D12 GCAGCTGCACCAGCTAAACCATCAAAAGGAAAACCAAAGAAGGCCGATT---------CA 351

G2H6 GCAGCTGCACCAGCTAAACCATCAAAAGGAAAACCAAAGAAGGCCGATT---------CA 351

L2F4 GCAGCTGCACCAGCTAAACCATCAAAAGGAAAACCAAAGAAGGCCGATT---------CA 351

S21G8 GCAGCTGCACCAGCTAAACCATCAAAAGGAAAACCAAAGAAGGCCGATT---------CA 351

G1C2 GCAGCTGCACCAGCTAAACCATCAAAAGGAAAACCAAAGAAGGCCGATTCACCAAAATCA 360

S16A9 GCAGCTGCACCAGCTAAACCATCAAA**G**G**C**AAAACCAAAGAAGGCCGATT---------CA 351

G5G10 GCAGCTGCACCAGCTAAACCATCAAAAGGAAAACCAAAGAAGGCCGATT---------CA 351

L9F5 CCAAAATCACCAAAA------------------CCAGCCCCAAAAAAAGCAGCCTCAAAG 393

G38F8 CCAAAATCACCAAAA------------------CCAGCCCCAAAAAAAGCAGCCTCAAAG 402

S21E5 CCAAAATCACCAAAA------------------CCAGCCCCAAAAAAAGCAGCCTCAAAG 393

G16C5 CCAAAATCACCAAAA------------------CCAGCCCCAAAAAAAGCAGCCTCAAAG 393

S8C8 CCAAAATCACCAAAA------------------CCAGCCCCAAAAAAAGCAGCCTCAAAG 391

G16E1 CCAAAATCACCAAAA------------------CCAGCCCCAAAAAAAGCAGCCTCAAAG 393

S4C12 CCAAAATCACCAAAA------------------CCAGCCCCAAAAAAAGCAGCCTCAAAG 393

L6G1 CCAAAATCACCAAAA------------------CCAGCCCCAAAAAAAGCAGCCTCAAAG 393

L6G2 CCAAAATCACCAAAA------------------CCAGCCCCAAAAAAAGCAGCCTCAAAG 393

S8H3 CCAAAATCACCAAAA------------------CCAGCCCCAAAAAAAGCAGCCTCAAAG 393

S19E10 CCAAAATCACCAAAA------------------CCAGCCCCAAAAAAAGCAGCCTCAAAG 355

S21C5 CCAAAATCACCAAAA------------------CCAGCCCCAAAAAAAGCAGC**TC**CAAAG 393

G8A6 CCAAAATCACCAAAA------------------CCAGCCCCAAAAAAAGCAGCCTCAAAG 393

G28H7 CCAAAATCACCAAAA------------------CCAGCCCCAAAAAAAGCAGCCTCAAAG 393

L6B5 CCAAAATCACCAAAA------------------CCAGCCCCAAAAAAAGCAGCCTCAAAG 393

L1H9 CCAAAATCACCAAAATCACCAAAATCACTAAAACCAGCCCCAAAAAAAGCAGCCTCAAAG 411

L6E2 CCAAAATCACCAAAA------------------CCAGCCCCAAAAAAAGCAGCCTCAAAG 393

G39G10 CCAAAATCACCAAAA------------------CCAGCCCCAAAAAAA**A**CAGCCTCAAAG 402

G27G6 CCAAAATCACCAAAA------------------CCAGCCCCAAAAAAAGCAGCCTCAAAG 393

L4E1 CCAAAATCACCAAAA------------------CCAGCCCCAAAAAAAGCAGCCTCAAAG 393

G8H8 -AAAAATCCCTGTTGGC------------------------------------------- 359

G39B9 CCAAAATCACCAAAA------------------CCAGCCCCAAAAAAAGCAGCCTCAAAG 392

L2C8 CCAAAATCACCAAAA------------------CCAGCCCCAAAAAAAGCAGCCTCAAAG 402

S11D7 CCAAAATCACCAAAA------------------CCAGCCCCAAAAAAAGCAGCCTCAAAG 355

L6D6 CCAAAATCACCAAAA------------------CCAGCCCCAAAAAAAGCAGCCTCAAAG 393

L9F12 CCAAAATCACCAAAA------------------CCAGCCCCAAAAAAAGCAGCCTCAAAG 393

G8B4 CCAAAATCACCAAAA------------------CCAGCCCCAAAAAAAGCAGCCTCAAAG 393

G3G5 CCAAAATCACCAAAA------------------CCAGCCCCAAAAAAAGCAGCCTCAAAG 393

G27E6 CCAAAATCACCAAAA------------------CCAGCCCCAAAAAAAGCAGCCTCAAAG 393

G5D4 CCAAAATCACCAAAA------------------CCAGCCCCAAAAAAAGCAGCCTCAAAG 393

G1A5 CCAAAATCACCAAAA------------------CCAGCCCCAAAAAAAGCAGCCTCAAAG 393

G6G2 CCAAAATCACCAAAA------------------CCAGCCCCAAAAAAAGCAGCCTCAAAG 393

G34G4 CCAAAATCACCAAAA------------------CCAGCCCCAAAAAAAGCAGCCTCAAAG 393

G38B12 CCAAAATCACCAAAA------------------CCAGCCCCAAAAAAAGCAGCCTCAAAG 392

G8B10 CCAAAATCACCAAAA------------------CCAGCCCCAAAAAAAGCAGCCTCAAAG 393

G1H6 CCAAAATCACCAAAA------------------CCAGCCCCAAAAAAAGCAGCCTCAAAG 393

G36E3 CCAAAATCACCAAAA------------------CCAGCCCCAAAAAAAGCAGCCTCAAAG 393

G4H9 CCAAAATCACCAAAA------------------CCAGCCCCAAAAAAAGCAGCCTCAAAG 393

G33B10 CCAAAATCACCAAAA------------------CCAGCCCCAAAAAAAGCAGCCTCAAAG 393

L3A11 CCAAAATCACCAAAA------------------CCAGCCCCAAAAAAAGCAGCCTCAAAG 393

L6H9 CCAAAATCACCAAAA------------------CCAGCCCCAAAAAAAGCAGCCTCAAAG 393

L5E1 CCAAAATCACCAAAA------------------CCAGCCCCAAAAAAAGCAGCCTCAAAG 393

L3G10 CCAAAATCACCAAAA------------------CCAGCCCCAAAAAAAGCAGCCTCAAAG 393

L7D10 CCAAAATCACCAAAA------------------CCAGCCCCAAAAAAAGCAGCCTCAAAG 393

L7B7 CCAAAATCACCAAAA------------------CCAGCCCCAAAAAAAGCAGCCTCAAAG 393

L7G9 CCAAAATCACCAAAA------------------CCAGCCCCAAAAAAAGCAGCCTCAAAG 393

L2F3 CCAAAATCACCAAAA------------------CCAGCCCCAAAAAAAGCAGCCTCAAAG 393

L1F3 CCAAAATCACCAAAA------------------CCAGCCCCAAAAAAAGCAGCCTCAAAG 393

L1D3 CCAAAATCACCAAAA------------------CCAGCCCCAAAAAAAGCAGCCTCAAAG 393

L4D7 CCAAAATCACCAAAA------------------CCAGCCCCAAAAAAAGCAGCCTCAAAG 402

L10A5 CCAAAA**C**CA---------------------------GCCCCAAAAAAAGCAGCCTCAAAG 393

L3D4 CCAAAATCACCAAAA------------------CCAGCCCCAAAAAAAGCAGCCTCAAAG 393

L8D6 CCAAAATCACCAAAA------------------CCAGCCCCAAAAAAAGCAGCCTCAAAG 393

S6F4 CCAAAATCACCAAAA------------------CCAGCCCCAAAAAAAGCAGCCTCAAAG 393

S19H4 CCAAAATCACCAAAA------------------CCAGCCCCAAAAAAAGCAGCCTCAAAG 393

S6C12 CCAAAATCACCAAAA------------------CCAGCCCCAAAAAAAGCAGCCTCAAAG 393

L1H10 CCAAAATCACCAAAA------------------CCAGCCCCAAAAAAAGCAGCCTCAAAG 402

L5G9 CCAAAATCACCAAAA------------------CCAGCCCCAAAAAAAGCAGCCTCAAAG 402

S8E10 CCAAAATCACCAAAA------------------CCAGCCCCAAAAAAAGCAGCCTCAAAG 393

S18B4 CCAAAATCACCAAAA------------------CCAGCCCCAAAAAAAGCAGCCTCAAAG 393

G34G2 CCAAAATCACCAAAA------------------CCAGCCCCAAAAAAAGCAGCCTCAAAG 393

G16C1 CCAAAATCACCAAAA------------------CCAGCCCCAAAAAAAGCAGCCTCAAAG 393

S18G4 CCAAAATCACCAAAA------------------CCAGCCCCAAAAAAAGCAGCCTCAAAG 393

S16F12 CCAAAATCACCAAAA------------------CCAGCCCCAAAAAAAGCAGCCTCAAAG 393

G27D12 CCAAAATCACCAAAA------------------CCAGCCCCAAAAAAAGCAGCCTCAAAG 393

G2H6 CCAAAATCACCAAAA------------------CCAGCCCCAAAAAAAGCAGCCTCAAAG 393

L2F4 CCAAAATCACCAAAA------------------CCAGCCCCAAAAAAAGCAGCCTCAAAG 393

S21G8 CCAAAATCACCAAAA------------------CCAGCCCCAAAAAAAGCAGCCTCAAAG 393

G1C2 CCAAAATCACCAAAA------------------CCAGCCCCAAAAAAAGCAGCCTCAAAG 402

S16A9 CCAAAATCACCAAAA------------------CCAGCCCCAAAAAAAGCAGCCTCAAAG 393

G5G10 CCAAAATCACCAAAA------------------CCAGCCCCAAAAAAAGCAGCCTCAAAG 393

L9F5 AAGAAG**TGA**TCATTTCATTCAATTGAAAGAACATTCGGAGACG**A**CGTGTAATCAAAATT**G** 453

G38F8 AAGAAG**TAA**T**G**ATTTCATTCAATTGAAAGAACATTCGGAGACGTCGTGTAATCAAAATT**G** 462

S21E5 AAGAAG**TGA**TCATTTCATTCAATTGAAAGAACATTCGGAGACGTCGTGTAATCAAAATTA 453

G16C5 AAGAAG**TAA**T**G**ATTTCATTCAATTGAAAGAACATTCGGAGGCGTCGTGTAATCAAAATTA 453

S8C8 AAGAAG**TGA**TCATTTCATTCAATTGAAAGAACATTCGGAGACGTCGTGTAATCAAAATTA 452

G16E1 AAGAAG**TAA**T**G**ATTTCATTCAATTGAAAGAACATTCGGAGGCGTCCTGTAATCAAAATTA 453

S4C12 AAGAAG**TGA**TCATTTCATTCAATTGAAAGAACATTCGGAGACGTCGTGTAATCAAAATTA 453

L6G1 AAGAAG**TGA**TCATTTCATTCAATTGAAAGAACATTCGGAGACGTCGTGTAATCAAAATTA 453

L6G2 AAGAAG**TGA**TCATTTCATTCAATTGAAAGAACATTCGGAGACG**A**CGTGTAATCAAAATT**G** 453

S8H3 AAGAAG**TGA**TCATTTCATTCAATTGAAAGAACATTCGGAGACGTCGTGTAATCAAAATTA 453

S19E10 AAGAAG**TGA**TCATTTCATTCAATTGAAAGAACATTCGGAGACGTCGTGTAATCAAAATTA 415

S21C5 AAGAAG**TGA**TCATTTCATTCAATTGAAAGAACATTCGGAGACGTCGTGTAATCAAAATTA 453

G8A6 AAGAAG**TAA**T**G**ATTTCATTCAATTGAAAGAACATTCGGAGGCGTCGTGTAATCAAAATTA 453

G28H7 AAGAAG**TGA**TCATTTCATTCAATTGAAAGAACATTCGGAGACG**A**CGTGTAATCAAAATT**G** 453

L6B5 AAGAAG**TGA**TCATTTCATTCAATTGAAAGAACATTCGGAGACG**A**CGTGTAATCAAAATT**G** 453

L1H9 AAGAAG**TGA**TCATTTCATTCAATTGAAAGAACATTCGGAGACGTCGTGTAATCAAAATTA 471

L6E2 AAGAAG**TGA**TCATTTCATTCAATTGAAAGAACATTCGGAGACGTCGTGTAATCAAAATTA 453

G39G10 AAGAAG**TGA**TCATTTCATTCAATTGAAAGAACATTCGGAGACGTCGTGTAATCAAAATTA 462

G27G6 AAGAAG**TAA**T**G**ATTTCATTCAATTGAAAGAACATTCGGAGGCGTCGTGTAATCAAAATTA 453

L4E1 AAGAAG**TAA**T**G**ATTTCATTCAATTGAAAGAACATTCGGAGGCGTCGTGTAATCAAAATTA 453

G8H8 ------------------------------------------------------------ 360

G39B9 AAGAAG**TGA**TCATTTCATTCAATTGAAAGAACATTCGGAGACG**A**CGTGAAATCAAAATT**G** 452

L2C8 AAGAAG**TAA**T**G**ATTTCATTCAATTGAAAGAACATTCGGAGACGTCGTGTAATCAAAATT**G** 462

S11D7 AAGAAG**TGA**TCATTTCATTCAATTGAAAGAACATTCGGAGACGTCGTGTAATCAAAATTA 415

L6D6 AAGAAG**TAA**T**G**ATTTCATTCAATTGAAAGAACATTCGGAG**G**CGTCGTGTAATCAAAATTA 453

L9F12 AAGAAG**TAA**T**G**ATTTCATTCAATTGAAAGAACATTCGGAG**G**CGTCGTGTAATCAAAATTA 453

G8B4 AAGAAG**TAA**T**G**ATTTCATTCAATTGAAAGAACATTCGGAG**G**CGTCGTGTAATCAAAATTA 453

G3G5 AAGAAG**TGA**TCATTTCATTCAATTGAAAGAACATTCGGAGAC------------------ 435

G27E6 AAGAAG**TGA**TCATTTCATTCAATTGAAAGAACATTCGGAG**G**CGTCGTGTAATCAAAATTA 453

G5D4 AAGAAG**TAA**T**G**ATTTCATTC**G**ATTGAAAGAACATTCGGAGACGTCGTGTAATCAAAATTA 453

G1A5 AAGAAG**TGA**TCATTTCATTCAATTGAAAGAACATTCGGAGACGTCGTGTAATCAAAATTA 453

G6G2 AAGAAG**TGA**TCATTTCATTCAATTGAAAGAACATTCGGAG**G**CGTCGTGTAATCAAAATTA 453

G34G4 AAGAAG**TAA**T**G**ATTTCATTCAATTGAAAGAACATTCGGAGACGTCGTGTAATCAAAATTA 453

G38B12 AAGAAG**TGA**TCATTTCATTCAATTGAAAGAACATTCGGAG**G**CGTCGTGTAATCAAAATTA 452

G8B10 AAGAAG**TAA**T**G**ATTTCATTCAATTGAAAGAACATTCGGAG**G**CGTCGTGTAATCAAAATTA 453

G1H6 AAGAAG**TAA**T**G**ATTTCATTCAATTGAAAGAACATTCGGAGACGTCGTGTAATCAAAATTA 453

G36E3 AAGAAG**TGA**TCATTTCATTCAATTGAAAGAACATTCGGAG**G**CGTCGTGTAATCAAAATTA 453

G4H9 AAGAAG**TAA**T**G**ATTTCATTCAATTGAAAGAACATTCGGAG**G**CGTCG-------------- 439

G33B10 AAGAAG**TAA**T**G**ATTTCATTCAATTGAAAGAACATTCGGAG**G**CGTCGTGTAATCAAAATTA 453

L3A11 AAGAAG**TAA**T**G**ATTTCATTCAATTGAAAGAACATTCGGAGACGTCGTGTAATCAAAATTA 453

L6H9 AAGAAG**TGA**TCATTTCATTCAATTGAAAGAACATTCGGAGACGTCGTGTAATCAAAATTA 453

L5E1 AAGAAG**TGA**TCATTTCATTCAATTGAAAGAACATTCGGAGACG**A**CGTGTAATCAAAATT**G** 453

L3G10 AAGAAG**TGA**TCATTTCATTCAATTGAAAGAACATTCGGAGACGTCGTGTAATCAAAATTA 453

L7D10 AAGAAG**TGA**TCATTTCATTCAATTGAAAGAACATTCGGAGACGTCGTGTAATCAAAATTA 453

L7B7 AAGAAG**TGA**TCATTTCATTCAATTGAAAGAACATTCGGAGACGTCGTGTAATCAAAATTA 453

L7G9 AAGAAG**TGA**TCATTTCATTCAATTGAAAGAACATTCGGAGACGTCGTGTAATCAAAATTA 453

L2F3 AAGAAG**TGA**TCATTTCATTCAATTGAAAGAACATTCGGAG**G**CG**A**CGTGTAATCAAAATT**G** 453

L1F3 AAGAAG**TGA**TCATTTCATTCAATTGAAAGAACATTCGGAGACG**A**CGTGTAATCAAAATT**G** 453

L1D3 AAGAAG**TGA**TCATTTCATTCAATTGAAAGAACATTCGGAGACGTCGTGTAATCAAAATTA 453

L4D7 AAGAAG**TGA**TCATTTCATTCAATTGAAAGAACATTCGGAGACGTCGTGTAATCAAAATTA 462

L10A5 AAGAAG**TGA**TCATTTCATTCAATTGAAAGAACATTCGGAGACGACGTGTAATCAAAATT**G** 453

L3D4 AAGAAG**TGA**TCATTTCATTCAATTGAAAGAACATTCGGAG**G**CGTCGTGTAATCAAAATTA 453

L8D6 AAGAAG**TAA**T**G**ATTTCATTCAATTGAAAGAACATTCGGAG**G**CGTCGTGTAATCAAAATTA 453

S6F4 AAGAAG**TAA**T**G**ATTTCATTCAATTGAAAGAACATTCGGAGACGTCGTGTAATCAAAATTA 453

S19H4 AAGAAG**TGA**TCATTTCATTC**G**ATTGAAAGAACATTCGGAGACGTCGTGTAATCAAAATTA 453

S6C12 AAGAAG**TGA**TCATTTCATTCAATTGAAAGAACATTCGGAGACGTCGTGTAATCAAAATTA 453

L1H10 AAGAAG**TAA**T**G**ATTTCATTCAATTGAAAGAACATTCGGAGACGTCGTGTAATCAAAATTA 462

L5G9 AAGAAG**TGA**TCATTTCATTCAATTGAAAGAACATTCGGAGACGTCGTGTAATCAAAATTA 462

S8E10 AAGAAG**TGA**TCATTTCATTCAATTGAAAGAACATTCGGAGACGTCGTGTAATCAAAATTA 453

S18B4 AAGAAG**TGA**TCATTTCATTCAATTGAAAGAACATTCGGAGACGTCGTGTAATCAAAATTA 453

G34G2 AAGAAG**TGA**TCATTTCATTCAATTGAAAGAACATTCGGAG**G**CGTCGTGTAATCAAAATTA 453

G16C1 AAGAAG**TAA**T**G**ATTTCATTCAATTGAAAGAACATTCGGAG**G**CGTCGTGTAATCAAAATTA 453

S18G4 AAGAAG**TGA**TCATTTCATTCAATTGAAAGAACATTCGGAGACGTCGTGTAATCAAAATTA 453

S16F12 AAGAAG**TGA**TCATTTCATTCAATTGAAAGAACATTCGGAGACGTCGTGTAATCAAAATTA 453

G27D12 AAGAAG**TAA**T**G**ATTTCATTCAATTGAAAGAACATTCGGAG**G**CGTCGTGTAATCAAAATTA 453

G2H6 AAGAAG**TGA**TCATTTCATTCAATTGAAAGAACATTCGGAGACGTCGTGTAATCAAAATTA 453

L2F4 AAGAAG**TGA**TCATTTCATTCAATTGAAAGAACATTCGGAGACGTCGTGTAATCAAAATTA 453

S21G8 AAGAAG**TGA**TCATTTCATTC**G**ATTGAAAGAACATTCGGAGACGTCGTGTAATCAAAATTA 453

G1C2 AAGAAG**TAA**T**G**ATTTCATTCAATTGAAAGAACATTCGGAGACGTCGTGTAATCAAAATT**G** 462

S16A9 AAGAAG**TGA**TCATTTCATTCAATTGAAAGAACATTCGGAGACGTCGTGTAATCAAAATTA 453

G5G10 AAGAAG**TAA**T**G**ATTTCATTCAATTGAAAGAACATTCGGAG**G**CGTCGTGTAATCAAAATTA 453

L9F5 AATAGTTATACGCTCTCATATTTTCAATTTGTCCATTGAAATCAATTCTTGAGATTAAAT 513

G38F8 AATAGTTATACGCTCT**T**ATATTTTCAATTTGTC**A**ATTGAAATCAATTCTTGAGATTAAAT 522

S21E5 AATAGTTATACGCTCTCATATTTTCAATTTGTC**A**ATTGAAATCAATTCTTGAGATTAAAT 513

G16C5 AAT**T**GTTATACGCTCTCATATTTTCAATTTGTCCATTGAAA**C**CATT**C**CTTGAGATTAAAT 513

S8C8 AATAGTTATAC**A**CTCTCATATTTTCAATTTGTC**A**ATTGAAATCAATTCTTGAGATTAAAT 512

G16E1 AAT**T**GTTATACGCTCT**G**ATATTTTCAATTTG**C**CCATTGAAATCAATTCTTGAGATTAAAT 513

S4C12 AATAGTTATACGCTCTCATATTTTCAATTTGTC**A**ATTGAAATCAATTCTTGAGATTAAAT 513

L6G1 AATAGTTATACGCTCTCATATTTTCAATTTGTC**T**ATTGAAATCAATTCTTGAGATTAAAT 513

L6G2 AATAGTTATACGCTCTCATATTTTCAATTTGTCCATTGAAATCAATTCTTGAGATTAAAT 513

S8H3 AATAGTTATAC**A**CTCTCATATTTTCAATTTGTC**A**ATTGAAATCAATTCTTGAGATTAAAT 513

S19E10 AATAGTTATAC**A**CTCTCATATTTTCAATTTGTC**A**ATTGAAATCAATTCTTGAGATTAAAT 475

S21C5 AATAGTTATACGCTCTCATATTTTCAATTTGTC**T**ATTGAAATCAATTCTTGAGATTAAAT 513

G8A6 AAT**T**GTTATACGCTCT**G**ATATTTTCAATTTGTCCATTGAAATCAATTCTTGAGATTAAAT 513

G28H7 AATAGTTATACGCTCTCATATTTTCAATTTGTCCATTGAAATCAATTCTTGAGATTAAAT 513

L6B5 AATAGTTATACGCTCTCATATTTTCAATTTGTCCATTGAAATCAATTCTTGAGATTAAAT 513

L1H9 AATAGTTATACGCTCT**T**ATATTTTCAATTTGTC**A**ATTGAAATCAATTCTTGAGATTAAAT 531

L6E2 AAT**T**GTTATACGCTCTCATATTTTCAATTTGTC**T**ATTGAAATCAATTCTTGAGATTAAAT 513

G39G10 AAT**T**GTTATACGCTCTCATATTTTCAATTTGTC**T**ATTGAAATCAATTCTTGAGATTAAAT 522

G27G6 AAT**T**GTTATACGCTCTCATATTTTCAATTTGTCCATTGAAA**C**CAATTCTTGAGATTAAAT 513

L4E1 AAT**T**GTTATACGCTCTCATATTTTCAATTTGTCCATTGAAA**C**CAATTCTTGAGATTAAAT 513

G8H8 ------------------------------------------------------------ 360

G39B9 AATAGTTATACGCTCTCATATTTTCAATTTGTCCATTGAAATCAATTCTTGAGATTAAAT 512

L2C8 AATAGTTATACGCTCTTATATTTTCAATTTGTC**A**ATTGAAATCAATTCTTGAGATTAAAT 522

S11D7 AATAGTTATACGCTCTCATATTTTCAATTTGTC**A**ATTGAAATCAATTCTTGAGATTAAAT 475

L6D6 AAT**T**GTTATACGCTCTCATATTTTCAATTTGTCCATTGAAA**C**CAATTCTTGAGATTAAAT 513

L9F12 AAT**T**GTTATACGCTCTCATATTTTCAATTTGTCCATTGAAA**C**CAATTCTTGAGATTAAAT 513

G8B4 AAT**T**GTTATACGCTCTCATATTTTCAATTTGTCCATTGAAATCAATTCTTGAGATTAAAT 513

G3G5 ------------------------------------------------------------ 436

G27E6 AAT**T**GTTATACGCTCTCATATTTTCAATTTGTCCATTGAAATCAATTCTTGAGATTAAAT 513

G5D4 AATAGCTATACGCTCTCATATTTTCAATTTGTC**T**ATTGAAATCAATTCTTGAGATTAAAT 513

G1A5 AATAGTTATA**T**GCTCTCATATTTTCAATTTGTCCATTGAAATCAATTCTTGAGATTAAAT 513

G6G2 AAT**T**GTTATACGCTCT**G**ATATTTTCAATTTGTCCATTGAAATCAATTCTTGAGATTAAAT 513

G34G4 AATAGTTATA**T**GCTCTCATATTTTCAATTTGTCCATTGAAATCAATTCTTGAGATTAAAT 513

G38B12 AAT**T**GTTATACGCTCTCATATTTTCAATTTGTCCATTGAAATCAATTCTTGAGATTAAAT 512

G8B10 AAT**T**GTTATACGCTCTCATATTTTCAATTTGTCCATTGAAATCAATTCTTGAGATTAAAT 513

G1H6 AATAGTTATACGCTCTCATATTTTCAATTTGTC**T**ATTGAAATCAATTCTTGAGATTAAAT 513

G36E3 AAT**T**GTTATACGCTC**CG**ATATTTTCAATTTGTCCATTGAAATCAATTCTTGAGATTAAAT 513

G4H9 ------------------------------------------------------------ 439

G33B10 AAT**T**GTTATACGCTCTCATATTTTCAATTTGTCCATTGAAATCAATTCTTGAGATTAAAT 513

L3A11 AATAGTTATACGCTCTCATATTTTCAATTTGTCCATTGAAATCAATTCTTGAGATTAAAT 513

L6H9 AATAGTTATA**T**GCTCTCATATTTTCAATTTGTCCATTGAAATCAATTCTTGAGATTAAAT 513

L5E1 AATAGTTATACGCTCTCATATTTTCAATTTGTCCATTGAAATCAATTCTTGAGATTAAAT 513

L3G10 AATAG**A**TATACGCTCTCATATTTTCAATTTGTC**T**ATTGAAATCAATTCTTGAGATTAAAT 513

L7D10 AATAGTTATA**T**GCTCTCATATTTTCAATTTGTCCATTGAAATCAATTCTTGAGATTAAAT 513

L7B7 AATAGTTATACGCTCTCATATTTTCAATTTGTC**A**ATTGAAATCAATTCTTGAGATTAAAT 522

L7G9 AATAGTTATACGCTCTCATATTTTCAATTTGTC**T**ATTGAAATCAATTCTTGAGATTAAAT 522

L2F3 AATAGTTATACGCTCTCATATTTTCAATTTGTCCATTGAAATCAATTCTTGAGATTAAAT 513

L1F3 AATAGTTATACGCTCTCATATTTTCAATTTGTCCATTGAAATCAATTCTTGAGATTAAAT 513

L1D3 AATAGTTATACGCTCTCATATTTTCAATTTGTC**T**ATTGAAATCAATTCTTGAGATTAAAT 522

L4D7 AAT**T**GTTATACGCTCTCATATTTTCAATTTGTC**T**ATTGAAATCAATTCTTGAGATTAAAT 522

L10A5 AATAGTTATACGCTCTCATATTTTCAATTTGTCCATTGAAATCAATTCTTGAGATTAAAT 513

L3D4 AAT**T**GTTATACGCTCTCATATTTTCAATTTGTCCATTGAAATCAATTCTTGAGATTAAAT 513

L8D6 AAT**T**GTTATACGCTCTCATATTTTCAATTTGTCCATTGAAATCAATTCTTGAGATTAAAT 513

S6F4 AAT**T**GTTATACGCTCTCATATTTTCAATTTGTCCATTGAAATCAATTCTTGAGATTAAAT 513

S19H4 AATAGTTATACGCTCTCATATTTTCAATTTGTCCATTGAAATCAATTCTTGAGATTAAAT 513

S6C12 AAT**T**GTTATACGCTCTCATATTTTCAATTTGTCCATTGAAATCAATTCTTGAGATTAAAT 522

L1H10 AAT**T**GTTATACGCTCTCATATTTTCAATTTGTC**T**ATTGAAATCAATTCTTGAGATTAAAT 522

L5G9 AAT**T**GTTATACGCTCTCATATTTTCAATTTGTC**T**ATTGAAATCAATTCTTGAGATTAAAT 513

S8E10 AATAGTTATAC**A**CTCTCATATTTTC**T**ATTTGTC**A**ATTGAAATCAATTCTTGAGATTAAAT 513

S18B4 AATAGTTATAC**A**CTCTCATATTTTCA**G**TTTGTC**A**ATTGAAATCAATTCTTGAGATTAAAT 513

G34G2 AAT**T**GTTATACGCTCTCATATTTTCAATTTGTCCATTGAAA**C**CAATTCTTGAGATTAAAT 513

G16C1 AAT**T**GTTATACGCTCT**G**ATATTTTCAATTTGTCCATTGAAATCAATTCTTGAGATTAAAT 513

S18G4 AATAGTTATAC**A**CTCTCATATTTTCAATTTGTC**A**ATTGAAATCAATTCTTGAGATTAAAT 513

S16F12 AATAGTTATAC**A**CTCTCATATTTTCAATTTGTC**A**ATTGAAATCAATTCTTGAGATTAAAT 513

G27D12 AAT**T**GTTATACGCTCTCATATTTTCAATTTGTCCATTGAAA**C**CAATTCTTGAGATTAAAT 513

G2H6 AATAGTTATACGCTCTCATATTTTCAATTTGTC**T**ATTGAAATCAATTCTTGAGATTAAAT 513

L2F4 AATAGTTATA**T**GCTCTCATATTTTCAATTTGTCCATTGAAATCAATTCTTGAGATTAAAT 513

S21G8 AATAGTTATACGCTCTCATATTTTCAATTTGTCCATTGAAATCAATTCTTGAGATTAAAT 513

G1C2 AATAGTTATACGCTCT**T**ATATTTTCAATTTGTC**A**ATTGAAATCAATTCTTGAGATTAAAT 522

S16A9 AATAGTTATAC**A**CTCTCATATTTTCAATTTGTC**A**ATTGAAATCAATTCTTGAGATTAAAT 513

G5G10 AAT**T**GTTATACGCTCTCATATTTTCAATTTGTCCATTGAAA**C**CAATTCTTGAGATTAAAT 513

L9F5 TCTTGAATGTAAAAAAATAAATAATTGAGTATTTTATC- 551

G38F8 TCTTGAATG------------------------------ 531

S21E5 TCTTGAATGT----------------------------- 523

G16C5 TCTTGAATG------------------------------ 522

S8C8 TCTTGAATGT----------------------------- 521

G16E1 TCTTGAATGT----------------------------- 522

S4C12 TCTTG---------------------------------- 518

L6G1 TCT------------------------------------ 516

L6G2 TCTTGAAT------------------------------- 521

S8H3 TCTTGAAT------------------------------- 521

S19E10 TCTTGAATGT----------------------------- 485

S21C5 TCTTGAATGTAAAAAAAT--------------------- 531

G8A6 TCTTG---------------------------------- 518

G28H7 TCTTGAAT------------------------------- 521

L6B5 TCTTGAAT------------------------------- 521

L1H9 TCTTGAATG------------ 540

L6E2 TCTTGAATGT----------------------------- 523

G39G10 TCTTGAATGTAAAAAAATAAATAATTGAGTATT------ 555

G27G6 TCTTGAATGT----------------------------- 523

L4E1 TCTTGAATGT----------------------------- 523

G8H8 --------------------- 360

G39B9 TCTTGA--------------------------------- 518

L2C8 TCTTGA--------------------------------- 528

S11D7 TCTTGAAT------------------------------- 483

L6D6 TCTTGAATGTAAAAAAAT--------------------- 531

L9F12 TCTTGA--------------------------------- 518

G8B4 TCTTGAATG------------------------------ 522

G3G5 --------------------- 436

G27E6 TCT------------------------------------ 516

G5D4 TCTTGAATGT----------------------------- 523

G1A5 TCTTGAATGT----------------------------- 523

G6G2 TCTTGAATGTAAAAAAATAAATAATTGAGTATTTTATTC 552

G34G4 TCTTGAATGTAAAAAAATAAATAATTGAGTATT------ 549

G38B12 TCTTGAATGTAAAAAAATAAATAATTGAGTATTTTATTC 552

G8B10 TCTTGAATGT----------------------------- 523

G1H6 TCTTGAATG------------------------------ 522

G36E3 TCTTGAATGTAAAAAAATAAATAATTGAGTATTTTATTC 552

G4H9 --------------------------------------- 439

G33B10 TCTTGAAT------------------------------- 521

L3A11 TCTTGAATGTAAAAAAATAAATAATTGAGTATTTTATTC 552

L6H9 TCTTGAATGTAAAAAAATAAATAATTGAGTATTTTATTC 552

L5E1 TCTTGAATGTAAAAAAATAAATAATTGAGTATTTTATTC 552

L3G10 TCTTGAATGTAAAAAAATAAATAATTGAGTATTTTATTC 552

L7D10 TCTTGAATGT----------------------------- 523

L7B7 TCTTGAATGT----------------------------- 532

L7G9 TCTTGAATGT----------------------------- 532

L2F3 TCTTGAATG------------------------------ 522

L1F3 TCTTGAATGTATAAAAATAAATAATTGAGTATTTTATTC 552

L1D3 TCTTGAATGT----------------------------- 532

L4D7 TCTTGAATGTAAAAAAATAAATAATTGAGTATTTTAT-- 559

L10A5 TCTTGAATGT 523

L3D4 TCTTGAAT------------------------------- 521

L8D6 TCTTGAATGTAAAAAACC--------------------- 531

S6F4 TCTTGAATGTAAAAAAGC--------------------- 531

S19H4 TCTTGAATGT----------------------------- 523

S6C12 TCTTGAATGTAAAAAAATAAATAATTGAGTATTTTATTC 561

L1H10 TCTTGAATGT----------------------------- 532

L5G9 TCT------------------------------------ 516

S8E10 TCTTGAAT------------------------------- 521

S18B4 TCTTGAAT------------------------------- 521

G34G2 TCTTGAATGTAAAAAAAT--------------------- 531

G16C1 TC------------------------------------- 515

S18G4 TCTTGAATG------------------------------ 522

S16F12 TCTTGAATGT----------------------------- 523

G27D12 TCTTGAATGT----------------------------- 523

G2H6 TCTTGAATG------------------------------ 522

L2F4 TCTTGAATGT----------------------------- 523

S21G8 TCTTGAATG------------------------------ 522

G1C2 TCTTGAATGT----------------------------- 532

S16A9 TCTTGAAT------------------------------- 521

G5G10 TCTTGAATG------------------------------ 522

**B**

G21E12 CAGTTCAATTCGAACC**G**TTAATTCTTTC**G**ATATCCACTGGAACATCC**G**AAAACGAAA**ATG** 60

G13A12 CAGTTCAATTCGAACC**G**TTAATTCTTTC**G**ATATCCACTGGAACATCCAAAAACGAAA**ATG** 60

G11C1 CAGTTCAATTCGAACC**G**TTAATTCTTTC**G**ATATCCACTGGAACATCCAAAAACGAAA**ATG** 60

G13B3 CAGTTCAATTCGAACC**G**TTAATTCTTTC**G**ATATCCACTGGAACATCCAAAAA**G**GAAA**ATG** 60

L6A5 CAGTTCAATTCGAACC**G**TTAATTCTTTC**G**ATATCCACTGGAACATCCAAAAACGAAA**ATG** 60

G38A8 --GTTCAATTCGAACC**G**TTAATTCT**G**TC**G**ATATCCACTGGAACATCCAAAAACGAAA**ATG** 58

G11B12 CAGTTCAATTCGAACC**G**TTAATTCTTTC**G**ATATCCACTGGAACATCCAAAAACGAAA**ATG** 60

G3H11 CAGTTCAATTCGAACC**G**TTAATTCTTTC**G**ATATCCACTGGAACATCCAAAAACGAAA**ATG** 60

G10A4 CAGTTCAATTCGAACC**G**TTAATTCTTTC**G**ATATCCACTGGAACATCCAAAAACGAAA**ATG** 60

L1F9 CAGTTCAATTCGAACC**G**TTAATTCTTTC**G**ATATCCACTGGAACATCCAAAAACGAAA**ATG** 60

G29E11 CAGTTC**C**ATTCGAACC**G**TTAATTCTTTC**G**ATATCCACTGGAACATCCAAAAACGAAA**ATG** 60

L3A11 CAGTTCAATTCGAACC**G**TTAATTCTTTC**G**ATATCCACTGGAACATCCAAAAACGAAA**ATG** 60

L8C1 CAGTTCAATTCGAACC**G**TTAATTCTTTC**G**ATATCCACTGGAACATCCAAAAACGAAA**ATG** 60

G12B12 CAGTTCAATTCGAACC**G**TTAATTCTTTC**G**ATATCCACTGGAACATCCAAAAACGAAA**ATG** 60

S16B9 -AGTTCAATTCGAACCATTAATTCTTTCAATATCCACTGGAACATCCAAAAACGAAA**ATG** 59

L8A5 CAGTTCAATTCGAACCATTAATTCTTTCAATATCCACTGGAACATCCAAAAACGAAA**ATG** 60

G3D11 CAGTTCAATTCGAACCATTAATTCTTTCAATATCCACTGGAACATCCAAAAACGAAA**ATG** 60

G15B2 CAGTTCAATTCGAACCATTAATTCTTTCAATATCCACTGGAACATCCAAAAACGAAA**ATG** 60

G17E10 CAGTTCAATTCGAACCATTAATTCTTTCAATATCCACTGGAACATCCAAAAACGAAA**ATG** 60

G28C4 CAGTTCAATTCGAACCATTAATTCTTTCAATATCCACTGGAACATCCAAAAACGAAA**ATG** 60

G17B1 CAGTTCAATTCGAACCATTAATTCTTTCAATATCCACTGGAACATCCAAAAACGAAA**ATG** 60

G16F5 CAGTTCAATTCGAACCATTAATTCTTTCAATATCCACTGGAACATCCAAAAACGAAA**ATG** 60

G2B3 CAGTTCAATTCGAACCATTAATTCTTTCAATATCCACTGGAACATCCAAAAACGAAA**ATG** 60

G33D9 CAGTTCAATTCGAACCATTAATTCTTTCAATATCCACTGGAACATCCAAAAACGAAA**ATG** 60

G31A5 CAGTTCAATTCGAACCATTAATTCTTTCAATATCCACTGGAACATCCAAAAACGAAA**ATG** 60

G8H5 CAGTTCAATTCGAACCATTAATTCTTTCAATATCCACTGGAACATCCAAAAACGAAA**ATG** 60

L2F2 CAGTTCAATTCGAACCATTAATTCTTTCAATATCCACTGGAACATCCAAAAACGAAA**ATG** 60

G4F2 CAGTTCAATTCGAACCATTAATTCTTTCAATATCCACTGGAACATCCAAAAACGAAA**ATG** 60

L3F1 CAGTTCAATTCGAACCATTAATTCTTTCAATATCCACTGGAACATCC**G**AAAACGAAA**ATG** 60

G36B7 CAGTTCAATTCGAACCATTAATTCTTTCAATATCCACTGGAACATCCAAAAACGAAA**ATG** 60

G18A2 CAGTTCAATTCGAACCATTAATTCTTTCAATATCCACTGGAACATCCAAAAACGAAA**ATG** 60

G38E8 CAGTTCAATTCGAACCATTAATTCTTTCAATATCCACTGGAACATCCAAAAACGAAA**ATG** 60

L3C5 CAGTTCAATTCGAACCATTAATTCTTTCAATATCCACTGGAACATCCAAAAACGAAA**ATG** 60

G36H7 CAGTTCAATTCGAACCATTAATTCTTTCAATATCCACTGGAACATCCAAAAACGAAA**ATG** 60

L1C6 CAGTTCAATTCGAACCATTAATTCTTTCAATATCCACTGGAACATCCAAAAACGAAA**ATG** 60

G17C9 CAGTTCAATTCGAAC**T**ATTAATTCTTTCAATATCCACTGGAACATCCAAAAACGAAA**ATG** 60

G12B5 CAGTTCAATTC**C**AACCATTAATTCTTTCAATATCCACTGGAACATCCAAAAACGAAA**ATG** 60

G21E5 CAGTTCAATTCGAACCATTAATTCTTTCAATATCCACTGGAACATCCAAAAACGAAA**ATG** 60

L3B1 CAGTTCAATTCGAACCATTAATTCTTTCAATATCCACTGGAACA**C**CCAAAAACGAAA**ATG** 60

G8H12 CAGTTCAA**C**TCGAACCATTAATTCTTTCAATATCCACTGGAACATCCAAAAACGAAA**ATG** 60

G10A9 CAGTTCAATTCGAACCATTAATTCTTTCAATATCCACTGGAACATCCAAAAACGAAA**ATG** 60

G3A11 CAGTTCAATTCGAACCATTAATTCTTTCAATATCCACTGGAACATCCAAAAACGAAA**ATG** 60

S10A3 CAGTTCAATTCGAACCATTAATTCTTTCAATATCCACTGGAACATCCAAAAACGAAA**ATG** 60

S4C1 CAGTTCAATTCGAACCATTAATTCTTTCAATATCCACTGGAACATCCAAAAACGAAA**ATG** 60

S10H9 CAGTTCAATTCGAACCATTAATTCTTTCAATATCCACTGGAACATCCAAAAACGAAA**ATG** 60

S10A11 CAGTTCAATTCGAACCATTAATTCTTTCAATATCCACTGGAACATCCAAAAACGAAA**ATG** 60

S10B5 CAGTTCAATTCGAACCATTAATTCTTTCAATATCCACTGGAACATCCAAAAACGAAA**ATG** 60

S2A12 CAGTTCAA**C**TCGAACCATTAATTCTTTCAATATCCACTGGAACATCCAAAAACGAAA**ATG** 60

S13G9 CAGTTCAATTCGAACC**G**TTAATTCTTTCAATATCCACTGGAACATCCAAAAACGAAA**ATG** 60

S8D11 CAGTTCAATTCGAACCATTAATTCTTTC**G**ATATCCACTGGAACATCCAAAAACGAAA**ATG** 60

S12G7 CAGTTCAATTCGAACCATTAATTCTTTC**G**ATATCCACTGGAACATCCAAAAACGAAA**ATG** 60

G33C1 CAGTTCAATTCGAACC**G**TTAATTCTTTC**G**ATATCCACTGGAACATCCAAAAACGAAA**ATG** 60

G3B9 CAGTTCAATTCGAACCATTAATTCTTTCAATATCCACTGGAACATCCAAAAACGAAA**ATG** 60

L7F3 CAGTTCAATTCGAACCATTAATTCTTTCAATATCCACTGGAACATCCAAAAACGAAA**ATG** 60

L4B1 CAGTTCAATTCGAACCATTAATTCTTTCAATATCCACTGGAACATCCAAAAACGAAA**ATG** 60

G14G7 CAGTTCAATTCGAACCATTAATTCTTTCAATATCCACTGGAACATCCAAAAACGAAA**ATG** 60

L6B11 CAGTTCAATTCGAACCATTAATTCTTTCAATATCCACTGGAACATCCAAAAACGAAA**ATG** 60

L5A6 CAGTTCAATTCGAACCATTAATTCTTTCAATATCCACTGGAACATCCAAAAACGAAA**ATG** 60

L6D10 CAGTTCAATTCGAACC**G**TTAATTCTTTC**G**ATATCCACTGGAACATCCAAAAACGAAA**ATG** 60

L6F9 CAGTTCAA**C**TCGAACCATTAATTCTTTC**G**ATATCCACTGGAACATCCAAAAACGAAA**ATG** 60

S17H5 ---------------------------------------GAACATCCAAAAACGAAA**ATG** 21

S11H2 CAGTTCAATTCGAACC**G**TTAATTCTTTCAATATCCACTGGAACATCCAAAAACGAAA**ATG** 60

S21F5 CAGTTCAATTCGAACC**G**TTAATTCTTTCAATATCCACTGGAACATCCAAAAACG**G**AA**ATG** 60

S18G6 CAGTTCAATTCGAACC**G**TTAATTCTTTCAATATCCACTGGAACATCCAAAAACGAAA**ATG** 60

S7C1 CAGTTCAATTCGAACC**G**TTAATTCTTTCAATATCCACTGGAACATCCAAAAACGAAA**ATG** 60

S13A12 CAGTTCAATTCGAACC**G**TTAATTCTTTC**G**ATATCCACTGGAACATCCAAAAACGAAA**ATG** 60

G21E12 TCAAAATTTTTACTAGCTTTCGCCGTCATCGCCGTCTGCCTTGT**T**GCAGCTCAGGCTGCT 120

G13A12 TCAAAATTTTTACTAGCTTTCGCCGTCATCGCCGTCTGCCTTGT**T**GCAGCTCAGGCTGCT 120

G11C1 TCAAAATTTTTACTAGCTTTCGCCGTCATCGCCGT**T**TGCCTTGT**T**GCAGCTCAGGCTGCT 120

G13B3 TCAAAATTTTTACTAGCTTTCGCCGTCATCGCCGTCTGCCTTGT**T**GCAGCTCAGGCTGCT 120

L6A5 TCAAAATTTTTACTAGCTTTCGCCGTCATCGCCGTCTGCCTTGT**T**GCAGCTCAGGCTGCT 120

G38A8 TCAAAATTTTTACTAGCTTTCGCCGTCATCGCCGTCTGCCTTGT**T**GCAGCTC**G**GGCTGCT 118

G11B12 TCAAAATTTTTACTAGCTTTCGCCGTCATCGCCGTCTGCCTTGT**T**GCAG**A**TCAGGCTGCT 120

G3H11 TCAAAATTTTTACTAGCTTTCGCCGTCATCGCCGTCTGCCTTGT**T**GCAGCTCAGGCTGCT 120

G10A4 TCAAAATTTTTACTAGCTTTCGCCGTCATCGCCGTCTGCCTTGT**T**GCAGCTCAGGCTGCT 120

L1F9 TCAAAATTTTTACTAGCTTTCGCCGTCATCGCCGTCTGCCTTGT**T**GCAGCTCAGGCTGCT 120

G29E11 TCAAAATTTTTACTAGCTTTCGCCGTCATCGCCGTCTGCCTTGT**T**GCAGCTCAGGCTGCT 120

L3A11 TCAAAATTTTTACTAGCTTTCGCCGTCATCGCCGTCTGCCTTGT**T**GCAGCTCAGGCTGCT 120

L8C1 TCAAAATTTTTACTAGCTTTCGCCGTCATCGCCGTCTGCCTTGT**T**GCAGCTCAGGCTGCT 120

G12B12 TCAAAATTTTTACTAGCTTTCGCCGTCATCGCCGTCTGCCTTGT**T**GCAGCTCAGGCTGCT 120

S16B9 TCAAAATTTTTACTAGCTTTCGCCGTCATCGCCGT**T**TGCCTTGTCGCAGCTCAGGCTGCT 119

L8A5 TCAAAATTTTTACTAGCTTTCGCCGTCATCGCCGTCTGCCTTGTCGCAGCTCAGGCTGCT 120

G3D11 TCAAAATTTTTACTAGCTTTCGCCGTCATCGCCGTCTGCCTTGTCGCAGCTCAGGCTGCT 120

G15B2 TCAAAATTTTTACTAGCTTTCGCCGTCATCGCCGTCTGCCTTGTCGCAGCTCAGGCTGCT 120

G17E10 TCAAAATTTTTACTAGCTTTCGCCGTCATCGCCGTCTGCCTTGTCGCAGCTCAGGCTGCT 120

G28C4 TCAAAATTTTTACTAGCTTTCGCCGTCATCGCCGTCTGCCTTGTCGCAGCTCAGGCTGCT 120

G17B1 TCAAAATTTTTACTAGCTTTCGCCGTCATCGCCGTCTGCCTTGTCGCAGCTCAGGCTGCT 120

G16F5 TCAAAATTTTTACTAGCTTTCGCCGTCATCGCCGTCTGCCTTGTCGCAGCTCAGGCTGCT 120

G2B3 TCAAAATTTTTACTAGCTTTCGCCGTCATCGCCGTCTGCCTTGTCGCAGCTCAGGCTGCT 120

G33D9 TCAAAATTTTTACTAGCTTTCGCCGTCATCGCCGTCTGCCTTGTCGCAGCTCAGGCTGCT 120

G31A5 TCAAAATTTTTACTAGCT**C**TCGCCGTCATCGCCGTCTGCCTTGTCGCAGCTCAGGCTGCT 120

G8H5 TCAAAATTTTTACTAGCTTTCGCCGTCATCGCCGTCTGCCTTGTCGCAGCTCAGGCTGCT 120

L2F2 TCAAAATTTTTACTAGCTTTCGCCGTCATCGCCGTCTGCCTTGTCGCAGCTCAGGCTGCT 120

G4F2 TCAAAATTTTTACTAGCTTTCGCCGTCATCGCCGTCTGCCTTGTCGCAGCTCAGGCTGCT 120

L3F1 TCAAAATTTTTACTAGCTTTCGCCGTCATCGCCGTCTGCCTTGTCGCAGCTCAGGCTGCT 120

G36B7 TCAAAATTTTTACTAGCTTTCGCCGTCATCGCCGTCTGCCTTGTCGCAGCTCAGGCTGCT 120

G18A2 TCAAAATTTTTACTAGCTTTCGCCGTCATCGCCGTCTGCCTTGTCGCAGCTCAGGCTGCT 120

G38E8 TCAAAATTTTTACTAGCTTTCGCCGTCATCGCCGTCTGCCTTGTCGCAGCTCAGGCTGCT 120

L3C5 TCAAAATTTTTACTAGCTTTCGCCGTCATCGCCGTCTGCCTTGTCGCAGCTC**G**GGCTGCT 120

G36H7 TCAAAATTTTTACTAGCTTTCGCCGTCATCGCCGTCTGCCTTG**C**CGCAGCTCAGGCTGCT 120

L1C6 TCAAAATTTTTACTAGCTTTCGCCGTCATCGCCGTCTGCCTTGTCGCAGCTCAGGCTGCT 120

G17C9 TCAAAATTTTTACTAGCTTTCGCCGTCATCGCCGTCTGCCTTGTCGCAGCTCAGGCTGCT 120

G12B5 TCAAAATTTTTACTAGCTTTCGCCGTCATCGCCGTCTGCCTTGTCGCAGCTCAGGCTGCT 120

G21E5 TCAAAATTTTTACTAGCTTTCGCCGTCATCGCCGTCTGCCTTGTCGCAGCTCAGGCTGCT 120

L3B1 TCAAAATTTTTACTAGCTTTCGCCGTCATCGCCGTCTGCCTTGTCGCAGCTCAGGCTGCT 120

G8H12 TCAAAATTTTTACTAGCTTTCGCCGTCATCGCCGTCTGCCTTGTCGCAGCTCAGGCTGCT 120

G10A9 TCAAAATTTTTACTAGCTTTCGCCGTCATCGCCGTCTGCCTTGTCGCAGCTCAGGCTGCT 120

G3A11 TCAAAATTTTTACTAGCTTTCGCCGTCATCGCCGTCTGCCTTGTCGCAGCTCAGGCTGCT 120

S10A3 TCAAAATTTTTACTAGCTTTCGCCGTCATCGCCGTCTGCCTTGT**T**GCAGCTCAGGCTGCT 120

S4C1 TCAAAATTTTTACTAGCTTTCGCCGTCATCGCCGTCTGCCTTGT**T**GCAGCTC**G**GGCTGCT 120

S10H9 TCAAAATTTTTACTAGCTTTCGCCGTCATCGCCGTCTGCCTTGT**T**GCAGCTCAGGCTGCT 120

S10A11 TCAAAATTTTTACTAGCTTTCGCCGTCATCGCCGTCTGCCTTGT**T**GCAGCTCAGGCTGCT 120

S10B5 TCAAAATTTTTACTAGCTTTCGCCGTCATCGCCGTCTGCCTTGT**T**GCAGCTCAGGCTGCT 120

S2A12 TCAAAATTTTTACTAGCTTTCGCCGTCATCGCCGTCTGCCTTGT**T**GCAGCTCAGGCTGCT 120

S13G9 TCAAAATTTTTACTAGCTTTCGCCGTCATCGCCGTCTGCCTTGT**T**GCAGCTCAGGCTGCT 120

S8D11 TCAAAATTTTTA**T**TAGCTTTCGCCGTCATCGCCGTCTGCCTTGT**T**GCAGCTCAGGCTGCT 120

S12G7 TCAAAATTTTTA**T**TAGCTTTCGCCGTCATCGCCGTCTGCCTTGT**T**GCAGCTCAGGCTGCT 120

G33C1 TCAAAATTTTTACTAGCTTTCGCCGTCATCGCCGTCTGCCTTGT**T**GCAGCTCAGGCTGCT 120

G3B9 TCAAAATTTTTACTAGCTTTCGCCGTCATCGCCGTCTGCCTTGTCGCAGCTCAGGCTGCT 120

L7F3 TCAAAATTTTTACTAGCTTTCGCCGTCATCGCCGTCTGCCTTGTCGCAGCTCAGGCTGCT 120

L4B1 TCAAAATTTTTACTAGCTTTCGCCGTCATCGCCGTCTGCCTTGTCGCAGCTCAGGCTGCT 120

G14G7 TCAAAATTTTTACTAGCTTTCGCCGTCATCGCCGTCTGCCTTG**C**CGCAGCTCAGGCTGCT 120

L6B11 TCAAAATTTTTACTAGCTTTCGCCGTCATCGCCGTCTGCCTTGTCGCAGCTCAGGCTGCT 120

L5A6 TCAAAATTTTTACTAGCTTTCGCCGTCATCGCCGTCTGCCTTGT**T**GCAGCTCAGGCTGCT 120

L6D10 TCAAAATTTTTACTAGCTTTCGCCGTCATCGCCGTCTGCCTTGT**T**GCAGCTCAGGCTGCT 120

L6F9 TCAAAATTTTTACTAGCTTT**T**GCCGTCATCGCCGTCTGCCTTGT**T**GCAG**T**TCAGGCTGCT 120

S17H5 TCAAAATTTTTA**T**TAGCTTTCGCCGTCATCGC**T**GT**T**TGCCTTGT**T**GCAGCTCAGGCTGCT 81

S11H2 TCAAAATTTTTA**T**TAGCTTTCGCCGTCATCGC**T**GTCTGCCTTGT**T**GCAGCTCAGGCTGCT 120

S21F5 TCAAAATTTTTA**T**TAGCTTTCGCCGTCATCGC**T**GTCTGCCTTGT**T**GCAGCTCAGGCTGCT 120

S18G6 TCAAAATTTTTA**T**TAGCTTTCGCCGTCATCGC**T**GTCTGCCTTGT**T**GCAG**T**TCAGGCTGCT 120

S7C1 TCAAAATTTTTA**T**TAGCTTTCGCCGTCATCGC**T**GTCTGCCTTGT**T**GCAGCTCAGGCTGCT 120

S13A12 TCAAAATTTTTA**T**TAGCTTTCGCCGTCATCGC**T**GTCTGCCTTGT**T**GCAGCTCAGGCTGCT 120

G21E12 **A**AACCTAAAAAAGGCAAAAAGCCCGCAGCTGCACCGGCAGGTCCAACCT**T**TGATCCAGCA 180

G13A12 **A**AACCTAAAAAAGGCAAAAAGCCCGCAGCTGCACCGGCAGGTCCAACCT**T**TGATCCAGCA 180

G11C1 **A**AACCTAAAAAAGGCAAAAAGCCCGCAGCTGCACCGGCAGGTCCAACCT**T**TGATCCAGCA 180

G13B3 **A**AACCTAAAAAAGGCAAAAAGCCCGCAGCTGCACCGGCAGGTCCA**C**CCTCTGATCCAGCA 180

L6A5 **A**AACCTAAAAAAGGCAAAAAGCCCGCAGCTGCACCGGCAGGTCCAACCTCTGATCCAGCA 180

G38A8 **A**AACCTAAAAAAGGCAAAAAGCCCGCAGCTGCACCGGCAGGTCCAACCTCTGATCCAGCA 178

G11B12 **A**AACCTAAAAAAGGCAAAAAGCCCGCAGCTGCACCGGCAGGTCCAACCTCTGATCCAGCA 180

G3H11 **A**AACCTAAAAAAGGCAAAAAGCCCGCAGCTGCACCGGCAGGTCCAACCTCTGATCCAGCA 180

G10A4 **A**AACCTAAAAAAGGCAAAAAGCCCGCAGCTGCACCGGCAGGTCCAACCTCTGATCCAGCA 180

L1F9 **A**AACCTAAAAAAGGCAAAAAGCCCGCAGCTGCACCGGCAGGTCCAACCTCTGATCCAGCA 180

G29E11 **A**AACCTAAAAAAGGCAAAAAGCCCGCAGCTGCACCGGCAGGTCCAACCTCTGATCCAGCA 180

L3A11 **A**AACCTAAAAAAGGCAAAAAGCCCGCAGCTGCACCGGCAGGTCCAACCTCTGATCCAGCA 180

L8C1 **A**AACCTAAAAAAGGCAAAAAGCCCGCAGCTGCACCGGCAGGTCCAACCTCTGAT**T**CAGCA 180

G12B12 **A**AACCTAAAAAAGGCAAAAAGCCCGCAGCTGCACCGGCAGGTCCAACCTCTGATCCAGCA 180

S16B9 **A**AACCTAAAAAAGGCAAAAAGCCCGCAGCTGCACCGGCAGGTCCAACCT**T**TGATCCAGCA 179

L8A5 **A**AACCTAAAAAAGGCAAAAAGCCCGCAGCTGCACCGGCAGGTCCAACCT**T**TGATCCAGCA 180

G3D11 **A**AACCTAAAAAAGGCAAAAAGCCCGCAGCTGCACCGGCAGGTCCAACCTCTGATCCAGCA 180

G15B2 **A**AACCTAAAAAAGGCAAAAAGCCCGCAGCTGCACCGGCAGGTCCA**C**CCTCTGATCCAGCA 180

G17E10 **A**AACCTAAAAAAGGCAAAAAGCCCGCAGCTGCACCGGCAGGTCCA**C**CCTCTGATCCAGCA 180

G28C4 **A**AACCTAAAAAAGGCAAAAAGCCCGCAGCTGCACCGGCAGGTCCAACCTCTGATCCAGCA 180

G17B1 **A**AACCTAAAAAAGGCAAAAAGCCCGCAGCTGCACCGGCAGGTCCAACCTCTGATCCAGCA 180

G16F5 **A**AACCTAAAAAAGGCAAAAAGCCCGCAGCTGCACCGGCAGGTCCAACCTCTGATCCAGCA 180

G2B3 **A**AACCTAAAAAAGGCAAAAAGCCCGCAGCTGCACCGGCAGGTCCAACCTCTGATCCAGCA 180

G33D9 **A**AACCTAAAAAAGGCAAAAAGCCCGCAGCTGCACCGGCAGGTCCAACCTCTGATCCAGCA 180

G31A5 **A**AACCTAAAAAAGGCAAAAAGCCCGCAGCTGCACCGGCAGGTCCAACC**C**CTGATCCAGCA 180

G8H5 **A**AACCTAAAAAAGGCAAAAAGCCCGCAGCTGCACCGGCAGGTCCAACCTCTGATCCAGCA 180

L2F2 **A**AACCTAAAAAAGGCAAAAAGCCCGCAGCTGCACCGGCAGGTCCAACCTCTGATCCAGCA 180

G4F2 **A**AACCTAAAAAAGGCAAAAAGCCCGCAGCTGCAC**T**GGCAGGTCCAACCTCTGATCCAGCA 180

L3F1 **A**AACCTAAAAAAGGCAAAAAGCCCGCAGCTGCACCGGCAGGTCCAACCTCTGATCCAGCA 180

G36B7 **A**AACCTAAAAAAGGCAAAAAGCCCGCAGCTGCACCGGCAGGTCCAACCTCTGATCCAGCA 180

G18A2 **A**AACCTAAAAAAGGCAAAAAGCCCGCAGCTGCACCGGCAGGTCCAACCTCTGATCCAGCA 180

G38E8 **A**AACCTAAAAAAGGCAAAAAGCCCGCAGCTGCACCGGCAGGTCCAACCTCTGAT**T**CAGCA 180

L3C5 **A**AACCTAAAAAAGGCAAAAAGCCCGCAGCTGCACCGGCAGGTCCAACCTCTGATCCAGCA 180

G36H7 **A**AACCTAAAAAAGGCAAAAAGCCCGCAGCTGCACCGGCAGGTCCAACCTCTGATCCAGCA 180

L1C6 **A**AACCTAAAAAAGGCAAAAAGCCCGCAGCTGCACCGGCAGGTCC**G**ACCTCTGATCCAGCA 180

G17C9 **A**AACCTAAAAAAGGCAAAAAGCCCGCAGCTGCACCGGCAGGTCCAACCTCTGATCCAGCA 180

G12B5 **A**AACCTAAAAAAGGCAAAAAGCCCGCAGCTGCACCGGCAGGTCCAACCTCTGATCCAGCA 180

G21E5 **A**AACCTAAAAAAGGCAAAAAGCCCGCAGCTGCACCGGCAGGTCCAACCTCTGATCCAGCA 180

L3B1 **A**AACCTAAAAAAGGCAAAAAGCCCGCAGCTGCACCGGCAGGTCCAACCTCTGATCCAGCA 180

G8H12 **A**AACCTAAAAAAGGCAAAAAGCCCGCAGCTGCACCGGCAGGTCCAACCTCTGATCCAGCA 180

G10A9 **A**AACCTAAAAAAGGCAAAAAGCCCGCAGCTGCACCGGCAGGTCCAACCTCTGATCCAGCA 180

G3A11 **A**AACCTAAAAAAGGCAAAAAGCCCGCAGCTGCACCGGCAGGTCCAACCTCTGATCCAGCA 180

S10A3 **A**AACCTAAAAAAGGCAAAAAGCCCGCAGCTGCACCGGCAGGTCCAACCTCTGATCCAGCA 180

S4C1 **A**AACCTAAAAAAGGCAAAAAGCCCGCAGCTGCACCGGCAGGTCCAACCTCTGATCCAGCA 180

S10H9 **A**AACCTAAAAAAGGCAAAAAGCCCGCAGCTGCACCGGCAGGTCCAACCTCTGATCCAGCA 180

S10A11 **A**AACCTAAAAAAGGCAAAAAGCCCGCAGCTGCACCGGCAGGTCCAACCTCTGATCCAGCA 180

S10B5 **A**AACCTAAAAAAGGCAAAAAGCCCGCAGCTGCACCGGCAGGTCCAACCTCTGATCCAGCA 180

S2A12 **A**AACCTAAAAAAGGCAAAAAGCCCGCAGCTGCACCGGCAGGTCCAACCTCTGATCCAGCA 180

S13G9 **A**AACCTAAAAAAGGCAAAAAGCCCGCAGCTGCACCGGCAGGTCCAACCTCTGATCCAGCA 180

S8D11 **A**AACCTAAAAAAGGCAAAAAG**G**CCGCAGCTGCACCGGCAGGTCCAACCTCTGATCCAGCA 180

S12G7 **A**AACCTAAAAAAGGCAAAAAG**G**CCGCAGCTGCACCGGCAGGTCCAACCTCTGATCCAGCA 180

G33C1 **A**AACCTAAAAAAGGCAAAAAGCCCGCAGCTGCACCGGCAGGTCCAACCTCTGATCCAGCA 180

G3B9 **A**AACCTAAAAAAGGCAAAAAGCCCGCAGCTGCACCGGCAGGTCCAACCTCTGATCCAGCA 180

L7F3 **A**AACCTAAAAAAGGCAAAAAGCCCGCAGCTGCACCGGCAGGTCCAACCTCTGATCCAGCA 180

L4B1 **A**AACCTAAAAAAGGCAAAAAGCCCGCAGCTGCACCGGCAGGTCCAACCTCTGATCCAGCA 180

G14G7 **A**AACCTAAAAAAGGCAAAAAGCCCGCAGCTGCACCGGCAGGTCCAACCTCTGATCCAGCA 180

L6B11 **A**AACCTAAAAAAGGCAAAAAGCCCGCAGCTGCACCGGCAGGTCCAACCTCTGATCCAGCA 180

L5A6 **A**AACCTAAAAAAGGCAAAAAGCCCGCAGCTGCACCGGCAGGTCCAACCTCTGATCCAGCA 180

L6D10 **A**AACCTAAAAAAGGCAAAAAGCCCGCAGCTGCACCGGCAGGTCCAACCTCTGATCCAGCA 180

L6F9 **A**AACCTAAAAAAGGCAAAAAGCCCGCAGCTGCACC**A**GCAGGTCCAACCTCTGATCCAGCA 180

S17H5 **A**AACCTAAAAAAGGCAAAAAGCCCGCAGCTGCACCGGCAGGTCCAACCT**T**TGATCCAGCA 141

S11H2 **A**AACCTAAAAAAGGCAAAAAGCCCGCAGCTGCACCGGCAGGTCCAACCT**T**TGATCCAGCA 180

S21F5 **A**AACCTAAAAAAGGCAAAAAGCCCGCAGCTGCACCGGCAGGTCCAACCTCTGATCCAGCA 180

S18G6 **A**AACCTAAAAAAGGCAAAAAGCCCGCAGCTGCACCGGCAGGTCCAACCTCTGATCCAGCA 180

S7C1 **A**AACCTAAAAAAGGCAAAAAGCCCGCAGCTGCACCGGCAGGTCCAACCTCTGATCCAGCA 180

S13A12 **A**AACCTAAAAAAGGCAAAAAGCCCGCAGCTGCACCGGCAGGTCCAACCTCTGATCCAGCA 180

G21E12 GTCGAGCCAAGCTCAGGTGACCTTTCTGATGACCAAACCATAAATTGGGATGCACTTTTA 240

G13A12 GTCGAGCCAAGCTCAGGTGACCTTTCTGATGACCAAACCATAAATTGGGATGCACTTTTA 240

G11C1 GTCGAGCCAAGCTCAGGTGACCTTT**T**TGATGACCAAACCATAAATTGGGATGCACTTTTA 240

G13B3 GTCGAGCCAAGCTCAGGTGACCTTT**T**TGATGACCAAACCATAAATTGGGATGCACTTTTA 240

L6A5 GTCGAGCCAAGCTCAGGTGACCTTTCTGATGACCAAACCATAAATTGGGATGCACTTTTA 240

G38A8 GTCGAGCCAAGCTCAGGTGACCTTTCTGATGACCAAACCATAAATTGGGATGCACTTTTA 238

G11B12 GTCGAGCCAAGCTCAGGTGACCTTTCTGATGACCAAACCATAAATTGGGATGCACTTTTA 240

G3H11 GTCGAGCCAAGCTCAGGTGACCTTTCTGATGACCAAACCATAAATTGGGATGCACTTTTA 240

G10A4 GTCGAGCCAAGCTCAGGTGACCTTTCTGATGACCAAACCATAAATTGGGATGCACTTTTA 240

L1F9 GTCGAGCCAAGCTCAGGTGACCTTTCTGATGACCAAACCATAAATTGGGATGCACTTTTA 240

G29E11 GTCGAGCCAAGCTCAGGTGACCTTTCTGATGACCAAACCATAAATTGGGATGCACTTTTA 240

L3A11 GTCGAGCCAAGCTCAGGTGACCTTTCTGATGACCAAACCATAAATTGGGATGCACTTTTA 240

L8C1 GTCGAGCCAAGCTCAGGTGACCTTTCTGATGACCAAACCATAAATTGGGATGCACTTTTA 240

G12B12 GTCGAGCCAAGCTCAGGTGACCTTTCTGATGACCAAACCATAAATTGGGATGCACTTTTA 240

S16B9 GTCGAGCCAAGCTCAGGTGACCTTTCTGATGACCAAACCATAAATTGGGATGCACTTTTA 239

L8A5 GTCGAGCCAAGCTCAGGTGACCTTT**T**TGATGACCAAACCATAAATTGGGATGCACTTTTA 240

G3D11 GTCGAGCCAAGCTCAGGTGACCTTTCTGATGACCAAACCATAAATTGGGATGCACTTTTA 240

G15B2 GTCGAGCCAAGCTCAGGTGACCTTTCTGATGACCAAACCATAAATTGGGATGCACTTTTA 240

G17E10 GTCGAGCCAAGCTCAGGTGACCTTTCTGATGACCAAACCATAAATTGGGATGCACTTTTA 240

G28C4 GTCGAGCCAAGCTCAGGTGACCTTTCTGATGACC**G**AACCATAAATTGGGATGCACTTTTA 240

G17B1 GTCGAGCCAAGCTCAGGTGACCTTTCTGATGACC**T**AACCATAAATTGGGATGCACTTTTA 240

G16F5 GTCGAGCCAAGCTCAGGTGACCTTTCTGATGACCAAACCATAAATTGGGATGCACTTTTA 240

G2B3 GTCGAGCCAAGCTCAGGTGACCTTTCTGATGACCAAACCATAAATTGGGATGCACTTTTA 240

G33D9 GTCGAGCCAAGCTCAGGTGACCTTTCTGATGACCAAACCATAAATTGGGATGCACTTTTA 240

G31A5 GTCGAGCCAAGCTCAGGTGACCTTTCTGATGACCAAACCATAAATTGGGATGCACTTTTA 240

G8H5 GTCGAGCCAAGCTCAGGTGACCTTTCTGATGACCAAACCATAAATTGGGATGCACTTTTA 240

L2F2 GTCGAGCCAAGCTCAGGTGACCTTTCTGATGACCAAACCATAAATTGGGATGCACTTTTA 240

G4F2 GTCGAGCCAAGCTCAGGTGACCTTTCTGATGACCAAACCATAAATTGGGATGCACTTTTA 240

L3F1 GTCGAGCCAAGCTCAGGTGACCTTTCTGATGACCAAACCATAAATTGGGATGCACTTTTA 240

G36B7 GTCGAGCCAAGCTCAGGTGACCTTTCTGATGACCAAACCATAAATTGGGATGCACTTTTA 240

G18A2 GTCGAGCCAAGCTCAGGTGACCTTTCTGATGACCAAACCATAAATTGGGATGCACTTTTA 240

G38E8 GTCGAGCCAAGCTCAGGTGACCTTTCTGATGACCAAACCATAAATTGGGATGCACTTTTA 240

L3C5 GTCGAGCCAAGCTCAGGTGACCTTTCTGATGACCAAACCATAAATTGGGATGCACTTTTA 240

G36H7 GTCGAGCCAAGCTCAGGTGACCTTTCTGATGACCAAACCATAAATTGGGATGCACTTTTA 240

L1C6 GTCGAGCCAAGCTCAGGTGACCTTTCTGATGACCAAACCATAAATTGGGATGCACTTTTA 240

G17C9 GTCGAGCCAAGCTCAGGTGACCTTTCTGATGACCAAACCATAAATTGGGATGCACTTTTA 240

G12B5 GTCGAGCCAAGCTCAGGTGACCTTTCTGATGACCAAACCATAAATTGGGATGCACTTTTA 240

G21E5 GTCGAGCCAAGCTCAGGTGACCTTTCTGATGACCAAACCATAAATTGGGATGCACTTTTA 240

L3B1 GTCGAGCCAAGCTCAGGTGACCTTTCTGATGACCAAACCATAAATTGGGATGCACTTTTA 240

G8H12 GTCGAGCCAAGCTCAGGTGACCTTTCTGATGACCAAACCATAAATTGGGATGCACTTTTA 240

G10A9 GTCGAGCCAAGCTCAGGTGACCTTTCTGATGACCAAACCATAAATTGGGATGCACTTTTA 240

G3A11 GTCGAGCCAAGCTCAGGTGACCTTTCTGATGACCAAACCATAAATTGGGATGCACTTTTA 240

S10A3 GTCGAGCCAAGCTCAGGTGACCTTTCTGA**C**GACCAAACCATAAATTGGGA**A**GCACTTTTA 240

S4C1 GTCGAGCCAAGCTCAGGTGACCTTTCTGA**C**GACCAAACCATAAATTGGGA**A**GCACTTTTA 240

S10H9 GTCGAGCCAAGCTCAGGTGACCTTTCTGA**C**GACCAAACCATAAATTGGGA**A**GCACTTTTA 240

S10A11 GTCGAGCCAAGCTCAGGTGACCTTTCTGA**C**GACCAAACCATAAATTGGGA**A**GCACTTTTA 240

S10B5 GTCGAGCCAAGCTCAGGTGACCTTTCTGA**C**GACCAAACCATAAATTGGGA**A**GCACTTTTA 240

S2A12 GTCGAGCCAAGCTCAGGTGACCTTTCTGA**C**GACCAAACCATAAATTGGGA**A**GCACTTTTA 240

S13G9 GTCGAGCCAAGCTCAGGTGACCTTTCTGA**C**GACCAAACCATAAATTGGGA**A**GCACTTTTA 240

S8D11 GTCGAGCCAAGCTCAGGTGACCTTTCTGA**C**GACCAAACCATAAATTGGGATGCACTTTTA 240

S12G7 GTCGAGCCAAGCTCAGGTGACCTTTCTGA**C**GACCAAACCATAAATTGGGATGCACTTTTA 240

G33C1 GTCGAGCCAAGCTCAGGTGACCTTTCTGATGACCAAACCATAAATTGGGATGCACTTTTA 240

G3B9 GTCGAGCCAAGCTCAGGTGACCTTTCTGATGACCAAACCATAAATTGGGATGCACTTTTA 240

L7F3 GTCGAGCCAAGCTCAGGTGACCTTTCTGATGACCAAACCATAAATTGGGATGCACTTTTA 240

L4B1 GTCGAGCCAAGCTCAGGTGACCTTTCTGATGACCAAACCATAAATTGGGATGCACTTTTA 240

G14G7 GTCGAGCCAAGCTCAGGTGACCTTTCTGATGACCAAACCATAAATTGGGATGCACTTTTA 240

L6B11 GTCGAGCCAAGCTCAGGTGACCTTTCTGATGACCAAACCATAAATTGGGATGCACTTTTA 240

L5A6 GTCGAGCCAAGCTCAGGTGACCTTTCTGATGACCAAACCATAAATTGGGATGCACTTTTA 240

L6D10 GTCGAGCCAAGCTCAGGTGACCTTT**T**TGATGACCAAACCATAAATTGGGATGCACTTTTA 240

L6F9 GTCGAGCCAAGCTCAGGTGACCTTTCTGA**C**GACCAAACCATAAATTGGGA**A**GCACTTTTA 240

S17H5 GTCGAGCCAAGCTCAGGTGACCTTT**T**TGA**C**GACCAAACCATAAATTGGGA**A**GCACTTTTA 201

S11H2 GTCGAGCCAAGCTCAGGTGACCTTTCTGA**C**GACCAAACCATAAATTGGGA**A**GCACTTTTA 240

S21F5 GTCGAGCCAAGCTCAGGTGACCTTTCTGA**C**GACCAAACCATAAATTGGGA**A**GCACTTTTA 240

S18G6 GTCGAGCCAAGCTCAGGTGACCTTTCTGA**C**GACCAAACCATAAATTGGGA**A**GCACTTTTA 240

S7C1 GTCGAGCCAAGCTCAGGTGACCTTTCTGA**C**GACCAAACCATAAATTGGGA**A**GCACTTTTA 240

S13A12 GTCGAGCCAAGCTCAGGTGACCTTTCTGA**C**GACCAAACCATAAATTGGGA**A**GCACTTTTA 240

G21E12 GCTGCCGACTCAAA---------TGCTAATGGGCTTGATGAAGC**C**GCAACGAAACCG**G**CA 291

G13A12 GCTGCCGACTCAAA---------TGCTAATGGGCTTGATGAAGC**C**GCAACGAAACCG**G**CA 291

G11C1 GCTGCCGACTCAAA---------TGCTAATGGGC**C**TGATGAAGC**C**GCAACGAAACCG**G**CA 291

G13B3 GCTGCCGACTCAAA---------TGCTAATGGGCTTGATGAAGC**C**GCAACGAAACCG**G**CA 291

L6A5 GCTGCCGACTCAAA---------TGCTAATGGGCTTGATGAAGC**C**GCAACGAAACCG**G**CA 291

G38A8 GCTGCCGACTCAAA---------TGCTAATGGGCTTGATGAAGC**C**GCAACGAAACCG**G**CA 289

G11B12 GCTGCCGACTCAAA---------TGCTAATGGGCTTGATGAAGC**C**GCAACGAAACCG**G**CA 291

G3H11 GCTGCCGACTCAAA---------TGCTAATGGGCTTGATGAAGC**C**GCAACGAAACCG**G**CA 291

G10A4 GCTGCCGACTCAAA---------TGCTAATGGGCTTGATGAAGC**C**GCAACGAAACCG**G**CA 291

L1F9 GCTGCCGACTCAAA---------TGCTAATGGGCTTGATGAAGC**C**GCAACGAAACCG**G**CA 291

G29E11 GCTGCCGACTCAAA---------TGCTAATGGGCTTGATGAAGC**C**GCAACGAAACCG**G**CA 291

L3A11 GCTGCCGACTCAAA---------TGCTAATGGGCTTGATGAAGC**C**GCAACGAAACCG**G**CA 291

L8C1 GCTGCCGACTCAAA---------TGCTAATGGGCTTGATGAAGC**C**GCAACGAAACCG**G**CA 291

G12B12 GCTGCCGACTCAAA---------TGCTAATGGGCTTGATGAAGC**C**GCAACGAAACCGACA 291

S16B9 GCTGCCGACTCAAATGCGGGAGATGCTAATGGGCTTGATGAAGCGGCAACGAAACCGACA 299

L8A5 GCTGCCGACTCAAATGCGGGAGATGCTAATGGGCTTGATGAAGCGGCAACGAAACCGACA 300

G3D11 GCTGCCGACTCAAATGCGGGAGATGCTAATGGGCTTGATGAAGCGGCAACGAAACCGACA 300

G15B2 GCTGCCGACTCAAATGCGGGAGATGCTAATGGGCTTGATGAAGCGGCAACGAAACCGACA 300

G17E10 GCTGCCGACTCAAATGCGGGAGATGCTAATGGGCTTGATGAAGCGGCAACGAAACCGACA 300

G28C4 GCTGCCGACTCAAATGCGGGAGATGCTAATGGGCTTGATGAAGCGGCAACGAAACCGACA 300

G17B1 GCTGCCGACTCAAATGCGGGAGATGCTAATGGGCTTGATGAAGCGGCAACGAAACCGACA 300

G16F5 GCTGCCGACTCAAATGCGGGAGATGCTAATGGGCTTGATGAAGCGGCAACGAAACCGACA 300

G2B3 GCTGCCGACTCAAATGCGGGAGATGCTAATGGGCTTGATGAAGCGGCAACGAAACCGACA 300

G33D9 GCTGCCGACTCAAATGCGGGAGATGCTAATGGGCTTGATGAAGCGGCAACGAAACCGAC**T** 300

G31A5 GCTGCCGACTCAAATGCGGGAGATGCTAATGGGCTTGATGAAGCGGCAACGAAACCGACA 300

G8H5 GCTGCCGACTCAAATGCGGGAGATGCTAATGGGCTTGATGAAGCGGCAACGAAACCGACA 300

L2F2 G**T**TGCCGACTCAAATGCGGGAGATGCTAATGGGCTTGATGAAGCGGCAACGAAACCGACA 300

G4F2 GCTGCCGACTCAAATGCGGGAGATGCTAATGGGCTTGATGAAGCGGCAACGAAACCGACA 300

L3F1 GCTGCCGACTCAAATGCGGGAGATGCTAATGGGCTTGATGAAGCGGCAACGAAACCGACA 300

G36B7 GCTGCCGACTCAAATGCGGGAGATGCTAATGGGC**C**TGATGAAGCGGCAACGAAACCGACA 300

G18A2 GCTGCCGACTCAAATGCGGGAGATGCTAATGGGCTTGATGAAGCGGCAACGAAACCGACA 300

G38E8 GCTGCCGACTCAAATGCGGGAGATGCTAATGGGCTTGATGAAGCGGCAACGAAACCGACA 300

L3C5 GCTGCCGACTCAAATGCGGGAGATGCTAATGGGCTTGATGAAGCGGCAACGAAACCGACA 300

G36H7 GCTGCCGACTCAAATGCGGGAGATGCTAATGGGCTTGATGAAGCGGCAACGAAACCGACA 300

L1C6 GCTGCCGACTCAAATGCGGGAGATGCTAATGGGCTTGATGAAGCGGCAACGAAACCGACA 300

G17C9 GCTGCCGACTCAAATGCGGGAGATGCTAATGGGCTTGATGAAGCGGCAACGAAACCGACA 300

G12B5 GCTGCCGACTCAAATGCGGGAGATGCTAATGGGCTTGATGAAGCGGCAACGAAACCGACA 300

G21E5 GCTGCCGACTCAAATGCGGGAGATGCTAATGGGCTTGATGAAGCGGCAACGAAACCGACA 300

L3B1 GCTGCCGACTCAAATGCGGGAGATGCTAATGGGCTTGATGAAGCGGCAACGAAACCGACA 300

G8H12 GCTGCCGACTCAAATGCGGGAGATGCTAATGGGCTTGATGAAGCGGCAACGAAACCGACA 300

G10A9 GCTGCCGACTCAAATGCGGGAGATGCTAATGGGCTTGATGAAGCGGCAACGAAACCG**G**CA 300

G3A11 GCTGCCGACTCAAATGCGGGAGATGCTAATGGGCTTGATGAAGCGGCAACGAAACCGACA 300

S10A3 GCTGCCGACTCAAATGCGGGAGATGCTAATGGGCTTGATGAAGCGGCAACGAAACCGACA 300

S4C1 GCTGCCGACTCAAATGCGGGAGATGCTAATGGGCTTGATGAAGCGGCAACGAAACCGACA 300

S10H9 GCTGCCGACTCAAATGCGGGAGATGCTAATGGGCTTGATGAAGCGGCAACGAAACCGACA 300

S10A11 GCTGCCGACTCAAATGCGGGAGATGCTAATGGGCTTGATGAAGCGGCAACGAAACCGACA 300

S10B5 GCTGCCGACTCAAATGCGGGAGATGCTAATGGGCTTGATGAAGCGGCAACGAAACCGACA 300

S2A12 GCTGCCGACTCAAATGCGGGAGATGCTAATGGGCTTGATGAAGCGGCAACGAAACCGACA 300

S13G9 GCTGCCGACTCAAATGCGGGAGATGCTAATGGGCTTGATGAAGCGGCAACGAAACCGACA 300

S8D11 GCTGCCGACTCAAATGCGGGAGATGCTAATGGGCTTGATGAAGCGGCAACGAAACCGACA 300

S12G7 GCTGCCGACTCAAATGCGGGAGATGCTAATGGGCTTGATGAAGCGGCAACGAAACCGACA 300

G33C1 GCTGCCGACTCAAA**C**GCGGGAGATGCTAATGGGCTTGATGAAGCGGCAACGAAACCGACA 300

G3B9 GCTGCCGACTCAAATGCGGGAGATGCTAATGGGCTTGATGAAGCGGCAACGAAACCGACA 300

L7F3 GCTGCCGACTCAAATGCGGGAGATGCTAATGGGCTTGATGAAGCGGCAACGAAACCGACA 300

L4B1 GCTGCCGACTCAAATGCGGGAGATGCTAATGGGCTTGATGAAGCGGCAACGAAACCGACA 300

G14G7 GCTGCCGACTCAAATGCGGGAGATGCTAATGGGCTTGATGAAGCGGCAACGAAACCGACA 300

L6B11 GCTGCCGACTCAAA---------TGCTAATGGGCTTGATGAAGC**C**GCAACGAAACCG**G**CA 291

L5A6 GCTGCCGACTCAAA---------TGCTAATGGGCTTGATGAAGC**C**GCAACGAAACCG**G**CA 291

L6D10 GCTGCCGACTCAAA---------TGCTAATGGGCTTGATGAAGC**C**GCAACGAAACCG**G**CA 291

L6F9 GCTGCCGACTCAAA---------TGCTAATGGGCTTGATGAAGC**C**GCAACGAAACCG**G**CA 291

S17H5 GCTGCCGACTCAAA**A**GCGGG**T**GATGCTAATGGGCTTGATGAAGC**C**GCAACGAAACCGACA 261

S11H2 GCTGCCGACTCAAA**A**GCGGG**T**GATGCTAATGGGCTTGATGAAGC**C**GCAACGAAACCGACA 300

S21F5 GCTGCCGACTCAAA**A**GCGGG**T**GATGCTAATGGGCTTGATGAAGC**C**GCAACGAAACCGACA 300

S18G6 GCTGCCGACTCAAA**A**GCGGG**T**GATGCTAATGGGCTTGATGAAGC**C**GCAACGAAACCGACA 300

S7C1 GCTGCCGACTCAAA**A**GCGGG**T**GATGCTAATGGGCTTGATGAAGC**C**GCAACGAAACCGACA 300

S13A12 GCTGCCGACTCAAA**A**GCGGG**T**GATGCTAATGGGCTTGATGAAGC**C**GC**G**ACGAAACCGACA 300

G21E12 GCCGCAAAAGGACCAAAGACGGCCGGAAAACCAGCTAAACCATCAAAGGCAAAACCAAAG 351

G13A12 GCCGCAAAAGGACCAAAGACGGCCGGAAAACCAGCTAAACCATCAAAGGCAAAACCAAAG 351

G11C1 GCCGCAAAAGGACCAAAGACGGCCGGAAAACCAGCTAAACCATCAAAGGCAAAACCAAAG 351

G13B3 GCCGCAAAAGGACCAAAG**C**CGGCCGGAAAACCAGCTAAACCATCAAAGGCAAAACCAAAG 351

L6A5 GCCGCAAAAGGACCAAAGACGGCCGGAAAACCAGCTAAACCATCAAAGGCAAAACCAAAG 351

G38A8 GCCGCAAAAGGACCAAAGACGGCCGGAAAACCAGCTAAACCATCAAAGGCAAAACCAAAG 349

G11B12 GCCGCAAAAGGACCAAAGACGGCCGGAAAACCAGCTAAACCATCAAAGGCAAAACCAAAG 351

G3H11 GCCGCAAAAGGACCAAAGACGGCCGGAAAACCAGCTAAACCATCAAAGGCAAAACCAAAG 351

G10A4 GCCGCAAAAGGACCAAAGACGGCCGGAAAACCAGCTAAACCATCAAAGGCAAAACCAAAG 351

L1F9 GCCGCAAAAGGACCAAAGACGGCCGGAAAACCAGCTAAACCATCAAAGGCAAAACCAAAG 351

G29E11 GCCGCAAAAGGACCAAAGACGGCCGGAAAACCAGCTAAACCATCAAAGGCAAAACCAAAG 351

L3A11 GCCGCAAAAGGACCAAAGACGGCCGGAAAACCAGCTAAACCATCAAAGGCAAAACCAAAG 351

L8C1 GCCGCAAAAGGACCAAAGACGGCCGGAAAACCAGCTAAACCATCAAAGGCAAAACCAAAG 351

G12B12 GCCGCAAAAGGACCAAAGACGGCCGGAAAACCAGCTAAACCATCAAAGGCAAAACCAAAG 351

S16B9 GCCGCAAAAGGACCAAAGACGGCCGGAAAACCAGCTAAACCATCAAAGGCAAAACCAAAG 359

L8A5 GCCGCAAAAGGACCAAAGACGGCCGGAAAACCAGCTAAACCATCAAAGGCAAAACCAAAG 360

G3D11 GCCGCAAAAGGACCAAAGACGGCCGGAAAACCAGCTAAACCATCAAAGGCAAAACCAAAG 360

G15B2 GCCGCAAAAGGACCAAAGACGGCCGGAAAACCAGCTAA**C**CCATCAAAGGCAAAACCAAAG 360

G17E10 GCCGCAAAAGGACCAAAGACGGCCGGAAAACCAGCTAA**C**CCATCAAAGGCAAAACCAAAG 360

G28C4 GCCGCAAAAGGACCAAAGACGGCCGGAAAACCAGCTAAACCATCAAAGGCAAAACCAAAG 360

G17B1 GCCGCAAAAGGACCAAAGACGGCCGGAAAACCAGCTAAACCATCAAAGGCAAAACCAAAG 360

G16F5 GCCGCAAAAGGACCAAAGACGGCCGGAAAACCAGCTAAACCATCAAAGGCAAAACCAAAG 360

G2B3 GCCG**T**AAAAGGACCAAAGACGGCCGGAAAACCAGCTAAACCATCAAAGGCAAAACCAAAG 360

G33D9 GCCGCAAAAGGACCAAAGACGGCCGGAAAACCAGCTAAACCATCAAAGGCAAAACCAAAG 360

G31A5 GCCGCAAAAGGACCAAA**C**ACGGCCGGAAAACCAGCTAAACCATCAAAGGCAAAACCAAAG 360

G8H5 GCCGCAAAAGGACCAAAGACGGCCGGAAAACCAGCTAAACCATCAAAGGCAAAACCAAAG 360

L2F2 GCCGCAAAAGGACCAAAGACGGCCGGAAAACCAGCTAAACCATCAAAGGCAAAACCAAAG 360

G4F2 GCCGCAAAAGGACCAAAGACGGCCGGAAAACCAGCTAAACCATCAAAGGCAAAACCAAAG 360

L3F1 GCCGCAAAAGGACCAAAGACGGCCGGAAAACCAGCTAAACCATCAAAGGCAAAACCAAAG 360

G36B7 GCCGCAAAAGGACCAAAGACGGCCGGAAAACCAGCTAAACCATCAAAGGCAAAACCAAAG 360

G18A2 GCCGCAAAAGGACCAAAGACGGCCGGAAAACCAGCTAAACCATCAAAGGCAAAACCAAAG 360

G38E8 GCCGCAAAAGGACCAAAGACGGCCGGAAAACCAGCTAAACCATCAAAGGCAAAACCAAAG 360

L3C5 GCCGCAAAAGGACCAAAGACGGCCGGAAAACCAGCTAAACCATCAAAGGCAAAACCAAAG 360

G36H7 GCCGCAAAAGGACCAAAGACGGCCGGAAAACCAGCTAAACCATCAAAGGCAAAACCAAAG 360

L1C6 GCCGCAAAAGGACCAAAGACGGCCGGAAAACCAGCTAAACCATCAAAGGCAAAACCAAAG 360

G17C9 GCCGCAAAAGGACCAAAGACGGCCGGAAAACCAGCTAAACCATCAAAGGCAAAACCAAAG 360

G12B5 GCCGCAAAAGGACCAAAGACGGCCGGAAAACCAGCTAAACCATCAAAGGCAAAACCAAAG 360

G21E5 GCCGCAAAAGGACCAAAGACGGCCGGAAAACCAGCTAAACCATCAAAGGCAAAACCAAAG 360

L3B1 GCCGCAAAAGGACCAAAGACGGCCGGAAAACCAGCTAAACCATCAAAGGCAAAACCAAAG 360

G8H12 GCCGCAAAAGGACCAAAGACGGCCGGAAAACCAGCTAAACCATCAAAGGCAAAACCAAAG 360

G10A9 GCCGCAAAAGGACCAAAGACGGCCGGAAAACCAGCTAAACCATCAAAGGCAAAACCAAAG 360

G3A11 GCCGCAAAAGGACCAAAGACGGCCGGAAAACCAGCTAAACCATCAAAGGCAAAACCAAAG 360

S10A3 GCCGCAAAAGGACCAAAGACGGCCGGAAAACCAGCTAAACCATCAAAGGCAAAACCAAAG 360

S4C1 GCCGCAAAAGGACCAAAGACGGCCGGAAAACCAGCTAAACCATCAAAGGCAAAACCAAAG 360

S10H9 GCCGCAAAAGGACCAAAGACGGCCGGAAAACCAGCTAAACCATCAAAGGCAAAACCAAAG 360

S10A11 GCCG**T**AAAAGGACCAAAGACGGCCGGAAAACCAGCTAAACCATCAAAGGCAAAACCAAAG 360

S10B5 GCCGCAAAAGGACCAAAGACGGCCGGAAAACCAGCTAAACCATCAAAGGCAAAACCAAAG 360

S2A12 GCCGCAAAAGGACCAAAGACGGCCGGAAAACCAGCTAAACCATCAAAGGCAAAACCAAAG 360

S13G9 GCCGCAAAAGGACCAAAGACGGCCGGAAAACCAGCTAAACCATCAAAGGCAAAACCAAAG 360

S8D11 GCCGCAAAAGGACCAAAGACGGCCGGAAAACCAGCTAAACCATCAAAGGCAAAACCAAAG 360

S12G7 GCCGCAAAAGGACCAAAGACGGCCGGAAAACCAGCTAAACCATC**G**AAGGCAAAACCAAAG 360

G33C1 GCCGCAAAAGGACCAAAGACGGCCGGAAAACCAGCTAAACCATCAAAGG**T**AAAACCAAAG 360

G3B9 GCCGCAAAAGGACCAAAGACGGCCGGAAAACCAGCTAAACCATCAAAGGCAAAACCAAAG 360

L7F3 GCCGCAAAAGGACCAAAGACGGCCGGAAAACCAGCTAAACCATCAAAGGCAAAACCAAAG 360

L4B1 GCCGCAAAAGGACCAAAGACGGCCGGAAAACCAGCTAAACCATCAAAGGCAAAACCAAAG 360

G14G7 GCCGCAAAAGGACCAAAGACGGCCGGAAAACCAGCTAAACCATCAAAGGCAAAACCAAAG 360

L6B11 GCCGCAAAAGGACCAAAGACGGCCGGAAAACCAGCTAAACCATCAAAGGCAAAACCAAAG 351

L5A6 GCCGCAAAAGGACCAAAGACGGCCGGAAAACCAGCTAAACCATCAAAGGCAAAACCAAAG 351

L6D10 GCCGCAAAAGGACCAAAGACGGCCGGAAAACCAGCTAAACCATCAAAGGCAAAACCAAAG 351

L6F9 GCCGCAAAAGGACCAAAGACGGCCGGAAAACCAGCTAAACCATCAAAGGCAAAACCAAAG 351

S17H5 GCCGCAAAAGGACCAAAGACGGC**T**GGAAAACCAGCTAAACCATCAAAGGCAAAACCAAAG 321

S11H2 GCCGCAAAAGGACCAAAGACGGC**T**GGAAAACCAGCTAAACCATCAAAGGCAAAACCAAAG 360

S21F5 GCCGCAAAAGGACCAAAGACGGC**T**GGAAAACCAGCTAAACCATCAAAGGCAAAACCAAAG 360

S18G6 GCCGCAAAAGGACCAAAGACGGC**T**GGAAAACCAGCTAAACCATCAAAGGCAAAACCAAAG 360

S7C1 GCCGCAAAAGGACCAAAGACGGC**T**GGAAAACCAGCTAAACCATCAAAGGCAAAACCAAAG 360

S13A12 GCCGCAAAAGGACCAAAGACGGC**T**GGAAAACCAGCTAAACCATCAAAGGCAAAACCAAAG 360

G21E12 AAGGCCGATTCACCAAAAAAA---GCAAAACCAGCCCCAAAGAAGAAG**TGA**TCATTTCAT 408

G13A12 AAGGCCGATTCACCAAAAAAA---GCAAAACCAGCCCCAAAGAAGAAG**TGA**TCATTTCAT 408

G11C1 AAGGCCGATTC**C**CCAAAAAAA---GCAAAACCAGCCCCAAAGAAGAAG**TGA**TCATTTCAT 408

G13B3 AAGGCCGATTCACCAAAAAAA---GCAAAACCAGCCCCAAAGAAGAAG**TGA**TCATTTCAT 408

L6A5 AAGGCCGATTCACCAAAAAAA**AAA**GCAAAACCAGCCCCAAAGAAGAAG**TGA**TCATTTCAT 411

G38A8 AAGGCCGATTCACCAAAAAAA---GCAAAACCAGCCCCAAAGAAGAAG**TGA**TCATTTCAT 406

G11B12 AAGGCCGATTCACCAAAAAAA---GCAAAACCAGCCCCAAAGAAGAAG**TGA**TCATTTCAT 408

G3H11 AAGGCCGATTCACCAAAAAAA---GCAAAACCAGCCCCAAAGAAGAAG**TGA**TCATTTCAT 408

G10A4 AAGGCCGATTCACCAAAAAAA---GCAAAACCAGCCCCAAAGAAGAAG**TGA**TCATTTCAT 408

L1F9 AAGGCCGATTCACCAAAAAAA---GCAAAACCAGCCCCAAAGAAGAAG**TGA**TCATTTCAT 408

G29E11 AAGGCCGATTCACCAAAAAAA---GCAAAACCAGCCCCAAAGAAGAAG**TGA**TCATTTCAT 408

L3A11 AAGGCCGATTC**G**CCAAAAAAA---GCAAAACCAGCCCCAAAGAAGAAG**TGA**TCATTTCAT 408

L8C1 AAGGCCGATTCACCAAAAAAA---GCAAAACCAGCCCCAAAGAAG**G**AG**TGA**TCATTTCAT 408

G12B12 AAGGCCGATTCACCAAAAAAA---GCAAAACCAGCCCCAAAGAAGAAG**TGA**TCATTTCAT 408

S16B9 AAGGCCGATTCACCAAAAAAA---GCAAAACCAGCCCCAAAGAAGAAG**TGA**TCATTTCAT 416

L8A5 AAGGCCGATTCACCAAAAAAA---GCAAAACCAGCCCCAAAGAAGAAG**TGA**TCATTTCAT 417

G3D11 AAGGCCGATTCACCAAAAAAA---GCAAAACCAGCCCCAAAGAAGAAG**TGA**TCATTTCAT 417

G15B2 AAGGCCGATTCACCAAAAAAA---GCAAAACCAGCCCCAAAGAAGAAG**TGA**TCATTTCAT 417

G17E10 AAGGCCGATTCACCAAAAAAA---GCAAAACCAGCCCCAAAGAAGAAG**TGA**TCATTTCAT 417

G28C4 AAGGCCGATTCACCAAAAAAA---GCAAAACCAGCCCCAAAGAAGAAG**TGA**TCATTTCAT 417

G17B1 AAGGCCGATTCACCAAAAAAA---GCAAAACCAGCCCCAAAGAAGAAG**TGA**TCATTTCAT 417

G16F5 AAGGCCGATTCACCAAAAAAA---GCAAAACCAGCCCCAAAGAAGAAG**TGA**TCATTTCAT 417

G2B3 AAGGCCGATTCACCAAAAAAA---GCAAAACCAGCCCCAAAGAAGAAG**TGA**TCATTTCAT 417

G33D9 AAGGCCGATTCACCAAAAAAA---GCAAAACCAGCCCCAAAGAAGAAG**TGA**TCATTTCAT 417

G31A5 AAGGCCGATTCACCAAAAAAA---GCAAAACCAGCCCCAAAGAAGAAG**TGA**TCATTTCAT 417

G8H5 AAGGCCGATTCACCAAAAAAA---GCAAAACCAGCCCCAAAGAAGAAG**TGA**TCATTTCAT 417

L2F2 AAGGCCGATTCACCAAAAAAA---GCAAAACCAGCCCCAAAGAAGAAG**TGA**TCATTTCAT 417

G4F2 AAGGCCGATTCACCAAAAAAA---GCAAAACCAGCCCCAAAGAAGAAG**TGA**TCATTTCAT 417

L3F1 AAGGCCGATTCACCAAAAAAA---GCAAAACCAGCCCCAAAGAAGAAG**TGA**TCATTTCAT 417

G36B7 AAGGCCGATTCACCAAAAAAA---GCAAAACCAGCCCCAAAGAAGAAG**TGA**TCATTTCAT 417

G18A2 AAGGCCGATTCACCAAAA**G**AA---GCAAAACCAGCCCCAAAGAAGAAG**TGA**TCATTTCAT 417

G38E8 AAGGCCGATTCACCAAAAAAA---GCAAAACCAGCCCCAAAGAAGAAG**TGA**TCATTTCAT 417

L3C5 AAGGCCGATTCACCAAAAAAA---GCAAAACCAGCCCCAAAGAAGAAG**TGA**TCATTTCAT 417

G36H7 AAGGCCGATTCACCAAAAAAA---GCAAAACCAGCCCCAAAGAAGAAG**TGA**TCATTTCAT 417

L1C6 AAGGCCGATTCACCAAAAAAA---GCAAAACCAGCCCCAAAGAAGAAG**TGA**TCATTTCAT 417

G17C9 AAGGCCGATTCACCAAAAAAA---GCAAAACCAGCCCCAAAGAAGAAG**TGA**TCATTTCAT 417

G12B5 AAGGCCGATTCACCAAAAAAA---GCAAAACCAGCCCCAAAGAAGAAG**TGA**TCATTTCAT 417

G21E5 AAGGCCGATTCACCAAAAAAA---GCAAAACCAGCCCCAAAGAAGAAG**TGA**TCATTTCAT 417

L3B1 AAGGCCGATTCACCAAAAAAA---GCAAAACCAGCCCCAAAGAAGAAG**TGA**TCATTTCAT 417

G8H12 AAGGCCGATTCACCAAAAAAA---GCAAAACCAGCCCCAAAGAAGAAG**TGA**TCATTTCAT 417

G10A9 AAGGCCGATTCACCAAAAAAA---GCAAAACCAGCCCCAAAGAAGAAG**TGA**TCATTTCAT 417

G3A11 AAGGCCGATTCACCAAAAAAA---GCAAAACCAGCCCCAAAGAAGAAG**TGA**TCATTTCAT 417

S10A3 AAGGCCGATTCACCAAAAAAA---GCAAAACCAGCCCCAAAGAAGAAG**TGA**TCATTTCAT 417

S4C1 AAGGCCGATTCACCAAAAAAA---GCAAAACCAGCCCCAAAGAAGAAG**TGA**TCATTTCAT 417

S10H9 AAGGCCGATTCACCAAAAAA**G**---GCAAAACCAGCCCCAAAGAAGAAG**TGA**TCATTTCAT 417

S10A11 AAGGCCGATTCACCAAAAAA**G**---GCAAAACCAGCCCCAAAGAAGAAG**TGA**TCATTTCAT 417

S10B5 AAGGCCGATTCACCAAAAAA**G**---GCAAAACCAGCCCCAAAGAAGAAG**TGA**TCATTTCAT 417

S2A12 AAGGCCGATTCACCAAAAAAA---GCAAAACCAGCCCCAAAGAAGAAG**TGA**TCATTTCAT 417

S13G9 AAGGCCGATTCACCAAAAAAA---GCAAAACCAGCCCCAAAGAAGAAG**TGA**TCATTTCAT 417

S8D11 AAGGCCGATTCACCAAAAAAA---GCAAAACCAGCCCCAAAGAAGAAG**TGA**T**T**ATTTCAT 417

S12G7 AAGGCCGATTCACCAAAAAAA---GCAAAACCAGCCCCAAAGAAGAAG**TGA**T**T**ATTTCAT 417

G33C1 AAGGCCGATTCACCAAAAAAA---GCAAAACCAGCCCCAAAGAAGAAG**TGA**TCAT**C**TCAT 417

G3B9 AAGGCCGATTCACCAAAAAAA---GCAAAACCAGCCCCAAAGAAGAAG**TGA**TCATTTCAT 417

L7F3 AAGGCCGATTCACCAAAAAAA---GCAAAACCAGCCCCAAAGAAGAAG**TGA**TCATTTCAT 417

L4B1 AAGGCCGATTCACCAAAAAAA---GCAAAACCAGCCCCAAAGAAGAAG**TGA**TCATTTCAT 417

G14G7 AAGGCCGATTCACCAAAAAAA---GCAAAACCAGCCCCAAAGAAGAAG**TGA**TCATTTCAT 417

L6B11 AAGGCCGATTCACCAAAAAAA---GCAAAACCAGCCCCAAAGAAGAAG**TGA**TCATTTCAT 408

L5A6 AAGGCCGATTC**G**CCAAAAAAA---GCAAAACCAGCCCCAAAGAAGAAG**TGA**TCATTTCAT 408

L6D10 AAGGCCGATTC**G**CCAAAAAAA---GCAAAACCAGCCCCAAAGAAGAAG**TGA**TCATTTCAT 408

L6F9 AAGGCCGATTCACCAAAAAAA---GCAAAACCAGCCCCAAAGAAGAAG**TGA**TCATTTCAT 408

S17H5 AAGGCCGATTCACCAAAAAAA---GCAAAACCAGC**T**CCAAAGAAGAAG**TGA**TCATTTCAT 378

S11H2 AAGGCCGATTCACCAAAAAAA---GCAAAACCAGC**T**CCAAAGAAGAAG**TGA**TCATTTCAT 417

S21F5 AAGGCCGATTCACCAAAAAAA---GCAAAACCAGC**T**CCAAAGAAGAAG**TGA**TCATTTCAT 417

S18G6 AAGGCCGATTCACCAAAAAAA---GCAAAACCAGC**T**CCAAAGAAGAAG**TGA**TCATTTCAT 417

S7C1 AAGGCCGATTCACCAAAAAAA---GCAAAACCAGC**T**CCAAAGAAGAAG**TGA**TCATTTCAT 417

S13A12 AAGGCCGATTCACCAAAAAAA---GCAAAACCAGC**T**CCAAAGAAGAAG**TGA**TCATTTCAT 417

G21E12 TCAATTGAAAGAACATTCGGAGACGTCGTGTAATCAAAATTAAATAGTTATACGCTCTCA 468

G13A12 TCAATTGAAAGA**C**CATTCGGAGACGTCGTGTAATCAAAATTAAATAGTTATACGCTCTCA 468

G11C1 TCAATTGAAAGAACATTCGGAGACGTCGTGTAATCAAAATTAAATAGTTATACGCTCTCA 468

G13B3 TCAATTGAAAGA**C**CATTCGGAGACGTCGTGTAATCAAAATTAAATAGTTATACGCTCTCA 468

L6A5 TCAATTGAAAGAACATTCGGAGACGTCGTGTAATCAAAATTAAATAGTTATACGCTCTCA 471

G38A8 TCAATTGAAAGAACATTCGGAGACGTCGTGTAATCAAAATTAAATAGTTATACGCTCTCA 466

G11B12 TCAATTGAAAGAACATTCGGAGACGTCGTGTAATCAAAATTAAATAGTTATACGCTCTCA 468

G3H11 TCAATTGAAAGAACATTC**A**GAG**G**CGTCGTGTAATCAAAATTAAATAGTTATACGCTCTCA 468

G10A4 TCAATTGAAAGAACATTCGGAGACGTC**A**TGTAATCAAAATTAAATAGTTATACGCTCTCA 468

L1F9 TCAATTGAAAGAACATTCGGAGACGTCGTGTAATCAAAATTAAATAGTTATACGCTCTCA 468

G29E11 TCAATTGAAAGAACATTCGGAGACGTCGTGTAATCAAAATTAAATAGTTATACGCTCTCA 468

L3A11 TCAATTGAAAGAACATTCGGAGACGTCGTGTAATCAAAATTAAATAGTTATACGCTCTCA 468

L8C1 TCAATTGAAAGAACATTCGGAGACGTCGTGTAATCAAAATTAAATAGTTATACGCTCTCA 468

G12B12 TCAATTGAAAGAACATTCGGAGACGTCGTGTAATCAAAATTAAATAGTTATACGCTCTCA 468

S16B9 TCAATTGAAAGAACATTCGGAGACGTC**A**TGTAATCAAAATTAAATAGTTATACGCTCTCA 476

L8A5 TCAATTGAAAGAACATTCGGAGACGTCGTGTAATCAAAATTAAATAGTTATACGCTCTCA 477

G3D11 TCAATTGAAAGAACATTCGGAGACGTCGTGTAATCAAAATTAAATAGTTATACGCTCTCA 477

G15B2 TCAATTGAAAGAACATTCGGAGACGTCGTGTAATCAAAATTAAATAGTTATACGCTCTCA 477

G17E10 TCAATTGAAAGA**C**CATTCGGAGACGTCGTGTAATCAAAATTAAATAGTTATACGCTCTCA 477

G28C4 TCAATTGAAAGAACATTCGGAGACGTCGTGTAATCAAAATTAAATAGTTATACGCTCTCA 477

G17B1 TCAATTGAAAGAACATTCGGAGACGTCGTGTAATCAAAATTAAATAGTTATACGCTCTCA 477

G16F5 TCAATTGAAAGAACATTCGGAGACGTCGTGTAATCAAAATTAAATAGTTATACGCTCTCA 477

G2B3 TCAATTGAAAGAACATTCGGAGACGTCGTGTAATCAAAATTAAATAGTTATACGCTCTCA 477

G33D9 TCAATTGAAAGAACATTCGGAGAC**C**TCGTGTAATCAAAATTAAATAGTTATACGCTCTCA 477

G31A5 TCAATTGAAAGAACATTCGGAGACGTCGTG**C**AATCAAAATTAAATAGTTATACGCTCTCA 477

G8H5 TCAATTGAAAGAACATTCGGAGACGTCGTGTAATCAAAATTAAATAGTTATACGCTCTCA 477

L2F2 TCAATTGAAAGAACATTCGGAGACGTCGTGTAATCAAAATTAAATAGTTATACGCTCTCA 477

G4F2 TCAATTGAAAGAACATTCGGAGACGTCGTGTAATCAAAATTAAATAGTTATACGCTCTCA 477

L3F1 TCAATTGAAAGAACATTCGGAGACGTCGTGTAATCAAAATTAAATAGTTATACGCTCTCA 477

G36B7 TCAATTGAAAGAACATTCGGAGACGTCGTGTAATCAAAATTAAATAGTTATACGCTCTCA 477

G18A2 TCAATTGAAAGAACATTCGGAGACGTCGTGTAATCAAAATTAAATAGTTATACGCTCTCA 477

G38E8 TCAATTGAAAGAACATTCGGAGACGTCGTGTAATCAAAATTAAATAGTTATACGCTCTCA 477

L3C5 TCAATTGAAAGAACATTCGGAGACGTCGTGTAATCAAAATTAAATAGTTATACGCTCTCA 477

G36H7 TCAATTGAAAGAACATTCGGAGACGTCGTGTAATCAAAATTAAATAGTTATACGCTCTCA 477

L1C6 TCAATTGAAAGAACATTCGGAGACGTCGTGTAATCAAAATTAAATAGTTATACGCTCTCA 477

G17C9 TCAATTGAAAGAACATTCGGAGACGTCGTGTAATCAAAATTAAATAGTTATACGCTCTCA 477

G12B5 TCAATTGAAAGAACATTCGGAGACGTCGTGTAATCAAAATTAAATAGTTATACGCTCTCA 477

G21E5 TCAATTGAAAGAACATTCGGAG**G**CGTCGTGTAATCAAAATTAAATAGTTATACGCTCTCA 477

L3B1 TCAATTGAAAGAACATTCGGAGACGTCGTGTAATCAAAATTAAATAGTTATACGCTCTCA 477

G8H12 TCAATTGAAAGAACATTCGGAGACGTCGTGTAATCAAAATTAAATAGTTATACGCTCTCA 477

G10A9 TCAATTGAAAGAACATTCGGAGACGTCGTGTAATCAAAATTAAATAGTTATACGCTCTCA 477

G3A11 TCAATTGAAAGAACATTCGGAGACGTCGTGTAATCAAAATTAAATAGTTATACGCTCTCA 477

S10A3 TCAATTGAAAGAACATT**T**GGAGACGTCGTGTAATCAAAATTAAATAGTTATACGCTCTCA 477

S4C1 TCAATTGAAAGAACATT**T**GGAGACGTCGTGTAATCAAAATTAAATAGTTATACGCTCTCA 477

S10H9 TCAATTGAAAGAACATT**T**GGAGACGTCGTGTAATCAAAATTAAATAGTTATACGCTCTCA 477

S10A11 TCAATTGAAAGAACATT**T**GGAGACGTCGTGTAATCAAAATTAAATAGTTATACGCTCTCA 477

S10B5 TCAATTGAAAGAACATT**T**GGAGAC**T**TCGTGTAATCAAAATTAAATAGTTATACGCTCTCA 477

S2A12 TCAATTGAAAGAACATT**T**GGAGACGTCGTGTAATCAAAATTAAATAGTTATACGCTCTCA 477

S13G9 TCAATTGAAAGAACATT**T**GGAGACGTCGTGTAATCAAAATTAAATAGTTATACGCTCTCA 477

S8D11 TCAATTGAAAGAACATTCGGAGACGTCGTGTAATCAAAATTAAATAGTTATACGCTCTCA 477

S12G7 TCAATTGAAAGAACATTCGGAGACGTCGTGTAATCAAAATTAAATAGTTATACGCTCTCA 477

G33C1 TCAATTGAAAGAACATTCGGAGAC**C**TCGTGTAATCAAAATTAAATAGTTATACGCTCTCA 477

G3B9 TCAATTGAAAGAACATTCGGAGACGTCGTGTAATCAAAATTAAATAGTTATACGCTCTCA 477

L7F3 TCAATTGAAAGAACATTCGGAGACGTC**A**TGTAACCAAAATTAAATAGTTATAC**A**CTCTCA 477

L4B1 TCAATTGAAAGAACATTCGGAGACGTCGTGTAATCAAAATTAAATAGTTATACGCTCTCA 477

G14G7 TCAATTGAAAGAACATTCGGAGACGTCGTGTAATCAAAATTAAATAGTTATACGCTCTCA 477

L6B11 TCAATTGAAAGAACATTCGGAGACGTCGTGTAATCAAAATTAAATAGTTATACGCTCTCA 468

L5A6 TCAATTGAAAGAACATTCGGAGACGTCGTGTAATCAAAATTAAATAGTTATACGCTCTCA 468

L6D10 TCAATTGAAAGAACATTCGGAGACGTCGTGTAATCAAAATTAAATAGTTATACGCTCTCA 468

L6F9 TCAATTGAAAGAACATTCGGAGACGTCGTGTAATCAAAATTAAATAGTTATACGCTCTCA 468

S17H5 TCAATTGAAAGAACATTCGGAGACGTCGTGTAATCAAAATTAAATAGTTATAC**A**CTCTCA 438

S11H2 TCAATTGAAAGAACATTCGGAGACGTCGTGTAATCAAAATTAAATAGTTATAC**A**CTCTCA 477

S21F5 TCAATTGAAAGAACATTCGGAGACGTCGTGTAATCAAAATTAAATAGTTATAC**A**CTCTCA 477

S18G6 TCAATTGAAAGAACATTCGGAGACGTCGTGTAATCAAAATTAAATAGTTATAC**A**CTCTCA 477

S7C1 TCAATTGAAAGAACATTCGGAGACGTCGTGTAATCAAAATTAAATAGTTATAC**A**CTCTCA 477

S13A12 TCAATTGAAAGAACATTCGGAGACGTCGTGTAATCAAAATTAAATAGTTATAC**A**CTCTCA 477

G21E12 TATTTTCAATTTGTC**C**ATTGAAATCAATTCTTGAGATTAAATTCTTGAATGT 520

G13A12 TATTTTCAATTTGTC**C**ATTGAAATCAATTCTTGAGATTAAATTCT------- 513

G11C1 TATTTTCAATTTGTC**C**ATTGAAATCAATTCTTGAGATTAAATTCTTG----- 515

G13B3 TATTTTCAATTTGTC**C**ATTGAAATCAATTCTTGAGATTAAATTCT------- 513

L6A5 TATTTTCAATTTGTC**C**ATTGAAATCAATTCTTGAGATTAAATTCTTG----- 518

G38A8 TATTTTCAATTTGTC**C**ATTGAAATCAATTCTTGAGATTAAATTCTTG----- 513

G11B12 TATTTTCAATTTGTC**C**ATTGAAATCAATTCTTGAGATTAAATTCTTG----- 515

G3H11 TATTTTCAATTTGTC**C**ATTGAAATCAATTCTTGAGATTAAATTCTTG----- 515

G10A4 TATTTTCAATTTGTC**C**ATTGAAATCAATTCTTGAGATTAAATTCTTGAATGT 520

L1F9 TATTTTCAATTTGTC**C**ATTGAAATCAATTCTTGAGATTAAATTCTTGAATGT 520

G29E11 TATTTTCAATTTGTC**C**ATTGAAATCAATTCTTGAGATTAAATTCTTGAAT-- 518

L3A11 TATTTTCAATTTGTC**C**ATTGAAATCAATTCTTGAGATTAAATTCTTG----- 515

L8C1 TATTTTCAATTTGTC**C**ATTGAAATCAATTCTTGAGATTAAATTCT------- 513

G12B12 TATTTTCAATTTGTC**C**ATTGAAATCAATTCTTGAGATTAAATTCTTGAATGT 520

S16B9 TATTTTCAATTTGTC**T**ATTGAAATCAATTCTTGAGATAAAATTCTTGAATG- 527

L8A5 TATTTTCAATTTGTCAATTGAAATCAATTCTTGAGATAAAATTCTTGAATGT 529

G3D11 TATTTTCAATTTGTC**T**ATTGAAATCAATTCTTGAGATAAAATTCTTGAATGT 529

G15B2 TATTTTCAATTTGTCAATTGAAATCAATTCTTGAGATAAAATTCTTGAATG- 528

G17E10 TATTTTCAATTTGTCAATTGAAATCAATTCTTGAGATAAAATTCTTGAAT-- 527

G28C4 TATTTTCAATTTGTCAATTGAAATCAATTCTTGAGATAAAATTCTTGAAT-- 527

G17B1 TATTTTCAATTTGTCAATTGAAATCAATTCTTGAGATAAAATTCTTGAAT-- 527

G16F5 TATTTTCAATTTG**G**CAATTGAAATCAATTCTTGAGATAAAATTCTTGAATG- 528

G2B3 TATTTTCAATTTG**G**CAATTGAAATCAATTCTTGAGATAAAATTCTTGA---- 525

G33D9 TATTTTCAATTTG**G**CAATTGAAATCAATTCTTGAGATAAAATTCTTGAATG- 528

G31A5 TATTTTCAATTTG**C**CAATTGAAATC**G**ATTCTTGAGATAAAATTC-------- 521

G8H5 TATTTTCAATTTGTCAATTGAAATCAATTCTTGAGATAAAATTCTTGAATGT 529

L2F2 TATTTTCAATTTGTCAATTGAAATCAATTCTTGAGATAAAATTCTTGAAT-- 527

G4F2 TATTTTCAATTTGTCAATTGAAATCAATTCTTGAGATAAAATTCTTGAAT-- 527

L3F1 TATTTTCAATTTGTCAATTGAAATCAATTCTTGAGATAAAATTCTTGAAT-- 527

G36B7 TATTTTCAATTTGTCAATTGAAATCAATTCTTGAGATAAAATTCTTGAAT-- 527

G18A2 TATTTTCAATTTGTCAATTGAAATCAATTCTTGAGATAAAATTCTTGAAT-- 527

G38E8 TATTTTCAATTTGTCAATTGAAATCAATTCTTGAGATAAAATTCTTGAAT-- 527

L3C5 TATTTTCAATTTGTCAATTGAAATCAATTCTTGAGATAAAATTCTTGAAT-- 527

G36H7 TATTTTCAATTTGTCAATTGAAATCAATTCTTGAGATAAAATTCTTGAAT-- 527

L1C6 TATTTTCAATTTGTCAATTGAAATCAATTCTTGAGATAAAATTCTTGAATG- 528

G17C9 TATTTTCAATTTGTCAATTGAAATCAATTCTTGAGATAAAATTCTTGAATG- 528

G12B5 TATTTTCAATTTGTCAATTGAAATCAATTCTTGAGATAAAATTCTTGAATG- 528

G21E5 TATTTTCAATTTGTCAATTGAAATCAATTCTTGAGATAAAATTCTTGAATG- 528

L3B1 TATTTTCAATTTGTCAATTGAAATCAATTCTTGAGATAAAATTCTTGAATGT 529

G8H12 TATTTTCAATTTGTCAATTGAAATCAATTCTTGAGATAAAATTCTTGAATGT 529

G10A9 TATTTTCAATTTGTCAATTGAAATCAATTCTTGAGATAAAATTCTTGAAT-- 527

G3A11 TATTTTCAATTTGTCAATTGAAATCAATTCTTGAGATAAAATT**T**TTGAATGT 529

S10A3 TATTTTCAATTTGTCAATTGAAATCAATTCTTGAGAT**T**AAATTCTT**A**AAT-- 527

S4C1 TATTTTCAATTTGTCAATTGAAATCAATTCTTGAGAT--------------- 514

S10H9 TATTTTCAATTTGTCAATTGAAATCAATTCTTGAGAT**T**AAATTCTT**A**AATG- 528

S10A11 TATTTTCAATTTGTCAATTGAAATCAATTCTTGAGAT**T**AAATTCTT**A**AAT-- 527

S10B5 TATTTTCAATTTGTCAATTGAAATCAATTCTTGAGAT**T**AAATTCTT------ 523

S2A12 TATTTTCAATTTGTCAATTGAAATCAATTCTTGAGAT**T**AAATTCTT------ 523

S13G9 TATTTTCAATTTGTCAATTGAAATCAATTCTTGAGAT**T**AAATTCTT**A**AATGT 529

S8D11 TATTTTCAATTTGTC**T**ATTGAAATCAATTCTTGAGAT**T**AAATTCTTGAAT-- 527

S12G7 TATTTTCAATTTGTC**T**ATTGAAATCAATTCTTGAGAT**T**AAATTCTTGAAT-- 527

G33C1 TATTTTCAATTTGTCAATTGAAAT**T**AATTCTTG------------------- 510

G3B9 TATTTTCAATTTGTCAATTGAAAT**T**AATTCTTG**G**GATAAAATTCTTGAATG- 528

L7F3 TA**AA**TTCAATTTGTCAATTGAAATCAATTCTTGAGAT**T**AAATTCTTGAATGT 529

L4B1 TATTTTCAATTTGTC**C**ATTGAAATCAATTCTTGAGAT**T**AAATTCTTGAAT-- 527

G14G7 TATTTTCAATTTGTC**C**ATTGAAATCAATTCTTGAGAT**T**AAATTCTTGAAT-- 527

L6B11 TATTTTCAATTTGTC**C**ATTGAAATCAATTCTTGAGAT**T**AAATTCTTG----- 515

L5A6 TATTTTCAATTTGTC**C**ATTGAAATCAATTCTTGAGAT**T**AAATTCTTG----- 515

L6D10 TATTTTCAATTTGTCAATTGAAATCAATTCTTGAGATAAAATTCT------- 513

L6F9 TATTTTCAATTTGTC**C**ATTGAAATCAATTCTTGAGAT**T**AAAT---------- 510

S17H5 TATTTTCAATTTGTC**T**ATTGAAATCAATTCTTGAGAT**T**AAATTCTTGAATGT 490

S11H2 TATTTTCAATTTGTC**T**ATTGAAATCAATTCTTGAGAT**T**AAATTCTTGAATGT 529

S21F5 TATTTTCAATTTGTC**T**ATTGAAATCAATTCTTGAGAT**T**AAATTCTTGAATGT 529

S18G6 TATTTTCAATTTGTC**T**ATTGAAATCAATTCTTGAGAT**T**AAATTCTTGAAT-- 527

S7C1 TATTTTCAATTTGTC**T**ATTGAAATCAATTCTTGAGAT**T**AAATTCTTGAA--- 526

S13A12 TATTTTCAATTTGTC**T**ATTGAAATCAATTCTTGAGAT**T**AAATTCTTGAAT-- 527

**C**

S13F8 CAGTTC**A**A**T**T**C**GAACCGTTAATTCTTTCAATATCCACTGGAACATCCAAAAACGAAA**ATG** 60

S20F10 CAGTTC**A**A**T**T**C**GAACCGTTAATTCTTTCAATATCCACTGGAACATC**T**AAAAACGAAA**ATG** 60

S15G1 CAGTT**TG**AATTGAACCGTTAATTCTTTCAATATCCACTGGAACATCCAAAAACGAAA**ATG** 60

S4C7 CAGTT**TG**AATTGAACCGTTAATTCTTTCAATATCCACTGGAACATCCAAAAACGAAA**ATG** 60

S21E3 CAGTT**TG**AATTGAACCGTTAATTCTTTCAATATCCACTGGAACATCCAAAAACGAAA**ATG** 60

S6A3 CAGTTC**G**AATTGAACCGTTAATTCTTTCAATATCCACTGGAACATCCAAAAACGAAA**ATG** 60

G2H7 CAGTTCCAATTGAACCGTT**T**ATTCTTTC**G**ATATCCACTGGAACATCCAAAAACGAAA**ATG** 60

G13A10 CAGTTCCAATTGAACCGTT**T**ATTCTTTC**G**ATATCCACTGGAACATCCAAAAACGAAA**ATG** 60

G9G12 CAGTTCCAATTGAACCGTT**T**ATTCTTTC**G**ATATCCACTGGAACATCCAAAAACGAAA**ATG** 60

G5E9 CAGTTCCAATTGAACCGTT**T**ATTCTTTC**G**ATATCCACTGGAACATCCAAAAACGAAA**ATG** 60

G19D10 CAGTTCCAATTGAACCGTT**T**ATTCTTTC**G**ATATCCACTGGAACATCCAAAAACGAAA**ATG** 60

L6G6 CAGTTCCAATTGAACCGTTAATTCTTTCAATATCCACTGGAACATCCAAAAACGAAA**ATG** 60

L3B2 CAGTTCCAATTGAACCGTTAATTCTTTCAATATCCACTGGAACATCCAAAAACGAAA**ATG** 60

G2H2 CAGTTCCAATTGAACCGTTAATTCTTTCAATATCCACTGGAACATCCAAAAACGAAA**ATG** 60

G20C6 CAGTTCCAATTGAACCGTTAATTCTTTCAATATCCACTGGAACATCCAAAAACGAAA**ATG** 60

G21G4 CAGTTCCAATTGAACCGTTAATTCTTTCAATATCCACTGGAACATCCAAAAACGAAA**ATG** 60

G16E2 CAGTTCCAATTGAACCGTTAATTCTTTCAATATCCACTGGAACATCCAAAAACGAAA**ATG** 60

G32F7 CAGTTC**A**A**T**T**C**GAACC**A**TTAATTCTTTCAATATCCACT**A**GAACATCCAAAAACGAAA**ATG** 60

G32H6 CAGTTC**A**A**T**T**C**GAACC**A**TTAATTCTTTCAATATCCACT**A**GAACATCCAAAAACGAAA**ATG** 60

G5D12 CAGTTC**A**A**T**T**C**GAACC**A**TTAATTCTTTCAATATCCACT**A**GAACATCCAAAAACGAAA**ATG** 60

S13F8 TCAAAATTTTTATTAGCTTTCGCCGTCATCGC**T**GTCTGCCTTGTTGCAGCTCAGGCT**G**-- 118

S20F10 TCAAAATTTTTATTAGCTTTCGCCGTCATCGC**T**GTCTGCCTTGTTGCAGCTCAGGCT**G**-- 118

S15G1 TCAAAATTTTTATTAGCTTTCGCCGTCATCGC**T**GTCTGCCTTGTTGCAGCTCAGGCT**G**-- 118

S4C7 TCAAAATTTTTATTAGCTTTCGCCGTCATCGC**T**GTCTGCCTTGTTGCAGCTCAGGCT**G**-- 118

S21E3 TCAAAATTTTTATTAGCTTTCGCCGTCATCGC**T**GTCTGCCTTGTTGCAGCTCAGGCT**G**-- 118

S6A3 TCAAAATTTTTATTAGCTTTCGCCGTCATCGC**T**GTCTGCCTTGTTGCAGCTCAGGCT**GCT** 120

G2H7 TCAAAATTTTT**C**TTAGCTTTCGCC**A**TCATCGCCGTCTGCCTTGTTGCAGCTCAGGCT**G**-- 118

G13A10 TCAAAATTTTT**C**TTAGCTTTCGCC**A**TCATCGCCGTCTGCCTTGTTGCAGCTCAGGCT**G**-- 118

G9G12 TCAAAATTTTT**C**TTAGCTTTCGCC**A**TCATCGCCGTCTGCCTTGTTGCAGCTCAGGCT**G**-- 118

G5E9 TCAAAATTTTT**C**TTAGC**C**TTCGCC**A**TCATCGCCGTCTGCCTTGTTGCAGCTCAGGCT**G**-- 118

G19D10 TCAAAATTTTT**C**TTAGCTTTCGCC**A**TCATCGCCGTCTGCCTTGTTGCAGCTCAGGCT**G**-- 118

L6G6 TCAAAATTTTT**C**TTA**A**CTTTCGCCGTCATCGCCGTCTGCCTTGTTGCAGCTCAGGCT**G**-- 118

L3B2 TCAAAATTTTT**C**TTAGCTTTCGCCGTCATCGCCGTCTGCCTTGTTGCAGCTCAGGCT**G**-- 118

G2H2 TCAAAATTTTT**C**TTAGCTTTCGCCGTCATCGCCGTCTGCCTTGTTGCAGCTCAGGCT**G**-- 118

G20C6 TCAAAATTTTTA**C**TAGCTTTCGCC**A**TCATCGCCGTCTGCCTTGTTGCAGCTCAGGCT**G**-- 118

G21G4 TCAAAATTTTTA**C**TAGCTTTCGCC**A**TCATCGCCGTCTGCCTTGTTGCAGCTCAGGCT**G**-- 118

G16E2 TCAAAATTTTTA**C**TAGCTTTCGCC**A**TCATCGCCGTCTGCCTTGTTGCAGCTCAGGCT**G**-- 118

G32F7 TC**G**AAATTTTT**GC**TAGCTTTCGCCGTCATCGCCGTCTGCCTTGTTGCAGCTCAGGCT**G**-- 118

G32H6 TC**G**AAATTTTT**GC**TAGCTTTCGCCGTCATCGCCGTCTGCCTTGTTGCAGCTCAGGCT**G**-- 118

G5D12 TCAAAATTTTT**GC**TAGCTTTCGCCGTCATCGCCGTCTGCCTTGTTGCAGCTCAGGCT**G**-- 118

S13F8 -TACACAGCAGCCATTCCACAGCTGCACACGCTGGTAAAACCCATGATCCAGCAAAAACG 177

S20F10 -TACACAGCAGCCATTCCACAGCTGCACACGCTGGTAAAACCCATGATCCAGCAAAAACG 177

S15G1 -TACACAGCAGCCATTCCACAGCTGCACACGCTGGTAAAACCCATGATCCAGCAAAAACG 177

S4C7 -TACACAGCAGCCATTCCACAGCTGCACACGCTGGTAAAACCCATGATCCAGCAAAAACG 177

S21E3 -TACACAGCAGCCATTCCACAGCTGCACACGCTGGTAAAACCCATGATCCAGCAAAAACG 177

S6A3 **G**TACACAGCAGCCA**C**TCCACAGCTGCACACGCTGGTAAAACCCATGATCCAGCAAAAACG 180

G2H7 -TACACAGCAGCCATTCCACAGCTGCACACGCTGGTAAAACCCATGA**G**CCAGCAAAAACG 177

G13A10 -TACACAGCAGCCATTCCACAGCTGCACACGCTGGTAAAACCCATGA**G**CCAGCAAAAACG 177

G9G12 -TACACAGCAGCCATTCCACAGCTGCACACGCTGGTAAAACCCATGA**G**CCAGCAAAAACG 177

G5E9 -TACACAGCAGCCATTCCACAGCTGCACACGCTGGTAAAACCCATGA**G**CCAGCAAAAACG 177

G19D10 -TACACAGCAGCCATTCCACAGCTGCACACGCTGGTAAAACCCATGA**G**CCAGCAAAAACG 177

L6G6 -TACACAGCAGCCATTCCACAGCTGCACACGCTGGTAAAACCCATGATCCAGCAAAAACG 177

L3B2 -TACACAGCAGCCATTCCACAGCTGCACACGCTGGTAAAACCCATGATCCAGCAAAAACG 177

G2H2 -TACACAGCAGCCATTCCACAGCTGCACACGCTGGTAAAACCCATGATCCAGCAAAAACG 177

G20C6 -TACACAGCAGCCATTCCACAGCTGCACAC**A**CTGGTAAAACCCATGATCCAGCAAAAACG 177

G21G4 -TACACAGCAGCCATTCCACAGCTGCACACGCTGGTAAAACCCATGATCCAGCAAAAACG 177

G16E2 -TACACAGCAGCCATTCCACAGCTGCACACGCTGGTAAAACCCATGATCCAGCAAAAACG 177

G32F7 -TACACAGCAGCCA**C**TCCACAGCTGCACACGCTGGTAAAACCCATGATCCAGCAAAAACG 177

G32H6 -TACACAGCAGCCA**C**TCCACAGCTGCACACGCTGGTAAAACCCATGATCCAGCAAAAACG 177

G5D12 -TACACAGCAGCCA**C**TCCACAGCTGCACACGCTGGTAAAACCCATGATCCAGCAAAAACG 177

S13F8 CAAACAGCCCAACCAGGCCTAGGTGACCATCTTGACGATGGTGAGCACTATTTCGACGCA 237

S20F10 CAAACAGCCCAACCAGGCCTAGGTGACCATCTTGACGATGGTGAGCACTATTTCGACGCA 237

S15G1 CAAACAGCCCAACCAGGCCTAGGTGACCATCTTGACGATGGTGAGCACTATTTCGACGCA 237

S4C7 CAAACAGCCCAACCAGGCCTAGGTGACCATCTTGACGATGGTGAGCACTATTTCGACGCA 237

S21E3 CAAACAGCCCAACCAGGCCTAGGTGACCATCTTGACGATGGTGAGCACTATTTCGACGCA 237

S6A3 CAAACAGCCCAACCAGGCCTAGGTGACCATCTTGACGATGGTGAGCACTATTTCGACGCA 240

G2H7 CAAACAGCCCAACCAGGC**G**TAGGTGA**T**CATCTTGACGATGG**G**GAGCACTATTTCGACGCA 237

G13A10 CAAACAGCCCAACCAGGC**G**TAGGTGA**T**CATCTTGACGATGGTGAGCACTATTTCGACGCA 237

G9G12 CAAACAGCCCAACCAGGC**G**TAGGTGA**T**CATCTTGACGATGGTGAGCACTATTTCGACGCA 237

G5E9 CAAACAGCCCAACCAGGC**G**TAGGTGA**T**CATCTTGACGATGGTGAGCACTATTTCGACGCA 237

G19D10 CAAACAGCCCAACCAGGC**G**TAGGTGA**T**CATCTTGACGATGGTGAGCACTATTTCGACGCA 237

L6G6 CAAACAGCCCAACCAGGC**G**TAGGTGA**T**CATCT**C**GACGATGGTGAGCACTATTTCGACGCA 237

L3B2 CAAACAGCCCAACCAGGC**G**TAGGTGA**T**CATCTTGACGATGGTGAGCACTATTTCGACGCA 237

G2H2 CAAACAGCCCAACCAGGC**G**TAGGTGA**T**CATCTTGACGATGGTGAGCACTATTTCGACGCA 237

G20C6 CAAACAGCCCAACCAGGCCTAGGTGACCATCTTGACGATGGTGAGCACTATTTCGACGCA 237

G21G4 CAAACAGCCCAACCAGGCCTAGGTGACCATCTTGACGATGGTGAGCAC**C**ATTTCGACGCA 237

G16E2 CAAACAGCCCAACCAGGCCTAGGTGACCATCTTGACGATGGTGAGCACTATTTCGACGCA 237

G32F7 CAAACAGCCCAAC**T**A**A**GCCTAG**C**TGAC**G**ATCTTGACGA**A**G**A**TGAGCACTATTTCGACGCA 237

G32H6 CAAACAGCCCAAC**T**A**A**GCCTAG**C**TGAC**G**ATCTTGACGA**A**G**A**TGAGCACTATTTCGACGCA 237

G5D12 CAAACAGCCCAAC**T**A**A**GCCTAG**C**TGAC**G**ATCTTGACGA**A**G**A**TGAGCACTATTTCGACGCA 237

S13F8 TCGGACAAAGATTTCGAATCATATTTCGAAGATGATAATGAGCCTAATGAAGCAGCCGTA 297

S20F10 TCGGACAAAGATTTCGAATCATATTTCGAAGATGATAATGAGCCTAATGAAGCAGCCGTA 297

S15G1 TCGGACAAAGATTTCGAATCATATTTCGAAGATGATAATGAGCCTAATGAAGCAGCCGTA 297

S4C7 TCGGACAAAGATTTCGAATCATATTTCGAAGATGATAATGAGCCTAATGAAGCAGCCGTA 297

S21E3 TCGGACAAAGATTTCGAATCATATTTCGAAGATGATAATGAGCCTAATGAAGCAGCCGTA 297

S6A3 TCGGACAAAGATTTCGAATCATATTTCGAAGATGATAATGAGCCTAATGAAGCAGCCGTA 300

G2H7 TCGGACA**G**AGATTTCGAATCATATTTCGAAGATGATAATGAGCCTAATGAAGCAGCCGTA 297

G13A10 TCGGACAAAGATTTCGAATCATATTTCGAAGATGATAATGAGCCTAATGAAGCAGCCGTA 297

G9G12 TCGGACAAAGATTTCGAATCATATTTCGAAGATGATAATGAGCCTAATGAAGCAGCCGTA 297

G5E9 TCGGACAAAGATTTCGAATCATATTTCGAAGATGATAATGAGCCTAATGAAGCAGCCGTA 297

G19D10 TCGGACAAAGATTTCGAATCATATTTCGAAGATGATAATGAGCCTAATGAAGCAGCCGTA 297

L6G6 TCGGACAAAGATTTCGAATCATATTTCGAAGATGATAATGAGCCTAATGAAGCAGCCGTA 297

L3B2 TCGGACAAAGATTTCGAATCATATTTCGAAGATGATAATGAGCCTAATGAAGCAGCCGTA 297

G2H2 TCGGACAAAGATTTCGAATCATATTTCGAAGATGATAATGAGCCTAATGAAGCAGCCGTA 297

G20C6 TCGGACAAAGATTTCGAATCATATTTCGAAGATG**G**TAATGAGCCTAATGAAGCAGCCGTA 297

G21G4 TCGGACAAAGATTTCGAATCATATTTCGAAGATG**G**TAATGAGCCTAATGAAGCAGCCGTA 297

G16E2 TCGGACAAAGATTTCGAATCATATTTCGAAGATG**G**TAATGAGCCTAATGAAGCAGCCGTA 297

G32F7 TCGGACAAAGATTTCGAATCATATTTCGA**G**GATGATAATGAGCCTAATGAAGCAGCCGTA 297

G32H6 TCGGACAAAGATTTCGAATCATATTTCGA**G**GATGATAATGAGCCTAATGAAGCAGCCGTA 297

G5D12 TCGGACAAAGATTTCGAATCATATTTCGA**G**GATGATAATGAGCCTAATGAAGCAGCCGTA 297

S13F8 CCGGCAGGAAAACCAACAAAACCA**ACAAAACCA**TTGAAGGCCAAAACATCGCCAAA**T**GGA 357

S20F10 CCGGCAGGAAAACCAACAAAACCA**ACAAAACCA**TTGAAGGCCAAAACATCGCCAAA**T**GGA 357

S15G1 CCGGCAGGAAAACCAACAAAACCA**ACAAAACCA**TTGAAGGCCAAAACATCGCCAAA**T**GGA 357

S4C7 CCGGCAGGAAAACCAACAAAACCA**ACAAAACCA**TTGAAGGCCAAAACATCGCCAAA**T**GGA 357

S21E3 CCGGCAGGAAAACCAACAAAACCA**ACAAAACCA**TTGAAGGCCAAAACATCGCCAAA**T**GGA 357

S6A3 CCGGCAGGAAAACCAACAAAACCA**ACAAAACCA**TTGAAGGCCAAAACATCGCCAAA**T**GGA 360

G2H7 CCGGCAGGAAAACCAACAAAACCA---------TTGAAGGCCAAAACATCGCCAAAAGGA 348

G13A10 CCGGCAGGAAAACCAACAAAACCA---------TTGAAGGCCAAAACATCGCCAAAAGGA 348

G9G12 CCGGCAGGAAAACCAACAAAACCA---------TTGAAGGCCAAAACATCGCCAAAAGGA 348

G5E9 CCGGCAGGAAAACCAACAAAACCA---------TTGAAGGCCAAAACATCGCCAAAAGGA 348

G19D10 CCGGCAGGAAAACCAACAAAACCA---------TTGAAGGCCAAAACATCGCCAAAAGGA 348

L6G6 CCGGCAGGAAAACCAACAAAACCA---------TTGAAGGCCAAAACATCGCCAAAAGGA 348

L3B2 CCGGCAGGAAAACCAACAAAACCA---------TTGAAGG**A**CAAAACATCGCCAAAAGGA 348

G2H2 CCGGCAGGAAAACCAACAAAACCA---------TTGAAGGCCAAAACATCGCCAAAAGGA 348

G20C6 CCGGCAGGAAAACCAACAAAACCA---------TTGAAGGCCAAAACATCGCCAAAA**AA**A 348

G21G4 CCGGCAGGAAAACCAACAAAACCA---------TTGAAGGCCAAAACATCGCCAAAA**AA**A 348

G16E2 CCGGCAGGAAAACCAACAAAACCA---------TTGAAGGCCAAAACATCGCCAAAA**AA**A 348

G32F7 CCGGCAGGAAAACCAACAAAACCA---------TTGAAGGCCAAAACATCGCCAAAA**AA**A 348

G32H6 CCGGCAGGAAAACCAACAAAACCA---------TTGAAGGCCAAAACATCGCCAAAA**AA**A 348

G5D12 CCGGCAGGAAAACCAACAAAACCA---------TTGAAGGCCAAAACATCGCCAAAA**AA**A 348

S13F8 AAAGGCCACCAAAAAAAG**TAA**TCATTTCATTCAATTGAAAGAACATTTGGAGGCGTCG**CG** 417

S20F10 AAAGGCCACCAAAAAAAG**TAA**TCATTTCATTCAATTGAAAGAACATTTGGAGGCGTCG**CG** 417

S15G1 AAAGGCCACCAAAAAAAG**TAA**TCATTTCATTCAATTGAAAGAACATTTGGAGGCGTCG**CG** 417

S4C7 AAAGGCCACCAAAAAAAG**TAA**TCATTTCATTCAATTGAAAGAACATTTGGAGGCGTCG**CG** 417

S21E3 ATAGGCCACCAAAAAAAG**TAA**TCATTTCATTCAATTGAAAGAACATTTGGAGGCGTCG**CG** 417

S6A3 AAAGGCCACCAAAAAAAG**TAA**TCATTTCATTCAATTGAAAGAACATTTGGAGGCGTCG**CG** 420

G2H7 AAAGGCCACCAAAAAAAA**TGA**TCATTTCATTCAATTGAAAGAACATTTGGAGGCGTCG**CG** 408

G13A10 AAAGGCCACCAAAAAAAA**TGA**TCATTTCATTCAATTGAAAGAACATTTGGAGGCGTCG**CG** 408

G9G12 AAAGGCCACCAAAAAAAA**TGA**TCATTTCATTCAATTGAAAGAACATTTGGAGGCGTCG**CG** 408

G5E9 AAAGGCCACCAAAAAAAA**TGA**TCATTTCATTCAATTGAAAGAACATTTGGAGGCGTCG**CG** 408

G19D10 AAAGGCC**C**C**A**AAAAAAAA**TGA**TCATTTCATTCAATTGAAAGAACATTTGGAGGCGTCG**CG** 408

L6G6 AAAGGCCACCAAAAAAAA**TGA**TCATTTCATTCAATTG**G**AAGAACA**A**TTGGAGGC**A**TCG-- 406

L3B2 AAAGGCCACCAAAAAAAA**TGA**TCATTTCATTCAATTG**G**AAGAACATTTGGAGGC**A**TCG-- 406

G2H2 AAAGGCCACCAAAAAAAA**TGA**TCATTTCATTCAATTG**G**AAGAACATTTGGAGGC**A**TCG-- 406

G20C6 **TC**AG**C**C**TCAA**A**G**AA**G**AA**GTGA**TCATTTCATTCAATTG**G**AAGAACATTTGGAGGC**A**TCG-- 406

G21G4 **TC**AG**C**C**TCAA**A**G**AA**G**AA**GTGA**TCATTTCATTCAATTG**G**AAGAACATTTGGAGGC**A**TCG-- 406

G16E2 **TC**AG**C**C**TCAA**A**G**AA**G**AA**GTGA**TCATTTCATTCAATTG**G**AAGAACATTTGGAGGC**A**TCG-- 406

G32F7 **TC**AG**C**C**TCAA**A**G**AA**G**AA**GTGA**T**T**ATTTCATTCAATTGAAAGAACATTTTGAGGCG**A**CG-- 406

G32H6 **TC**AG**C**C**TCAA**A**G**AA**G**AA**GTGA**TTATTTCATTCAATTGAAAGAACATTTTGAGGCG**A**CG-- 406

G5D12 **TC**AG**C**C**TCAA**A**G**AA**G**AA**GTGA**TCATTTCATTCAATTGAAAGAACATTTTGAGGCG**A**CG-- 406

S13F8 TGTAA**T**CAAAATTATATAGTTATAC**G**CTCTCATATTTTCAATTTGTCAATTGAAATCAAT 477

S20F10 TGTAA**T**CAAAATTATATAGTTATAC**G**CTCTCATATTTTCAATTTGTCAATTGAAATCAAT 477

S15G1 TGTAA**T**CAAAATTATATAGTTATAC**G**CTCTCATATTTTCAATTTGTCAATTGAAATCAAT 477

S4C7 TGTAA**T**CAAAATTATATAGTTATAC**G**CTCTCATATTTTCAATTTGTCAATTGAAATCAAT 477

S21E3 TGTAA**T**CAAAATTATATAGTTATAC**G**CTCTCATATTTTCAATTTGTCAATTGAAATCAAT 477

S6A3 TGTAA**T**CAAAATTATATAGTTATAC**G**CTCTCATATTTTCAATTTGTCAATTGAAATCAAT 480

G2H7 TGTAACCAAAATTATATAGTTATACACTCTCATATTTTCAATTTGT-------------- 454

G13A10 TGTAACCAAAATTATATAGTTATACACTCTCATATTTTCAATTTGCCTATTGAAATCAAT 468

G9G12 TGTAACCAAAATTATATAGTTATACACTCTCATATTTTCAATTTGTCTATTGAAATCAAT 468

G5E9 TGTAACCAAAATTATATAGTTATACACTCTCATATTTTCAATTTGTCTATTGAAATCAAT 468

G19D10 TGTAACCAAAATTATATAGTTATAC**C**CTCTCATATTTTCAATTTGTCTATTGAAATCAAT 468

L6G6 TGTAACCAAAATTA**A**ATAGTTATACACTCTCATATTTTCAATTTGTCAATTGAAATCAAT 466

L3B2 TGTAACCAAAATTA**A**ATAGTTATACACTCTCATATTTTCAATTTGTCAATTGAAATCAAT 466

G2H2 TGTAACCAAAATTA**A**ATAGTTATACACTCTCATATTTTCAATTTGTCAATTGAAATCAAT 466

G20C6 TGTAACCAAAATTA**A**ATAGTTATACACTCTCATA**AA**TTCAATTTGTCAATTGAAATCAAT 466

G21G4 TGTAACCAAAATTA**A**ATAGTTATACACTCTCATA**AA**TTCAATTTGTCAATTGAAATCAAT 466

G16E2 TGTAACCAAAATTA**A**ATAGTTATACACTCTCATA**AA**TTCAATTTGTCAATTGAAATCAAT 466

G32F7 TGTAA**T**CAAAATTA**A**ATAGTTATACACTCTCATATTTTCAATTTGTCTATTGAAATCAAT 466

G32H6 TGTAA**T**CAAAATTA**A**ATAGTTATACACTCTCATATTTTCAATTTGCCTATTGAAATCAAT 466

G5D12 TGTAA**T**CAAAATTA**A**ATAGTTATACACTCTCATATTTTCAATTTGTCTATTGAAATCAAT 466

S13F8 TCTTGAAATTAAATTCTTGAATGT 501

S20F10 TCTTGAAATTAAATTCTTG----- 496

S15G1 TCTTGAAATTAAATTCTTGAATGT 501

S4C7 TCTTGAAATTAAAT**C**CTTGAAT-- 499

S21E3 TCTTGAAATTAAATTCTTGAATG- 500

S6A3 TCTTGAAATTAAATTCTTGAATGT 504

G2H7 ------------------------

G13A10 TCTTGAAATTAAAT**C**CTTGAAT-- 490

G9G12 TCTTGAAATTAAATTCTTG----- 487

G5E9 TCTTGAAATTAAATTCTTGAAT-- 490

G19D10 TCTTGAAATTAAATTCTTGAATGT 492

L6G6 TCTTGAAATTAAATTCTTGAAT-- 488

L3B2 TCTTGAAATTAAATTCTTG----- 485

G2H2 TCTTGAAATTAAATTCTTG----- 485

G20C6 TCTTGAAATTAAATTCTTGAATG- 489

G21G4 TCTTGAAATTAAATTCTTGAATG- 489

G16E2 TCTTGAAATTAAATTCTTGAAT-- 488

G32F7 TCTTGAAATTAAATTCTTGAATG- 489

G32H6 TCTTGAAATTAAATTCTTGAATG- 489

G5D12 TCTTGAAATTAAATTCTTGAATG- 489

Figure S6. Sequence alignments of cDNAs corresponding to *SSSGP-1A* (A), *SSSGP-1B1* (B), and *SSSGP-1C1* (C). Nonsynonymous substitutions are in red while synonymous substitutions are in blue. Substitutions outside of the coding regions are in green. Indels are bold. ‘ATG’ start codon, start position of MPCR, and stop codon are bold and underlined.
