# Supplementary material for: Unusual conservation among genes encoding small secreted salivary gland proteins from a gall midge
Source: BMC Evol Biol. 2010 Sep 28;10:296. doi: 10.1186/1471-2148-10-296 (PMC2955719; doi:10.1186/1471-2148-10-296)
Supplement: Additional file 7 — Figure S7: Sequence alignment of cDNAs encoding ribosomal proteins. [file 1471-2148-10-296-S7.DOC]

**A**

G1G9 ---AACACCAATACGGGCAACAACTCAAATATAGAATAATATCAGCGAACCTGATTTAAA 57

GW13D6 ------------------AACAACTCAAATATAGAATAATATCAGCGAACCTGATTTAAA 42

GW18C7 ------------------AACAACTCAAATATAGAATAATATCAGCGAACCTGATTTAAA 42

GW14A6 -----------------------CTCAAATATAGAATAATATCAGCGAACCTGATTTAAA 37

L18C2 -GAAACACCAACACGGGCAACAACTCAAATATAGAATAATATCAGCGAACCTGATTTAAA 59

GW8A12 -----------------CAACAACTCAAATATAGAATAATATCAGCGAACCTGATTTAAA 43

S14C7 TGAAACACCAATACGGGCAACAACTCAAATATAGAATAATATCAGCGAACCTGATTTAAA 60

G1G9 **ATG**GGTAAATACGCACGTGAAGCAGATAATGCAGCCAAATCCTGCAAATCCCGTGGATCA 117

GW13D6 **ATG**GGTAAATACGCACGTGAAGCAGATAATGCAGCCAAATCCTGCAAATCCCGTGGATCA 102

GW18C7 **ATG**GGTAAATACGCACGTGAAGCAGATAATGCAGCCAAATCCTGCAAATC**T**CGTGGATCA 102

GW14A6 **ATG**GGTAAATACGCACGTGAAGCAGATAATGCAGCCAAATCCTGCAAATC**T**CGTGGATCA 97

L18C2 **ATG**GGTAAATACGCACGTGAAGCAGATAATGCAGCCAAATCCTGCAAATC**T**CGTGGATCA 119

GW8A12 **ATG**GGTAAATACGCACGTGAAGCAGATAATGCAGCCAAATCCTGCAAATCCCGTGGATCA 103

S14C7 **ATG**GGTAAATACGCACGTGAAGCAGATAATGCAGCCAAATCCTGCAAATCCCGTGGATCA 120

G1G9 CATCTTCGTGTCCACTTTAAGAACACCCATGAAGCGGCTCAAGCCATCAAGCATATGCCA 177

GW13D6 CATCTTCGTGTCCACTTTAAGAACACCCATGAAGCGGCTCAAGCCATCAAGCATATGCCA 162

GW18C7 CATCTTCGTGTCCACTTTAAGAACACCCATGAAGCGGCTCAAGCCATCAAGCATATGCCA 162

GW14A6 CATCTTCGTGTCCACTTTAAGAACACCCATGAAGCGGCTCAAGCCATCAAGCATATGCCA 157

L18C2 CATCTTCGTGTCCACTTTAAGAACACCCATGAAGCGGCTCAAGCCATCAAGCATATGCCA 179

GW8A12 CATCTTCGTGTCCACTTTAAGAACACCCATGAAGCGGCTCAAGCCATCAAGCATATGCCA 163

S14C7 CATCTTCGTGTCCACTTTAAGAACACCCATGAAGCGGCTCAAGCCATCAAGCATATGCCA 180

G1G9 TTGCGTCGTGCTCAACGTTTTTTGAAAAACGTTGTTGACAAGAAAGAATGTGTTCCATTT 237

GW13D6 TTGCGTCGTGCTCAACGTTTTTTGAAAAACGTTGTTGACAAGAAAGAATGTGTTCCATTT 222

GW18C7 TTGCGTCGTGCTCAACGTTTTTTGAAAAACGTTGTTGACAAGAAAGAATGTGTTCCATTT 222

GW14A6 TTGCGTCGTGCTCAACGTTTTTTGAAAAACGTTGTTGACAAGAAAGAATGTGTTCCATTT 217

L18C2 TTGCGTCGTGCTCAACGTTTTTTGAAAAACGTTGTTGACAAGAAAGAATGTGTTCCATTT 239

GW8A12 TTGCGTCGTGCTCAACGTTTTTTGAAAAACGTTGTTGACAAGAAAGAATGTGTTCCATTT 223

S14C7 TTGCGTCGTGCTCAACGTTTTTTGAAAAACGTTGTTGACAAGAAAGAATGTGTTCCATTT 240

G1G9 CGTAAATTTAATGGTGGTGTTGGCCGTTGTGCACAAGCAAAACAATGGGGAACCACACAA 297

GW13D6 CGTAAATTTAATGGTGGTGTTGGCCGTTGTGCACAAGCAAAACAATGGGGAACCACACAA 282

GW18C7 CGTAAATTTAATGGTGGTGTTGGCCGTTGTGCACAAGCAAAACAATGGGGAACCACACAA 282

GW14A6 CGTAAATTTAATGGTGGTGTTGGCCGTTGTGCACAAGCAAAACAATGGGGAACCACACAA 277

L18C2 CGTAAATTTAATGGTGGTGTTGGCCGTTGTGCACAAGCAAAACAATGGGGAACCACACAA 299

GW8A12 CGTAAATTTAATGGTGGTGTTGGCCGTTGTGCACAAGCAAAACAATGGGGAACCACACAA 283

S14C7 CGTAAATTTAATGGTGGTGTTGGCCGTTGTGCACAAGCAAAACAATGGGGAACCACACAA 300

G1G9 GGACGTTGGCCAAAAAAATCGGCCGATTTCTTGTTGCAATTGTTGAAAAATGCCGAATCA 357

GW13D6 GGACGTTGGCCAAAAAAATCGGCCGATTTCTTGTTGCAATTGTTGAAAAATGCCGAATCA 342

GW18C7 GGACGTTGGCCAAAAAAATCGGCCGATTTCTTGTTGCAATTGTTGAAAAATGCCGAATCA 342

GW14A6 GGACGTTGGCCAAAAAAATCGGCCGATTTCTTGTTGCAATTGTTGAAAAATGCCGAATCA 337

L18C2 GGACGTTGGCCAAAAAAATCGGCCGATTTCTTGTTGCAATTGTTGAAAAATGCCGAATCA 359

GW8A12 GGACGTTGGCCAAAAAAATCGGCCGATTTCTTGTTGCAATTGTTGAAAAATGCCGAATCA 343

S14C7 GGACGTTGGCCAAAAAAATCGGCCGATTTCTTGTTGCAATTGTTGAAAAATGCCGAATCA 360

G1G9 AATGCCGAATACAAAGGTTTGGACGTTGATCGACTTGTCATCGATCACATTCAAGTAAAC 417

GW13D6 AATGCCGAATACAAAGGTTTGGACGTTGATCGACTTGTCATCGATCACATTCAAGTAAAC 402

GW18C7 AATGCCGAATACAAAGGTTTGGACGTTGATCGACTTGTCATCGATCACATTCAAGTAAAC 402

GW14A6 AATGCCGAATACAAAGGTTTGGACGTTGATCGACTTGTCATCGATCACATTCAAGTAAAC 397

L18C2 AATGCCGAATACAAAGGTTTGGACGTTGATCGACTTGTCATCGATCACATTCAAGTAAAC 419

GW8A12 AATGCCGAATACAAAGGTTTGGACGTTGATCGACTTGTCATCGATCACATTCAAGTAAAC 403

S14C7 AATGCCGAATACAAAGGTTTGGACGTTGATCGACTTGTCATCGATCACATTCAAGTAAAC 420

G1G9 CGTGCTCCATGCCTTCGTCGTCGTACATACCGTGCTCATGGTCGTATCAACCCATACATG 477

GW13D6 CGTGCTCCATGCCTTCGTCGTCGTACATACCGTGCTCATGGTCGTATCAACCCATACATG 462

GW18C7 CGTGCTCCATGCCTTCGTCGTCGTACATACCGTGCTCATGGTCGTATCAACCCATACATG 462

GW14A6 CGTGCTCCATGCCTTCGTCGTCGTACATACCGTGCTCATGGTCGTATCAACCCATACATG 457

L18C2 CGTGCTCCATGCCTTCGTCGTCGTACATACCGTGCTCATGGTCGTATCAACCCATACATG 479

GW8A12 CGTGCTCCATGCCTTCGTCGTCGTACATACCGTGCTCATGGTCGTATCAACCCATACATG 463

S14C7 CGTGCTCCATGCCTTCGTCGTCGTACATACCGTGCTCATGGTCGTATCAACCCATACATG 480

G1G9 TCGTCACCATGCCACATCGAAGTCATCCTTTCAGAGAAAGAAGATGTTGTTGCGAAAGTT 537

GW13D6 TCGTCACCATGCCACATCGAAGTCATCCTTTCAGAGAAAGAAGATGTTGTTGCGAAAGTT 522

GW18C7 TCGTCACCATGCCACATCGAAGTCATCCTTTCAGAGAAAGAAGATGTTGTTGCGAAAGTT 522

GW14A6 TCGTCACCATGCCACATCGAAGTCATCCTTTCAGAGAAAGAAGATGTTGTTGCGAAAGTT 517

L18C2 TCGTCACCATGCCACATCGAAGTCATCCTTTCAGAGAAAGAAGATGTTGTTGCGAAAGTT 539

GW8A12 TCGTCACCATGCCACATCGAAGTCATCCTTTCAGAGAAAGAAGATGTTGTTGCGAAAGTT 523

S14C7 TCGTCACCATGCCACATCGAAGTCATCCTTTCAGAGAAAGAAGATGTTGTTGCGAAAGTT 540

G1G9 AATGACGATGAACCATCAAAGAAAAAGCTTTCCAAAAAGAAGTTGCAACGTCAAAAAGAA 597

GW13D6 AATGACGATGAACCATCAAAGAAAAAGCTTTCCAAAAAGAAGTTGCAACGTCAAAAAGAA 582

GW18C7 AATGACGATGAACCATCAAAGAAAAAGCTTTCCAAAAAGAAGTTGCAACGTCAAAAAGAA 582

GW14A6 AATGACGATGAACCATCAAAGAAAAAGCTTTCCAAAAAGAAGTTGCAACGTCAAAAAGAA 577

L18C2 AATGACGATGAACCATCAAAGAAAAAGCTTTCCAAAAAGAAGTTGCAACGTCAAAAAGAA 599

GW8A12 AATGACGATGAACCATCAAAGAAAAAGCTTTCCAAAAAGAAGTTGCAACGTCAAAAAGAA 583

S14C7 AATGACGATGAACCATCAAAGAAAAAGCTTTCCAAAAAGAAGTTGCAACGTCAAAAAGAA 600

G1G9 AAAATGATGCGCAATGAA**TAA**ACGGAACG**GTT**TTTTTCTTTAAAAAAACAATTTTAAGTA 657

GW13D6 AAAATGATGCGCAATGAA**TAA**ACGGAACG**GTT**TTTTTCTTTAAAAAAACAATTTTAAGTA 642

GW18C7 AAAATGATGCGCAATGAA**TAA**ACGGAACG**GTT**TTTTTCTTTAAAAAAACAATTTTAAGTA 642

GW14A6 AAAATGATGCGCAATGAA**TAA**ACGGAACG**GTT**TTTTTCTTTAAAAAAACAATTTTAAGTA 637

L18C2 AAAATGATGCGCAATGAA**TAA**ACGGAACG**GTT**TTTTTCTTTAAAAAAACAATTTTAAGTA 659

GW8A12 AAAATGATGCGCAATGAA**TAA**ACGGAACG**GTT**TTTTTCTTTAAAAAAACAATTTTAAGTA 643

S14C7 AAAATGATGCGCAATGAA**TAA**ACGGAACG---TTTTT**T**TTTAAAAAAACAATTT**G**AAGTA 657

G1G9 TGAAGTTAACAGCATTCATATGTGAAAATAAATATAAATCATAATGTTTCTATGATTT-- 715

GW13D6 TGAAGTTAACAGCATTCATATGTGAAAATAAATATAAATCATAATGTTTC---------- 692

GW18C7 TGAAGTTAACAGCATTCATATGTGAAAATAAATATAAATCATAATGTTTCTATGATTTG- 701

GW14A6 TGAAGTTAACAGCATTCATATGTGAAAATAAATATAAATCATAATGTTTCTATGATTTG- 696

L18C2 TGAAGTTAACAGCATTCATATGTGAAAATAAATATAAATCATAATGTTTCTATGATTTGA 719

GW8A12 TGAAGTTAACAGCATTCATATGTGAAAATAAATATAAATCATAATGTTTCTATGATTTG- 702

S14C7 TGAAGTTAACAGCATTCATATGTGAAAATAAATATAAATCATAATGT**C**TCTATGATTTGG 717

G1G9 ----------------------

GW13D6 ----------------------

GW18C7 ----------------------

GW14A6 ----------------------

L18C2 AAAAGATAAAGTAATTAAAAAC 741

GW8A12 ----------------------

S14C7 ----------------------

**B**

G8C9 ---------------------------TCTCCTTCAAAT**ATG**GGTATTGACATTTGTCAT 33

GW20B6 CAAAGCTAAGTGAAACGTCCGGTCTTTTCTCCTTCAAAT**ATG**GGTATTGACATTTGTCAT 60

G8B11 -----------------------------TCCTTCAAAT**ATG**GGTATTGACATTTGTCAT 31

GW19G5 -------AAGTGAAACGTCCGGTCTTTTCTCCTTCAAAT**ATG**GGTATTGACATTTGTCAT 53

GW13B7 -----------------------------------------------TGACATTTGTCAT 13

S17F4 ------------------------------------------------------------

G8C9 AAATACGACCGAAAGGTCCGTCGTACGGAACCTAAGAGCCAAGATGTTTACTTGCGACTT 93

GW20B6 AAATACGACCGAAAGGTCCGTCGTACGGAACCTAAGAGCCAAGATGTTTACTTGCGACTT 120

G8B11 AAATACGACCGAAAGGTCCGTCGTACGGAACCTAAGAGCCAAGATGTTTACTTGCGACTT 91

GW19G5 AAATACGACCGAAAGGTCCGTCGTACGGAACCTAAGAGCCAAGATGTTTACTTGCGACTT 113

GW13B7 AAATACGACCGAAAGGTCCGTCGTACGGAACCTAAGAGCCAAGATGTTTACTTGCGACTT 73

S17F4 ------------------------------------------------------------

G8C9 CTTGTTAAGTTGTACCGTTTCCTGTACAGACGTACATACAAAAAGTTTAACAAAATAAT**A** 153

GW20B6 CTTGTTAAGTTGTACCGTTTCCTGTACAGACGTACATACAAAAAGTTTAACAAAATAATT 180

G8B11 CTTGTTAAGTTGTACCGTTTCCTGTACAGACGTACATACAAAAAGTTTAACAAAATAAT**A** 151

GW19G5 CTTGTTAAGTTGTACCGTTTCCTGTACAGACGTACATACAAAAAGTTTAACAAAATAATT 173

GW13B7 CTTGTTAAGTTGTACCGTTTCCTGTACAGACGTACATACAAAAAGTTTAACAAAATAATT 133

S17F4 ------------------------------------------------------------

G8C9 TTGAAACGTTTGTTCATGAGCCGCATCAATCAACCACCAATGTCATTGCAACGAGTGGTT 213

GW20B6 TTGAAACGTTTGTTCATGAGCCGCATCAATCAACCACCAATGTCATTGCAACGAGTGGTT 240

G8B11 TTGAAACGTTTGTTCATGAGCCGCATCAATCAACCACCAATGTCATTGCAACGAGTGGTT 211

GW19G5 TTGAAACGTTTGTTCATGAGCCGCATCAATCAACCACCAATGTCATTGCAACGAGTGGTT 233

GW13B7 TTGAAACGTTTGTTCATGAGCCGCATCAATCAACCACCAATGTCATTGCAACGAGTGGTT 193

S17F4 ------------------------------------------------------------

G8C9 CGTTTGTTGAAAAAAAGCGGCAAAGAAGAAAAAATTGTCTGCGTTGTTGGTACAGTAACA 273

GW20B6 CGTTTGTTGAAAAAAAGCGGCAAAGAAGAAAAAATTGTCTGCGTTGTTGGTACAGTAACA 300

G8B11 CGTTTGTTGAAAAAAAGCGGCAAAGAAGAAAAAATTGTCTGCGTTGTTGGTACAGTAACA 271

GW19G5 CGTTTGTTGAAAAAAAGCGGCAAAGAAGAAAAAATTGTCTGCGTTGTTGGTACAGTAACA 293

GW13B7 CGTTTGTTGAAAAAAAGCGGCAAAGAAGAAAAAATTGTCTGCGTTGTTGGTACAGTAACA 253

S17F4 ------------------GGCAAAGAAGAAAAAATTGTCTGCGTTGTTGGTACAGTAACA 42

G8C9 GACGATGCACGTTTGTTAAATGTACCAAAATTGAAATTGTG**T**GCACTTCGCGTTACCGAA 333

GW20B6 GACGATGCACGTTTGTTAAATGTACCAAAATTGAAATTGTGCGCACTTCGCGTTACCGAA 360

G8B11 GACGATGCACGTTTGTTAAATGTACCAAAATTGAAATTGTG**T**GCACTTCGCGTTACCGAA 331

GW19G5 GACGATGCACGTTTGTTAAATGTACCAAAATTGAAATTGTGCGCACTTCGCGTTACCGAA 353

GW13B7 GACGATGCACGTTTGTTAAATGTACCAAAATTGAAATTGTGCGCACTTCGCGTTACCGAA 313

S17F4 GACGATGCACGTTTGTTAAATGTACCAAAATTGAAATTGTGCGCACT**C**CG**T**GTTACCGAA 102

G8C9 AAGGCACGTGGACGTATCTTGGCCGCTGGTGGTGAGGTCATCACCTTCGATCAATTGGCT 393

GW20B6 AAGGCACGTGGACGTATCTTGGCCGCTGGTGGTGAGGTCATCACCTTCGATCAATTGGCT 420

G8B11 AAGGCACGTGGACGTATCTTGGCCGCTGGTGGTGAGGTCATCACCTTCGATCAATTGGCT 391

GW19G5 AAGGCACGTGGACGTATCTTGGCCGCTGGTGGTGAGGTCATCACCTTCGATCAATTGGCT 413

GW13B7 AAGGCACGTGGACGTATCTTGGCCGCTGGTGGTGAGGTCATCACCTTCGATCAATTGGCT 373

S17F4 AAGGCACGTGGACGTATCTTGGCCGCTGGTGGTGAGGTCATCAC**A**TTCGATCAATTGGCT 162

G8C9 ATCCGTTCACCAACGGGCAAAGGTACATTGTTGATCCAAGGCAAACGTACCGCTCGTGTT 453

GW20B6 ATCCGTTCACCAACGGGCAAAGGTACATTGTTGATCCAAGGCAAACGTACCGCTCGTGTT 480

G8B11 ATCCGTTCACCAACGGGCAAAGGTACATTGTTGATCCAAGGCAAACGTACCGCTCGTGTT 451

GW19G5 ATCCGTTCACCAACGGGCAAAGGTACATTGTTGATCCAAGGCAAACGTACCGCTCGTGTT 473

GW13B7 ATCCGTTCACCAACGGGCAAAGGTACATTGTTGATCCAAGGCAAACGTACCGCTCGTGTT 433

S17F4 ATCCGTTCACCAACGGGCAAAGGTACATTGTTGATCCAAGGCAAACGTACCGCTCGTGTT 222

G8C9 GCCAACAAACATTTCGGTAAAGCTCCAGGTGTTCCACATTCTCACACCCGACCATATGTC 513

GW20B6 GCCAACAAACATTTCGGTAAAGCTCCAGGTGTTCCACATTCTCACACCCGACCATATGTC 540

G8B11 GCCAACAAACATTTCGGTAAAGCTCCAGGTGTTCCACATTCTCACACCCGACCATATGTC 511

GW19G5 GCCAACAAACATTTCGGTAAAGCTCCAGGTGTTCCACATTCTCACACCCGACCATATGTC 533

GW13B7 GCCAACAAACATTTCGGTAAAGCTCCAGGTGTTCCACATTCTCACACCCGACCATATGTC 493

S17F4 GCCAACAAACATTTCGGTAAAGCTCCAGGTGTTCCACATTCTCACACCCGACCATATGTC 282

G8C9 CGATCAAAGGGACGCAAATTCGAACGTGCTCGTGGTCGTCGCAGTGGTTGCGGTTACAAA 573

GW20B6 CGATCAAAGGGACGCAAATTCGAACGTGCTCGTGGTCGTCGCAGTGGTTGCGGTTACAAA 600

G8B11 CGATCAAAGGGACGCAAATTCGAACGTGCTCGTGGTCGTCGCAGTGGTTGCGGTTACAAA 571

GW19G5 CGATCAAAGGGACGCAAATTCGAACGTGCTCGTGGTCGTCGCAGTGGTTGCGGTTACAAA 593

GW13B7 CGATCAAAGGGACGCAAATTCGAACGTGCTCGTGGTCGTCGCAGTGGTTGCGGTTACAAA 553

S17F4 CGATCAAAGGGACGCAAATTCGAACGTGCTCGTGGTCGTCGCAGTGGTTGCGGTTACAAA 342

G8C9 AAA**TAA**ACTGGCTGATCATATCAATCAGTTTTTACATAGATTGATTACGCTGTATCATCT 633

GW20B6 AAA**TAA**ACTGGCTGATCATATCAATCAGTTTTTACATAGATTGATTACGCTGTATCATCT 660

G8B11 AAA**TAA**ACTGGCTGATCATATCAATCAGTTTTTACATAGATTGATTACGCTGTATCATCT 631

GW19G5 AAA**TAA**ACTGGCTGATCATATCAATCAGTTTTTACATAGATTGATTACGCTGTATCATCT 653

GW13B7 AAA**TAA**ACTGGCTGATCATATCAATCAGTTTTTACATAGATTGATTACGCTGTATCATCT 613

S17F4 AAA**TAA**ACTGGCTGATCATATCAATCAGTTTTTACATAGATTGAT**C**ACGCTGTATCATCT 402

G8C9 GGCGATTTTCAATGTTAAAACAACATTTTTGGTGTAAAACTATGTTAATTACAATGAA-- 691

GW20B6 GGCGATTTTCAATGTTAAAACAACATTTTTGGTGTAAAACTATGTTAATTACAATGAAGT 720

G8B11 GGCGATTTTCAATGTTAAAACAACATTTTTGGTGTAAAACTATGTTAATTACAATGAAGT 691

GW19G5 GGCGATTTTCAATGTTAAAACAACATTTTTGGTGTAAAACTATGTTAATTACAATGAAGT 713

GW13B7 GGCGATTTTCAATGTTAAAACAACATTTTTGGTGTAAAACTATGTTAATTACAATGAAGT 673

S17F4 GGCGATTTTCAATGTTAAAACAACATTTTTGGTGTAAAACTATGTTAATTACAATGAAGT 462

G8C9 ------------------------------------------------------------

GW20B6 TTTATTTGTAATGTTTACGAAATGAGAATAAACTCAATGGAACTTTAACATC-------- 772

G8B11 TTTATTTGTAATGTTTACGAAATGAGAATAAACTCAATGGAACTTTATCATCAA------ 745

GW19G5 TTTATTTGTAATGTTTACGAAATGAGAATAAACTCAATGGAACTTTAACATCAAAC---- 769

GW13B7 TTTATTTGTAATGTTTACGAAATGAGAATAAACTCAATGGAACTTTAACATCAAAC---- 729

S17F4 TTTATTTGTAATGTTTACGAAATGAGAATAAACTCAATGGAACTTTAACATC-------- 513

**C**

G26A7 ------------------------------AAAA**ATG**AAGGCTAAGGGACAGTTGAGAGA 30

GW14H9 -----------------------------CAAAA**ATG**AAGGCTAAGGGACAGTTGAGAGA 31

GW3D10 ---------------------------AGCAAAA**ATG**AAGGCTAAGGGACAGTTGAGAGA 33

S17E4 -------------------------ACAGCAAAA**ATG**AAGGCTAAGGGACAGTTGAGAGA 35

L4C16 ------------ACAGTATCGAAACACAGCAAAA**ATG**AAGGCTAAGGGACAGTTGAGAGA 48

GW7A2 TAAATATTTAACACAGTATCGAAACACAGCAAAA**ATG**AAGGCTAAGGGACAGTTGAGAGA 60

GW11G11 ------------ACAGTATCGAAACACAGCAAAA**ATG**AAGGCTAAGGGACAGTTGAGAGA 48

G26A7 ATACGAAGTTATTGGCCGTAAGTTGCCAACCGAAAAGGAACCATCCACTCCATTGTACAA 90

GW14H9 ATACGAAGTTATTGGCCGTAAGTTGCCAACCGAAAAGGAACCATCCACTCCATTGTACAA 91

GW3D10 ATACGAAGTTATTGGCCGTAAGTTGCCAACCGAAAAGGAACCATCCACTCCATTGTACAA 93

S17E4 ATACGAAGTTATTGGCCGTAAGTTGCCAACCGAAAAGGAACCATCCACTCCATTGTACAA 95

L4C16 ATACGAAGTTATTGGCCGTAAGTTGCCAACCGAAAAGGAACCATCCACTCCATTGTACAA 108

GW7A2 ATACGAAGTTATTGGCCGTAAGTTGCCAACCGAAAAGGAACCATCCACTCCATTGTACAA 120

GW11G11 ATACGAAGTTATTGGCCGTAAGTTGCCAACCGAAAAGGAACCATCCACTCCATTGTACAA 108

G26A7 GATGCGAATTTTCGCACCAGACAACATTGTGGCTAAATCCCGTTTTTGGTATTTTTTGCG 150

GW14H9 GATGCGAATTTTCGCACCAGACAACATTGTGGCTAAATCCCGTTTTTGGTATTTTTTGCG 151

GW3D10 GATGCGAATTTTCGCACCAGACAACATTGTGGCTAAATCCCGTTTTTGGTATTTTTTGCG 153

S17E4 GATGCGAATTTTCGCACCAGACAACATTGTGGCTAAATCCCGTTTTTGGTATTTTTTGCG 155

L4C16 GATGCGAATTTTCGCACCAGACAACATTGTGGCTAAATCCCGTTTTTGGTATTTTTTGCG 168

GW7A2 GATGCGAATTTTCGCACCAGACAACATTGTGGCTAAATCCCGTTTTTGGTATTTTTTGCG 180

GW11G11 GATGCGAATTTTCGCACCAGACAACATTGTGGCTAAATCCCGTTTTTGGTATTTTTTGCG 168

G26A7 TCAATTGAAAAAATTTAAGAAGACCACCGGTGAAATCGTTTCATTGAAACAAGTCTATGA 210

GW14H9 TCAATTGAAAAAATTTAAGAAGACCACCGGTGAAATCGTTTCATTGAAACAAGTCTATGA 211

GW3D10 TCAATTGAAAAAATTTAAGAAGACCACCGGTGAAATCGTTTCATTGAAACAAGTCTATGA 213

S17E4 TCAATTGAAAAAATTTAAGAAGACCACCGGTGAAATCGTTTCATTGAAACAAGTCTATGA 215

L4C16 TCAATTGAAAAAATTTAAGAAGACCACCGGTGAAATCGTTTCATTGAAACAAGTCTATGA 228

GW7A2 TCAATTGAAAAAATTTAAGAAGACCACCGGTGAAATCGTTTCATTGAAACAAGTCTATGA 240

GW11G11 TCAATTGAAAAAATTTAAGAAGACCACCGGTGAAATCGTTTCATTGAAACAAGTCTATGA 228

G26A7 AACTTCACCCACCAAAATCAAGAATTTTGGTATCTGGTTGCGGTACGATTCACGTTCCGG 270

GW14H9 AACTTCACCCACCAAAATCAAGAATTTTGGTATCTGGTTGCGGTACGATTCACGTTCCGG 271

GW3D10 AACTTCACCCACCAAAATCAAGAATTTTGGTATCTGGTTGCGGTACGATTCACGTTCCGG 273

S17E4 AACTTCACCCACCAAAATCAAGAATTTTGGTATCTGGTTGCGGTACGATTCACGTTCCGG 275

L4C16 AACTTCACCCACCAAAATCAAGAATTTTGGTATCTGGTTGCGGTACGATTCACGTTCCGG 288

GW7A2 AACTTCACCCACCAAAATCAAGAATTTTGGTATCTGGTTGCGGTACGATTCACGTTCCGG 300

GW11G11 AACTTCACCCACCAAAATCAAGAATTTTGGTATCTGGTTGCGGTACGATTCACGTTCCGG 288

G26A7 TACACACAACATGTACCGTGAATATCGTGACTTGACCGTTGGTGATGCTGTTACACAATG 330

GW14H9 TACACACAACATGTACCGTGAATATCGTGACTTGACCGTTGGTGATGCTGTTACACAATG 331

GW3D10 TACACACAACATGTACCGTGAATATCGTGACTTGACCGTTGGTGATGCTGTTACACAATG 333

S17E4 TACACACAACATGTACCGTGAATATCGTGACTTGACCGTTGGTGATGCTGTTACACAATG 335

L4C16 TACACACAACATGTACCGTGAATATCGTGACTTGACCGTTGGTGATGCTGTTACACAATG 348

GW7A2 TACACACAACATGTACCGTGAATATCGTGACTTGACCGTTGGTGATGCTGTTACACAATG 360

GW11G11 TACACACAACATGTACCGTGAATATCGTGACTTGACCGTTGGTGATGCTGTTACACAATG 348

G26A7 TTACCGTGATATGGGTGCTCGTCATCGTGCTCGTGCTCATTCAATCCAA**G**TCATCAAAGT 390

GW14H9 TTACCGTGATATGGGTGCTCGTCATCGTGCTCGTGCTCATTCAATCCAAATCATCAAAGT 391

GW3D10 TTACCGTGATATGGGTGCTCGTCATCGTGCTCGTGCTCATTCAATCCAAATCATCAAAGT 393

S17E4 TTACCGTGATATGGGTGCTCGTCATCGTGCTCGTGCTCATTCAATCCAAATCATCAAAGT 395

L4C16 TTACCGTGATATGGGTGCTCGTCATCGTGCTCGTGCTCATTCAATCCAAATCATCAAAGT 408

GW7A2 TTACCGTGATATGGGTGCTCGTCATCGTGCTCGTGCTCATTCAATCCAAATCATCAAAGT 420

GW11G11 TTACCGTGATATGGGTGCTCGTCATCGTGCTCGTGCTCATTCAATCCAAATCATCAAAGT 408

G26A7 TGAGCCAATTGCAGCCAACAAATGCCGCCGTACTCATGTCACACAGTTCCATGATGCAAA 450

GW14H9 TGAGCCAATTGCAGCCAACAAATGCCGCCGTACTCATGTCACACAGTTCCATGATGCAAA 451

GW3D10 TGAGCCAATTGCAGCCAACAAATGCCGCCGTACTCATGTCACACAGTTCCATGATGCAAA 453

S17E4 TGAGCCAATTGCAGCCAACAAATGCCGCCGTACTCATGTCACACAGTTCCATGATGCAAA 455

L4C16 TGAGCCAATTGCAGCCAACAAATGCCGCCGTACTCATGTCACACAGTTCCATGATGCAAA 468

GW7A2 TGAGCCAATTGCAGCCAACAAATGCCGCCGTACTCATGTCACACAGTTCCATGATGCAAA 480

GW11G11 TGAGCCAATTGCAGCCAACAAATGCCGCCGTACTCATGTCACACAGTTCCATGATGCAAA 468

G26A7 GATTCGTTTCCCACTCGTCCAACGAGTACACCATAGAGGATCACGGGCTCTTTTCTCGTA 510

GW14H9 GATTCGTTTCCCACTCGTCCAACGAGTACACCATAGAGGATCACGGGCTCTTTTCTCGTA 511

GW3D10 GATTCGTTTCCCACTCGTCCAACGAGTACACCATAGAGGATCACGGGCTCTTTTCTCGTA 513

S17E4 GATTCGTTTCCCACTCGTCCAACGAGTACACCATAGAGGATCACGGGCTCTTTTCTCGTA 515

L4C16 GATTCGTTTCCCACTCGTCCAACGAGTACACCATAGAGGATCACGGGCTCTTTTCTCGTA 528

GW7A2 GATTCGTTTCCCACTCGTCCAACGAGTACACCATAGAGGATCACGGGCTCTTTTCTCGTA 540

GW11G11 GATTCGTTTCCCACTCGTCCAACGAGTACACCATAGAGGATCACGGGCTCTTTTCTCGTA 528

G26A7 CAGAAAGCCAAAAACATACTTCTTG**TAA**ACAGAATCACCATCAACAGAAAGTTT**A**CTCCT 570

GW14H9 CAGAAAGCCAAAAACATACTTCTTG**TAA**ACAGAATCACCATCAACAGAAAGTTTGCTCCT 571

GW3D10 CAGAAAGCCAAAAACATACTTCTTG**TAA**ACAGAATCACCATCAACAGAAAGTTTGCTCCT 573

S17E4 CAGAAAGCCAAAAACATACTTCTTG**TAA**ACAGAATCACCATCAACAGAAAG**C**TTGCTCCT 575

L4C16 CAGAAAGCCAAAAACATACTTCTTG**TAA**ACAGAATCACCATCAACAGAAAGTTTGCTCCT 588

GW7A2 CAGAAAGCCAAAAACATACTTCTTG**TAA**ACAGAATCACCATCAACAGAAAGTTTGCTCCT 600

GW11G11 CAGAAAGCCAAAAACATACTTCTTG**TAA**ACAGAATCACCATCAACAGAAAGTTTGCTCCT 588

G26A7 GTTATT**T**GTCGCTGCAGATCATCGCAAGTGATACCCAACAAATTGATTTGAGATCGAAAA 630

GW14H9 GTTATTCGTCGCTGCAGATCATCGCAAGTGATACCCAACAAATTGATTTGAGATCGAAAA 631

GW3D10 GTTATTCGTCGCTGCAGATCATCGCAAGTGATACCCAACAAATTGATTTGAGATCGAAAA 633

S17E4 GTTATTCGTCGCTGCAGATCATCGCAAGTGATACCCAACAAATTGATTTGAGATCGAAAA 635

L4C16 GTTATTCGTCGCTGCAGATCATCGCAAGTGATACCCAACAAATTGATTTGAGATCGAAAA 648

GW7A2 GTTATTCGTCGCTGCAGATCATCGCAAGTGATACCCAACAAATTGATTTGAGATCGAAAA 660

GW11G11 GTTATTCGTCGCTGCAGATCATCGCAAGTGATACCCAACAAATTGATTTGAGATCGAAAA 648

G26A7 TAGTTTTTCCTTTCAATTGTCTTTTGATTTTATTCGTAATTTAAAAATGCGAAT**G**AATTT 690

GW14H9 TAGTTTTTCCTTTCAATTGTCTTTTGATTTTATTCGTAATTTAAAAATGCGAATAAATTT 691

GW3D10 TAGTTTTTCCTTTCAATTGTCTTTTGATTTTATTCGTAATTTAAAAATGCGAATAAATTT 693

S17E4 TAGTTTTTCCTTTCAATTGTCTTTTGATTTTATTCGTAATTTAAAAATGCGAATAAATTT 695

L4C16 TAGTTTTTCCTTTCAATTGTCTTTTGATTTTATTCGTAATTTAAAAATGCGAATAAATTT 708

GW7A2 TAGTTTTTCCTTTCAATTGTCTTTTGATTTTATTCGTAATTTAAAAATGCGAATAAATTT 720

GW11G11 TAGTTTTTCCTTTCAATTGTCTTTTGATTTTATTCGTAATTTAAAAATGCGAATAAATTT 708

G26A7 CGGATTCAATGATAAATAAATATTACACACACAC 724

GW14H9 CGGATTCAATGATAAATAAATATTACACACACAC 725

GW3D10 CGGATTC**C**ATGATAAATAAATATTACACACACAC 727

S17E4 CGGATTCAATGATAAATAAATATTACACACACAC 729

L4C16 CGGATTCAATGATAAATAAATATTACACACACAC 742

GW7A2 CGGATTCAATGATAAATAAATATT---------- 744

GW11G11 CGGATTCAATGATAAATAAATA------------ 730

**D**

L10E8 CTTTTCGTTCTGCTTTATTATAGTTGACGGATAATATACCTGCAAA**ATG**ACAAATTCAAA 60

S7A3 ------------------------------------------------------------

L14H11 CTTTTCGTTCTGCTTTATTATAGTTGACGGATAATATACCTGCAAA**ATGG**CAAATTCAAA 60

L10E8 GGGTTATCGTCGTGGTACACGAGACATGTTCTCGCGTCCATTCAAAAAGCATGGAGTTAT 120

S7A3 ------------------------------------------------------------

L14H11 GGGTTATCGTCGTGGTACACGAGACATGTTCTCGCGTCCATTCAAAAAGCATGGAGTTAT 120

L10E8 TCCACTTTCAACATACATGAAAGTATACAAAATCGGTGATATCGTCGACATCAAGGGTCA 180

S7A3 --------------------------------------------------------GTCA 4

L14H11 TCCACTTTCAACATACATGAAAGTATACAAAATCGGTGATATCGTCGACATCAAGGGTCA 180

L10E8 TGGTGCCGTTCAAAAGGGTATGCCATACAAAGCCTACCACGGTAAAACTGGCCGAGTATA 240

S7A3 TGGTGCCGTTCAAAAGGGTATGCCATACAAAGCCTACCACGGTAAAACTGGCCGAGTATA 64

L14H11 TGGTGCCGTTCAAAAGGGTATGCCATACAAAGCCTACCACGGTAAAACTGGCCGAGTATA 240

L10E8 TAACGTAACTCCACATGCTGTTGGAATAATCGTTAACAAACGTGTTCGCGGTAAAATTCT 300

S7A3 TAACGTAACTCCACATGCTGTTGGAATAATCGTTAACAAACGTGTTCGCGGTAAAATTCT 124

L14H11 TAACGTAACTCCACATGCTGTTGGAATAATCGT**C**AACAAACGTGTTCG**T**GGTAAAATTCT 300

L10E8 AGCAAAACGTATCAACGTTCGTATTGAACACATCAAACACTCAAAATGCCGTGAGGATTT 360

S7A3 AGCAAAACGTATCAACGTTCGTATTGAACACATCAAACACTCAAAATGCCGTGAGGATTT 184

L14H11 AGCAAAACGTATCAACGTTCGTATTGAACACATCAAACACTCAAAATGCCGTGAGGATTT 360

L10E8 CTTGCGTCGTGTTAAGGAAAACGAACGTTTATTGAAAGATGCCAAAGAAAAGGGTAAATG 420

S7A3 CTTGCGTCGTGTTAA**A**GAAAACGAACGTTTATTGAAAGATGCCAAAGAAAAGGGTAAATG 244

L14H11 CTTGCGTCGTGTTAAGGAAAACGAACGTTTATTGAAAGA**A**GCCAAAGAAAAGGGTAAATG 420

L10E8 GGTTTCTTTGAAACGTCAGCCAAAACCACCACGTAACGCTGTTATCGTCAAGAACCCACC 480

S7A3 GGTTTC**G**TTGAAACGTCAGCCAAAACCACCACGTAACGCTGTTATCGTCAAGAACCCACC 304

L14H11 GGTTTCTTTGAAACGTCAGCCAAAACCACCACGTAACGCTGTTATCGTCAAGAACCCACC 480

L10E8 AACACCAATTGCATTGGCACCAATTCCATACGAATTCATTGCT**TAA**ATGAAAGCTGGATA 540

S7A3 AACACCAATTGCATTGGCACCAATTCCATACGAATTCATTGCT**TAA**ATGAAAGCTGGATA 364

L14H11 AACACCAATTGCATTGGCACCAATTCCATACGAATTCATTGCT**TAA**ATGAAAGCTGGATA 540

L10E8 TCGAACATTGATTTTGTACGCTAAATCAAGAACTCCGGAAATATAAGTGAAATATTTCC 599

S7A3 TCGAACATTGATTTTGTACGCTAAATCAAGAACTCCGGAAATATAAGTGAAATAT---- 419

L14H11 TCGAACATTGATTTTGTACGCTAAATCAAGAACTCCGGAAATATAAGTGAAATATTTCC 599

**E**

GW16F4 ------------------------------------------------------------

S7H6 ------------------------------------------------------------

L14F3 TTTTCCATTAAA**ATG**GCACCGAAGAAACCAACTGAGAAGTCTGGCGAAAAGAAAGCAGCT 60

G10A3 TTTTCCATTAAA**ATG**GCACCGAAGAAACCAACTGAGAAGTCTGGCGAAAAGAAAGCAGCT 60

GW16F4 ------------------------------------------------------------

S7H6 ------------------------------------------------------------

L14F3 CCAGCTGCTTCGACCTCGAAGGATACCAAAAAAGCTGCTCCAGCAGCCAAAAAACCAGCC 120

G10A3 CCAGCTGCTTCGACCTCGAAGGATACCAAAAAAGCTGCTCCAGCAGCCAAAAAACCAGCC 120

GW16F4 ----------------------------------------------------------CT 2

S7H6 ------------------------------------------------------------

L14F3 GCTGAAAAGAAACCGGCCGCTGCTAAGCCAGCAGCCGCTAAGCCAGCTACCAAACCGGCT 180

G10A3 GCTGAAAAGAAACCGGCCGCTGCTAAGCCAGCAGCCGCTAAGCCAGCTACCAAACCGGC**A** 180

GW16F4 GCATCAAAAGCTCCAGCTAAAGCCGCTGAAAAAAAACCAGCAACAAAACGCCCAGCCGGT 62

S7H6 ------------------------------------------------------------

L14F3 GCATCAAAAGCTCCAGCTAAAGCCGCTGAAAAAAAACCAGCAACAAAACGCCCAGCCGGT 240

G10A3 GCATCAAAAGCTCCAGCTAAAGCCGCTGA**T**AAAAAACCAGCAACAAAACGCCC**G**GCCGGT 240

GW16F4 GATGCCAAAGCACCAGCATCGAAAAAACCAGCCACCGCTAAACCAGCCGCTAAAAATGCT 122

S7H6 --TGCCAAAGCACCAGCATCGAAAAAACCAGCCACCGCTAAACCAGCCGCTAAAAATGCT 58

L14F3 GATGCCAAAGCACCAGCATCGAAAAAACCAGCCACCGCTAAACCAGCCGCTAAAAATGCT 300

G10A3 GATGCCAAAGCACCAGCATCGAAAAAACCAGCCACCGCTAAACCAGCCGCTAAAAATGCT 300

GW16F4 GCTACCAAAAAGCCAGCAACCAAAAAACCAGCCGCTGCCAAGCCAAAAACTAAAGTTGCT 182

S7H6 GCTACCAAAAAGCCAGCAACCAAAAAACCAGCCGCTGCCAAGCCAAAAACTAAAGTTGCT 118

L14F3 GCTACCAAAAAGCCAGCAACCAAAAAACCAGCCGCTGCCAAGCCAAAAACTAAAGTTGCT 360

G10A3 GCTACCAAAAAGCCAGCAACCAAAAAACCAGCCGCTGCCAAGCCAAAAACTAAAGTTGCT 360

GW16F4 GTTAAGAAACCAATCGCTAAGAAAACAACCACCAAAGCTGGCGCTAAATTGGCTGCTAAA 242

S7H6 GTTAA**A**AAACCAATCGCTAAGAAAACAAC**A**ACCAAAGCTGGCGCTAAATTGGC**C**GCTAAA 178

L14F3 GTTAAGAAACCAATCGCTAAGAAAACAACCACCAAAGCTGGCGCTAAATTGGCTGCTAAA 420

G10A3 GTTAAGAAACCAATCGCTAAGAAAACAACCACCAAAGCTGGCGCTAAATTGGCTGCTAAA 420

GW16F4 GTGAAAACATTGAAAAAACCATCAGCCAAAGGTGCCGTTTTGTCTAAAAATAAGAAGGCT 302

S7H6 GTGAAAACATTGAAAAAACCATCAGCCAAAGGTGCCGTTTTGTCTAAAAATAAGAAGGCT 238

L14F3 GTGAAAACATTGAAAAAACCATCAGCCAAAGGTGCCGTTTTGTCTAAAAATAAGAAGGCT 480

G10A3 GTGAAAACATTGAAAAAACCATCAGCCAAAGGTGCCGTTTTGTCTAAAAATAAGAAGGCT 480

GW16F4 GCCGCTGCTGTTGTCCGTGCTAAAAAAGTACAAAAGAAGGTCGTCAAAGGTCCATTTGGC 362

S7H6 GCCGCTGCTGTTGTCCGTGCTAAAAAAGTACAAAAGAAGGTCGTCAAAGGTCCATTTGGC 298

L14F3 GCCGCTGCTGTTGTCCGTGCTAAAAAAGTACAAAAGAAGGTCGTCAAAGGTCCATTTGGC 540

G10A3 GC**T**GCTGCTGTTGTCCGTGCTAAAAAAGTACAAAAGAAGGTCGTCAAAGGTCCATTTGGC 540

GW16F4 ACACGTACACGCAAGATCCGTACCAGTGTTACATTCCGTCGCCCAAAAACATTGGCATTG 422

S7H6 ACACGTACACGCAA**A**ATCCGTACCAGTGTTACATTCCGTCGCCCAAAAACATTGGCATTG 358

L14F3 ACACGTACACGCAAGATCCGTACCAGTGTTACATTCCGTCGCCCAAAAACATTGGCATTG 600

G10A3 ACACGTACACGCAA**A**ATCCGTACCAGTGTTACATTCCGTCGCCCAAAAACATTGGCATTG 600

GW16F4 CCACGGAATCCAAAATATCCACGTAAATCGGTTCCAACCAGAGATCGCATGGATCCATTC 482

S7H6 CCACGGAATCCAAAATATCCACGTAAATCGGTTCCAACCAGAGATCGCATGGATCCATTC 418

L14F3 CCACGGAATCCAAAATATCCACGTAAATCGGTTCCAACCAGAGATCGCATGGATCCATTC 660

G10A3 CCACGGAATCCAAAATATCCACGTAAATCGGTTCCAACCAGAGATCGCATGGATCCATTC 660

GW16F4 AACATTGTCAAATA**T**CCATTGACAACTGAAGCCGCCATGAAGAAGATCGAAGACAATAAT 542

S7H6 AACATTGTCAAATACCCATT**T**ACAACTGAAGCCGCCATGAAGAAGATCGAAGACAATAAT 478

L14F3 AACATTGTCAAATACCCATTGACAACTGAAGCCGCCATGAAGAAGATCGAAGACAATAAT 720

G10A3 AACATTGTCAAATACCCATTGACAACTGAAGCCGCCATGAAGAAGATCGAAGACAATAAT 720

GW16F4 ACTTTGGTATTCTTGACACATTTGCGTGCAAACAAACACCATGTACGAGCTGCTGTTCGT 602

S7H6 ACTTTGGTATTCTTGACACATTTGCGTGCAAACAAACACCATGTACGAGCTGCTGTTCGT 538

L14F3 ACTTTGGTATTCTTGACACATTTGCGTGCAAACAAACACCATGTACGAGCTGCTGTTCGT 780

G10A3 ACTTTGGTATTCTTGACACATTTGCGTGCAAACAAACACCATGTACGAGCTGCTGTTCGT 780

GW16F4 AAATTATACGATATTAAAGTAGCTAAGGTTAACTTGTTGATTAGACCCGATGGACAAAAG 662

S7H6 AAATTATACGATATTAAAGTAGCTAA**A**GTTAACTTGTTGATTAGACCCGATGGACAAAAG 598

L14F3 AAATTATACGATATTAAAGTAGCTAAGGTTAACTTGTTGATTAGACCCGATGGACAAAAG 840

G10A3 AAATTATACGATATTAAAGTAGCTAAGGTTAACTTGTTGATTAGACCCGATGGACAAAAG 840

GW16F4 AAAGCCTACGTTCGTCTGGCACGAGACTACGATGCTTTAGATATTGCAAACAAAATCGGC 722

S7H6 AAAGCCTACGTTCGTCTGGCACGAGACTACGATGCTTTAGATATTGCAAACAAAATCGGC 658

L14F3 AAAGCCTACGTTCGTCTGGCACGAGACTACGATGCTTTAGATATTGCAAACAAAATCGGC 900

G10A3 AAAGCCTACGTTCGTCTGGCACGAGACTACGATGCTTTA**A**ATATTGCAAACAAAATCGGC 900

GW16F4 ATCATA**TAA**ATCATTCATTTCAGAAATGTACACAGATTTTCTACATTGTTTTTTTTTCTC 782

S7H6 ATCATA**TAA**ATCATTCATTTCAGAAATGTACACAGATTTTCTACATTGTTTTTTTTTCTC 718

L14F3 ATCATA**TAA**ATCATTCATTTCAGAAATGTACACAGATTTTCTACATTGTTTTTTTTTCTC 960

G10A3 AT---------------------------------------------------------- 902

GW16F4 GATTTTGCCCAAAATGGATGCAGAAGAAATGTAACATGTTCTC**A**CAATCTAATTACGAAT 842

S7H6 GATTTTGCCCAAAATGGATGCAGAATAAATGTAACATGTTCTC-CAATCTAATTACGAAT 777

L14F3 GATTTTGCCCAAAATGGATGCAGAAGAAATGTAACATGTTCTC**A**CAATCTAATTACGAAT 1020

G10A3 ------------------------------------------------------------

GW16F4 AAATCTG 849

S7H6 AAATCTG 784

L14F3 AAATCTG 1027

G10A3 -------

**F**

L12H5 ----TTTCACGTGTTGTTTTCATACATTACGGTTGTCGATATTAATTCAAA**ATG**AAGTTC 56

GW13D1 --------------------CATACATTACGGTTGTCGATATTAATTCAAA**ATG**AAGTTC 40

L21C5 TT**G**TTTTCACGTGTTGTTTTCATACATTACGGTTGTCGATATTAATTCAAA**ATG**AAGTTC 60

L2E1 TTTTTTTCACGTGTTGTTTTCATACATTACGGTTGTCGATATTAATTCAAA**ATG**AAGTTC 60

L1B8 --T**C**TTTCACGTGTTGTTTTCATACATTACGGTTGTCGATATTAATTCAAA**ATG**AAGTTC 58

L12H5 AACCAACACGTCACATCAGATCGTAGCAAGAACCGCAAACGGCATTTCACTGCCCCATCA 116

GW13D1 AACCAACACGTCACATCAGATCGTAGCAAGAACCGCAAACGGCATTTCACTGCCCCATCA 100

L21C5 AACCAACACGTCACATCAGATCGTAGCAAGAACCGCAAACGGCATTTCACTGCCCCATCA 120

L2E1 AACCAACACGTCACATCAGATCGTAGCAAGAACCGCAAA**T**GGCATTTCACTGCCCCATCA 120

L1B8 AACCAACACGTCACATCAGATCGTAGCAAGAACCGCAAACGGCATTTCACTGCCCCATCA 118

L12H5 CACATTCGCCGAAAGCTGATGTCAGCTCCATTGTCCAAAGAACTGAGACAAAAATACAAT 176

GW13D1 CACATTCGCCGAAAGCTGATGTCAGCTCCATTGTCCAAAGAACTGAGACAAAAATACAAT 160

L21C5 CACATTCGCCGAAAGCTGATGTCAGCTCCATTGTCCAAAGAACTGAGACAAAAATACAAT 180

L2E1 CACATTCGCCGAAAGCTGATGTCAGCTCCATTGTCCAAAGAACTGAGACAAAAATACAAT 180

L1B8 CACATTCGCCGAAAGCTGATGTCAGCTCCATTGTCCAAAGAA**T**TGAGACAAAAATACAAT 178

L12H5 GTTCGCCGGATGCCAATCCGTAAGGACGATGAAGTTCAAGTGGTCCGTGGTCACTACAAG 236

GW13D1 GTTCGCCGGATGCCAATCCGTAAGGACGATGAAGTTCAAGTGGTCCGTGGTCACTACAAG 220

L21C5 GTTCGCCGGATGCCAATCCGTAAGGA**T**GATGAAGTTCAAGTGGTCCGTGGTCACTACAAG 240

L2E1 GTTCGCCGGATGCCAATCCGTAAGGA**T**GATGAAGTTCAAGTGGTCCGTGGTCACTACAAG 240

L1B8 GTTCGCCGGATGCCAATCCGTAAGGACGATGAAGTTCAAGTGGTCCGTGGTCACTACAAG 238

L12H5 AGCAACACCGTTGGCAAAGTTATCCAAGTCTACAG**G**AAAAAATTTGTAGTTTACATCGAA 296

GW13D1 AGCAACACCGTTGGCAAAGTTATCCAAGTCTACAGAAAAAAATTTGTAGTTTACATCGAA 280

L21C5 AGCAACACCGTTGGCAAAGTTATCCAAGTCTACAGAAAAAAATTTGTAGTTTACATCGAA 300

L2E1 AGCAACACCGTTGGCAAAGTTATCCAAGTCTACAGAAAAAAATTTGTAGTTTACATCGAA 300

L1B8 AGCAACACCGTTGGCAAAGTTATCCAAGTCTACAGAAAAAAATTTGTAGTTTACATCGAA 298

L12H5 CGTATCCAACGTGAAAAAACTAACGGTACAAACGCTCCAGTCGGTATTCACCCATCAAAA 356

GW13D1 CGTATCCAACGTGAAAAAACTAACGGTACAAACGCTCCAGTCGGTATTCACCCATCAAAA 340

L21C5 CGTATCCAACGTGAAAAAACTAACGGTACAAACGCTCCAGTCGGTATTCACCCATCAAAA 360

L2E1 CGTATCCAACGTGAAAAAACTAACGGTAC**T**AA**T**GCTCCAGTCGGTATTCACCCATCAAAA 360

L1B8 CGTATCCAACGTG**G**AAAAACTAACGGTACAAA**T**GCTCCAGTCGGTATTCACCCATCAAAA 357

L12H5 TGTGTTATCGTTAAATTGAAGTTGGACAAAGATCGTAAAGCCATTCTGGAACGTCGTGCT 416

GW13D1 TGTGTTATCGTTAAATTGAAGTTGGACAAAGATCGTAAAGCCATTCTGGAACGTCGTGCT 400

L21C5 TGTGTTATCGTTAAATTGAAGTTGGACAAAGATCGTAAAGCCATTCTGGAACGTCGTGCT 420

L2E1 TGTGTTATCGTTAAATTGAAGTTGGACAAAGATCGTAA**G**GCCATTCTGGAACGTCGTGCT 420

L1B8 TGTGTTATCGTTAAATTGAAGTTGGACAAAGATCGTAAAGCCATTCTGGAACGTCGTGCT 417

L12H5 AAAGGACGTTTGGCTGCTCTTGGCAAAGACAAGGGTAAATACACTGAAGAAACAACAACC 476

GW13D1 AAAGGACGTTTGGCTGCTCTTGGCAAAGACAAGGGTAAATACACTGAAGAAACAACAACC 460

L21C5 AAAGGACGTTTGGCTGCTCTTGGCAAAGACAAGGGTAAATACACTGAAGAAACAACAACC 480

L2E1 AAAGGACGTTTGGCTGCTCTTGGCAAAGACAAGGGTAAATACACTGAAGAAACAACAACC 480

L1B8 AAAGGACGTTTGGCTGCTCTTGGCAAAGA**T**AAGGGTAAA**C**ACACTGAAGAAACAACAACC 477

L12H5 GCCACTCCAATGGAAACCGCT**TAA**ACGATTCAATCCCGAAA-AAAATTGCAGTGCGGAAT 535

GW13D1 GCCACTCCAATGGAAACCGCT**TAA**ACGATTCAATCCCGAAA-AAAATTGCAGTGCGGAAT 519

L21C5 GCCACTCCAATGGAAACCGCT**TAA**ACGATTCAATCCCGAAA**G**AAAATTGCAGTGCGGAAT 540

L2E1 GCCACTCCAATGGAAACCGCT**TAA**ACGATTCAATCCCGAAA**G**AAAATTGCAGTGCGGAAT 540

L1B8 GCCACTCCAATGGAAACCGCT**TAA**ACGATTCAATCCCGAAA-AAAATTGCAGTG**T**GGAAT 536

L12H5 TTTTTTTTTCGTACGTCATTCAATCGAATCGATTCAAATGTAATTAAAAAAGCAACGAAG 595

GW13D1 TTTTTTTTTCGTACGTCATTCAATCGAATCGATTCAAATGTAATTAAAAAAGCAACGAAG 579

L21C5 TTTTTTTTTCGTACGTCATTCAATCGAATCGATTCAAATGTAATTAAAAAAGCAACGAAG 600

L2E1 TTTTTTTTTCGTA**GA**TCATTCAATCGAATCGATTCAAATGTAATTAAAAAAGCAACGAAG 600

L1B8 TTTTTTTTTCGT**G**CGTCATTCAATCGAATCGATTCAAATGTAATTAAAAAAGCAACGAAG 596

L12H5 AATTCGTTCTTCATTCCATAAAAAACC- 622

GW13D1 AATTCGTTCTTCATTCCATAAAAAAC**A**G 607

L21C5 AATTCGTTCTTCATTCCATAAAAAACC- 627

L2E1 AATTCGTTCTTCATTCCAT--------- 619

L1B8 AATTCGTTCTTCATTCC----------- 613

**G**

GW14H4 ------------------------------------------------------------

G38D10 ------------------------------------TTCAAGCAGTGAGGATAATTTGAA 24

G7G3 ---------------------CTTCTTCTTTCAGACTTCAAGCAGTGAGGATAATTTGAA 39

G20G12 TGGAAAACACATGTCAAAGTTCTTCTTCTTTCAGACTTCAAGCAGTGAGGATAATTTGAA 60

GW14H4 AGCCGTTCAAA**ATG**GTTGCCCCAAAAAAACAAAAAAAAGCTTTAGAAAGCACCAATGCCC 60

G38D10 AGCCGTTCAAA**ATG**GTTGCCCCAAAAAAACAAAAAAAAGCTTTAGAAAGCACCAATGCCC 84

G7G3 AGCCGTTCAAA**ATG**GTTGC**T**CCAAAAAAACAAAAAAAAGCTTTAGAAAGCACCAATGCCC 99

G20G12 AGCCGTTCAAA**ATG**GTTGC**T**CCAAAAAAACAAAAAAAAGCTTTAGAAAGCACCAATGCCC 120

GW14H4 GTTTGGCTTTGGTTATGAAATCCGGCAAATATTGTTTGGGCTACAAACAAACATTGAAAT 120

G38D10 GTTTGGCTTTGGTTATGAAATCCGGCAAATATTGTTTGGGCTACAAACAAACATTGAAAT 144

G7G3 GTTTGGCTTTGGTTATGAAATCCGGCAAATATTGTTTGGGCTACAAACAAACATTGAAAT 159

G20G12 GTTTGGCTTTGGTTATGAAATCCGGCAAATATTGTTTGGGCTACAAACAAACATTGAAAT 180

GW14H4 CATTGCGTCAAGGCAAAGCTAAATTGGTCATCATTGGCAACAATACACCACCGCTAAGAA 180

G38D10 CATTGCGTCAAGGCAAAGCTAAATTGGTCATCATTGGCAACAATACACC**C**CCGCTAAGAA 204

G7G3 CATTGCGTCAAGGCAAAGCTAAATTGGTCATCATTGGCAACAATACACCACCGCTAAGAA 219

G20G12 CATTGCGTCAAGGCAAAGCTAAATTGGTCATCATTGGCAACAATACACCACCGCTAAGAA 240

GW14H4 AATCCGAAATCGAATACTACGCCATGTTGGCCAAAACTGGTGTTCACCATTACAGCGGCA 240

G38D10 AATCCGAAATCGAATACTACGCCATGTTGGCCAAAACTGGTGTTCACCATTACAGCGGCA 264

G7G3 AATCCGAAATCGAATACTACGCCATGTTGGCCAAAACTGGTGTTCA**T**CA**C**TACAGCGGCA 279

G20G12 AATCCGAAATCGAATACTACGCCATGTTGGCCAAAACTGGTGTTCA**T**CA**C**TACAGCGGCA 300

GW14H4 ACAACATCGAATTAGGTACCGCTTGCGGTAAATACTTCCGTGTTTGCACCATGTCCATCA 300

G38D10 ACAACATCGAATTAGG**G**ACCGCTTGCGGTAAATACTTCCGTGTTTGC**C**CCATGTCCATCA 324

G7G3 ACAACATCGAATTAGGTACCGCTTGCGGTAAATACTTCCGTGTTTGCACCATGTCCATCA 339

G20G12 ACAACATCGAATTAGGTACCGCTTGCGGTAAATACTTCCGTGTTTGCACCATGTCCATCA 360

GW14H4 CAGATCCAGGTGATTCGGACATCATCCGAACAATGCCAGAAGCTCAAGTTCAA**TAA**TAAA 360

G38D10 CAGATCCAGGTGATTCGGACATCATCCGAACAATGCCAGAAGCTCAAGTTCAA**TAA**TAAA 384

G7G3 CAGATCCAGGTGA**C**TCGGACATCATCCGAACAATGCCAGAAGCTCAAGTTCAA**TAAA**A**TT** 399

G20G12 CAGATCCAGGTGA**C**TCGGACATCATCCGAACAATGCCAGAAGCTCAAGTTCAA**TAAA**A**TT** 420

GW14H4 AAAACGTTGAAGCATTGCGTTTTTGAAATTTTTAAATAAAGTACAAAATTTTTGTTTGAA 420

G38D10 AAAACGTTGAAGCATTGCGTTTTTGAAATTTTTAAATAAAGTACAAAATTTTTGTTTGAA 444

G7G3 **TTGCAT**TT**TG**A**TG**A**A**TG**TACGC**T**C**GAAA----**A**AAA**AC**AA**A**TA**A**AA**GCAAA**T**GA**TT**CA**A**T** 455

G20G12 **TTGCAT**TT**TG**A**TG**A**A**TG**TACGC**T**C**GAAA----**A**AAA**AC**AA**A**TA**A**AA**GCAAA**T**GA**TT**CA**A**T** 476

GW14H4 AACAAATAAATGCAAATATCGATTCAAT-------------------------------- 448

G38D10 AACAAATAAATGCAAATATCGATTCAATAAATTATTTTCTACTAATTTCGTCGACTTTCA 504

G7G3 A**TATG**A**ATC**AT**C**C**GC**A**GCC**CG**T**-------------------------------------- 477

G20G12 A**TATG**A**ATC**AT**C**C**GC**A**G**A**C**C**AT**-------------------------------------- 498

GW14H4 --------

G38D10 TCTGTCGA 512

G7G3 --------

G20G12 --------

**H**

GW14H8 --------AGAAATTGTGTGAAAA**ATG**GTTCAACGATTGACATTAAGACGACGATTGTCG 52

GW2E10 -------CAGAAATTGTGTGAAAA**ATG**GTTCAACGATTGACATTAAGACGACGATTGTCG 53

GW3G7 GTTCATACAGAAATTGTGTGAAAA**ATG**GTTCAACGATTGACATTAAGACGACGATTGTCG 60

G14E9 GTTCATACAGAAATTGTGTGAAAA**ATG**GTTCAACGATTGACATTAAGACGACGATTGTCG 60

GW14H8 TACAACACAAACTCAAACAAACGGCGCATTGTTCGTACACCGGGCGGCCGTTTGGTATAT 112

GW2E10 TACAACACAAACTCAAACAAACGGCGCATTGTTCGTACACCGGGCGGCCGTTTGGTATAT 113

GW3G7 TACAACACAAACTCAAACAAACGGCGCATTGTTCGTACACCGGGCGGCCGTTTGGTATAT 120

G14E9 TACAACACAAACTCAAACAAACGGCGCATTGTTCGTACACCGGGCGGCCGTTTGGTATAT 120

GW14H8 CAATACGTGAAAAAGCGCAAGAATGTGCCAAAATGCGGTCAATGCAAGGAAAAATTGAAG 172

GW2E10 CAATACGTGAAAAAGCGCAAGAATGT**C**CCAAAATGCGGTCAATGCAAGGAAAAATTGAAG 173

GW3G7 CAATACGTGAAAAAGCGCAAGAATGTGCCAAAATGCGGTCAATGCAAGGAAAAATTGAAG 180

G14E9 CAATACGTGAAAAAGCGCAAGAA**C**GTGCCAAAATGCGGTCAATGCAAGGAAAAATTGAAG 180

GW14H8 GGAATCCGGCCAACACGTCCATTAGAACGTTCACGCATTTCAAAACGTCAAAAAACTGTA 232

GW2E10 GGAATCCGGCCAACACGTCCATTAGAACGTTCACGCATTTCAAAACGTCAAAAAACTGTA 233

GW3G7 GGAATCCGGCCAACACGTCCATTAGAACGTTCACGCATTTCAAAACGTCAAAAAACTGTA 240

G14E9 GGAATCCGGCCAACACGTCCATTAGAACGTTCACGCATTTCAAAACGTCAAAAAACTGTG 240

GW14H8 GCCCGTACATATGGTGGTGTTTTGTGCCATCAATGCGTTCGTGAACGTATCGTTCGTGCT 292

GW2E10 GCCCGTACATATGGTGGTGTTTTGTGCCATCAATGCGTTCGTGAACGTATCGTTCGTGCT 293

GW3G7 GCCCGTACATATGGTGGTGTTTTGTGCCATCAATGCGTTCGTGAACGTATCGTTCGTGCT 300

G14E9 GCCCGTACATATGGTGGTGTTTTGTGCCATCAATGCGTTCGTGAACGTATCGTTCGTGCT 300

GW14H8 TTCCTCATCGAGGAACAAAAGATCGTTAAGGTTCTCAGTCGATCACCAAAACTTGCGGTC 352

GW2E10 TTCCTCATCGAGGAACAAAAGATCGTTAA**A**GTTCTCAGTCGATCACCAAAACTTGCGGTC 353

GW3G7 TTCCTCATCGAGGAACAAAAGATCGTTAAGGTTCTCAGTCGATCACCAAAACTTGCGGTC 360

G14E9 TTCCTCATCGAGGAACAAAAGATCGTTAAGGTTCTCAGTCGATCACCAAAACTTGCGGTC 360

GW14H8 AAA**TAA**ATTTCATGCATTTTATTTAAATCAAATGAA---AAAAAAAAACAAATCCATGAA 409

GW2E10 AAA**TAA**ATTTCATGCATTTTATTTAAATCAAATGAA**AGA**AAAAAAAAACAAATCCA**A**GAA 413

GW3G7 AAA**TAA**ATTTCATGCATTTTATTTAAATCAAATGAA---AAAAAAAAACAAATCCATGAA 417

G14E9 AAA**TAA**ATTTCATGCATTTTATTTAAATC------------------------------- 393

GW14H8 GAAAAATCATAAAACAATGTTATCGTTTTAAATGATAAAGATTGACATTAATAAAATGTT 469

GW2E10 GAAAAATCATAAAACAATGTTATCGTTTTAAATGATAAAGATTGACATTAATAAAATGTT 473

GW3G7 GAAAAATCATAAAACAATGTTATCGTTTTAAATGATAAAGATTGACATTAATAAAATGTT 477

G14E9 ------------------------------------------------------------

GW14H8 AATTCAATCCT----- 480

GW2E10 AATTCAATCCTCGTGC 489

GW3G7 AATTCAATCCT----- 488

G14E9 ----------------

**I**

GW9D9 -----------CAAAATCAACCAAG**ATG**GGAAAAGTTAAGTGCTCTGAATTGCGTACAAA 49

S14D10 ------------------------------------------------------------

S10E8 --------TCTCAAAATCAACCAAG**ATG**GGAAAAGTTAAGTGCTCTGAATTGCGTACAAA 52

S14F11 ------GTTCTCAAAATCAACCAAG**ATG**GGAAAAGTTAAGTGCTCTGAATTGCGTACAAA 54

S14G5 ----ACGTTCTCAAAATCAACCAAG**ATG**GGAAAAGTTAAGTGCTCTGAATTGCGTACAAA 56

L16E7 TTTTACGTTCTCAAAATCAACCAAG**ATG**GGAAAAGTTAAGTGCTCTGAATTGCGTACAAA 59

G36G4 --TTACGTTCTCAAAATCAACCAAG**ATG**GGAAAAGTTAAGTGCTCTGAATTGCGTACAAA 59

GW9D9 GGACAAAAAAGAGTTGACCAAGCAACTCGATGAACTCAAAACGGAGCTCCTTGGATTGCG 109

S14D10 ---------------GACCAAGCAACTCGATGAACTCAAAACGGAGCTCCTTGGATTGCG 45

S10E8 GGACAAAAAAGAGTTGACCAAGCAACTCGATGAACTCAAAACGGAGCTCCTTGGATTGCG 112

S14F11 GGACAAAAAAGAGTTGACCAAGCAACTCGATGAACTCAAAACGGAGCTCCTTGGATTGCG 114

S14G5 GGACAAAAAAGAGTTGACCAAGCAACTCGATGAACTCAAAACGGAGCTCCTTGGATTGCG 116

L16E7 GGACAAAAAAGAGTTGACCAAGCAACTCGATGAACTCAAAACGGAGCTCCTTGGATTGCG 119

G36G4 GGACAAAAAAGAGTTGACCAAGCAACTCGATGAACTCAAAACGGAGCTCCTTGGATTGCG 119

GW9D9 TGTCGCCAAAGTAACCGGTGGTGCTGCATCAAAATTGTCAAAAATCCGTGTGGTCCGCAA 169

S14D10 TGTCGCCAAAGTAACCGGTGGTGCTGCATCAAAATTGTCAAAAATCCGTGT**T**GTCCGCAA 105

S10E8 TGTCGCCAAAGTAACCGGTGGTGCTGCATCAAAATTGTCAAAAATCCGTGTGGTCCGCAA 172

S14F11 TGTCGCCAAAGTAACCGGTGGTGCTGCATCAAAATTGTCAAAAATCCGTGTGGTCCGCAA 174

S14G5 TGTCGCCAAAGTAACCGGTGGTGCTGCATCAAAATTGTCAAAAATCCGTGT**T**GTCCGCAA 176

L16E7 TGTCGCCAAAGTAACCGGTGGTGCTGCATCAAAATTGTCAAAAATCCGTGTGGTCCGCAA 179

G36G4 **G**GTCGCCAAAGTAACCGGTGGTGCTGCATCAAAATTGTCAAAAATCCGTGTGGTCCGCAA 179

GW9D9 GGCTATTGCCCGTGTTAACATTGTGATGCACCAAAAAACCAAGGAAAATTTGCGCAAATT 229

S14D10 GGCTATTGCCCGTGTTAACATTGTGATGCACCAAAAAACCAAGGAAAATTTGCGCAAATT 165

S10E8 GGCTATTGCCCGTGTTAACATTGTGATGCACCAAAAAACCAAGGAAAATTTGCGCAAATT 232

S14F11 GGCTATTGCCCGTGTTAACATTGTGATGCACCAAAAAACCAAGGAAAATTTGCGCAAATT 234

S14G5 GGCTATTGCCCGTGTTAACATTGTGATGCACCAAAAAACCAAGGAAAATTTGCGCAAATT 236

L16E7 GGCTATTGCCCGTGTTAACATTGTGATGCACCAAAAAACCAAGGAAAATTTGCGCAAATT 239

G36G4 GG**T**TATTGCCCGTGTTAACATTGTGATGCACCAAAAAACCAAGGAAAATTTGCGCAAATT 239

GW9D9 CTACAGAGACAAACGTTTGAAGCCATTGGACTTGAGGCCAAAGAAAACCCGTGCTATCCG 289

S14D10 CTACAGAGACAAACGTTTGAAGCCATTGGACTTGAGGCCAAAGAAAACCCGTGCTATCCG 225

S10E8 CTACAGAGACAAACGTTTGAAGCCATTGGACTTGAGGCCAAAGAAAACCCGTGCTATCCG 292

S14F11 CTACAGAGACAAACGTTTGAAGCCATTGGACTTGAGGCCAAAGAAAACCCGTGCTATCCG 294

S14G5 CTACAGAGACAAACGTTTGAAGCCATTGGACTTGAGGCCAAAGAAAACCCGTGCTATCCG 296

L16E7 CTACAGAGACAAACGTTTGAAGCCATTGGACTTGAGGCCAAAGAAAACCCGTGCTATCCG 299

G36G4 CTACAGAGACAAACGTTTGAAGCCATTGGACTTGAGGCCAAA**A**AAAACCCGTG------- 292

GW9D9 TAAGGCATTGTCACCACGCGACGCCAACCGCAAAACAGCAAAGGCATTGAAAAAGATGTC 349

S14D10 TAAGGCATTGTCACCACGCGACGCCAACCGCAAAACAGC**C**AAGGCATTGAAAAAGATGTC 285

S10E8 TAAGGCATTGTCACCACGCGACGCCAACCGCAAAACAGCAAAGGCATTGAAAAAGATGTC 352

S14F11 TAAGGCATTGTCACCACGCGACGCCAACCGCAAAACAGCAAAGGCATTGAAAAAGATGTC 354

S14G5 TAAGGCATTGTCACCACGCGACGCCAACCG**T**AAAACAGC**C**AAGGCATTGAAAAAGATGTC 356

L16E7 TAAGGCATTGTCACCACGCGACGCCAACCG**T**AAAACAGC**C**AAGGCATTGAAAAAGATGTC 359

G36G4 ------------------------------------------------------------

GW9D9 AGTGTATCCACAACGCAAATATGCGCTCAAAGCA**TAA**GCAGTGAAAGTTTTTCAGTTTCA 409

S14D10 AGTGTATCCACAACGCAAATATGCGCTCAAAGCA**TAA**GCAGTGAAAGTTTTTCAGTTTCA 345

S10E8 AGTGTATCCACAACGCAAATATGCGCTCAAAGCA**TAA**GCAGTGAAAGTTTTTCAGTTTCA 412

S14F11 AGTGTATCCACAACGCAAATATGCGCTCAAAGCA**TAA**GCAGTGAAAGTTTTTCAGTTTCA 414

S14G5 AGTGTATCCACAACGCAAATATGCGCTCAAAGCA**TAA**GCAGTGAAAGTTTTTCAGTTTCA 416

L16E7 AGTGTATCCACAACGCAAATATGCGCTCAAAGCA**TAA**GCAGTGAAAGTTTTTCAGTTTCA 419

G36G4 ------------------------------------------------------------

GW9D9 TTTCCAACGGTTAGAATGTGTGAATGGCGTTCTATTGGATGGAACGACACCATCATCTAA 469

S14D10 TTTCCAACGGTTAGAATGTGTGAATGGCGTTCTATTGGATGGAACGACACCATCATCTAA 405

S10E8 TTTCCAACGGTTAGAATGTGTGAATGGCGTTCTATTGGATGGAACGACACCATCATCTAA 472

S14F11 TTTCCAACGGTTAGAATGTGTGAATGGCGTTCTATTGGATGGAACGACACCATCATCTAA 474

S14G5 TTTCCAACGGTTAGAATGTGTGAATGGCGTTCTATTGGATGGAACGACACCATCATCTAA 476

L16E7 TTTCCAACGGTTAGAATGTGTGAATGGCGTTCTATTGGATGGAACGACACCATCATCTAA 479

G36G4 ------------------------------------------------------------

GW9D9 TTCAGTTTGTTATTTTACACGAATAAACAAATCGAAATTGATTCAGATCAATT 522

S14D10 TTCAGTTTGTTATTTTACACGAATAAACAAATCGAAATTGATTCAGATCAATT 458

S10E8 TTCAGTTTGTTATTTTACACGAATAAACAAATCGAAATTGATTCAG------- 518

S14F11 TTCAGTTTGTTATTTTACACGAATAAACAAATCGAAATTGATTCAG------- 520

S14G5 TTCAGTTTGTTATTTTAC**G**CGAATAAACAAATCGAAATTGATTC**C**GAT----- 524

L16E7 TTCAGTTTGTTATTTTACACGAATAAACAAATC**A**AAATTGATTCAGATCAATT 532

G36G4 -----------------------------------------------------

**J**

G24C2 --------------------------------TTTTTTTCGCCATTGAAAACGCATTCGT 28

L13F6 GAGCGCTAGTGTTTCGTCTATTTCATATAAACGTTCTTTCGCCATTGAAAACGCATTCGT 60

S10C2 ------------------------------------------CATTGAAAACGCATTCGT 18

G24C2 TTTTAAATACAAAACTTAAT**ATG**GCTGATACACAACCCGCTGTCGCAGCTCCAGCTGCTG 88

L13F6 TTTTAAATACAAAACTTAAT**ATG**GCTGATACACAACCCGCTGTCGCAGCTCCAGCTGCTG 120

S10C2 TTTTAAATACAAAACTTAAT**ATG**GCTGATACACAACCCGCTGTCGCAGCTCCAGCTGCTG 78

G24C2 CCAAATCAAAAGCAGGCAAAACTGAAAAGGCAACACCAGCCGCTCCAG**T**CCCAGTGTCAA 148

L13F6 CCAAATCAAAAGCAGGCAAAACTGAAAAGGCAACACCAGCCGCTCCAGCCCCAGTGTCAA 180

S10C2 CCAAA**C**CAAAAGCAGGCAAAACTGAAAAGGCAACACCAGCCGCTCCAGCCCCAGTGTC**G**A 138

G24C2 CAAGACGGCCACCAGCTCGTGGTCGCTTGTATGCTAAAGCTGTCTTCACCGGCTACAAAC 208

L13F6 CAAGACGGCCACCAGCTCGTGGTCGCTTGTATGCTAAAGCTGTCTTCACCGGCTACAAAC 240

S10C2 CAAGACGGCCACCAGCTCGTGGTCGCTTGTATGCTAAAGCTGTCTTCACCGGCTACAA**G**C 198

G24C2 GTGGACTGAGAAATCAACACGAAGGCCAAGCTATCTTGAAGATCGATGGTTGCCGTAAAA 268

L13F6 GTGGACTGAGAAATCAACACGAAGGCCAAGCTATCTTGAAGATCGATGGTTGCCGTAAAA 300

S10C2 GTGGACTGAGAAATCAACACGAAGGCCAAGCTATCTTGAAGATCGATGGTTGCCGTAAAA 258

G24C2 TGGAACATGGAAAATTCTACGTTGGCAAACGATGTGTTTATGTATTCAAAGCACCAACAC 328

L13F6 TGGAACATGGAAAATTCTACGTTGGCAAACGATGTGTTTATGTATTCAAAGCACCAACAC 360

S10C2 TGGAACATGGAAAATTCTA**T**GTTGGCAAACGATGTGTTTATGTATTCAAAGCACCAACAC 318

G24C2 GCAAAGCATTGCCACAAAAACCATACATTAAATCGCGAGTTCGTGCTATTTGGGG**A**AAAG 388

L13F6 GCAAAGCATTGCCACAAAAACCATACATTAAATCGCGAGTTCGTGCTATTTGGGGCAAAG 420

S10C2 GCAAAGCATTGCCACAAAAACCATACATTAAATCGCGAGTTCGTGCTATTTGGGGCAAAG 378

G24C2 TAACCCGATTGCACGGTAGCACTGGTGCCGTTCGCGCTCGATTCCGTAAAAATTTGCCCG 448

L13F6 TAACCCGATTGCACGGTAGCACTGGTGCCGTTCGCGCTCGATTCCGTAAAAATTTGCCCG 480

S10C2 TAACCCGATTGCACGGTAGCACTGGTGCCGTTCGCGCTCGATTCCGTAAAAATTTGCCCG 438

G24C2 GACACGCAATGGGACAAGCTATCCGCATCATGTTGTACCCTTCAAGAATT**TAA**GTGTGTT 508

L13F6 GACACGCAATGGGACAAGCTATCCGCATCATGTTGTACCCTTCAAGAATT**TAA**GTGTGTT 540

S10C2 GACACGCAATGGGACAAGCTATCCGCATCATGTTGTACCCTTCAAGAATT**TAA**GTGTGTT 498

G24C2 CCGACCAACGTTTGGGGCATTCAATCTTTAATTGAATAAAATCATTTTGTTTTT-AACT- 566

L13F6 CCGACCAACGTTTGGGGCATTCAATCTTTAATTGAATAAAATCATTTTGTTTTT**T**AACT- 599

S10C2 CCGACCAACGTTTGGGGCATTCAATC**CAA**AATTGAATAAAATCATTTTGTTTTT**T**AACT**G** 558

G24C2 ---TTGGAAATGAAACCGGAATGTTGTTTGAAATCCAAAAATAAAATGGCATTCA----- 618

L13F6 ---TTGGAAATGAAACCGGAATGTTGTT**C**GAAATCCAAAAATAAAATGGCATTCATGATT 656

S10C2 **GAA**TTGGAAATGAAACCGGAATGTTGTTTGAAATCCAAAAATAAAATGGCATTCATGA-- 616

G24C2 ------------

L13F6 AAAAAGAAACAG 668

S10C2 ------------

**K**

GW10F6 GCAAAGATATAAGTAGTCGAAG**ATG**GCTGTACGTTACGAATTGGCTGTTGGTCTTAACAA 60

GW19D1 -CAAAGATATAAGTAGTCGAAG**ATG**GCTGTACGTTACGAATTGGCTGTTGGTCTTAACAA 59

GW11D12 GCAAAGATATAAGTAGTCGAAG**ATG**GCTGTACGTTACGAATTGGCTGTTGGTCTTAACAA 60

GW14A11 GCAAAGATATAAGTAGTCGAAG**ATG**GCTGTACGTTACGAATTGGCTGTTGGTCTTAACAA 60

GW1E3 -CAAAGATATAAGTAGTCGAAG**ATG**GCTGTACGTTACGAATTGGCTGTTGGTCTTAACAA 59

GW13B3 ----AGATATAAGTAGTCGAAG**ATG**GCTGTACGTTACGAATTGGCTGTTGGTCTTAACAA 56

G17F5 GCAAAGATATAAGTAGTCGAAG**ATG**GCTGTACGTTACGAATTGGCTGTTGGTCTTAACAA 60

GW11B1 GCAAAGATATAAGTAGTCGAAG**ATG**GCTGTACGTTACGAATTGGCTGTTGGTCTTAACAA 60

GW10F6 AGGCTTCAAAACAACTAAAATCCGTCGTGTTACCTACAAAGGAGACAAAAAAATTAAAGG 120

GW19D1 AGGCTTCAAAACAACTAAAATCCGTCGTGTTACCTACAAAGGAGACAAAAAAATTAAAGG 119

GW11D12 AGGCTTCAAAACAACTAAAATCCGTCGTGTTACCTACAAAGGAGACAAAAAAATTAAAGG 120

GW14A11 AGGCTTCAAAACAACTAAAATCCGTCGTGTTACCTACAAAGGAGACAAAAAAATTAAAGG 120

GW1E3 AGGCTTCAAAACAACTAAAATCCGTCGTGTTACCTACAAAGGAGACAAAAAAAT**C**AAAGG 119

GW13B3 AGGCTTCAAAACAACTAAAATCCGTCGTGTTACCTACAAAGGAGACAAAAAAATTAAAGG 116

G17F5 AGGCTTCAAAACAACTAAAATCCGTCGTGTTACCTACAAAGGAGACAAAAAAATTAAAGG 120

GW11B1 AGGCTTCAAAACAACTAAAATCCGTCGTGTTACCTACAAAGGAGACAAAAAAATTAAAGG 120

GW10F6 TCTTCGTGGAACAAATGTGAAAAACATTCAAACGAAACACACAAAATTCGTTCGTGATTT 180

GW19D1 TCTTCGTGGAACAAATGTGAAAAACATTCAAACGAAACACACAAAATTCGTTCGTGATTT 179

GW11D12 TCTTCGTGGAACAAATGTGAAAAACATTCAAACGAAACACACAAAATTCGTTCGTGATTT 180

GW14A11 TCTTCGTGGAACAAATGTGAAAAACATTCAAACGAAACACACAAAATTCGTTCGTGATTT 180

GW1E3 TCTTCGTGGAACAAATGTGAAAAACATTCAAACGAAACACACAAAATTCGTTCGTGATTT 179

GW13B3 TCTTCGTGGAACAAATGTGAAAAACATTCAAACGAAACACACAAAATTCGTTCGTGATTT 176

G17F5 TCTTCGTGGAACAAATGTGAAAAACAT**C**CAAACGAAACACACAAAATTCGTTCGTGATTT 180

GW11B1 TCTTCGTGGAACAAATGTGAAAAACAT**C**CAAACGAAACACACAAAATTCGTTCGTGATTT 180

GW10F6 GGTACGTGAAGTTGTTGGACACGCTCCATATGAAAAGCGATGCATGGAATTGTTAAAAAT 240

GW19D1 GGTACGTGAAGTTGTTGGACACGCTCCATATGAAAAGCGATGCATGGAATTGTTAAAAAT 239

GW11D12 GGTACGTGAAGTTGTTGGACACGCTCCATATGAAAAGCGATGCATGGAATTGTTAAAAAT 240

GW14A11 GGTACGTGAAGTTGTTGGACACGCTCCATATGAAAAGCGATGCATGGAATTGTTAAAAAT 240

GW1E3 GGTACGTGAAGTTGTTGGACACGCTCCATATGAAAAGCGATGCATGGAATTGTTAAAAAT 239

GW13B3 GGTACGTGAAGTTGTTGGACACGCTCCATATGAAAAGCGATGCATGGAATTGTTAAAAAT 236

G17F5 GGTACGTGAAGTTGTTGGACACGCTCCATATGAAAAGCGATGCATGGAATTGTTAAAAAT 240

GW11B1 GGTACGTGAAGTTGTTGGACACGCTCCATATGAAAAGCGATGCATGGAATTGTTAAAAAT 240

GW10F6 TTCAAAGGATAAACGTGCATTGAAATTCTGTAAAGCTCGTTTGGGCACACACATCCGTGG 300

GW19D1 TTCAAAGGATAAACGTGCATTGAAATTCTGTAAAGCTCGTTTGGGCACACACATCCGTGG 299

GW11D12 TTCAAAGGATAAACGTGCATTGAAATTCTGTAAAGCTCGTTTGGGCACACACATCCGTGG 300

GW14A11 TTCAAAGGATAAACGTGCATTGAAATTCTGTAAAGCTCGTTTGGGCACACACATCCGTGG 300

GW1E3 TTCAAAGGATAAACGTGCATTGAAATTCTGTAAAGCTCGTTTGGGCACACACATCCGTGG 299

GW13B3 TTCAAAGGATAAACGTGCATTGAAATTCTGTAAAGCTCGTTTGGGCACACACATCCGTGG 296

G17F5 TTCAAAGGATAAACGTGCATTGAAATTCTGTAAAGCTCGTTTGGGCACACACATCCGTGG 300

GW11B1 TTCAAAGGATAAACGTGCATTGAAATTCTGTAAAGCTCGTTTGGGCACACACATCCGTGG 300

GW10F6 AAAGCGAAAGCGTGAAGAATTGTCCAACATTTTGACACAAATGCGTAAAGCCGGTCATGC 360

GW19D1 AAAGCGAAAGCGTGAAGAATTGTCCAACATTTTGACACAAATGCGTAAAGCCGGTCATGC 359

GW11D12 AAAGCGAAAGCGTGAAGAATTGTCCAACATTTTGACACAAATGCGTAAAGCCGGTCATGC 360

GW14A11 AAAGCGAAAGCGTGAAGAATTGTCCAACATTTTGACACAAATGCGTAAAGCCGGTCATGC 360

GW1E3 AAAGCGAAAGCGTGAAGAATTGTCCAACATTTTGACACAAATGCGTAAAGCCGGTCATGC 359

GW13B3 AAAGCGAAAGCGTGAAGAATTGTCCAACATTTTGACACAAATGCGTAAAGCCGGTCATGC 356

G17F5 AAAGCGAAAGCGTGAAGAATTGTCCAAC**G**TTTTGACACAAATGCGTAAAGCCGGTCATGC 360

GW11B1 AAAGCGAAAGCGTGAAGAATTGTCCAACATTTTGACACAAATGCGTAAAGCCGGTCATGC 360

GW10F6 TAAG**TAA**ACATAGTTTATGTTTACCGAATGTTTCCATTTGAATGCATTGGACCAGCAATG 420

GW19D1 TAAG**TAA**ACATAGTTTATGTTTACCGAATGTTTCCATTTGAATGCATTGGACCAGCAATG 419

GW11D12 TAAG**TAA**ACATAGTTTATGTTTACCGAATGTTTCCATTTGAATGCATTGGACCAGCAATG 420

GW14A11 TAAG**TAA**ACATAGTTTATGTTTACCGAATGTTTCCATTTGAATGCATTGGACCAGCAATG 420

GW1E3 TAAG**TAA**ACATAGTTTATGTTTACCGAATGTTTCCATTTGAATGCATTGGACCAGCAATG 419

GW13B3 TAAG**TAA**ACATAGTTTATGTTTACCGAATGTTTCCATTTGAATGCATTGGACCAGCAATG 416

G17F5 TAAG**TAA**ACATAGTTTATGTTTACCGAATGTTTCCATTTGAATGCATTGGACCAGCAATG 420

GW11B1 TAAG**TAA**ACATAGTTTATGTTTACCGAATGTTTCCATTTGAATGCATTGGACCAGCAATG 420

GW10F6 TAACATCGTTCATAATGTTTATAACATCTCATATTTTATGAATGTAAAATTAAAACGAAA 480

GW19D1 TAACATCGTTCATAATGTTTATAACATCTCATATTTTATGAATGTAAAATTAAAACGAAA 479

GW11D12 TAACATCGTTCATAATGTTTATAACATCTCATATTTTATGAATGTAAAATTAAAACGAAA 480

GW14A11 TAACATCGTTCATAATGTTTATAACATCTCATATTTTATGAATGTAAAATTAAAACGAAA 480

GW1E3 TAACATCGTTCATAATGTTTATAACATCTCAT**G**TTTTATGAATGTAAAATTAAAACGAAA 479

GW13B3 TAACATCGTTCATAATGTTTATAACATCTCAT**G**TTTTATGAATGTAAAATTAAAACGAAA 476

G17F5 TAACATC**A**TTCATAATGTTTATAACATCTCAT**G**TTTTATGAATGTAAAATTAAAACGAAA 480

GW11B1 TAACATC**A**TTCATAATGTTTATAACATCTCAT**G**TTTTATGAATGTAAAATTAAAACGAAA 480

GW10F6 AAATTTCAATAAAAATCGAAATTTCTTCATTTGAAAACTAAC 522

GW19D1 AAATTTCAATAAAAATCGAAATTTCTTCATTTGAAAAC---- 517

GW11D12 AAATTTCAATAAAAATCGAAATTTCTTCATTTGAAAACT--- 519

GW14A11 AAATTTCAATAAAAATCGAAATTTCTTCATTTGAAAACTAAC 522

GW1E3 AAATTTCAATAAAAATCGAAATTTCTTCATTTGAAAAC---- 517

GW13B3 AAATTTCAATAAAAATCGAAATTTCTTCATTTG--------- 509

G17F5 AAATTTCAATAAAAATCGAAATTTCTTCATTTG--------- 513

GW11B1 AAATTTCAATAAAAATCGAAATTTCTTCATTTGAAAACT--- 519

**L**

G14D9 --CTTTTCACTTTAGAATCTGACAAGCAAACATCCACAAAA**ATG**GCAAAACGTACAAAGA 58

G20B3 --CTTTTCGCTTTAGAATCTGACAAGCAAACATCCACAAAA**ATG**GCAAAACGTACAAAGA 58

L14C4 CTCTTTTCACTTTAGAATCTGACAAGCAAACATCCACAAAA**ATG**GCAAAACGTACAAAGA 60

GW15H2 -------------------TGACAAGCAAACATCCACAAAA**ATG**GCAAAACGTACAAAGA 41

GW1G4 --------------------------CAAACATCCACAAAA**ATG**GCAAAACGTACAAAGA 34

G9F8 CTCTTTTCGCTTTAGAATCTGACAAGCAAACATCCACAAAA**ATG**GCAAAACGTACAAAGA 60

G14D9 AAGTTGGAATCGTCGGTAAATATGGTACCCGTTATGGTGCCTCACTCCGTAAGATGGTGA 118

G20B3 AAGTTGGAAT**T**GTCGGTAAATATGGTACCCGTTATGGTGCCTCACTCCGTAAGATGGTGA 118

L14C4 AAGTTGGAATCGTCGGTAAATATGGTACCCGTTATGGTGCCTCACTCCGTAAGATGGTGA 120

GW15H2 AAGTTGGAATCGTCGGTAAATATGGTACCCGTTATGGTGCCTCACTCCGTAAGATGGTGA 101

GW1G4 AAGTTGGAATCGTCGGTAAATATGGTACCCGTTATGGTGCCTCACTCCGTAAGATGGTGA 94

G9F8 AAGTTGGAATCGTCGGTAAATATGGTACCCGTTATGGTGCCTCACTCCGTAAGATGGTGA 120

G14D9 AAAAAATCGAAATTACCCAGCATTCGAAATATACATGCTCTTTCTGTGGAAAGGACTCTA 178

G20B3 AAAAAATCGAAATTACCCAGCATTCGAAATATACATGCTCTTTCTGTGGAAAGGACTCTA 178

L14C4 AAAAAATCGAAATTACCCAGCATTCGAAATATACATGCTCTTTCTGTGGAAAGGACTCTA 180

GW15H2 AAAAAATCGAAATTACCCAGCATTCGAAATATACATGCTCTTTCTGTGGAAAGGACTC**A**A 161

GW1G4 AAAAAATCGAAATTACCCAGCATTCGAAATATACATGCTCTTTCTGTGGAAAGGACTC**A**A 154

G9F8 AAAAAATCGAAATTACCCAGCATTCGAAATATACATGCTCTTTCTGTGGAAAGGACTC**A**A 180

G14D9 TGAAACGTGCTGTTGTCGGAATTTGGTCATGCAATCGATGCAAACGTACCGTTGCTGGTG 238

G20B3 TGAAACGTGCTGTTGTCGGAATTTGGTCATGCAATCGATGCAAACGTACCGTTGCTGGTG 238

L14C4 TGAAACGTGCTGTTGTCGGAATTTGGTCATGCAATCGATGCAAACGTACCGTTGCTGGTG 240

GW15H2 TGAAACGTGCTGTTGTCGGAATTTGGTCATGCAATCGATGCAAACGTACCGTTGCTGGTG 221

GW1G4 TGAAACGTGCTGTTGTCGGAATTTGGTCATGCAATCGATGCAAACGTACCGTTGCTGGTG 214

G9F8 TGAAACGTGCTGTTGTCGGAATTTGGTCATGCAATCGATGCAAACGTACCGTTGCTGGTG 240

G14D9 GTGCTTGGGTATACTCAACCACAGCCGCTGCTTCAGTTCGATCAGCTGT**T**CGTCGTCTCC 298

G20B3 GTGCTTGGGTATACTCAACCACAGCCGCTGCTTCAGTTCGATCAGCTGT**T**CGTCGTCTCC 298

L14C4 GTGCTTGGGTATACTCAACCACAGCCGCTGCTTCAGTTCGATCAGCTGTCCGTCGTCTCC 300

GW15H2 GTGCTTGGGTATACTCAACCACAGCCGCTGCTTCAGTTCGATCAGCTGTCCGTCGTCTCC 281

GW1G4 GTGCTTGGGTATACTCAACCACAGCCGCTGCTTCAGTTCGATCAGCTGTCCGTCGTCTCC 274

G9F8 GTGCTTGGGTATACTCAACCACAGCCGCTGCTTCAGTTCGATCAGCTGTCCGTCGTCTCC 300

G14D9 GTGACACCAAAGAACAG**TAA**ACCGATGCACACTGTGTTTTTTTTTT**T**GTTTTCTCTTCAA 358

G20B3 GTGACACCAAAGAACAG**TAA**ACCGATGCACACTGTGTTTTTTTTTT-GTTTTCTCTTCAA 357

L14C4 GTGACACCAAAGAACAG**TAA**ACCGATGCACACTGTGTTTTTTTTTT-GTTTTCTCTTCAA 359

GW15H2 GTGACACCAAAGAACA**ATAA**ACCGATGCACACTGTGTTTTTTTTTT-GTTTTCTCTTCAA 340

GW1G4 GTGACACCAAAGAACA**ATAA**ACCGATGCACACTGTGTTTTTTTTTT-GTTTTCTCTTCAA 333

G9F8 GTGACACCAAAGAACA**ATAA**ACCGATGCACACTGTGTTTTTTTTTT**T**GTTTTCTCTTCAA 360

G14D9 TAAATTAAATTGAAAGCAAAAATCCACC-------------------------------- 386

G20B3 TAAATTAAATTGAAAGCAAAAATCCACCAAAAATCAAAT**GGAT**T**CGA**AAAA**GTGATGTGA** 417

L14C4 TAAATTAAATTGAAAGCAAAAATCCACCAAAAATCAAAT**GGAT**T**CGA**AAAA**GTGATGTGA** 419

GW15H2 TAAATTAAATTGAAAGCAAAAATCCACCAAAAATCAAATTGGGTTCGAAAAAGTGATGTG 400

GW1G4 TAAATTAAATTGAAAGCAAAAATCCACCAAAAATCAAATTGGGTTCGAAAAAGTGATGTG 393

G9F8 TAAATTAAATTGAAAGCAAAAATCCACCAAAAATCAAATTGGGTTCGAAAAAGTGATGTG 420

G14D9 ------------------------------------------------------

G20B3 **T**TTTCCAAAACACAATTCCAATTGTGAATAAAACAA**A**TTCCAACACGTTTTCA- 470

L14C4 **T**TTTCCAAAACACAATTCCAATTGTGAATAAAACAA**A**TTCCAACACGTTTTCA- 472

GW15H2 ATTTCCAAAACACAATTCCAATTGTGAATAAAACAATTTCCAACACGTTTTCA- 453

GW1G4 ATTTCCAAAACACAATTCCAATTGTGAATAAAACAATTTCCAACACGTTTT--- 444

G9F8 ATTTCCAAAACACAATTCCAATTGTGAATAAAACAATTTCCAACACGTTTTCAT 474

**M**

GW17C11 -------------------GTGAAGCATCAGGACGGTGTGCGAATTGCAATTCTTGTCTA 41

GW5A1 --------------------------ATCAGGACGGTGTGCGAATTGCAATTCTTGTCTA 34

G7E4 TT**T**TCTCTTTTTTAAACGTGTGAAGCATCAGGACGGTGTGCGAATTGCAATTCTTGTCTA 59

G12A9 TTCTTTCTTTTTTAAACGTGTGAAGCATCAGGACGGTGTGCGAATTGCAATTCTTGTCTA 60

S18F11 ------CTTTTTTAAACGTGTGAAGCATCAGGACGGTATGCGAATTGCAATTCTTGTCTA 55

S18F10 ------CTTTTTTAAACGTGTGAAGCATCAGGACGGTATGCGAATTGCAATTCTTGTCTA 55

G38H12 -------TTTTCCAAACGTGT**T**AA**ACG**TCA**A**GA**A**GGT**G**TGC**ATT**TT**CACTG**TCT**C**GT**A**TA 53

G38F12 --------TTTCCAAACGTGT**T**AA**ACG**TCA**A**GA**A**GGT**G**TGC**ATT**TT**CACTG**TCT**C**GT**A**TA 52

L6C1 -------TTTTCCAAACGTGT**T**AA**ACG**TCA**A**GA**A**GGT**G**TGC**ATT**TT**CACTG**TCT**C**GT**A**TA 53

GW17C11 ATTTGAAATAATATAA**AGTTGA**AAAA**ATG**CCCAAAGAAATCAAAGAAGTGAAGGATTTTC 101

GW5A1 ATTTGAAATAATATAA**AGTTGA**AAAA**ATG**CCCAAAGAAATCAAAGAAGTGAAGGATTTTC 94

G7E4 ATTTGAAATAATATAA**AGTTGA**AAAA**ATG**CCCAAAGAAATCAAAGAAGTGAAGGATTTTC 119

G12A9 ATTTGAAATAATATAA**AGTTGA**AAAA**ATG**CCCAAAGAAATCAAAGAAGTGAAGGATTTTC 120

S18F11 ATTTGAAATAATATAA**AGTTGA**AAAA**ATG**CCCAAAGAAATCAAAGAAGTGAAGGATTTTC 115

S18F10 ATTTGAAATAATATAA**AGTTGA**AAAA**ATG**CCCAAAGAAATCAAAGAAGTGAAGGATTTTC 115

G38H12 ATTT**ACG**A**A**A**T**TA**A**AA------**C**AAA**ATG**CC**A**AAAGAAATCAAAGAAGT**T**AA**A**GATTTTC 107

G38F12 ATTT**ACG**A**A**A**T**TA**A**AA------**C**AAA**ATG**CC**A**AAAGAAATCAAAGAAGT**T**AA**A**GATTTTC 106

L6C1 ATTT**ACG**A**A**A**T**TA**A**AA------**C**AAA**ATG**CC**A**AAAGAAATCAAAGAAGT**T**AA**A**GATTTTC 107

GW17C11 TTATTAAAGCACGCCGAAAGGATGCTCGTGCCGTTAAAATAAAGAAGAATCCATTGAATA 161

GW5A1 TTATTAAAGCACGCCGAAAGGATGCTCGTGCCGTTAAAATAAAGAAGAATCCATTGAATA 154

G7E4 TTATTAAAGCACGCCGAAAGGATGCTCGTGCCGTTAAAATAAAGAAGAATCCATTGAATA 179

G12A9 TTATTAAAGCACGCCGAAAGGATGCTCGTGCCGTTAAAATAAAGAAGAATCCATTGAATA 180

S18F11 TTATTAAAGCACGCCGAAAGGATGCTCGTGCCGTTAAAATAAAGAAGAATCCATTGAATA 175

S18F10 TTATTAAAGCACGCCGAAAGGATGCTCGTGCCGTTAAAATAAAGAAGAATCCATTGAATA 175

G38H12 T**A**AT**C**AAAGCACGCCGAAAGGA**C**GC**C**CGTGCCGTTAAAATAAAGAA**A**AATCCA**GAA**AA**C**A 167

G38F12 T**A**AT**C**AAAGCACGCCGAAAGGA**C**GC**C**CGTGCCGTTAAAATAAAGAA**A**AATCCA**GAA**AA**C**A 166

L6C1 T**A**AT**C**AAAGCACGCCGAAAGGATGC**C**CGTGCCGTTAAAATAAAGAA**A**AATCCA**GAA**AA**C**A 167

GW17C11 CGAAATTCAAGATTCGTTGTTCCCGCTTTTTGTACACATTGGTTGTAGCTGACAAGGAAA 221

GW5A1 CGAAATTCAAGATTCGTTGTTCCCGCTTTTTGTACACATTGGTTGTAGCTGACAAGGAAA 214

G7E4 CGAAATTCAAGATTCGTTGTTCCCGCTTTTTGTACACATTGGTTGTAGCTGACAAGGAAA 239

G12A9 CGAAATTCAAGATTCGTTGTTCCCGCTTTTTGTACACATTGGTTGTAGCTGACAAGGAAA 240

S18F11 CGAAATTCAAGATTCGTTGTTCCCGCTTTTTGTACACATTGGTTGTAGCTGACAAGGAAA 235

S18F10 CGAAATTCAAGATTCGTTGTTCCCGCTTTTTGTACACATTGGTTGTAGCTGACAAGGAAA 235

G38H12 C**C**AAATTCAAGAT**C**CGTTG**C**TC**A**CG**T**TTTTTGTACACATTGGTTGTTGC**C**GA**T**AAGGAAA 227

G38F12 C**C**AAATTCAAGAT**C**CGTTG**C**TC**A**CG**T**TTTTTGTACACATTGGTTGTTGC**C**GA**T**AAGGAAA 226

L6C1 C**C**AAATTCAAGAT**C**CGTTG**C**TC**A**CG**T**TTTTTGTACACATTGGTTGTTGC**C**GA**T**AAGGAAA 227

GW17C11 AGGCCGATAAATTGAAACAATCATTGCCACCAGGTCTTCAAGTTAAAGAAGTCAAA**TAA**A 281

GW5A1 AGGCCGATAAATTGAAACAATCATTGCCACCAGGTCTTCAAGTTAAAGAAGTCAAA**TAA**A 274

G7E4 AGGCCGATAAATTGAAACAATCATTGCCACCAGGTCTTCAAGTTAAAGAAGTCAAA**TAA**A 297

G12A9 AGGCCGATAAATTGAAACAATCATTGCCACCAGGTCTTCAAGTTAAAGAAGTCAAA**TAA**A 300

S18F11 AGGCCGATAAATTGAAACAATCATTGCCACCAGGTCTTCAAGTTAAAGAAGTCAAA**TAA**A 295

S18F10 AGGCCGATAAATTGAAACAATCATTGCCACCAGGTCTTCAAGTTAAAGAAGTCAAA**TAA**A 295

G38H12 AGGC**T**GA**A**AAATTGAA**G**C**G**ATCATTGCCACCAGGTCTTCAAGTTAAAGAAGTCAA**GTAA**A 287

G38F12 AGGC**T**GA**A**AAATTGAA**G**C**G**ATCATTGCCACCAGGTCTTCAAGTTAAAGAAGTCAA**GTAA**A 286

L6C1 AGGC**T**GA**A**AAATTGAA**G**C**A**ATCATTGCCACCAGGTCTTCAAGTTAAAGAAGTCAA**GTAA**A 287

GW17C11 TA------TTTCAATCA-TGTAAATAAATTG**A**AGGACAACTTCGTTAGCCA**A**CAGTTATC 334

GW5A1 TA------TTTCAATCA-TGTAAATAAATTG**A**AGGACAACTTCGTTAGCCACCAGTTATC 327

G7E4 TA------TTTCAATCA-TGTAAATAAATTG**A**AGGACAACTTCGTTAGCCACCAGTTATC 350

G12A9 TA------TTTCAATCA-TGTAAATAAATTG**A**AGGACAACTTCGTTAGCCA**A**CAGTTATC 353

S18F11 TA------TTTCAATCA-TGTAAATAAATTG**A**AGGACAACTTCGTTAGCCACCAGTTATC 348

S18F10 TA------TTTCAATCA-TGTAAATAAATTG**A**AGGACAACTTCGTTAGCCACCAGTTATC 348

G38H12 T**CATTGAT**TTT**T**AATCA**AC**G**GGGGGCGGG**TG-**GAA**A**A**AAC**A**TC**A**T**C**A-C**A**ACC**CAAAGC**C 345

G38F12 T**CATTGAT**TTT**T**AATCA**AC**G**GGGGGCGGG**TG-**GAA**A**A**AAC**A**TC**A**T**C**A-C**A**ACC**CAAAGC**C 344

L6C1 T**CATTGAT**TTT**T**AATCA**AC**G**GGGGGCGGG**TG**AGAA**A**A**AAC**AC**C**A**T**C**A-C**A**ACC**CAAAGC**C 346

GW17C11 CGTTTAAT-------------------------------------- 342

GW5A1 CGTTTAAT**CCAACGAAAACA**T**CT**TTT-------------------- 353

G7E4 CGTTTAAT-------------------------------------- 358

G12A9 CGTTTAAT-------------------------------------- 361

S18F11 CGTTTAAT-------------------------------------- 356

S18F10 CGTTTAAT-------------------------------------- 356

G38H12 C**AA**T**A**AA**G**AGTTTTTTCTTTTTGTTTTTAATT-------------- 377

G38F12 C**AA**T**A**AA**G**AGTTTTTTCTTTTTGTTTTTAATT-------------- 376

L6C1 C**AA**T**A**AA**G**AGTTTTTTCTTTTTGTTTTTAATTCACAAAAATTAAAT 392

**N**

L15F5 CGAAGAACAATCGAT**C**AAA**ATG**GTGAATGTTCCCAAACAGCGCCGGACTTTTTGCAAAAA 60

GW9E2 ----GAACAATCGAT**C**AAA**ATG**GTGAATGTTCCCAAACAGCGCCGGACTTTTTGCAAAAA 56

GW16H5 CGAAGAACAATC**T**AT-AAA**ATG**GTGAATGTTCCCAAACAGCGCCGGACTTTTTGCAAAAA 59

GW10F9 CGAAGAACAATC**T**AT-AAA**ATG**GTGAATGTTCC**A**AAACAGCGCCGGACTTTTTGCAAAAA 59

S15C10 ------------------------------------------------------------

L15F5 ATGCAAAGTGCACAAGTTGCACAAAGTCACACAGTACAAAAAATCGAAGGAACGTACTGC 120

GW9E2 ATGCAAAGTGCACAAGTTGCACAAAGTCACACAGTACAAAAAATCGAAGGAACGTACTGC 116

GW16H5 ATGCAAAGTGCACAAGTTGCACAAAGTCACACAGTACAAAAAATCGAAGGAACGTACTGC 119

GW10F9 ATGCAAAGTGCACAAGTTGCACAAAGTCACACAGTACAAAAAATCGAAGGAACGTACTGC 119

S15C10 ----------------------------------------------------------GC 2

L15F5 CTCACAAGGACGTCGTCGTTACGATCGTAAGCAACAAGGTTTCGGTGGTCAAACCAAACC 180

GW9E2 CTCACAAGGACGTCGTCGTTACGATCGTAAGCAACAAGGTTTCGGTGGTCAAACCAAACC 176

GW16H5 CTCACAAGGACGTCGTCGTTACGATCGTAA**A**CAACAAGGTTTCGGTGGTCAAACCAAACC 179

GW10F9 CTCACAAGGACGTCGTCGTTACGATCGTAAGCAACAAGGTTTCGGTGGTCAAACCAAACC 179

S15C10 CTCACAAGGACGTCGTCGTTACGATCGTAAGCAACAAGGTTTCGGTGGTCAAACCAAACC 62

L15F5 AATCTTCAGAAAGAAGGCAAAGACAACCAAAAAAATTGTCCTTCGTATGGAATGTACTGA 240

GW9E2 AATCTTCAGAAAGAAGGCAAAGACAACCAAAAAAATTGTCCTTCGTATGGAATGTACTGA 236

GW16H5 AATCTTCAGAAAGAAGGCAAA**A**ACAACCAAAAAAATTGTCCTTCGTATGGAATGTACTGA 239

GW10F9 AATCTTCAGAAAGAAGGCAAA**A**ACAACCAAAAAAATTGTCCTTCGTATGGAATGTACTGA 239

S15C10 AATCTTCAGAAAGAAGGCAAAGACAACCAAAAAAATTGTCCTTCGTATGGAATGTACTGA 122

L15F5 ATGCAAGTACCGCAAACAAAAGCCAATCAAACGTTGCAAACATTTCGAATTGGGTGGAGA 300

GW9E2 ATGCAAGTACCGCAAACAAAAGCCAATCAAACGTTGCAAACATTTCGAATTGGGTGGAGA 296

GW16H5 ATGCAA**A**TACCGCAAACAAAAGCCAATCAAACGTTGCAAACATTTCGAATTGGGTGGAGA 299

GW10F9 ATGCAA**A**TACCGCAAACAAAAGCCAATCAAACGTTGCAAACATTTCGAATTGGGTGGAGA 299

S15C10 ATGCAAGTACCGCAAACAAAAGCCAATCAAACGTTGCAAACATTTCGAATTGGGTGGAGA 182

L15F5 CAAAAAACGCAAGGGTCAAATGATCCAATTC**TAA**ACGATTTCCTATCGCTTCATTTCCAT 360

GW9E2 CAAAAAACGCAAGGGTCAAATGATCCAATTC**TAA**ACGATTTCCTATCGCTTCATTTCCAT 356

GW16H5 CAAAAAACGCAAGGGTCAAATGATCCAATTC**TAA**ACGATTT**T**CT**G**TCGCTTCATTTCCAT 359

GW10F9 CAAAAAACGCAA**A**GGTCAAATGATCCAATTC**TAA**ACGATTT**T**CT**G**CCGCTTCATTTCCAT 359

S15C10 CAAAAAACGCAAGGGTCAAATGATCCAATTC**TAA**ACGGTTTCCTATCGCTTCATTTCCAT 242

L15F5 CAGTTTTATGTTTAACGATTTTTTAGTTATTGTTTGTAA**T**CATGTGGAACTACAATAACA 420

GW9E2 CAGTTTTATGTTTAACGATTTTTTAGTTATTGTTTGTAA**T**CATGTGGAACTACAATAACA 416

GW16H5 CAGTTTTATGTTTAACGATTTTTTAGTTATTGTTTGTAACCATGTGGAACTACAATAACA 419

GW10F9 CAGTTTTATGTTTAACGATTTTTTAGTTATTGTTTGTAACCATGTGGAACTACAATAACA 419

S15C10 CAGTT**C**TATGTTTAACGATTTTTTAGTTATTGTTTG**C**AACCATGTGGAACTACAATAACA 302

L15F5 TCAAACGTTTTGCTGGGAAATAATAAAATCTTATTTCGATGCAAATTGAAGGTTAAGTTA 480

GW9E2 TCAAACGTTTTGCTGGGAAATAATAAAATCTTATTTCGATGCAAATTGAAGGTTAAGTTA 476

GW16H5 TCAAACGTTTTGCTGGGAAATAATAAAATCTTATTTCGATGCAAATTGAAGGTTAAGTTA 479

GW10F9 TCAAACGTTTTGCTGGGAAATAATAAAATCTTATTTCGATGCAAATTGAAGGTTAAGTTA 479

S15C10 TCAAACGTTTTGCT**A**GGAAATAATAAAATCTTATTTCGATGCAAAT**C**GAAGGTTAAGTTA 362

L15F5 TTCCAAACTTAAATTTCGTTTGTGAATGAAAAGAAAAATAATAAT 525

GW9E2 TTCCAAACTTAAATTTCGTTTGTGAATG----------------- 504

GW16H5 TTCCAAACTTAAATTTCGTTTGTGAATGAAAAGAAAAAT------ 518

GW10F9 TT**T**CAAACTTAAA**A**TTC**A**TTTGTGAATGAAAAGAAAAATAATA-- 522

S15C10 TTCC**G**AA**A**TTAAA**A**T**A**C**A**TTTGTGAATGAAAAGAAAAATAATAAT 407

**O**

L10C3 --------TTGTAAGAAGTACATTACAATC**ATG**GCATTGCCAATATCTAAAAAACGAAAG 52

GW13B10 ---GTTCTTTGTAAGAAGTACATTACAATC**ATG**GCATTGCCAATATCTAAAAAACGAAAG 57

GW16D10 CACGTTCTTTGTAAGAAGTACATTACAATC**ATG**GCATTGCCAATATCTAAAAAACGAAAG 60

L10C3 TTCGTATTGAACGGAATCTTCAAAGCTGAATTGAACGAGTTCTTGACCCGTCAATTGGCC 112

GW13B10 TTCGTATTGAACGGAATCTTCAAAGCTGAATTGAACGAGTTCTTGACCCGTCAATTGGCC 117

GW16D10 TTCGTATTGAACGGAATCTTCAAAGCTGAATTGAACGAGTTCTTGACCCGTCAATTGGCC 120

L10C3 GAAGATGGTTACTCAGGAGTTGAAATTCGTGTCAC**T**CCAACACGTACCGAAATCATTATC 172

GW13B10 GAAGATGGTTACTCAGGAGTTGAAATTCGTGTCACACCAACACGTACCGAAATCATTATC 177

GW16D10 GAAGATGGTTACTCAGGAGTTGAAATTCGTGTCACACCAACACGTACCGAAATCATTATC 180

L10C3 ATGGCCACAAAAACATTGAATGTTTTGGGAGAAAAAGGTCGCCGTATTCGTGAACTTACA 232

GW13B10 ATGGCCACAAAAACATTGAATGTTTTGGGAGAAAAAGGTCGCCGTATTCGTGAACTTACA 237

GW16D10 ATGGCCACAAAAACATTGAATGTTTTGGGAGAAAAAGGTCGCCGTATTCGTGAACTTACA 240

L10C3 GCTGTTGTACAAAAAAGATTCGGTTTTGCACCGGGCACCGTTGAATTGTATGCCGAAAAG 292

GW13B10 GCTGTTGTACAAAAAAGATTCGGTTTTGCACCGGGCACCGTTGAATTGTATGCCGAAAAG 297

GW16D10 GCTGTTGTACAAAAAAGATTCGGTTTTGCACCGGGCACCGTTGAATTGTATGCCGAAAAG 300

L10C3 GT**T**GCTACACGTGGTTTGTGCGCCATTGCACAAGCTGAATCATTGCGTTATAAATTGACC 352

GW13B10 GTAGCTACACGTGGTTTGTGCGCCATTGCACAAGCTGAATCATTGCGTTATAAATTGACC 357

GW16D10 GTAGCTACACGTGGTTTGTGCGCCATTGCACAAGCTGAATCATTGCGTTATAAATTGACC 360

L10C3 GGTGGTTTGGCTGTTCGTCGTGCATGCTATGGTGTTTTGCGTTTCATTATGGAATCCGGT 412

GW13B10 GGTGGTTTGGCTGTTCGTCGTGCATGCTATGGTGTTTTGCGTTTCATTATGGAATCCGGT 417

GW16D10 GGTGGTTTGGCTGTTCGTCGTGCATGCTATGGTGTTTTGCGTTTCATTATGGAATCCGGT 420

L10C3 GC**C**AAGGGCTGTGAAGTTGTTGTATCTGGTAAATT**G**CGTGGTCAACGTGCCAAATCAATG 472

GW13B10 GCTAAGGGCTGTGAAGTTGTTGTATCTGGTAAATTACGTGGTCAACGTGCCAAATCAATG 477

GW16D10 GCTAAGGGCTGTGAAGTTGTTGTATCTGGTAAATTACGTGGTCAACGTGCCAAATCAATG 480

L10C3 AAATTCGTTGATGGTTTGATGATACATTCGGGTGATCCATGTAATGCATATGTTGATTCT 532

GW13B10 AAATTCGTTGATGGTTTGATGATACATTCGGGTGATCCATGTAATGCATATGTTGATTCT 537

GW16D10 AAATTCGTTGATGGTTTGATGATACATTCGGGTGATCCATGTAATGCATATGTTGATTCT 540

L10C3 GCCACACGTCATGTATTATTGCGTCAAGGTGTACTTGGTATCAAAGTTAAAATCATGTTG 592

GW13B10 GCCACACGTCATGTATTATTGCGTCAAGGTGTACTTGGTATCAAAGTTAAAATCATGTTG 597

GW16D10 GCCACACGTCATGTATTATTGCGTCAAGGTGTACTTGGTATCAAAGTTAAAATCATGTTG 600

L10C3 CCATATGATCCAAATGGCAAAATTGGACCAAAGAAACCATTGCCAGACAATGTTACGGTC 652

GW13B10 CCATATGATCCAAATGGCAAAATTGGACCAAAGAAACCATTGCCAGACAATGTTACGGTC 657

GW16D10 CCATATGATCCAAATGGCAAAATTGGACCAAAGAAACCATTGCCAGAC**G**ATGTTACGGTC 660

L10C3 ATTGAACCAAAAGAGGAGCAAATTTATGAACAGCCAGAAACTGAATATAAAACACCACAA 712

GW13B10 ATTGAACCAAAAGAGGAGCAAATTTATGAACAGCCAGAAACTGAATATAAAACACCACAA 717

GW16D10 ATTGAACCAAAAGAGGAGCAAATTTATGAACAGCC**C**GAAACTGAATATAA**G**ACACCACAA 720

L10C3 GCAGCGGCTAGCGAACCAGTTCATGAAGCTATCGAA**TAA**ATTGATAACACACAAACAAAT 772

GW13B10 GCAGCGGCTAGCGAACCAGTTCATGAAGCTATCGAA**TAA**ATTGATAACACACAAACAAAT 777

GW16D10 GCAGCGGCTAGCGAACCAGTTCATGAAGCTATCGAA**TAA**ATTGATAACACACAAACAAAT 780

L10C3 AAATGTTATTAAAACCAAAATATTATTTGGTTTTAGCATTTAATCAATAAAAATGAAAAA 832

GW13B10 AAATGTTATTAAAACCAAAATATTATTTGGTTTTAGCATTTAATCAATAAAAATGAAAAA 837

GW16D10 ------------------------------------------------------------

L10C3 CAATTT--------- 838

GW13B10 CAATTTACAAAAACC 852

GW16D10 ---------------

**P**

**(5‘ truncate)**

GW14F1 ATCAACATGGCAGTCGGAAAAAATAAAGGTCTCTCGAAGGGAGGCAAAAAAGGATCAAAA 60

S33B10 ------------------------------------------------------------

GW8C6 -------------TCGGAAAAAATAAAGGTCTCTCGAAGGGAGGCAAAAAAGGATCAAAA 47

L31G3 -------------TCGGAAAAAATAAAGGTCTCTCGAAGGGAGGCAAAAAAGGATCAAAA 47

GW11E11 ----------------------ATAA**G**GGTCT**A**TC**C**AA**A**GG**T**GG**T**AAAAA**G**GGATC**G**AAA 38

GW14F1 AAGAAGGTTGTTGATCCATTTTCTCGCAAAGATTGGTATGATATCAAAGCACCAAATATG 120

S33B10 ------------------------------------------------------------

GW8C6 AAGAAGGTTGTTGATCCATTTTCTCGCAAAGATTGGTATGATATCAAAGCACCAAATATG 107

L31G3 AAGAAGGTT**A**T**C**GATCCATTTTC**C**CGCAAAGATTGGTATGATATCAAA**A**CACCAAA**C**ATG 107

GW11E11 AAGAAGGTT**A**T**C**GATCC**C**TTTTC**C**CG**T**AAAGATTGGTATGATATCAAAGCACCAAA**C**ATG 98

GW14F1 TTTGCCCAACGGCAAGTTGGAAAAACATTGGTAAATCGTACACAAGGTACACGAATTGCA 180

S33B10 ------------------------------------------------------------

GW8C6 TTTGCCCAACGGCAAGTTGGAAAAACATTGGTAAATCGTACACAAGGTACACGAATTGCA 167

L31G3 TTTGCCCAACGGCAAGTTGGAAAAACATTGGTAAA**C**CGTACACAAGGTACACGAATTGCA 167

GW11E11 TTT**T**C**T**CAACG**T**CAAGTTGGAAAAACATTGGT**T**AA**C**CGTAC**C**CAAGGTAC**C**CGAATTGCA 158

GW14F1 TCAGATGGTTT**G**AAACACCGCGTGTACGAAATTTCATTGGCCGATTTACAAAACGACAAT 240

S33B10 ------------------------------------------------------------

GW8C6 TCAGATGGTTT**G**AAACACCGCGTGTACGAAAT**T**TCATTGGCCGATTTACAAAACGACAAT 227

L31G3 TCAGATGGTTTAAAACACCGCGTGTACGAAATATCATTGGCCGATTTACAAAACGATAAT 227

GW11E11 TC**G**GATGGTTTAAAACACCGCGTGTA**T**GAA**G**TATCATTGGCTGATTTACAAAACGATAAT 218

GW14F1 GATGCTGAACGTTCGTTCCGTAAATTCCGTTTGGTTTGTGAAGATGTTCAAGGCAGCCAC 300

S33B10 ------------------------------------------------------------

GW8C6 GATGCTGAACGTTCGTTCCGTAAATTCCGTTTGGTTTGTGAAGATGTTCAAGGCAGCCAC 287

L31G3 GATGCTGAACGTTC**A**TTCCGTAAATT**T**CGTTTG**A**TTTG**C**GAAGATGTTCAAGG**T**AGCCAC 287

GW11E11 GATGCTGAACGTTC**A**TTCCGTAAATT**T**CGTTTG**A**TTTG**C**GAAGATGTTCAAGG**T**AGCCAC 278

GW14F1 TGTTTAACCAATTTCCATGGCATGAATTTAACCACCGATAAATTGCGTTCAATGGTGAAA 360

S33B10 -------------------GCATGAATTTAACCACCGATAAATTGCGTTCAATGGTGAAA 41

GW8C6 TGTTTAACCAATTTCCATGGCATGAATTTAACCACCGATAAATTGCGTTCAATGGTGAAA 347

L31G3 TGTTTA**TGT**AATTTTCATGGCATGAATTT**G**ACCACCGA**C**AAATTGCGTTCAATGGT**C**AAG 347

GW11E11 TGTTTA**TGT**AATTTTCATGGCATGAATTT**G**ACCACCGA**C**AAATTGCGTTCAATGGT**C**AAG 338

GW14F1 AAATGGCAAACATTGATTGAAGCCAGCATTGATGTAAAAACAACTGACGGTTACTTGCTT 420

S33B10 AAATGGCAAACATTGATTGAAGCCAGCATTGATGTAAAAACAACTGACGGTTACTTGCTT 101

GW8C6 AAATGGCAAACATTGATTGAAGCCAGCATTGATGTAAAAACAACTGACGGTTACTTGCTT 407

L31G3 AAATGGCAAACATTGATTGAA**TG**CA**A**CATTGATGT**C**AAAAC**C**ACTGACGGTTACTTGCTT 407

GW11E11 AAATGGCAAACATTGATTGAA**TG**CA**A**CATTGATGT**C**AAAAC**C**ACTGACGGTTACTTGCTT 398

GW14F1 CGTGTTTTCTGCATCGGTTTCACCACAAAAGATGCCATGACATCGCGCAAAACATGCTAT 480

S33B10 CGTGTTTTCTGCATCGGTTTCACCACAAAAGATGCCATGACATCGCGCAAAACATGCTAT 161

GW8C6 CGTGTTTTCTGCATCGGTTTCACCACAAAAGATGCCATGACATCGCGCAAAACATGCTAT 467

L31G3 CGTGT**A**TTCTGCAT**T**GG**A**TT**T**ACC**G**C**C**AA**G**GAT**C**C**A**ATG**T**C**TCAT**CGCAAAACAT**T**CTA**C** 467

GW11E11 CGTGT**A**TTCTGCAT**T**GG**A**TT**T**ACC**G**C**C**AA**G**GAT**C**C**A**ATG**T**C**TCAT**CGCAAAACAT**T**CTA**C** 458

GW14F1 GCTCAGCACTCACAAACTCGTGCCATCCGTAAAAAGATGTGCGAAATTATTACACGTGAT 540

S33B10 GCTCAGCACTCACAAACTCGTGCCATCCGTAAAAAGATGTGCGAAATTATTACACGTGAT 221

GW8C6 GCTCAGCACTCACAAACTCGTGCCATCCGTAAAAAGATGTGCGAAATTATTACACGTGAT 527

L31G3 GC**A**CA**A**CACTCACAA**GT**TCGTGCCATCCGTAA**G**AAGATG**A**GCGACATTATTA**TT**CGTGAT 527

GW11E11 GC**A**CA**A**CACTCACAA**GT**TCGTGCCATCCGTAA**G**AAGATG**A**GCGACATTATTA**TT**CGTGAT 518

GW14F1 GTGTCCAGCTCTGACTTGAAAGAAGTTGTCAACAAATTGTTGCCGGACTCAATTGCTAAA 600

S33B10 GTGTCCAGCTCTGACTTGAAAGAAGTTGTCAACAAATTGTTGCC**A**GACTCAATTGCTAAA 281

GW8C6 GTGTCCAGCTCTGACTTGAAAGAAGTTGTCAACAAATTGTTGCCGGACTCAATTGCTAAA 587

L31G3 GTG**A**CCAG**T**TCTGACTTGAAAGAAGTTGTCAACAAATTGTTGCC**C**GA**T**TC**C**ATTGCTAA**G** 587

GW11E11 GTG**A**CCAG**T**TCTGACTTGAAAGAAGTTGTCAACAAATTGTTGCC**C**GA**T**TC**C**ATTGCTAA**G** 578

GW14F1 GATATTGAAAAGGCATGCCACAGCATCTATCCATTGCACGATGTATACATCCGTAA**G**GTG 660

S33B10 GATATTGAAAAGGCATGCCACAGCATCTATCCATTGCACGATGTATACATCCGTAAAGTG 341

GW8C6 GATATTGAAAAGGCATGCCACAGCATCTATCCATTGCACGATGTATACATCCGTAAAGTG 647

L31G3 GATATTGAAAAGGC**C**TG**T**CA**TG**G**T**ATCTATCCA**C**T**T**CA**T**GATGT**T**TACAT**T**CGTAAAGT**T** 647

GW11E11 GATATTGAAAAGGC**C**TG**T**CA**TG**G**T**ATCTATCCA**C**T**T**CA**T**GATGT**T**TACAT**T**CGTAAAGT**T** 638

GW14F1 AAAGTGTTGAAAAAACCACGTTTTGATTTATCAAGATTGTTGGAATTGCACGGTGACGGA 720

S33B10 AAAGTGTTGAAAAAACCACGTTTTGATTTATCAAGATTGTTGGAATTGCACGGTGACGGA 401

GW8C6 AAAGTGTTGAAAAAACCACGTTTTGATTTATCAAGATTGTTGGAATTGCACGGTGACGGA 707

L31G3 AAAGT**T**TTGAAAAAACCACG**A**TTTGA**C**TTATCA**C**G**C**TTGTTGGAATT**A**CACGGTGA**T**GG**C** 707

GW11E11 AAAGT**T**TTGAAAAAACCACG**A**TTTGA**C**TTATCA**C**G**C**TTGTTGGAATT**A**CACGGTGA**T**GG**C** 698

GW14F1 AGCGGTAAATCAGCTGAAGGTGTTACCAATGCCGAAGGTGTCGTTGTTGAACGTCCAGA**G** 780

S33B10 AGCGGTAAATCAGCTGAAGGTGTTACCAATGCCGAAGGTGTCGTTGTTGAACGTCCAGA**G** 461

GW8C6 AGCGGTAAATCAGCTGAAGGTGTTACCAATGCCGAAGGTGTCGTTGTTGAACGTCCAGAA 767

L31G3 AGCGG**C**AAATCAGCTGAAGGTGTTACCAATGCCGAAGG**C**GT**T**GTTGTTGAACG**C**CC**G**GAA 767

GW11E11 AGCGG**C**AAATCAGCTGAAGGTGTTACCAATGCCGAAGG**C**GT**T**GTTGTTGAACG**C**CC**G**GAA 758

GW14F1 GGATACGAACCACCAGTTCAAGAAGCTGTT**TAA**A**C**AATCAACACGCTATTCTTTTTGATC 840

S33B10 GGATACGAACCACCAGTTCAAGAAGCTGTT**TAA**A**C**AATCAACACGCT**T**TTCTTTTTGATC 521

GW8C6 GGATACGAACCACCAGTTCAAGAAGCTGTT**TAA**A**C**AATCAACACGCTATTCTTTTTGATC 827

L31G3 GG**T**TA**T**GAACCACCAGTTCAAGAAGC**C**GTT**TAA**G-AA**CGGT**CA**TTT**TATTC**A**TT**GAA**ATC 826

GW11E11 GG**T**TA**T**GAACCACCAGTTCAAGAAGC**C**GTT**TAA**G-AA**CGGT**CA**TTT**TATTC**A**TT**GAA**ATC 817

GW14F1 GACTTTTTAACACAA**A**CACCTTGAATACAATA**G**TATTTTTTTTTAT**G**GTGAAAAAAAGGC 900

S33B10 GACTTTTTAACACAA**A**CACCTTGAATACAATATTATTTTTTTTTATTGTGAAAAAAAGGC 581

GW8C6 GACTTTTTAACACAA-CACCTTGAATACAATATTATTTTTTTTTATTGTGAAAAAAAGGC 886

L31G3 **A**A**G**--------------------------------------------------------- 829

GW11E11 **A**A**G**--------------------------------------------------------- 820

GW14F1 CCAAAATAGTTTTAAAATAAATGAAAAGCTGTTTATAGCCCAAATG 946

S33B10 CCA**G**AATAGTTTTAAAATAAATGAAAAGCTGTTTATAG-------- 619

GW8C6 CCAAAATAGTTTTAAAATAAATGAAAAGCTGTTTATAGCCCA---- 928

L31G3 ----------------------------------------------

GW11E11 ----------------------------------------------

**Q**

GW2E11 -------------ACCTGTGAATTATATATTAATTAAATTTCACA**ATG**GCTGAAGTTGAA 47

GW4H11 TCTTACAGTGCGGACCTGTGAATTATATATTAATTAAATTTCACA**ATG**GCTGAAGTTGAA 60

L14E7 --------------CCTGTGAATTATATATTAATTAAATTTCACA**ATG**GCTGAAGTTGAA 46

L19C9 -----------------------------------------CACA**ATG**GCTGAAGTTGAA 19

GW2E11 GAACAACAGTTCGATAATTACGAGGATCAACCTCAGGAAAATGTCGAAGAAATGGGCGAA 107

GW4H11 GAACAACAGTTCGATAATTACGAGGATCAACCTCAGGAAAATGTCGAAGAAATGGGCGAA 120

L14E7 GAACAACAGTTCGATAATTACGAGGATCAACCTCAGGAAAATGTCGAAGAAATGGGCGAA 106

L19C9 GAACAACAGTTCGATAATTACGAGGATCAACCTCAGGAAAATGTCGAAGAAATGGGCGAA 79

GW2E11 GAAGTTGAAGCCGATGGTTTGGTTTACGGTACAACCGCTGAAATGCCAGAAATCAAATTG 167

GW4H11 GAAGTTGAAGCCGATGGTTTGGTTTACGGTACAACCGCTGAAATGCCAGAAATCAAATTG 180

L14E7 GAAGTTGAAGCCGATGGTTTGGTTTACGGTACAACCGCTGAAATGCCAGAAATCAAATTG 166

L19C9 GAAGTTGAAGCCGATGGTTTGGTTTACGGTACAACCGCTGAAATGCCAGAAATCAAATTG 139

GW2E11 TTCGGCCGATGGAGCTGCGATGATGTAAATGTCTCAGACATGTCATTAGCTGATTACATT 227

GW4H11 TTCGGCCGATGGAGCTGCGATGATGTAAATGTCTCAGACATGTCATTAGCTGATTACATT 240

L14E7 TTCGGCCGATGGAGCTGCGATGATGTAAATGTCTCAGACATGTCATTAGCTGATTACATT 226

L19C9 TTCGGCCGATGGAGCTGCGATGATGTAAATGTCTCAGACATGTCATTAGCTGATTACATT 199

GW2E11 GCCGTCAAAGAAAAATACGCCCGTTATTTGCCACATTCGGCTGGACGTTATGCTGCTAAA 287

GW4H11 GCCGTCAAAGAAAAATACGCCCGTTATTTGCCACATTCGGCTGGACGTTATGCTGCTAAA 300

L14E7 GCCGTCAAAGAAAAATACGCCCGTTATTTGCCACATTCGGCTGGACGTTATGCTGCTAAA 286

L19C9 GCCGTCAAAGAAAAATACGCCCGTTATTTGCCACATTCGGCTGGACGTTATGCTGCTAAA 259

GW2E11 CGTTTCCGTAAAGCACAATGCCCCATCGTTGAACGATTGACCAATTCATTGATGATGAAA 347

GW4H11 CGTTTCCGTAAAGCACAATGCCCCATCGTTGAACGATTGACCAATTCATTGATGATGAAA 360

L14E7 CGTTTCCGTAAAGCACAATGCCCCATCGTTGAACGATTGACCAATTCATTGATGATGAAA 346

L19C9 CGTTTCCGTAAAGCACAATGCCCCATCGTTGAACGATTGACCAATTCATTGATGATGAAA 319

GW2E11 GGTCGCAACAATGGTAAAAAATTGATGGCTGTTCGTATTGTTAAACATGCGTTCGAAATT 407

GW4H11 GGTCGCAACAATGGTAAAAAATTGATGGCTGTTCGTATTGTTAAACATGCGTTCGAAATT 420

L14E7 GGTCGCAACAATGGTAAAAAATTGATGGCTGTTCGTATTGTTAAACATGCGTTCGAAATT 406

L19C9 GGTCGCAACAATGGTAAAAAATTGATGGCTGTTCGTATTGTTAAACATGCGTT**T**GAAATT 379

GW2E11 ATCTACTTGTTGACCGACAAAAACCCATTACACATTCTGGTAACAGCTATCATCAATTCT 467

GW4H11 ATCTACTTGTTGACCGACAAAAACCCATTACACATTCTGGTAACAGCTATCATCAATTCT 480

L14E7 ATCTACTTGTTGACCGACAA**G**AACCCATTACACATTCTGGTAACAGCTATCATCAATTCT 466

L19C9 ATCTACTTGTTGACCGACAA**G**AACCCATTACACATTCTGGTAACAGCTATCATCAATTCT 439

GW2E11 GGACCACGTGAAGATTCCACACGTATCGGTCGTGCTGGTACTGTTCGTCGTCAAGCTGTT 527

GW4H11 GGACCACGTGAAGATTCCACACGTATCGGTCGTGCTGGTACTGTTCGTCGTCAAGCTGTT 540

L14E7 GGACCACGTGAAGATTCCACACGTATCGGTCGTGCTGGTACTGTTCGTCGTCAAGCTGTT 526

L19C9 GGACCACGTGAAGATTCCACACGTATCGGTCGTGCTGGTACTGTTCGTCGTCAAGCTGTT 499

GW2E11 GATGTTTCACCATTGCGTCGTGTTAATCAAGCAATCTGGCTGCTCTGCACCGGTGCACGT 587

GW4H11 GATGTTTCACCATTGCGTCGTGTTAATCAAGCAATCTGGCTGCTCTGCACCGGTGCACGT 600

L14E7 GATGTTTCACCATTGCGTCGTGTTAATCAAGCAATCTGGCTGCTCTGCACCGGTGCACGT 586

L19C9 GATGTTTCACCATTGCGTCGTGTTAATCAAGCAATCTGGCTGCT**T**TGCACCGGTGCACGT 559

GW2E11 GAAGCTGCATTCCGTAATATTAAATCTATTGCTGAATGTTTGGCCGATGAATTGATCAAC 647

GW4H11 GAAGCTGCATTCCGTAATATTAAATCTATTGCTGAATGTTTGGCCGATGAATTGATCAAC 660

L14E7 GAAGCTGCATTCCGTAATATTAAATCTATTGCTGAATGTTTGGCCGATGAATTGATCAAC 646

L19C9 GAAGCTGCATTCCGTAATATTAAATCTATTGCTGAATGTTTGGCCGATGAATTGATCAAC 619

GW2E11 GCTGCCAAGGGCTCATCCAACTCGTACGCCATCAAAAAGAAGGACGAATTGGAACGTGTC 707

GW4H11 GCTGCCAAGGGCTCATCCAACTCGTACGCCATCAAAAAGAAGGACGAATTGGAACGTGTC 720

L14E7 GCTGCCAAGGGCTCATCCAACTCGTACGCCATCAAAAAGAAGGACGAATTGGAACGTGTC 706

L19C9 GCTGCCAAGGGCTCATCCAACTCGTACGCCATCAAAAAGAAGGACGAATTGGAACGTGTC 679

GW2E11 GCCAAATCGAATCGT**TAA**AGAAAACAACCATTTTTTTTTT-GCTATCTTCACCCTTTATT 766

GW4H11 GCC**C**AATCGAATCGT**TAA**AGAAAACAACCATTTTTTTTTT-GCTATCTTCACCCTTTATT 779

L14E7 GCC**C**AATCGAATCGT**TAA**AGAAAACA**C**CCATTTTTTTTTT**T**GCTATCTTCACCCTTTATT 766

L19C9 GCCAAATCGAATCGT**TAA**AGAAAACAACCATTTTTTTTTT**T**GCTATCTTCACCCTTTATT 738

GW2E11 CGAAACATTACTACAATGTTGCTCCTTGAGAACAGTGTTCAATTGGAATTCTAAGCGAAG 826

GW4H11 CGAAACATTACTACAATGTTGCTCCTTGAGAACAGTGTTCAATTGGAATTCTAAGCGAAG 839

L14E7 CGAAACATTACTACAATGTTGCTCCTTGAGAACAGTGTTCAATTGGAATTCTAAGCGAAG 826

L19C9 CGAAACATTACTACAATGTTGCTCCTTGAGAACAGTGTTCAATTGGAATTCTAAGCGAAG 797

GW2E11 GG-CATAAAT---------- 835

GW4H11 GG-CATAAATTAAATACT-- 856

L14E7 GG**G**CATAAAATAAATACTCC 846

L19C9 GG**G**CATAAAATAAATACTCC 817

**R**

G12G4 TTTCTAGTTCAGCAGCTCATTAAAC**ATG**CAGCAAAAGAAGAAGGAACCTCTACAAGCTGT 60

G34A8 ----TAGTTCAGCAGC**C**CATTAAAC**ATG**CAGCAAAAGAAGAAGGAACCTCTACAAGCTGT 56

G12G4 CCAAGTCTTTGGCAAAAAGAAAACAGCAACCGTTGTTGCCCATTGCAAACGCGGTCGTGG 120

G34A8 CCAAGTCTTTGGCAAAAAGAAAACAGCAACCG**C**TGTTGCCCATTGCAAACGCGGTCGTGG 116

G12G4 ATTGATCCGCATCAATGGACGCCCATTGGATATGATTGAACCAAAAGTTCTCCAATACAA 180

G34A8 ATTGATCCGCATCAATGGACGCCCATTGGATATGATTGAACCAAAAGTTCTCCAATACAA 176

G12G4 ACTCCAAGAACCAATCCTTTTGTTGGGCAAAGAAAAATTCGCCGGTGTTGATATTCGTGT 240

G34A8 ACTCCAAGAACCAATCCTTTTGTTGGGCAAAGAAAAATTCGCCGGTGTTGATATTCGTGT 236

G12G4 TCGAGTGAACGGTGGTGGTCATGTATCCCAAATTTATGCCATCCGTCAAGCCATCTCAAA 300

G34A8 TCGAGTGAACGGTGGTGGTCATGTATCCCAAAT**C**TATGCCATCCGTCAAGCCATCTCAAA 296

G12G4 GGCTTTGGTTTCATACTATCAAAAATATGTTGATGAAGCCTCAAAGAAAGAATTGAAAGA 360

G34A8 GGCTTTGGTTTCATACTATCAAAAATATGTTGATGAAGCCTCAAA**A**AAAGAATTGAAAGA 356

G12G4 CATTTTGATTCAATACGATCGTACATTGTTGGTTGGTGACCCACGTCGTTGCGAACCAAA 420

G34A8 CATTTTGATTCAATACGATCGTACATTGTTGGTTGGTGACCCACGTCGTTGCGAACCAAA 416

G12G4 GAAATTCGGTGGTCCGGGTGCCCGTGCTCGCTACCAAAAATCATACCGT**TAA**GAATTTTC 480

G34A8 GAAATTCGGTGGTCCGGGTGCCCGTGCTCGCTACCAAAAATCATACCGT**TAA**GAATTTTC 476

G12G4 ATTTCTTCTTTCGTACCAAAATTGGAGAAGAAAAACAACAACAACAACCAAACATTATTC 540

G34A8 ATTTCTTCTTTCGTACCAAAATTGGAAAAGAAAAA**T**AACAACAACAACCAAACATTATTC 536

G12G4 ATCTTATTGTTGTGCTTTTGTTTTGGTAAAGAAAAATATAATCTTTTAACGCC 593

G34A8 ATCTTATTGTTGTGCTTTTGTTTTGGTAAAGAAAAATATAATCTTTTAACG-- 587

**S**

GW20C9 --------------------------------CTTTAAAACGTAAAA**ATG**TTTATGCCAA 28

GW11H2 -----------------------------AACCTTTAAAACGTAAAA**ATG**TTTATGCCAA 31

G12F9 ---------CTTTTCGTTCCGGTGTTCGAAACCTTTAAAACGTAAAA**ATG**TTTATGCCAA 51

L15C2 CCTTGGTCTCTTTTCGTTCCGGTGTTCGAAACCTTTAAAACGTAAAA**ATG**TTTATGCCAA 60

G6H4 -------CTCTTTTCGTTCCGGTGTTCGAAACCTTTAAAACGTAAAA**ATG**TTTATGCCAA 53

GW20C9 AGCAACATCGTGTCGCTATCTATGAACATCTTTTCAAAGAAGGTGTCATGGTAGCCAAAA 88

GW11H2 AGCAACATCGTGTCGCTATCTATGAACATCTTTTCAAAGAAGGTGTCATGGTAGCCAAAA 91

G12F9 AGCAACATCGTGTCGCTATCTATGAACATCTTTTCAAAGAAGGTGTCATGGTAGCCAAAA 111

L15C2 AGCAACATCGTGTCGCTATCTATGAACATCTTTTCAAAGAAGGTGTCATGGTAGCCAAAA 120

G6H4 AGCAACATCGTGTCGCTATCTATGAACATCTTTTCAAAGAAGGTGTCATGGTAGCCAAAA 113

GW20C9 AAGATTTCAACTTACCAAAACATCCAGAATTGGATGTCCCAAATTTACATGTCATTAAAA 148

GW11H2 AAGATTTCAACTTACCAAAACA**C**CCAGAATT**A**GATGTCCCAAAT**C**TACATGTCATTAAAA 151

G12F9 AAGATTTCAACTTACCAAAACATCCAGAATTGGATGTCCCAAAT**C**TACATGT**T**ATTAAAA 171

L15C2 AAGATTTCAACTTACCAAAACATCCAGAATTGGATGTCCCAAATTTACATGTCATTAAAA 180

G6H4 AAGATTTCAACTTACCAAAACATCCAGAATTGGATGTCCCAAATTTACATGTCATTAAAA 173

GW20C9 CCATGCAATCTTTGCAATCGAAAGGTTTGGTTCGCTCACAATTCGCATGGAGACATTACT 208

GW11H2 CCATGCAATCTTTGCAATCGAAAGG**C**TTGGTTCGCTCACAATTCGCATGGAGACATTACT 211

G12F9 CCATGCAATCTTTGCAATCGAAAGGTTTGGTTCGCTCACAATTCGCATGGAGACATTACT 231

L15C2 CCATGCAATCTTTGCAATCGAAAGGTTTGGTTCGCTCACAATTCGCATGGAGACATTACT 240

G6H4 CCATGCAATCTTTGCAATCGAAAGGTTTGGTTCGCTCACAATTCGCATGGAGACATTACT 233

GW20C9 ACTGGTATTTGGAAAATGAAGGCATCGAATACCTTCGCACATATTTGCATTTGCCATCAG 268

GW11H2 ACTGGTATTTGGAAAATGAAGGCATCGAATACCTTCGCACATATTTGCATTTGCCATCAG 271

G12F9 ACTGGTATTTGGAAAATGAAGGCATCGAATACCTTCGCACATATTTGCATTTGCCATCAG 291

L15C2 ACTGGTATTTGGAAAATGAAGGCATCGAATACCTTCGCACATATTTGCATTTGCCATCAG 300

G6H4 ACTGGTATTTGGAAAATGAAGGCATCGAATACCTTCGCACATATTTGCATTTGCCATCAG 293

GW20C9 AAATCGTTCCGTCAACATTGAAACGTGCTGCCCGTACAGAACCAGCTCGTGCTCGTCCAA 328

GW11H2 AAATCGTTCCGTCAACATTGAAACGTGCTGCCCGTACAGAACCAGCTCGTGCTCGTCCAA 331

G12F9 AAATCGTTCCGTCAACATTGAAACGTGCTGCCCGTACAGAACCAGCTCGTGCTCGTCCAA 351

L15C2 AAATCGTTCCGTCAACATTGAAACGTGCTGCCCGTACAGAACCAGCTCGTGCTCGTCCAA 360

G6H4 AAATCGTTCCGTCAACATTGAAACGTGCTGCCCGTACAGAACCAGCTCGTGCTCGTCCAA 353

GW20C9 GTGCTGTTCCGCGATCAGGTGATGCTAGCAAAACTGGTGCTGACCGTTCAGCTTATCGTC 388

GW11H2 GTGCTGTTCCGCGATCAGGTGATGCTAGCAAAACTGGTGCTGACCGTTCAGCTTATCGTC 391

G12F9 GTGCTGTTCCGCGATCAGGTGATGCTAGCAAAACTGGTGCTGACCGTTCAGCTTATCGTC 411

L15C2 GTGCTGTTCCGCGATCAGGTGATGCTAGCAAAACTGGTGCTGACCGTTCAGCTTATCGTC 420

G6H4 GTGCTGTTCCGCGATCAGGTGATGCTAGCAAAACTGGT**T**CTGACCGTTCAGCTTATCGTC 413

GW20C9 GTGCTCCGGCTGATGGTGTTGACAAAAAGGCCGATGTTGGCGCTGGTGCCGGAGATGTTG 448

GW11H2 GTGCTCCGGCTGATGGTGTTGACAAAAAGGCCGATGTTGGCGCTGGTGCCGGAGATGTTG 451

G12F9 GTGCTCCGGCTGATGGTGTTGACAAAAAGGCCGATGTTGGCGCTGGTGCCGGAGATGTTG 471

L15C2 GTGCTCCGGCTGATGGTGTTGACAAAAAGGCCGATGTTGGCGCTGGTGCCGGAGATGTTG 480

G6H4 GTGCTCCGGCTGATGGTGTTGACAAAAAGGCCGATGTTGGCGCTGGTGCCGGAGATGTTG 473

GW20C9 AATTCCGTGGTGGATTCGGACGTGGTTCACGGCCACAA**TAA**TAATGTTTTTATCACAACA 508

GW11H2 AATTCCGTGGTGGATTCGGACGTGGTTCACGGCCACAA**TAA**TAATGTTTTTATCACAACA 511

G12F9 AATTCCGTGGTGGATTCGGACGTGGTTCACGGCCACAA**TAA**TAATGTTTTTATCACAACA 531

L15C2 AATTCCGTGGTGGATTCGGACGTGGTTCACGGCCACAA**TAA**TAATGTTTTTATCACAACA 540

G6H4 AATTCCGTGGTGGATTCGGACGTGGTTCACGGCCACAA**TAA**TAATGTTTTTATCACAACA 533

GW20C9 TTGTGAATTAAATGTCGAAATAAAGCAACATTTCTAACAAACATTCAAGAAC 560

GW11H2 TTGTGAATTAAATGTCGAAATAAAGCAACATTTCTAACAAACATTCAAGAAC 563

G12F9 TTGTGAATTAAATGTCGAAATAAA**A**CAACATTTCTAACAAACATTCAAGAGC 583

L15C2 TTGTGAATTAAATGTCGAAATAAAGCAACATTTCTAACAAACAT-------- 584

G6H4 TTGTGAATTAAATGTCGAAATAAAGCAACATTTCTAACAAACAT-------- 577

**T**

G48A9 ---------------------------------------TTTTACATTTTGGTTGCGGCA 21

GW19B10 -----------------------------------------------------------A 1

L14G10 ---------------------------------------TTTTACATTTTGGTTGCGGCA 21

GW6C6 ------------------------------------------------------------

GW4A11 --------------------AAACGACATCCGGCATTTCTTTTACATTTTGGTTGCGGCA 40

L17C3 ---------------------------------------TTTTACATTTTGGTTGCGGCA 21

G8G11 TATTCACTTTGGCAGTCCCGAAACGACATCCGGCATTTCTTTTACATTTTGGTTGCGGCA 60

GW1F9 -----------------------------------------------------------A 1

G7C11 ---------------------------------------TTTTACATTTTGGTTGCGGCA 21

G48A9 ATAGAAACAAGCAAAA**ATG**TCGTTGGTTATCCCAGAAAAATTCCAGCATATCTTGCGTAT 81

GW19B10 ATAGAAACAAGCAAAA**ATG**TCGTTGGTTATCCCAGAAAAATTCCAGCATATCTTGCGTAT 61

L14G10 ATAGAAACAAGCAAAA**ATG**TCGTTGGTTATCCCAGAAAAATTCCAGCATATCTTGCGTAT 81

GW6C6 ---------------------------------------------------CTTGCGTAT 9

GW4A11 ATAGAAACAAGCAAAA**ATG**TCGTTGGTTATCCCAGAAAAATTCCAGCATATCTTGCGTAT 100

L17C3 ATAGAAACAAGCAAAA**ATG**TCGTTGGTTATCCCAGAAAAATTCCAGCATATCTTGCGTAT 81

G8G11 ATAGAAACAAGCAAAA**ATG**TCGTTGGTTATCCCAGAAAAATTCCAGCATATCTTGCGTAT 120

GW1F9 ATAGAAACAAGCAAAA**ATG**TCGTTGGTTATCCCAGAAAAATTCCAGCATATCTTGCGTAT 61

G7C11 ATAGAAACAAGCAAAA**ATG**TCGTTGGTTATCCCAGAAAAATTCCAGCATATCTTGCGTAT 81

G48A9 TATGAATACAAACATCGATGGTAATCGTAAAGTTACGATTGCAATGACAGCCATCAAGGG 141

GW19B10 TATGAATACAAACATCGATGGTAATCGTAAAGTTACGATTGCAATGACAGCCATCAAGGG 121

L14G10 TATGAATACAAACATCGATGGTAATCGTAAAGTTACGATTGCAATGACAGCCATCAAGGG 141

GW6C6 TATGAATACAAACATCGATGGTAATCGTAAAGTTACGATTGCAATGACAGCCATCAAGGG 69

GW4A11 TATGAATACAAACATCGATGGTAATCGTAAAGTTACGATTGCAATGACAGCCATCAAGGG 160

L17C3 TATGAATACAAACATCGATGGTAATCGTAAAGTTACGATTGCAATGACAGCCATCAAGGG 141

G8G11 TATGAATACAAACATCGATGGTAATCGTAAAGTTACGATTGCAATGACAGCCATCAAGGG 180

GW1F9 TATGAATACAAACATCGATGGTAATCGTAAAGTTACGATTGCAATGACAGCCATCAAGGG 121

G7C11 TATGAATACAAACATCGATGGTAATCGTAAAGTTACGATTGCAATGACAGCCATCAAGGG 141

G48A9 AGTTGGTCGTCGTTATGCCAACATCGTTTTGAAAAAGGCCGATGTTGATTTGACCAAACG 201

GW19B10 AGTTGGTCGTCGTTATGCCAACATCGTTTTGAAAAAGGCCGATGTTGATTTGACCAAACG 181

L14G10 AGTTGGTCGTCGTTATGCCAACATCGTTTTGAAAAAGGCCGATGTTGATTTGACCAAACG 201

GW6C6 AGTTGGTCGTCGTTATGCCAACATCGTTTTGAAAAAGGCCGATGTTGATTTGACCAAACG 129

GW4A11 AGTTGGTCGTCGTTATGCCAACATCGTTTTGAAAAAGGCCGATGTTGATTTGACCAAACG 220

L17C3 AGTTGGTCGTCGTTATGCCAACATCGTTTTGAAAAAGGCCGATGTTGATTTGACCAAACG 201

G8G11 AGTTGGTCGTCGTTATGCCAACATCGTTTTGAAAAAGGCCGATGTTGATTTGACCAAACG 240

GW1F9 AGTTGGTCGTCGTTATGCCAACATCGTTTTGAAAAAGGCCGATGTTGATTTGACCAAACG 181

G7C11 AGTTGGTCGTCGTTATGCCAACATCGTTTTGAAAAAGGCCGATGTTGATTTGACCAAACG 201

G48A9 TGCTGGTGAATGCACCGAAGAGGAAGTTGACAAAATTGTCACCATTATCTCAAATCCACG 261

GW19B10 TGCTGGTGAATGCACCGAAGAGGAAGTTGACAAAATTGTCACCATTATCTCAAATCCACG 241

L14G10 TGCTGGTGAATGCACCGAAGAGGAAGTTGACAAAATTGTCACCATTATCTCAAATCCACG 261

GW6C6 TGCTGGTGAATGCACCGAAGAGGAAGTTGACAAAATTGTCACCATTATCTCAAATCCACG 189

GW4A11 TGCTGGTGAATGCACCGAAGAGGAAGTTGACAAAATTGTCACCATTATCTCAAATCCACG 280

L17C3 TGCTGGTGAATGCACCGAAGAGGAAGTTGACAAAATTGTCACCATTATCTCAAATCCACG 261

G8G11 TGCTGGTGAATGCACCGAAGAGGAAGTTGACAAAATTGTCACCATTATCTCAAATCCACG 300

GW1F9 TGCTGGTGAATGCACCGAAGAGGAAGTTGACAAAATTGTCACCATTATCTCAAATCCACG 241

G7C11 TGCTGGTGAATGCACCGAAGAGGAAGTTGACAAAATTGTCACCATTATCTCAAATCCACG 261

G48A9 TCAATACAAAATCCCAAATTGGTTTTTGAACAGACAAAAGGATATCATTGATGGTAAATA 321

GW19B10 TCAATACAAAATCCCAAATTGGTTTTTGAACAGACAAAAGGATATCATTGATGGTAAATA 301

L14G10 TCAATACAAAATCCCAAATTGGTTTTTGAACAGACAAAAGGATATCATTGATGGTAAAT**G** 321

GW6C6 TCAATACAAAATCCCAAATTGGTTTTTGAACAGACAAAAGGATATCATTGATGGTAAATA 249

GW4A11 TCAATACAAAATCCCAAATTGGTTTTTGAACAGACAAAA**A**GATATCATTGATGGTAAATA 340

L17C3 TCAATACAAAATCCCAAATTGGTTTTTGAACAGACAAAAGGATATCATTGATGGTAAATA 321

G8G11 TCAATACAA**G**ATCCCAAATTGGTTTTTGAACAGACAAAAGGATATCATTGATGGTAAATA 360

GW1F9 TCAATACAAAATCCCAAATTGGTTTTTGAACAGACAAAAGGATATCATTGATGGTAAATA 301

G7C11 TCAATACAAAATCCCAAATTGGTTTTTGAACAGACAAAAGGATATCATTGATGGTAAATA 321

G48A9 CGCA**T**AATTGACCTCATCCAATTTGGACTCAAAATT**A**CGTGAAGATTTGGAACGTTTGAA 381

GW19B10 CGCACAATTGACCTCATCCAATTTGGACTCAAAATT**A**CGTGAAGATTTGGAACGTTTGAA 361

L14G10 CGCACAATTGACCTCATCCAATTTGGACTCAAAATTGCGTGAAGATTTGGAACGTTTGAA 381

GW6C6 CGCACAATTGACCTCATCCAATTTGGACTCAAAATTGCGTGAAGATTTGGAACGTTTGAA 309

GW4A11 CGCACAATTGACCTCATCCAATTTGGACTCAAAATTGCGTGAAGATTTGGAACGTTTGAA 400

L17C3 CGCACAATTGACCTCATCCAATTTGGACTCAAAATTGCGTGAAGATTTGGAACGTTTGAA 381

G8G11 CGCACAATTGACCTCATCCAATTTGGACTCAAAATTGCGTGAAGATTTGGAACGTTTGAA 420

GW1F9 CGCACAATTGACCTCATCCAATTTGGACTCAAAATTGCGTGAAGATTTGGAACGTTTGAA 361

G7C11 CGCACAATTGACCTCATCCAATTTGGACTCAAAATTGCGTGAAGATTTGGAACGTTTGAA 381

G48A9 GAAAATCCGTGCCCATCGTGGTATGCGTCACTACTGGGGTCTCCGTGTCCGTGGTCAACA 441

GW19B10 GAAAATCCGTGCCCATCGTGGTATGCGTCACTACTGGGGTCTCCGTGTCCGTGGTCAACA 421

L14G10 GAAAATCCGTGCCCATCGTGGTATGCGTCACTACTGGGGTCTCCGTGTCCGTGGTCAACA 441

GW6C6 GAAAATCCGTGCCCATCGTGGTATGCGTCACTACTGGGGTCTCCGTGTCCGTGGTCAACA 369

GW4A11 GAAAATCCGTGCCCATCGTGGTATGCGTCACTACTGGGGTCTCCGTGTCCGTGGTCAACA 460

L17C3 GAAAATCCGTGCCCATCGTGGTATGCGTCACTACTGGGGTCTCCGTGTCCGTGGTCAACA 441

G8G11 GAAAATCCGTGCCCATCGTGGTATGCGTCACTACTGGGGTCTCCGTGTCCGTGGTCAACA 480

GW1F9 GAAAATCCGTGCCCATCGTGGTATGCGTCACTACTGGGGTCTCCGTGTCCGTGGTCAACA 421

G7C11 GAAAATCCGTGCCCATCGTGGTATGCGTCACTACTGGGGTCTCCGTGTCCGTGGTCAACA 441

G48A9 CACAAAAACAACCGGTCGTCGTGGACGTACCGTTGGTGTATCGAAAAAGAAG**TAA**A**C**TAT 501

GW19B10 CACAAAAACAACCGGTCGTCGTGGACGTACCGTTGGTGTATCGAAAAAGAAG**TAA**A**C**TAT 481

L14G10 CACAAAAACAACCGGTCGTCGTGGACGTACCGTTGGTGTATCGAAAAAGAAG**TAA**ATTAT 501

GW6C6 CACAAAAACAACCGGTCGTCGTGGACGTACCGTTGGTGTATCGAAAAAGAAG**TAA**ATTAT 429

GW4A11 CACAAAAACAACCGGTCGTCGTGGACGTACCGTTGGTGTATCGAAAAAGAAG**TAA**ATTAT 520

L17C3 CACAAAAACAACCGGTCGTCGTGGACGTACCGTTGGTGTATCGAAAAAGAAG**TAA**ATTAT 501

G8G11 CACAAAAACAACCGGTCGTCGTGGACGTACCGTTGGTGTATCGAAAAAGAAG**TAA**ATTAT 540

GW1F9 CACAAAAACAACCGGTCGTCGTGGACGTACCGTTGGTGTATCGAAAAAGAAG**TAA**ATTAT 481

G7C11 CACAAAAACAACCGGTCGTCGTGGACGTACCGTTGGTGTATCGAAAAAGAAG**TAA**ATTAT 501

G48A9 ATTACAATTTATTCATACTTTATGTTCTTTTTTTTCGACGTGTTTTTACCCAA**C**AACACA 561

GW19B10 ATTACAATTTATTCATACTTTATGTTCTTTTTTTTCGACGTGTTTTTACCCAA**C**AACACA 541

L14G10 ATTACAATTTATTCATACTTTATGTTCTTTTTTTTCGACGTGTTTTTACCCAATAACACA 561

GW6C6 ATTACAATTTATTCATACTTTATGTTCTTTTTTTTCGACGTGTTTTTACCCAATAACACA 489

GW4A11 ATTACAATTTATTCATACTTTATGTTCTTTTTTTTCGACGTGTTTTTACCCAATAACACA 580

L17C3 ATTACAATTTATTCATACTTTATGTTCTTTTTTTTCGACGTGTTTTTACCCAATAACACA 561

G8G11 ATTACAATTTATTCATACTTTATGTTCTTTTTTTTCGACGTGTTTTTACCCAATAACACA 600

GW1F9 ATTACAATTTATTCATACTTTATGTTCTTTTTTTTCGACGTGTTTTTACCCAATAACACA 541

G7C11 ATTACAATTTATTCATACTTTATGTTCTTTTTTTTCGACGTGTTTTTACCCAATAAC**G**CA 561

G48A9 AACATTTTACCATCTTTC**A**AACACACCAAAAAAAACGAAAATCAAACTATTTGGTGGC**C**A 621

GW19B10 AACATTTTACCATCTTTC**A**AACACACCAAAAAAAACGAAAATCAAACTATTTGGTGGC**C**A 601

L14G10 AACATTTTACCATCTTTCGAACACACCAAAAAAAACGAAAATCAAACTATTTGGTGGCTA 621

GW6C6 AACATTTTACCATCTTTCGAACACACCAAAAAAAACGAAAATCAAACTATTTGGTGGCTA 549

GW4A11 AACATTTTACCATCTTTCGAACACACCAAAAAAAACGAAAATCAAACTATTTGGTGGCTA 640

L17C3 AACATTTTACCATCTTTCGAACACACCAAAAAAAACGAAAATCAAACTATTTGGTGGCTA 621

G8G11 AACATTTTACCATCTTTCGAACACACCAAAAAAAACGAAAATCAAACTATTTGGTGGCTA 660

GW1F9 AACATTTTACCATCTTTCGAACAC------------------------------------ 565

G7C11 AACATTTTACCATCTTTCGAACACACCAAAAAAAACGAAAATCAAACTATTTGGTGGCTA 621

G48A9 CTTTAACAATGTTTAAAATTGTTTTTACTTATTTCTATGGA**A**TGTGAAAATCATTTGGAT 681

GW19B10 CTTTAACAATGTTTAAAATTGTTTTTACTTATTTCTATGGA**A**TGTGAAAATCATTTGGAT 661

L14G10 CTTTAACAATGTTTAAAATTGTTTTTACTTATTTCTATGGATTGTGAAAATCATTTGGAT 681

GW6C6 CTTTAACAATGTTTAAAATTGTTTTTACTTATTTCTATGGATTGTGAAAATCATTTGGAT 609

GW4A11 CTTTAACAATGTTTAAAATTGTTTTTACTTATTTCTATGGATTGTGAAAATCATTTGGAT 700

L17C3 CTTTAACAATGTTTAAAATTGTTTTTACTTATTTCTATGGATTGTGAAAATCATTTGGAT 681

G8G11 CTTTAACAATGTTTAAAATTGTTTTTACTTATTTCTATGGATTGTGAAAATCATTTGGAT 720

GW1F9 ------------------------------------------------------------

G7C11 CTTTAACAATGTTTAAAATTGTTTTTACTTATTTCTATGGATTGTGAAAATCATTTGGAT 681

G48A9 GTCATTTTTGTGGGGATAAAGTAAAGATAAC--- 712

GW19B10 GTCATTTTTGTGGGGATAAAGTAAAGATAACATT 695

L14G10 GTCATTTTTGTGGGGATAAAGTAAAGATAACATT 715

GW6C6 GTCATTTTTGTGGGGATAAAGTAAAGATAACATT 643

GW4A11 GTCATTTTTGTGGGGATAAAGTAAAGATAACATT 734

L17C3 GTCATTTTTGTGGGGATAAAGTAAAGATAAC--- 714

G8G11 GTCATTTTTGTGGGGATAAAGTAAAGAT------ 748

GW1F9 ----------------------------------

G7C11 GTCATTTTTGTGGGGATAAAGTAAAGAT------ 709

**U**

S16G2 --------------------GGTCTTGAACGCTGTATACTTGC**A**ATCAGCTAGATACAAA 40

GW13A6 -------------------------------CTGTATACTTGCGATCAGCTAGATACAAA 29

GW9C3 --------------------------------------CTTGCGATCAGCTAGATACAAA 22

L16C4 -----CTT**T**TTTTCATATGCGGTCTTGAACGCTGTATACTTGCGATCAGCTAGATACAAA 55

G9B4 -----CTTCTTTTCATATGCGGTCTTGAACGCTGTATACTTGCGATCAGCTAGATACAAA 55

G5H12 -----**T**TTCTTTTCATATGCGGTCTTGAACGCTGTATACTTGCGATCAGCTAGA**A**ACAAA 55

G21H7 ACTTCCTTCTTTTCATATGCGGTCTTGAACGCTGTATACTTGCGATCAGCTAGATACAAA 60

S16G2 AATAAATCGAAGACACA**ATG**GTTCGTATGAATGTTT**C**GGCTGATGCACTTAAATGCATAA 100

GW13A6 AATAAATCGAAGACACA**ATG**GTTCGTATGAATGTTTTGGCTGATGCACTTAAATGCATAA 89

GW9C3 AATAAATCGAAGACACA**ATG**GTTCGTATGAATGTTTTGGCTGATGCACTTAAATGCATAA 82

L16C4 AATAAATCGAAGACACA**ATG**GTTCGTATGAATGTTTTGGCTGATGCACTTAAATGCATAA 115

G9B4 AATAAATCGAAGACACA**ATG**GTTCGTATGAATGTTTTGGCTGATGCACTTAAATGCATAA 115

G5H12 AATAAATCGAAGACACA**ATG**GTTCGTATGAATGTTTTGGCTGATGCACTTAAATGCATAA 115

G21H7 AATAAATCGAAGACACA**ATG**GTTCGTATGAATGTTTTGGCTGATGCACTTAAATGCATAA 120

S16G2 ACAATGCTGAAAAACGTGGCAAACGTCAAGTTTTGTTGCGTCCATGCTCAAAAGTCATCA 160

GW13A6 ACAATGCTGAAAAACGTGGCAAACGTCAAGTTTTGTTGCGTCCATGCTCAAAAGTCATCA 149

GW9C3 ACAATGCTGAAAAACGTGGCAAACGTCAAGTTTTGTTGCGTCCATGCTCAAAAGTCATCA 142

L16C4 ACAATGCTGAAAAACGTGGCAAACGTCAAGTTTTGTTGCGTCCATGCTCAAAAGTCATCA 175

G9B4 ACAATGCTGAAAAACGTGGCAAACGTCAAGTTTTGTTGCGTCCATGCTCAAAAGTCATTA 175

G5H12 ACAATGCTGAAAAACGTGGCAAACGTCAAGTTTTGTTG**T**GTCCATGCTCAAAAGTCATCA 175

G21H7 ACAATGCTGAAAAACGTGGCAAACGTCAAGTTTTGTTGCGTCCATGCTCAAAAGTCATCA 180

S16G2 TTAAATTTTTAACTGTTATGATGAAACATGGTTACATTGGTGAATTCGAAATTGTTGATG 220

GW13A6 TTAAATTTTTGACTGTTATGATGAAACATGGTTACATTGGTGAATTCGAAATTGTTGATG 209

GW9C3 TTAAATTTTTAACTGTTATGATGAAACATGGTTACATTGGTGAATTCGAAATTGTTGATG 202

L16C4 TTAAATTTTTAACTGTTATGATGAAACATGGTTACATTGGTGAATTCGAAATTGTTGATG 235

G9B4 TTAAATTTTTAACTGTTATGATGAAACATGGTTACATTGGTGAATTCGAAATTGTTGATG 235

G5H12 TTAAATTTTTAACTGTTATGATGAAACATGGTTACATTGGTGAATTCGAAATTGTTGATG 235

G21H7 TTAAATTTTT**G**ACTGTTATGATGAAACATGGTTACATTGGTGAATTCGAAATTGTTGATG 240

S16G2 ACCATCGTTCAGGCAAAGTTGTTGTCAACTTAACTGGTCGTCTCAACAAATGCGGTGTCA 280

GW13A6 ACCATCGTTCAGGCAAAGTTGTTGTCAACTTAACTGGTCGTCTCAACAAATGCGGTGTCA 269

GW9C3 ACCATCGTTCAGGCAAAGTTGTTGTCAACTTAACTGGTCGTCTCAACAAATGCGGTGTCA 262

L16C4 ACCATCGTTCAGGCAAAGTTGTTGTCAACTTAACTGGTCGTCTCAACAAATGCGGTGTCA 295

G9B4 ACCATCGTTCAGGCAAAGTTGTTGTCAACTTAACTGGTCGTCTCAACAAATGCGGTGTCA 295

G5H12 ACCATCGTTCAGGCAAAGTTGTTGTCAACTTAACTGGTCGTCTCAACAAATGCGGTGTCA 295

G21H7 ACCATCGTTCAGGCAAAGTTGTTGTCAACTTAACTGGTCGTCTCAACAAATGCGGTGTCA 300

S16G2 TCTCACCACGATTTGA**T**GTTCCAATCAATGACATCGAAAAATGGACAAACAATTTATTGC 340

GW13A6 TCTCACCACGATTTGA**T**GTTCCAATCAATGACATCGAAAAATGGACAAACAATTTATTGC 329

GW9C3 TCTCACCACGATTTGACGTTCCAATCAATGACATCGAAAAATGGACAAACAATTTATTGC 322

L16C4 TCTCACCACGATTTGACGTTCCAATCAATGACATCGAAAAATGGACAAACAATTTATTGC 355

G9B4 TCTCACCACGATTTGACGTTCCAATCAATGACATCGAAAAATGGACAAACAATTTATTGC 355

G5H12 TCTCACCACGATTTGA**T**GTTCCAATCAATGACATCGAAAAATGGACAAACAATTTATTGC 355

G21H7 TCTCACCACGATTTGACGTTCCAATCAATGACATCGAAAAATGGACAAACAATTTATTGC 360

S16G2 CATCTCGTCAATTTGGTTATGTTGTTTTAACCACCAGCGGCGGAATCATGGACCATGAAG 400

GW13A6 CATCTCGTCAATTTGGTTATGTTGTTTTAACCACCAGCGGCGGAATCATGGACCATGAAG 389

GW9C3 CATCTCGTCAATTTGGTTATGTTGTTTTAACCACCAGCGGCGGAATCATGGACCATGAAG 382

L16C4 CATCTCGTCAATTTGGTTATGTTGTTTTAACCACCAGCGGCGGAATCATGGACCATGAAG 415

G9B4 CATCTCGTCAATTTGGTTATGTTGTTTTAACCACCAGCGGCGGAATCATGGACCATGAAG 415

G5H12 CATCTCGTCAATTTGGTTATGTTGTTTT**G**ACCACCAGCGGCGGAATCATGGACCATGAAG 415

G21H7 CATCTCGTCAATTTGGTTATGTTGTTTTAACCACCAGCGGCGGAATCATGGACCATGAAG 420

S16G2 AAGCCCGGCGAAAACATCTCGGAGGCAAAATTCTCGGTTTCTTCTTC**TAA**ACCGAATGCA 460

GW13A6 AAGCCCGGCGAAAACATCTCGGAGGCAAAATTCTCGGTTTCTTCTTC**TAA**ACCGAATGCA 449

GW9C3 AAGCCCGGCGAAAACATCTCGGAGGCAAAATTCTCGGTTTCTTCTTC**TAA**ACCGAATGCA 442

L16C4 AAGCCCGGCGAAAACATCTCGGAGGCAAAATTCTCGGTTTCTTCTTC**TAA**ACCGAA**C**GCA 475

G9B4 AAGCCCGGCGAAAACATCTCGGAGGCAAAATTCTCGGTTTCTTCTTC**TAA**ACCGAA**C**GCA 475

G5H12 AAGCCCG**A**CGAAAACATCTCGGAGGCAAAATTCTCGGTTTCTTCTTC**TAA**ACCGAATGCA 475

G21H7 AAGCCCGGCGAAAACATCTCGGAGGCAAAATTCTCGGTTTCTTCTTC**TAA**ACCGAA**C**GCA 480

S16G2 ACTGAATCAACGCTAAGGAAGAACTATATATATTTCTTTTTTCAATGCGAAAAAAATCAA 520

GW13A6 ACTGAATCAACGCTAAGGAAGAACT**C**TATATATTTCTTTTTTCAATGCGAAAAAAATCAA 509

GW9C3 ACTGAATCAACGCTAAGGAAGAACTATATATATTTCTTTTTTCAATGCGAAAAAAATCAA 502

L16C4 ACTGAATCAACGCTAAGGAAGAACTATATATATTTCTTTTTTCAATGCGA**G**AAAAATCAA 535

G9B4 ACTGAATCAACGCTAAGGAAGAACTATATATATTTCTTTTTTCAATGCGA**G**--------- 526

G5H12 ACTGAATCAA**T**GCTAAGGAAGAACTATATATATTTCTTTTTTCAATGCG----------- 524

G21H7 ACTGAATCAACGCTAAGGAAGAACTATATATATTTCTTTTTTCAATGCGA**G**AAAAATCAA 540

S16G2 TAAAACTTTC 530

GW13A6 TAAAACTTTC 519

GW9C3 TAAAACTTTC 512

L16C4 TAAAACTTTC 545

G9B4 ----------

G5H12 ----------

G21H7 T--------- 541

**V**

L18C2 GTCAAATCAATAAG**ATG**CCAGGCGTTATCGTAAAAGACGTTGATCAAGCTGCGCTTGTAA 60

GW8C2 GTCAAATCAATAAG**ATG**CCAGGCGTTATCGTAAAAGACGTTGATCAAGCTGCGCTTGTAA 60

GW5D10 ---AAATCAATAAG**ATG**CCAGGCGTTATCGTAAAAGACGTTGATCAAGCTGCGCTTGTAA 57

L18C2 AAGCCACAGCCGAATTCTTGAAAAAATCCGGTAAACTAAAGGTTCCAGAACAAATGGAAA 120

GW8C2 AAGCCACAGCCGAATTCTTGAAAAAATC**G**GGTAAACTAAAGGTTCCAGAACAAATGGAAA 120

GW5D10 AAGCCACAGCCGAATTCTTGAAAAAATCCGGTAAACTAAAGGTTCCAGAACAAATGGAAA 117

L18C2 TCGTTAAGACCGCTAAATTCAAGGAATTGTCACCAACCGATCCGGATTGGTTCTATACAC 180

GW8C2 TCGTTAAGACCGCTAAATTCAAGGAATTGTCACCAACCGATCCGGATTGGTTCTATACAC 180

GW5D10 TCGTTAAGACCGCTAAATTCAAGGAATTGTCACCAACCGATCCGGATTGGTTCTATACAC 177

L18C2 GTTGTGCATCAATTTTGCGTCACACATACCA**T**CGTCGTTCAGTCGGTGTTGGTGCCATCA 240

GW8C2 GTTGTGCATCAATTTTGCGTCACACATACCACCGTCGTTCAGTCGGTGTTGGTGCCATCA 240

GW5D10 GTTGTGCATCAATTTTGCGTCACACATACCACCGTCGTTCAGTCGGTGTTGGTGCCATCA 237

L18C2 CCAAAATTTATGGTGGCCGTAAACGTAATGGAGTTCATCCATCACATTTCTGTCGTTCAG 300

GW8C2 CCAAAATTTATGGTGGCCGTAAACGTAATGGAGTTCATCCATCACATTTCTGTCGTTCAG 300

GW5D10 CCAAAATTTATGGTGGCCGTAAACGTAATGGAGTTCATCCATCACATTTCTGTCGTTCAG 297

L18C2 CTGACGGTGTTGCACGTAAAGCGTTACAAGCTTT**A**GAAGCTGTTAAATTGATCGAAAAAC 360

GW8C2 CTGACGGTGTTGCACGTAAAGCGTTACAAGCTTTGGAAGCTGTTAAATTGATCGAAAAAC 360

GW5D10 CTGACGGTGTTGCACGTAAAGCGTTACAAGCTTTGGAAGCTGTTAAATTGATCGAAAAAC 357

L18C2 ATACCGACGGTGGCCGCTCATTGACATCTCAAGGTCGCCGACATCTCGATCATATTGCTA 420

GW8C2 ATACCGACGGTGGCCGCTCATTGACATCTCAAGGTCGCCGACATCTCGATCATATTGCTA 420

GW5D10 ATACCGACGGTGGCCGCTCATTGACATCTCAAGGTCGCCGACATCTCGATCATATTGCTA 417

L18C2 ACCAAATTGTTACAAAACAAAGAGCTGCCATCAAGGCTGCCGCCAGCGCTGTTATCATTA 480

GW8C2 ACCAAATTGTTACAAAACAAAGAGCTGCC**T**TCAAGGCTGCCGCCAGCGCTGTTATCATTA 480

GW5D10 ACCAAATTGTTACAAAACAAAGAGCTGCCATCAAGGCTGCCGCCAGCGCTGTTATCATTA 477

L18C2 CAGAA**TAA**ACAAACAAAAAGTTTTTTTTGAATTG**AT**TCATTTACGATCGAAATGATACAA 540

GW8C2 CAGAA**TAA**ACAAACAAAAAGTTTTTTTTGAATTG--TCATTTACGATCGAAATGATACAA 538

GW5D10 CAGAA**TAA**ACAAACAAAAAGTTTTTTTTGAATTG--TCATTTACG**G**TCGAAATGATACAA 535

L18C2 TAACGATGCATTATTTCAAATAAAATCAGAATCCAATCCATG 582

GW8C2 TAACGATGCATTATTTCAAATAAAATCAGAATCCAATCCAT- 579

GW5D10 TAACGATGCATTATTTCAAATAAAATCAGAA**C**CCAATCCAT- 576

**W**

G10G4 ----CGGTATTGTCGACTCTTCTAAAGACTCCGCCAAAAAATCTTATTAAAA**ATG**GAAAA 56

GW3A12 ATTACGGTATTGTCGACTCTTCTAAAGACTCCGCCAAAAAATCT**A**ATTAAAA**ATG**GAAAA 60

G12D4 -TTACGGTATTGTCGACTCTTCTAAAGACTCCGCCAAAAAATCTTATTAAAA**ATG**GAAAA 59

G18d2 ATTACGGTATTGTCGACTCTTCTAAAGACTCCGCCAAAAAATCTTATTAAAA**ATG**GAAAA 60

L11C15 -TTACGGTATTGTCGACTCTTCTAAAGACTCCGCCAAAAAATCTTATTAAAA**ATG**GAAAA 59

G10G4 CGACGCTGGTGAA**G**TCGTTGATTTGTACTGCCCACGAAAATGTTCGGCTAGCAACCGTAT 116

GW3A12 CGACGCTGGTGAATTCGTTGATTTGTACTGCCCACGAAAATGTTCGGCTAGCAACCGTAT 120

G12D4 CGACGCTGGTGAATTCGTTGATTTGTACTGCCCACGAAAATGTTCGGCTAGCAACCGTAT 119

G18d2 CGACGCTGGTGAATTCGTTGATTTGTACTGCCCACGAAAATGTTCGGCTAGCAACCGTAT 120

L11C15 CGACGCTGGTGAATTCGTTGATTTGTACTGCCCACGAAAATGTTCGGCTAGCAACCGTAT 119

G10G4 CATCCATGCTAAAGATCATGCTTCGGTTCAACTTAATCTTGCCGAAGTTGATCCAAACAC 176

GW3A12 CATCCATGCTAAAGATCATGCTTC**A**GTTCAACTTAATCTTGCCGAAGTTGATCCAAACAC 180

G12D4 CATCCATGCTAAAGATCATGCTTCGGTTCAACTTAATCTTGCCGAAGTTGATCCAAACAC 179

G18d2 CATCCATGCTAA**G**GATCATGCTTCGGTTCAACTTAATCTTGCCGAAGTTGATCCAAACAC 180

L11C15 CATCCATGCTAAAGATCATGCTTCGGTTCAACTTAATCTTGCCGAAGTTGATCCAAACAC 179

G10G4 TGGAATCATGACCGGTACATCAAAAGTTTATGCCATCTGTGGAGCTATCCGTGGTATGGG 236

GW3A12 TGGAATCATGACCGGTACATCAAAAGTTTATGCCATCTGTGGAGCTATCCGTGGTATGGG 240

G12D4 TGGAATCATGACCGGTACATCAAAAGTTTATGCCATCTGTGGAGCTATCCGTGGTATGGG 239

G18d2 TGGAATCATGACCGGTACATCAAAAGTTTATGCCATCTGTGGAGCTATCCGTGGTATGGG 240

L11C15 TGGAATCATGACCGGTACATCAAAAGTTTATGCCATCTGTGGAGCTATCCGTGGTATGGG 239

G10G4 TGAATCAGATGATTGCATTGCCCGTTTGGCTAAACGTGACAACATTTTGACAAAGAACTA 296

GW3A12 TGAATCAGATGATTGCATTGCCCGTTTGGCTAAACGTGACAACATTTTGACAAAGAACTA 300

G12D4 TGAATCAGATGATTGCATTGCCCGTTTGGCTAAACGTGACAACATTTTGACAAAGAACTA 299

G18d2 TGAATCAGATGATTGCATTGCCCGTTTGGCTAAACGTGACAACATTTTGACAAAGAACTA 300

L11C15 TGAATCAGATGATTGCATTGCCCGTTTGGCTAAACGTGACAACATTTTGACAAAGAACTA 299

G10G4 T**TGA**ATTGATTTTTATCGGTCAACACATTAACAATGTGAAAAAACATCAATCGTTTAAGA 356

GW3A12 T**TGA**ATTGATTTTTATCGGTCAACACATTAACAATGTGAAAAAACATCAATCGTTTAAGA 360

G12D4 T**TGA**ATTGATTTTTATCGGTCAACACATTAACAATGTGAAAAAACATCAATCGTTTAAGA 359

G18d2 T**TGA**ATTGATTTTTATCGGTCAACACATTAACAATGTGAAAAAACATCAATCGTTTAAGA 360

L11C15 T**TGA**ATTGATTTTTATCGGTCAACACATTAACAATGTGAAAAAACATCAATCGTTTAAGG 359

G10G4 A**A**TAAAAATTAATTGATTTTTTTTTATGCC------- 386

GW3A12 A**A**TAAAAATTAATTGATTTTTTTTTATGTCAAAAACT 397

G12D4 A**A**TAAAAATTAATTGATTTTTTTTTATGTCAAAAACT 396

G18d2 A**A**TAAAAATTAATTGATTTTTTTTTATGTCAAAAGCT 397

L11C15 T-CAAAAA**C**T--------------------------- 368

**X**

G15G3 ------------------------------------------------------------

GW5A3 ------------------------------------------------------------

GW15C10 ------------------------------------------------------------

GW15H12 ------------------------------------------------------------

GW3D9 ------------------------------------------------------------

GW18E4 -----------------------AGAGTTTGCATTTTTCATCGTTTCCGTTCTTTTCTTA 37

Lg2E9 ---------------------AAAGAGTTTGCATTTTTCATCGTTTCCGTTCTTTTCTTA 39

S14C8 ------------------------------------------------------------

G8G3 ------------------------------------------------------------

GW12H10 ------------------------------------------------------------

GW13D2 ------------------------------------------------------------

L1G9 --------------------------------------------------------CTTA 4

G14A10 -----------------------------------------------------TTTCTTA 7

G18F9 TTCAAATTGGTGAACTGAGGCAAAGAGTTTGCATTTTTCATCGTTTCCGTTCTTTTCTTA 60

G12E1 ---------------------------------------------------TTTTTCTTA 9

G15G3 ------------------------------------------------------------

GW5A3 -------------------**G**ACAAAAAAACGTCGTAACGGAGGACGTTGCAAACATAACC 41

GW15C10 ---------------AG**ATG**ACAAAAAAACGTCGTAACGGAGGACGTTGCAAACATAACC 45

GW15H12 ---------------AG**ATG**ACAAAAAAACGTCGTAACGGAGGACGTTGCAAACATAACC 45

GW3D9 ------------------**TG**ACAAAAAAACGTCGTAACGGAGGACGTTGCAAACATAACC 42

GW18E4 GTAACAGGCCAATCAAG**ATG**ACAAAAAAACGTCGTAACGGAGGACGTTGCAAACATAACC 97

Lg2E9 GTAACAGGCCAATCAAG**ATG**ACAAAAAAACGTCGTAACGGAGGACGTTGCAAACATAACC 99

S14C8 GTAACAGGCCAATCAAG**ATG**ACAAAAAAACGTCGTAACGGAGGACGTTGCAAACATAACC 60

G8G3 GTAACAGGCCAATCAAG**ATG**ACAAAAAAACGTCGTAACGGAGGACGTTGCAAACATAACC 60

GW12H10 ------GGCCAATCAAG**ATG**ACAAAAAAACGTCGTAACGGAGGACGTTGCAAACATAACC 54

GW13D2 -------GCCAATCAAG**ATG**ACAAAAAAACGTCGTAACGGAGGACGTTGCAAACATAACC 53

L1G9 GTAACAGGCCAATCAAG**ATG**ACAAAAAAACGTCGTAACGGAGGACGTTGCAAACATAACC 64

G14A10 GTAACAGGCCAATCAAG**ATG**ACAAAAAAACGTCGTAACGGAGGACGTTGCAAACATAACC 67

G18F9 GTAACAGGCCAATCAAG**ATG**ACAAAAAAACGTCGTAACGGAGGACGTTGCAAACATAACC 120

G12E1 GTAACAGGCCAATCAAG**ATG**ACAAAAAAACGTCGTAACGGAGGACGTTGCAAACATAACC 69

G15G3 -------TGTAAAGCCGGTTCGCTGTACAAACTGTGCAAGGTGCGTCCCAAAGGATAAGG 53

GW5A3 GTGGCCATGTAAAGCCGGTTCGCTGTACAAACTGTGCAAGGTGCGTCCCAAAGGATAAGG 101

GW15C10 GTGGCCATGTAAAGCCGGTTCGCTGTACAAACTGTGCAAGGTGCGTCCCAAAGGATAAGG 105

GW15H12 GTGGCCATGTAAAGCCGGTTCGCTGTACAAACTGTGCAAGGTGCGTCCCAAAGGATAAGG 105

GW3D9 GTGGCCATGTAAAGCCGGTTCGCTGTACAAACTGTGCAAGGTGCGTCCCAAAGGATAAGG 102

GW18E4 GTGGCCATGTAAAGCCGGTTCGCTGTACAAACTGTGCAAGGTGCGTCCCAAAGGATAAGG 157

Lg2E9 GTGGCCATGTAAAGCCGGTTCGCTGTACAAACTGTGCAAGGTGCGTCCCAAAGGATAAGG 159

S14C8 GTGGCCATGTAAAGCCGGTTCGCTGTACAAACTGTGCAAGGTGCGTCCCAAAGGATAAGG 120

G8G3 GTGGCCATGTAAAGCCGGTTCGCTGTACAAACTGTGCAAGGTGCGTCCCAAAGGATAAGG 120

GW12H10 GTGGCCATGTAAAGCCGGTTCGCTGTACAAACTGTGCAAGGTGCGTCCCAAAGGATAAGG 114

GW13D2 GTGGCCATGTAAAGCCGGTTCGCTGTACAAACTGTGCAAGGTGCGTCCCAAAGGATAAGG 113

L1G9 GTGGCCATGTAAAGCCGGTTCGCTGTACAAACTGTGCAAGGTGCGTCCCAAAGGATAAGG 124

G14A10 GTGGCCATGTAAAGCCGGTTCGCTGTACAAACTGTGCAAGGTGCGTCCCAAAGGATAAGG 127

G18F9 GTGGCCATGTAAAGCCGGTTCGCTGTACAAACTGTGCAAGGTGCGTCCCAAAGGATAAGG 180

G12E1 GTGGCCATGTAAAGCCGGTTCGCTGTACAAACTGTGCAAGGTGCGTCCCAAAGGATAAGG 129

G15G3 CAATCAAAAAGTTTGTTATTCGTAACATTGTTGAAGCTGCAGCTGTCCGTGATATCACTG 113

GW5A3 CAATCAAAAAGTTTGTTATTCGTAACATTGTTGAAGCTGCAGCTGTCCGTGATATCACTG 161

GW15C10 CAATCAAAAAGTTTGTTATTCGTAACATTGTTGAAGCTGCAGCTGTCCGTGATATCACTG 165

GW15H12 CAATCAAAAAGTTTGTTATTCGTAACATTGTTGAAGCTGCAGCTGTCCGTGATATCACTG 165

GW3D9 CAATCAAAAAGTTTGTTATTCGTAACATTGTTGAAGCTGCAGCTGTCCGTGATATCACTG 162

GW18E4 CAATCAAAAAGTTTGTTATTCGTAACATTGTTGAAGCTGCAGCTGTCCGTGATATCACTG 217

Lg2E9 CAATCAAAAAGTTTGTTATTCGTAACATTGTTGAAGCTGCAGCTGTCCGTGATATCACTG 219

S14C8 CAATCAAAAAGTTTGTTATTCGTAACATTGTTGAAGCTGCAGCTGTCCGTGATATCACTG 180

G8G3 CAATCAAAAAGTTTGTTATTCGTAACATTGTTGAAGCTGCAGCTGTCCGTGATATCACTG 180

GW12H10 CAATCAAAAAGTTTGTTATTCGTAACATTGTTGAAGCTGCAGCTGTCCGTGATATCACTG 174

GW13D2 CAATCAAAAAGTTTGTTATTCGTAACATTGTTGAAGCTGCAGCTGTCCGTGATATCACTG 173

L1G9 CAATCAAAAAGTTTGTTATTCGTAACATTGTTGAAGCTGCAGCTGTCCGTGATATCACTG 184

G14A10 CAATCAAAAAGTTTGTTATTCGTAACATTGTTGAAGCTGCAGCTGTCCGTGATATCACTG 187

G18F9 CAATCAAAAAGTTTGTTATTCGTAACATTGTTGAAGCTGCAGCTGTCCGTGATATCACTG 240

G12E1 CAATCAAAAAGTTTGTTATTCGTAACATTGTTGAAGCTGCAGCTGTCCGTGATATCACTG 189

G15G3 AAGCTTCAGTTTACACTGCTTACGTGCTCCCCAAGTTGTACGCAAAACTTCATTATTGCG 173

GW5A3 AAGCTTCAGTTTACACTGCTTACGTGCTCCCCAAGTTGTACGCAAAACTTCATTATTGCG 221

GW15C10 AAGCTTCAGTTTACACTGCTTACGTGCTCCCCAAGTTGTACGCAAAACTTCATTATTGCG 225

GW15H12 AAGCTTCAGTTTACACTGCTTACGTGCTCCCCAAGTTGTACGCAAAACTTCATTATTGCG 225

GW3D9 AAGCTTCAGTTTACACTGCTTACGTGCTCCCCAAGTTGTACGCAAAACTTCATTATTGCG 222

GW18E4 AAGCTTCAGTTTACACTGCTTACGTGCTCCCCAAGTTGTACGCAAAACTTCATTATTGCG 277

Lg2E9 AAGCTTCAGTTTACACTGCTTACGTGCTCCCCAAGTTGTACGCAAAACTTCATTATTGCG 279

S14C8 AAGCTTCAGTTTACACTGCTTACGTGCTCCCCAAGTTGTACGCAAAACTTCATTATTGCG 240

G8G3 AAGCTTCAGTTTACACTGCTTACGTGCTCCCCAAGTTGTACGCAAAACTTCATTATTGCG 240

GW12H10 AAGCTTCAGTTTACACTGCTTACGTGCTCCCCAAGTTGTACGCAAAACTTCATTATTGCG 234

GW13D2 AAGCTTCAGTTTACACTGCTTACGTGCTCCCCAAGTTGTACGCAAAACTTCATTATTGCG 233

L1G9 AAGCTTCAGTTTACACTGCTTACGTGCTCCCCAAGTTGTACGCAAAACTTCATTATTGCG 244

G14A10 AAGCTTCAGTTTACACTGCTTACGTGCTCCCCAAGTTGTACGCAAAACTTCATTATTGCG 247

G18F9 AAGCTTCAGTTTACACTGCTTACGTGCTCCCCAAGTTGTACGCAAAACTTCATTATTGCG 300

G12E1 AAGCTTCAGTTTACACTGCTTACGTGCTCCCCAAGTTGTACGCAAAACTTCATTATTGCG 249

G15G3 TATCATGCGCTATTCATTCAAAGGTCGTTCGTAATCGATCGAAAGAGGCCCGCAGAATCC 233

GW5A3 TATCATGCGCTATTCATTCAAAGGTCGTTCGTAATCGATCGAAAGAGGCCCGCAGAATCC 281

GW15C10 TATCATGCGCTATTCATTCAAAGGTCGTTCGTAATCGATCGAAAGAGGCCCGCAGAATCC 285

GW15H12 TATCATGCGCTATTCATTCAAAGGTCGTTCGTAATCGATCGAAAGAGGCCCGCAGAATCC 285

GW3D9 TATCATGCGCTATTCATTCAAAGGTCGTTCGTAATCGATCGAAAGAGGCCCGCAGAATCC 282

GW18E4 TATCATGCGCTATTCATTCAAAGGTCGTTCGTAATCGATCGAAAGAGGCCCGCAGAATCC 337

Lg2E9 TATCATGCGCTATTCATTCAAAGGTCGTTCGTAATCGATCGAAAGAGGCCCGCAGAATCC 339

S14C8 TATCATGCGCTATTCATTCAAAGGTCGTTCGTAATCGATCGAAAGAGGCCCGCAGAATCC 300

G8G3 TATCATGCGCTATTCATTCAAAGGTCGTTCG**C**AATCGATCGAAAGAGGCCCGCAGAATCC 300

GW12H10 TATCATGCGCTATTCATTCAAAGGTCGTTCGTAATCGATCGAAAGAGGCCCGCAGAATCC 294

GW13D2 TATCATGCGCTATTCATTCAAAGGTCGTTCGTAATCGATCGAAAGAGGCCCGCAGAATCC 293

L1G9 TATCATGCGCTATTCATTCAAAGGTCGTTCGTAATCGATCGAAAGAGGCCCGCAGAATCC 304

G14A10 TATCATGCGCTATTCATTCAAAGGTCGTTCGTAATCGATCGAAAGAGGCCCGCAGAATCC 307

G18F9 TATCATGCGCTATTCATTCAAAGGTCGTTCGTAATCGATCGAAAGAGGCCCGCAGAATCC 360

G12E1 TATCATGCGCTATTCATTCAAAGGTCGTTCGTAATCGATCGAAAGAGGCCCGCAGAATCC 309

G15G3 GAACTCCACCACAACGTGCATTTGCACGTGATCCAAAGCAAGCTCAACAAGCTAGAAAG**T** 293

GW5A3 GAACTCCACCACAACGTGCATTTGCACGTGATCCAAAGCAAGCTCAACAAGCTAGAAAG**T** 341

GW15C10 GAACTCCACCACAACGTGCATTTGCACGTGATCCAAAGCAAGCTCAACAAGCTAGAAAG**T** 345

GW15H12 GAACTCCACCACAACGTGCATTTGCACGTGATCCAAAGCAAGCTCAACAAGCTAGAAAG**T** 345

GW3D9 GAACTCCACCACAACGTGCATTTGCACGTGATCCAAAGCAAGCTCAACAAGCTAGAAAG**T** 342

GW18E4 GAACTCCACCACAACGTGCATTTGCACGTGATCCAAAGCAAGCTCAACAAGCTAGAAAG**T** 397

Lg2E9 GAACTCCACCACAACGTGCATTTGCACGTGATCCAAAGCAAGCTCAACAAGCTAGAAAG**T** 399

S14C8 GAACTCCACCACAACGTGCATTTGCACGTGATCCAAAGCAAGCTCAACAAGCTAGAAAG**T** 360

G8G3 GAACTCCACCACAACGTGCATTTGCACGTGATCCAAAGCAAGCTCAACAAGCTAGAAAG**T** 360

GW12H10 GAACTCCACCACAACGTGCATTTGCACGTGATCCAAAGCAAGCTCAACAAGCTAGAAAG**T** 354

GW13D2 GAACTCCACCACAACGTGCATTTGCACGTGATCCAAAGCAAGCTCAACAAGCTAGAAAG**T** 353

L1G9 GAACTCCACCACAACGTGCATTTGCACGTGATCCAAAGCAAGCTCAACAAGCTAGAAAG**T** 364

G14A10 GAACTCCACCACAACGTGCATTTGCACGTGATCCAAAGCAAGCTCAACAAGCTAGAAAG**T** 367

G18F9 GAACTCCACCACAACGTGCATTTGCACGTGATCCAAAGCAAGCTCAACAAGCTAGAAAG**T** 420

G12E1 GAACTCCACCACAACGTGCATTTGCACGTGATCC-------------------------- 343

G15G3 **AA**AAAATTTCAAGTGCTTTGTCTTTTAACACAATGTTAAAAAAACAATAAACAGAATATA 353

GW5A3 **AA**AAAATTTCAAGTGCTTTGTCTTTTAACACAATGTTAAAAAAACAATAAACAGAATATA 401

GW15C10 **AA**AAAATTTCAAGTGCTTTGTCTTTTAACACAATGTTAAAAAAACAATAAACAGAATATA 405

GW15H12 **AA**AAAATTTCAAGTGCTTTGTCTTTTAACACAATGTTAAAAAAACAATAAACAGAATATA 405

GW3D9 **AA**AAAATTTCAAGTGCTTTGTCTTTTAACACAATGTTAAAAAAACAATAAACAGAATATA 402

GW18E4 **AA**AAAATTTCAAGTGCTTTGTCTTTTAACACAATGTTAAAAAAACAATAAACAGAATATA 457

Lg2E9 **AA**AAAATTTCAAGTGCTTTGTCTTTTAACACAATGTTAAAAAAACAATAAACAGAATATA 459

S14C8 **AA**AAAATTTCAAGTGCTTTGTCTTTTAACACAATGTTAAAAAAACAATAAACAGAATATA 420

G8G3 **AA**AAAATTTCAAGTGCTTTGTCTTTTAACACAATGTTAAAAAAACAATAAACAGAATATA 420

GW12H10 **AA**AAAATTTCAAGTGCTTTGTCTTTTAACACAATGTTAAAAAAACAATAAACAGAATATA 414

GW13D2 **AA**AAAATTTCAAGTGCTTTGTCTTTTAACACAATGTTAAAAAAACAATAAACAGAATATA 413

L1G9 **AA**AAAATTTCAAGTGCTTTGTCTTTTAACACAATGTTAAAAAAACAATAAACAGAATATA 424

G14A10 **AA**AAAATTTCAAGTGCTTTGTCTTTTAACACAATGTTAAAAAAACAATAAACAGAATATA 427

G18F9 **AA**AAAATTTCAAGTGCTTTGTCTTTTAACACAATGTTAAAAAAACAATAAACAGAATATA 480

G12E1 ------------------------------------------------------------

G15G3 AATTCCTTG 362

GW5A3 AATTCCTTG 410

GW15C10 AATTCCTTG 414

GW15H12 AATTCCTTG 414

GW3D9 AATTCCTT- 410

GW18E4 AATTCCTTG 466

Lg2E9 AATTCCTTG 468

S14C8 AATTCCTT- 428

G8G3 AATTCCGT- 428

GW12H10 AAT------ 417

GW13D2 AAT------ 416

L1G9 AATTCCT-- 431

G14A10 AATTCCTGC 436

G18F9 AATT----- 484

G12E1 ---------

**Y**

G15G11 GCACGGACACGTGAATTAATTCTTCGATTAATTTCAACAAATAAATTCATTCGAA**ATG**GA 60

GW13A10 GCACGGACACGTGAATTAATTCTTCGATTAATTTCAACAAATAAATTCATTCGAA**ATG**GA 60

GW15E11 GCACGGACACGTGAATTAATTCTTCGATTAATTTCAACAAATAAATTCATTCGAA**ATG**GA 60

GW11A6 GCACGGACACGTGAATTAATTCTTCGATTAATTTCAACAAATAAATTCATTCGAA**ATG**GA 60

GW20B9 GCACGGACACGTGAATTAATTCTTCGATTAATTTCAACAAATAAATTCATTCGAA**ATG**GA 60

S18C12 GCACGGACACGTGAATTAATTCTTCGATTAATTTCAACAAATAAATTCATTCGAA**ATG**GA 60

GW4F8 GCACGGACACGTGAATTAATTCTTCGATTAATTT**T**AACAAATAAATTCATTCGAA**ATG**GA 60

GW15E3 GCACGGACACGTGAATTAATTCTTCGATTAATTTCAACAAATAAATTCATTCGAA**ATG**GA 60

G15G11 AAAACCAGTTGTTTTGGCACGAGTCATCAAAGTTTTGGGCCGTACAGGATCCCAAGGACA 120

GW13A10 AAAACCAGTTGTTTTGGCACGAGTCATCAAAGTTTTGGGCCGTACAGGATCCCAAGGACA 120

GW15E11 AAAACCAGTTGTT**C**TGGCACGAGTCATCAAAGTTTTGGGCCGTACAGGATCCCAAGGACA 120

GW11A6 AAAACCAGTTGTT**C**TGGCACGAGTCATCAAAGTTTTGGGCCGTACAGGATCCCAAGGACA 120

GW20B9 AAAACCAGTTGTT**C**TGGCACGAGTCATCAAAGTTTTGGGCCGTACAGGATCCCAAGGACA 120

S18C12 AAAACCAGTTGTTTTGGCACGAGTCATCAAAGTTTTGGGCCGTAC**G**GGATCCCAAGGACA 120

GW4F8 AAAACCAGTTGTTTTGGCACGAGTCATCAAAGTTTTGGGCCGTACAGGATCCCAAGGACA 120

GW15E3 AAAACCAGTTGTTTTGGCACGAGTCAT**T**AAAGTTTTGGGCCGTACAGGATCCCAAGGACA 120

G15G11 ATGTACCCAAGTTAAAGTTGAATT**C**ATTGGCGAACAAAATCGTCAAATCATCCGTAACGT 180

GW13A10 ATGTACCCAAGTTAAAGTTGAATT**C**ATTGGCGAACAAAATCGTCAAATCAT**T**CGTAACGT 180

GW15E11 ATGTACCCAAGTTAAAGTTGAATTTATTGGCGAACAAAATCGTCAAATCATCCGTAACGT 180

GW11A6 ATGTACCCAAGTTAAAGTTGAATTTATTGGCGAACAAAATCGTCAAATCATCCGTAACGT 180

GW20B9 ATGTACCCAAGTTAAAGTTGAATTTATTGGCGAACAAAATCGTCAAATCATCCGTAACGT 180

S18C12 ATGTACCCAAGTTAAAGTTGAATTTATTGGCGAACAAAATCGTCAAATCATCCGTAACGT 180

GW4F8 ATGTACCCAAGTTAAAGTTGAATTTATTGGCGAACAAAATCGTCAAATCATCCGTAACGT 180

GW15E3 ATGTAC**T**CAAGTTAAAGTTGAATTTATTGGCGAACAAAATCGTCAAATCATCCGTAACGT 180

G15G11 TAAAGGTCCAGTTCGTGAAGGTGATATCCTCACATTGTTGGAATCTGAACGTGAAGCTAG 240

GW13A10 TAAAGGTCCAGTTCGTGAAGGTGATATCCTCACATTGTTGGAATCTGAACGTGAAGCTAG 240

GW15E11 TAAAGGTCCAGTTCGTGAAGGTGATATCCTCACATTGTTGGAATCTGAACGTGAAGCTAG 240

GW11A6 TAAAGGTCCAGTTCGTGAAGGTGATATCCTCACATTGTTGGAATCTGAACGTGAAGCTAG 240

GW20B9 TAAAGGTCCAGTTCGTGAAGGTGATATCCTCACATTGTTGGAATCTGAACGTGAAGCTAG 240

S18C12 TAAAGGTCCAGTTCGTGAAGGTGATATCCTCACATTGTTGGAATCTGAACGTGAAGCTAG 240

GW4F8 TAAAGGTCCAGTTCGTGAAGGTGATATCCTCACATTGTTGGAATCTGAACGTGAAGC**A**AG 240

GW15E3 TAAAGGTCCAGTTCGTGAAGGTGATATCCTCACATTGTTGGAATCTGAACGTGAAGCTAG 240

G15G11 AAGATTACGT**TAA**TTTCAATCATTCAACAGTAATTTTTCTACAATAAGATGTT**T**CTCAAA 300

GW13A10 AAGATTACGT**TAA**TTTCAATCATTCAACAGTAATTTTTCTACAATAAGATGTT**T**CTCAAA 300

GW15E11 AAGATTACGT**TAA**TTTCAATCATTCAACAGTAATTTTTCTACAATAAGATGTT-CTCAAA 299

GW11A6 AAGATTACGT**TAA**TTTCAATCATTCAACAGTAATTTTTCTACAATAAGATGTT-CTCAAA 299

GW20B9 AAGATTACGT**TAA**TTTCAATCATTCAACAGTAATTTTTCTACAATAAGATGTT-CTCAAA 299

S18C12 AAGATTACGT**TAA**TTTCAA**C**CATTCAACAGTAATTTTTCTACAATAAGATGTT-CTCAAA 299

GW4F8 AAGATTACGT**TAA**TTTCAATCATTCAACAGTAATTTTTCTACAATAAGATGTT-CTCAAA 299

GW15E3 AAGATTACGT**TAA**TTTCAA**C**CATTCAACAGTAATTTTTCTACAATAAGATGTT-CTCAAA 299

G15G11 TGTTATTCATCGTCAACAACA**GCA**ACAAAGCACCAGCAGTTGAAGATGCCTGAGCACAAT 360

GW13A10 TGTTATTCATCGTCAACAACA**ACA**ACAAAGCACCAGCAGTTGAAGATGCCTGAGCACAAT 360

GW15E11 TGTTATTCATCGTCATCAACA---ACAAAGCAC**T**AGCAGTTGAAGATGCCTGAGCACAAT 356

GW11A6 TGTTATTCATCGTCATCAACA---ACAAAGCAC**T**AGCAGTTGAAGATGCCTGAGCACAAT 356

GW20B9 TGTTATTCATCGTCATCAACA---ACAAAGCAC**T**AGCAGTTGAAGATGCCTGAGCACAAT 356

S18C12 TGTTATTCATCGTCATCAACA---ACAAAGCAC**T**AGCAGTTGAAGATGCCTGAGCACAAT 356

GW4F8 TGTTATTCATCGTCAACAACA**ACA**ACAAAGCACCAGCAGTTGAAGATGCCT**AG**GCACAAT 359

GW15E3 TGTTATTCATCGTCAACAACA**ACA**ACAAAGCACCAGCAGTTGAAGATGCCT**AG**GCACAAT 359

G15G11 GAAATTAAACAAACTTCAACCAATACCA**T**CATTGTCCAGATGTGCTTAAAATAAAATATT 420

GW13A10 GAAATTAAACAAACTTCAACCAATACCACCATTGTCCAGATGTGCTTAAAATAAAATATT 420

GW15E11 GAAATTAAACAAACTT**T**AACCAATACCACCATTGTCCAGATGTGCTTAAAATAAAATATT 416

GW11A6 GAAATTAAACAAACTT**T**AACCAATACCACCATTGTCCAGATGTGCTTAAAATAAAATATT 416

GW20B9 GAAATTAAACAAACTT**T**AACCAATACCACCATTGTCCAGATGTGCTTAAAATAAAATATT 416

S18C12 GAAATTAAACAAACTT**T**AACCAATACCACCATTGTCCAGATGTGCTTAAAATAAAATATT 416

GW4F8 GAAATTAAACAAACTTCAACCAATACCACCATTGTCCAG**C**TGTGCTTAAAATAAAATATT 419

GW15E3 GAAATTAAACAAA**T**TTCAACCAATACCAC**T**ATTGTCCAGATGTGCTTAAAATAAAATATT 419

G15G11 CATTTGATGAATCC--------------------------------- 434

GW13A10 CATTTGATGAATCCAAAGTGAAAT----------------------- 444

GW15E11 CATTTGATGAATCCAAAGTGAAATAAATTTTGTGTTTTTCGTTTTTC 463

GW11A6 CATTTGATGAATCCAAAGTG--------------------------- 436

GW20B9 CATTTGATGAATCC--------------------------------- 430

S18C12 CATTTGATGAATCC--------------------------------- 430

GW4F8 CATTTGATGAATCCAAAGTGAAATAAATTTTGTGTTTTTCGTTTTTC 466

GW15E3 CATTTGATGAATCCAAAGTGAAATAAATTTTGTGTTTTTCGTTTTTC 466

Figure S7. Sequence alignments of cDNAs coding for ribosomal proteins L17 (A), L18 (B), L18A (C), L21 (D), L23A (E), L26E (F), L30 (G), L34 (H), L35 (I), L35A (J), L36-P1 (K), L37A (L), L38 (M), L44E (N), S3 (O), S3A (P), S5E (Q), S9 (R), S10 (S), S13 (T), S15A (U), S19 (V), S21 (W), S26 (X), and S28 (Y). Nonsynonymous substitutions are in red while synonymous substitutions are in blue. Substitutions outside of the coding regions are in green. Indels are bold. Start and stop codons are in bold and underlined.
