# Supplementary material for: Unusual conservation among genes encoding small secreted salivary gland proteins from a gall midge
Source: BMC Evol Biol. 2010 Sep 28;10:296. doi: 10.1186/1471-2148-10-296 (PMC2955719; doi:10.1186/1471-2148-10-296)
Supplement: Additional file 8 — Table S1: Primers used for PCR reactions. [file 1471-2148-10-296-S8.DOC]

Table S1. Primers for quantitative real-time PCR^1^

| **Gene** | **Direction** | **Sequence** | **Amplicon size** |
| --- | --- | --- | --- |
| SSSGP-1A | Forward | 5'-GCCAACAGCCCAATCAAACC | 109 bp |
|  | Reverse | 5'-GGCAGGTCCATTAGAATTTCCG |  |
| SSSGP-1B1 | Forward | 5'-CCAGCAGTCGAGCCAAGC | 183 bp |
|  | Reverse | 5'-TGGTTTTGCCTTTGATGGTTTAGC |  |
| SSSGP-1C1 | Forward | 5'-CTTGACGATGGTGAGCACTATTTC | 93 bp |
|  | Reverse | 5'-CGGTACGGCTGCTTCATTAGG |  |
| SSSGP-1C2 | Forward | 5'-CTTGACGAAGATGAGCACTGG | 84 bp |
|  | Reverse | 5'-TGCTTCATTAGGGCCATTATCATC |  |
| SSSGP-1D1 | Forward | 5'-AAACCGCACACAGCCCAAC | 105 bp |
|  | Reverse | 5'-TCCATTCTGCCCTCCTAAATTTCC |  |
| SSSGP-1E1 | Forward | 5'-CCAGCACCGCAAACAGTCC | 99 bp |
|  | Reverse | 5'-TTCAGTCCTCGTTGGCATTCC |  |
| Gene G9E4 | Forward | 5'-ATGTGTTCCTGCTCCGTTACC | 94 bp |
|  | Reverse | 5'-TAGGCATGGAACCGATGATGG |  |
| Gene G10C9 | Forward | 5'-AACTTCCACTCTTCCTGTTCCA | 142 bp |
|  | Reverse | 5'-AAGAGTTGCTGCTTGACTTGGA |  |

^1^Except for genes *SSSGP-1A1* and *1A2* that share a common primer pair because of identical coding sequences, primers specific for each gene were designed. G9E4 and G10C9 represent two genes that have identical or very similar sequences with cDNAs G9E4 and G10C9 in the coding region. These two genes belong to the *SSSGP-31* group (Fig. S1B).
